# Expanding Natural Diversity: Tailored Enrichment of the 8,12-Sesquiterpenoid Lactone Chemical Space through Divergent Synthesis

**DOI:** 10.1021/acs.orglett.4c01374

**Published:** 2024-05-23

**Authors:** Vera P. Demertzidou, Maria Kourgiantaki, Alexandros L. Zografos

**Affiliations:** Aristotle University of Thessaloniki, Department of Chemistry, Laboratory of Organic Chemistry, Thessaloniki 54124, Greece

## Abstract

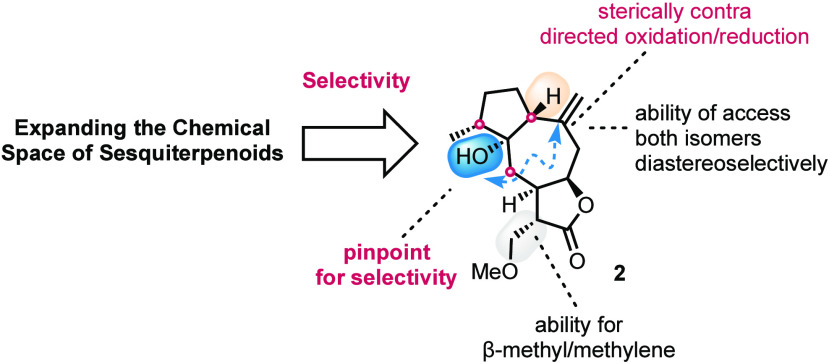

The divergent synthesis of a non-natural 8,12-sesquiterpenoid lactone collection is described. The synthesis relies on a rationally designed guaianolide scaffold bearing a tertiary hydroxyl as the pinpoint for inducing its selective diversification. Key reactions include an unprecedented Suarez-type CH lactonization and a highly diastereoselective oxy-Cope/ene cascade that allows the introduction of three stereocenters in a single operation. Selective oxidative/reductive and redox neutral transformations follow to highlight the synthesis of naturally unpresented highly substituted 8,12-guaianolides.

Secondary metabolites are a significant source of drugs and drug leads.^1^ Gaining efficient access to their rich chemical diversity facilitates more immediate responses to societal health needs. With respect to this goal, divergent synthesis plays a leading role in enriching the chemical space by utilizing appropriate divergency scaffolds that mimic Nature’s biosynthetic logic.^2^ In practical terms though, divergent synthesis suffers from the difficulty of identifying and synthesizing the optimum divergency scaffold that can lead to maximum diversity. Focusing on this drawback, our group has initiated a campaign for identifying divergent scaffolds that cover the maximum chemical space possible.^3^ As part of this effort, the divergent synthesis of sesquiterpenoid lactones (SQLs), a highly diverse family that may play a pivotal role in discovering leads for anticancer drugs, has been targeted.^4^

The biological properties of SQLs are attributed to the highly electrophilic sites they bear, in the form of an α-methylene-γ-butenolide core and/or cyclopentenone and their special electronic and steric motifs.^5^ However, in addition to the well-established connection of numerous natural SQLs with important biological activities, their direct implementations as drugs are hampered by their high toxicity and poor pharmacological profiles.^4^ On the contrary, masking α-methylene-γ-butenolide moiety returned promising prodrug candidates currently in clinical trials as anticancer drugs.^4^ In contrast to the widely distributed chemical space of natural guaianolide SQLs (Scheme 1A), a rather limited number of potential stereochemical patterns have been synthetically accessed.

**Scheme 1:**
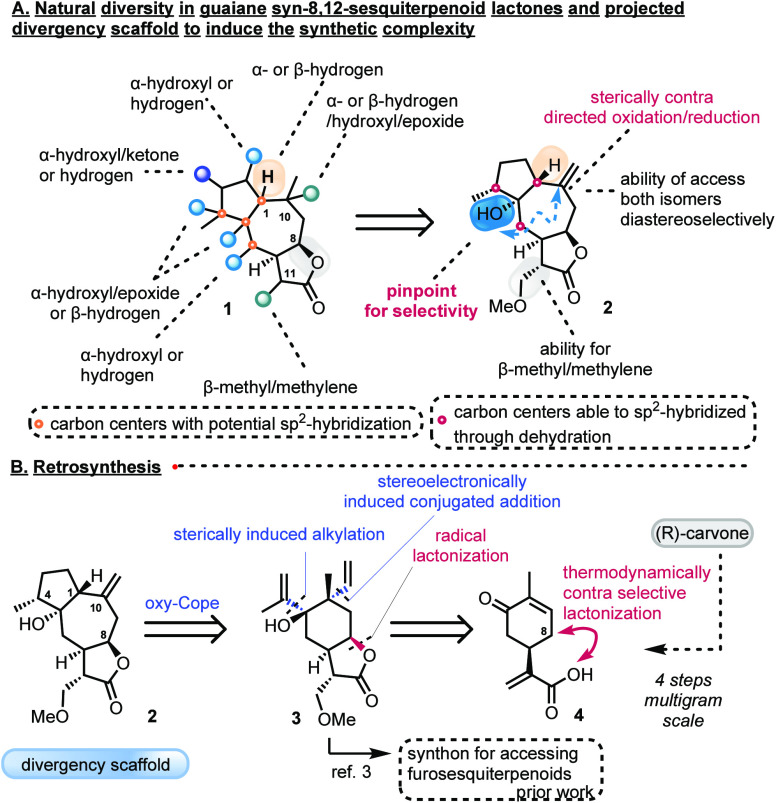
Natural Diversity and a Retrosynthetic Plan for Its Expansion

On the basis of these facts, we sought to move away from the trivial logic of targeting the highly potent natural products of the family^6^ and focusing on the less explored 8,12-sesquiterpenoids.^7^ Recognizing the scarcity of those members, compared to the isolated diversity of 6,12-isomers, we questioned whether it would be possible to enrich the sesquiterpenoid lactone chemical space by non-natural 8,12-congeners by utilizing an optimum divergency scaffold.^3^ Despite our extensive experience in the synthesis of furosesquiterpenoids^3^ and related congeners,^8^ extending this approach to access 8,12-SQLs poses several challenges. These primarily stem from the enhanced sterics introduced by the highly electrophilic *α-*methylene-γ-butenolide core and our objective of achieving access to all possible diastereoisomers across diverse positions of the carbocycle. Given these facts, we considered the synthesis of divergency scaffold **2** bearing a crucially positioned hydroxyl moiety as the pinpoint for achieving diverse selectivity. To achieve its synthesis, the oxy-Cope/ene reaction of an appropriately *syn*-substituted diene **3** was envisioned (Scheme 1B). The *syn* relation was expected to lock a sole chair conformation for the oxy-Cope reaction to deliver high diastereoselectivity for the ene reaction that follows. An early stage introduction of α-methylene-γ-butenolide core toward **3** was anticipated not only to ensure the stereoselective construction of the remaining tertiary stereocenters but also to produce more biologically relevant scaffolds toward the final goal.^9,10^ Further retrosynthetic analysis of **3** led to (*R*)-carvonic acid (**4**) and (*R*)-carvone as its direct potential precursors (Scheme 1B).

The synthesis began with the oxidation of (*R*)-carvone to (*R*)-carvonic acid (**4**) (Scheme 2). Despite the apparent simplicity of the described transformation, the reported methods were proven to be incapable of delivering reproducible yields of **4** in a multigram scale synthesis (<10% overall yield).^11^ This early failure prompted us to access **4** by a novel ene-chlorination of (*R*)-carvone, followed by successive oxidations. The mixing of TMSCl and oxone in the presence of (*R*)-carvone provided a 75% yield of allylic chloride, even at a 100 g scale.^12^ Quantitative hydrolysis of allylic chloride by 10% sodium bicarbonate in dioxane yielded allylic alcohol **5**, which was readily oxidized by PCC to afford aldehyde **6** in 47% yield. Synthesis of **4** was achieved following the Lindgren protocol to prepare ≤38 g of the product (35% overall yield over four steps) in a single batch. Alternatively, smaller batches were prepared by the direct oxidation of alcohol **5** to **4** by copper(II) bromide/TEMPO aerobic oxidation in 35% overall yield.^13^

**Scheme 2:**
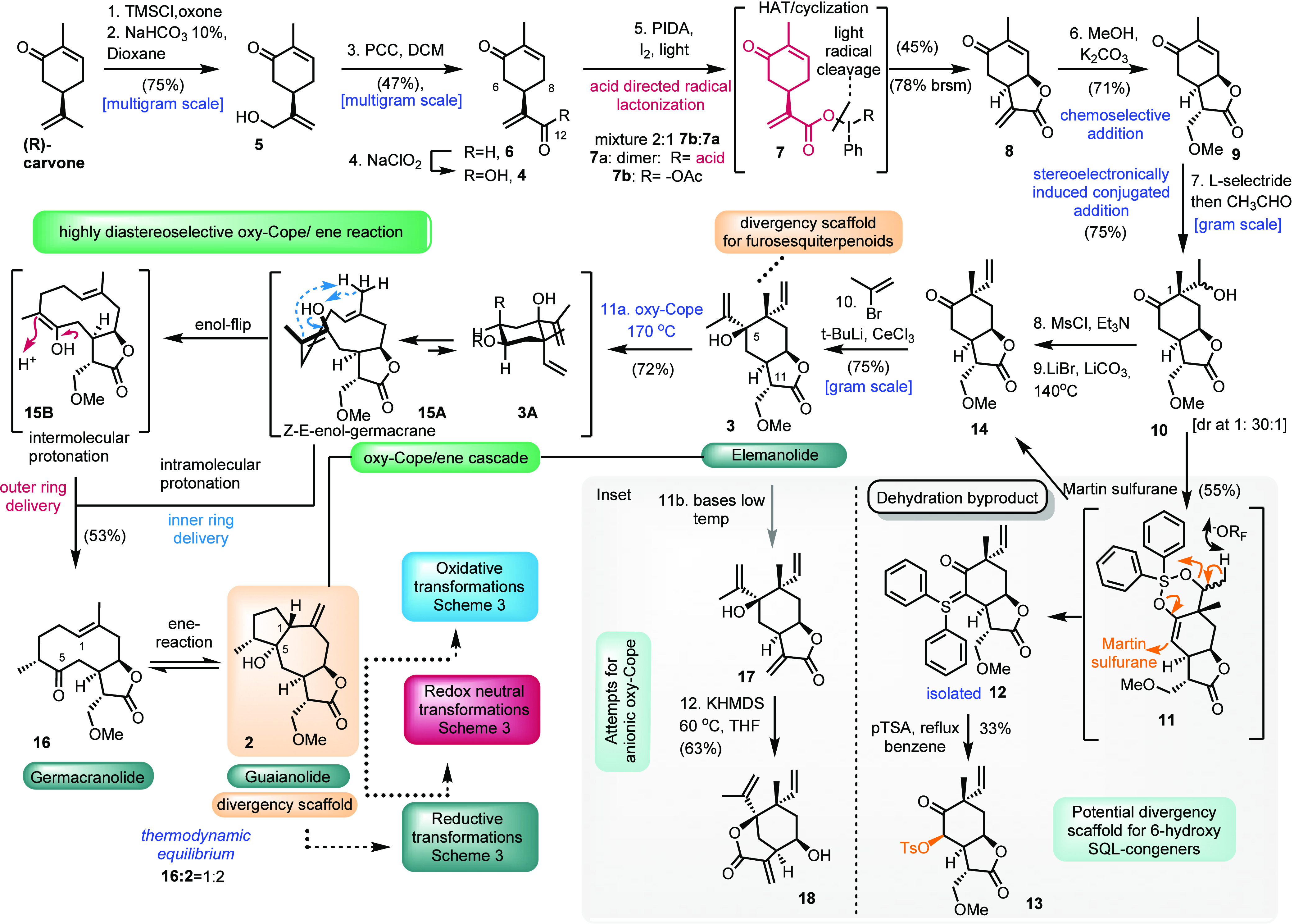
Gram Scale Synthetic Route to Divergency Scaffold **2**

With multigram quantities of (*R*)-carvonic acid (**4**) in our hands, we next turned our attention to the key direct *syn*-8,12-lactonization step. To scale this reaction up, the NBS radical allylic bromination/cyclization sequence to afford **8** previously developed by our group^3^ resulted in inconsistent results and low yields (28%). Attempts to optimize the yield by direct allylic oxidation^14^ and carboxyl as a directing group to induce CH lactonization by known protocols^15−17^ failed to provide the desired 8,12-lactone. Considering these facts, we turned our focus to achieving a carboxy radical-directed lactonization. Despite the lack of reported methods, acrylic acids under mild radical conditions were expected to survive the extrusion of carbon dioxide, based on their enhanced stability.^18^ Indeed, when carvonic acid **4** was allowed to react with an excess of PIDA and an equimolar amount of iodine under visible light irradiation, a highly chemoselective *syn* lactonization was witnessed, affording **8** in 45% yield (78% brsm) after a single recrystallization (Scheme 2). Monitoring the reaction mixture by ^1^H nuclear magnetic resonance (NMR) revealed hypervalent iodine conjugates **7a** and **7b** as the active to homolysis species (see the Supporting Information). The latter allows the directed hydrogen atom transfer only from the most stable allylic position, leading to its direct cyclization with the aid of iodine. With respect to the synthesis of **2**, lactone **8** was protected^3,19^ and reductively alkylated by L-selectride and acetaldehyde to afford **10**. The stereoelectronic effects of the *syn*-8,12-lactone element ensured the selectivity of acetaldehyde addition, providing high diastereocontrol in **10** (Scheme 2).^20^ Dehydration of **10** by the Burgess reagent at multigram scale resulted in nonreproducible yields of **14** and unreactive sulfonyl carbamates.^3^ After numerous attempts, we found that optimum yields were delivered by transforming **10** to its mesylate followed by LiBr, LiCO_3_ demesylation (52% over two steps). It is interesting to note that direct dehydration by Martin sulfurane resulted in product **14** in low yields (25%) along with sulfur ylide **12**. The latter can be efficiently transformed into **13** with PTSA, a potential 6-functionalized analogue of scaffold **14**, which did not proceed further.^21^

With an efficient gram scale access to **14**, serving as the divergency scaffold to all prior described routes,^3^ we set the stage for the introduction of the isopropenyl chain solely to the α-face of C5 of **3**. Early results indicated that the C5-β-epimeric compound was unable to initiate the key oxy-Cope reaction even under prolonged heating conditions. Once again, the early introduction of the lactone moiety ensured the stereoselective addition of the isopropenyl chain due to sterics, when the cerium 2-propenyl nucleophile was used (75%, ds > 20:1). Interestingly, the addition of harder nucleophiles such as lithium and magnesium 2-propenyl reagents failed to produce satisfactory yields of **3**, due to their preferential attack on the lactone moiety, providing stable lactole products.^12^

As a confirmation of our expectations, heating **3** at 170 °C produced stereoselectively methoxy-protected germacranolide **16** along with guaianolide **2** as a 1:2 mixture in 72% yield. Subjecting chromatographically pure guaianolide **2** to heating at 170 °C returned **16** and **2** in the same 1:2 ratio, demonstrating their thermal equilibration. The high selectivity observed in guaianolide **2** is attributed to **3A**, as it is the only sterically viable conformer for initiating the oxy-Cope/ene reaction. The sole isolation of α-methyl germacranolide **16** points toward either the intramolecular inner ring protonation of the initially formed enol **15A** or the enol flip to **15B** followed by its intermolecular outer ring protonation. The ene reaction that follows produces the expected **2** as the sole isomer, bearing naturally unfavorable β-H at position C1. Contrary to the thermal oxy-Cope/ene reaction, anionic oxy-Cope reaction using KHMDS failed to produce germacranolide **16** but did provide translactonized product **18** that resembles irregular natural elemanolides (Scheme 2).^22^ Interestingly, addition of a mild base to **3** (Et_3_N and K_2_CO_3_), even at low temperatures, resulted in the rapid deprotection of the methoxy group to **17** by hydroxyl-assisted deprotonation of C11, demonstrating the rather stable, nonflexible conformation posed by **3A**.

Providing scalable access to the guaianolide **2** core, we chose to enrich its chemical space by manipulating selectively positions C4, C5, and C10, which were found to be among the most pharmacophoric based on SAR and QSAR studies for their anticancer activity.^23^ Decoration of the guaianolide core of **2** was pursued following three main axes: reductive, redox neutral (dehydration), and oxidative at the initial stage of the transformation, aiming for optimal reaction selectivity (Scheme 3). The critically positioned tertiary hydroxyl at position C5 was projected as the pinpoint for inducing selectivity, according to earlier findings of our group.^8^ Then, the products were further elaborated to achieve the maximum diversity.

**Scheme 3:**
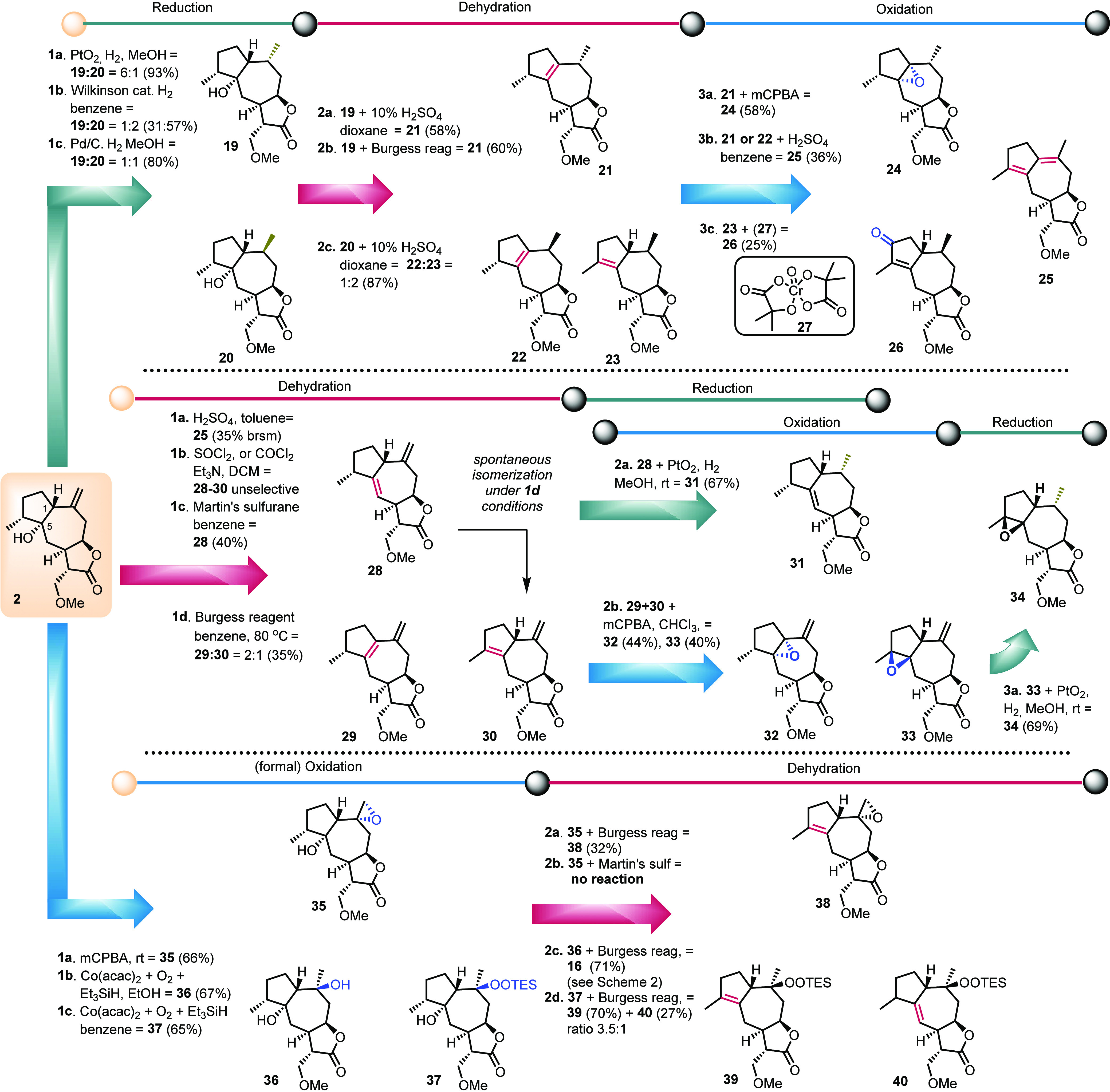
Inducing Selective Diversity from Scaffold **2**

The initial reduction of **2** with hydrogen under Adam’s catalysis resulted in a 6:1 mixture of compounds **19** and **20** (Scheme 3). Taking advantage of the hydroxyl at C5 allows us to overcome sterics, delivering the sterically contra **20** isomer as the major component when the Wilkinson catalyst was applied in the hydrogenation reaction. Diastereoisomers **19** and **20** can be further diverged by applying different sets of dehydration conditions. The Burgess reagent or 10% H_2_SO_4_ in dioxane on **19** selectively provided bridged alkene **21** in almost 60% yield. On the contrary, the reaction of **20** with 10% H_2_SO_4_ in dioxane led to the synthesis of **22** and **23** as a 2:1 mixture, though in a higher 87% yield (Scheme 3). Interestingly, when bridged alkenes **21** or **22** were treated with concentrated sulfuric acid in benzene, an oxidative process was witnessed to deliver compound **25** as a sole product in low yield. Testing **21** toward epoxidation with *m*-CPBA resulted in the synthesis of compound **24** in excellent diastereoselectivity. Finally, oxidation of **23** with the aid of chromium complex **27** led to the synthesis of compound **26** resembling 6-deoxogeigerin, the only known natural structure bearing a β-positioned bridgehead hydrogen atom (Scheme 3).

Attempts to initially dehydrate divergent scaffold **2** were found to be repeatedly unsuccessful or unselective under various conditions. Dehydration of **2** with the aid of Lewis acids or organic acids failed to initiate any reaction, while the use of concentrated sulfuric acid in toluene resulted in the quantitative formation of diene **25**. Thionyl chloride or sulfuryl chloride in the presence of different bases led to the unselective formation of alkenes **28**–**30**, potentially through the E1 elimination mechanism. On the contrary, Martin sulfurane reaction on **2** resulted in the synthesis of **28** selectively, albeit in low yield (14%), even after prolonged reaction times. A partially selective process was witnessed when the Burgess reagent was used, affording **29** and **30** in a 2:1 ratio. This partial selectivity was attributed to the spontaneous isomerization of **28** under the reaction conditions, verified by the existence of **28** when the Burgess reaction was terminated before the full consumption of the starting material. Unfortunately, despite the exclusion of **28** from the reaction mixture, **29** and **30** could not be separated and were used as a mixture for further elaboration. Fortuitously, both hydrogenation of **28** under Adam’s catalysis and epoxidation of the mixture of **29** and **30** provided the diastereoselective synthesis of **31**–**33**, respectively. Interestingly, the reduction of **33** once again highlights diastereoselectivity over the β-face of the alkene to deliver **34**.

Once again, taking advantage of the hydroxyl group at C5 allowed us to selectively diverge between both stereocenters of C10 when formal oxidative conditions were initially applied to **2**. Hydroxyl-directed epoxidation of **2** by *m*-CPBA allowed the sterically contra diastereoselective α-epoxidition to **35**. On the contrary, Mukaiyama hydration conditions provided only the sterically favored β-hydroxylation on the alkene to yield both hydroxy or hyperoxy **36** and **37** depending on the reaction conditions. Epoxide **35** can be selectively dehydrated under Burgess reagent conditions to provide compound **38** with excellent chemoselectivity. Surprisingly, testing compound **36** for dehydration under the Burgess reagent resulted in the formation of germacrane **16** (see Scheme 2). Peroxy analogue **37** provided the expected alkenes **39** and **40**.

Even though preliminary results show the direct *in vitro* deprotection of methoxy groups in a cell line culture,^19^ removal of the protecting group in chemical terms was attempted with the aid of TBAF. In every case, the reaction progressed smoothly even in the presence of the labile tertiary alcohol and epoxide (Scheme 4).

**Scheme 4:**
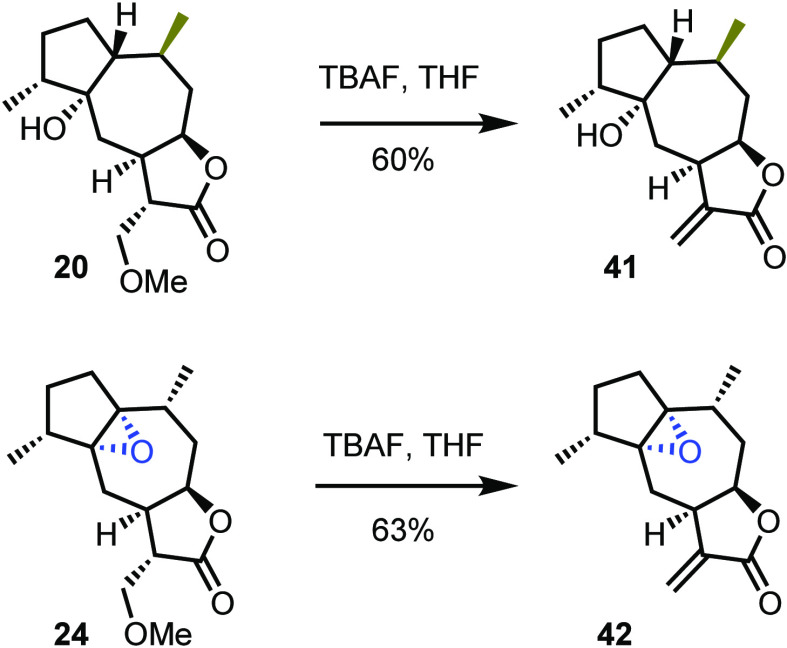
Indicative Deprotections to Reveal the α-Methylene-γ-butenolide Core

In conclusion, scalable access to a rich variety of biologically relevant, unprecedented *syn*-8,12-SQLs is demonstrated. The expansion of the chemical space of SQLs relies on the utilization of the rationally designed guaiane scaffold (**2**) that enables its selective diversification at positions C4, C5, and C10 of the carbocycle in terms of reduction, oxidation, and dehydration sequences. Key reactions of the divergent synthesis involve the development of acid-directed CH radical lactonization and the highly diastereoselective oxy-Cope/ene cascade to construct the guaiane carbocycle. Biological screening of these compounds regarding their anticancer activity is underway.

## Data Availability

The data underlying this study are available in the published article and its Supporting Information.

## Supporting Information Available

Synthetic protocols and spectroscopic characterization and ^1^H and ^13^C NMR spectra of new compounds (PDF)
