# Supplementary material for: Expanding Natural Diversity: Tailored Enrichment of the 8,12-Sesquiterpenoid Lactone Chemical Space through Divergent Synthesis
Source: Org Lett. 2024 May 23;26(22):4648–53. doi: 10.1021/acs.orglett.4c01374 (PMC11187629; doi:10.1021/acs.orglett.4c01374)
Supplement: Supplementary file 1 — ol4c01374_si_001.pdf [file ol4c01374_si_001.pdf]

Expanding Natural Diversity: Tailored enrichment of 8,12-Sesquiterpenoid Lactone Chemical Space through Divergent Synthesis

Vera P. Demertzidou,† Maria Kourgiantaki† and Alexandros L. Zografos*

Laboratory of Organic Chemistry, Department of Chemistry, Aristotle University of Thessaloniki, Main University Campus, 54124, Thessaloniki, Greece

Corresponding Authors:

E-mail: alzograf@chem.auth.gr

Supporting Information

Table of Contents

	Page
**1. Materials and Methods**	**SI-5**
**2. Biosynthetic considerations-How lactone moiety precludes differentiation in sesquiterpenoids**	**SI-6**
**3. Selected divergent syntheses of sesquiterpenoids**	**SI-7**
**4. Designing common synthetic scaffolds. The logic behind the selection**	**SI-10**
**5. Modelling lactonization reaction**	**S-10**
**5.1. Synthesis of substrates**	**SI-10**
**5.2 Attempts for 8,12-CH-lactonization**	**SI-11**
**6. Divergent synthesis of sesquiterpenoids**	**SI-13**
**6.1 Optimization for the multigram synthesis of (R)-carvonic acid**	**SI-14**
**6.1.1 Allylic chloride 9: Development of a new method for the allylic chlorination of (*R*)-carvone (step 1)**	**SI-14**
**6.1.2 Synthesis of carvonic acid 8 (step 2)**	**SI-16**
**6.2 Early-stage introduction of *α*-methylene-*γ*-butyrolactone core from carvonic acid 8-Optimization for CH-lactonization**	**SI-21**
**6.2.1 Attempting allylic halogenation**	**SI-21**
**6.2.2 Attempting allylic hydroxylation-alkoxylation**	**SI-23**
**6.2.3 Carboxylic acid as directing group for radical CH-lactonization-modified Suarez conditions-Postulated reaction mechanism**	**SI-24**
**6.3 Final alkylation for the synthesis of common scaffold 19**	**SI-32**
**6.4 Synthesis of sulfur-ylides for accessing trans-6-hydroxy derivatives**	**SI-37**
**6.5 Utilization of common scaffold for the synthesis of sesquiterpenoid lactones**	**SI-41**
**6.5.1 Synthesis of germacranolides**	**SI-41**

**6.5.2 Synthesis of guaianolide lactones**	**SI-41**
**6.5.3 Mechanistic considerations-conformational analysis**	**SI-41**
**6.6 Enrichment of the chemical space of guaianolide 27**	**SI-44**
**6.6.1 Reduction-Dehydration-Oxidation sequence**	**SI-44**
**6.6.2 Dehydration-Reduction or Oxidation sequence**	**SI-47**
**6.6.3 (formal)Oxidation-Dehydration sequence**	**SI-51**
**6.6.4 Deprotection of the lactone moiety**	**SI-54**
**7. Spectra of substrates and products**	**SI-56**
**8. References**	**SI-160**

Abbreviations

AIBN	Azobisisobutyronitrile
DCM	Dichloromethane
DIBAL	Diisobutylaluminium hydride
DMAP	4-Dimethylaminopyridine
DMF	Dimethylformamide
DMP	Dess-Martin periodinane
DMS	Dimethyl sulfide
DMSO	Dimethyl sulfoxide
HMPA	Hexamethylphosphoramide
IBX	2-Iodoxybenzoic acid
KHMDS	Potassium bis(trimethylsilyl)amide
L-Selectride	Lithium tri-sec-butylborohydride
LiHMDS	Lithium bis(trimethylsilyl)amide
MB	Methylene blue
*m*CPBA	Meta-Chloroperoxybenzoic acid
NBS	N-Bromosuccinimide
NCS	N-Chlorosuccinimide
NIS	N-Iodosuccinimide
NMO	N-Methylmorpholine N-oxide
o/n	Overnight
PCC	Pyridinium chlorochromate
PDC	Pyridinium dichromate
PIDA	(Diacetoxyiodo)benzene
*p*TSA	p-Toluenesulfonic acid
r.t.	room temperature
TEMPO	(2,2,6,6-Tetramethylpiperidin-1-yl)oxyl
TMAO	Trimethylamine N-oxide
TMSCl	Trimethylsilyl chloride

1. Materials and Methods

All reactions were carried out under an argon (Ar) atmosphere with dry solvents under anhydrous conditions. Anhydrous solvents were either obtained from commercial sources (dry DMF, dioxane, DMSO and MeOH) or dried accordingly. Dry diethyl ether (Et_2O), and tetrahydrofuran (THF), were obtained by refluxing the solvents with sodium metal as drying agent and benzophenone as indicator for several hours, dry acetonitrile was dried by distillation from P_2O_5, whereas methylene chloride (CH_2Cl_2) from CaH_2. The solvents were kept under Ar using molecular sieves 4Å in their bottles. Petroleum ether refers to the 40-60°C boiling fraction. Commercially available reagents were purchased at the highest commercial quality and used without further purification or where specified, purified by standard techniques.

Reactions were monitored by thin-layer chromatography (TLC) carried out on S-2 0.25 mm E. Merck silica gel plates (60F-254) using UV light as visualizing agent (λ_{max}= 254 nm or 360 nm) and ethanolic *p*-anisaldehyde as developing agent or by *Seebach* TLC stain solution, followed by heating. E. Merck silica gel (60, particle size 0.040–0.063 mm) was used for flash column chromatography. Preparative TLC plates (S-2 0.5mm E. Merck silica gel plates precoated with silica gel 60-F254) were used in cases where the separation with usual flash column chromatography were inadequate. NMR spectra were recorded at 298 K using an Agilent Technologies DD2 500 spectrometer and calibrated by residual solvent peaks. ^{1}H NMR spectra were recorded at 500 MHz and residual solvent peaks were used as an internal reference ($CDCl_3$ δ 7.26). Data are reported as follows: chemical shift in ppm, multiplicity (s = singlet, brs = broad singlet, d = doublet, brd = broad doublet, t = triplet, brt = broad triplet, q = quartet, m = multiplet or overlap of nonequivalent resonances, coupling constants are reported in Hz, integration is included. ^{13}C NMR spectra were recorded at 125 MHz and residual solvent peaks were used as an internal reference ($CDCl_3$ δ 77.00). Data are reported as follows: chemical shift in ppm, multiplicity deduced. The assignment of ^{1}H and ^{13}C signals was assisted by COSY, HSQC, HMBC and NOESY experiments where necessary.

High Pressure Liquid Chromatography (HPLC) was performed on an Agilent 1260 Infinity II spectrometer to monitor reaction kinetics. Optical rotations were recorded on a Krüss Optronic polarimeter at 589 nm and are reported in units of 10–1(deg cm2 g–1). High-resolution mass spectra (HRMS) were recorded on an Agilent ESI-TOF (time of light) mass spectrometer at a 4000V emitter voltage. Melting points were obtained by Stuart Melting Point Apparatus SMP3, Bibby Scientific. Microanalyses were performed on a Perkin-Elmer 2400-II element analyzer. The reactions carried under microwave conditions were performed on a Biotage Initiator EXP EU 355301, 112310-25W.

2. Biosynthetic considerations-How lactone moiety precludes differentiation in sesquiterpenoids

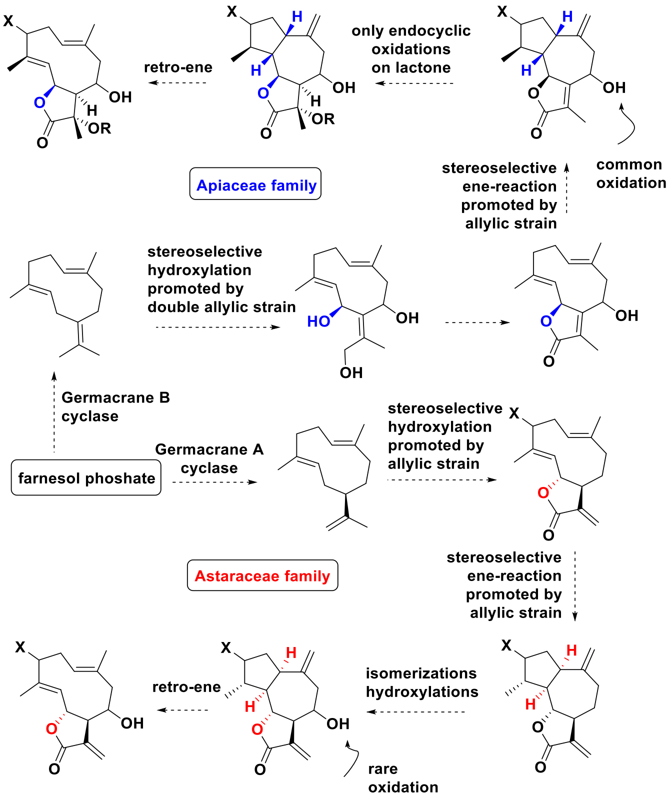

Figure 1. Biosynthetic considerations.

A close inspection of different classes of guainolide sesquiterpenoids reveals the distinct production of the *anti*-relationship between the bridgehead hydrogen atoms of the cyclopentane core and the pendant allyl chain that forms the lactone moiety, for *Asteraceae* derived sesquiterpenoids (Figure 1; red highlighted), while the *Apiaceae* family tends to produce the *syn*-relationship between the same centers (Figure 1; blue highlighted). Postulated biosynthesis for the observed diversity as translated for germacranolides and guaianolides is presented above. Based on that, allylic oxidations of germacrane A and B are delivered stereoselectively by allylic strain. Ene-reactions that follow for the formation of guaianolides are concerted. The locked conformations of germacrane A and B cores, as resulted from the mono and bis allylic strain respectively, drive the selective formation of guaianolides. The observed specificity for *Asteraceae* and Apiaceae natural products stereochemistry is attributed to selected formation of germacrane A and B respectively. Based on these observations, the early formation of α-methylene-γ-butyrolactone core is considered as a key point for advancing stereospecificity in current synthetic plan.

3. Selected divergent syntheses of sesquiterpenoids

A. Baran's synthetic plan to various sesquiterpenoids (Angew. Chem. Int. Ed., 2012, 51, 46, 11491)

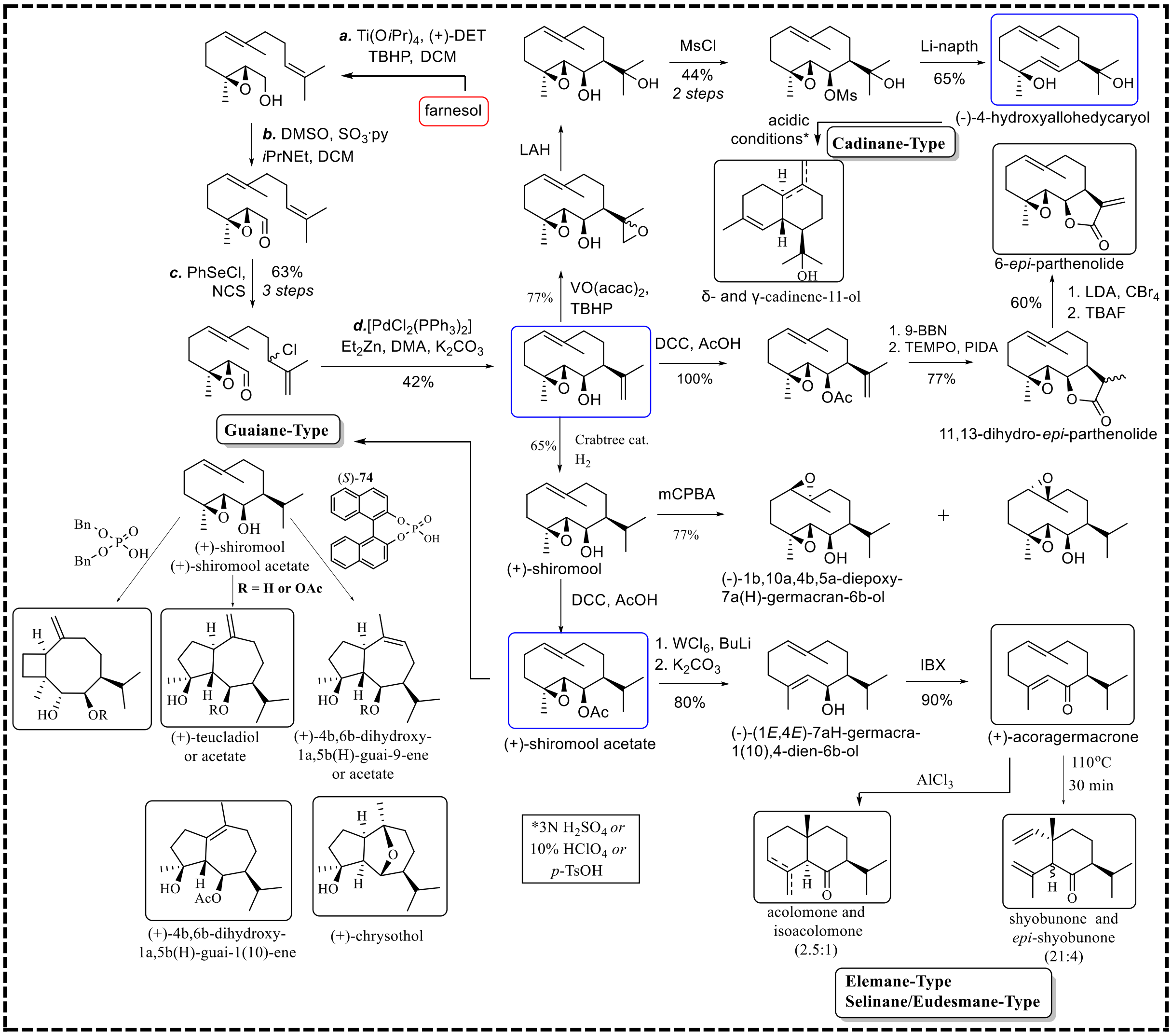

B. Winssinger's approach to guaianolide complexity (Angew. Chem. Int. Ed., 2012, 51, 5391)

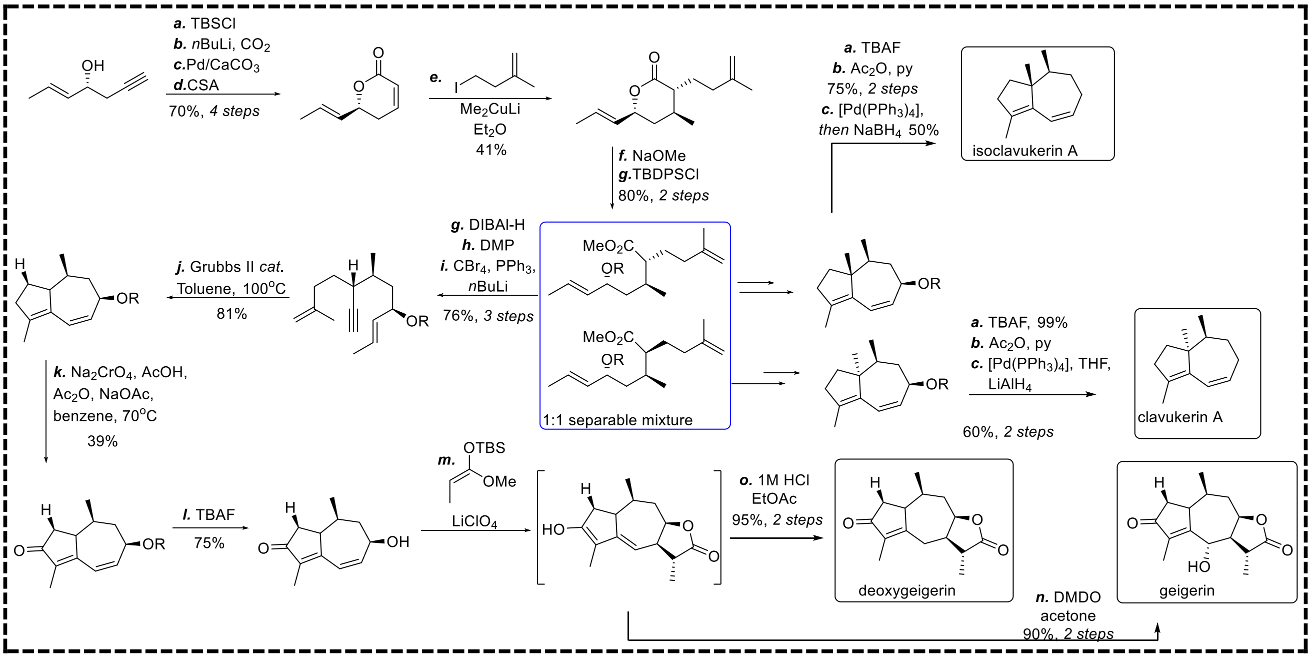

C. Maimone’s synthetic plan to diverse guaianolides (JACS, 2019, 141, 37, 14904)

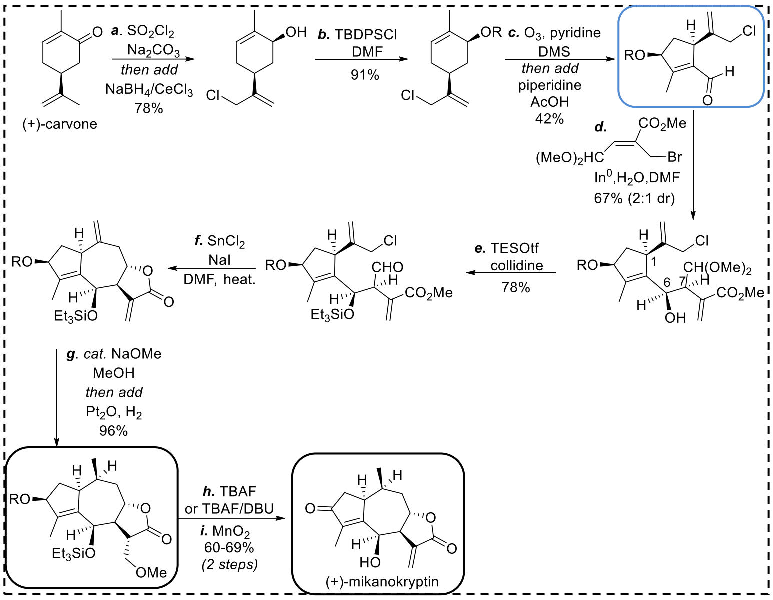

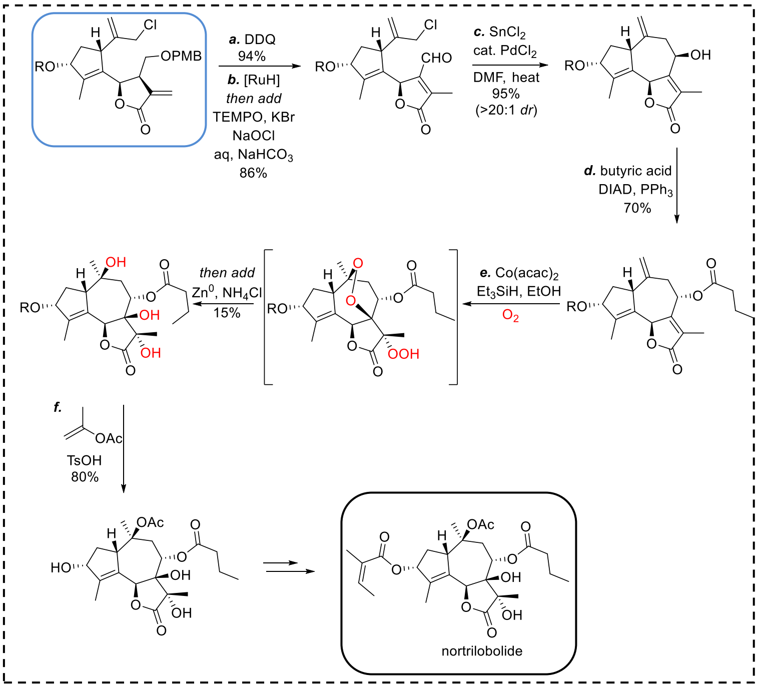

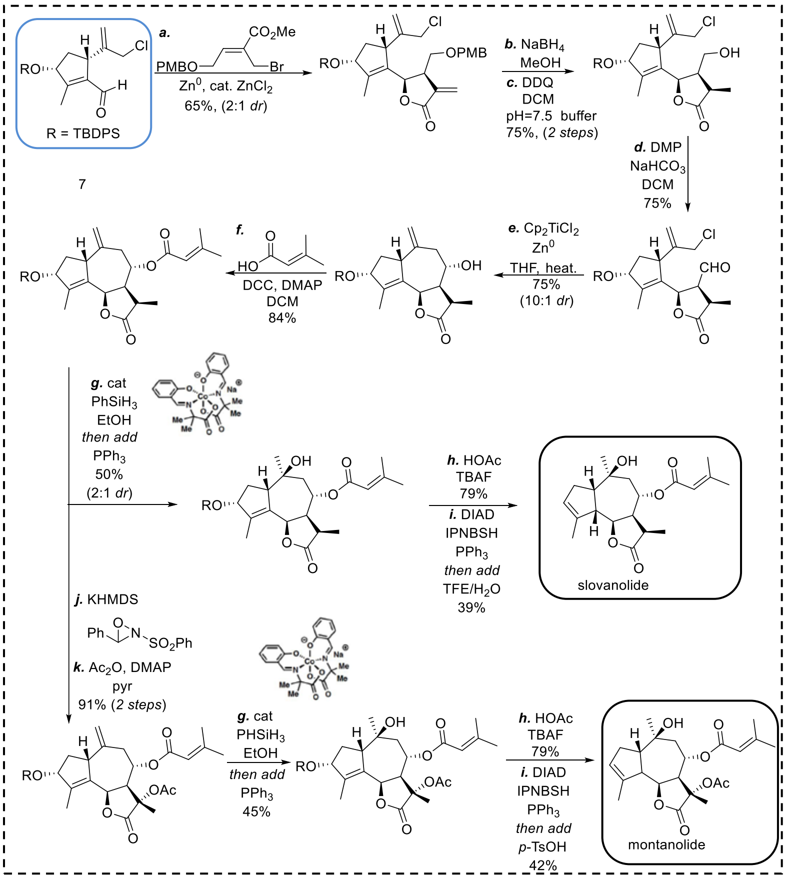

D. Zografos's divergent total synthesis of sesquiterpenoids (Org. Lett., 2013, 15, 152; Chem. Comm., 2015, 51, 2364; Org. Biomol. Chem., 2016, 14, 6942)

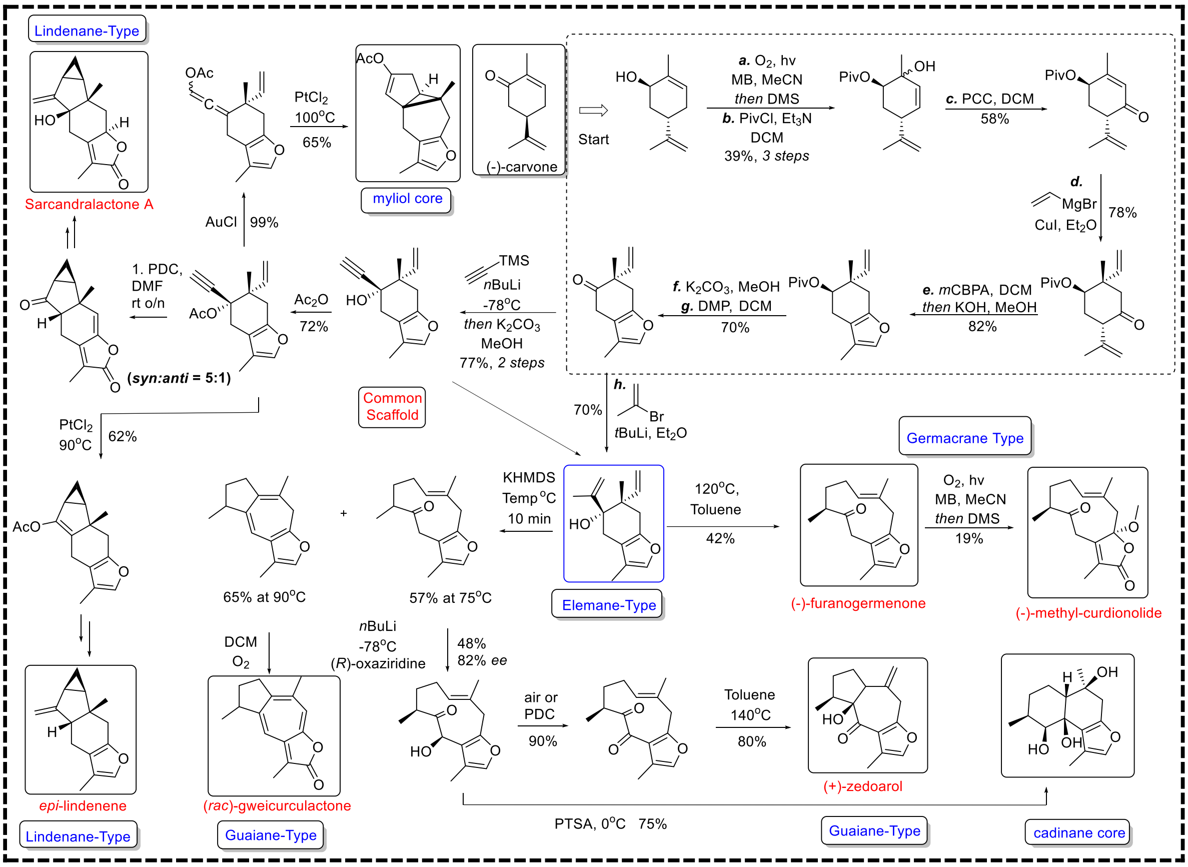

Comparison of characteristics for selected divergent strategies and current work.

Route	Multiple carbocycles	Ability to access both 6- and 8,12-lactones	Access to highly oxidized products	Steps to common scaffold	Average steps to natural products	Gram scale
A. Baran's	✓	✗	✓	4	4	✓
B. Winssinger's	✗	✓	✗	10	3	✗
C. Maimone's	✗	✓	✓	3	7	✓
D. Zografos's	✓	✗	✓	10	3	✗
Current work	✓	✓	✓	8	3	✓

Our previously published synthetic route utilizes furan-elemanes as common synthetic scaffolds to access various carbocyclic cores of sesquiterpeneoids. The selection of furan-elemane common scaffolds relied on the ability of *trans*-positioned unsaturations (diene and enyne) to succeed: 1. a non-reversible, non-biomimetic, enantioselective oxy-Cope reaction for the synthesis of a crucial biosynthetic germacrane intermediate and 2. highly selective cycloisomerizations to deliver cyclopropane sesquiterpenoids. Biomimetic utilization of the germacrane intermediate enriched the route with furoguaiane and furocadinane sesquiterpenoids. Further oxidation steps allowed the addition of further functionality following the biomimetic two-phase protocol (cyclase-oxidase phase).

Major drawback of the described plan is the inaccessibility to α-methylene-γ-butyrolactone sesquiterpenoids. Direct oxidation of furan functionality resulted in only poor yields of α,β-unsaturated-γ-butenolides, which we were unable

to transform into the desired exocyclic methylene lactone. Also, despite the undeniable success of this plan to provide diversity, it lacks scalability majorly due to the incorporation of a photochemical oxidation and the instability of furo-ketone *en-route* to the common synthetic scaffold.

Considering these facts, we turned our attention to alternative synthetic routes that can still provide the described diversity, enriching it with the ability to introduce the biologically important α-methylene-γ-butyrolactone cores.

4. Designing common synthetic scaffolds: The logic behind the selection.

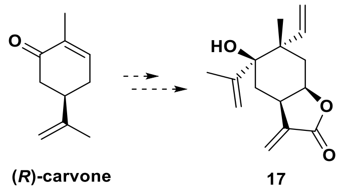

Scheme S1. Common synthetic scaffolds

Wishing to overpass the drawbacks of our previous plan, we considered the synthesis of common scaffold **17**, bearing the syn-α-methylene-γ-butyrolactone functionality. The *syn*-conformation of the lactone moiety was selected based on the lower rigidity of the structure that favours better biological profiles.[1] *Syn*-relation between methyl group and lactone moiety is crucial to retain the access to lindenane and myliol sesquiterpenoids (see plan above). Finally, ideally α-side is preferred for 2-propenyl-chain to gain access to elemane natural products. Its construction was envisioned directly from the acrylic acid functionality, through a CH-lactonization protocol.

5. Modelling lactonization reaction

5.1. Synthesis of substrates

Considering that no precedents for direct lactonization of functionalized acrylic acids are reported, we modelled the sequence by utilizing the appropriate functionalization. Direct evolution of (*R*)-carvone to functionalized intermediate **SI-1** is known[2] to produce the *anti*-relation between the methyl- and the 2-propenyl group, which is epimeric to the desired **17**. Although this intermediate will eventually hamper our access to lindenane and myliol sesquiterpenoids, we utilized (*R*)-carvone as a cheap starting material to test the feasibility of a direct CH-lactonization.

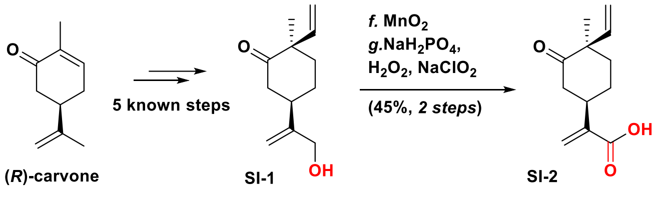

Scheme S2. Synthesis of model compound **SI-2** for the CH-lactonization[2].

2-((1*R*,4*R*)-4-methyl-3-oxo-4-vinylcyclohexyl)acrylic acid (SI-2)

Compound SI-1 (109 mg, 0.56 mmol, 1.0 eq) was dissolved in CH_2Cl_2 (6 mL) and MnO_2 (488 mg, 5.61 mmol, 10 eq) was added in one portion and the reaction was stirred at room temperature for 12 h. The resulting black suspension was then poured over a pad of Celite® and eluted with Et_2O. The filtrate was concentrated under reduced pressure to

provide the corresponding aldehyde which was used without further purification in the next step. For characterization purposes the resulting crude mixture was chromatographed (silica gel) with gradient from 5:1 hexane: EtOAc to 1:1 hexane: EtOAc to afford the corresponding pure aldehyde **SI-1a** the spectroscopic data of which are: ^{1}H NMR (500 MHz, $CDCl_3$): δ = 9.52 (s, 1H), 6.25 (s, 1H), 6.04 (s, 1H), 5.93 (dd, *J* = 17.6, 10.7 Hz, 1H), 5.18 (d, *J* = 10.6 Hz, 1H), 5.01 (d, *J* = 17.6 Hz, 1H), 2.92 (d, *J* = 12.2 Hz, 1H), 2.61 (t, *J* =13.4 Hz, 1H), 2.42-2.28 (m, 1H), 2.06-2.00 (m, 1H), 1.90-1.63 (m, 3H), 1.16 (s, 3H). ^{13}C NMR (125 MHz, CDCl3): δ = 211.3, 193.8, 152.4, 142.3, 133.6, 115.8, 51.8, 43.3, 38.7, 37.7, 27.3, 24.1. The resulting crude mixture was dissolved in CH_3CN (5 mL) and the mixture was cooled to 0 °C. Then, $Na_2H_2PO_4$ (41 mg, 0.35 mmol, 0.6 eq) was dissolved in water (0.7 mL) and was added to the above solution, followed by the addition of a solution of H_2O_2 (30% wt, 0.62 mL, 9.6 eq) and the resulting mixture was stirred at 0 °C for 20 minutes. $NaClO_2$ (59 mg, 0.648 mmol, 4 eq) was then dissolved in water (1.4 mL) and was added dropwise to the reaction mixture, which was allowed to warm to room temperature and stir for 8 h. The solvent was removed *in vacuo* and the aqueous residue was brought to pH 3.0 with HCl (6N, 2 mL). The aqueous layer was extracted three times with EtOAc (3x5 mL) and the combined organic extracts were dried over $NaSO_4$, filtered and concentrated under reduced pressure. The residue was chromatographed (silica gel) with gradient from 2:1 hexane: EtOAc to 1:2 hexane: EtOAc [R_f = 0.5 (hexane:EtOAc 1:3, UV active on TLC, stains brown upon *p*-anisaldehyde staining)] to afford pure **Compound SI-2** as a colorless oil (53 mg, 45%, two steps). $[\alpha]^{20}_{D}$ = +76.8 (*c* 1.3, $CHCl_3$). HRMS (ESI, m/z): calcd. for $C_{12}H_{16}O_3Na^+$ ($[M+Na]^+$): 231.0997, found: 231.0993. ^{1}H NMR (500 MHz, $CDCl_3$): δ = 6.38 (s, 1H), 5.92 (dd, *J* =17.6, 10.8 Hz, 1H), 5.68 (s, 1H), 5.18 (d, *J* =10.6 Hz, 1H), 5.02 (d, *J* =17.7 Hz, 1H), 2.97-2.81 (m, 1H), 2.60 (t, *J* = 13.4, 1H), 2.43 (ddd, *J* = 13.6, 3.9, 2.2 Hz 1H), 2.07 (dt, *J* =13.7, 3.3 Hz, 1H), 1.93-1.87 (m, 1H), 1.87-1.76 (m, 1H), 1.75-1.58 (m, 1H), 1.17 (s, 3H). ^{13}C NMR (125 MHz, $CDCl_3$): δ = 211.9, 171.8, 142.6, 142.2, 126.4, 115.9, 51.7, 44.1, 40.4, 38.7, 27.8, 24.1.

5.2 Attempts for 8,12-CH-lactonization.

Compound **SI-2** was tested for CH-lactonization, to produce lactones **SI-3** and **SI-4**. Several protocols were applied utilizing carboxylic acid as directing group, or as radical mediator. In all cases, the desired compound was not delivered (Table S1). In some cases, the synthesis of **SI-3** was observed. Differentiation between radical and ionic conditions leads to the observation that radical conditions tend to produce more selective reactions, at least for the formation of compound **SI-3**. Optimization of reaction conditions allowed the selective production of *syn*-6,12-*α*-methylene-*γ*-butyrolactone **SI-3** in 58% yield (Table S1). The failure to synthesize even traces of compound **SI-4** indicates the great challenge to achieve the CH-lactonization.

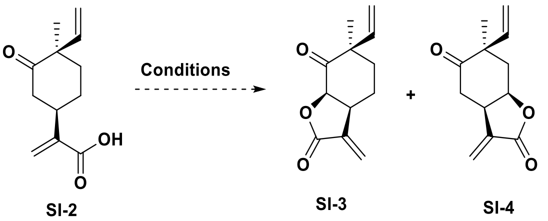

Table S1. Selected attempts to induce 8,12-CH-lactonization to compound **SI-2**.

Entry	Radical vs ionic	Conditions	Products	Yields
1	Radical conditions	**SI-2**, PIDA (4 equiv), I_2 (1 equiv), rt 2h, UV-VIS irradiation benzene	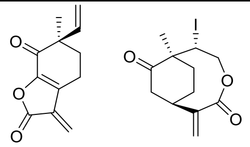 SI-5 SI-6 decomposition products	**SI-5** (5%); **SI-6** (29%)
2		**SI-2**, PIDA (4 equiv), I_2 (1 equiv), H_2O (1equiv), rt 2 hr, UV-VIS irradiation benzene	**SI-5, SI-6**, polyiodinations	**SI-5** (7%); **SI-6** (8%)
3		**SI-2**, NBS (1 equiv), AIBN (0.1 equiv), 80 °C, benzene, 2.5 hr	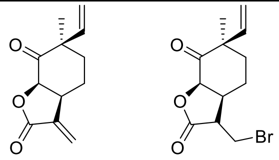 SI-3 SI-7 polybrominated products	**SI-3** (22%), **SI-7** (5%)
4		**SI-2**, NBS (1 equiv), AIBN (0.1 equiv), 80 °C, CCl_4, 2.5 hr	**SI-3**, **SI-7,** polybrominated products	**SI-3** (10%), **SI-7** (10%),
5		**SI-2**, NBS (1 equiv), AIBN (0.1 equiv), 80 °C, Cs_2CO_3 (1equiv), Benzene, 2.5h	**SI-2**	Unreacted starting material
6		**SI-2**, NBS (1 equiv) slow addition, UV irradiation, 3 hr	**SI-3, SI-2**	**SI-3** (58%), **SI-2** (30%)
7	Ionic conditions	**SI-2**, LiHMDS (2 equiv), $CuCl_2$, THF, -78 °C to rt, 4 hr	**SI-3, SI-2**	**SI-3** (14%), **SI-2** (80%)
8		**SI-2**, n-BuLi (2 equiv), $FeCl_3$, THF, -78 °C to rt, 4 hr	Complex mixture of products	Complex mixture
9		**SI-2**, Br_2 (1 equiv), Et_2O, -78 °C to rt, 1.5 hr	polybrominated products	Complex mixture
10		**SI-2**, PIDA (1 equiv), benzene, reflux, 12h	**SI-3, SI-2**	**SI-3** (28%), **SI-2** (63%)

Despite the failure of delivering *syn*-8,12-*α*-methylene-*γ*-butyrolactones, the model plan served as platform to develop crucial reactions, as the introduction of tertiary vinyl group and the sequence to obtain acrylic acid functionality. Also, the survey resulted a selected method to synthesize *syn*-6,12-*α*-methylene-*γ*-butyrolactone **SI-3** in moderate yield (58%, 83% brsm) (entry 6, Table S1).

(3a*S*,6*R*,7a*R*)-6-methyl-3-methylene-6-vinyltetrahydrobenzofuran-2,7(3H,4H)-dione (SI-3).

Compound SI-2 (400 mg, 1.92 mmol, 1.0 eq) was dissolved in Et_2O (48 mL) in a large test tube under Ar atmosphere before it was exposed to UV irradiation with simultaneous dropwise addition of a solution of NBS (342 mg, 1.92 mmol, 1.0 eq) in 12 mL THF with the aid of syringe pump (addition rate: 0.2 mL/min). After the addition was over, the reaction mixture was left under visible light irradiation for additional 2.5 h before it was quenched with saturated aqueous

solution of $NaHCO_3$ (20 mL). The aqueous layer was then separated and extracted three times with EtOAc (4x10 mL). The combined organic layers were washed twice with brine (2x30 mL), dried over $NaSO_4$, filtered, and concentrated under reduced pressure. The residue was chromatographed (silica gel) with gradient from 20:1 hexane: EtOAc to 12:1 hexane: EtOAc[R_f = 0.73 (hexane:EtOAc = 1:3, UV active on TLC, stains purple upon Seebach staining)] to afford pure **Compound SI-3** as a white amorphous solid (230 mg, 58%). $[\alpha]^{20}_{D}$ = -58.3 (*c* 0.38, $CHCl_3$). HRMS (ESI, m/z): calcd. for $C_{12}H_{15}O_3^+$ ($[M+H]^+$): 207.1021, found: 207.1018.^{1}H NMR (500 MHz, $CDCl_3$) δ = 6.30 (d, *J* = 3.2 Hz, 1H), 6.02 (dd, *J* = 17.6, 10.8 Hz, 1H), 5.60 (d, *J* = 2.8 Hz, 1H), 5.16 (d, *J* = 10.9 Hz, 1H), 5.08 (s, 1H), 5.05 (d, *J* = 7.1 Hz, 1H), 3.73 – 3.64 (m, 1H), 2.31 – 2.18 (m, 1H), 2.04 – 1.94 (m, 1H), 1.85 – 1.73 (m, 2H), 1.33 (s, 3H).^{13}C NMR (125 MHz, $CDCl_3$) δ = 207.1, 169.2, 140.1, 136.2, 122.5, 114.3, 78.1, 50.5, 42.6, 33.2, 23.2, 22.7.

(1*R*,6*S*,7*S*)-6-iodo-7-methyl-2-methylene-4-oxabicyclo[5.2.2]undecane-3,8-dione (SI-6)

Compound SI-2 (20 mg, 0.1 mmol, 1.0 eq) was dissolved in benzene (2 mL) in a light permeable glass tube and PIDA (134 mg, 0.4 mmol, 4.0 eq) was added to the above solution. The resulting mixture was left stirring at room temperature under Ar for 10 min before it was exposed to visible light with simultaneous dropwise addition of a solution of iodine (25 mg, 0.1 mmol, 1eq) in 20 mL benzene with the aid of syringe pump (addition rate: 0.1 mL/min). After the addition was over, the reaction mixture was left under visible light irradiation for additional 12 h before it was quenched with saturated aqueous solution of $Na_2S_2O_3$ (20 mL), whereupon it was left stirring for 2h. The aqueous layer was then separated and extracted three times with EtOAc (3x10 mL). The combined organic layers were washed twice with saturated aqueous $NaHCO_3$ (2x30 mL), dried over $NaSO_4$, filtered, and concentrated under reduced pressure. The residue wa chromatographed (silica gel) with gradient from 20:1 hexane: EtOAc to 12:1 hexane: EtOAc [R_f = 0.32 (hexane:EtOAc = 2:1, UV active on TLC, stains purple upon *p*-anisaldehyde staining)] to afford pure **Compound SI-6** as a yellowish amorphous solid (9 mg, 29%). $[\alpha]^{20}_{D}$ = -38.5 (*c* 0.51, $CHCl_3$). HRMS (ESI, m/z): calcd. for $C_{12}H_{16}IO_3^+$ ($[M+H]^+$): 335.0144, found: 335.0140. ^{1}H NMR (500 MHz, $CDCl_3$) δ = 6.36 (s, 1H), 5.57 (s, 1H), 4.56 – 4.46 (m, 2H), 4.16 – 4.07 (m, 1H), 3.06 (s, 1H), 2.31 (dd, *J* = 13.6, 3.9 Hz, 1H), 2.13 – 2.07 (m, 1H), 1.63 (dd, *J* = 13.6, 4.2 Hz, 3H), 1.46 – 1.37 (m, 1H), 1.20 (s, 3H). ^{13}C NMR (125 MHz, $CDCl_3$) δ = 207.4, 164.4, 138.5, 126.7, 74.7, 48.1, 35.6, 30.5, 28.6, 28.4, 27.5, 22.3.

6. Divergent synthesis of sesquiterpenoids

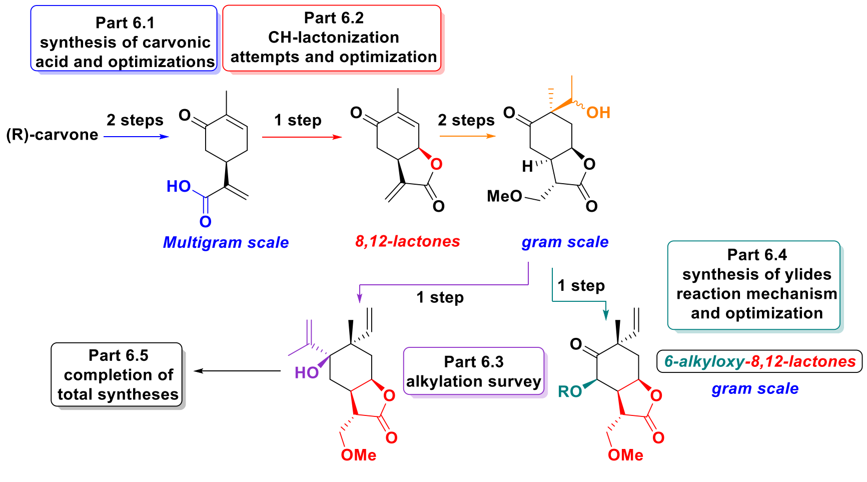

Scheme S3. Index to guide through the synthetic route.

6.1 Optimization for the multigram synthesis of (*R*)-carvonic acid 4

Followed our design above, we scanned previously reported but also newly developed methods to efficiently access (*R*)-carvonic acid **4**. Our plans involved the synthetic sequence shown below.

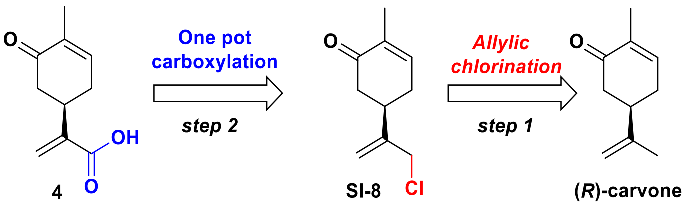

Scheme S4. Retrosynthesis of carvonic acid **4**.

6.1.1 Allylic chloride SI-8: Development of a new method for the allylic chlorination of (*R*)-carvone (step 1)

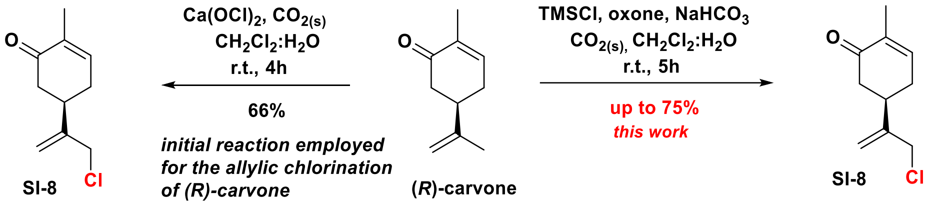

Scheme S5. Developing a novel allylic chlorination of carvone

Allylic chlorination of terminal alkenes is a useful chemical process that usually requires the utilization of hypochlorite salts or chlorine gas for its accomplishment. Small scale reactions are avoiding chlorine gas, due to safety issues and rely on hypochlorite salts as a safe alternative. Allylic chlorination of carvone raises an additional regioselectivity issue, due to the existence of a second, though non-activated endocyclic double bond. Based on that, the only reliable method for chlorination of carvone that utilizes hypochlorite salts (calcium, barium, sodium) provided in our hands, yields between 55-66%.[3] Use of hypochloride solution usually results in non-reproducible yields in large scale reactions. On the other hand, the use of calcium and barium salts, results copious filtrations and persistent wash ups to retrieve the product, especially during scale up. Development of a new protocol, based on oxone and TMSCl was developed for this work, to allow us to easy scale-up up to 100 g (see below). Our method provided easier isolation on large scale and on average better yields of the desired carvone allylic chloride **SI-8** (75% compared to 66%). Please see Table S2 below for comparison of methods and optimization attempts.

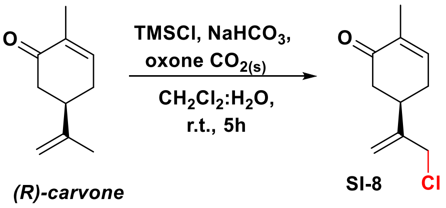

Table S2. Optimization conditions for preparation of **SI-8**.

Entry	Conditions[a]	Solvent (ratio)	Time	Conversion	Yield
**1**	$NaOCl_{aq}$ (1M) (2.5 equiv), dry ice	CH_2Cl_2: H_2O (1:1)	3h	100%	68%
**2**	CaOCl (1.5 equiv), dry ice	CH_2Cl_2: H_2O (1:1)	4h	100%	66%
**3**	TMSCl (4 equiv), oxone (4 equiv)	CH_3CN: H_2O (1:1)	1h	60%	25%
**4**	TMSCl (2 equiv), oxone (4 equiv), $NaHCO_3$ (4 equiv)	CH_3CN: H_2O (1:1)	12h	100%	67%

**5**	TMSCl (2 equiv), oxone (4 equiv), $NaHCO_3$ (4 equiv)	1,4-Dioxane:H_2O (1:1)	3h	100%	17%
**6**	TMSCl (2 equiv), oxone (4 equiv), $NaHCO_3$ (4 equiv)	Cyclohexane:H_2O (1:2)	4h	59%	23%
**7**	TMSCl (2 equiv), oxone (4 equiv), $NaHCO_3$ (4 equiv)	CH_2Cl_2: H_2O (1:1)	1h	50%	30%
**8**	TMSCl (2 equiv), oxone (4 equiv), $NaHCO_3$ (4 equiv)	CH_2Cl_2: H_2O (1:2)	6h	100%	40%
**9**	TMSCl (2 equiv), oxone (4 equiv), $NaHCO_3$ (4 equiv), dry ice	CH_2Cl_2: H_2O (3:2)	5h	100%	56%
**10**	TMSCl (3 equiv), oxone (4 equiv), $NaHCO_3$ (4 equiv)	CH_2Cl_2: H_2O (3:2)	3h	100%	54%
**11**	TMSCl (3 equiv), oxone (4 equiv), $NaHCO_3$ (4 equiv), dry ice	CH_2Cl_2: H_2O (3:2)	3h	100%	65%
**12**[b]	TMSCl (3 equiv), oxone (3 equiv), $NaHCO_3$ (4 equiv), dry ice	CH_2Cl_2: H_2O (3:2)	5h	100%	75%
**13**[b]	$NaOCl_{aq}$ (1M) (2.5 equiv), dry ice	CH_2Cl_2: H_2O (1:1)	5h	100%	54%
**14**[b]	CaOCl 1.5eq, dry ice	CH_2Cl_2: H_2O (1:1)	5h	100%	62%
**15**[c]	TMSCl (3 equiv), oxone (3 equiv), $NaHCO_3$ (4 equiv), dry ice	CH_2Cl_2: H_2O (3:2)	5h	100%	76%
**16**[c]	CaOCl 1.5eq, dry ice	CH_2Cl_2 :H_2O (1:1)	5h	100%	55%

[a] All reactions were ran to 0.33 mmol scale of (R)-carvone, unless otherwise noted. Carvone is dissolved followed by chlorinating reagents or TMSCl and oxone. Addition of small pieces of dry ice throughout the 3 following hours is advantageous; [b] Reaction scale at 0.13 mol; [c] Reaction scale at 0.32 mol.

(*R*)-5-(3-chloroprop-1-en-2-yl)-2-methylcyclohex-2-en-1-one (SI-8)

Hypochlorite Method:[3] In a 1000 mL round-bottom-flask, $Ca(OCl)_2$ (45.6 g, 0.32 mol, 1.0 eq) was dissolved in H_2O (240 mL) and a solution of (*R*)-carvone (48 g, 0.32 mol) in CH_2Cl_2 (340 mL) was added to the above suspension. Upon that, small pieces of $CO_{2(s)}$ were added regularly in the reaction mixture until complete consumption of the starting material, which was monitored by TLC (approx. 3h). Then, the reaction mixture was filtered under vacuum to remove the insoluble inorganic components and the resulting filtrate was transferred to a separation funnel. The aqueous phase was separated, extracted three times with CH_2Cl_2 (3X100 mL) and the combined organic layers were dried over Na_2SO_4 and concentrated under reduce pressure. The resulting chloride **SI-8** (66% yield) was used without purification in the next step.

Oxone Method: (*R*)-carvone (48 g, 0.32 mol, 1.0 eq) was dissolved in the mixture of CH_2Cl_2:H_2O (3:2) (340:240 mL) in a 500 mL round-bottom flask and the solid $NaHCO_3$ (44.8 g, 0.52 mol, 4.0 eq) was then added in one portion. Next followed the slow addition of TMSCl (125 mL, 0.96 mol, 3.0 eq) and upon that, oxone (148 g, 0.96 mol, 3.0 eq) was added portionwise (every 10-15 min). Each portion addition was accompanied by the addition of pieces of $CO_{2(s)}$, which were also added to the reaction mixture during the time between the additions. Upon the consumption of the starting material, which was monitored through TLC, the reaction mixture was diluted with ca. 150 mL water and was transferred to a separation funnel. The aqueous phase was extracted three times with CH_2Cl_2 (3X250 mL) and the combined organic layers were dried over Na_2SO_4 and concentrated under reduce pressure. The resulting chloride **SI-8** was used in the next step without further purification. For characterization purposes the residue was chromatographed (silica gel) with gradient from 50:1 hexane: EtOAc to 25:1 hexane: EtOAc [R_f = 0.5 (hexane:EtOAc = 5:1, UV active on TLC, stains purple upon *p*-anisaldehyde staining)] to afford pure **Compound SI-8** as a yellow oil (44.3 g, 75%). $[\alpha]^{20}_D$ = -35.2 (*c* 0.4, $CHCl_3$). HRMS (ESI, m/z): calcd. for $C_{10}H_{13}ClONa^+$ ($[M+Na]^+$): 207.0553, found: 207.0551.

^{1}H NMR (500 MHz, $CDCl_3$) δ = 6.74 (ddd, J = 5.7, 2.6, 1.3 Hz, 1H), 5.24 (s, 1H), 5.04 (d, J = 1.2 Hz, 1H), 4.08 (d, J = 2.2 Hz, 2H), 2.95 (ddd, J = 14.1, 9.3, 4.7 Hz, 1H), 2.64 (ddd, J = 16.1, 3.8, 1.6 Hz, 1H), 2.60 – 2.49 (m, 1H), 2.41 – 2.26 (m, 2H), 1.78 (d, J = 1.2 Hz, 3H). ^{13}C NMR (125 MHz, $CDCl_3$) δ = 199.0, 146.6, 144.1, 135.6, 115.1, 47.0, 43.0, 37.9, 31.4, 15.7.

6.1.2 Synthesis of carvonic acid 8 (step 2)

6.1.2.1 Through allylic alcohol (**5**): Sequential oxidations to carvonic acid **4**

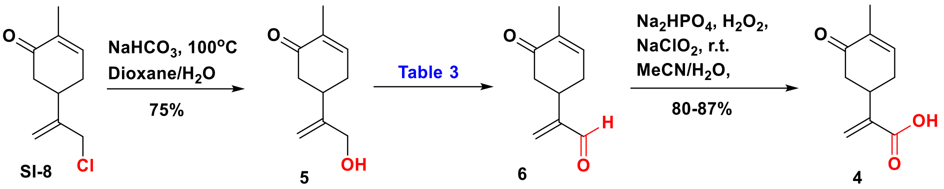

Scheme S6. Synthesis of carvonic acid **4**.

In our first approaches towards carvonic acid (**4**), allylic alcohol (**5**) was attempted to be utilized as its direct precursor. Allylic alcohol was prepared directly from crude allylic chloride following a known protocol[4] which provides 75% yield of **5** (see below). Several routes towards carboxylic acid **4** have been attempted by the sequential oxidation of allylic alcohol **5** to aldehyde **6** and then to carvonic acid **4**. The first oxidation step results in irreproducible results and average yields (35-60%) independent of the oxidant used for this process (see Table S3 for more details). Typical oxidations such as DMP and MnO_2 are not scalable providing 20-35% yields at 7 mmol scale. Likely, oxidation with PCC-silica was able to provide the aldehyde in 57% yield for a gram scale oxidation (entry 2) and also a satisfying result at a multigram scale (54 mmol), affording aldehyde in 48% yield (entry 3). The drop in yield is attributed to the difficulty of washing off the product from the sticky PCC residue. Aldehyde (**6**) to carvonic acid (**4**) transformation using the Lindgren oxidation protocol resulted good yields in general even at scale-up (80-87%).

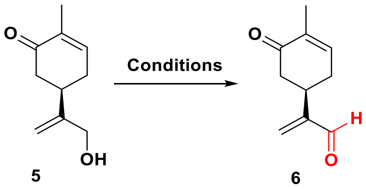

Table S3. Optimization conditions for the synthesis of **6**.

Entry[a]	Oxidant	Solvent	Temperature	Time	Yield
**1**	PDC (2eq)	dry CH_2Cl_2	rt	6h	45%
**2**	PCC (1.5eq)	dry CH_2Cl_2	rt	1h	57%
**3**[b]	PCC (1.5eq)	dry CH_2Cl_2	rt	1h	48%
**4**	MnO_2 (6 eq)	dry CH_2Cl_2	rt	12h	53%
**5**[c]	MnO_2 (6 eq)	dry CH_2Cl_2	rt	12h	35%
**6**	DMP (1.5 eq)	dry CH_2Cl_2	rt	1h	60%
**7**	$FeNO_3$ 10%, KCl 10%, TEMPO 10%, O_2,	dry CH_2Cl_2 or dry CH_3CN,	rt	12h	40%
**8**[c]	$FeNO_3$ 10%, KCl 10%, TEMPO 10%, O_2,	dry CH_3CN	rt	12h	20%
**9**[c]	CuBr 0.75%, Phenanthroline 0.75%, TEMPO 0.75%	dry CH_3CN	rt	12h	45%

	DMAP 0.75%, O_2

[a] All oxidations were conducted at 0.7 mmol scale unless otherwise noted; [b] oxidation ran at 54 mmol scale; [c] oxidations ran at 7 mmol scale.

(*R*)-5-(3-hydroxyprop-1-en-2-yl)-2-methylcyclohex-2-en-1-one (5)[4]

Compound SI-8 (4 g, 21.4 mmol, 1.0 eq) was dissolved in a mixture of 1,4-dioxane:H_2O (1:3) (60 mL) in a 250 mL round-bottom flask, and then $NaHCO_3$ (4.5 g, 53.4 mmol, 2.5 eq) was added in one portion. The reaction mixture was stirred at reflux for 7 h before being cooled to room temperature and quenched with ca. 40 mL of saturated aqueous NH_4Cl. The aqueous phase was separated and extracted three times with CH_2Cl_2 (3x40 mL) and the combined organic layers were dried over Na_2SO_4 and concentrated under reduce pressure to provide 3 g of **5** (86% yield) that was used without purification in the next step. For characterization purposes the residue was chromatographed (silica gel) with gradient from 40:1 hexane: EtOAc to 2:1 hexane: EtOAc to afford pure **Compound 5** as a yellow oil (2.7 g, 75%). The characterization data of this compound matches those reported in the literature: $[\alpha]^{20}_{D}$ = -71.5 (*c* 1.19, $CHCl_3$). HRMS (ESI, m/z): calcd. for $C_{10}H_{14}O_2Na^+$ ($[M+Na]^+$): 189.0891, found: 189.0888. ^1H NMR (500 MHz, $CDCl_3$) δ = 6.75 – 6.69 (m, 1H), 5.10 (s, 1H), 4.89 (s, 1H), 4.09 (s, 2H), 2.77 (m, 1H), 2.60 – 2.39 (m, 3H), 2.43 – 2.23 (m, 2H), 1.73 (d, *J* = 2.1, 3H). ^{13}C NMR (125 MHz, $CDCl_3$) δ = 199.9, 150.2, 144.9, 135.4, 110.2, 64.5, 43.2, 38.1, 31.6, 15.6.

(*R*)-2-(4-methyl-5-oxocyclohex-3-en-1-yl)acrylaldehyde (6)

To a stirring solution of **Compound 5** (1 g, 6 mmol, 1.0 eq) in CH_2Cl_2 (170 mL), was added a 1:1 mixture of silica-PCC (1.95 g, 9 mmol, 1.5 eq) and the reaction mixture was left stirring at room temperature for 30 min. The resulting brown suspension was then poured over a pad of Celite® and eluted with Et_2O. The filtrate was concentrated under reduced pressure and the residue was chromatographed (silica gel) with gradient from 15:1 hexane: EtOAc to 8:1 hexane: EtOAc [R_f = 0.46 (hexane:EtOAc = 1:1, UV active on TLC, stains greenish upon *p*-anisaldehyde staining)] to afford pure Compound **6** as a pink oil (562 mg, 57%). $[\alpha]^{20}_{D}$ = -66.8 (*c* 1.2, $CHCl_3$). HRMS (ESI, m/z): calcd. for $C_{10}H_{13}O_2^+$ ($[M+H]^+$): 165.0916, found: 165.0918. ^1H NMR (500 MHz, $CDCl_3$) δ = 9.51 (s, 1H), 6.74 – 6.65 (m, 1H), 6.26 (d, *J* = 1.1 Hz, 1H), 6.07 (s, 1H), 3.32 – 3.13 (m, 1H), 2.61 – 2.50 (m, 2H), 2.43 (dd, *J* = 16.1, 12.4 Hz, 1H), 2.35 – 2.14 (m, 1H), 1.75 (s, 3H). ^{13}C NMR (125 MHz, $CDCl_3$) δ = 198.7, 193.9, 151.4, 144.1, 135.6, 134.3, 42.1, 33.9, 31.0, 15.8.

(*R*)-2-(4-methyl-5-oxocyclohex-3-en-1-yl)acrylic acid (4)

To a stirring solution of **Compound 6** (562 mg, 3.42 mmol, 1.0 eq) in 6 ml CH_3CN at 0 °C, a solution of NaH_2PO_4 (360 mg, 2.31 mmol, 0.675 eq) in 0.4 mL H_2O and H_2O_2 (0.76 mL, 6.67 mmol, 30% wt, 1.95 eq) were added successively and the resulting mixture was left stirring at 0 °C for 10 min. Then, a solution of $NaClO_2$ (928 mg, 10.26 mmol, 3.0 eq) in 1 mL H_2O was added dropwise to the reaction mixture at 0 °C, which was left stirring at room temperature 1h. The solvent was removed *in vacuo* and the aqueous residue was brought to pH 8.0 with solid and/or saturated aqueous solution of $NaHCO_3$ before it was extracted three times with CH_2Cl_2 (3x5 mL) to remove non-acidic byproducts. The aqueous layer was then brought to pH 3.0 with HCl 6N at 0 °C before it was extracted three times with Et_2O (3x10 mL). The combined organic extracts were dried over $NaSO_4$, filtered and concentrated under reduced pressure. The residue

was chromatographed (silica gel) with gradient from 4:1 hexane: EtOAc to 1:2 hexane: EtOAc [R_f = 0.46 (hexane:EtOAc = 1:3, UV active on TLC, stains brown upon *p*-anisaldehyde staining)] to afford pure **Compound 4** as a colorless oil (511 mg, 83%). $[\alpha]^{20}_{D}$ = -64.8 (*c* 1.67, $CHCl_3$). HRMS (ESI, m/z): calcd. for $C_{10}H_{12}O_3Na^+$ ($[M+Na]^+$): 203.0684, found: 203.0680. ^{1}H NMR (500 MHz, $CDCl_3$) δ = 10.37 (brs, 1H), 6.75 (ddd, *J* = 5.9, 2.9, 1.5 Hz, 1H), 6.42 (s, 1H), 5.70 (d, *J* = 1.3 Hz, 1H), 3.28 – 3.18 (m, 1H), 2.72 – 2.56 (m, 2H), 2.46 (dd, *J* = 16.1, 12.8 Hz, 1H), 2.32 (ddt, *J* = 18.2, 10.5, 2.6 Hz, 1H), 1.78 (d, *J* = 2.0 Hz, 3H). ^{13}C NMR (125 MHz, $CDCl_3$) δ = 199.2, 171.54, 144.4, 141.6, 135.5, 126.9, 42.8, 36.5, 31.6, 15.6.

6.1.2.2 Through allylic alcohol (**5**): One pot oxidation of the allylic alcohol **5** to carvonic acid **4**

Several direct methods exist for the transformation of alcohols to carboxylic acids.[5] These methods are usually not applied in the case of allylic alcohols to acrylic acids mainly because of the sensitivity of the parent alcohols to the applied acidic conditions. All attempted reactions and their results are presented in Table S4. During our survey, an optimized protocol was developed for the direct, one pot oxidation of **5** to **4** which is highly applicable for scale up. The developed method was based on the copper-catalyzed aerobic oxidation of allylic alcohol to aldehyde **6**, followed by a Lindgren oxidation in the same flask. The method is amenable for scale up (up to 12 mmol of substrate). For comparison of different conditions please advise Spectra 1.

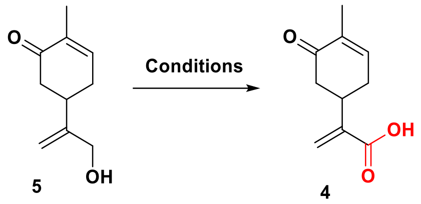

Table S4. Study on the direct oxidation of allylic alcohol **5** to carvonic acid **4**.

Entry[a]	Conditions	Time	Observations	Product	Yield of 8
**1**	Jones reagent 8N (6 equiv), acetone, 0 °C to rt	12h	mixture	Aldehyde (**6**), Acid (**4**)	25%
**2**	PIDA (3 equiv), DCM	12h	Not further oxidation	Aldehyde (**6**)	-
**3**	IBX (0.3 equiv), oxone (1.5 equiv) CH_3CN/H_2O, 2:1, 70 °C	12h	Decomposition	-	-
**4**	Iodobenzoic acid (1 equiv), oxone (1.15 equiv) CH_3CN/H_2O, 2:1, 70 °C,	1 h	Decomposition	-	-
**5**[b]	CuBr 0.75%, Phenanthroline 0.75%, TEMPO 0.75% DMAP 0.75%, O_2, CH_3CN, r.t; followed by Lindgren oxidation in the same pot (see exp below)	12h	Some decomposition is observed; In general, a clean reaction; scalable up to 24 mmol	Acid (**4**)	35%

[a] All reactions have been tested on 7 mmol scale; [b] Reaction has similar yield at higher for up to 12 mmol scale.

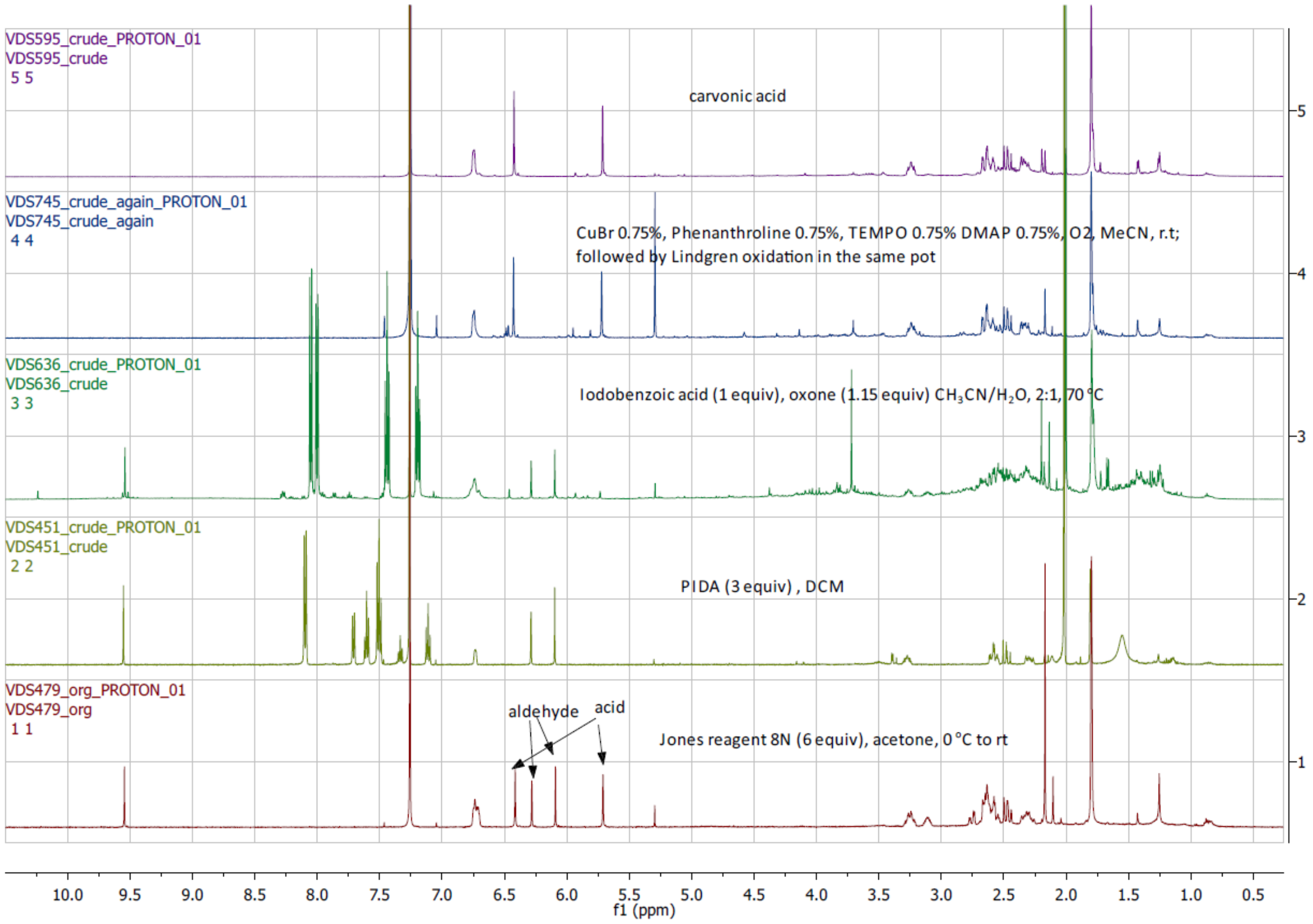

Spectra 1. Screening conditions for the direct oxidation of alcohol **9** to acid **8.**

Experimental procedure to carvonic acid 4 (entry 5, Table 4): Direct oxidation

Compound 5 (1.9 g, 11.44 mmol, 1.0 eq) was dissolved in CH_3CN (4 mL) in a Schlenk apparatus and next followed the sequential addition of the solid reagents as it follows: 1) CuBr (12 mg, 0.086 mmol, 0.75%) 2) Phenanthroline (16 mg, 0.086 mmol, 0.75%), 3) TEMPO (13 mg, 0.086 mmol, 0.75%) and 4) DMAP (21 mg, 0.172 mmol, 1.5%). The Schlenk was then backfilled with O_2, sealed and the reaction mixture was left stirring under O_2 atmosphere for 12 h. Consequently, the reaction mixture was diluted with 50 mL MeCN and the mixture was cooled to 0 °C. Then, $Na_2H_2PO_4$ (618 mg, 5.15 mmol) was dissolved in water (4 mL) and was added to the above mixture, followed by the addition of a solution of H_2O_2 (30% wt, 1.6 mL) and the resulting mixture was stirred at that temperature for 20 minutes. $NaClO_2$ (2 g, 22.88 mmol) was then dissolved in water (19 mL) and added dropwise to the reaction, which was allowed to warm to room temperature and stir for 8 h. The solvent was removed *in vacuo* and the aqueous residue was brought to pH 8.0 with solid and/or saturated aqueous solution of $NaHCO_3$ before it was extracted three times with CH_2Cl_2 (3x5 mL) to remove non-acidic byproducts. The aqueous layer was then brought to pH 3.0 with HCl 6N at 0 °C before it was extracted three times with EtOAc (3x10 mL). The combined organic extracts were dried over $NaSO_4$, filtered and concentrated under reduced pressure to afford pure **Compound 4** (721 mg, 35%, two steps).

6.1.2.3 Synthesis of carvonic acid **4** from allylic chloride **SI-8** (multigram scale)

The direct oxidation of allylic chloride **SI-8** to aldehyde **6** was studied using a modified Kornblum oxidation protocol (Table S5).[6] Trimethylamine N-oxide (TMAO) was found robust to deliver moderate yield of aldehyde, up to the scale of 30 mmol of substrate (60%, entry 4). Solvent modification allowed the one pot oxidation to carvonic acid **4** by sequential application of a Lindgren oxidation.[7] However, when the reaction scale was increased to 133 mmol the yield declined significantly (29%, entry 5). A promising alternative was found to be the use of $NaHCO_3$/NaI (42%, entry 9) for the direct transformation to aldehyde **6**.

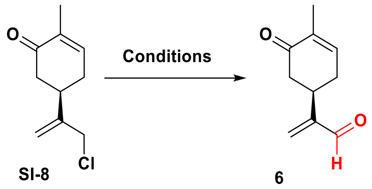

Table S5. Optimization for the direct transformation of allylic chloride **SI-8** to allylic aldehyde **SI-9**.

Entry[a]	Oxidant	Solvent	Temperature	Time	Yield
**1**	*N*-Methylmorpholine-*N*-Oxide (5.0 eq)	DMSO	r.t.	12h	28%
**2**	*N*-Methylmorpholine-*N*-Oxide (5.0 eq)	DMSO	45 °C	12h	32%
**3**	N-Trimethylamine oxide (5.0 eq)	DMSO	r.t.	12h	37%
**4**[b]	N-Trimethylamine oxide (5.0 eq)	DMSO	50 °C	4h	60%
**5**[c]	N-Trimethylamine oxide (5.0 eq)	DMSO	50 °C	4h	29%
**5**	N-Trimethylamine oxide (4.0 eq)	CH_3CN	r.t.	12h	traces
**6**	N-Trimethylamine oxide (5.0 eq)	CH_3CN: H_2O (1:1)	80 °C	12h	40%
**7**	N-Trimethylamine oxide (2.5 eq)	CH_3CN: H_2O (7.5:1)	80 °C	12h	60%
**8**[b]	$NaHCO_3$ (2.0 eq), NaI (1.5 eq)	DMSO	120°C	2h	20%
**9**[d]	$NaHCO_3$ (2.0 eq), NaI (1.5 eq)	DMSO	60°C	3.5h	42%

[a] Reactions at 3 mmol scale unless otherwise noted; [b] 30 mmol scale; [c] 133 mmol scale; [d] 5.5 mmol scale.

Direct transformation of allylic chloride SI-8 to allylic aldehyde 6

To a stirring solution of **compound SI-8** (1.0 g, 5.42 mmol, 1.0 eq) in 2 mL DMSO, $NaHCO_3$ (910 mg, 10.84 mmol, 2.0 eq) and NaI (1.2 g, 8.13 mmol, 1.5 eq) were added in one potion. The reaction mixture was heated at 60°C for 3.5h before it was cooled to room temperate and quenched with 6 mL H_2O. Three extractions with EtOAc (3x6 mL) followed and the combined organic extracts were washed with H_2O twice, dried over $NaSO_4$, filtered and concentrated under reduced pressure. The residue was chromatographed (silica gel) with gradient from 30:1 hexane: EtOAc to 8:1 hexane: EtOAc to afford pure compound **6** (420 mg, 42% yield).

One pot experimental procedure from allylic chloride SI-8 to carvonic acid 4

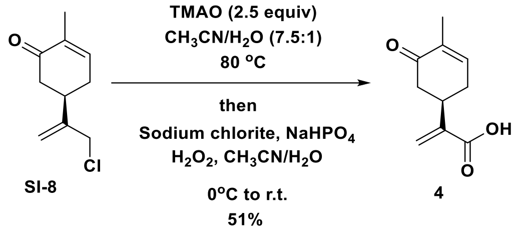

Scheme S7. Direct oxidation of allylic chloride **SI-8** to acid **4**.

Compound SI-8 (76.4 g, 414 mmol, 1 eq) was dissolved in CH_3CN (600 mL) in a round bottomed flask and TMAO (77.8 g, 1.4 mol, 2.5 eq) was dissolved in H_2O (90 mL) and was added to the above solution in one portion. The reaction mixture was refluxed at 80 °C for 12 h before it was allowed to be cooled to room temperature. Then the reaction mixture was then subjected to the conditions of Lindgren oxidation described above to afford pure **Compound 4** (38 g, 51% 2 steps).

6.2 Early-stage introduction of α-methylene-γ-butyrolactone core from carvonic acid 8-Optimization for CH-lactonization.

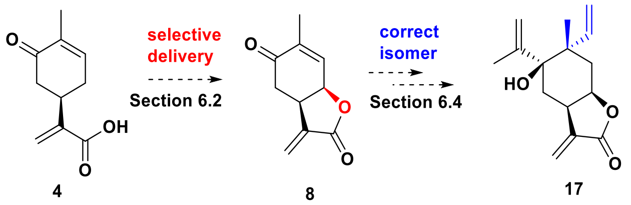

Scheme S8. Steps forward to the development of common scaffold.

Considering the results from section **5.2** (epimeric methyl-group and inability to access the lactone functionalization), we next turned our focus to an early-stage introduction of α-methylene-γ-butyrolactone core from carvonic acid **4**. Carvonic acid **4** was intended to play a dual role, firstly to selectively deliver syn-α-methylene-γ-butyrolactone by utilizing its allylic functionality and secondly to orient the introduction of vinyl- and 2-propenyl chains accordingly, as the *syn*-locked conformation of the lactone moiety was expected by DFT calculations to be oriented vertically to the cyclohexane carbocycle (conducted by Gaussian b3lyp/m06).

Although both 6- and 8-positions are activated enough to enable their functionalization in carvonic acid **4**, several methods to introduce substituents as halogens or hydroxyls failed to deliver clean reactions. Below are given selected examples of our failed routes towards **8** and the optimization attempts for the direct lactonization of carvonic acid **4** via a modified Suarez protocol.

6.2.1 Attempting allylic halogenation

Several halogenation protocols have been attempted for the allylic halogenation of carvonic acid and its subsequent lactonization to **8**. Despite the promising in some cases synthesis of the desired 8,12-lactone **8**, the yields were low (10-15%) due to the formation of overoxidized product **SI-9**, 6,12-lactone **SI-10**, polyhalogenated compounds **SI-11**, and

halolactonization derivatives **SI-12** and **SI-13**, (Table S6). Moderate yields of 6,12-lactone **SI-10** were isolated chemoselectively by using a base (LHMDS) and iron or copper chloride. Mechanistic investigation reveals formation of α-chlorinated ketone intermediate instead of a radical initiated process that leads to compound **SI-10**.

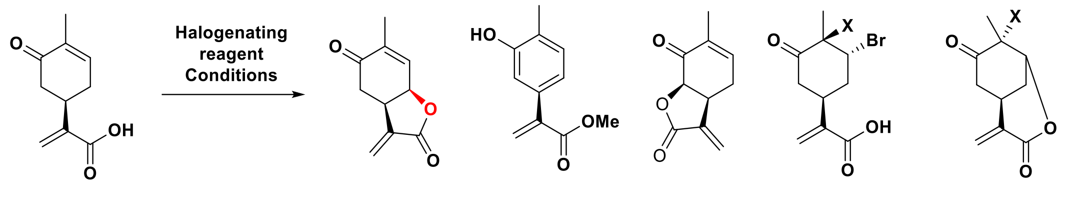

4 8 SI-9 SI-10 SI-11a, X = Br SI-11b, X = OH SI-12, X = Br SI-13, X = I

Table S6. Attempts for the synthesis of **8** utilizing halogenating reagents.

Entry[a]	Conditions	Products	Conversion	Yields for major products
1	NBS (1.1 equiv), AIBN (25 mol%) two portions, CCl_4, 80 °C, 105 min	**8, SI-9, SI-10**	66%	**8** (10%), **SI-9** (21%), **SI-10** (3%)
2	NBS (1.1 equiv), AIBN (15 mol%), CCl_4, 80 °C, 1 h, deoxygenation	Polybrominated products, **8, SI-10**	55%	**8** (10%), **SI-10** (3%)
3	NBS (1.1 equiv), AIBN (15 mol%), CCl_4, 80 °C, 2 h, deoxygenation, microwave	**8, SI-9, SI-10**	52%	**8** (10%), **SI-9** (30%), **SI-10** (6%)
4	NBS (1 equiv), AIBN (15 mol%), CCl_4, 80 °C, 7 h	**8, SI-9, SI-10**	100%	**8** (12%), **SI-9** (80%), **SI-10** (4%)
5	NBS (1.5 equiv), AIBN (15 mol%), Benzene, 80 °C, 1 h	**8, SI-12**	100%	**8** (19%), **SI-12** (70%)
6[b]	NBS (1 equiv), AIBN (10 mol%), Benzene, 80 °C, 1h, deoxygenation	**8, SI-9**	100%	**8** (35%), **SI-9** (19%)
7	NIS (1 equiv), AIBN (10 mol%), Benzene, 80 °C, 1 h, deoxygenation	**SI-13, unidentified products**	60%	**SI-13** (31%)
8	NCS (1 equiv), AIBN (10 mol%), Benzene, 80 °C, 1 h, deoxygenation	Complex mixture of products	75%	-
9	NBS (1 equiv), UV light, Benzene, rt, 2 h	Complex mixture of products	-	-
10	NBS (1 equiv), Et_3B, Benzene, -20 °C, 1 h	Complex mixture of products	-	-
11	NBS (1 equiv), benzoyl peroxide (10 mol%), Benzene, 80 °C, 1.5 h, deoxygenation[8]	**8, SI-9, SI-10**	73%	**8** (22%), **SI-9** (19%), **SI-10** (7%)
12	Br_2 (1 equiv), in two portions Et_2O, -78 °C to -30 °C 100 min and then rt for 30 min	**SI-10, SI-11, SI-12**	82%	**SI-10** (40%), **SI-11a** (16%) **SI-11b** (8%), **SI-12** (18%)
13	I_2 (1 equiv), in two portions Et_2O, -78 °C to -30 °C 100 min and then rt for 30 min	Complex mixture of products	-	-
14	I_2 (1 equiv) UV-VIS light, Benzene,	**SI-13 and minor polyiodination**	78%	**SI-13** (28%)
15	LHMDS (2 equiv), $FeCl_3$ (4 equiv), THF, -78 to -15 °C, o/n	**SI-10**	30%	**SI-10** (20%)
16	LHMDS (2 equiv), CuOTf (4 equiv), THF, -78 to -15 °C, o/n	-	0	-
17	LHMDS (2 equiv), $Fe(acac)_3$ (4 equiv), THF, -78 to 5 °C, o/n	-	0	-

**18**	LHMDS (2 equiv), $CuCl_2$ (4 equiv), THF, -78 °C to rt, o/n	**SI-10**	35%	**SI-10** (35%)
**19**	LHMDS (2 equiv), $TiCl_4$ (4 equiv), THF, -78 °C to rt, o/n	-	0	-
**20**	LHMDS (2 equiv), $ZnCl_2$ (4 equiv), THF, -78 °C to rt, o/n	-	0	-
**21**	KHMDS (2 equiv), $CuCl_2$ (4 equiv), THF, -78 °C to rt, o/n	**SI-10**	18%	**SI-10** (15%)
**22**	LHMDS (2 equiv), CuI (4 equiv), THF, -78 °C to rt, o/n	-	0	-

[a] All reactions were conducted in up to 2.22 mmol scale unless otherwise noted. [b] Reaction was scaled up to 19.4 mmol.

3a*R*,7a*R*)-6-methyl-3-methylene-3a,7a-dihydrobenzofuran-2,5(3H,4H)-dione (8).

To a stirring solution of **compound 4** (3.5 g, 19.4 mmol, 1.0 eq) in thoroughly degassed benzene (350 mL) equipped by refluxed condenser and argon balloon, AIBN (318 mg, 0.19 mmol, 10% mmol) and NBS (3.45 g, 19.4 mmol, 1.0 eq) were added successively and the reaction mixture was left at reflux for 1h sharp. After being cooled down, the reaction was quenched with saturated aqueous $NaHCO_3$. The two layers were separated and the organic layer was extracted one time with saturated aqueous $NaHCO_3$ and two times with saturated aqueous $Na_2S_2O_3$.The combined aqueous layers were extracted one time with benzene. The combined organic extracts were dried over Na_2SO_4, filtered and concentrated under reduced pressure. The residue was chromatographed (silica gel) with gradient from 5:1 hexane: EtOAc to 1:1 hexane: EtOAc [R_f = 0.5 (hexane:EtOAc = 1:3, UV active on TLC, stains purple upon *p*-anisaldehyde staining)] to afford pure **compound 8** as a white amorphous solid (1.2 g, 35%). $[\alpha]^{20}_{D}$ = -68.6 (*c* 0.7, $CHCl_3$). HRMS (ESI, m/z): calcd. for $C_{10}H_{11}O_3^+$ ($[M+H]^+$): 179.0703, found: 179.0702. ^1H NMR (500 MHz, $CDCl_3$) δ = 6.53 – 6.48 (m, 1H), 6.33 (d, *J* = 3.1 Hz, 1H), 5.64 (d, *J* = 3.1 Hz, 1H), 5.31 – 5.25 (m, 1H), 3.74 – 3.66 (m, 1H), 2.89 – 2.80 (m, 2H), 1.82 (s, 3H). ^{13}C NMR (125 MHz, $CDCl_3$) δ = 195.0, 168.8, 138.5 137.2, 136.5, 122.7, 73.1, 38.3, 37.1, 15.9.

6.2.2 Attempting allylic hydroxylation-alkoxylation

Despite the extended literature present on the topic of allylic hydroxylation and alkoxylation, none of the tested methods provided even traces of the desired products. Utilization of singlet oxygen and selenium dioxide resulted inseparable complex mixture of products while radical lactonization with the aid of strong oxidants as $K_2S_2O_8$ led to decomposition. Attempts to hydroxylate the allylic position by the oxidation of the *γ*-stabilized enol form provided only traces of hydroxylated intermediate **SI-14**. Efforts to use carboxylic acid as directing group for palladium-mediated CH-activation of allylic position,[9] resulted only in unreacted starting material.

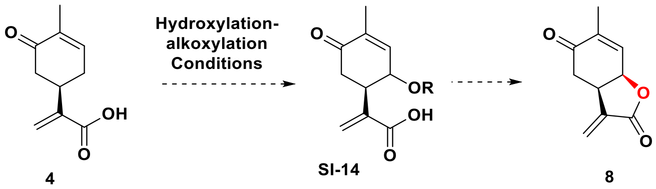

Table S7. Selected attempts for hydroxylation and CH-lactonization of carvonic acid **8**.

Entry	Hydroxylation reagent	Product(s)	Conversion	Yield

**1**	O_2, methylene blue, VIS light 300W, CH_3CN, rt, 3 days	Unreacted SM	-	-
**3**	t-BuOK (2 equiv), EtOH, rt, O_2[10]	SM	0	-
**4**	CH_3MgBr (5 equiv), $FeCl_3$ (1.2 equiv), TMSCl, (1.2 equiv), nitrosobenzene (1.5 equiv), 0 °C to rt, 5 hr[11]	Hydroxylated product **SI-14**	40%	15%
**5**	$K_2S_2O_8$ (2 equiv), $Cu(OAc)_2$ (10 mol%), CH_3CN, 80 °C, 12h[12]	Decomposition	-	-
**6**	$K_2S_2O_8$ (2 equiv), $Cu(OAc)_2$ (10 mol%), DMF/H_2O 5:1, 80 °C, 12h[12]	Decomposition	-	-
**7**	SeO_2 (0.5 eq), t-BuOOH (3 equiv), CH_3CN/H_2O, rt to 50 °C, 12h [13]	Complex mixture of products	-	-
**8**	$Pd(OAc)_2$ (10 mol%), oxone (1.5 equiv), K_2CO_3 (1 equiv), 1,4-dioxane, rt, 12h	Unreacted SM	-	-
**9**	$Pd(Oac)_2$ (5 mol%), oxone (1.5 equiv), K_2CO_3 (1 equiv), DMSO 110 °C, 12h	SM	-	-
**10**	$Pd(OAc)_2$ (10 mol%), benzoquinone (2 equiv), DMSO 130 °C, 12h	**SI-10**	20%	8%
**11**	$Pd(OAc)_2$ (10 mol%), benzoquinone (2 equiv), DMSO, AcOH 70 °C, 12h	**SI-10**	20%	8%
**12**	$Pd(OAc)_2$ (10 mol%), PIDA (1.5 equiv), tBuOK (2 equiv), t-BuOH, 80 °C, 12h	Unreacted SM	-	-

6.2.3 Carboxylic acid as directing group for radical CH-lactonization-modified Suarez conditions- Postulated reaction mechanism

After extensive experimentation, modified Suarez conditions[14] were found the only protocols to succeed clean transformation to 8,12-lactone **8**. Based on that, irradiation with visible light of carvonic acid with PIDA (4 equivalents) and iodine (1 equivalent) resulted the formation of **8** in up to 45% yield (78% brsm). After its optimization (Table S8), the protocols described above allows the preparation of 8,12-lactone in gram scale in the absence of iodo-lactonization product **SI-13**, 6,12-lactone **SI-10** or polyiodinated compounds.

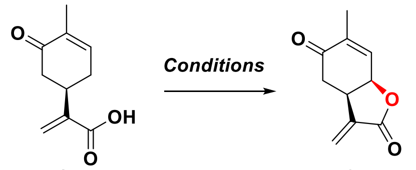

4 8

Table S8. Optimization conditions for direct lactonization of carvonic acid **8**.

Entry	Keep	PIDA equiv	Solution of I_2 (equiv)	Screen	Change	Time	Yield
**1**		1.1	1.1 in DCE	Light	Fluorescent lamp (400W) (absorption max 630-610, 550-540, 430-420 nm)	5	13%
**2**		1.1	1.1 in DCE		UV light (400-315 nm)	5	<5%
**3**		1.1	1.1 in DCE		Mercury lamp (400 W)(absorption max 580, 545, 436 nm)	5	16%

**4**		1.1	1.1 in DCE		Xenon lamp (400 W) (absorption max 919, 886, 827 nm)	5	-
**5**	Fluorescent light	1.1	0.5	Solvent	1,2-DCE	5	10%
**6**		1.1	0.5		CH_3CN	5	<5%
**7**		1.1	0.5		$CHCl_3$	5	-
**8**		1.1	0.5		1,4-Dioxane	5	-
**9**		1.1	0.5		Benzene	5	21%
**10**	Benzene	2.2	0.5	Equivalents and portions	1 portion of I_2 0.057M	5	12%
**11**		1.1	1.1		1 portion of I_2 0.057M	5	18%
**12**		2.2	1.1		2 portions of I_2 0.057M	5	27%
**13**		4	1		2 portions of I_2 0.057M	5	32%
**14**	Large excess of PIDA	4	1	Dilution and rate	I_2 0.028M in 0.8 ml/min by syringe pump	5	43%
**15**		4	1		I_2 0.035M in 0.6 ml/min by syringe pump	8	37%
**16**		4	1		I_2 0.035M in 0.3 ml/min by syringe pump	5	37%
**17**		4	1		I_2 0.035M in 0.1 ml/min by syringe pump	3	45% (78% brsm)

[a] All reactions were set at 500mg of carvonic acid **4**; Keep column corresponds to factors that we use for all proceeding tries. The reactions were performed by dissolving carvonic acid in 50 ml of the indicated solvent followed by the addition of PIDA. The cloudy mixture is stirred for 5 min before the addition of iodine as a solution in the indicated solvent. Dilution of iodine is 0.057M unless otherwise noted. In most cases minor amount of **SI-13** (<5%) is observed.

In order to investigate the reaction mechanism, several reactions were monitored by NMR. When carvonic acid **4** was dissolved in $CDCl_3$ followed by PIDA, a rapid formation of two iodo-complexes were observed in a ratio of 3:1 with the subsequent loss of acetic acid. The two complexes were identified as the mono- and bis-acid phenyl-iodo acetates **7a** and **7b** (Spectra 2). The complexes remained stable throughout the pass of several hours in the absence of water. On the contrary, when the same solution was irradiated with fluorescent light (peaks max 630-610 nm and 550-540 nm) a cleavage was witnessed to form the carvonic acid and iodobenzene, indicating the slow production of IOAc (Spectra 3). The slow addition of iodine solution in freshly prepared **7a** and **7b** by utilizing excess of PIDA, followed by irradiation lead to slow formation of 8,12-α-methylene-γ-butyrolactone **8** with expulsion of iodobenzene (Spectra 4). It is interesting to notice that addition of 1 equivalent of water accelerates the formation of 8,12-lactone **8**, majorly in the expense of complex **SI-15**. Extended irradiation in the presence of water finally hydrolyze complexes back to carvonic acid. Addition of extra equivalents of PIDA and iodine can turn on again the reaction boosting the formation of the desired product (Spectra 4). Considering these observations, we postulate that the mechanism involves the slow radical cleavage of mono- and bis-acid complexes to **SI-16** (carboxylic iodo), following by the production of carboxyl-radical **SI-17** (Scheme S9). 1,5-Hydrogen atom transfer serves as the crucial factor for the observed chemoselectivity, compared to other methods (Spectra 5). Finally, addition of water seems to play an analogous role to the activation of other

hypervalent iodine reagents, producing compound **SI-18**, that is readily cleaved upon irradiation to produce **8** (Scheme S9).

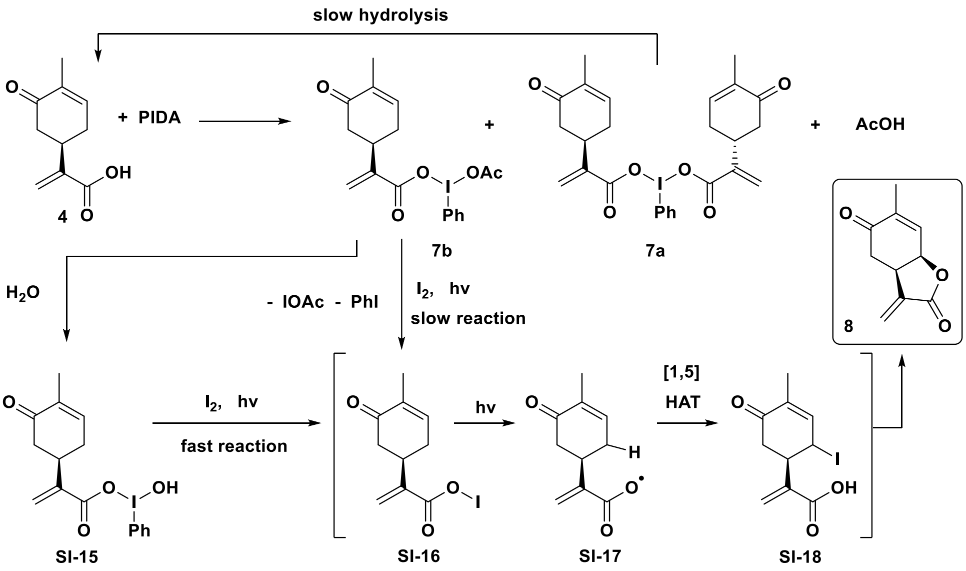

Scheme S9. Postulated reaction mechanism.

Optimization of the reaction (Table S8) as evidenced by H^1NMR shows that irradiation by visible light (fluorescent light or light produced by mercury lamp max absorption peaks at 540-570nm and 440-420nm) led to best results while irradiation with UV and xenon lamp provided complex mixture of products or unreacted starting material respectively (Spectra 6). Irradiation under different solvents indicated a cleaner reaction profile for benzene, while the slow addition (0.1ml/min) of 1 equivalent of iodine in high dilution was found extremely advantageous (Spectra 7-9).

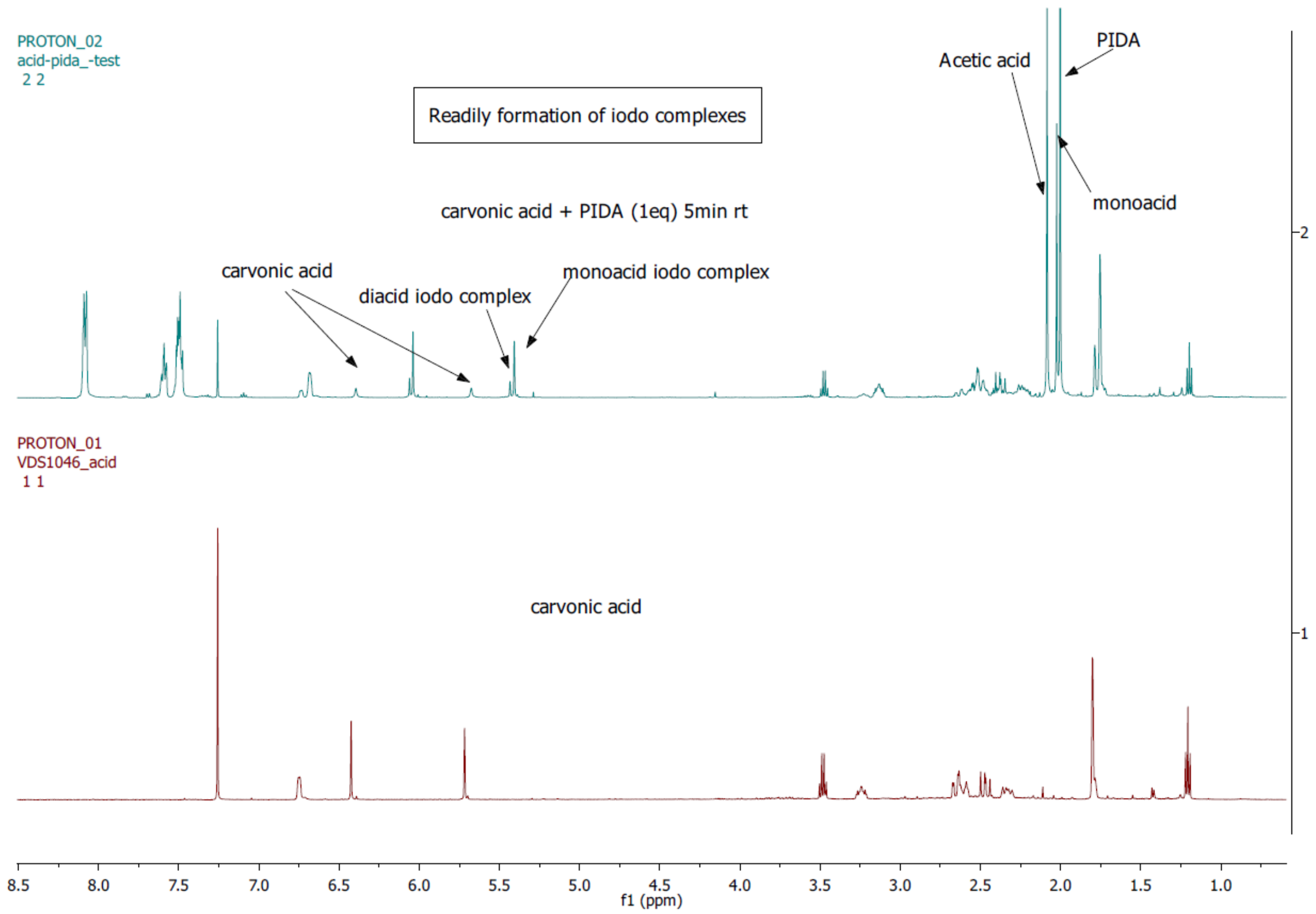

Spectra 2. Formation of iodo-complexes **7a** and **7b**.

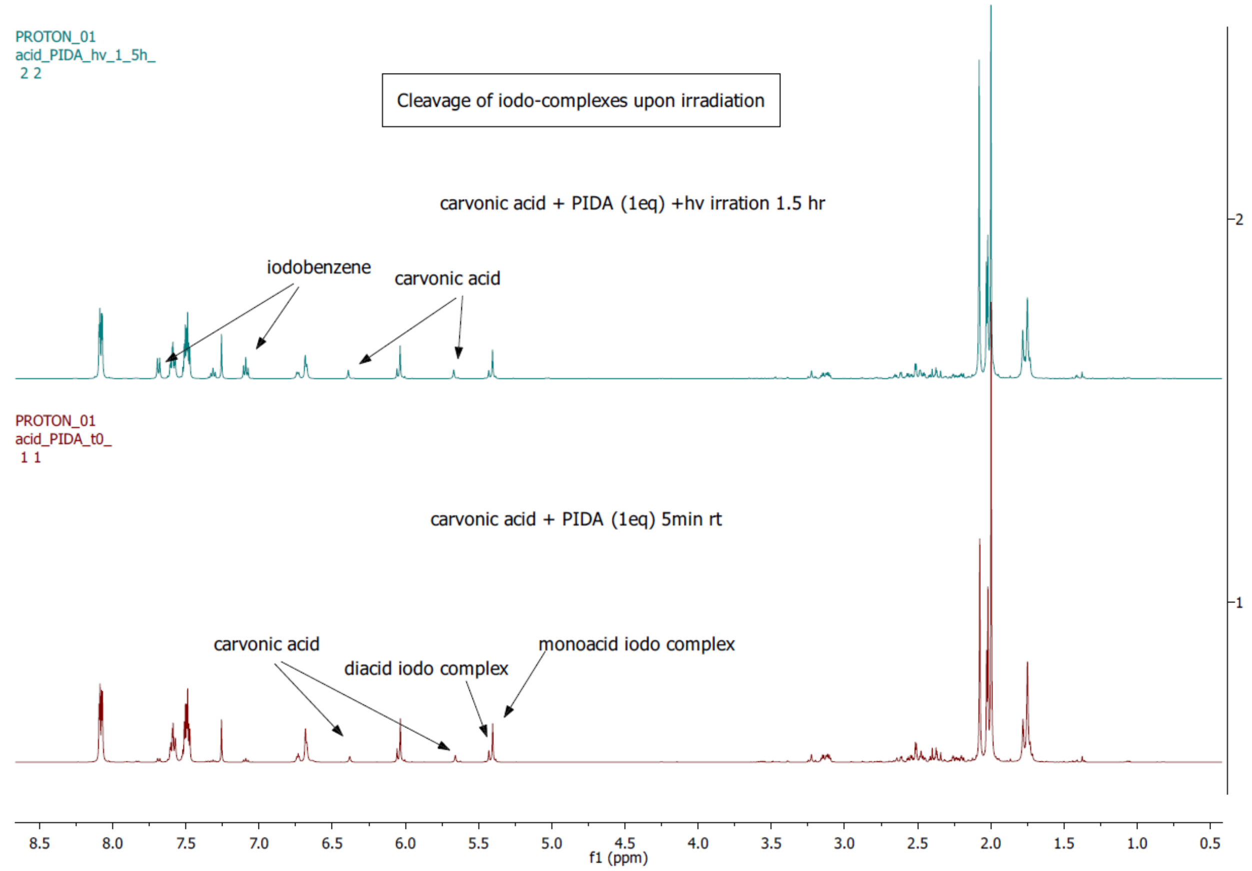

Spectra 3. Cleavage of iodo-complexes in the presence of fluorescent light.

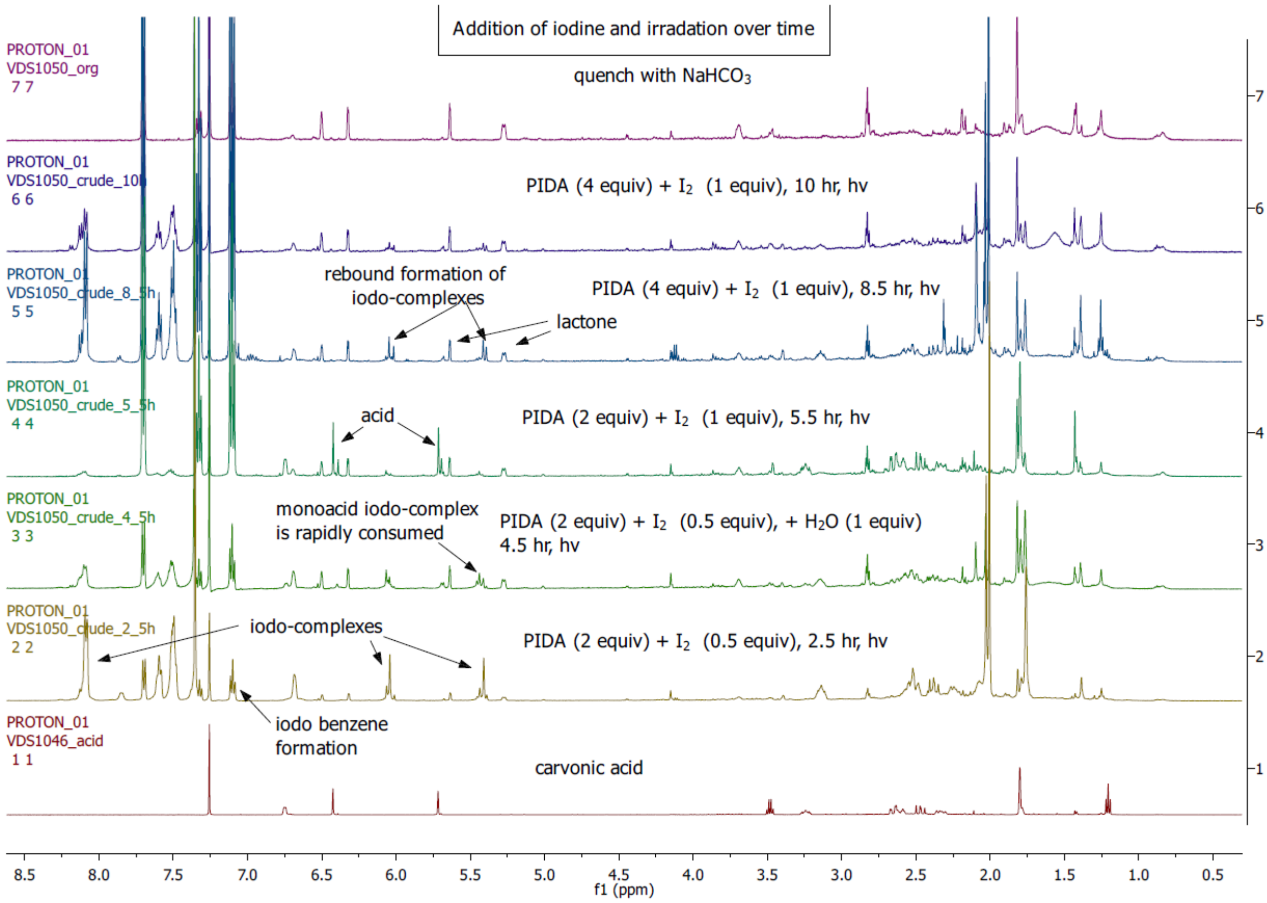

Spectra 4. Formation of 8,12-lactone over time from complexes **10a-10b** with the aid of iodine and light.

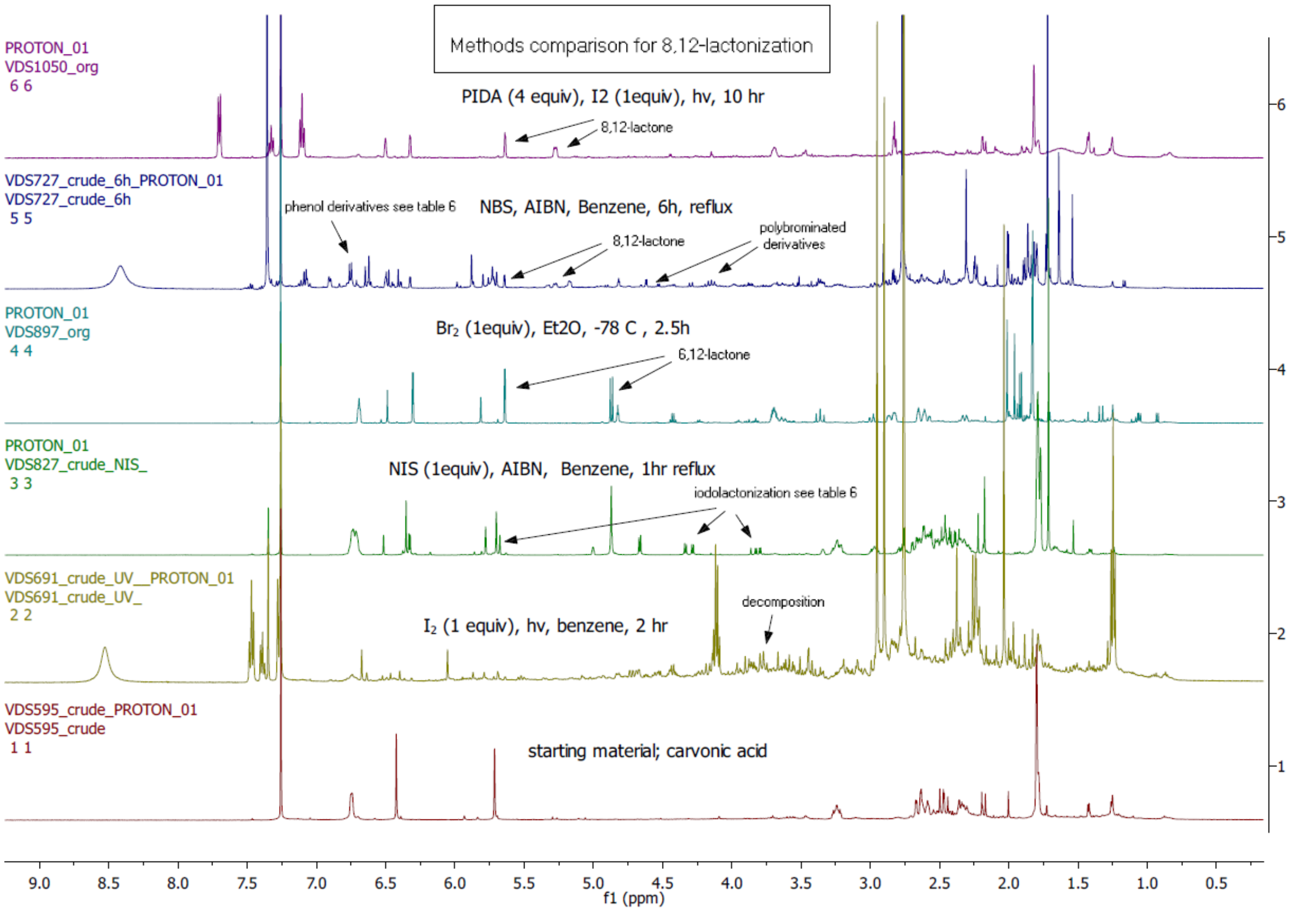

Spectra 5. Comparison of different methods to achieve 8,12-lactonization.

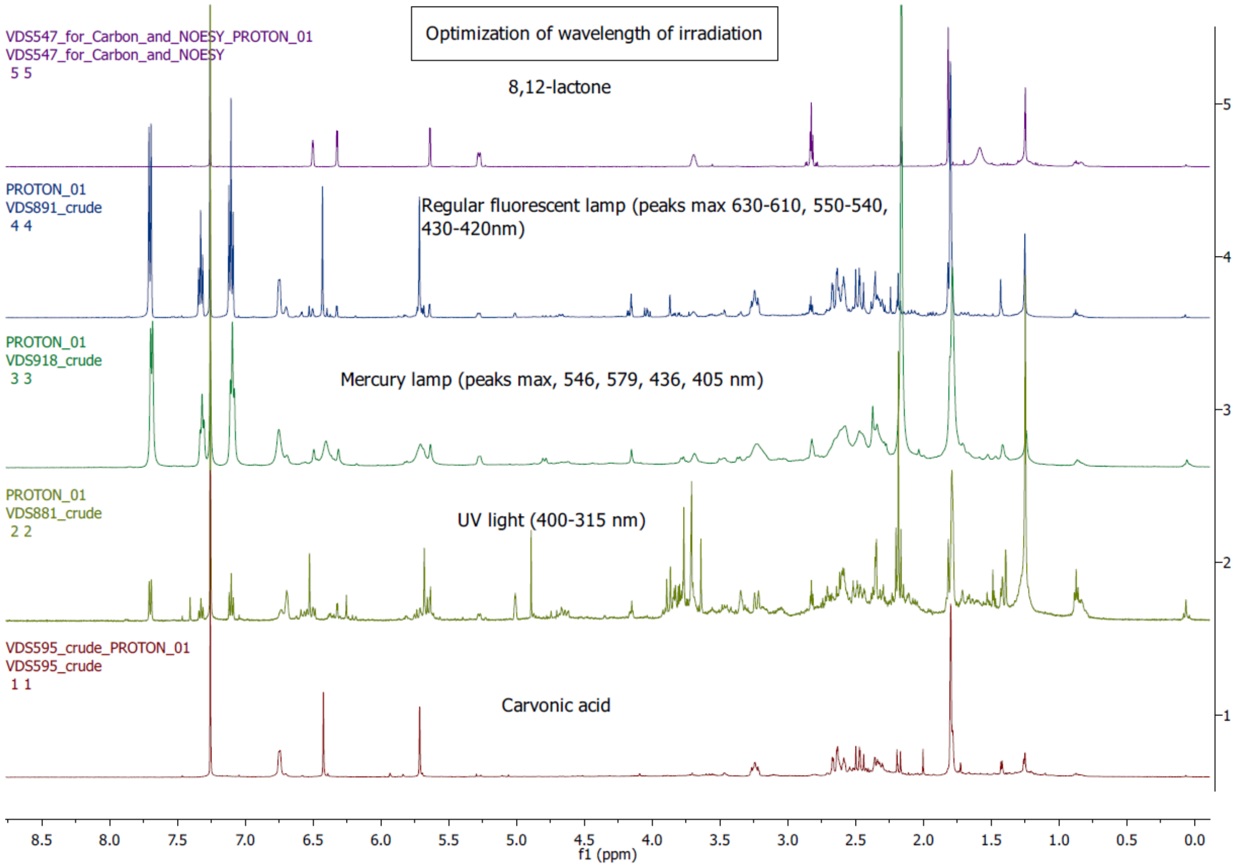

Spectra 6. Screening of irradiation source.

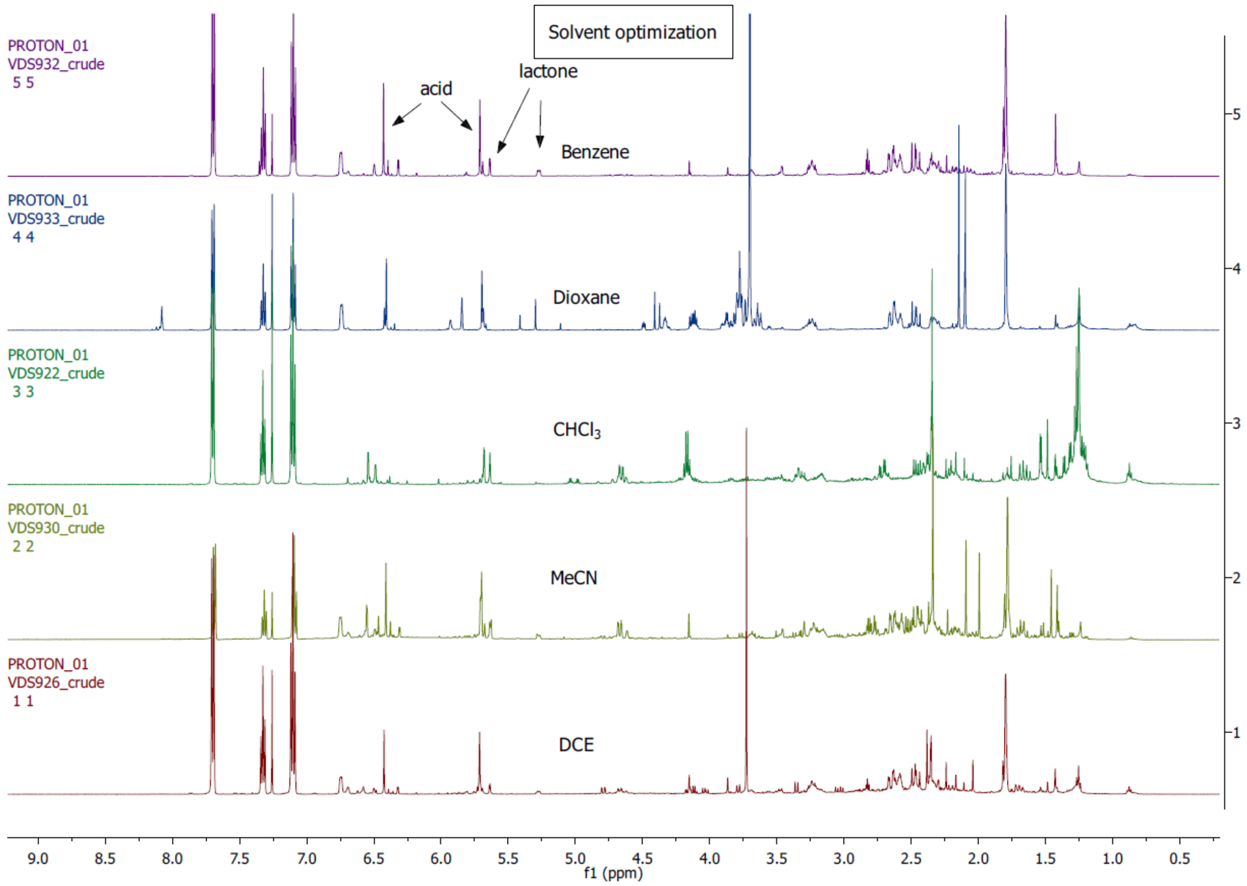

Spectra 7. Screening of solvents.

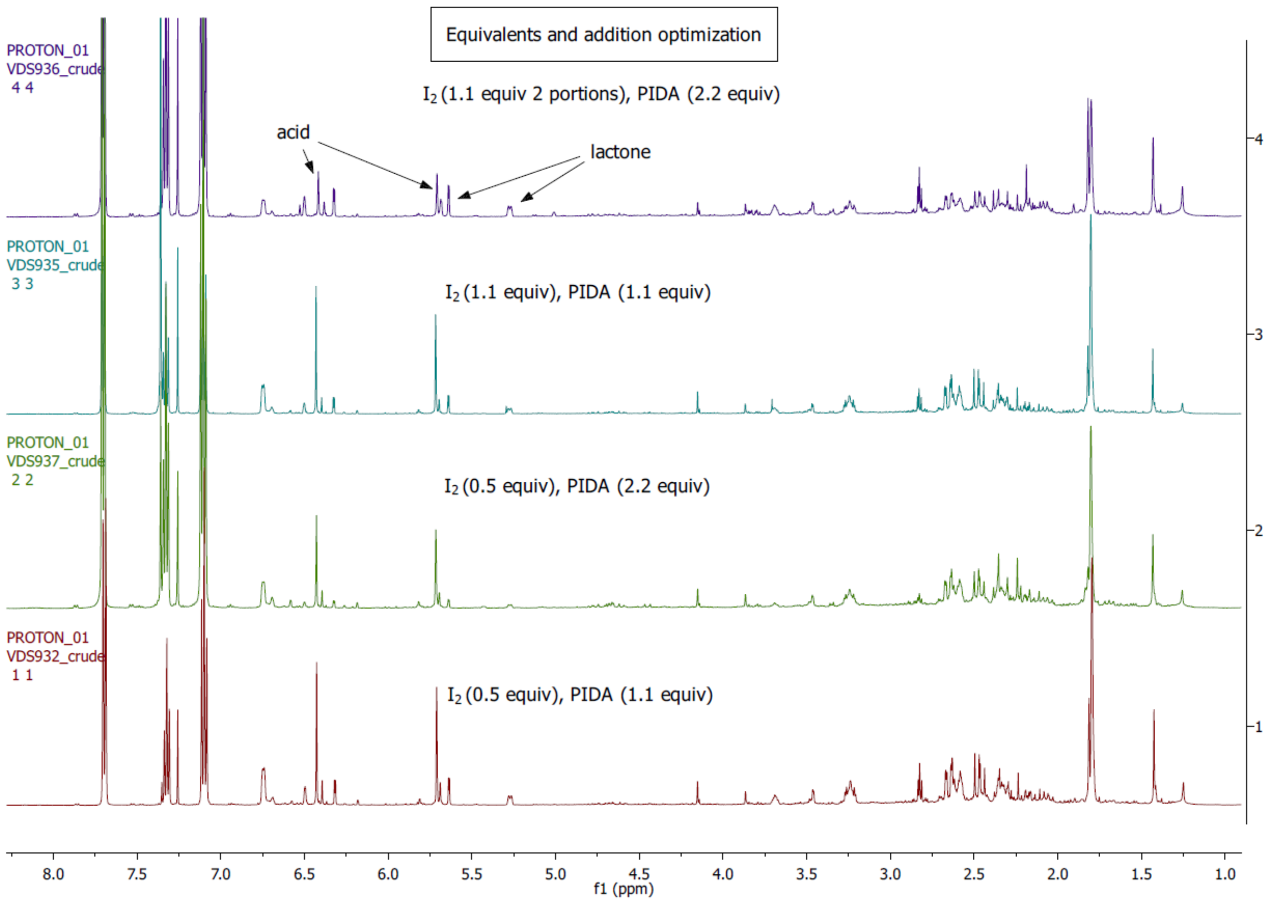

Spectra 8. Screening equivalents.

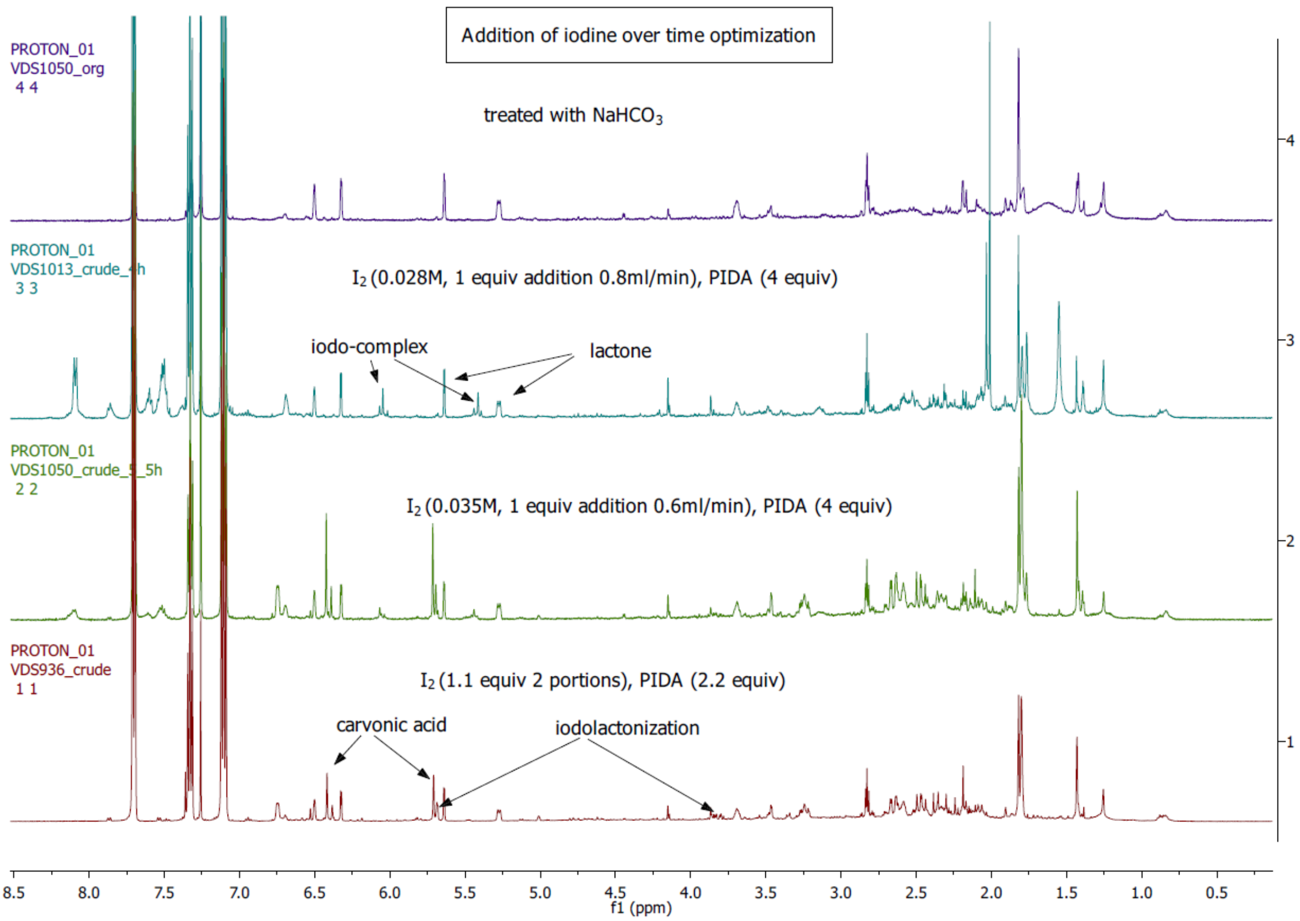

Spectra 9. Screening the rate of iodine addition.

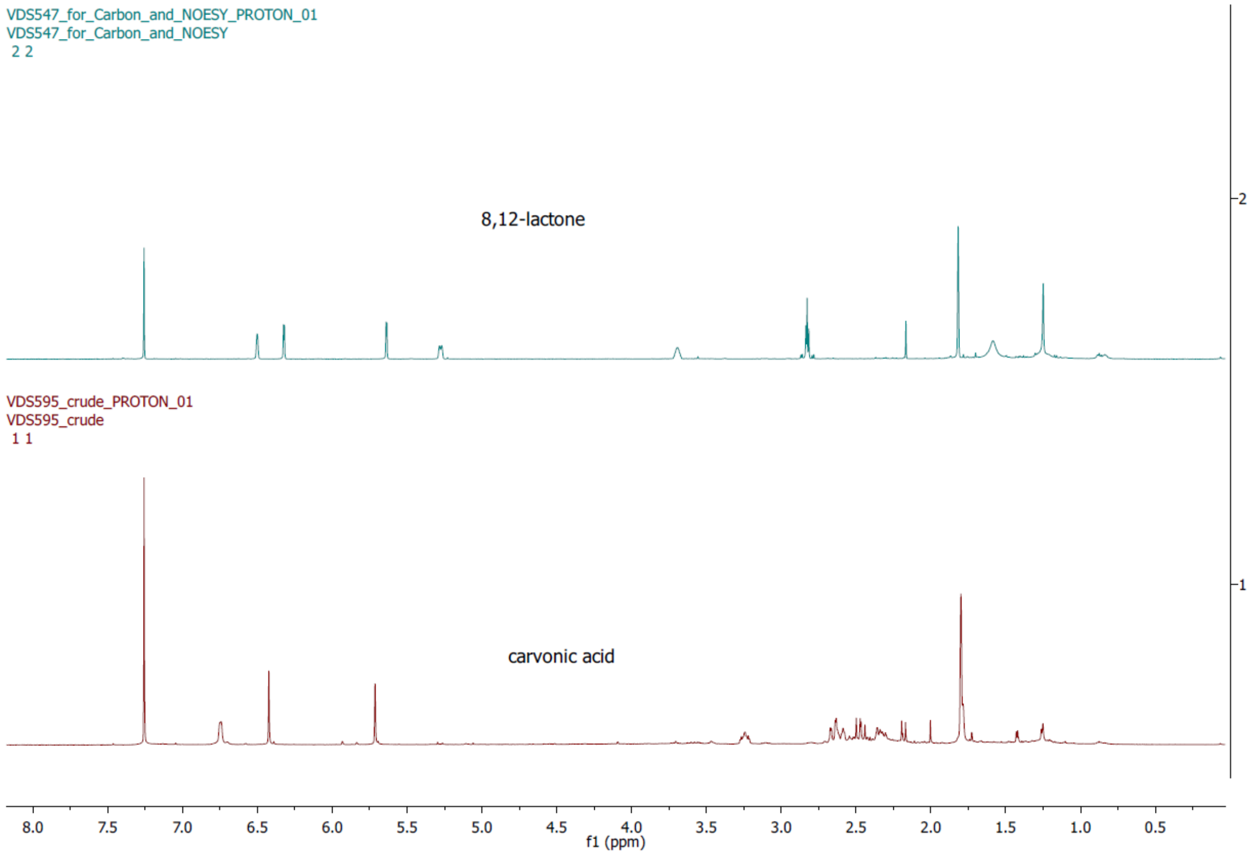

Spectra 10. Comparison of carvonic acid **4** and the desired 8,12-lactone **8**.

Photochemical radical CH-lactonization of compound 4 to the desired 8 (Modified Suarez Reaction)

Compound 4 (500 mg, 2.77 mmol, 1.0 eq) was dissolved in benzene (50 mL) in a light permeable glass tube and PIDA (3.57 g, 11.08 mmol, 4.0 eq) was added to the above solution. The resulting mixture was left stirring at room temperature under Ar for 10 min before it was exposed to visible light with simultaneous addition of H_2O (50 µL, 2.77 mmol, 1.0 eq) followed by the dropwise addition of a solution of iodine (703 mg, 2.77 mmol, 1.0 eq) in 80 mL benzene with the aid of syringe pump (addition rate: 0.1 mL/min). After the addition was over, the reaction mixture was left under visible light irradiation for additional 8 h before it was quenched with saturated aqueous solution of $Na_2S_2O_3$ (20 mL), whereupon it was left stirring for 12 h. The aqueous layer was then separated and extracted three times with EtOAc (3x10 mL). The combined organic layers were washed twice with saturated aqueous $NaHCO_3$ (2x30 mL), dried over $NaSO_4$, filtered, and concentrated under reduced pressure. The residue was recrystallized from CH_3OH to afford pure **Compound 8** (222 mg, 45%).

6.3 Final alkylation for the synthesis of common scaffold 19.

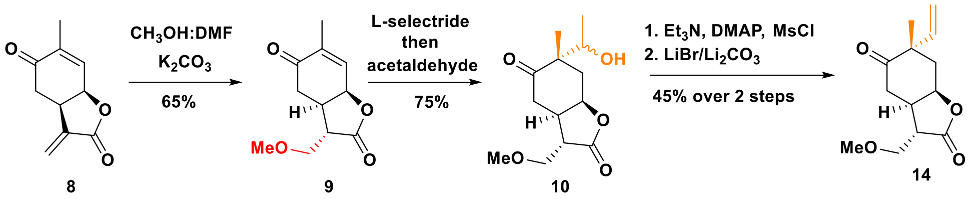

Scheme S10. Introduction of the α-vinyl chain

Following the successful 8,12-lactonization, the introduction of syn vinyl and isopropenyl chains remained towards the completion of common scaffold **3**. Attempts to introduce the vinyl chain in the presence of *α*-methylene-*γ*-butyrolactone functionality failed, due to the highly reactive nature of the lactone. Based on that, protection of methylene group was surveyed. The chemoselective protection of α-methylene side was accomplished by the reversible introduction of methyl ether with K_2CO_3 and CH_3OH in a mixture of DMF. The dilution of the substrate (<0.1M) and the ratio of DMF (DMF:CH_3OH= 3:1) is a crucial factor for the completion of the reaction (Table S9). We must note that unreacted starting material is inseparable from the product.

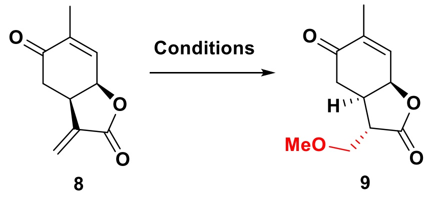

Table S9. Optimization for the protection of **8**.

Entry[1]	Base	Solvent	Conversion	Yield of 12
**1**	CH_3ONa (1.5 equiv), rt, 2 h	CH_3OH	100%	Complex mixture of products
**2**	K_2CO_3 (0.2 equiv), rt, 3 h	CH_3OH	55%	Complex mixture of products
**3**	K_2CO_3 (0.2 equiv), -78 °C to rt, 5 h	CH_3OH	30%	25%
**4**	Substrate >0.2M K_2CO_3 (1 equiv), -78 °C, 6 h	CH_3OH	68%	45%
**5**	Substrate <0.1M, K_2CO_3 (1 equiv), -20 °C, 4 h	CH_3OH: DMF 3:1	84%	59%
**6**	Substrate <0.1M, K_2CO_3 (2 equiv), -20 °C, 4 h	CH_3OH: DMF 1:3	100%	71%

[1] Yields reported as calculated by H^1NMR with internal standard.

Optimization attempts as monitored by NMR are shown below (Spectra 11).

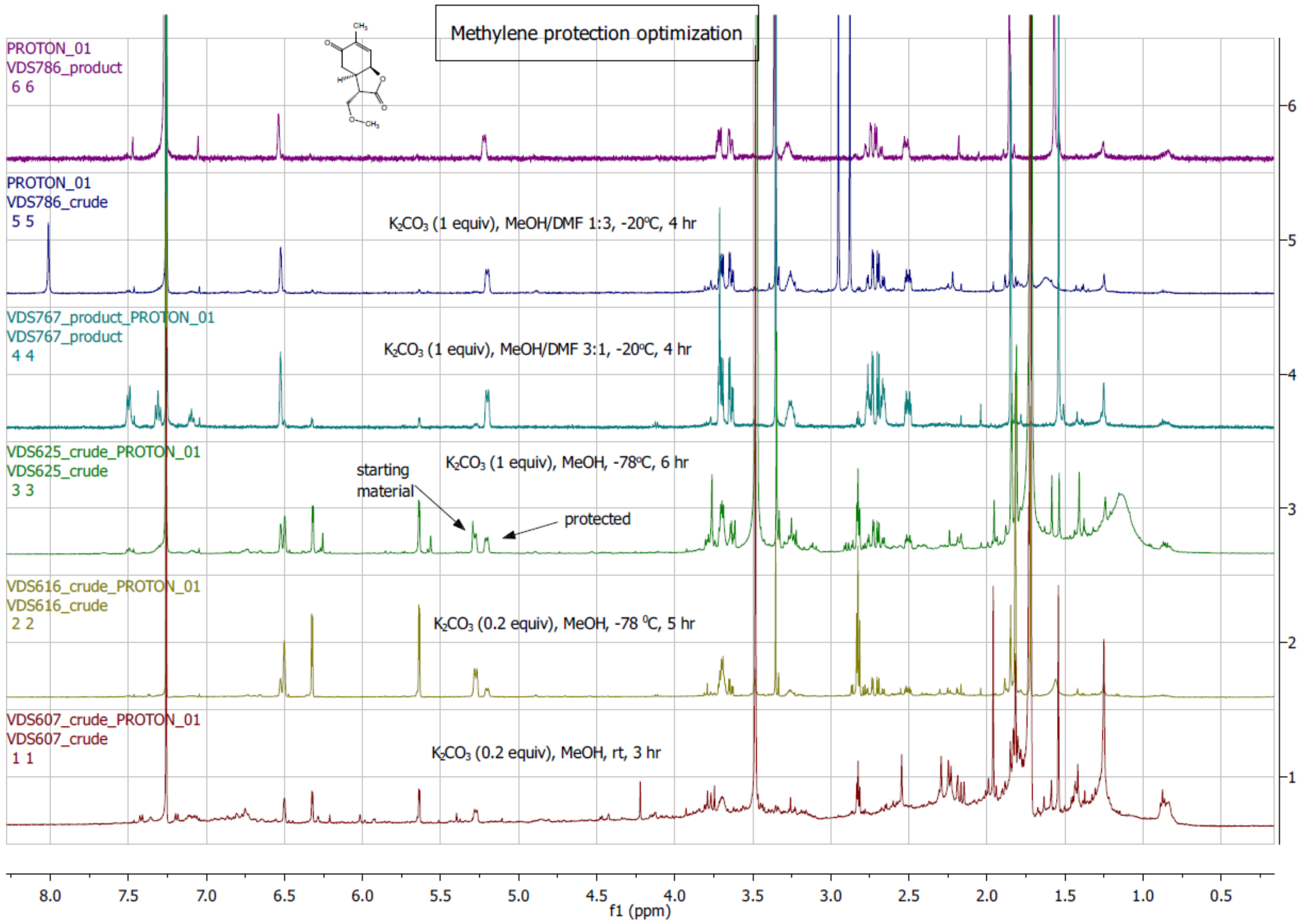

Spectra 11. Screening conditions for α-methylene protection.

Following the protection, conjugated reduction of **9** by L-selectride and subsequent addition of acetaldehyde provided compound **10** in 75% yield, as a mixture of diastereoisomers majoring the Zimmerman-Traxler predicted alcohol. Mesylation followed by elimination using LiBr/Li_2CO_3 led to compound **14** in 34% yield over 2 steps.

(3*S*,3a*R*,7a*S*)-3-(methoxymethyl)-6-methyl-3a,7a-dihydrobenzofuran-2,5(3H,4H)-dione (9)

To a stirring solution of **Compound 8** (3 g, 16.83 mmol, 1.0 eq) in a mixture of DMF/CH_3OH 3:1 (180/60 mL) at -20 °C, K_2CO_3 (4.6 g, 33.66 mmol, 2 eq) was added in one portion and the reaction mixture was left stirring at that temperature for 4 h. Upon completion, 100 mL of Et_2O were added and the reaction mixture was left at rest without stirring at -20 °C for 30 min for any excess of K_2CO_3 to be precipitated, before it was filtered under vacuum and quenched with saturated aqueous NH_4Cl (90mL). After separation, the aqueous layer was extracted two times with Et_2O (2x150 mL) and two times with EtOAc (2x50 mL). The combined organic extracts were washed once with H_2O (120 mL), dried over Na_2SO_4 and concentrated under reduced pressure. The residue was chromatographed (silica gel) with gradient from 3:1 hexane: EtOAc to 1:1 hexane: EtOAc [R_f= 0.67 (hexane:EtOAc = 1:3, UV inactive on TLC, stains brown upon *p*-anisaldehyde staining)] to afford pure **Compound 9** as a white amorphous solid (2.5 g, 71%). $[\alpha]^{20}_D$ = -70 (*c* 0.2, $CHCl_3$). HRMS (ESI, m/z): calcd. for $C_{11}H_{14}O_4Na^+$ ($[M+Na]^+$): 233.0784, found:233.0783. ^1H NMR (500 MHz, $CDCl_3$) δ = 6.54 – 6.50 (m, 1H), 5.19 (dt, *J* = 7.0, 2.1 Hz, 1H), 3.69 (dd, *J* = 9.8, 4.8 Hz, 1H), 3.63 (dd, *J* = 9.8, 3.5 Hz, 1H), 3.34 (s, 3H), 3.30 – 3.21

(m, 1H), 2.70 (qd, *J* = 17.2, 4.8 Hz, 1H), 2.50 (dt, *J* = 10.5, 4.1 Hz, 1H), 1.84 (s, 3H). ^{13}C NMR (125 MHz, $CDCl_3$) δ = 196.0, 175.2, 138.5, 137.6, 73.7, 68.9, 59.3, 44.7, 37.2, 36.9, 15.8.

(3*S*,3a*R*,6*R*,7a*R*)-6-(1-hydroxyethyl)-3-(methoxymethyl)-6-methyltetrahydrobenzofuran-2,5(3H,4H)-dione (10)

To a flame-dried round-bottom flask, **Compound 9** (3 g, 14.27 mmol, 1.0 eq) was dissolved in THF (100 mL) under Ar atmosphere and was cooled to -78 °C. L-Selectride (14.3 mL, 14.27 mmol, 1.0 M in THF, 1.0 eq) was then added and the resulting mixture was stirred for 1 h at -78 °C. Acetaldehyde (4.8 mL, 85.62 mmol, 6eq) was then added and the reaction mixture was stirred for 1 h at the same temperature before it was quenched with saturated aqueous NH_4Cl (15 mL). The resulting mixture was allowed to warm to room temperature and stir vigorously for an additional hour. The aqueous layer was separated and extracted three times with EtOAc (3x80 mL). The combined organic extracts were dried over $NaSO_4$ and concentrated under reduced pressure. The residue was chromatographed (silica gel) with gradient from 8:1 hexane: EtOAc to 1:2 hexane: EtOAc [R_f= 0.47 (hexane:EtOAc = 1:3, UV inactive on TLC, stains brown upon *p*-anisaldehyde staining)] to afford pure **Compound 10** as a white amorphous solid (2.6 g, 71%). HRMS (ESI, m/z): calcd. for $C_{13}H_{20}O_5Na^+$ ($[M+Na]^+$): 279.1203, found: 279.1203. ^{1}H NMR (500 MHz, $CDCl_3$) δ = 4.96 (td, *J* = 7.7, 5.5 Hz, 1H), 3.84 (dq, *J* = 6.6, 3.8 Hz, 1H), 3.70 (dd, *J* = 9.5, 4.7 Hz, 1H), 3.60 (dd, *J* = 9.4, 3.4 Hz, 1H), 3.34 (s, 3H), 3.06 (m, 1H), 2.68 (dd, *J* = 14.7, 6.8 Hz, 1H), 2.55 – 2.45 (m, 2H), 2.30 (dd, *J* = 15.0, 5.5 Hz, 1H), 1.98 (dd, *J* = 15.1, 7.6 Hz, 1H), 1.19 (d, *J* = 6.4 Hz, 3H), 1.10 (s, 3H). ^{13}C NMR (125 MHz, CDCl3$_3$) δ = 214.8, 176.1, 75.5, 71.3, 70.8, 59.2, 50.2, 48.0, 41.3, 37.9, 37.5, 18.7, 17.6.

(3*S*,3a*R*,6*S*,7a*R*)-3-(methoxymethyl)-6-methyl-6-vinyltetrahydrobenzofuran-2,5(3H,4H)-dione (14)

To a round bottom flask anhydrous Et_3N (3.82 mL, 27.4 mmol, 6.0 eq) and DMAP (306 mg, 2.5 mmol, 0.55 eq) were added successively and it was cooled at 0°C. To this temperature a solution of **compound 10** (1.17 g, 4.56 mmol, 1.0 eq) in 60 mL dry CH_2Cl_2 was added followed by dropwise addition of MsCl (1 mL, 13.7 mmol, 3.0 eq). The reaction mixture was stirred at rt for 1.5h before it was quenched with saturated aqueous NH_4Cl. After separation, the aqueous layer was extracted three times with CH_2Cl_2. The combined organic extracts were washed one time with saturated aqueous NH_4Cl, dried over Na_2SO_4, filtered and concentrated under reduced pressure to provide the diastereomers of the desired mesylates (HRMS (ESI, m/z): calcd: $C_{14}H_{23}O_7S^+$ ($[M+H]^+$): 335.1159, found: 335.1155. ^{1}H NMR (500 MHz, $CDCl_3$) δ = 4.98 – 4.90 (m, 1H), 4.85 (ddd, *J* = 10.1, 8.1, 6.5 Hz, 1H), 3.72 – 3.67 (m, 1H), 3.62 – 3.56 (m, 1H), 3.32 (s, 3H), 3.16 – 3.10 (m, 1H), 3.00 (s, 3H), 2.78 (dd, *J* = 15.0, 7.2 Hz, 1H), 2.61 – 2.54 (m, 1H), 2.48 – 2.44 (m, 1H), 2.40 (dd, *J* = 15.3, 5.3 Hz, 1H), 1.98 (dd, *J* = 15.3, 7.2 Hz, 1H), 1.44 (d, *J* = 6.4 Hz, 3H), 1.15 (s, 3H). ^{13}C NMR (125 MHz, $CDCl_3$) δ = 210.0, 175.8, 81.1, 74.9, 70.8, 59.2, 49.9, 47.5, 42.00, 40.9, 39.0, 37.5, 36.9, 35.8, 33.1, 20.2, 16.4.) The residue (1.21 g, 3.62 mmol, 1.0 eq) was dissolved in 16 mL dry DMF and Li_2CO_3 (1.6 g, 21,72 mmol, 6.0 eq), LiBr (1.57 g, 18.1 mmol, 5.0 eq) were added in one potion. The reaction mixture was refluxed under Ar at 140°C for 4h. Finally, the reaction was allowed to cool to room temperature before it was quenched with aqueous HCl 1N and then extracted four times with EtOAc. The combined organic extracts were washed two times with H_2O, dried over Na_2SO_4 and concentrated under reduced pressure. The residue was chromatographed (silica gel) with gradient from 2:1 hexane: Et_2O to 1:2 hexane: Et_2O [R_f = 0.65 (hexane:EtOAc = 1:3, UV active on TLC, stains greenish upon *p*-anisaldehyde staining)] to afford pure

Compound 14 as a white amorphous solid (367 mg, 34% over 2 steps). $[\alpha]^{20}_D$ = +73.7 (*c* 0.7, $CHCl_3$). HRMS (ESI, m/z): calcd. for $C_{13}H_{19}O_4^+$ ($[M+H]^+$): 239.1278, found: 239.1281. ^1H NMR (500 MHz, $CDCl_3$) δ = 5.82 (dd, *J* = 17.6, 10.7 Hz, 1H), 5.19 (d, *J* = 10.7 Hz, 1H), 5.09 (d, *J* = 17.6 Hz, 1H), 4.91 (ddd, *J* = 9.7, 7.9, 6.4 Hz, 1H), 3.71 (dd, *J* = 9.3, 4.6 Hz, 1H), 3.61 (dd, *J* = 9.3, 3.4 Hz, 1H), 3.35 (s, 3H), 2.99 – 2.89 (m, 1H), 2.64 (dd, *J* = 15.9, 6.2 Hz, 1H), 2.58 – 2.46 (m, 3H), 1.92 (dd, *J* = 14.3, 9.9 Hz, 1H), 1.20 (s, 3H). ^{13}C NMR (125 MHz, $CDCl_3$) δ = 210.5, 176.1, 139.6, 115.6, 75.0, 70.9, 59.3, 49.7, 47.4, 40.3, 38.3, 36.2, 24.9.

Having in our hands derivative **14**, we next turned our attention to the final introduction of α-propenyl-alkyl chain. The highly congested carbocyclic system of **14** was found surprisingly difficult to be alkylated. Several attempts by utilizing various nucleophiles resulted exclusive alkylation to the lactone moiety producing stable lactols **SI-19**–**SI-20** and dehydrated derivative **SI-21** (Table S10). Finally, incorporation of cerium chloride in the reaction mixture resulted clean introduction of isopropenyl-chain to complete the synthesis of common synthetic scaffold **19**.

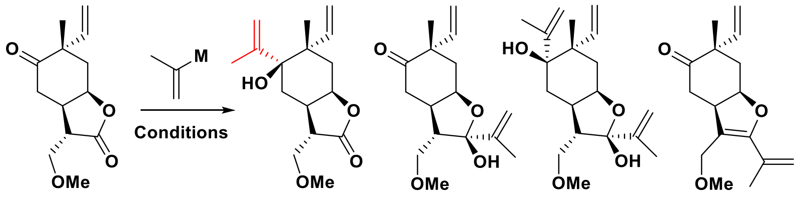

14 3 SI-19 SI-20 SI-21

Table S10. Attempts to introduce the final chain.

Entry	Conditions	Conversion	Products and yields
1	2-bromopropene (2.5 equiv), t-BuLi (5 equiv), THF, -78 °C, 3 h	75%	**3** (5%), **SI-19** (29%), **SI-20** (12%)
2	2-bromopropene (4 equiv), t-BuLi (8 equiv), Et_2O, -78 °C, 3 h	80%	**3** (9%), **SI-19** (30%), **SI-20** (15%), **SI-21** (8%)
3	Isopropenyl magnesium bromide 0.5M in THF (1.5 equiv), THF, 0 °C to rt, 8 h	0%	-
4	Isopropenyl magnesium bromide 0.84M in THF (1 equiv), $CeCl_3$ (1 equiv), THF, -78 °C, 3 h	80%	**3** (75%)

(3*S*,3a*R*,5*S*,6*S*,7a*R*)-5-hydroxy-3-(methoxymethyl)-6-methyl-5-(prop-1-en-2-yl)-6-vinylhexahydrobenzofuran-2(3H)-one (3)

Cerium chloride ($CeCl_3$) (1.55 g, 6.3 mmol, 1.0 eq) was quickly added to a Schlenk apparatus which was immersed in an oil bath and heated gradually to 135-140 °C with evacuation (ca. 0.1 Torr). The cerium chloride was completely dried in vacuo by stirring at the same temperature for 2 h. While the Schlenlk apparatus was still hot, Ar gas was introduced and the flask was then cooled in an ice bath. THF (20 mL, freshly distilled from sodium benzophenone) was added all at once with vigorous stirring. The ice bath was then removed, and the suspension was well stirred overnight under Ar at room temperature. The flask was immersed in a dry ice/acetone cooling bath and the isopropenyl Grignard reagent (7.6 mL, 0.84 M in THF, 6.3 mmol, 1.0 eq) was added dropwise. After stirring for 1.5 h at -78°C, **Compound 14** (1.5 g, 6.3 mmol, in 50 mL THF, 1.0 eq) was added dropwise and the stirring was continued for 1.5 h. The reaction mixture was quenched saturated aqueous NH_4Cl (50 mL). The organic layer was separated, and the aqueous layer was extracted

with EtOAc (4×40 mL). The organic layers were combined and dried over Na_2SO_4. The dried solution was filtered, and concentrated *in vacuo*. The residue was chromatographed (silica gel) with gradient from 6:1 hexane: EtOAc to 3:1 hexane: EtOAc [R_f = 0.52 (hexane:EtOAc = 1:1, UV active on TLC, stains purple upon *p*-anisaldehyde staining)]mto afford pure **Compound 3** as a white amorphous solid (1.32 g, 75%). $[\alpha]^{20}_D$ = +17.3 (*c* 1.5, $CHCl_3$). HRMS (ESI, m/z): calcd. for $C_{16}H_{25}O_4^+$ ($[M+H]^+$):281.1747, found:281.1748. ^1H NMR (500 MHz, $CDCl_3$) δ = 6.05 (dd, *J* = 17.4, 11.0 Hz, 1H), 5.12 (dd, *J* = 12.8, 1.1 Hz, 1H), 5.09 (dd, *J* = 6.3, 1.1 Hz, 1H), 5.02 (s, 1H), 4.99 (s, 1H), 4.77 (ddd, *J* = 11.6, 8.1, 6.4 Hz, 1H), 3.75 – 3.64 (m, 1H), 3.65 – 3.56 (m, 2H), 3.34 (s, 3H), 2.83 – 2.72 (m, 1H), 2.31 (dd, *J* = 15.4, 6.7 Hz, 1H), 2.05 (dd, *J* = 13.2, 11.5 Hz, 1H), 1.89 (dd, *J* = 15.6, 1.5 Hz, 1H), 1.82 (dd, *J* = 13.2, 6.3 Hz, 1H), 1.77 (d, *J* = 1.4 Hz, 3H), 1.71 (s, 1H), 1.04 (s, 3H). ^{13}C NMR (125 MHz, $CDCl_3$) δ = 177.5, 149.1, 141.8, 114.5, 114.0, 78.2, 75.3, 70.3, 59.3, 45.2, 43.8, 38.5, 36.6, 32.0, 22.3, 20.9.

(2*S*,3*S*,3a*R*,6*S*,7a*R*)-2-hydroxy-3-(methoxymethyl)-6-methyl-2-(prop-1-en-2-yl)-6- vinylhexahydrobenzofuran-5(4H)-one (SI-19)

To a stirred solution of 2-bromopropene (75 µl, 0,84 mmol, 4 eq) in dry Et_2O (1 mL) at -78 °C, *t*BuLi (0.86 ml, 1.67 mmol, 8 eq) was added slowly under Ar atmosphere. The mixture was stirred for 15 min at the same temperature, before a solution of **Compound 14** (50 mg, 0.21 mmol, 1 eq) in dry Et_2O (4 ml) was added dropwise. Upon consumption of the starting material after 1 h, the mixture was quenched by the addition of the addition of saturated NH_4Cl (3 ml). The organic layer was separated and the aqueous layer was extracted with EtOAc (4×10 mL). The organic layers were combined and dried over Na_2SO_4. The dried solution was filtered, and concentrated *in vacuo*. The residue was chromatographed (silica gel) with gradient from 20:1 hexane: EtOAc to 2:1 hexane: EtOAc [R_f = 0.53 (hexane:EtOAc = 1:1, UV active on TLC, stains purple upon *p*-anisaldehyde staining)] to afford pure **Compound SI-19** as a white amorphous solid (18 mg, 30%). $[\alpha]^{20}_D$ = +14.6 (*c* 0.8, $CHCl_3$). HRMS (ESI, m/z): calcd. for $C_{16}H_{24}O_4Na^+$ ($[M+Na]^+$): 303.1572, found: 303.1570. ^1H NMR (500 MHz, $CDCl_3$) δ = 5.81 (dd, *J* = 17.6, 10.7 Hz, 1H), 5.29 (s, 1H), 5.18 – 5.01 (m, 3H), 4.57 (td, *J* = 8.8, 5.4 Hz, 1H), 3.63 – 3.46 (m, 2H), 3.33 (s, 3H), 2.77 – 2.68 (m, 1H), 2.64 (dd, *J* = 14.7, 6.3 Hz, 1H), 2.33 (ddd, *J* = 14.7, 8.0, 2.5 Hz, 2H), 2.04 – 1.94 (m, 2H), 1.81 (s, 3H), 1.22 (s, 3H). ^{13}C NMR (125 MHz, $CDCl_3$) δ = 213.7, 145.3, 140.8, 114.5, 113.8, 105.8, 73.1, 71.0, 59.3, 52.9, 49.2, 42.2, 38.7, 38.2, 25.6, 18.5.

(2*S*,3*S*,3a*R*,5*S*,6*S*,7a*R*)-3-(methoxymethyl)-6-methyl-2,5-di(prop-1-en-2-yl)-6-vinyloctahydrobenzofuran-2,5-diol (SI-20)

To a stirred solution of 2-bromopropene (75 µl, 0,84 mmol, 4 eq) in dry Et_2O (1 mL) at -78 °C, *t*BuLi (0.86 ml, 1.67 mmol, 8 eq) was added slowly under Ar atmosphere. The mixture was stirred for 15 min at the same temperature, before a solution of **Compound 14** (50 mg, 0.21 mmol, 1 eq) in dry Et_2O (4 ml) was added dropwise. Upon consumption of the starting material after 1 h, the mixture was quenched by the addition of the addition of saturated NH_4Cl (3 ml). The organic layer was separated and the aqueous layer was extracted with EtOAc (4×10 mL). The organic layers were combined and dried over Na_2SO_4. The dried solution was filtered, and concentrated *in vacuo*. The residue was chromatographed (silica gel) with gradient from 20:1 hexane: EtOAc to 2:1 hexane: EtOAc [R_f = 0.57 (hexane:EtOAc = 1:1, UV active on TLC, stains purple upon *p*-anisaldehyde staining)] to afford pure **Compound SI-20** as a white

amorphous solid (18 mg, 30%). $[\alpha]^{20}_{D}$ = +19.2 (*c* 0.6, $CHCl_3$). HRMS (ESI, m/z): calcd. for $C_{19}H_{30}O_4Na^+$ ($[M+Na]^+$): 345.2042, found: 345.2044. ^{1}H NMR (500 MHz, $CDCl_3$) δ = 5.72 (dd, *J* = 17.4, 10.8 Hz, 1H), 5.19 (dd, *J* = 2.2, 1.1 Hz, 1H), 4.99 – 4.84 (m, 4H), 4.79 (d, *J* = 1.8 Hz, 1H), 4.56 (t, *J* = 5.2 Hz, 1H), 3.29 (s, 3H), 3.21 (dd, *J* = 9.8, 4.6 Hz, 1H), 3.00 (t, *J* = 9.7 Hz, 1H), 2.74 (t, *J* = 5.9 Hz, 1H), 2.29 – 2.01 (m, 2H), 1.87 – 1.76 (m, 4H), 1.77 – 1.67 (m, 5H), 1.33 (s, 3H). ^{13}C NMR (125 MHz, $CDCl_3$) δ = 148.1, 146.3, 141.3, 113.0, 112.4, 110.5, 106.9, 82.4, 75.7, 71.6, 59.1, 52.8, 45.5, 38.5, 36.0, 34.7, 24.4, 21.4, 20.0.

(3a*R*,6*S*,7a*R*)-3-(methoxymethyl)-6-methyl-2,6-divinyl-3a,6,7,7a-tetrahydrobenzofuran-5(4H)-one (SI-21)

To a stirred solution of 2-bromopropene (75 µl, 0,84 mmol, 4 eq) in dry Et_2O (1 mL) at -78 °C, *t*BuLi (0.86 ml, 1.67 mmol, 8 eq) was added slowly under Ar atmosphere. The mixture was stirred for 15 min at the same temperature, before a solution of **Compound 14** (50 mg, 0.21 mmol, 1 eq) in dry Et_2O (4 ml) was added dropwise. Upon consumption of the starting material after 1 h, the mixture was quenched by the addition of the addition of saturated NH_4Cl (3 ml). The organic layer was separated, and the aqueous layer was extracted with EtOAc (4×10 mL). The organic layers were combined and dried over Na_2SO_4. The dried solution was filtered and concentrated *in vacuo*. The residue was chromatographed (silica gel) with gradient from 20:1 hexane: EtOAc to 2:1 hexane: EtOAc [R_f = 0.55 (hexane:EtOAc = 1:1, UV active on TLC, stains purple upon *p*-anisaldehyde staining)] to afford pure **Compound SI-21** as a white amorphous solid (18 mg, 30%). $[\alpha]^{20}_{D}$ = +12.7 (*c* 0.9, $CHCl_3$). HRMS (ESI, m/z): calcd. for $C_{16}H_{23}O_3^+$ ($[M+H]^+$): 263.1647, found: 263.1644. ^{1}H NMR (500 MHz, $CDCl_3$) δ = 5.80 (dd, *J* = 17.5, 10.7 Hz, 1H), 5.21 (dd, *J* = 2.1, 1.1 Hz, 1H), 5.14 – 5.03 (m, 3H), 4.84 (ddd, *J* = 10.3, 8.3, 5.5 Hz, 1H), 4.06 (d, *J* = 11.5 Hz, 1H), 3.95 (d, *J* = 11.6 Hz, 1H), 3.52 – 3.39 (m, 2H), 3.29 (s, 3H), 2.63 (dd, *J* = 14.9, 6.1 Hz, 1H), 2.47 – 2.38 (m, 1H), 2.19 – 2.10 (m, 1H), 1.91 (t, *J* = 1.2 Hz, 3H), 1.22 (s, 3H). ^{13}C NMR (125 MHz, $CDCl_3$) δ = 212.9, 154.9, 140.9, 134.5, 118.0, 114.3, 109.7, 76.5, 66.5, 57.9, 49.78, 43.0, 40.2, 38.3, 25.3, 20.7

6.4 Synthesis of sulfur-ylides for accessing trans-6-hydroxy derivatives.

Incorporation of a hydroxyl- or alkoxy-carbonyl functionality at 6-position of the common synthetic scaffold **14** will allow the expansion of our divergent plan to biologically important *trans*-6,12-*α*-methylene-*γ*-butyrolactones. Unfortunately, basic conditions which are commonly used for introduction of such groups *α* to a carbonyl moiety failed to deliver the desired compounds, due to the readily deprotection of methyl ether group of the lactone core. Introduction by acidic media precludes the participation of an enol form which is not stabilized in the case of **14**. During our extensive experimentation for the dehydration of compound **10** we discovered the formation of a stabilized enol when Martin sulfurane was utilized as dehydrating reagent. This stabilized enol was spontaneously transformed to sulfur ylide **12** which is stable and can be easily purified. When **12** was treated with acidic conditions as PTSA, substituted compound **13** through a stereoselective protonation-S_N2 substitution sequence.

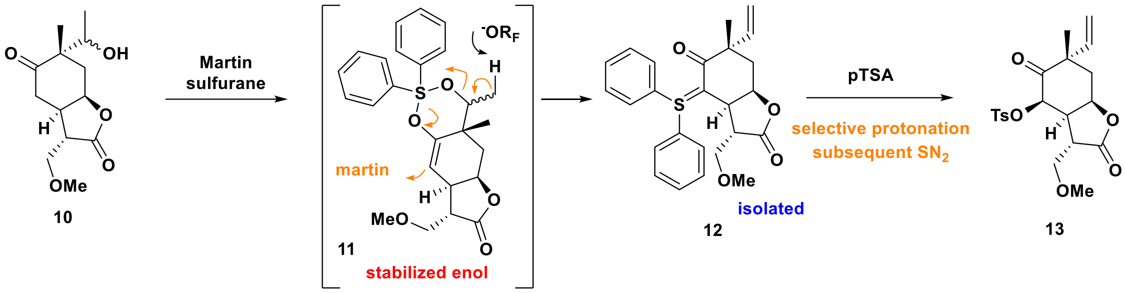

Scheme S11. Key observation and solution for introduction of oxygen functionality in 6-position.

(3*S*,3a*R*,6*S*,7a*R*)-4-(diphenyl-l4-sulfanylidene)-3-(methoxymethyl)-6-methyl-6-vinyltetrahydrobenzofuran-2,5(3H,4H)-dione (12)

To a stirring solution of **Compound 10** (400 mg, 1.57 mmol, 1 eq) in benzene (30 mL), Martin sulfurane was added (2.2 g, 3.27 mmol, 2 eq) and the reaction mixture was left stirring at room temperature for 30 min before it was evaporated to dryness. The residue was chromatographed (silica gel) with gradient from 10:1 hexane: EtOAc to 1:3 hexane: EtOAc [R_f = 0.4 (EtOAc, UV active on TLC, stains upon Seebach staining)] to afford pure **Compound 12** as a white amorphous solid (365 mg, 55%). $[\alpha]^{20}_{D}$ = +23.1 (*c* 4.1, $CHCl_3$). HRMS (ESI, m/z): calcd. for $C_{25}H_{26}O_4SNa^+$ ($[M+Na]^+$): 445.1449, found: 445.1445. ^1H NMR (500 MHz, $CDCl_3$) δ = 7.73 – 7.64 (m, 2H), 7.62 – 7.52 (m, 2H), 7.49 (qd, *J* = 7.0, 3.8 Hz, 3H), 7.44 – 7.37 (m, 3H), 5.71 (dd, *J* = 17.4, 10.6 Hz, 1H), 4.90 (td, *J* = 8.0, 4.1 Hz, 1H), 4.85 (d, *J* = 10.7 Hz, 1H), 4.63 (d, *J* = 17.5 Hz, 1H), 3.73 – 3.63 (m, 2H), 3.63 – 3.52 (m, 1H), 3.18 (s, 3H), 2.67 (dt, *J* = 10.1, 3.9 Hz, 1H), 2.23 (dd, *J* = 13.3, 4.6 Hz, 1H), 1.95 (t, *J* = 12.6 Hz, 1H), 1.19 (s, 3H). ^{13}C NMR (125 MHz, $CDCl_3$) δ = 185.1, 176.4, 143.5, 131.5, 131.2, 131.0, 130.9, 130.0, 129.6, 129.5, 129.4, 129.2, 129.1, 129.0, 113.2, 74.8, 69.7, 64.0, 59.0, 49.9, 46.2, 42.7, 38.7, 26.2.

(3*S*,3a*R*,4*S*,6*S*,7a*R*)-3-(methoxymethyl)-6-methyl-2,5-dioxo-6-vinyloctahydrobenzofuran-4-yl 4-methylbenzene sulfonate (13)

Compound 12 (162 mg, 0.369 mmol, 1 eq) was dissolved in toluene (10 mL) before *p*-TsOH·H_2O (64 mg, 0.369 mmol, 1 eq) was added and the resulting mixture was refluxed at 80 °C for 3h. Then, evaporation to dryness followed and the crude mixture was chromatographed (silica gel) with gradient from 10:1 hexane: EtOAc to 1:1 hexane: EtOAc [R_f = 0.57 (hexane:EtOAc = 1:1, UV active on TLC, stains upon Seebach staining)]nto afford pure **Compound 13** as a white amorphous solid (67 mg, 60%). $[\alpha]^{20}_{D}$ = -31.4 (*c* 2.7, $CHCl_3$). HRMS (ESI, m/z): calcd. for $C_{20}H_{24}O_7SNa^+$ ($[M+Na]^+$): 431.1140, found: 431.1137. ^1H NMR (500 MHz, $CDCl_3$) δ = 7.80 (d, *J* = 7.9 Hz, 2H), 7.34 (d, *J* = 7.9 Hz, 2H), 5.67 (dd, *J* = 17.5, 10.6 Hz, 1H), 5.41 (d, *J* = 7.8 Hz, 1H), 5.11 (d, *J* = 10.6 Hz, 1H), 4.98 – 4.88 (m, 2H), 3.79 (dd, *J* = 9.2, 2.4 Hz, 1H), 3.61 – 3.48 (m, 2H), 3.31 (s, 3H), 2.75 – 2.66 (m, 1H), 2.49 – 2.40 (m, 4H), 2.12 (dd, *J* = 15.2, 5.9 Hz, 1H), 1.21 (s, 3H). ^{13}C NMR (125 MHz, $CDCl_3$) δ = 203.0, 175.1, 145.5, 139.9, 132.8, 129.9, 128.0, 115.7, 77.1, 74.4, 71.0, 59.2, 50.0, 43.2, 42.5, 37.8, 24.5, 21.7.

Extended heating during the protonation and nucleophilic substitution of ylide **12** (and by using <1 eq of p-TSA/AcOH) can lead to phenyl migration resulting Compound **SI-22** (Scheme 12). For similar observations please see reference 15.

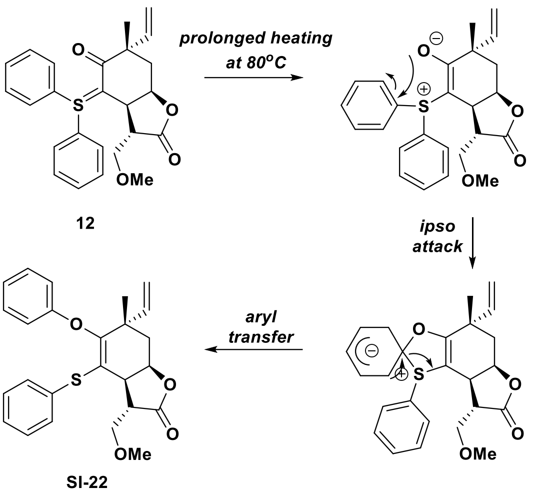

Scheme S12. Mechanistic considerations for Compound **SI-22**.

(3*S*,3a*R*,6*S*,7a*R*)-3-(methoxymethyl)-6-methyl-5-phenoxy-4-(phenylthio)-6-vinyl-3a,6,7,7a-tetrahydrobenzofuran-2(3H)-one (SI-22)

Compound 12 (162 mg, 0.369 mmol, 1 eq) was dissolved in toluene (10 mL) before *p*-TsOH·H_2O (29 mg, 0.169 mmol, 0.46 eq) was added and the resulting mixture was refluxed at 80 °C for 3h. Then, evaporation to dryness followed and the crude mixture was chromatographed (silica gel) with gradient from 10:1 hexane: EtOAc to 6:1 hexane: EtOAc [R_f = 0.57 (hexane:EtOAc = 1:1, UV active on TLC, stains upon Seebach staining)] to afford pure **Compound SI-22** as a white amorphous solid (20 mg, 13%). $[\alpha]^{20}_{D}$=+22.8 (*c* 2.3, $CHCl_3$). HRMS (ESI, m/z): calcd. for $C_{25}H_{26}O_4SNa^+$ ($[M+Na]^+$): 445.1449, found: 445.1450 ^{1}H NMR (500 MHz, $CDCl_3$) δ = 7.31 – 7.17 (m, 7H), 6.98 (tt, *J* = 7.4, 1.3 Hz, 1H), 6.87 – 6.79 (m, 2H), 6.01 – 5.87 (m, 1H), 5.25 (d, *J* = 17.3 Hz, 1H), 5.21 (d, *J* = 10.6 Hz, 1H), 4.81 (ddd, *J* = 9.8, 7.7, 4.5 Hz, 1H), 3.81 (dd, *J* = 9.0, 2.4 Hz, 1H), 3.61 (dd, *J* = 9.1, 3.0 Hz, 1H), 3.54 (dd, *J* = 9.3, 6.8 Hz, 1H), 3.05 (d, *J* = 2.5 Hz, 3H), 2.85 (dt, *J* = 8.3, 2.8 Hz, 1H), 2.31 – 2.21 (m, 1H), 1.97 – 1.89 (m, 1H), 1.26 (s, 3H). ^{13}C NMR (126 MHz, $CDCl_3$) δ = 176.3, 158.2, 156.5, 142.6, 133.6, 130.0, 129.6, 129.1, 126.8, 121.7, 119.4, 115.0, 114.2, 74.4, 70.3, 58.8, 48.3, 43.7, 39.7, 38.7, 25.4.

Some minor isolated products from Martin sulfurane reaction are presented below (Scheme 13). The mechanism by which these intermediates are formed is still under investigation.

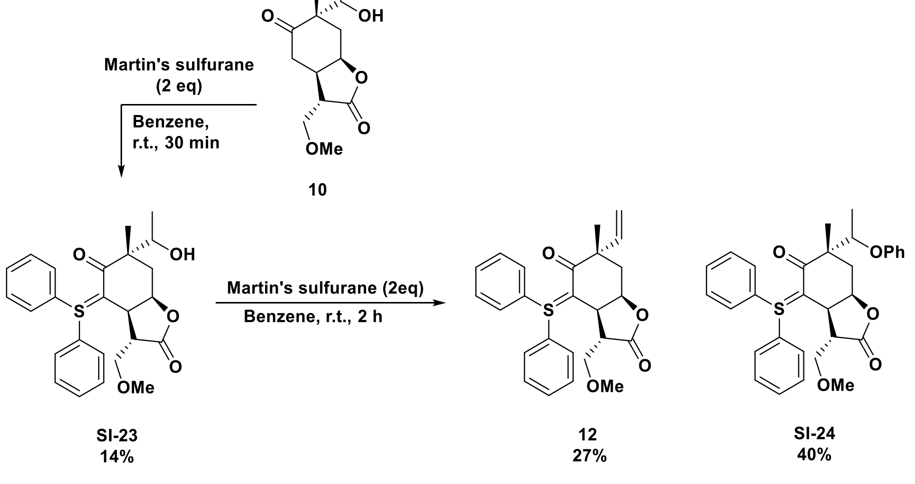

Scheme S13. Minor side products resulting from the effect of Martin sulfurane on Compound **10**.

(3*RS*,3a*SR*,6*RS*,7a*SR*)-4-(diphenyl-l4-sulfaneylidene)-6-((S)-1-hydroxyethyl)-3-(methoxymethyl)-6-methyltetrahydrobenzofuran-2,5(3H,4H)-dione (SI-23)

To a stirring solution of **Compound 10** (400 mg, 1.573 mmol, 1 eq) in benzene (30 mL), Martin sulfurane was added (2.2 g, 3.271 mmol, 2 eq) and the reaction mixture was left stirring at room temperature for 30 min before it was evaporated to dryness. The residue was chromatographed (silica gel) with gradient from 10:1 hexane: EtOAc to 1:3 hexane: EtOAc [R_f = 0.2 (EtOAc, UV active on TLC, stains upon Seebach staining)] to afford pure **Compound SI-23** as a white amorphous solid (83 mg, 14%). $[\alpha]^{20}_{D}$ = +33.8 (*c* 1.5, $CHCl_3$). HRMS (ESI, m/z): calcd. for $C_{25}H_{28}O_5SNa^+$ ($[M+Na]^+$): 463.1555, found: 463.1551. ^{1}H NMR (500 MHz, $CDCl_3$) δ = 7.51 (m, 10H), 4.94 (td, *J* = 7.3, 3.8 Hz, 1H), 3.78 (q, *J* = 6.4 Hz, 1H), 3.50 (dd, *J* = 9.5, 4.7 Hz, 1H), 3.40 (t, *J* = 6.3 Hz, 1H), 3.15 (s, 3H), 3.20 (s, 1H), 2.48 (q, *J* = 4.5 Hz, 1H), 2.04 (dd, *J* = 14.3, 7.0 Hz, 1H), 1.86 (dd, *J* = 14.6, 3.9 Hz, 1H), 1.22 (s, 3H), 1.06 (d, *J* = 6.3 Hz, 3H). ^{13}C NMR (125 MHz, $CDCl_3$) δ = 188.9, 176.5, 131.7, 131.5, 130.1, 129.8, 129.4, 128.9, 128.8, 76.3, 72.1, 70.8, 66.6, 59.1, 44.1, 42.7, 36.0, 18.0, 16.1.

(3*S*,3a*R*,6*S*,7a*R*)-4-(diphenyl-l4-sulfaneylidene)-3-(methoxymethyl)-6-methyl-6-((R)-1-phenoxyethyl)tetrahydrobenzofuran-2,5(3H,4H)-dione (SI-24)

To a stirring solution of **Compound SI-23** (68 mg, 0.154 mmol, 1 eq) in benzene (8 mL), Martin sulfurane was added (207 mg, 0.308 mmol, 2 eq) and the reaction mixture was left stirring at room temperature for 1 h before it was evaporated to dryness. The residue was chromatographed (silica gel) with gradient from 5:1 hexane: EtOAc to 1:1 hexane: EtOAc [R_f = 0.5 (hexane:EtOAc = 1:3, UV active on TLC, stains upon Seebach staining)] to afford pure **Compound SI-24** as a white amorphous solid (32 mg, 40%). $[\alpha]^{20}_{D}$ = +28.9 (*c* 1.9, $CHCl_3$). HRMS (ESI, m/z): calcd. for $C_{31}H_{32}O_5SNa^+$ ($[M+Na]^+$): 539.1868, found: 539.1870. ^{1}H NMR (500 MHz, $CDCl_3$) δ = 7.80 – 7.21 (m, 15H), 5.19 (td, *J* = 7.2, 4.5 Hz, 1H), 4.32 (d, *J* = 6.5 Hz, 1H), 3.67 – 3.53 (m, 2H), 3.43 – 3.36 (m, 1H), 3.21 (s, 3H), 2.57 (td, *J* = 5.5, 3.5 Hz, 1H), 2.42 (dd, *J* = 15.0, 4.4 Hz, 1H), 2.23 – 2.15 (m, 1H), 1.40 (s, 3H), 0.61 (d, *J* = 6.2 Hz, 3H). ^{13}C NMR (125 MHz, $CDCl_3$) δ = 184.8, 176.8, 131.2, 131.2, 131.0, 130.9, 129.8, 129.8, 129.2, 129.0, 128.9, 128.1, 127.9, 82.4, 77.0, 70.9, 65.9, 59.1, 51.8, 47.3, 43.3, 34.0, 26.3, 16.9.

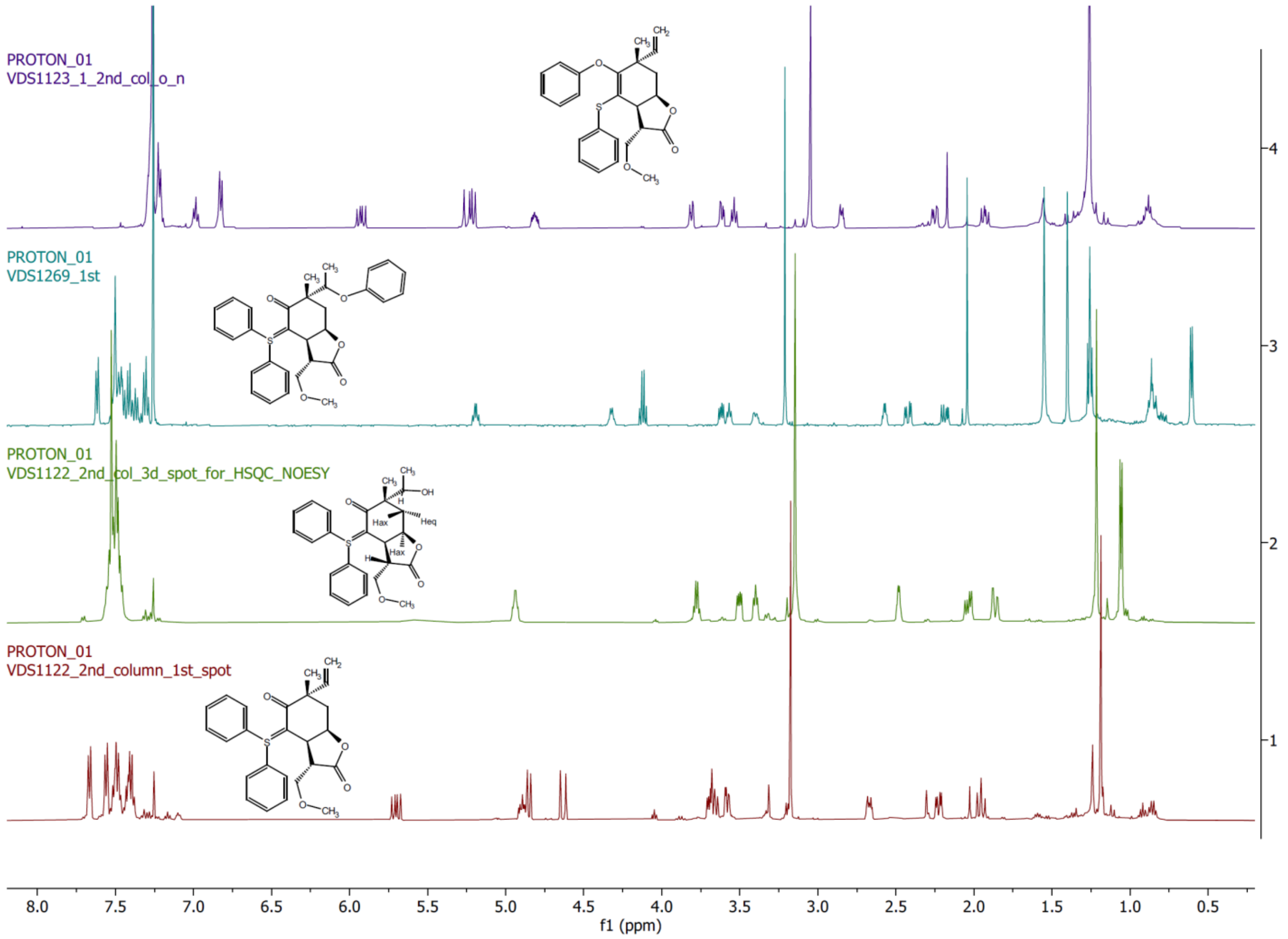

Spectra 12. Diversity of sulfur compounds obtained by Martin sulfurane depending on the conditions.

6.5 Utilization of common scaffold for the synthesis of sesquiterpenoid lactones

6.5.1 Synthesis of germacranolides

With common scaffold in hands, we tested its ability to produce diverse carbocycles. Despite the *syn*-orientation of the alkyl chains of elemane core **3**, molecular models showed the preferred chair orientation for initiation of an oxy-Cope expansion to the ten-member macrocycle of germacranes. Reaction under thermal conditions (170 °C) provided compound **16** in 53% yield as a mixture of two distinct conformers which are not interconvert to each other even at high temperatures, corroborating earlier reports on similar compounds.

6.5.2 Synthesis of guaianolide lactones

Heating compound **3** at higher temperature (170 °C) led to a highly diastereoselective ene-reaction to build 5,7-guaiane skeleton and afording compound **2** in 72% yield. On the contrary to the thermal, anionic oxy-Cope using KHMDS failed to produce germacranolide **16**, but instead provided translactonized product **18** that resembles irregular natural elemanolides. Interestingly, employment of a mild base in **3** (Et_3N, K_2CO_3), even at low temperatures, resulted the rapid deprotection of methoxy group to **17** by a hydroxyl-assisted deprotonation of C11.

6.5.3 Mechanistic considerations-conformational analysis

The high selectivity observed in guaianolide **27** is attributed to **3A**, as the only sterically viable conformer to initiate the oxy-Cope/ene reaction. The sole isolation of α-methyl germacranolide **16** points towards either the intramolecular

inner ring protonation of the initially formed enol **15A**, or the enol flip to **15B** followed by its intermolecular outer ring protonation. As shown by the 3D model in Scheme S14, the short distance (2.9 Å) between the methyl group and the enol moiety fortifies the intramolecular theory. The ene reaction that follows produces the expected **2** as the sole isomer, bearing the naturally unfavorable β-H at the C1 position.

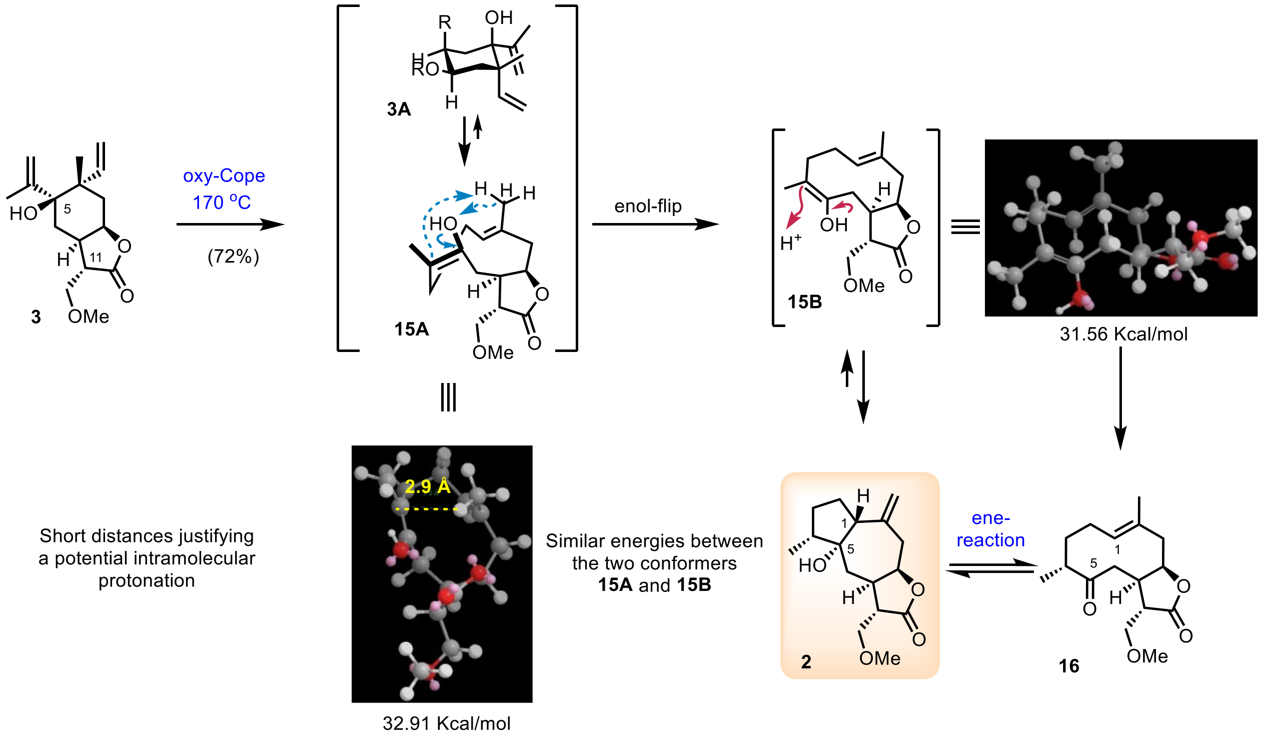

Scheme S14. Mechanistic explanation of the oxy/cope-ene products.

(3a*R*,5*S*,6*S*,7a*R*)-5-hydroxy-6-methyl-3-methylene-5-(prop-1-en-2-yl)-6-vinylhexahydrobenzofuran-2(3H)-one (17)

To a solution of **Compound 3** (18 mg, 0.064 mmol, 1.0 eq) in THF (1.5 mL) a solution of KHMDS was added (0.25 mL, 0.125 mmol, 0.5M in toluene, 6 eq) at r.t. and the resulting mixture was left stirring at the same temperature for 1 h. The reaction was quenched with 10 % HCl (0.25 mL), the two phases were separated, and the aqueous layer was extracted with EtOAc (3×4 mL). The combined organic extracts were dried over Na_2SO_4, filtered, and evaporated under reduced pressure. The residue was chromatographed (silica gel) with gradient from 8:1 hexane/EtOAc to 5:1 hexane:EtOAc [R_f = 0.64 (hexane/EtOAc = 1:1, UV active on TLC, stains purple upon *p*-anisaldehyde staining)] to afford pure Compound **17** as a white amorphous solid (13 mg, 82%). $[\alpha]^{20}_D$ = +19.1 (*c* 0.8, $CHCl_3$). HRMS (ESI, m/z): calcd for $C_{15}H_{21}O_3^+$ ($[M+H]^+$): 249.1491, found: 249.1494. ^{1}H NMR (500 MHz, $CDCl_3$) δ = 6.32 (d, *J* = 4.3 Hz, 1H), 6.06 (dd, *J* = 17.4, 11.0 Hz, 1H), 5.60 (d, *J* = 3.4 Hz, 1H), 5.16 – 4.99 (m, 4H), 4.92 (dd, *J* = 17.3, 8.4 Hz, 1H), 3.31 (m, 1H), 2.55 (dd, *J* = 15.5, 7.4 Hz, 1H), 2.06, (s, 1H), 1.95 (ddd, *J* = 20.4, 13.5, 8.6 Hz, 3H), 1.82 (s, 3H), 1.02 (s, 3H). ^{13}C NMR (125 MHz, $CDCl_3$) δ = 170.2, 148.2, 141.5, 139.2, 120.1, 114.3, 114.0, 77.2, 75.0, 45.2, 38.9, 37.1, 33.3, 22.0, 21.1.

(5*R*,6*R*,8*S*)-6-hydroxy-8-methyl-4-methylene-1-(prop-1-en-2-yl)-8-vinyl-2-oxabicyclo[3.3.1]nonan-3-one (18)

Compound 3 (18 mg, 0.064 mmol, 1.0 eq) was dissolved in a sealed tube in THF (1.5 mL) and a solution of KHMDS was added (0.25 mL, 0.125 mmol, 0.5M in toluene, 6 eq). The tube was then sealed, and the reaction mixture was heated

to 60 °C for 1 h. The reaction was quenched with 10 % HCl (0.25 mL), the two phases were separated, and the aqueous layer was extracted with EtOAc (3×4 mL). The combined organic extracts were dried over Na_2SO_4, filtered, and evaporated under reduced pressure. The residue was chromatographed (silica gel) with gradient from 2:1 hexane:EtOAc to EtOAc [R_f = 0.32 (hexane:EtOAc = 1:1, UV active on TLC, stains blue upon *p*-anisaldehyde staining)] to afford pure **Compound 18** as a white amorphous solid (10 mg, 63%). $[\alpha]^{20}_D$ = -23.1 (*c* 0.5, $CHCl_3$). HRMS (ESI, m/z): calcd for $C_{15}H_{21}O_3^+$ ($[M+H]^+$): 249.1491, found: 249.1490. ^1H NMR (500 MHz, $CDCl_3$) δ = 6.64 (d, *J* = 1.6 Hz, 1H), 6.05 (dd, *J* = 17.5, 11.0 Hz, 1H), 5.65 (d, *J* = 1.0 Hz, 1H), 5.22 – 5.11 (m, 3H), 5.02 (s, 1H), 4.14 – 4.07 (m, 1H), 2.96 (d, *J* = 3.5 Hz, 1H), 2.42 (dd, *J* = 14.4, 3.0 Hz, 1H), 2.00 (dt, *J* = 16.9, 5.0 Hz, 2H), 1.91 (dd, *J* = 14.1, 3.8 Hz, 1H), 1.87 (s, 3H), 1.32 (m, 1H), 1.11 (s, 3H). ^{13}C NMR (125 MHz, $CDCl_3$) δ = 164.5, 144.2, 141.1, 132.8, 131.2, 115.6, 114.8, 87.7, 66.8, 47.2, 41.9, 40.3, 32.1, 22.8, 22.0.

(3*S*,3a*R*,6*R*,11a*R*,E)-3-(methoxymethyl)-6,10-dimethyl-3a,6,7,8,11,11a-hexahydrocyclodeca[b]furan-2,5(3H,4H)-dione (16)

Compound 3 (25 mg, 0.09 mmol) was dissolved in toluene (1 mL) in a sealed tube and it was left stirring at 170°C for 12 h before it was evaporated to dryness. The residue was chromatographed (silica gel) with gradient from 10:1 benzene: Et_2O to 8:1 benzene:Et_2O [R_f = 0.38 (benzene:Et_2O = 4:1, UV active on TLC, stains greenish upon *p*-anisaldehyde staining)] to afford pure **Compound 16** as a white amorphous solid (13 mg, 53%). $[\alpha]^{20}_D$ = +29.5 (*c* 0.3, $CHCl_3$). HRMS (ESI, m/z): calcd. for $C_{16}H_{25}O_4^+$ ($[M+H]^+$): 281.1753, found: 281.1755. ^1H NMR (500 MHz, $CDCl_3$) (two rotamers) δ = 5.50 (brs, 1H), 4.79 (m, 3H), 3.61 (m, 2H), 3.50 (m, 2H), 3.25 (s, 6H), 3.09 (m, 2H), 2.97-2.58 (m, 3H), 2.42 (m, 4H), 2.30 (m, 2H), 2.08 (m, 4H), 1.83 (m, 2H), 1.68 (m, 3H) 1.53 (brs, 2H), 1.45 (s, 6H), 0.88 (d, J = 6.8 Hz, 6H). ^{13}C NMR (125 MHz, $CDCl_3$) δ = 211.2, 176.7, 132.1, 125.1, 74.8, 69.0, 58.2, 44.2, 42.8, 42.1, 41.9, 36.1, 34.9, 24.1, 18.0, 14.2.

(3S,3aR,4aR,5R,7aR,9aR)-4a-hydroxy-3-(methoxymethyl)-5-methyl-8-methylenedecahydroazuleno[6,5-b]furan-2(3H)-one (2)

Compound 3 (55 mg, 0.2 mmol) was dissolved in toluene (1 mL) in a sealed tube and it was left stirring at 170°C for 12 h before it was evaporated to dryness. The residue was chromatographed (silica gel) with gradient from 10:1 benzene: Et_2O to 8:1 benzene:Et_2O [R_f = 0.2 (benzene:Et_2O = 4:1, UV active on TLC, stains greenish upon *p*-anisaldehyde staining)] to afford pure **Compound 2** as a white amorphous solid (40 mg, 72%). $[\alpha]^{20}_D$ = +34.3 (*c* 0.23, $CHCl_3$). HRMS (ESI, m/z): calcd. for $C_{16}H_{25}O_4^+$ ($[M+H]^+$): 281.1747, found: 281.1747. ^1H NMR (500 MHz, $CDCl_3$) δ = 5.14 (s, 1H), 4.95 (s, 1H), 4.58, (ddd, *J* = 12.2, 8.7, 3.5 Hz, 1H), 3.66 (qd, *J* = 9.4, 4.2 Hz, 2H), 3.36 (s, 3H), 2.94 (dtd, *J* = 11.8, 9.1, 5.9 Hz, 1H), 2.74 (dd, *J* = 12.6, 3.4 Hz, 1H), 2.53-2.47 (m, 1H), 2.29 (dt, *J* = 11.7, 5.7 Hz, 3H), 1.96-1.76 (m, 3H), 1.74-1.67 (m, 1H), 1.51 (dd, *J* = 14.4, 11.8 Hz, 2H), 1.36 (m, 1H), 0.96 (d, *J* = 6.7 Hz, 3H).^{13}C NMR (125 MHz, $CDCl_3$) δ = 176.3, 142.6, 115.1, 82.0, 79.5, 70.6, 59.2, 58.4, 47.3, 43.4, 40.7, 37.5, 36.2, 28.6, 24.0, 12.9.

6.6 Enrichment of the chemical space of guaianolide 2.

6.6.1 Reduction-Dehydration-Oxidation sequence

After securing an efficient and scalable synthetic route for guaianolide **2**, we aimed to enrich its chemical space by selectively manipulating the positions C-4, C-5 and C-10 which were found to be of importance on the pharmacophore of sesquiterpenoid molecules showing anticancer activity. In order to achieve high selectivity in transformations and a wide scope of divergency in structures, three main axes were followed: reduction, dehydration and oxidation. The critically positioned C-5 hydroxyl serves as the pinpoint to induce selectivity.

Firstly, hydrogenation of **2** under Adam's catalyst resulted in a 6:1 mixture of **19:20** favoring the α-orientation of the methyl group. Altering the catalyst to Wilkinson resulted to a 1:2 mixture of **19:20**, as a result of hydroxyl-biased hydrogenation. Dehydration of **19** and **20** applying different conditions led to some impressive results. Syn-regiospecific dehydration of **19** by Burgess reagent but also dehydration using 10% conc. sulfuric acid in 1,4-dioxane provided exclusively the alkene **21**. On the contrary, reaction of **20** with 10% conc. sulfuric acid in 1,4-dioxane results in a slight preference for the C4-C5 dehydration generating alkenes **22**:**23** in a 1:2 ratio. Interestingly, when alkenes **21**-**22** were treated with conc. sulfuric acid in benzene compound **25** was delivered indicating an oxidative process taking place (see table S11, page SI-50). Finally, allylic oxidation of alkene **23** under Baran's protocol[16] afforded cyclopentenone **26**, while epoxidation of **21** using mCPBA yielded the epoxide **24** in a 4:1 ratio with its isomer.

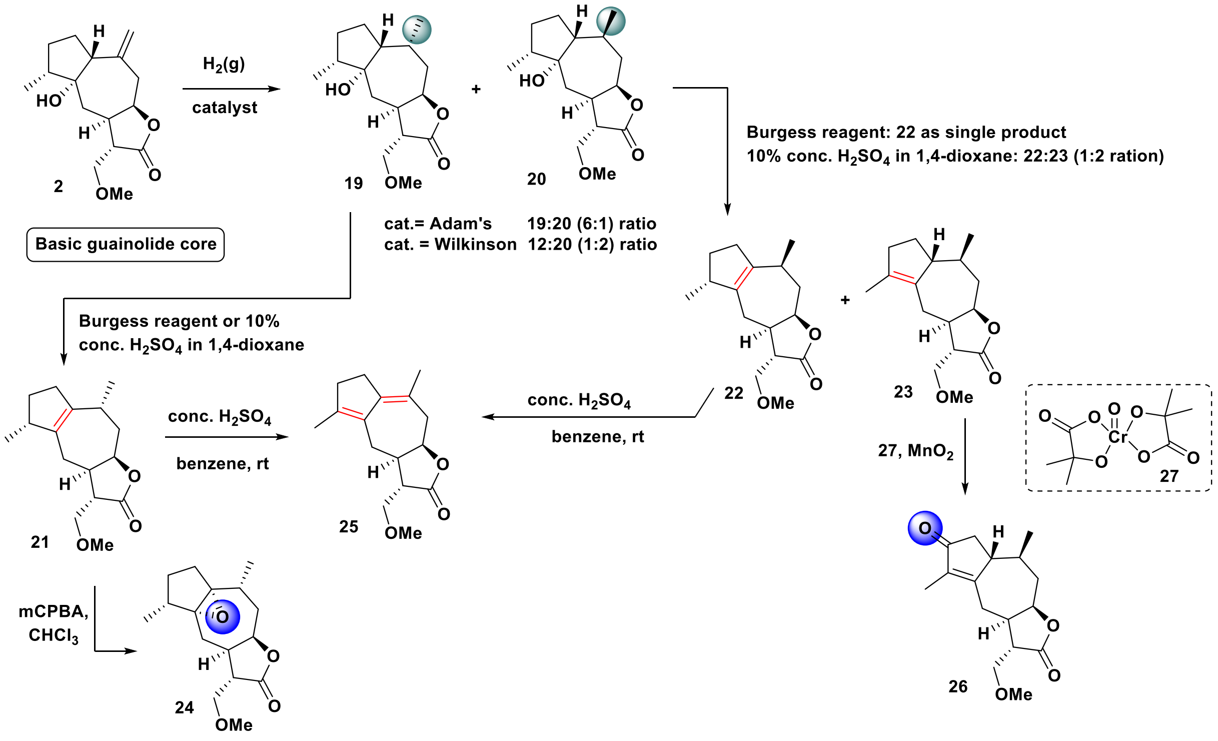

Scheme S15: Reductive manipulation of C-10, dehydration of $\Delta^{4,5}$ double bond and oxidative manipulation.

3S,3aR,4aR,5R,7aR,8R,9aR)-4a-hydroxy-3-(methoxymethyl)-5,8-dimethyldecahydroazuleno[6,5-b]furan-2(3H)-one (19)

Method A:

Compound 2 (40 mg, 0.14 mmol, 1.0 eq) was dissolved in CH_3OH (4 mL) in a sealed tube, Adams catalyst (PtO_2*H_2O) (3 mg, 10%mmol) was added, followed by bubbling with H_2 for 15 min. The tube was then sealed and the reaction mixture

was left stirring at r.t. for 2 h. The resulting black suspension is filtered through Celite® and the filtrate was evaporated to dryness. The residue was chromatographed (silica gel) with gradient from 8:1 benzene: Et_2O to 5:1 benzene: Et_2O to afford pure **Compound 19** as a white amorphous solid (37 mg, 93%).

Method B:

Compound 2 (106 mg, 0.38 mmol, 1.0 eq) was dissolved in benzene (12 mL) in a sealed tube, Wilkinson catalyst ($RhCl(PPh_3)_3$) (70 mg, 20%mmol) was added, followed by bubbling with H_2 for 30 min. The tube was the sealed and the reaction mixture was left stirring at 45°C for 12 h. The resulting orange suspension is filtered through Celite® and the filtrate was evaporated to dryness. The residue was chromatographed (silica gel) with gradient from 15:1 benzene: Et_2O to 8:1 benzene: Et_2O [R_f = 0.24 (benzene:Et_2O = 4:1, UV inactive on TLC, stains greenish upon *p*-anisaldehyde staining)] to afford pure **Compound 19** as a white amorphous solid (33 mg, 31%). $[\alpha]^{20}_D$ = +46.7 (*c* 0.3, $CHCl_3$). HRMS (ESI, m/z): calcd for $C_{16}H_{27}O_4^+$ ($[M+H]^+$): 283.1909, found: 283.1912. ^1H NMR (500 MHz, $CDCl_3$) δ = 5.02 (ddd, *J* = 11.9, 8.7, 2.8 Hz, 1H), 3.70 – 3.62 (m, 2H), 3.37 (s, 3H), 3.01 – 2.91 (m, 1H), 2.48 (dt, *J* = 8.3, 3.7 Hz, 1H), 2.21 – 2.11 (m, 2H), 2.05 – 2.01 (m, 1H), 1.94 – 1.85 (m, 2H), 1.78 – 1.67 (m, 2H), 1.60 – 1.54 (m, 2H), 1.40 (dd, *J* = 14.3, 11.8 Hz, 1H), 1.33 – 1.28 (m, 2H), 1.15 (d, *J* = 7.3 Hz 3H), 0.88 (d, *J* = 6.9 Hz, 3H). ^{13}C NMR (125 MHz, $CDCl_3$) δ = 176.8, 82.7, 78.8, 70.6, 59.2, 55.2, 47.3, 43.7, 37.8, 37.1, 36.7, 29.9, 28.8, 24.9, 13.9, 12.4.

(3S,3aR,4aR,5R,7aR,8S,9aR)-4a-hydroxy-3-(methoxymethyl)-5,8-dimethyldecahydroazuleno[6,5-b]furan-2(3H)-one (20)

Compound 2 (106 mg, 0.38 mmol, 1.0 eq) was dissolved in benzene (12 mL) in a sealed tube, Wilkinson catalyst ($RhCl(PPh_3)_3$) (70 mg, 20%mmol) was added, followed by bubbling with H_2 for 30 min. The tube was the sealed and the reaction mixture was left stirring at 45°C for 12 h. The resulting orange suspension is filtered through Celite® and the filtrate was evaporated to dryness. The residue was chromatographed (silica gel) with gradient from 15:1 benzene: Et_2O to 8:1 benzene: Et_2O [R_f = 0.22 (benzene:Et_2O = 4:1, UV inactive on TLC, stains greenish upon *p*-anisaldehyde staining)] to afford pure **Compound 20** as a white amorphous solid (61 mg, 57%). $[\alpha]^{20}_D$ = -35.2 (*c* 0.3, $CHCl_3$). HRMS (ESI, m/z): calcd for $C_{16}H_{27}O_4^+$ ($[M+H]^+$): 283.1909, found: 283.1910. ^1H NMR (500 MHz, $CDCl_3$) δ = 4.83 – 4.78 (m, 1H), 3.65 (d, J = 4.4 Hz, 2H), 3.36 (s, 3H), 2.88 (ddd, J = 15.6, 11.3, 8.5 Hz, 1H), 2.47 (dt, J = 11.4, 4.4 Hz, 1H), 2.31 (dd, J = 14.3, 6.9 Hz, 1H), 1.86 – 1.78 (m, 3H), 1.65 – 1.60 (m, 2H), 1.57 (s, 1H), 1.35 – 1.28 (m, 3H), 1.25 (s, 1H), 0.96 (d, J = 6.3 Hz, 3H), 0.91 (d, J = 6.7 Hz, 3H).^{13}C NMR (125 MHz, $CDCl_3$) δ = 176.5, 82.4, 80.5, 70.1, 59.3, 59.2, 46.5, 43.4, 39.3, 36.8, 36.6, 32.3, 28.6, 27.3, 21.7, 12.4.

The diverse results on the dehydration attempts on guaiane scaffolds **19** and **20** are summarized on the table below.

Table S11. All dehydration attempts on compounds **19**:**20**.

Entry	*Starting Material*	*Reagent*	*Solvent*	*Temperature*	*Time*	*Product(s)*
**1**	**19**	Burgess	toluene	110°C	12h	**21**
**2**	**19**	TFA	dry CH_2Cl_2	rt	24h	**21**

**3**	**19**	conc.H_2SO_4	1,4-dioxane	rt	24h	**21**
**4**	**20**	conc.H_2SO_4	1,4-dioxane	rt	24h	**23:22** (2:1)
**5**	**19/20**	conc.H_2SO_4	benzene	rt	10min	**25**
**6**	**21/22**	conc.H_2SO_4	benzene	rt	10imn	**25**

(3S,3aR,5R,8R,9aR)-3-(methoxymethyl)-5,8-dimethyl-3a,4,5,6,7,8,9,9a-octahydroazuleno[6,5-b]furan-2(3H)-one (21)

Compound 19 (3 mg, 0.010 mmol, 1.0 eq) was dissolved in toluene (1 mL) in a sealed tube before Burgess reagent was added (10 mg, 0.040 mmol, 4 eq) and the resulting reaction mixture was left stirring at 110 °C for 12 h before it was evaporated to dryness. The residue was chromatographed (silica gel) with gradient from 10:1 benzene: Et_2O to 8:1 benzene: Et_2O [R_f = 0.9 (cyclohexane:Et_2O = 4:1, UV active on TLC, stains blue upon *p*-anisaldehyde staining)] to afford pure **Compound 21** as a colorless oil (1.6 mg, 60%). $[\alpha]^{20}_D$ = +28.7 (c 0.4, $CHCl_3$). HRMS (ESI, m/z): calcd for $C_{16}H_{24}O_3Na^+$ ($[M+Na]^+$): 287.1617, found: 287.1616. ^1H NMR (500 MHz, $CDCl_3$) δ= 4.86 (td, J = 8.1, 3.4 Hz, 1H), 3.67 (dd, J = 9.3, 5.1 Hz, 2H), 3.56 (dd, J = 9.3, 3.4 Hz, 2H), 3.34 (s, 3H), 2.83 – 2.76 (m, 1H), 2.60 (s, 1H), 2.43 (d, J = 7.1 Hz, 21H), 2.25 (d, J = 4.6 Hz, 2H), 2.14 – 1.94 (m, 3H), 1.38 – 1.29 (m, 1H), 1.05 (d, J = 7.1 Hz, 3H), 0.97 (d, J = 6.7 Hz, 3H).^{13}C NMR (125 MHz, $CDCl_3$) δ = 177.8, 140.2, 136.5, 80.3, 71.3, 59.2, 50.4, 45.8, 41.3, 36.1, 34.3, 30.8, 30.5, 26.9, 20.1, 19.4.

(3S,3aR,5R,8S,9aR)-3-(methoxymethyl)-5,8-dimethyl-3a,4,5,6,7,8,9,9a-octahydroazuleno[6,5-b]furan-2(3H)-one (22)

To a vial **compound 19** (21 mg, 0.074 mmol, 1.0 eq) was dissolved in 2.6 mL of 10% solution of conc. H_2SO_4 in 1,4-dioxane. The reaction mixture was left stirring overnight at room temperature before it was quenched with 4 mL saturated aqueous $NaHCO_3$. After separation, the aqueous layer was extracted three times with EtOAc (3x 4 mL). The combined organic extracts were dried over Na_2SO_4, filtered, and evaporated under reduced pressure. The residue was chromatographed (silica gel) with gradient from 15:1 pentane:EtOAc to 6:1 pentane:EtOAc [R_f = 0.32 (n-pentane:Et_2O= 5:1, UV inactive on TLC, stains green upon *p*-anisaldehyde staining)] to afford **Compounds 22:23** as a colorless oil in a 1:2 inseparable mixture (17 mg, 87%).

(3S,3aR,7aR,8S,9aR)-3-(methoxymethyl)-5,8-dimethyl-3a,4,6,7,7a,8,9,9a-octahydroazuleno[6,5-b]furan-2(3H)-one (23)

To a vial **compound 20** (21 mg, 0.074 mmol, 1.0 eq) was dissolved in 2.6 mL of 10% solution of conc. H_2SO_4 in 1,4-dioxane. The reaction mixture was left stirring overnight at room temperature before it was quenched with 4 mL saturated aqueous $NaHCO_3$. After separation, the aqueous layer was extracted three times with EtOAc (3x 4 mL). The combined organic extracts were dried over Na_2SO_4, filtered, and evaporated under reduced pressure. The residue was chromatographed (silica gel) with gradient from 15:1 pentane:EtOAc to 6:1 pentane:EtOAc [R_f= 0.28 (n-pentane:EtOAc= 5:1, UV inactive on TLC, stains green upon *p*-anisaldehyde staining)] to afford **Compounds 22:23** as a colorless oil in a 1:2 inseparable mixture (17 mg, 87%). $[\alpha]^{20}_D$ = -18.9 (c 0.5, $CHCl_3$). HRMS (ESI, m/z): calcd for $C_{16}H_{24}O_3K^+$ ($[M+K]^+$): 303.1357, found: 303.1357. ^1H NMR (500 MHz, $CDCl_3$) δ = 4.53 (dd, *J* = 15.3, 7.9 Hz, 1H), 3.67 (dd, *J* = 9.1, 4.4 Hz, 2H), 3.37 (s, 3H), 2.66 – 2.61 (m, 2H), 2.51 – 2.46 (m, 1H), 2.35 – 2.26 (m, 2H), 2.20 – 2.11 (m, 2H), 2.04 (ddd, *J* =

14.6, 9.1, 3.4 Hz, 1H), 1.81 (dd, *J* = 10.1, 6.6 Hz, 2H), 1.63 (s, 3H), 1.46 – 1.39 (m, 1H), 1.26 (s, 1H), 1.00 (d, *J* = 6.5 Hz, 3H). ^{13}C NMR (125 MHz, $CDCl_3$) δ = 176.9, 134.7, 134.1, 82.5, 70.8, 59.3, 57.6, 47.9, 40.1, 38.1, 37.8, 36.5, 29.0, 28.6, 22.3, 13.9.

(3S,3aR,4aR,5R,7aR,8R,9aR)-3-(methoxymethyl)-5,8-dimethylhexahydro-4H,5H-4a,7a-epoxyazuleno[6,5-b]furan-2(3H)-one (24)

To a vial **compound 21** (21 mg, 0.08 mmol, 1.0 eq) was dissolved in $CHCl_3$ (3 mL), $NaHCO_3$ and mCPBA (18.3 mg, 0.08 mmol, 1 eq) were added successively. The reaction mixture was stirred vigorously for 30 min before it was quenched with saturated aqueous $Na_2S_2O_3$ (4 mL). After separation, the aqueous layer was extracted three times with CH_2Cl_2 (3x 4 mL). The combined organic extracts were washed on time with saturated aqueous $NaHCO_3$, dried over Na_2SO_4, filtered, and evaporated under reduced pressure. The residue was chromatographed (silica gel) with gradient from 15:1 n-hexane:EtOAc to 1:1 n-hexane:EtOAc [R_f= 0.39 (n-hexane:EtOAc= 2:1, UV inactive on TLC, stains green upon *p*-anisaldehyde staining)] to afford pure **Compound 24** as a colorless oil (13 mg, 58%). $[\alpha]^{20}_D$ = +24.7 (*c* 0.3, $CHCl_3$). HRMS (ESI, m/z): calcd for $C_{16}H_{25}O_4^+$ ($[M+H]^+$): 281.3713, found: 281.3716. ^{1}H NMR (500 MHz, $CDCl_3$) δ= 4.74 – 4.69 (m, 1H), 3.67 – 3.60 (m, 2H), 3.35 (s, 3H), 2.82 (ddd, J = 17.7, 9.3, 3.6 Hz, 1H), 2.48 – 2.43 (m, 1H), 2.21 – 2.09 (m, 3H), 2.05 (dd, J = 13.8, 8.3 Hz, 1H), 1.89 – 1.83 (m, 1H), 1.79 – 1.74 (m, 2H), 1.61 (dt, J = 12.7, 8.0 Hz, 1H), 1.46 (ddd, J = 13.9, 10.5, 8.5 Hz, 1H), 1.12 (d, J = 7.0 Hz, 3H), 1.00 (d, J = 6.7 Hz, 3H), 0.98 – 0.92 (m, 1H).^{13}C NMR (125 MHz, $CDCl_3$) δ = 176.8, 79.2, 72.9, 72.3, 71.0, 59.3, 45.3, 39.0, 37.2, 33.1, 31.0, 30.0, 27.7, 26.7, 17.6, 13.5.

(3S,3aR,7aR,8S,9aR)-3-(methoxymethyl)-5,8-dimethyl-3a,7,7a,8,9,9a-hexahydroazuleno[6,5-b]furan-2,6(3H,4H)-dione (26)

To vial **compound 23** (7.8 mg, 0.03 mmol, 1.0 eq) was dissolved in toluene (2 mL) and Cr-complex **27** (40.15 mg, 0.15 mmol, 5.0 eq), MnO_2 (128.2 mg, 1.5 mmol, 50 eq) and 18-crown-6 (31.16mg, 0.12 mmol, 4.0 eq) were added successively. The reaction mixture was left stirring vigorously at 85°C overnight (aprox. 20h) before it was filtered through silica gel and the filtrate was evaporated to dryness. The residue was chromatographed (silica gel) with gradient from 15:1 n-hexane:EtOAc to EtOAc [R_f= 0.17 (n-hexane:EtOAc= 2:1, UV active on TLC, stains yellow upon *p*-anisaldehyde staining)] to afford pure **Compound 26** as a colorless oil (2 mg, 25%). $[\alpha]^{20}_D$ = -19.7 (*c* 0.6, $CHCl_3$). HRMS (ESI, m/z): calcd for $C_{16}H_{23}O_4^+$ ($[M+H]^+$): 279.1591, found: 279.1590. ^{1}H NMR (500 MHz, $CDCl_3$) δ= 4.51 (dd, J = 15.2, 8.5 Hz, 1H), 3.72 – 3.69 (m, 2H), 3.39 (s, 3H), 3.01 (dd, J = 12.7, 4.9 Hz, 1H), 2.80 – 2.71 (m, 1H), 2.66 – 2.58 (m, 2H), 2.32 (t, J = 12.6 Hz, 2H), 2.06 (d, J = 19.2 Hz, 1H), 1.96 (d, J = 6.8 Hz, 2H), 1.72 (s, 3H), 1.25 (s, 1H), 1.14 (d, J = 6.7 Hz, 3H).^{13}C NMR (125 MHz, $CDCl_3$) δ = 208.0, 175.7, 170.9, 137.5, 81.3, 70.8, 59.3, 50.2, 47.7, 41.8, 38.8, 38.6, 37.5, 31.6, 22.9, 7.8.

6.6.2 Dehydration-Reduction or Oxidation sequence

Attempts to initially dehydrate **2** proved problematic. Dehydration using Lewis Acids (eg. BF_3Et_2O) or organic acids (eg. HCl) failed to initiate any reaction, while conc. sulfuric acid resulted in the formation of diene **25** as the sole product. Thionyl chloride or sulfuric chloride led to unselective formation of alkenes **28-30** through E1 elimination mechanism. Similarly, burgess reagent yielded an inseparable mixture of alkenes **28**:**29**:**30** in 0.5:1.5:1 ratio. Since, burgess is known

to perform syn-elimination the formation of alkene **30** must derive from the spontaneous isomerization of alkene **28** considering it is more thermodynamically stable. The alkenes mixture was treated with mCPBA providing epoxides **32** and **33**. However, alkene **28** didn't produce an epoxide hence it was recovered and hydrogenated with Adam's catalyst leading to compound **31**. Finally, hydrogenation of epoxide **33** afforded compound **34.**

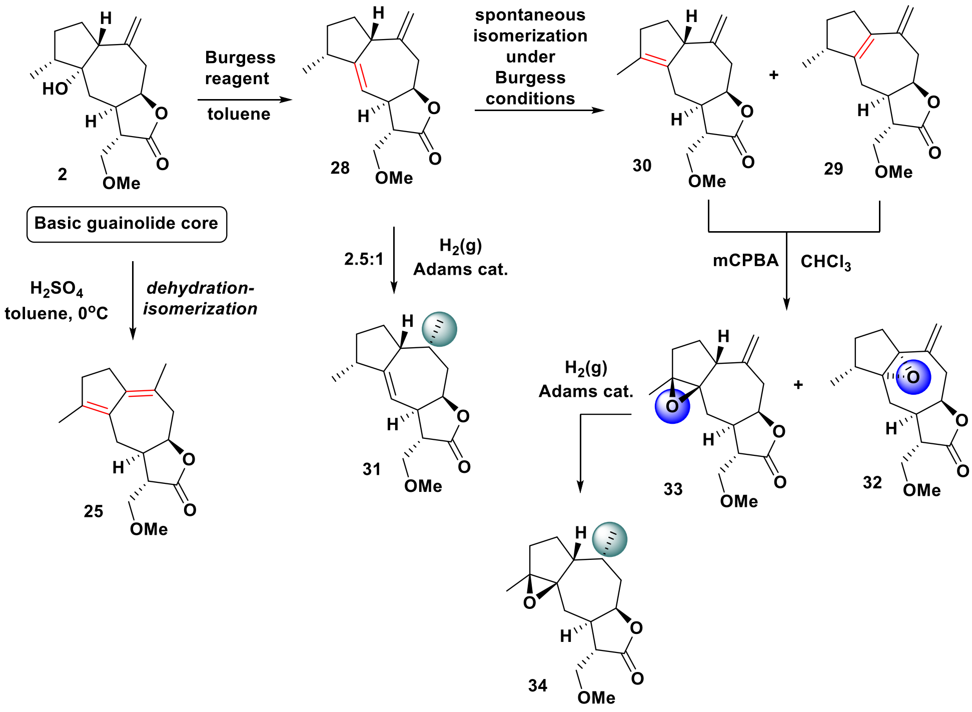

Scheme S16. Initial dehydration of common scaffold **2** followed by reductive/oxidative transformations

(3S,3aR,9aR)-3-(methoxymethyl)-5,8-dimethyl-3a,4,6,7,9,9a-hexahydroazuleno-[6,5-b]furan-2(3H)-one (25)

To a round bottom flask, **Compound 2** (37 mg, 0.13 mmol, 1.0 eq) was dissolved in toluene (3 mL), silica gel was added and cooled at 0°C before catalytic amount of concentrated H_2SO_4 was added. The reaction stirred for 5h at 0°Cbefore it was quenched with 3 mL saturated aqueous $NaHCO_3$. After separation, the aqueous layer was extracted three times with EtOAc (3x 3 mL). The combined organic extracts were dried over Na_2SO_4, filtered, and evaporated under reduced pressure. The residue was chromatographed (silica gel) with gradient from 14:1 hexane:EtOAc to 9:1 hexane:EtOAc [R_f= 0.68 (hexane:EtOAc= 2:1, UV active on TLC, stains green upon *p*-anisaldehyde staining)] to afford pure **Compound 25** as a colorless oil (10 mg, 35% brsm). $[\alpha]^{20}_D$ = +16.7 (*c* 0.8, $CHCl_3$). HRMS (ESI, m/z): calcd for: $C_{16}H_{22}O_3Na^+$ $[M+Na]^+$ 285.1461, found:285.1463. ^{1}H NMR (500 MHz, $CDCl_3$) δ= 4.73 (td, *J* = 9.3, 2.9 Hz, 1H), 3.68 (dd, *J* = 9.4, 5.3 Hz, 1H), 3.62 (dd, *J* = 9.3, 3.6 Hz, 1H), 3.36 (s, 3H), 2.76 (ddd, *J* = 19.1, 8.9, 3.6 Hz, 2H), 2.62 (dd, *J* = 14.7, 3.8 Hz, 1H), 2.56 – 2.51 (m, 1H), 2.45 – 2.31 (m, 6H), 1.73 (s, 3H), 1.72 (s, 3H). ^{13}C NMR (125 MHz, $CDCl_3$) δ = 177.0, 144.6, 142.1, 134.9, 116.8, 81.4, 71.1, 59.2, 39.2, 35.5, 35.3, 29.7, 27.9, 27.4, 23.0, 14.7.

Non-selective dehydration of compound 2. Formation of inseparable mixture of alkenes 28-30.

Compound 2 (80 mg, 0.28 mmol, 1.0 eq) was dissolved in a sealed tube in toluene (2 mL) and burgess reagent was added (200 mg, 0.85, 3.0 eq). The reaction mixture was heated at 80°C and stirred for 1h before it was evaporated to dryness. The residue was chromatographed (silica gel) with gradient from 30:1 benzene:Et_2O to 25:1 benzene:Et_2O [R_f= 0.65 (benzene:Et_2O= 4:1, UV active on TLC, stains green upon *p*-anisaldehyde staining)] to afford the inseparable mixture of **Compounds 28-30** as a pale yellow oil (26 mg, 33%).

Epoxidation of the alkenes 28-30 mixture. Formation of epoxides 32 and 33.

To a stirring solution of the mixture of **compounds 28-30** (26 mg, 0.1 mmol, 1.0 eq) in $CHCl_3$ (3 mL), $NaHCO_3$ and mCPBA (22 mg, 0.1 mmol, 1 eq) were added successively. The reaction mixture was stirred vigorously for 3.5h before it was quenched with saturated aqueous $Na_2S_2O_3$ (10 mL). After separation, the aqueous layer was extracted three times with CH_2Cl_2 (3x 10 mL). The combined organic extracts were washed on time with saturated aqueous $NaHCO_3$, dried over Na_2SO_4, filtered, and evaporated under reduced pressure. The residue was chromatographed to afford the pure epoxides.

(3S,3aR,5R,7aR,9aR)-3-(methoxymethyl)-5-methyl-8-methylene-3a,5,6,7,7a,8,9,9a-octahydroazuleno[6,5-b]furan-2(3H)-one (28)

Didn't yield an epoxide. Retrieved in gradient n-pentane: Et_2O 4:1 (4.5mg, 15%) [R_f= 0.9 (n-pentane:Et_2O= 1:1, UV active on TLC, stains green upon *p*-anisaldehyde staining)]as a colorless oil. $[\alpha]^{20}_D$ = -32.5 (*c* 0.3, $CHCl_3$). HRMS (ESI, m/z): calcd for: $C_{16}H_{23}O_3^+$ $[M+H]^+$ 263.1642, found:263.1642. ^1H NMR (500 MHz, $CDCl_3$) δ= 5.53 (dt, J = 8.7, 2.4 Hz, 1H), 5.03 (s, 1H), 4.97 (s, 1H), 4.76 (dt, J = 8.9, 3.6 Hz, 1H), 3.64 (dd, J = 9.7, 4.1 Hz, 1H), 3.50 (dd, J = 9.7, 3.5 Hz, 1H), 3.34 (s, 3H), 3.32 – 3.26 (m, 1H), 2.80 (dt, J = 11.8, 3.8 Hz, 1H), 2.62 (s, 1H), 2.46 (qd, J = 13.6, 3.6 Hz, 2H), 1.87 (ddd, J = 16.6, 10.9, 5.2 Hz, 3H), 1.61 (ddd, J = 11.8, 10.2, 4.0 Hz, 1H), 1.36 (ddd, J = 9.8, 6.8, 4.3 Hz, 1H), 1.07 (d, J = 7.0 Hz, 3H).^{13}C NMR (125 MHz, $CDCl_3$) δ = 177.0, 154.2, 142.7, 116.1, 115.8, 80.4, 69.3, 59.1, 51.19, 47.9, 38.4, 37.8, 36.7, 32.7, 32.2, 20.9.

(3S,3aR,4aR,5R,7aR,9aR)-3-(methoxymethyl)-5-methyl-8-methylenehexahydro-4H,5H-4a,7a-epoxyazuleno[6,5-b]furan-2(3H)-one (32)

Isolated in a gradient n-pentane:Et_2O 4:1 [R_f= 0.68 (n-pentane:Et_2O= 1:1, UV active on TLC, stains brown upon *p*-anisaldehyde staining)] as a white amorphous solid in 44% yield. $[\alpha]^{20}_D$ = +27.3 (*c* 0.3, $CHCl_3$). HRMS (ESI, m/z): calcd for: $C_{16}H_{23}O_4^+$ $[M+H]^+$ 279.1591, found:279.1590. ^1H NMR (500 MHz, $CDCl_3$) δ= 5.35 (s, 1H), 5.25 (s, 1H), 4.67 – 4.63 (m, 1H), 3.63 (dd, J = 9.4, 3.6 Hz, 1H), 3.56 (dd, J = 9.4, 6.0 Hz, 1H), 3.34 (s, 3H), 2.87 – 2.80 (m, 1H), 2.63 (dd, J = 14.4, 3.2 Hz, 1H), 2.44 (dd, J = 14.4, 4.7 Hz, 1H), 2.39 (ddd, J = 11.0, 6.0, 3.6 Hz, 1H), 2.31 (dd, J = 15.9, 6.7 Hz, 1H), 2.15 (dd, J = 15.9, 2.6 Hz, 1H), 2.05 – 2.00 (m, 2H), 1.95 – 1.86 (m, 2H), 1.66 (dt, J = 12.6, 7.9 Hz, 1H), 1.06 (d, J = 6.7 Hz, 3H). ^{13}C NMR (125 MHz, $CDCl_3$) δ= 176.5, 140.1, 120.5, 79.0, 71.7, 71.0, 70.9, 59.3, 44.1, 40.0, 38.6, 34.6, 28.5, 27.4, 26.2, 13.3.

(1aS,3aR,5aR,8S,8aR,9aR)-8-(methoxymethyl)-1a-methyl-4-methyleneoctahydro-3H-oxireno[2',3':3,3a]azuleno [6,5-b]furan-7(8H)-one (33)

Isolated in a gradient n-pentane:Et_2O 1:1 [R_f= 0.56 (CH_2Cl_2:acetone= 50:1, UV active on TLC, stains brown upon *p*-anisaldehyde staining)] as a white amorphous oil in 40% yield. $[\alpha]^{20}_D$ = +30.6 (*c* 0.6, $CHCl_3$). HRMS (ESI, m/z): calcd for: $C_{16}H_{23}O_4^+$ $[M+H]^+$ 279.1591, found:279.1591. ^1H NMR (500 MHz, $CDCl_3$) δ= 5.06 (s, 1H), 4.93 (s, 1H), 4.68 – 4.62 (m, 1H), 3.69 (dd, J = 9.1, 4.9 Hz, 1H), 3.64 (dd, J = 9.2, 3.5 Hz, 1H), 3.34 (s, 3H), 2.89 (dd, J = 12.8, 4.5 Hz, 1H), 2.47 (dd, J = 8.9, 5.1 Hz, 2H), 2.43 – 2.36 (m, 2H), 2.18 – 2.10 (m, 2H), 2.00 (dd, J = 13.7, 7.9 Hz, 1H), 1.80 (s, 1H), 1.69 – 1.60 (m, 2H), 1.37 (s, 3H).^{13}C NMR (125 MHz, $CDCl_3$) δ= 176.8, 141.0, 114.4, 81.7, 71.7, 69.4, 69.1, 59.2, 50.5, 49.0, 39.5, 37.8, 31.7, 30.2, 21.6, 15.7.

(3S,3aR,5R,7aR,8R,9aR)-3-(methoxymethyl)-5,8-dimethyl-3a,5,6,7,7a,8,9,9a-octahydroazuleno[6,5-b]furan-2(3H)-one (31)

Compound 28 (4.5 mg, 0.017 mmol, 1.0 eq) was dissolved in CH_3OH (2 mL) in a sealed tube, Adams catalyst (PtO_2*H_2O) (0.4 mg, 10%mmol) was added, followed by bubbling with H_2 for 15 min. The tube was then sealed, and the reaction mixture was left stirring at r.t. for 20 min. The resulting black suspension is filtered through Celite® and the filtrate was evaporated to dryness. The residue was chromatographed (silica gel) with gradient from 10:1 n-pentane:Et_2O to 180:1 n-pentane:Et_2O [R_f= 0.62 (n-pentane:Et_2O= 6:1, UV active on TLC, stains brown upon *p*-anisaldehyde staining)] to afford pure **Compound 31** as a white amorphous solid (3 mg, 67%). $[\alpha]^{20}_D$ = -39.4 (*c* 0.5, $CHCl_3$). HRMS (ESI, m/z): calcd for $C_{16}H_{24}O_3K^+$ ($[M+K])^+$:303,1357, found: 303,1359. ^1H NMR (500 MHz, $CDCl_3$) δ= 5.41 (d, J = 7.7 Hz, 1H), 4.69 (ddd, J = 11.7, 7.2, 4.5 Hz, 1H), 3.74 (dd, J = 9.7, 3.6 Hz, 1H), 3.62 (dd, J = 9.8, 3.7 Hz, 1H), 3.38 (s, 3H), 2.68 – 2.63 (m, 1H), 2.62 – 2.58 (m, 1H), 2.56 – 2.51 (m, 1H), 2.25 – 2.19 (m, 1H), 2.12 – 2.01 (m, 1H), 1.80 – 1.65 (m, 6H), 1.00 (d, J = 7.1 Hz, 3H), 0.89 (d, J = 7.2 Hz, 3H).^{13}C NMR (125 MHz, $CDCl_3$) δ= 176.6, 152.4, 114.0, 82.5, 68.4, 59.3, 49.6, 45.5, 41.3, 40.8, 39.1, 32.6, 30.3, 30.1, 29.3, 20.5, 12.3.

(1aS,3aR,4R,5aR,8S,8aR,9aR)-8-(methoxymethyl)-1a,4-dimethyloctahydro-3H-oxireno[2',3':3,3a]azuleno[6,5-b]furan-7(8H)-one (34)

Compound 33 (5 mg, 0.018 mmol, 1.0 eq) was dissolved in CH_3OH (1 mL) in a sealed tube, Adams catalyst (PtO_2*H_2O) (0.41 mg, 10%mmol) was added, followed by bubbling with H_2 for 15 min. The tube was then sealed, and the reaction mixture was left stirring at r.t. for 20 min. The resulting black suspension is filtered through Celite® and the filtrate was evaporated to dryness. The residue was chromatographed (silica gel) with gradient from 250:1 CH_2Cl_2:Acetone to 185:1 CH_2Cl_2:Acetone [R_f=0.6 (CH_2Cl_2:Acetone =50:1, UV inactive on TLC, stains brown upon *p*-anisaldehyde staining)] to afford pure **Compound 34** as a white amorphous solid (4 mg, 69%). $[\alpha]^{20}_D$ = -28.7 (*c* 0.2, $CHCl_3$). HRMS (ESI, m/z): calcd for $C_{16}H_{24}O_4Na^+$ ($[M+Na])^+$:303,1567, found: 303,1566. ^1H NMR (500 MHz, CDCl3) δ= 4.96 – 4.90 (m, 1H), 3.73 (dd, J = 9.0, 4.4 Hz, 1H), 3.64 (dd, J = 9.0, 3.4 Hz, 1H), 3.35 (s, 2H), 2.86 – 2.79 (m, 1H), 2.39 (ddd, J = 13.4, 7.4, 4.5 Hz, 2H), 2.19 – 2.13 (m, 1H), 2.13 – 2.06 (m, 1H), 2.01 – 1.92 (m, 2H), 1.84 – 1.77 (m, 1H), 1.41 (dd, J = 14.5, 3.1 Hz, 2H), 1.32 (s, 3H), 1.03 (d, J = 7.4 Hz, 3H). ^{13}C NMR (125 MHz, CDCl3) δ= 177.3, 80.7, 72.2, 70.3, 67.4, 59.3, 50.6, 50.1, 38.5, 36.4, 32.2, 31.3, 29.7, 28.9, 15.6, 13.7.

6.6.3 (formal)Oxidation-Dehydration sequence

Switching to initial (formal)oxidation of the basic guaiane core **2**, hydroxyl-directed epoxidation afforded α-epoxide **35** using mCPBA in HFIP. Subsequent dehydration under Burgess reagent conditions led exclusively to alkene **38**.

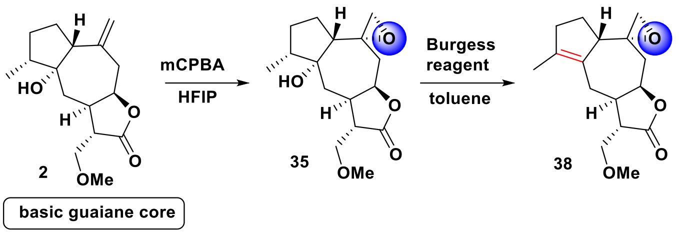

Scheme S17: Enantioselective epoxidation of guaianolide **2**.

(3S,3aR,4aR,5R,7aS,8R,9aR)-4a-hydroxy-3-(methoxymethyl)-5-methyldecahydro-2H-spiro[azuleno[6,5-b]furan-8,2'-oxiran]-2-one (35)

To a round bottom flask **Compound 2** (65 mg, 0.23 mmol, 1.0 eq) was dissolved in HFIP(9 mL), $NaHCO_3$ and mCPBA (80 mg, 0.35 mmol, 1.5 eq) were added successively. The reaction mixture was stirred vigorously for 5h before it was quenched with saturated aqueous $Na_2S_2O_3$ (10 mL). After separation, the aqueous layer was extracted three times with CH_2Cl_2 (3x 10 mL). The combined organic extracts were washed on time with saturated aqueous $NaHCO_3$, dried over Na_2SO_4, filtered, and evaporated under reduced pressure. The residue was chromatographed (silica gel) with gradient from 15:1 hexane:EtOAc to 3:1 hexane:EtOAc [R_f = 0.31 (benzene:Et_2O = 1:1, UV inactive on TLC, stains blue upon *p*-anisaldehyde staining)] to afford pure **Compound 35** as a white amorphous solid (45 mg, 66%). $[\alpha]^{20}_D$ = +31.6 (*c* 0.4, $CHCl_3$). HRMS (ESI, m/z): calcd for $C_{16}H_{25}O_5^+$ ($[M+H]^+$):297.1797, found: 297.1800. ^1H NMR (500 MHz, $CDCl_3$) δ = 5.09 – 5.03 (m, 1H), 3.68 (qd, J = 9.5, 4.6 Hz, 2H), 3.37 (s, 3H), 3.13 – 3.04 (m, 1H), 2.56 (d, J = 4.7 Hz, 1H), 2.52 (d, J = 4.7 Hz, 1H), 2.48 (dd, J = 14.5, 6.7 Hz, 1H), 2.29 – 2.23 (m, 1H), 2.18 – 2.13 (m, 1H), 1.87 – 1.80 (m, 1H), 1.79 – 1.74 (m, 1H), 1.71 (dd, J = 13.7, 2.7 Hz, 2H), 1.49 – 1.29 (m, 5H), 0.94 (d, J = 6.6 Hz, 3H). ^{13}C NMR (125 MHz, $CDCl_3$) δ = 176.3, 81.2, 78.7, 70.5, 59.25, 55.6, 54.2, 51.1, 46.7, 44.0, 38.0, 36.9, 29.7, 28.2, 20.6, 12.3.

(3S,3aR,7aS,8R,9aR)-3-(methoxymethyl)-5-methyl-3,3a,4,6,7,7a,9,9a-octahydro-2H-spiro[azuleno[6,5-b]furan-8,2'-oxiran]-2-one (38)

Compound 35 (50 mg, 0.17 mmol, 1.0 eq) was dissolved in a sealed tube in toluene (12 mL) and burgess reagent was added (121 mg, 0.51 mmol, 3.0 eq). The reaction mixture was heated at 80°C and stirred for 5h before it was evaporated to dryness. The residue was chromatographed (silica gel) with gradient from 10:1 hexane:EtOAc to 4:1 hexane:EtOAc [R_f=0.55 (hexane:EtOAc= 2:1, UV inactive on TLC, stains green upon *p*-anisaldehyde staining)] to afford pure **Compound 38** as pale yellow amorphous solid (15 mg, 32%). $[\alpha]^{20}_D$ = +23.8 (*c* 0.5, $CHCl_3$). HRMS (ESI, m/z): calcd for: $C_{16}H_{23}O_4^+$ $[M+H]^+$ 279.1591, found:279.1591. ^1H NMR (500 MHz, $CDCl_3$) δ = 4.78 (ddd, J = 10.7, 7.7, 3.3 Hz, 1H), 3.68 (qd, J = 9.4, 4.6 Hz, 2H), 3.36 (s, 3H), 2.97 (s, 1H), 2.69 (d, J = 4.8 Hz, 1H), 2.64 (d, J = 4.8 Hz, 1H), 2.52 (td, J = 5.6, 3.9 Hz, 1H), 2.29 (dd, J = 14.0, 10.5 Hz, 3H), 2.22 – 2.13 (m, 1H), 2.05 (dd, J = 16.4, 11.3 Hz, 1H), 1.92 – 1.88 (m, 1H), 1.71 (ddd, J = 12.8, 8.7, 4.2 Hz, 1H), 1.66 (s, 3H), 1.43 – 1.34 (m, 2H). ^{13}C NMR (125 MHz, $CDCl_3$) δ = 176.3, 81.2, 78.7, 70.5, 59.2, 55.6, 54.2, 51.1, 46.7, 44.0, 38.0, 36.9, 29.7, 28.2, 20.6, 12.3.

Alternatively, a redox neutral treatment of **2** with typical Mukaiyama hydration conditions[17] afforded β- alcohol **36** in a 6:1 ratio with its α-OH diastereoisomer. Subsequent dehydration by Burgess reagent resulted in a retro-ene ring-opening to germacranolide **16**. Therefore, replacement of the traditionally used solvent (ethanol) with benzene[18] led to peroxy-protected guaianolide **37**, which upon treatment with burgess reagent yielded alkenes **39** and **40** in 3:1 ratio.

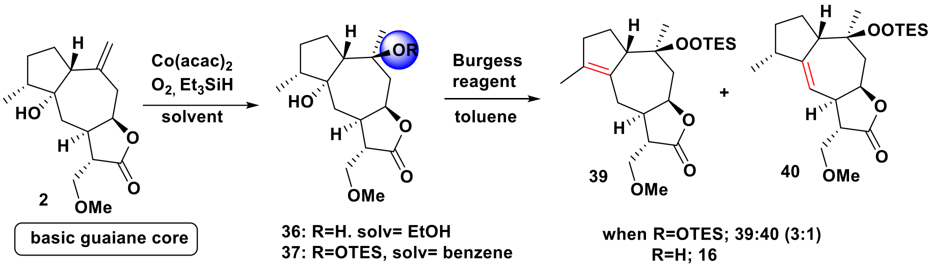

Scheme S18: Mukaiyama hydration of guaianolide **27** followed by dehydration

(3S,3aR,4aR,5R,7aR,8S,9aR)-4a,8-dihydroxy-3-(methoxymethyl)-5,8-dimethyldecahydroazuleno[6,5-b]furan-2(3H)-one (36)

Method A

Complex Co(acac)$_2$ (3.87 mg, 0.015 mmol, 0.2 eq) was added in a microwave vial followed by a solution of **compound 2** (21 mg, 0.075 mmol, 1.0 eq) in 2 mL EtOH. The mixture was bubbled with $O_{2(g)}$ for 10 minutes and then a solution of Et_3SiH (60 µL, 0.375 mmol, 5.0 eq) in 0.6 mL EtOH was added in a span of 6h. After the addition was completed the reaction mixture was stirred at room temperature for 12h before it was quenched with Me_2S and it was evaporated to dryness. The residue was chromatographed (silica gel) with gradient from 10:1 benzene: Et2O to 1:5 benzene:Et_2O to afford pure **compound 36** as a colorless oil (15 mg, 67%)

Method B

Complex Co(acac)$_2$ (7.54 mg, 0.029 mmol, 0.2 eq) was added in a microwave vial followed by a solution of **compound 2** (41 mg, 0.146 mmol, 1.0 eq) in 4 mL benzene. The mixture was bubbled with $O_{2(g)}$ for 10 minutes and then a solution of Et_3SiH (0.11 mL, 0.73 mmol, 5.0 eq) in 1.6 mL benzene was added in a span of 3h. After the addition was completed the reaction mixture was stirred at room temperature for 12h before it was quenched with Me_2S and it was evaporated to dryness. The residue was chromatographed (silica gel) with gradient from 20:1 benzene: Et2O to 1:5 benzene:Et_2O [R_f = 0.15 (benzene: Et_2O = 1:1, UV inactive on TLC, stains green upon *p*-anisaldehyde staining)] to afford pure **compound 36** as a colorless oil (7 mg, 16%). $[\alpha]^{20}_D$ = -36.6 (*c* 0.8, $CHCl_3$). HRMS (ESI, m/z): calcd. for $C_{16}H_{26}O_5Na^+$ ($[M+Na]^+$):321.1672, found:321.1672. ^1H NMR (500 MHz, $CDCl_3$) δ = 4.82 – 4.77 (m, 1H), 3.65 (t, J = 4.2 Hz, 2H), 3.36 (s, 3H), 2.95 – 2.86 (m, 1H), 2.51 – 2.46 (m, 1H), 2.22 (dd, J = 14.4, 6.3 Hz, 1H), 2.10 (t, J = 12.8 Hz, 1H), 2.01 (dd, J = 13.1, 2.6 Hz, 2H), 1.93 – 1.85 (m, 2H), 1.83 – 1.73 (m, 5H), 1.41 (s, 3H), 0.89 (d, J = 6.8 Hz, 3H). ^{13}C NMR (125 MHz, $CDCl_3$) δ = 176.3, 79.6, 78.2, 72.5, 70.2, 61.8, 59.3, 46.8, 45.9, 43.6, 37.4, 35.9, 28.1, 23.4, 22.3, 12.4.

(3S,3aR,4aR,5R,7aS,8S,9aR)-4a-hydroxy-3-(methoxymethyl)-5,8-dimethyl-8-((triethylsilyl)peroxy)-decahydroazuleno[6,5-b]furan-2(3H)-one (37)

Complex Co(acac)$_2$ (3l68 mg, 0.014 mmol, 0.2 eq) was added in a microwave vial followed by a solution of **compound 2** (20 mg, 0.071 mmol, 1.0 eq) in 2 mL benzene. The mixture was bubbled with $O_{2(g)}$ for 10 minutes and then a solution

of Et_3SiH (57 µL, 0.35 mmol, 5.0 eq) in 0.8 mL benzene was added in a span of 3h. After the addition was completed the reaction mixture was stirred at room temperature for 12h before it was quenched with Me_2S and it was evaporated to dryness. The residue was chromatographed (silica gel) with gradient from 20:1 benzene: Et2O to 1:5 benzene:Et_2O [R_f = 0.55 (benzene:Et_2O = 1:1, UV active on TLC, stains brown upon *p*-anisaldehyde staining)] to afford pure **compound 37** as a green amorphous solid (20 mg, 65%). $[\alpha]^{20}_D$ = +14.7 (*c* 0.2, $CHCl_3$). HRMS (ESI, m/z): calcd. for $C_{22}H_{40}O_6SiNa^+$ ($[M+Na]^+$): 451.2486, found: 451.2486. ^1H NMR (500 MHz, $CDCl_3$) δ = 4.81 (ddd, J = 12.0, 8.9, 2.8 Hz, 1H), 3.69 – 3.63 (m, 2H), 3.36 (s, 3H), 2.94 – 2.85 (m, 1H), 2.53 – 2.48 (m, 1H), 2.27 – 2.18 (m, 3H), 2.02 (dd, J = 11.7, 8.3 Hz, 2H), 1.86 (dd, J = 17.3, 11.1 Hz, 1H), 1.78 (dd, J = 16.0, 9.1 Hz, 1H), 1.75 – 1.62 (m, 4H), 1.32 (s, 3H), 0.96 (t, *J* = 8.0 Hz, 9H), 0.88 (d, J = 6.7 Hz, 3H), 0.65 (dd, *J* = 15.4, 7.7 Hz, 6H). ^{13}C NMR (125 MHz, $CDCl_3$) δ = 176.5, 83.8, 80.3, 78.4, 70.3, 59.2, 55.9, 46.8, 43.6, 40.3, 37.2, 36.2, 29.7, 28.3, 22.2, 18.4, 12.4, 6.7, 3.8.

(3S,3aR,7aS,8S,9aR)-3-(methoxymethyl)-5,8-dimethyl-8-((triethylsilyl)peroxy)-3a,4,6,7,7a,8,9,9a-octahydroazuleno[6,5-b]furan-2(3H)-one (39)

Compound 37 (30 mg, 0.1 mmol, 1.0 eq) was dissolved in a sealed tube in 4.4 mL toluene and burgess reagent was added (72 mg, 0.3 mmol, 3.0 eq). The reaction mixture was heated at 80°C and stirred for 40 minutes before it was evaporated to dryness. The residue was chromatographed (silica gel) with gradient from 10:1 hexane:Et_2O to 6:1 hexane:Et_2O [R_f= 0.25 (hexane:Et_2O= 4:1, UV inactive on TLC, stains green upon *p*-anisaldehyde staining)] to afford pure **Compound 39** as a colorless oil (20 mg, 70%). $[\alpha]^{20}_D$ = -45.2 (*c* 0.4, $CHCl_3$). HRMS (ESI, m/z): calcd. for $C_{22}H_{39}O_5Si^+$ ($[M+H]^+$): 411.2561, found: 411.2586. ^1H NMR (500 MHz, $CDCl_3$) δ = 4.63 – 4.56 (m, 1H), 3.69 (dd, J = 9.3, 4.5 Hz, 1H), 3.63 (dd, J = 8.9, 3.1 Hz, 1H), 3.37 (s, 3H), 2.85 (d, J = 9.0 Hz, 1H), 2.56 (dd, J = 13.3, 4.0 Hz, 1H), 2.52 – 2.50 (m, 3H), 2.26 (d, J = 6.9 Hz, 1H), 2.17 (s, 1H), 2.14 (s, 1H), 2.06 (s, 1H), 1.89 – 1.85 (m, 1H), 1.81 – 1.76 (m, 1H), 1.66 (s, 3H), 1.04 (s, 3H), 0.97 (t, J = 7.9 Hz, 9H), 0.66 (q, J = 7.9 Hz, 6H). ^{13}C NMR (125 MHz, $CDCl_3$) δ = 176.7, 136.7, 131.6, 84.8, 79.2, 71.5, 59.3, 59.2, 57.7, 49.06, 41.0, 39.4, 37.7, 29.8, 23.4, 16.1, 13.9, 6.7, 3.8.

(3S,3aR,5R,7aS,8S,9aR)-3-(methoxymethyl)-5,8-dimethyl-8-((triethylsilyl)peroxy)-3a,5,6,7,7a,8,9,9a-octahydra-zuleno[6,5-b]furan-2(3H)-one (40)

Compound 39 (30 mg, 0.1 mmol, 1.0 eq) was dissolved in a sealed tube in 4.4 mL toluene and burgess reagent was added (72 mg, 0.3 mmol, 3.0 eq). The reaction mixture was heated at 80°C and stirred for 40 minutes before it was evaporated to dryness. The residue was chromatographed (silica gel) with gradient from 10:1 hexane:Et_2O to 6:1 hexane:Et_2O [R_f= 0.31 (hexane:Et_2O= 4:1, UV inactive on TLC, stains brownish upon *p*-anisaldehyde staining)] to afford pure **Compound 40** as a pale yellow oil(7 mg, 27%). $[\alpha]^{20}_D$ = +15.8 (*c* 0.2, $CHCl_3$). HRMS (ESI, m/z): calcd. for $C_{22}H_{39}O_5Si^+$ ($[M+H]^+$): 411.2561, found: 411.2572. ^1H NMR (500 MHz, $CDCl_3$) δ = 5.43 (d, *J* = 7.3 Hz, 1H), 4.60 (ddd, *J* = 12.2, 7.0, 4.8 Hz, 1H), 3.75 (dd, *J* = 9.9, 3.6 Hz, 1H), 3.63 (dd, *J* = 9.8, 3.5 Hz, 1H), 3.38 (s, 3H), 3.34 – 3.27 (m, 1H), 2.90 (t, *J* = 7.3 Hz, 1H), 2.62 (ddd, *J* = 13.1, 8.3, 4.1 Hz, 2H), 2.30 (dd, *J* = 14.6, 10.1 Hz, 1H), 2.09 (t, *J* = 13.0 Hz, 1H), 1.84 – 1.77 (m, 1H), 1.74 – 1.67 (m, 3H), 1.08 (s, 3H), 1.01 (d, *J* = 7.1 Hz, 3H), 0.97 (t, *J* = 7.9 Hz, 9H), 0.66 (q, *J* = 7.9 Hz, 6H). ^{13}C NMR (125 MHz, $CDCl_3$) δ = 176.0, 151.0, 114.3, 82.2, 68.2, 59.3, 51.5, 45.2, 42.5, 41.3, 40.0, 32.0, 29.7, 25.9, 21.1, 16.2, 6.3, 3.8.

6.6.4 Deprotection of the lactone moiety.

The α-methylene-γ-butyrolactone of the natural sesquiterpenoids is a Michael acceptor and is attributed several of their biological activities[19]. Hence, we chose to attempt the removal of the methoxy protection group, indicatively, in products **20** and **24** to unmask the bioactive moiety. In both cases TBAF, as previously reported in the literature[20], worked exceptionally despite the presence of labile functional groups such as an epoxide and a tertiary alcohol.

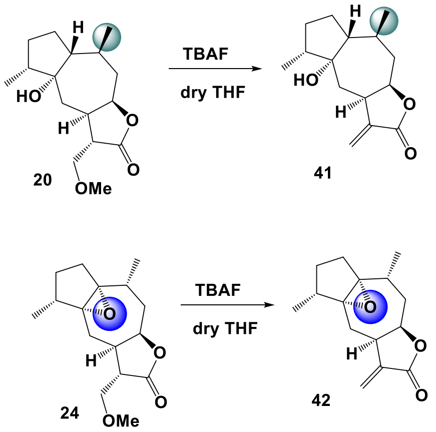

Scheme S19: Indicative deprotection to reveal the α-methylene-γ-butenolide

(3aR,4aR,5R,7aR,8S,9aR)-4a-hydroxy-5,8-dimethyl-3-methylenedecahydroazuleno[6,5-b]furan-2(3H)-one (41)

To a vial **compound 20** (5 mg, 0.017 mmol, 1.0 eq) was dissolved in dry THF (0.5 mL) and TBAF (70 μL, 0.071 mmol, 1M in THF, 4.0 eq) was added dropwise at 0°C. After the addition, the reaction mixture was allowed to cool at room temperature and stirred overnight before it was quenched with HCl_{aq} 0.5 N (0.5 mL). After separation, the aqueous layer was extracted three times with EtOAc (3x 1 mL). The combined organic extracts were dried over Na_2SO_4, filtered, and evaporated under reduced pressure. The residue was chromatographed (silica gel) with gradient from 15:1 n-hexane:EtOAc to 1:1 n-hexabe:EtOAc [R_f= 0.33 (n-hexabe:EtOAc= 1.5:1, UV inactive on TLC, stains green upon *p*-anisaldehyde staining)] to afford pure **Compound 41** as an amorphous white solid (2.5 mg, 59%). $[\alpha]^{20}_D$ = -23.7 (c 0.2, $CHCl_3$) HRMS (ESI, m/z): calcd for $C_{15}H_{22}O_3Na^+$ ($[M+Na]^+$): 273.1461 , found: 273.1461. ^{1}H NMR (500 MHz, $CDCl_3$) δ= 6.24 (d, J = 3.5 Hz, 1H), 5.49 (d, J = 3.1 Hz, 1H), 4.92 – 4.82 (m, 1H), 3.42 (d, J = 4.2 Hz, 1H), 2.35 (dd, J = 14.1, 6.6 Hz, 1H), 1.84 (dt, J = 19.9, 9.3 Hz, 4H), 1.65 – 1.59 (m, 1H), 1.52 – 1.39 (m, 2H), 1.32 (dd, J = 16.6, 9.0 Hz, 3H), 0.95 (dd, J = 6.6, 1.8 Hz, 6H).^{13}C NMR (125 MHz, $CDCl_3$) δ = 170.2, 140.2, 120.8, 82.1, 80.2, 77.2, 77.0, 76.8, 59.2, 43.3, 40.3, 38.0, 35.9, 31.6, 28.6, 27.1, 21.7, 12.4.

(3aR,4aR,5R,7aR,8R,9aR)-5,8-dimethyl-3-methylenehexahydro-4H,5H-4a,7a-epoxyazuleno[6,5-b]furan-2(3H)-one (42)

To a vial **compound 24** (9 mg, 0.032 mmol, 1.0 eq) was dissolved in dry THF (1 mL) and TBAF (128 μL, 0.128 mmol, 1M in THF, 4.0 eq) was added dropwise at 0°C. After the addition, the reaction mixture was allowed to cool at room temperature and stirred for 3h before it was quenched with HCl_{aq} 0.5 N (1 mL). After separation, the aqueous layer was extracted three times with EtOAc (3x 2 mL). The combined organic extracts were dried over Na_2SO_4, filtered, and evaporated under reduced pressure. The residue was chromatographed (silica gel) with gradient from 12:1 n-

hexane:EtOAc to 6:1 n-hexabe:EtOAc [R_f= 0.45 (n-hexabe:EtOAc= 2:1, UV inactive on TLC, stains green upon *p*-anisaldehyde staining)] to afford pure **Compound 42** as an amorphous white solid (5 mg, 63%).$[\alpha]^{20}_{D}$ = +41.4 (*c* 0.3, $CHCl_3$). HRMS (ESI, m/z): calcd for: $C_{15}H_{21}O_3^+$ $[M+H]^+$ 249.1485, found: 249.1482. ^{1}H NMR (500 MHz, $CDCl_3$) δ= 6.31 (d, J = 3.0 Hz, 1H), 5.60 (d, J = 2.6 Hz, 1H), 4.84 – 4.78 (m, 1H), 3.35 (d, J = 3.8 Hz, 1H), 2.31 – 2.19 (m, 3H), 2.02 (dd, J = 13.8, 8.3 Hz, 1H), 1.94 – 1.86 (m, 2H), 1.81 – 1.76 (m, 1H), 1.65 – 1.60 (m, 1H), 1.49 – 1.41 (m, 1H), 1.15 (d, J = 7.1 Hz, 3H), 1.03 (d, J = 6.7 Hz, 3H), 0.98 – 0.91 (m, 1H). ^{13}C NMR (125 MHz, $CDCl_3$) δ = 170.09, 139.1, 122.2, 77.4, 72.8, 72.6, 38.7, 37.6, 33.3, 31.1, 30.2, 28.0, 27.8, 16.7, 13.6.

7. Spectra of substrates and products

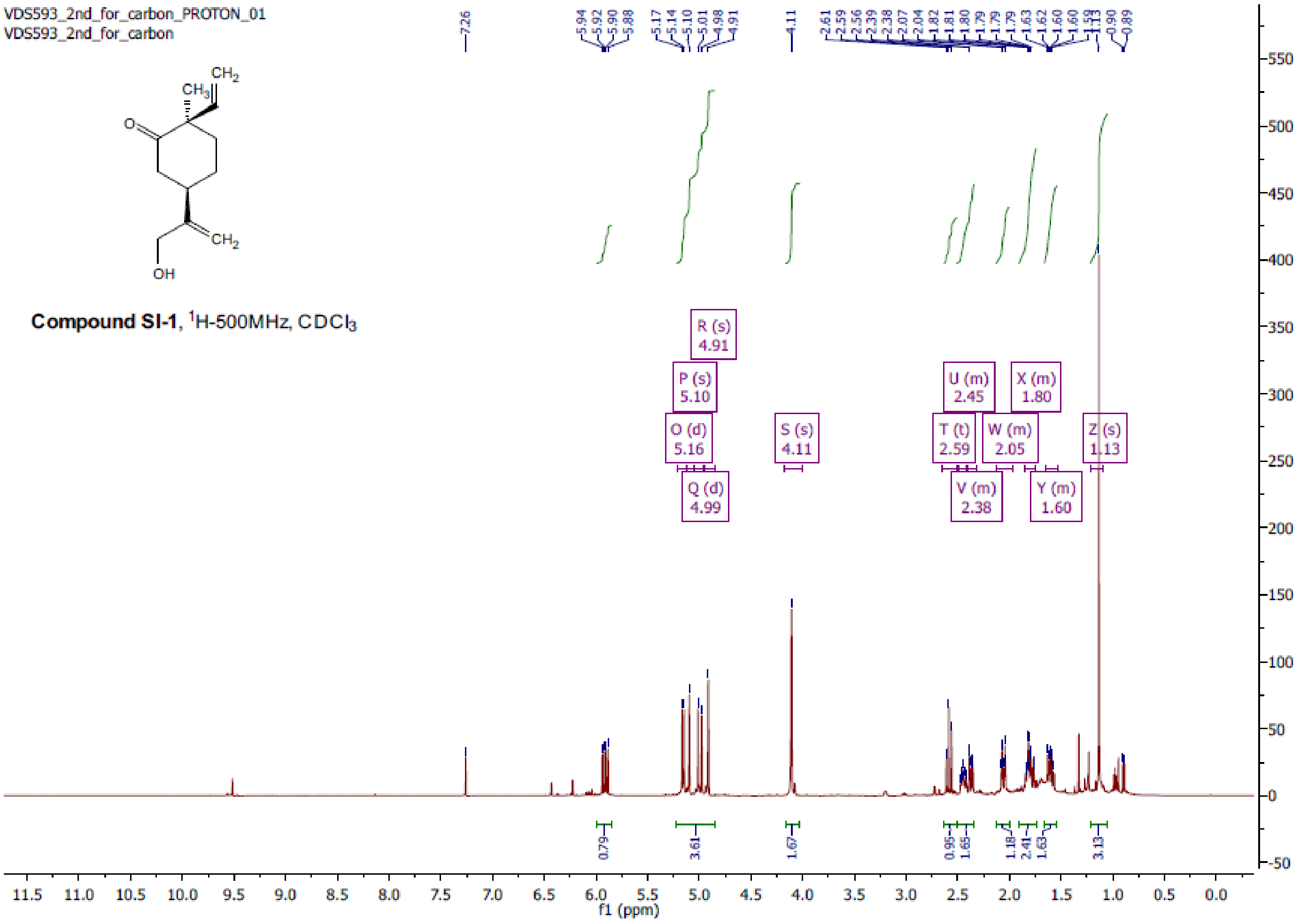

Compound SI-1, ^{1}H-500MHz, $CDCl_3$

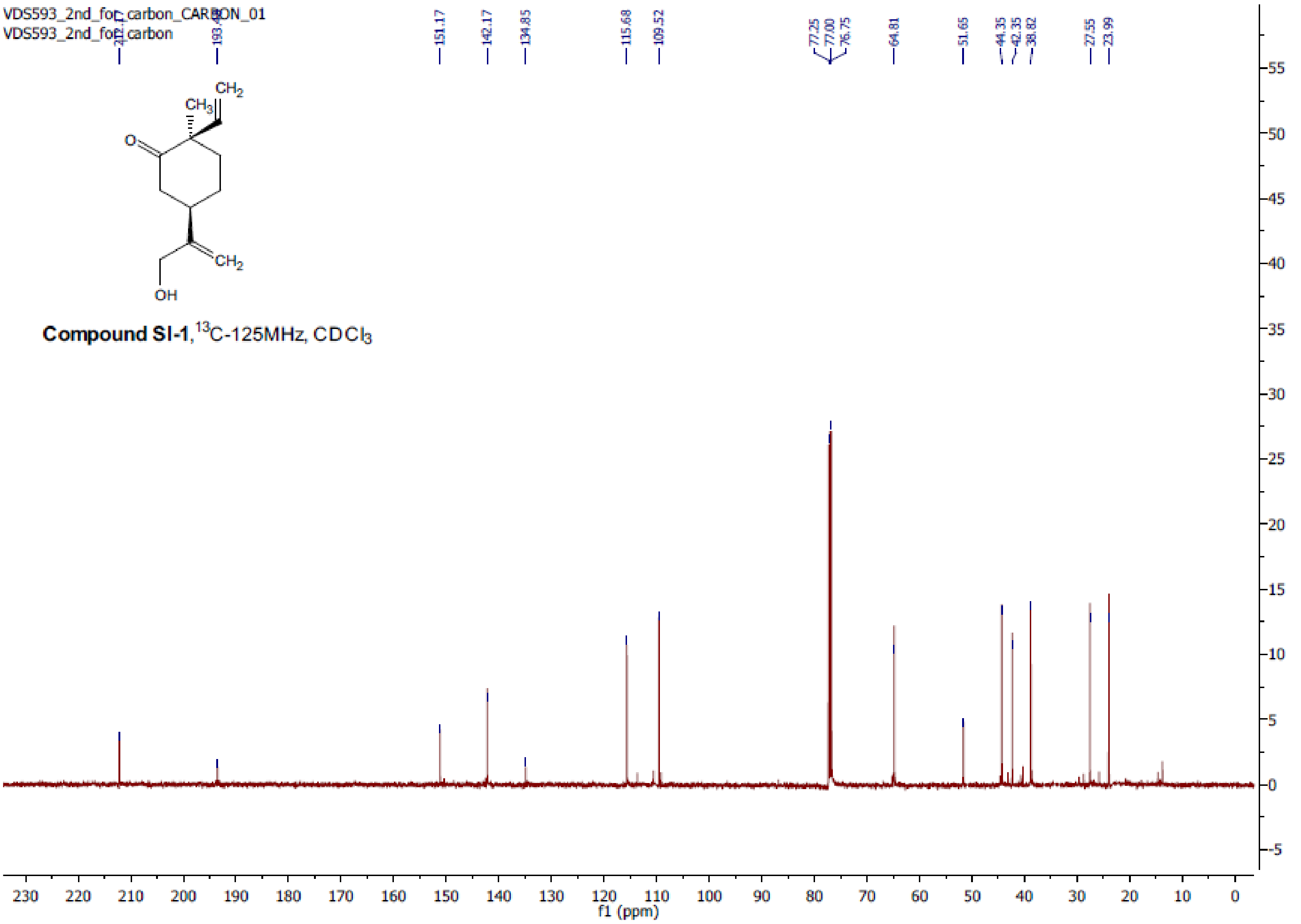

Compound SI-1, ^{13}C-125MHz, $CDCl_3$

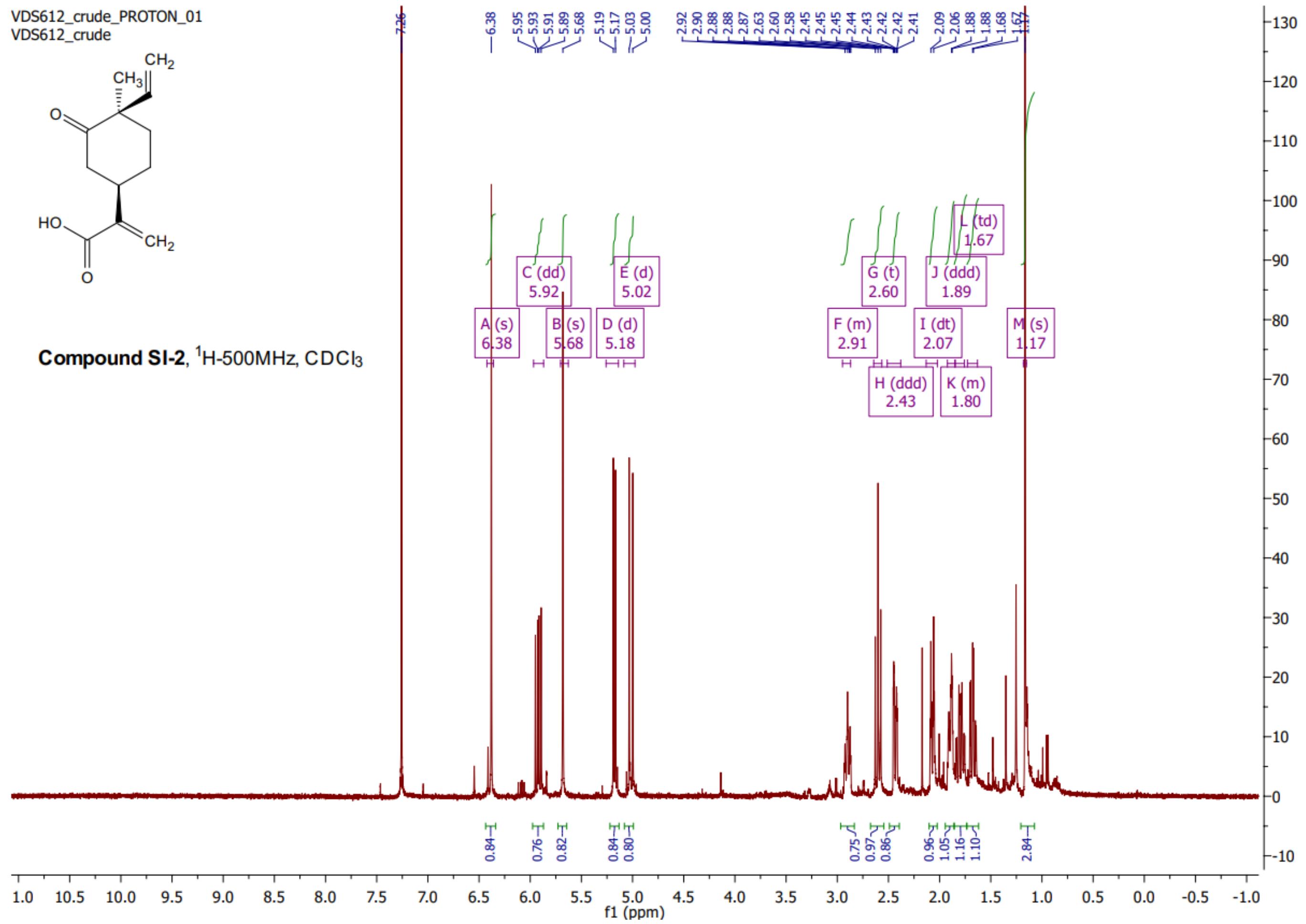
VDS612_crude_PROTON_01
VDS612_crude
Compound SI-2, 1H-500MHz, CDCl3
f1 (ppm)

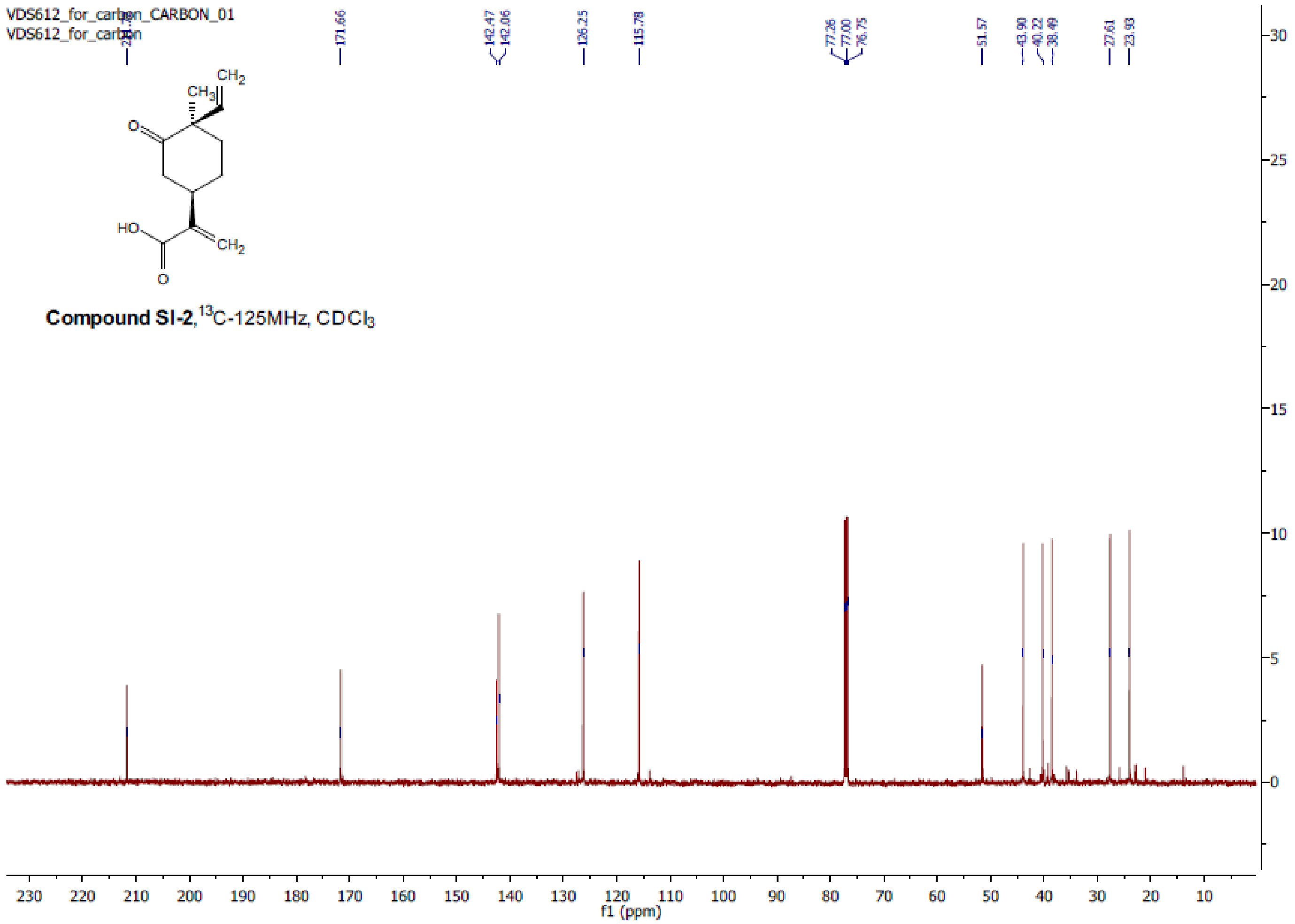
VDS612_for_carbon_CARBON_01
VDS612_for_carbon
Compound SI-2, 13C-125MHz, CDCl3
f1 (ppm)

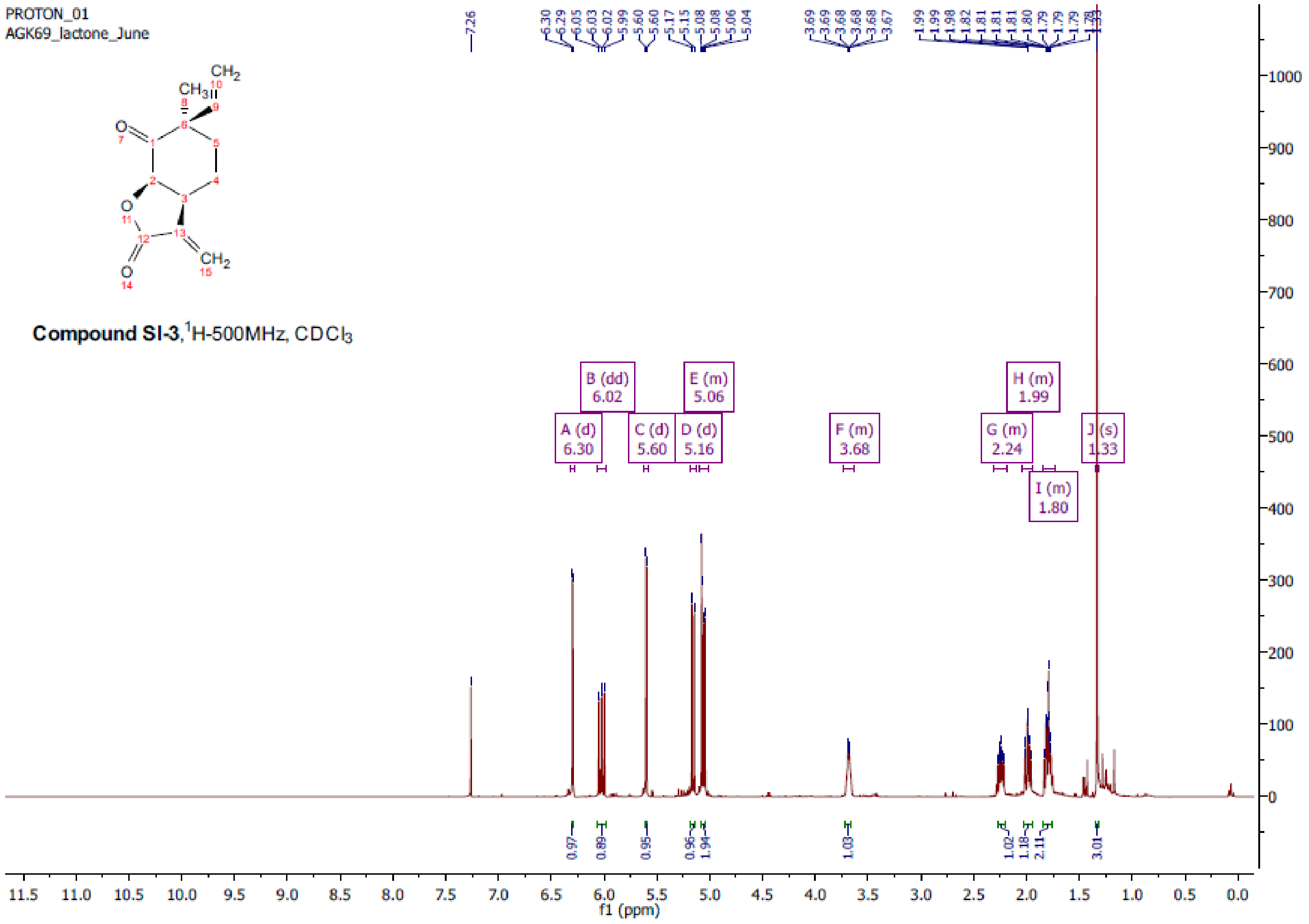
PROTON_01
AGK69_lactone_June
Compound SI-3, 1H-500MHz, CDCl3
7.26
6.30 6.29 6.05 6.03 6.02 5.99 5.60 5.60 5.17 5.15 5.08 5.08 5.06 5.04
3.69 3.69 3.68 3.68 3.68 3.67
1.99 1.99 1.98 1.82 1.81 1.81 1.80 1.79 1.79 1.79 1.78 1.33
A (d) 6.30
B (dd) 6.02
C (d) 5.60
D (d) 5.16
E (m) 5.06
F (m) 3.68
G (m) 2.24
H (m) 1.99
I (m) 1.80
J (s) 1.33
0.97 0.89 0.95 0.96 1.94 1.03 1.02 1.18 2.11 3.01
f1 (ppm)

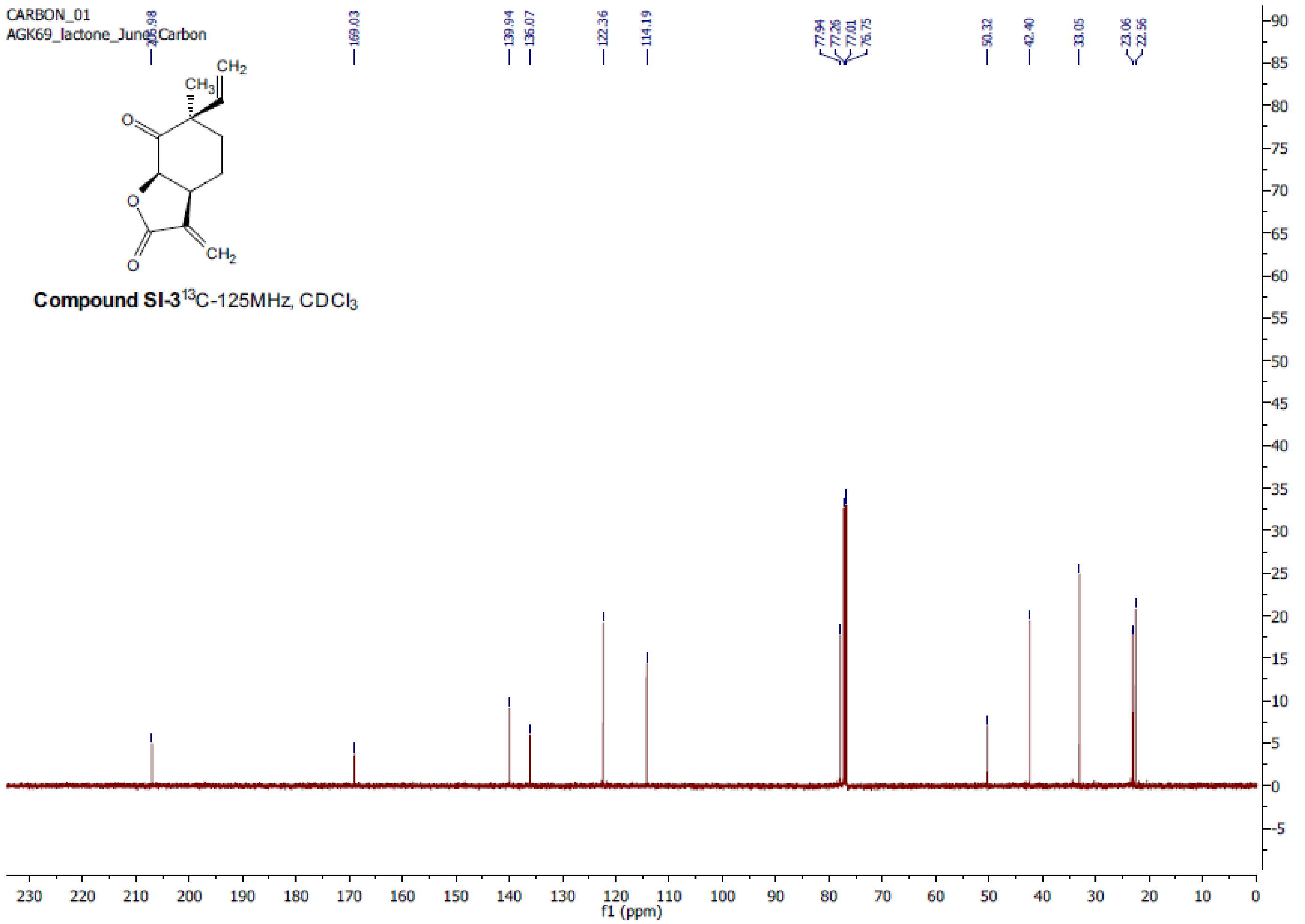
CARBON_01
AGK69_lactone_June Carbon
Compound SI-3 13C-125MHz, CDCl3
206.98 169.03 139.94 136.07 122.36 114.19 77.94 77.26 77.01 76.75 50.32 42.40 33.05 23.06 22.56
f1 (ppm)

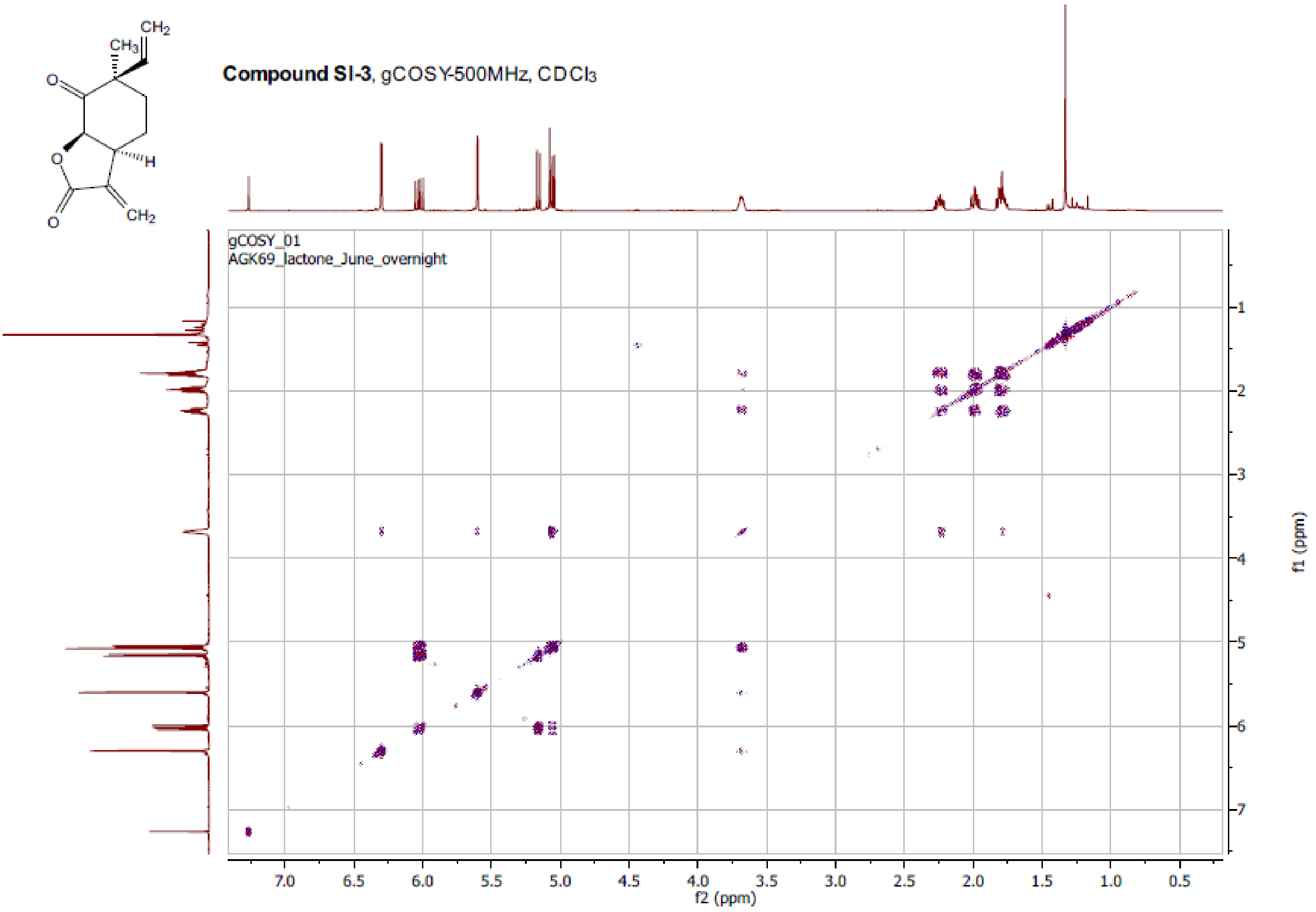
Compound SI-3, gCOSY-500MHz, CDCl3
gCOSY_01
AGK69_lactone_June_overnight
f2 (ppm)
f1 (ppm)

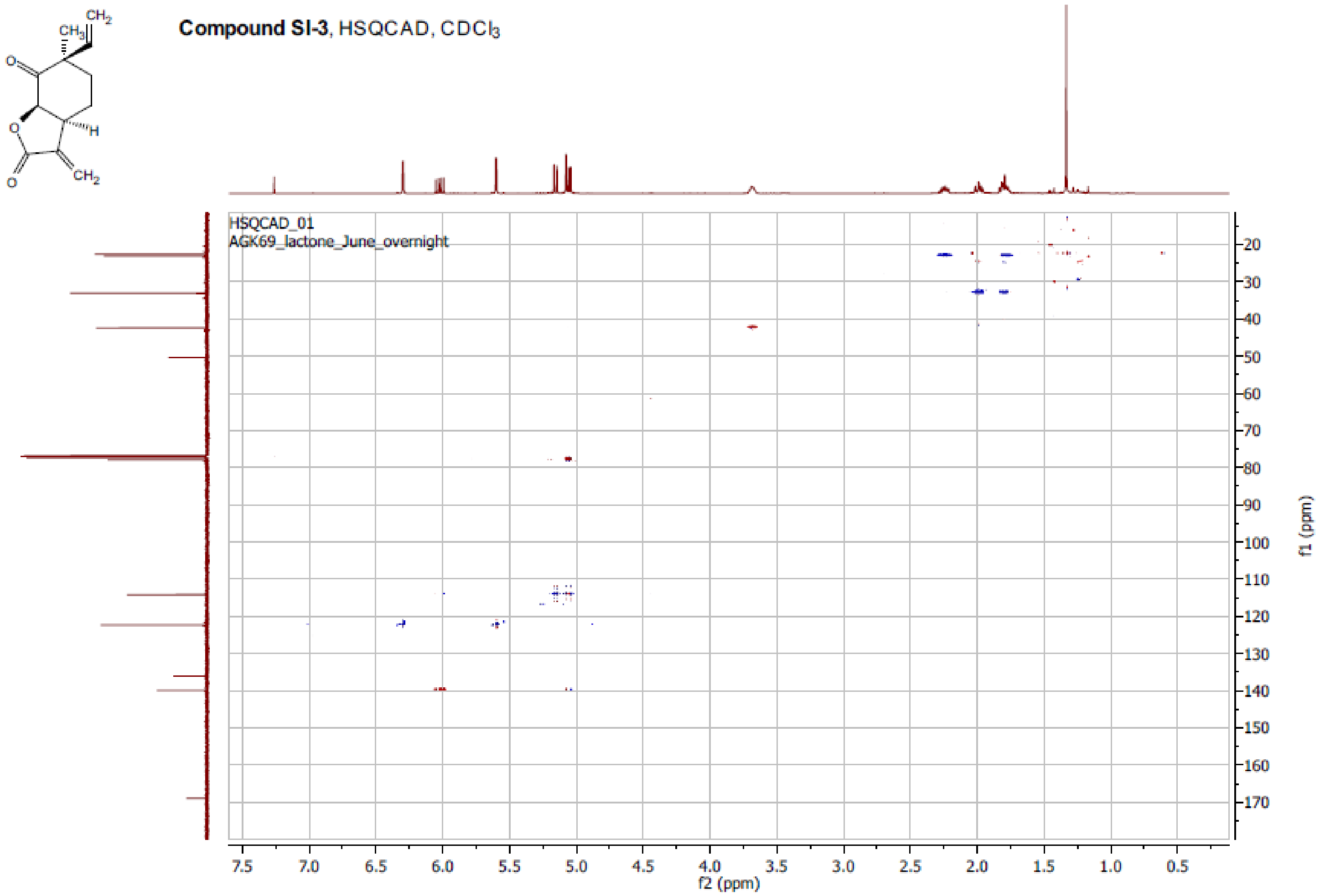
Compound SI-3, HSQCAD, CDCl3
HSQCAD_01
AGK69_lactone_June_overnight
f2 (ppm)
f1 (ppm)

Compound SI-3, NOESY-500MHz, $CDCl_3$

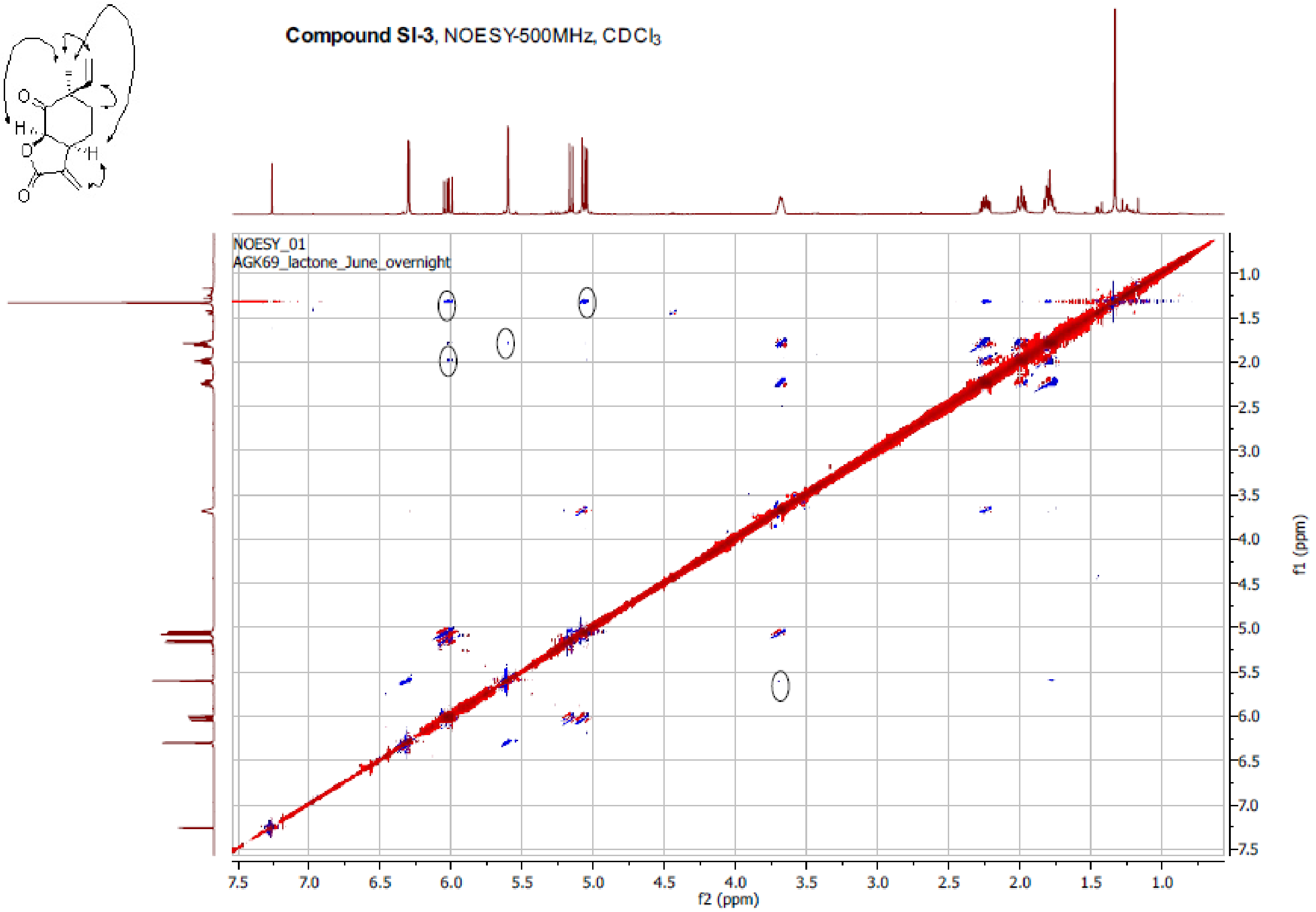

Compound SI-6, ^{1}H-500MHz, $CDCl_3$

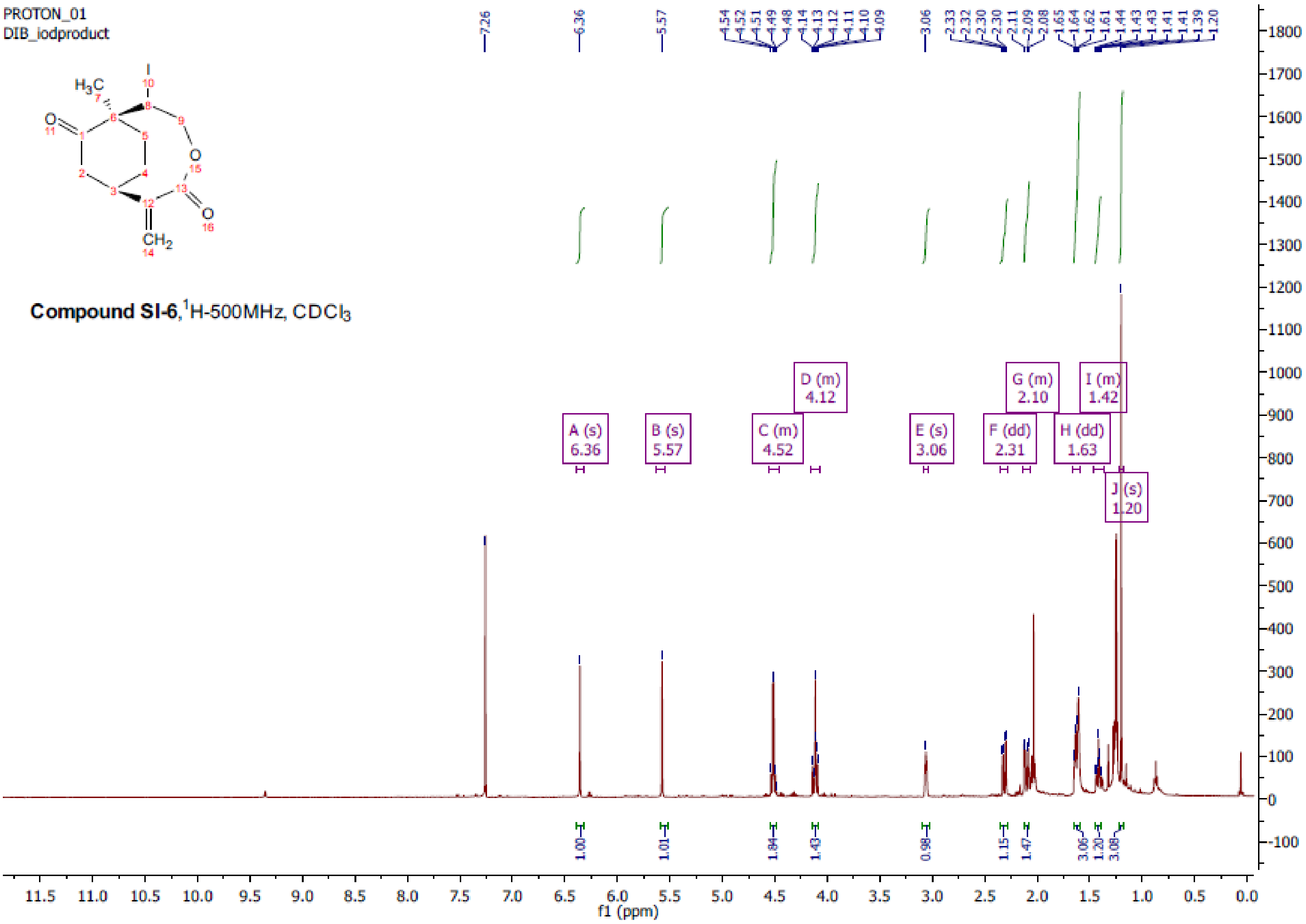

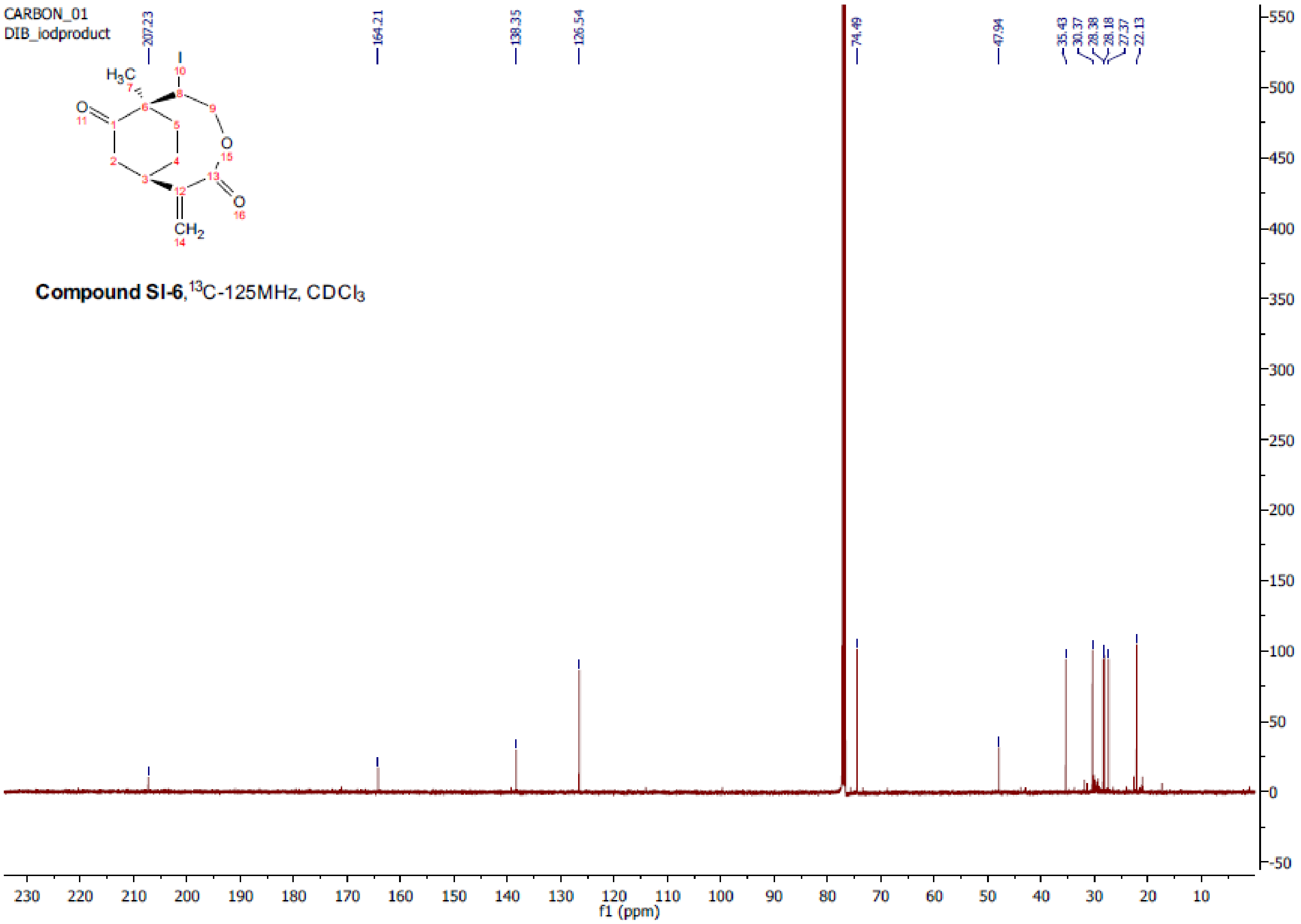

Compound SI-6, ^{13}C-125MHz, $CDCl_3$

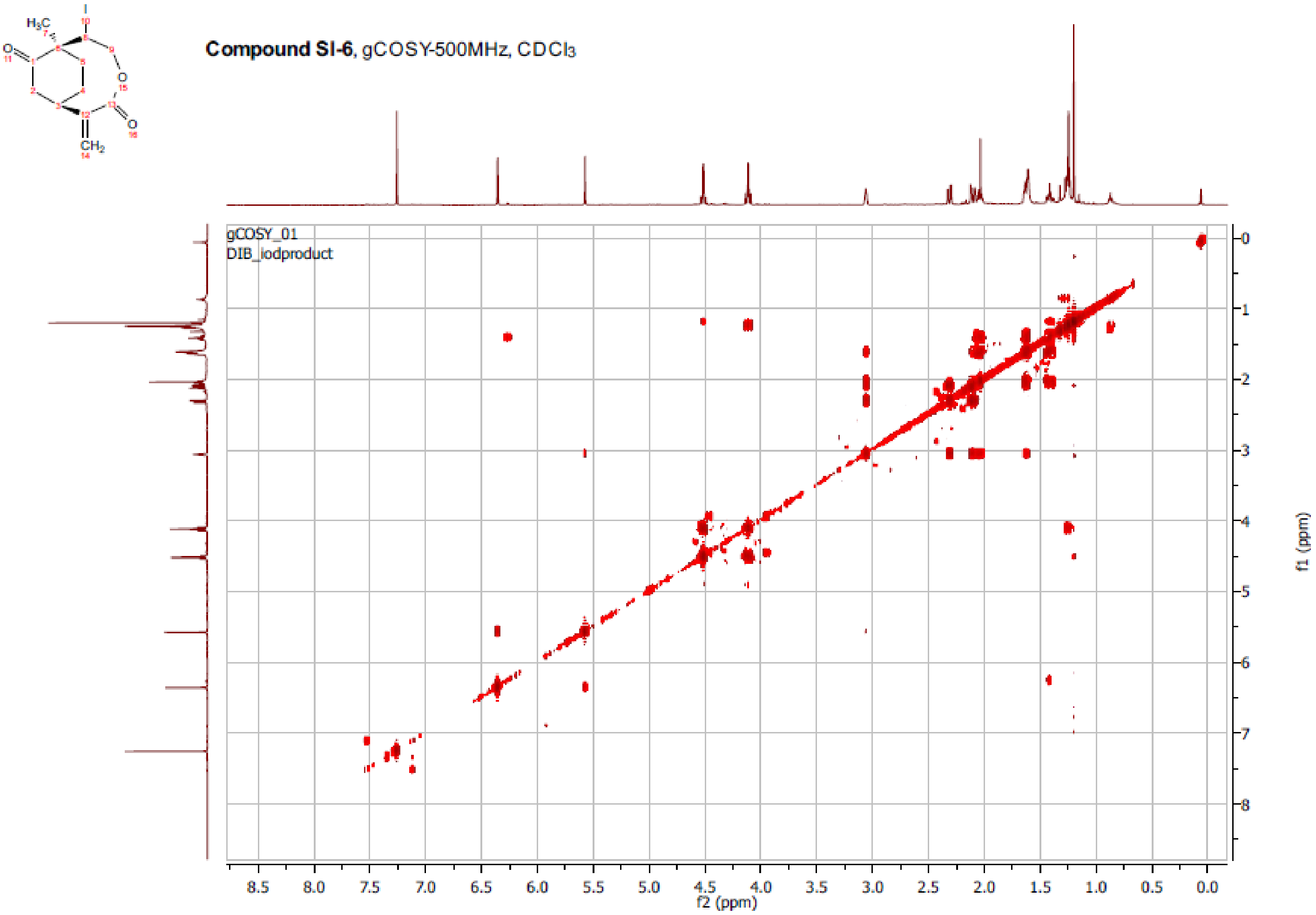

Compound SI-6, gCOSY-500MHz, $CDCl_3$

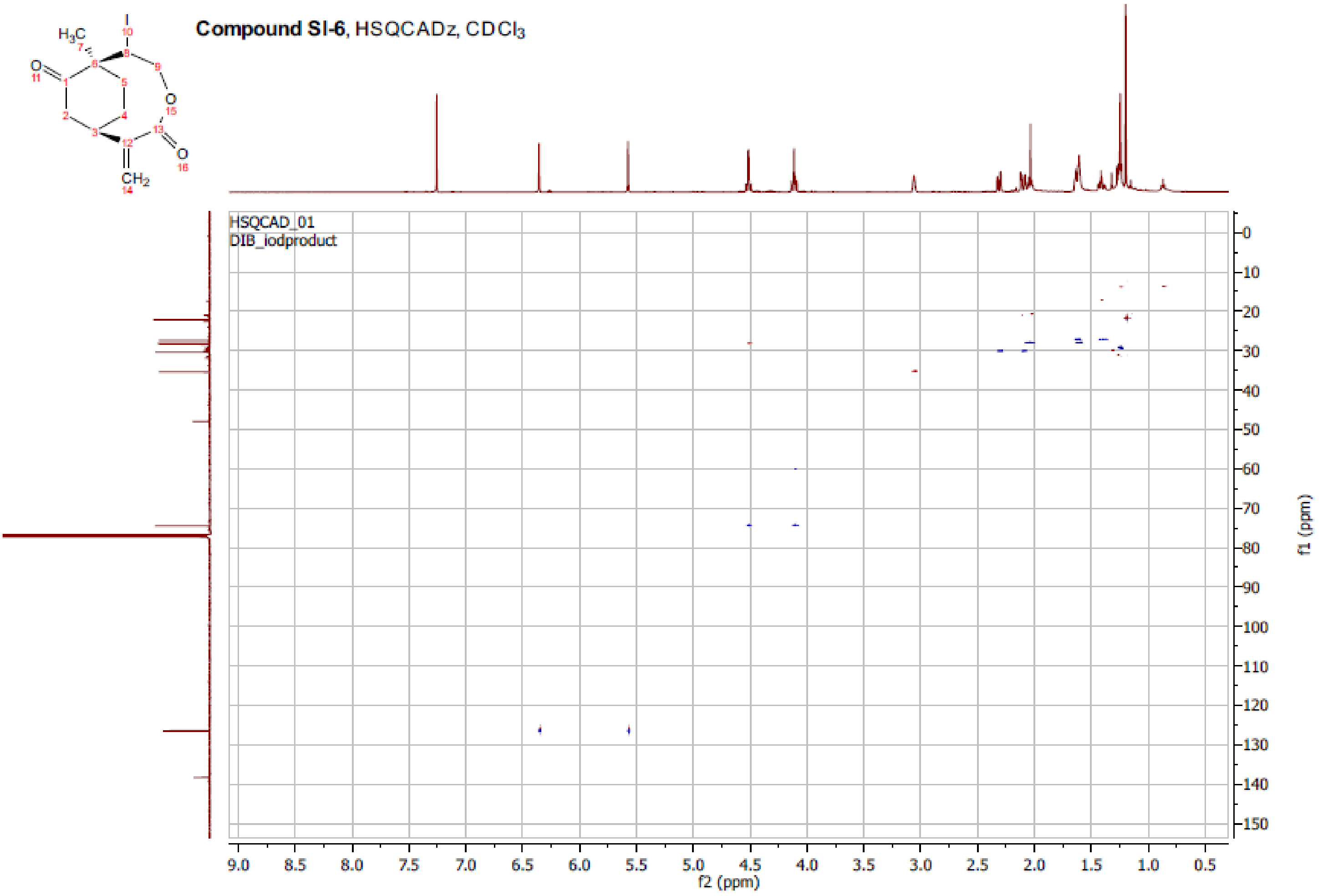
Compound SI-6, HSQCADz, CDCl3
HSQCAD_01
DIB_iodproduct
f2 (ppm)
f1 (ppm)

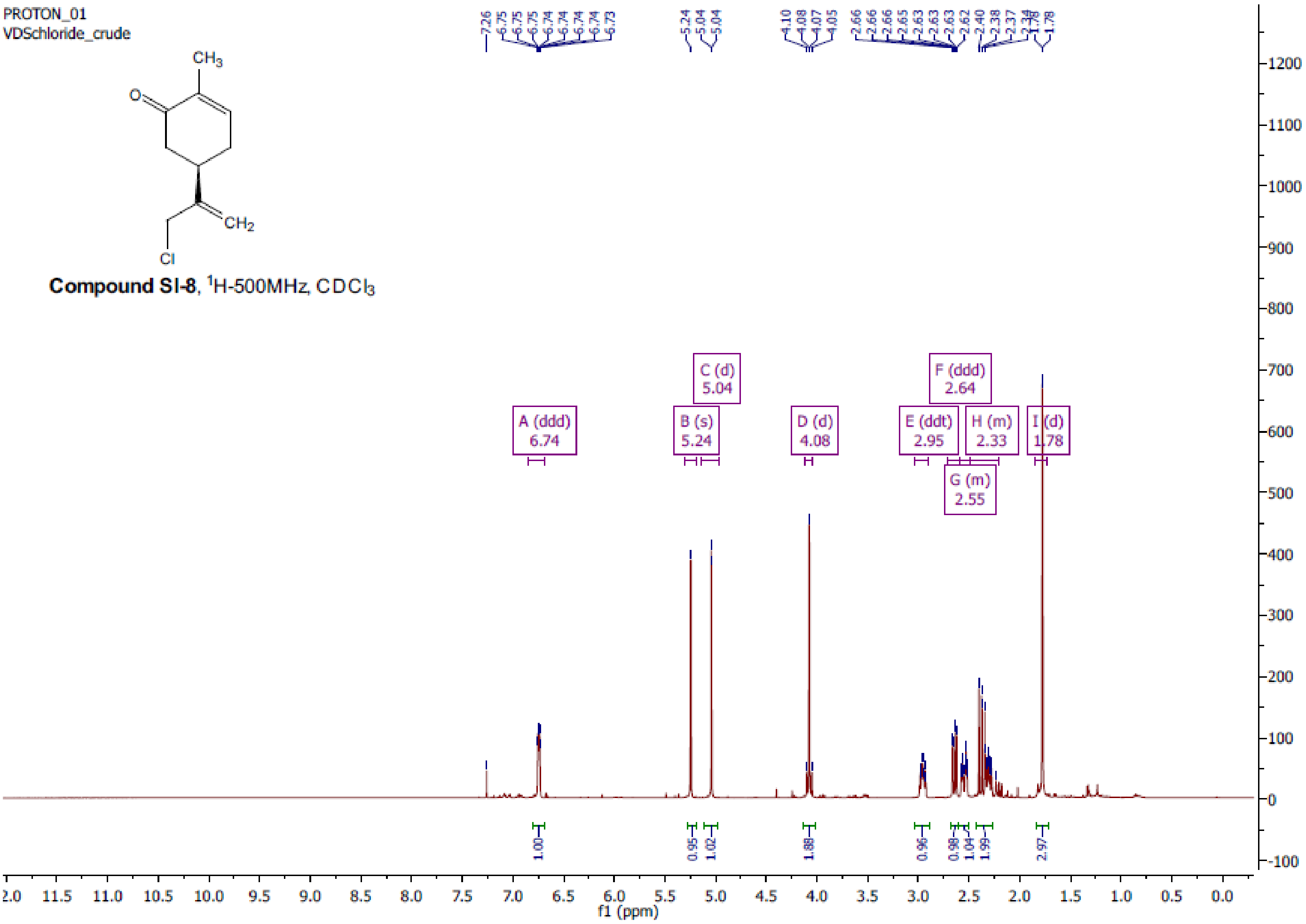
PROTON_01
VDSchloride_crude
Compound SI-8, 1H-500MHz, CDCl3
A (ddd) 6.74
B (s) 5.24
C (d) 5.04
D (d) 4.08
E (ddt) 2.95
F (ddd) 2.64
G (m) 2.55
H (m) 2.33
I (d) 1.78
f1 (ppm)

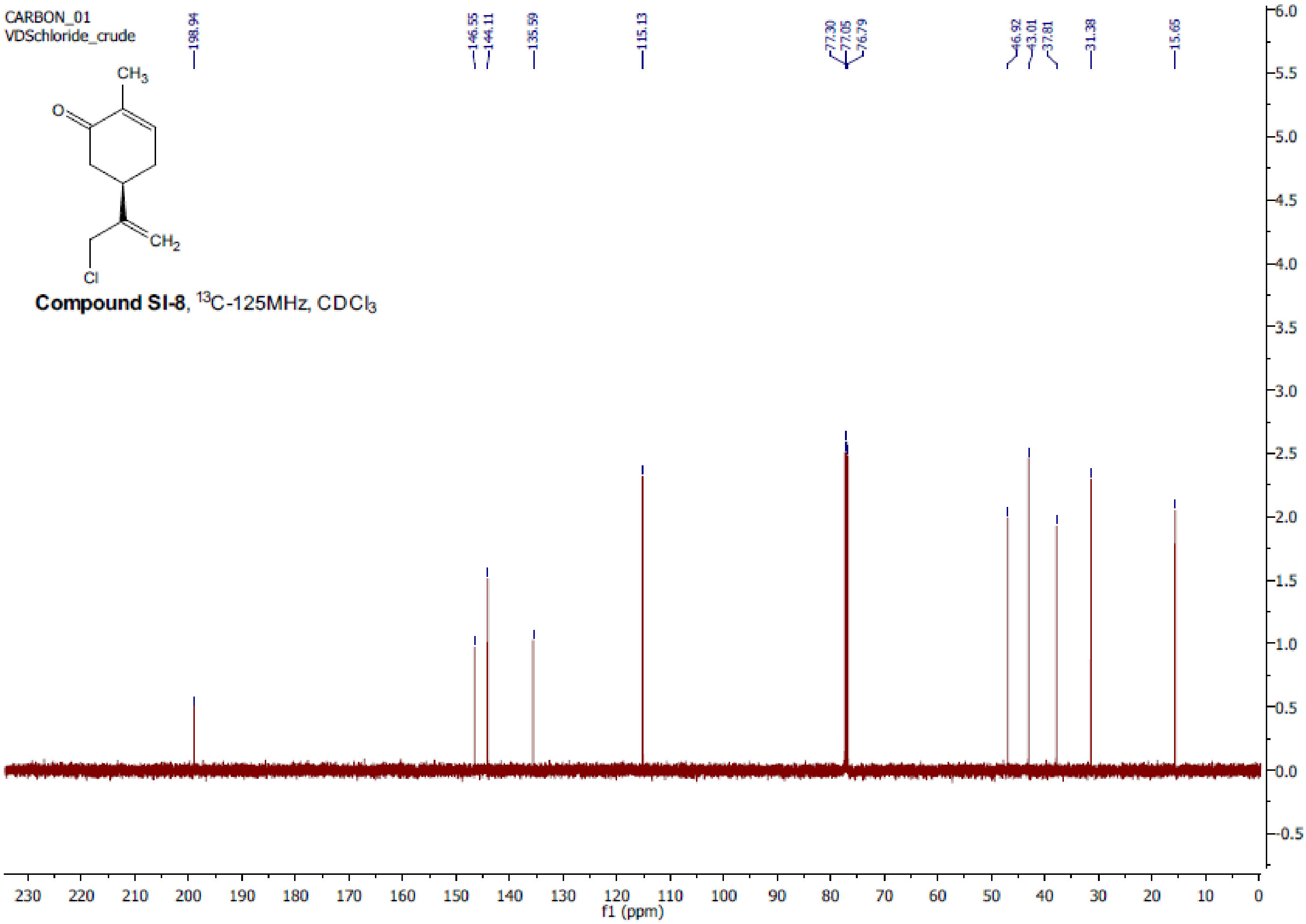
CARBON_01
VDSchloride_crude
198.94
146.55
144.11
135.59
115.13
77.30
77.05
76.79
46.92
43.01
37.81
31.38
15.65
CH3
O
CH2
Cl
Compound SI-8, 13C-125MHz, CDCl3
f1 (ppm)

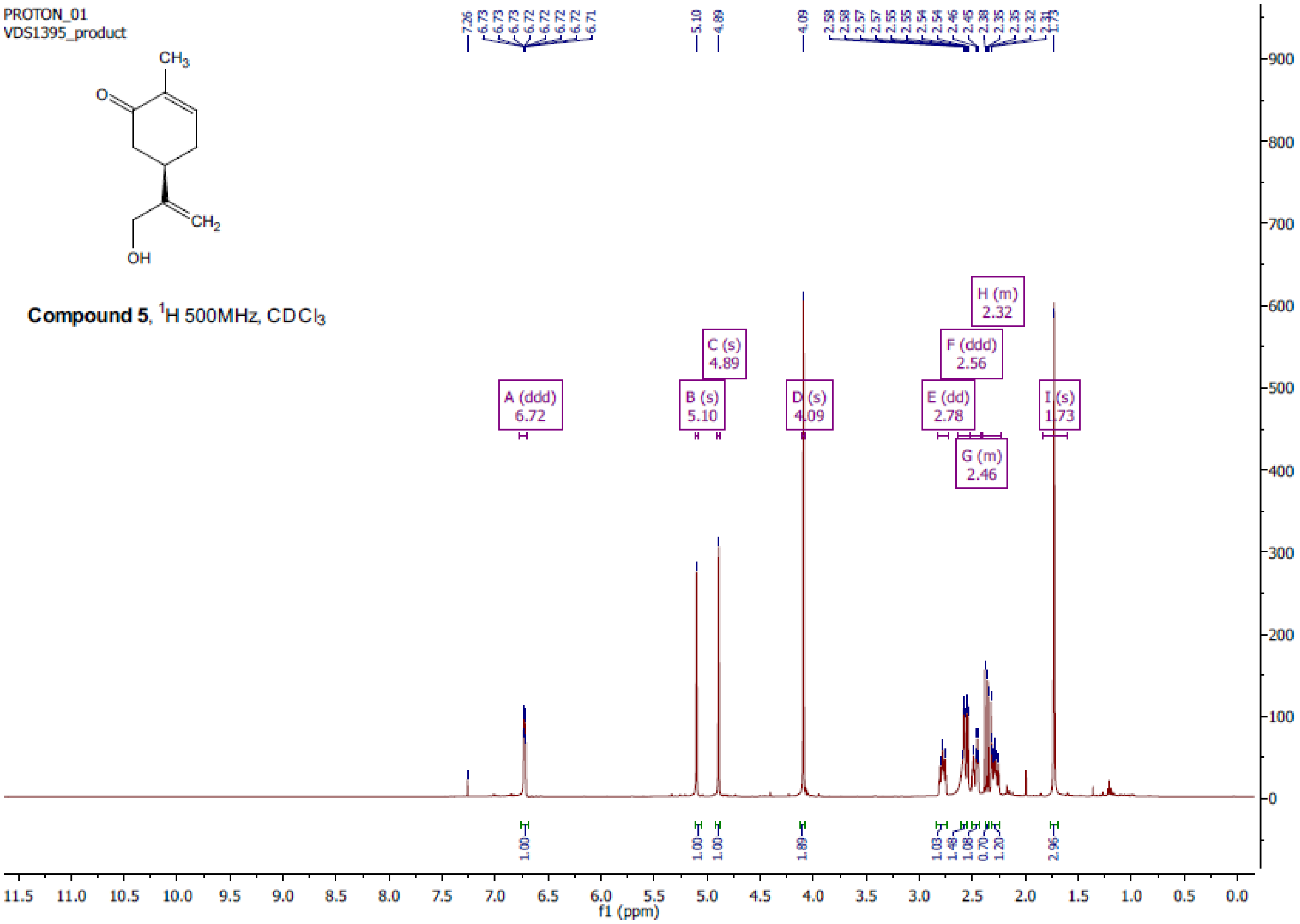
PROTON_01
VDS1395_product
7.26
6.73
6.73
6.72
6.72
6.72
6.72
6.71
5.10
4.89
4.09
2.58
2.58
2.57
2.55
2.55
2.54
2.54
2.46
2.45
2.38
2.35
2.35
2.32
2.31
1.73
CH3
O
CH2
OH
Compound 5, 1H 500MHz, CDCl3
A (ddd) 6.72
B (s) 5.10
C (s) 4.89
D (s) 4.09
E (dd) 2.78
F (ddd) 2.56
G (m) 2.46
H (m) 2.32
I (s) 1.73
1.00
1.00
1.00
1.89
1.03
1.48
1.08
0.70
1.20
2.96
f1 (ppm)

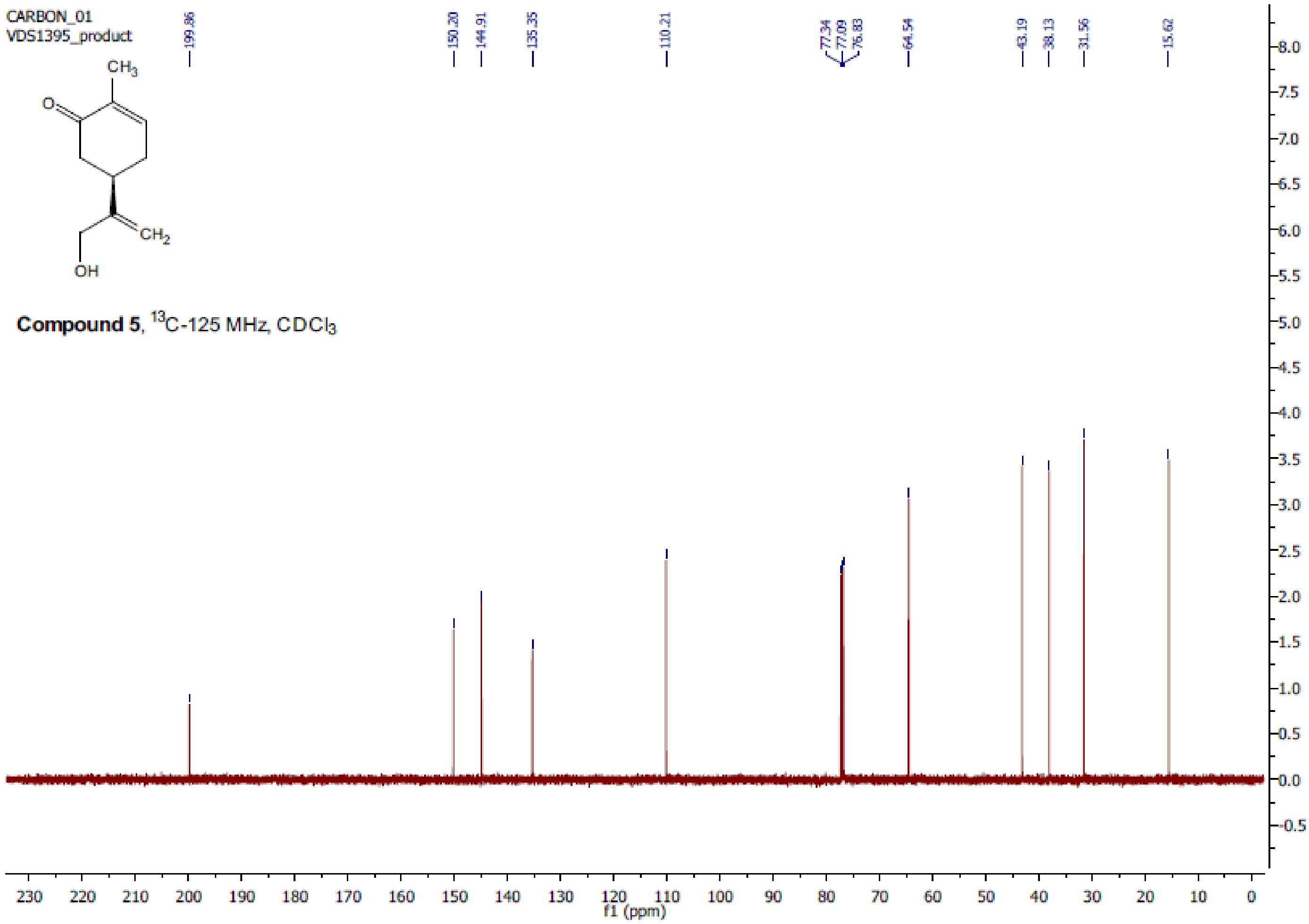

Compound 5, ^{13}C-125 MHz, $CDCl_3$

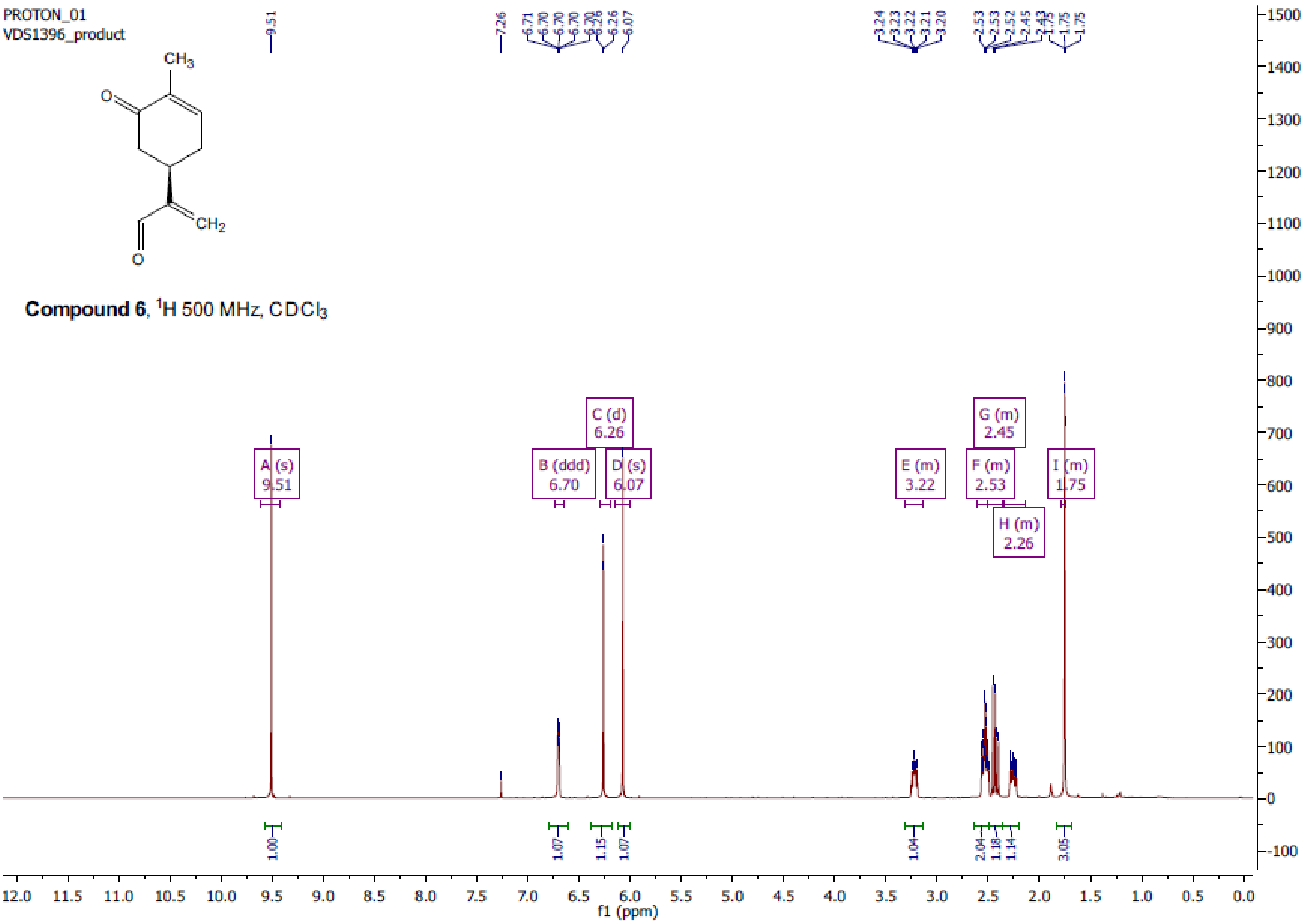

Compound 6, ^{1}H 500 MHz, $CDCl_3$

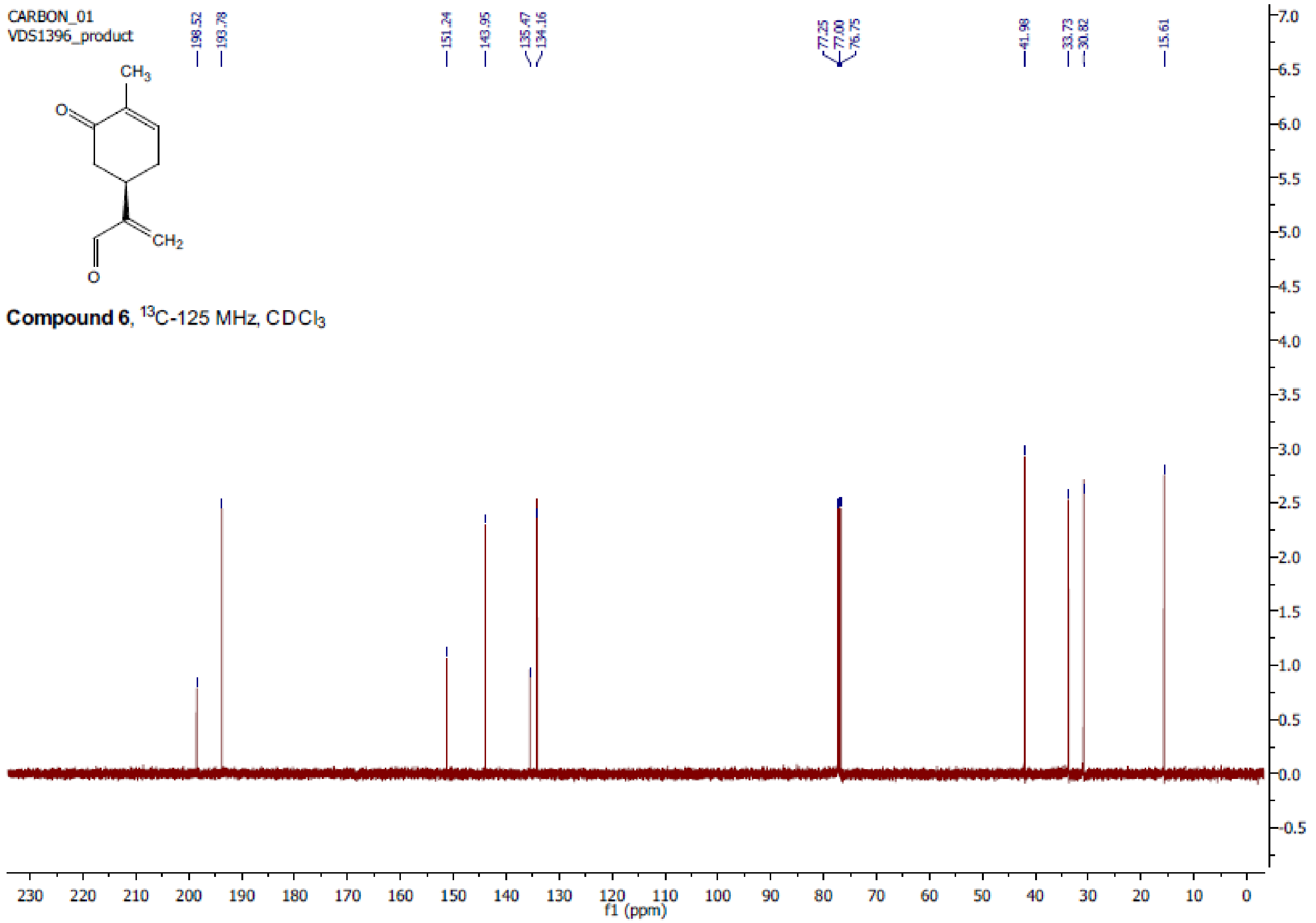

Compound 6, ^{13}C-125 MHz, $CDCl_3$

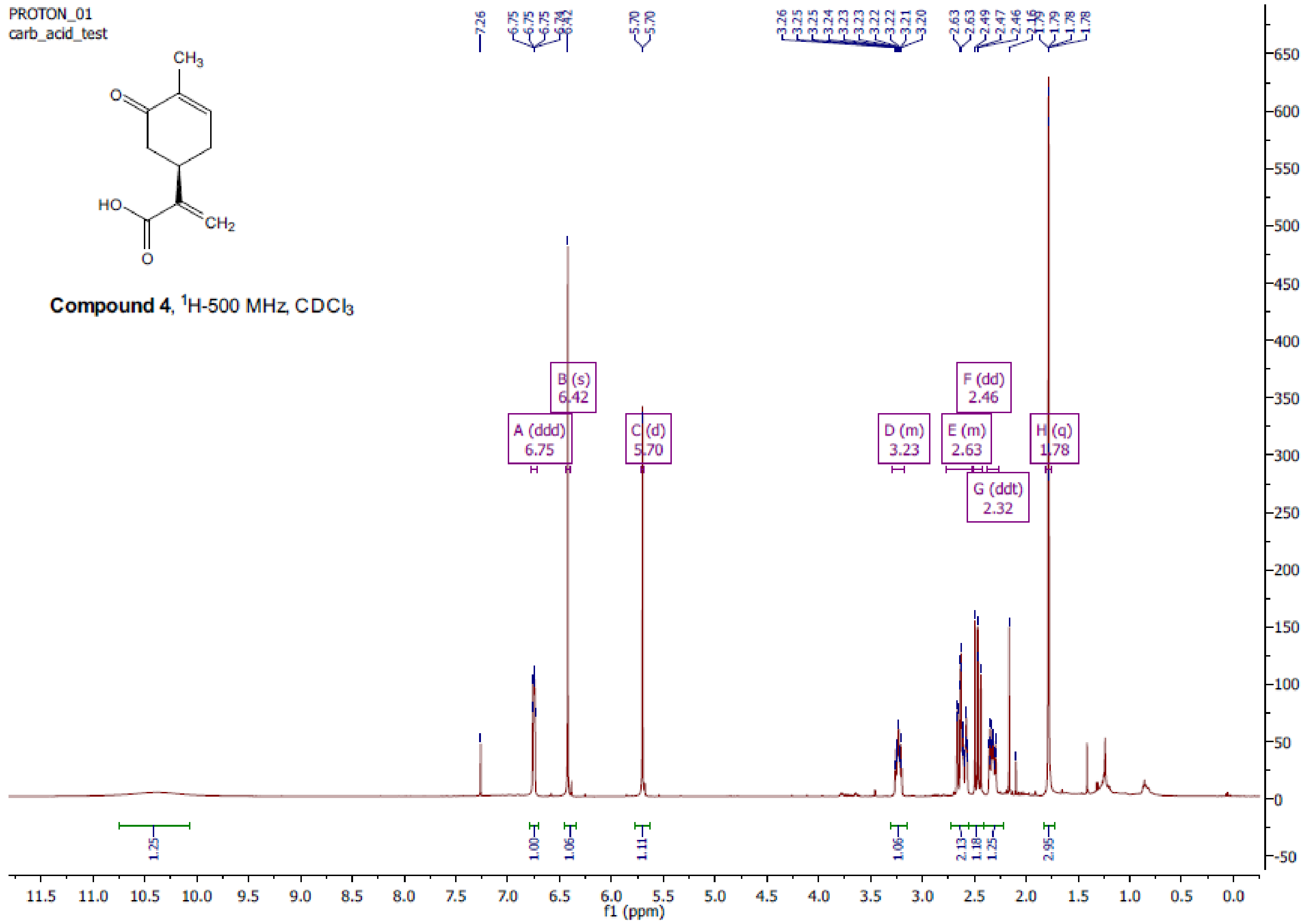

Compound 4, ^{1}H-500 MHz, $CDCl_3$

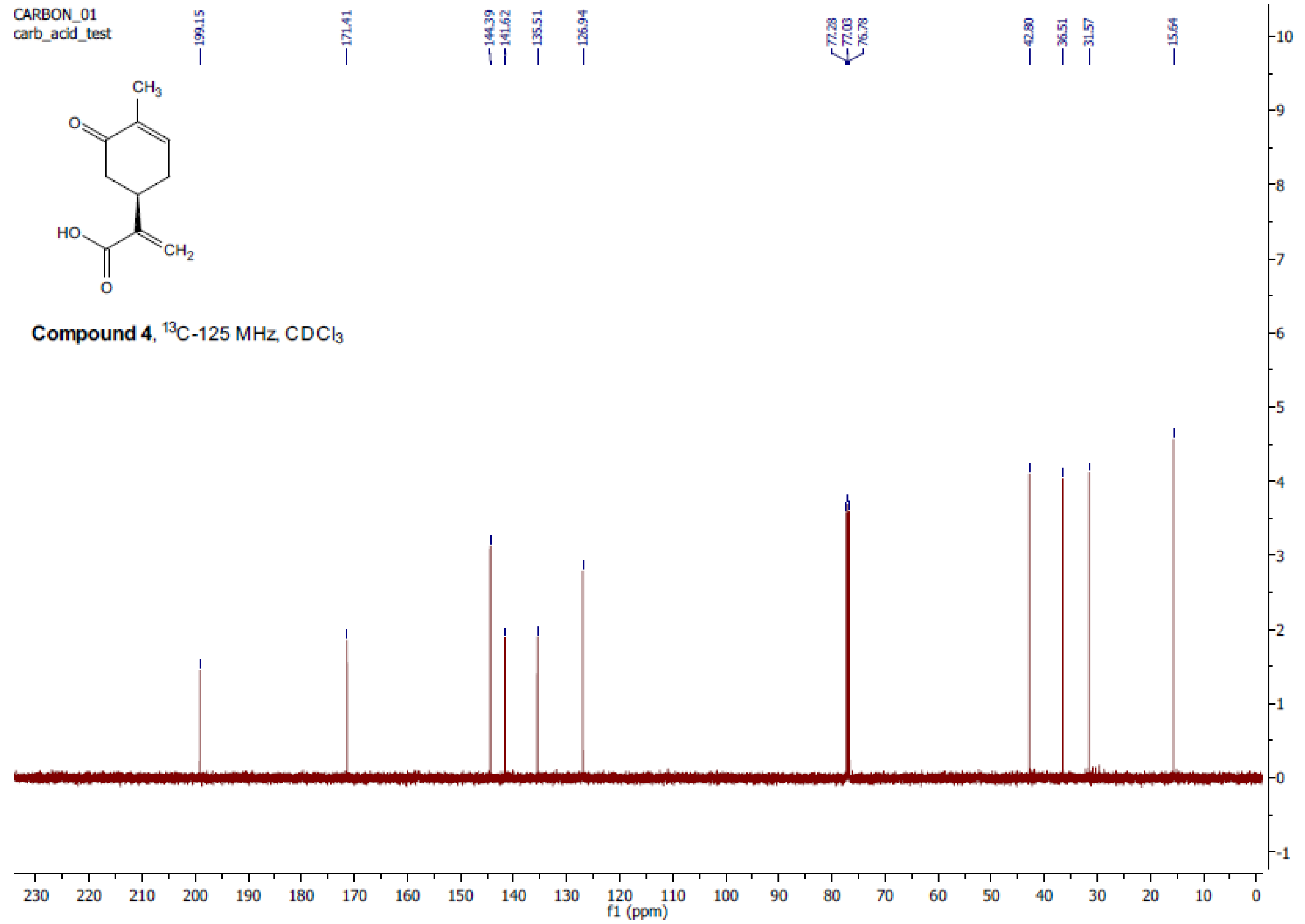

Compound 4, ^{13}C-125 MHz, $CDCl_3$

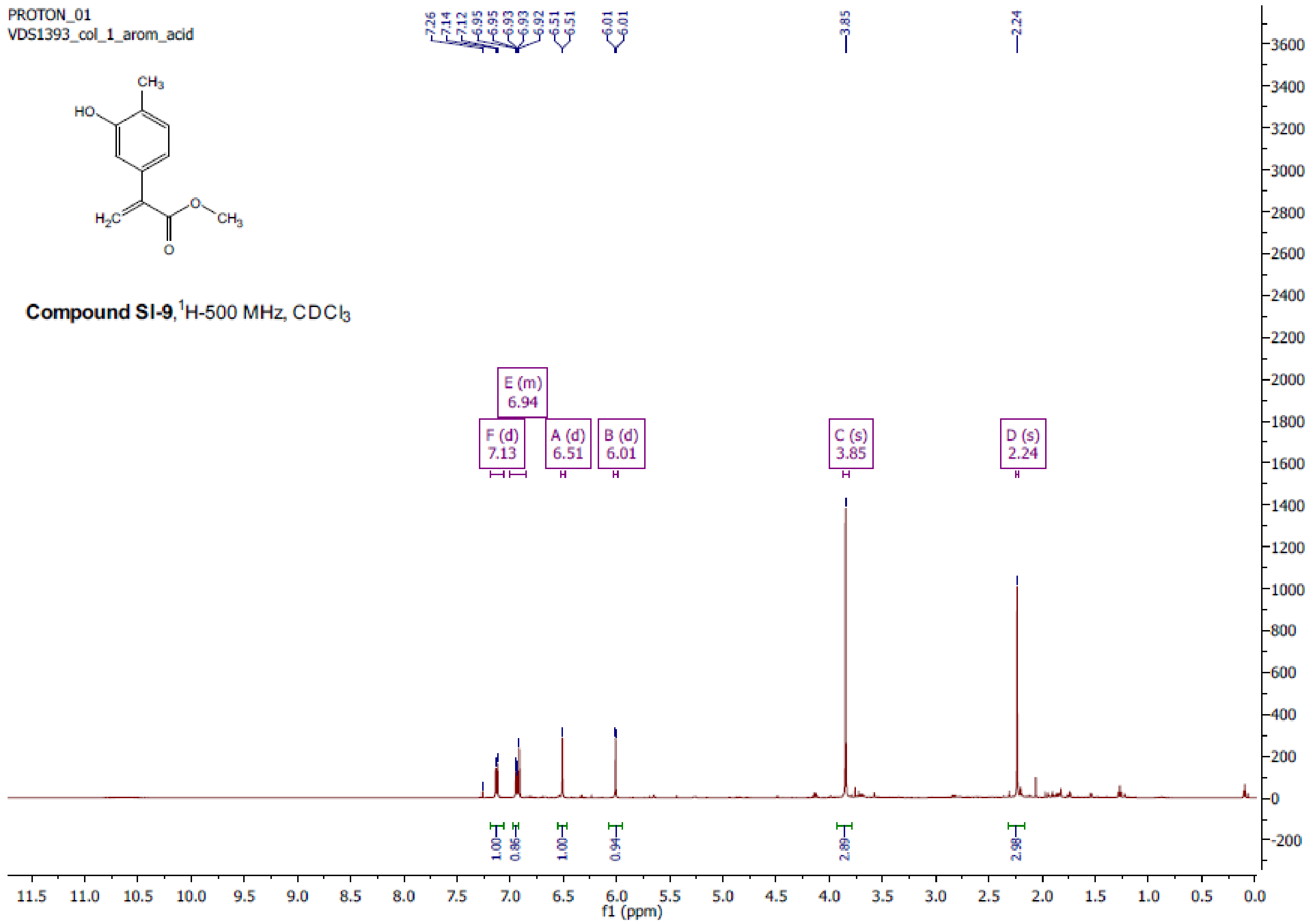

Compound SI-9, ^{1}H-500 MHz, $CDCl_3$

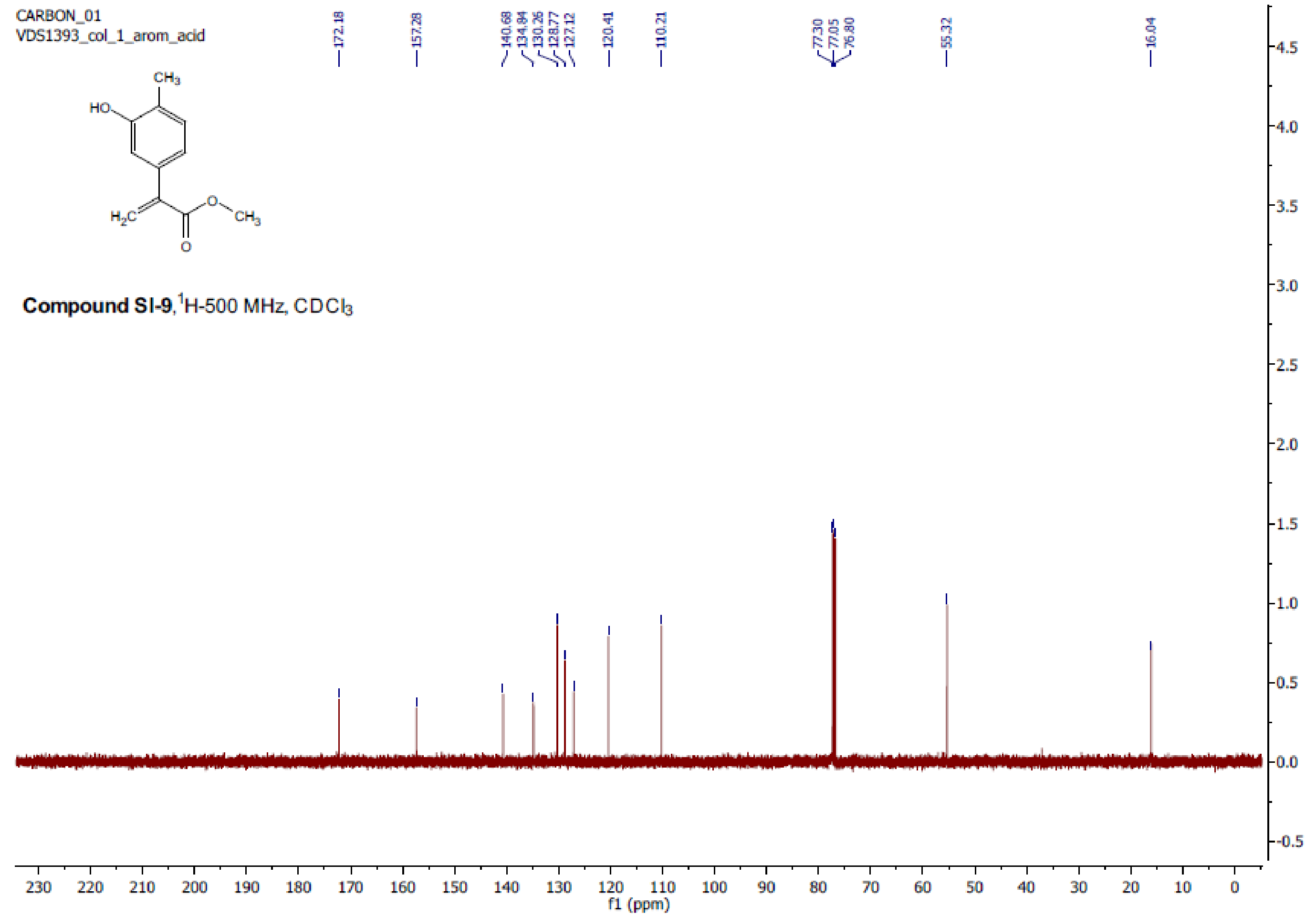
CARBON_01
VDS1393_col_1_arom_acid
Compound SI-9, 1H-500 MHz, CDCl3
172.18
157.28
140.68
134.84
130.26
128.77
127.12
120.41
110.21
77.30
77.05
76.80
55.32
16.04
f1 (ppm)

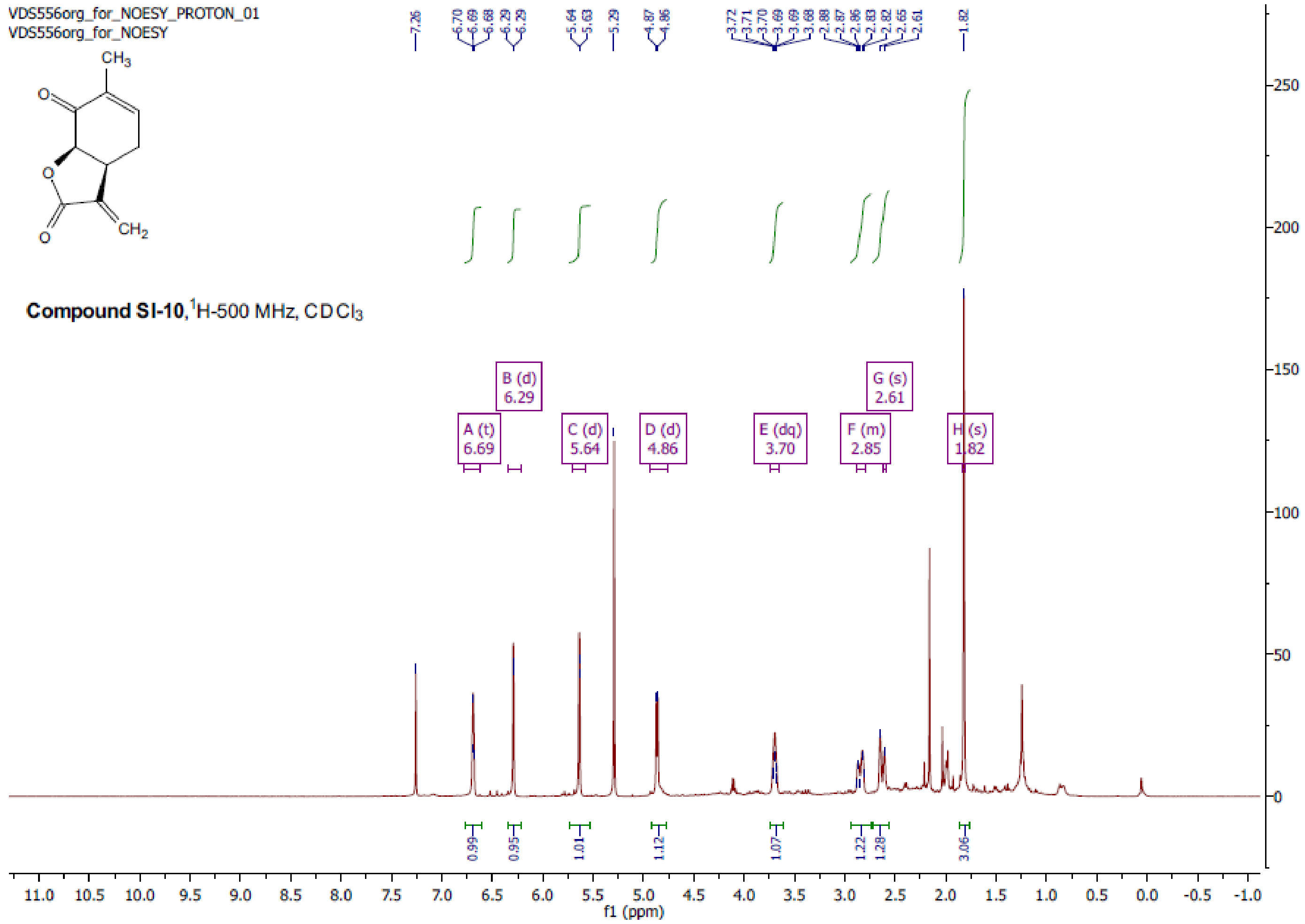
VDS556org_for_NOESY_PROTON_01
VDS556org_for_NOESY
Compound SI-10, 1H-500 MHz, CDCl3
A (t) 6.69
B (d) 6.29
C (d) 5.64
D (d) 4.86
E (dq) 3.70
F (m) 2.85
G (s) 2.61
H (s) 1.82
0.99
0.95
1.01
1.12
1.07
1.22
1.28
3.06
f1 (ppm)

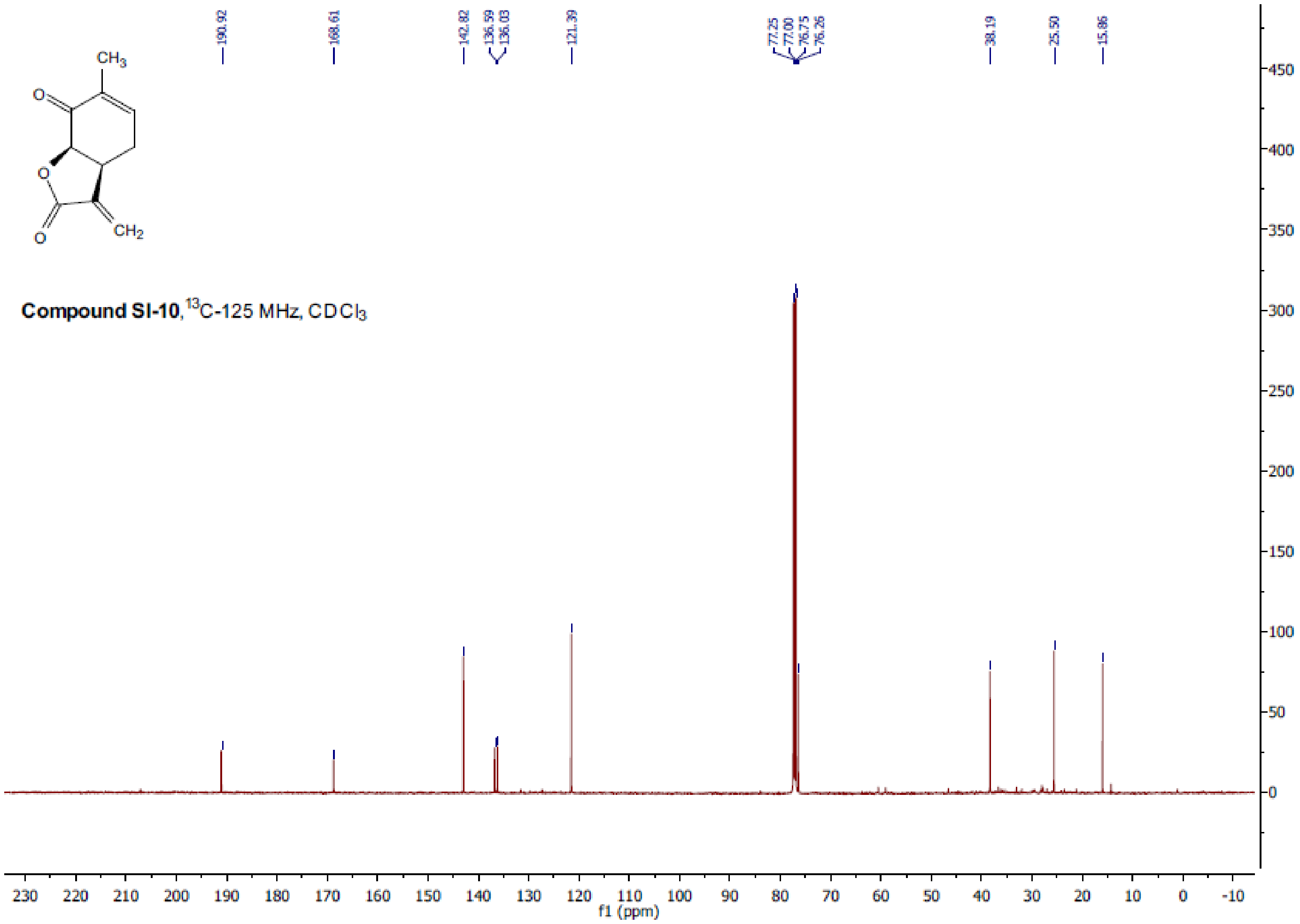

CH3
O
O
O
CH2
Compound SI-10, 13C-125 MHz, CDCl3
190.92
168.61
142.82
136.59
136.03
121.39
77.25
77.00
76.75
76.26
38.19
25.50
15.86
f1 (ppm)

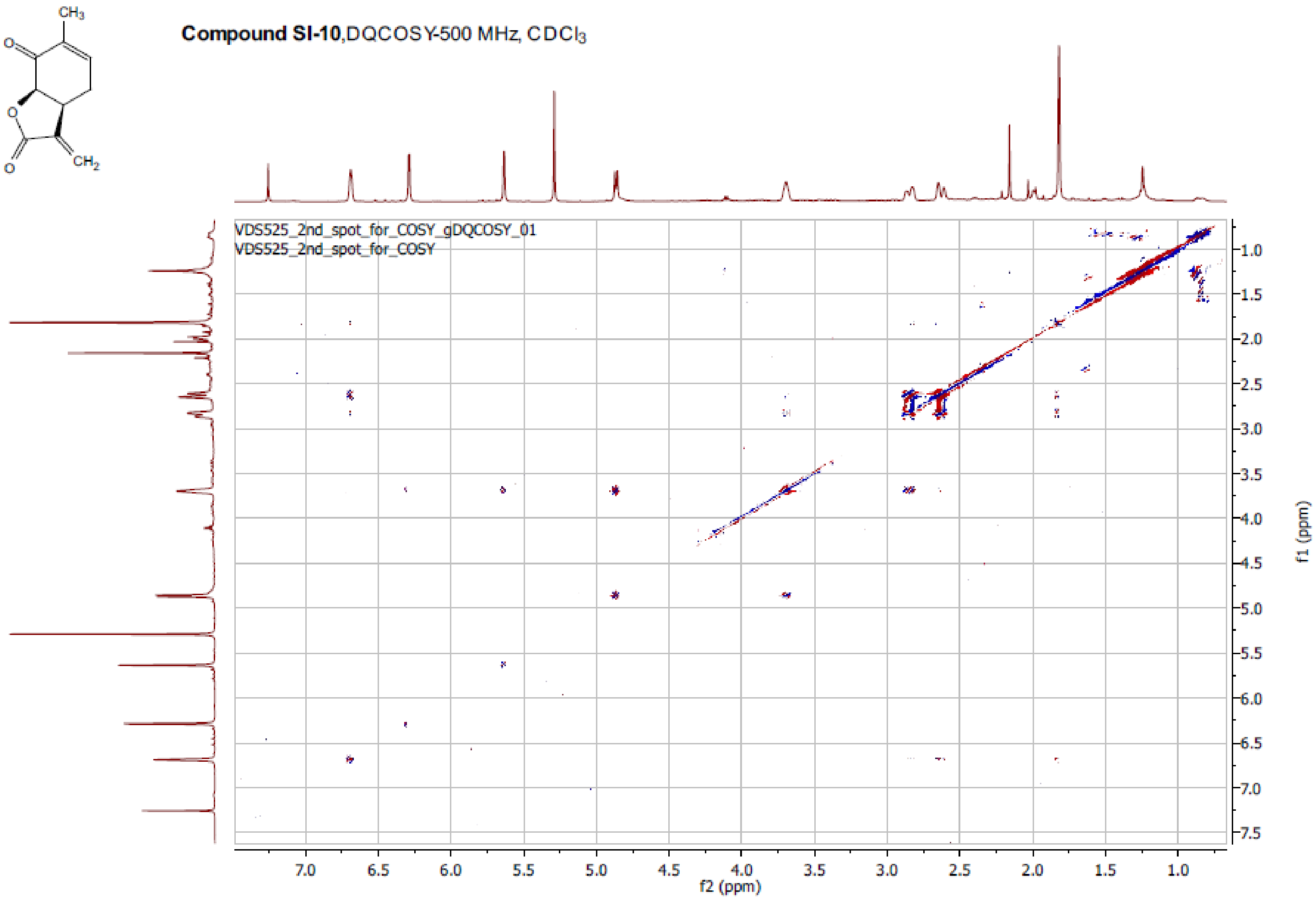

CH3
O
O
O
CH2
Compound SI-10, DQCOSY-500 MHz, CDCl3
VDS525_2nd_spot_for_COSY_gDQCOSY_01
VDS525_2nd_spot_for_COSY
f2 (ppm)
f1 (ppm)

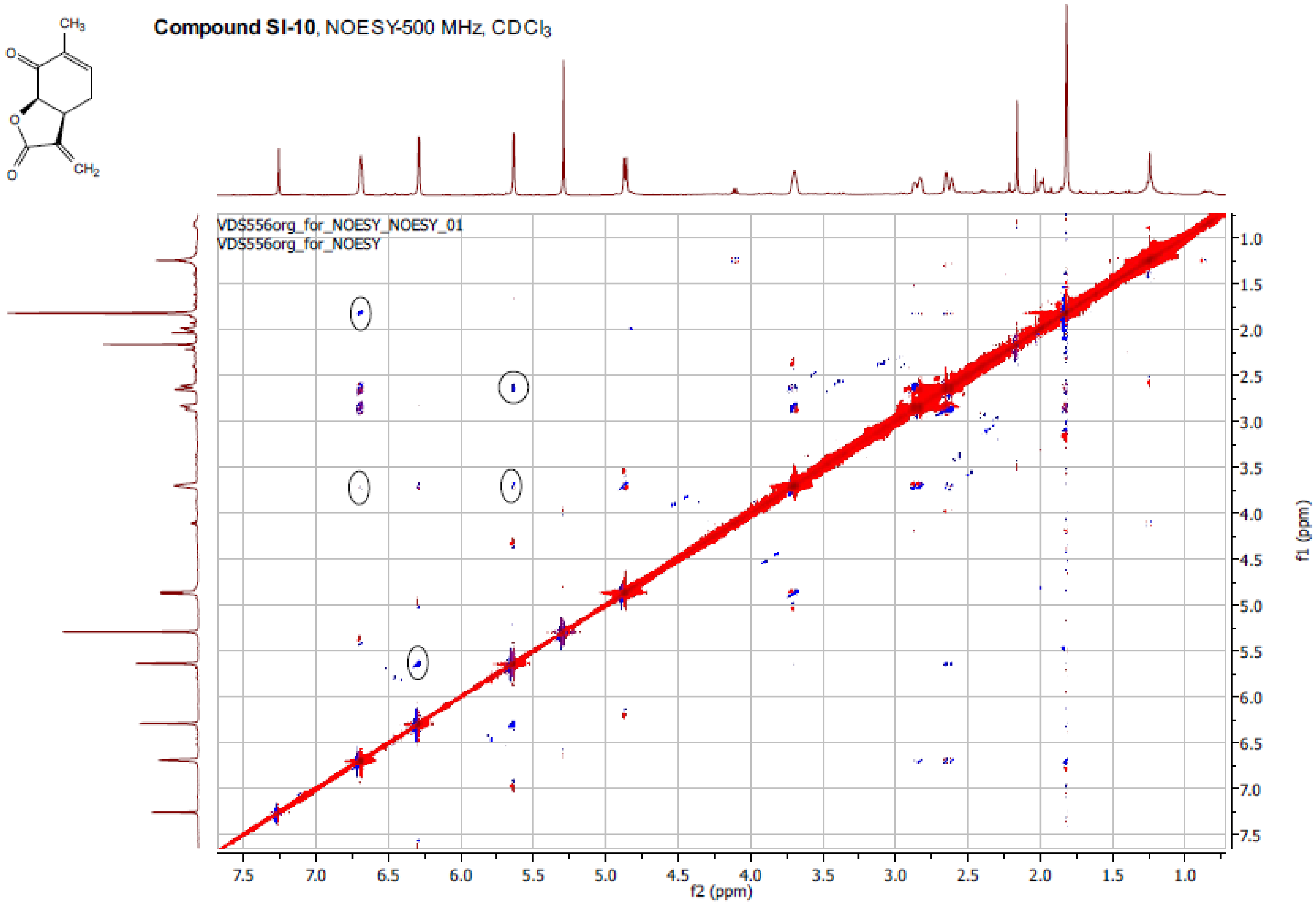

Compound SI-10, NOESY-500 MHz, CDCl3
VDS556org_for_NOESY_NOESY_01
VDS556org_for_NOESY
f2 (ppm)
f1 (ppm)

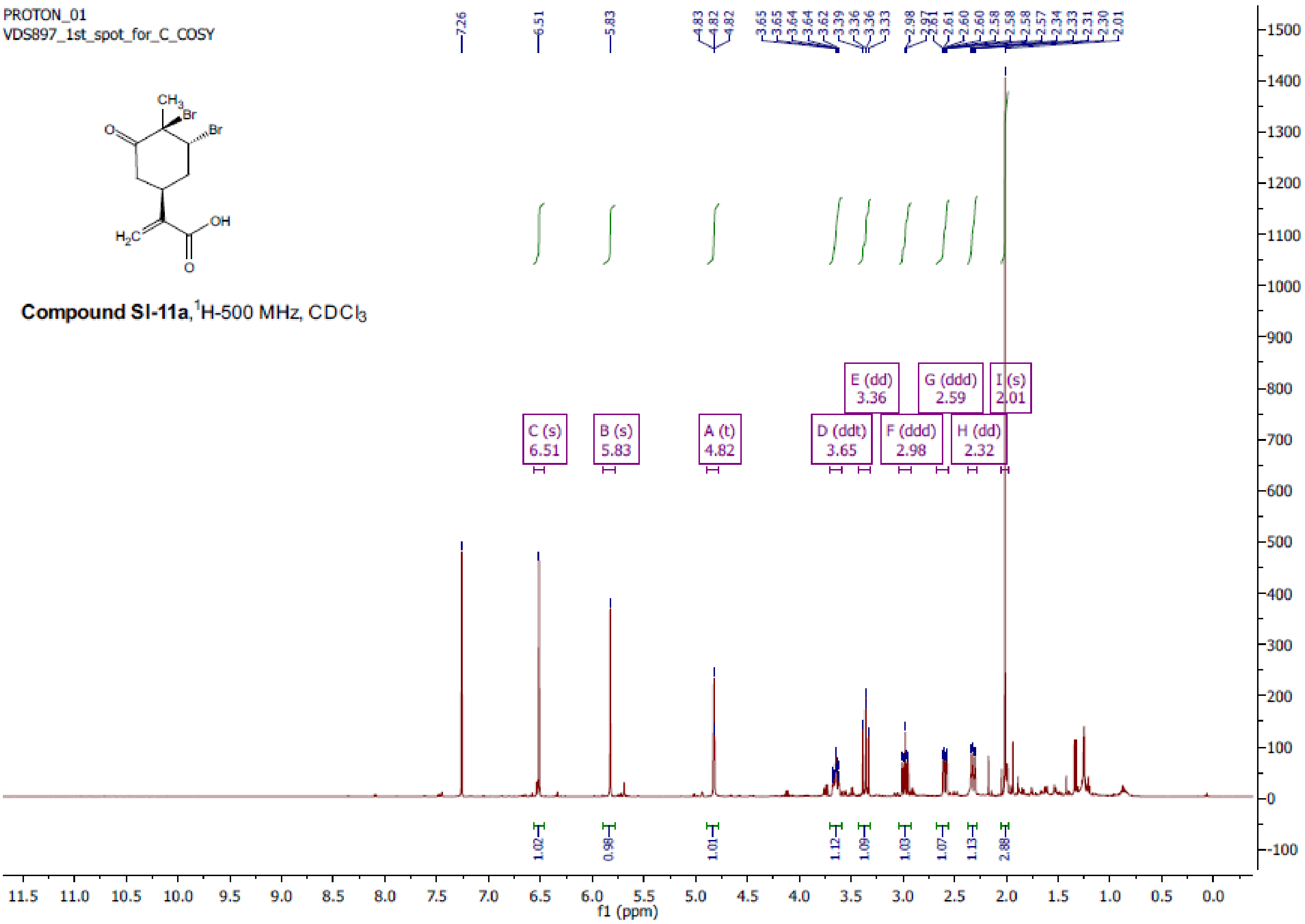

PROTON_01
VDS897_1st_spot_for_C_COSY
Compound SI-11a, 1H-500 MHz, CDCl3
C (s) 6.51
B (s) 5.83
A (t) 4.82
D (ddt) 3.65
E (dd) 3.36
F (ddd) 2.98
G (ddd) 2.59
H (dd) 2.32
I (s) 2.01
f1 (ppm)

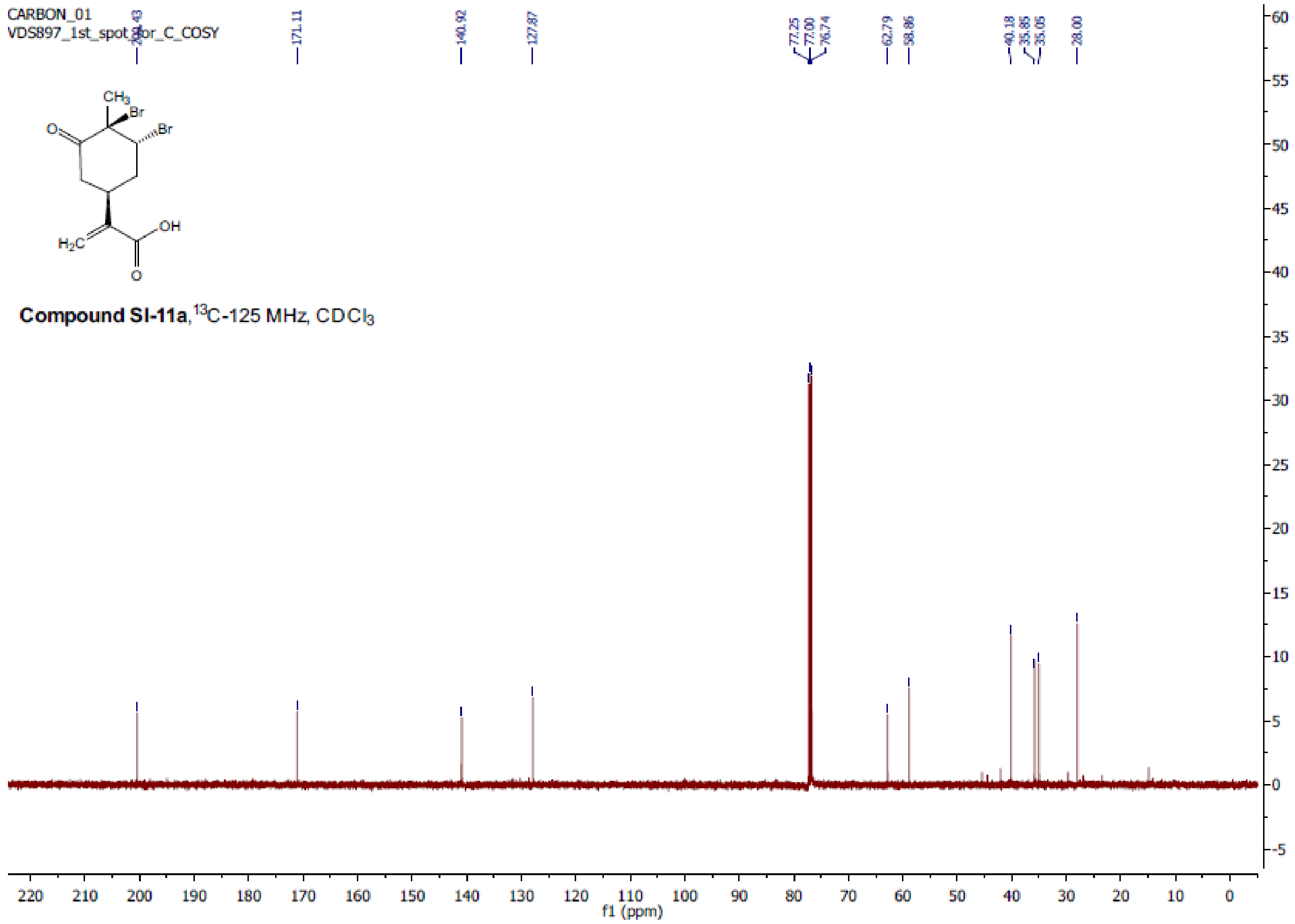
CARBON_01
VDS897_1st_spot_for_C_COSY
171.11
140.92
127.87
77.25
77.00
76.74
62.79
58.86
40.18
35.85
35.05
28.00
Compound SI-11a, ^{13}C-125 MHz, CDCl$_3$
f1 (ppm)

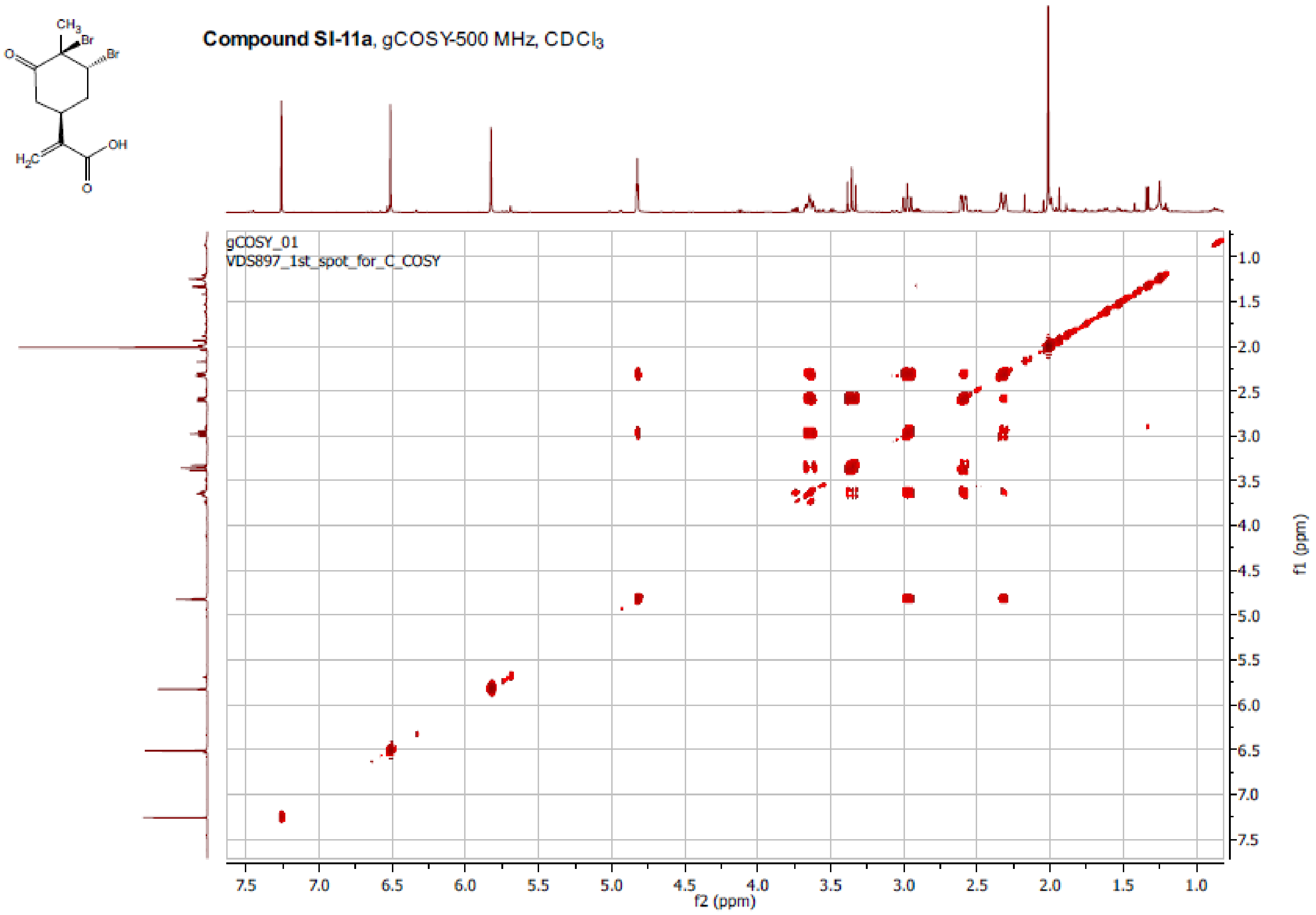
Compound SI-11a, gCOSY-500 MHz, CDCl$_3$
gCOSY_01
VDS897_1st_spot_for_C_COSY
f2 (ppm)
f1 (ppm)

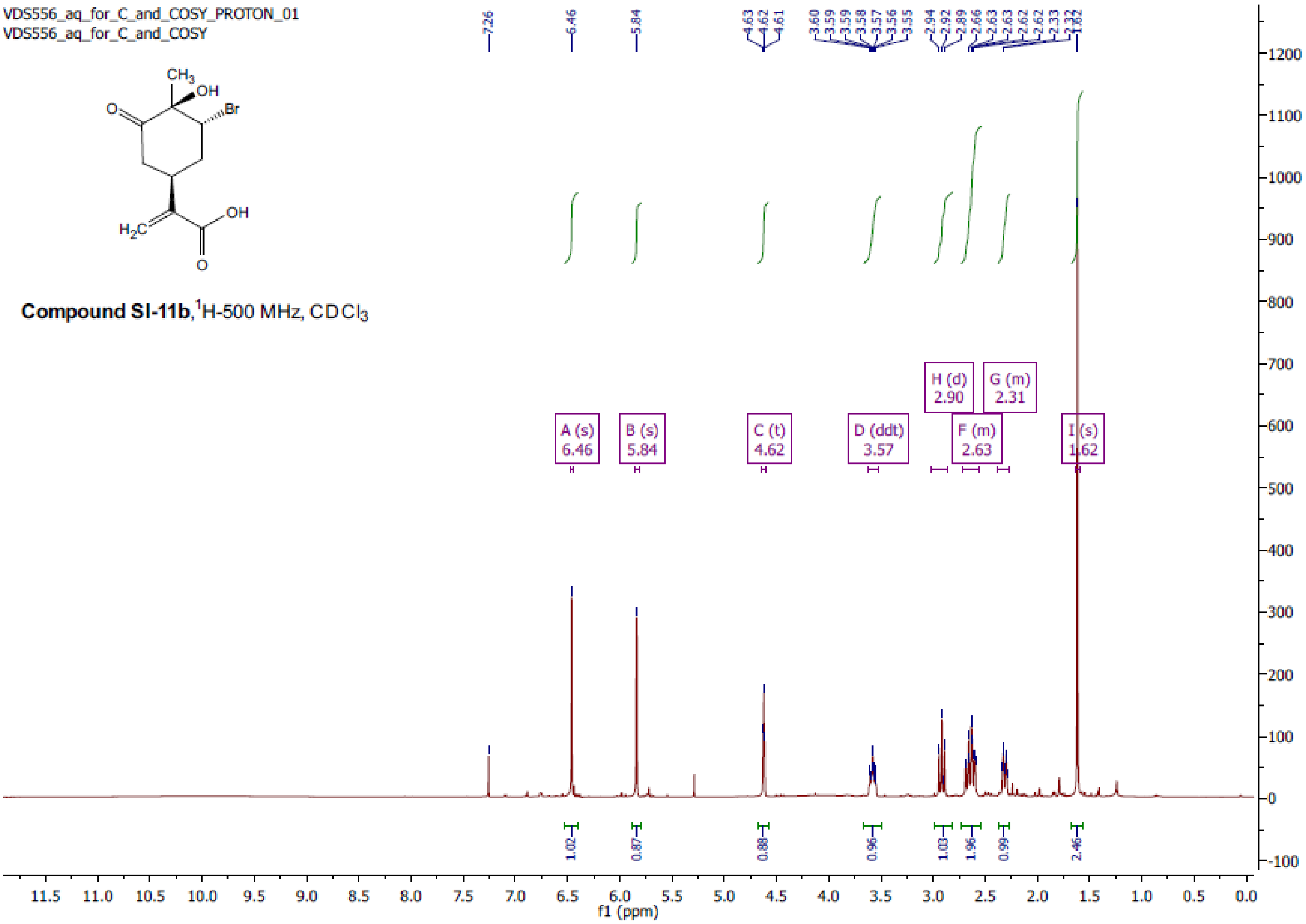

VDS556_aq_for_C_and_COSY_PROTON_01
VDS556_aq_for_C_and_COSY
Compound SI-11b, ¹H-500 MHz, CDCl3
7.26
6.46
5.84
4.63 4.62 4.61
3.60 3.59 3.59 3.58 3.57 3.56 3.55
2.94 2.92 2.89 2.66 2.63 2.63 2.62 2.62 2.33 1.62 1.62
A (s) 6.46
B (s) 5.84
C (t) 4.62
D (ddt) 3.57
H (d) 2.90
F (m) 2.63
G (m) 2.31
I (s) 1.62
1.02 0.87 0.88 0.96 1.03 1.96 0.99 2.46
f1 (ppm)

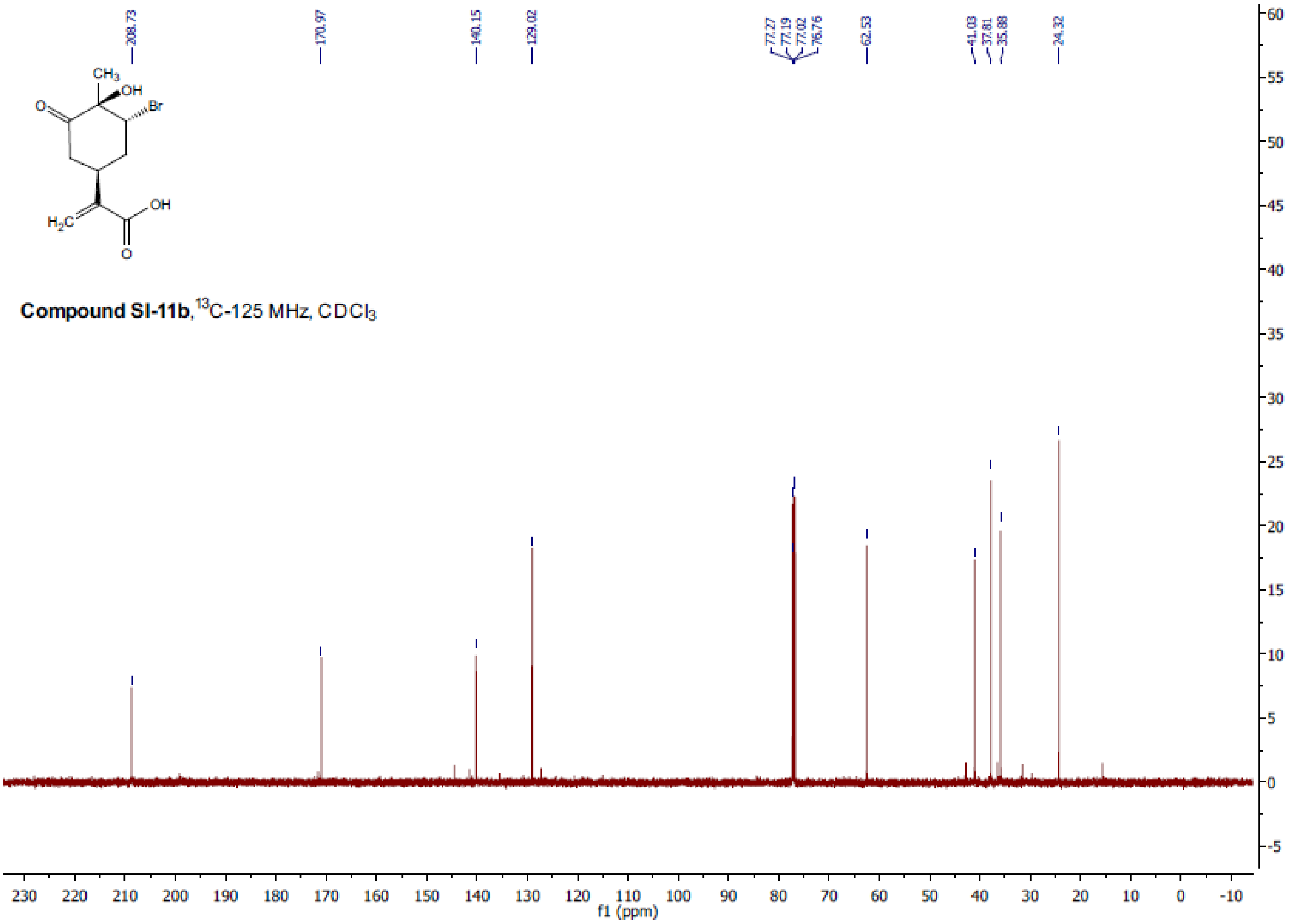

208.73
170.97
140.15
129.02
77.27 77.19 77.02 76.76
62.53
41.03 37.81 35.88
24.32
Compound SI-11b, ¹³C-125 MHz, CDCl3
f1 (ppm)

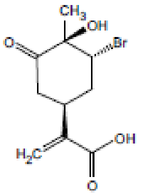

Compound SI-11b, gCOSY-500 MHz, $CDCl_3$

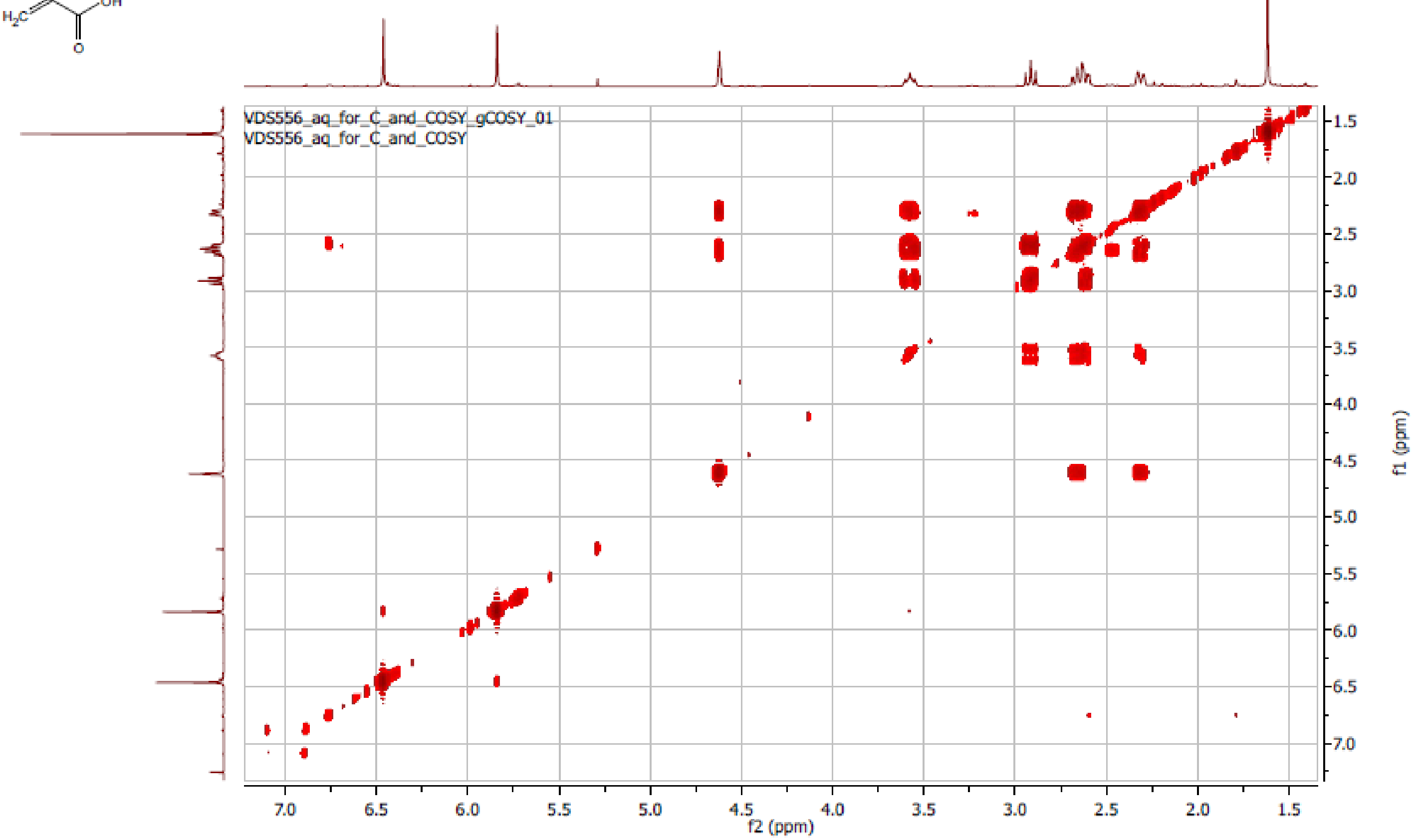

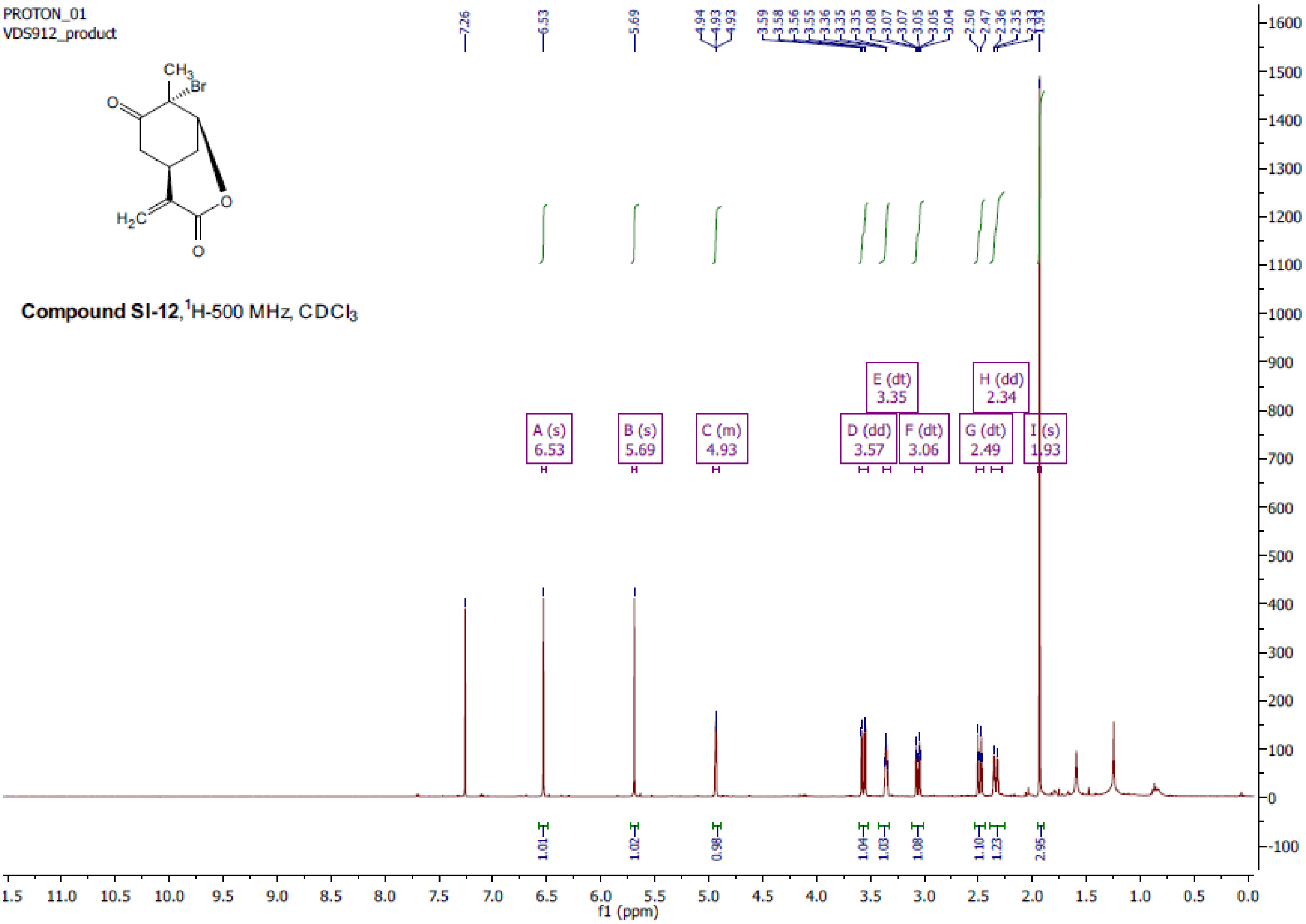

Compound SI-12, ^{1}H-500 MHz, $CDCl_3$

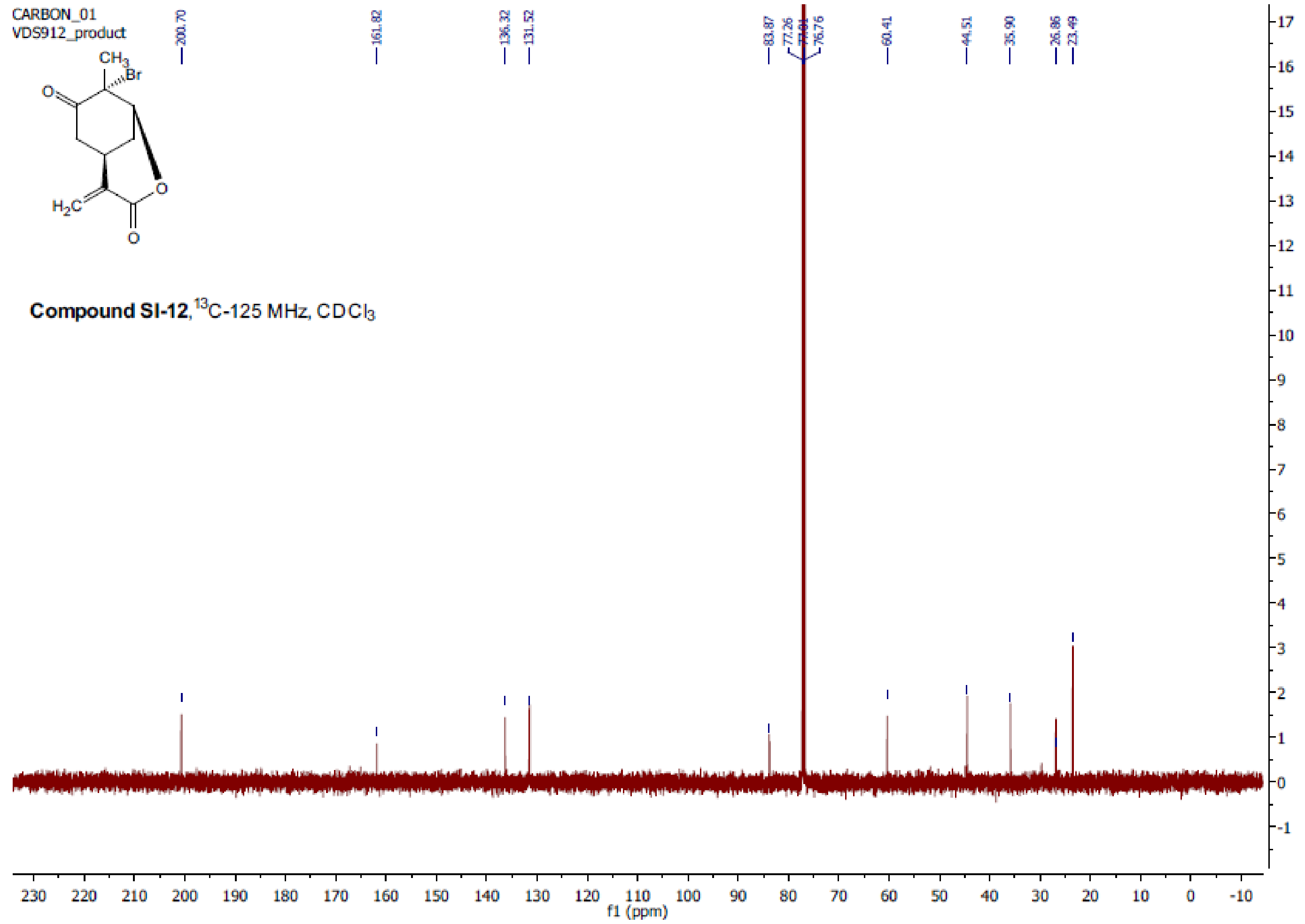

Compound SI-12, ^{13}C-125 MHz, $CDCl_3$

Compound SI-12, gCOSY, $CDCl_3$

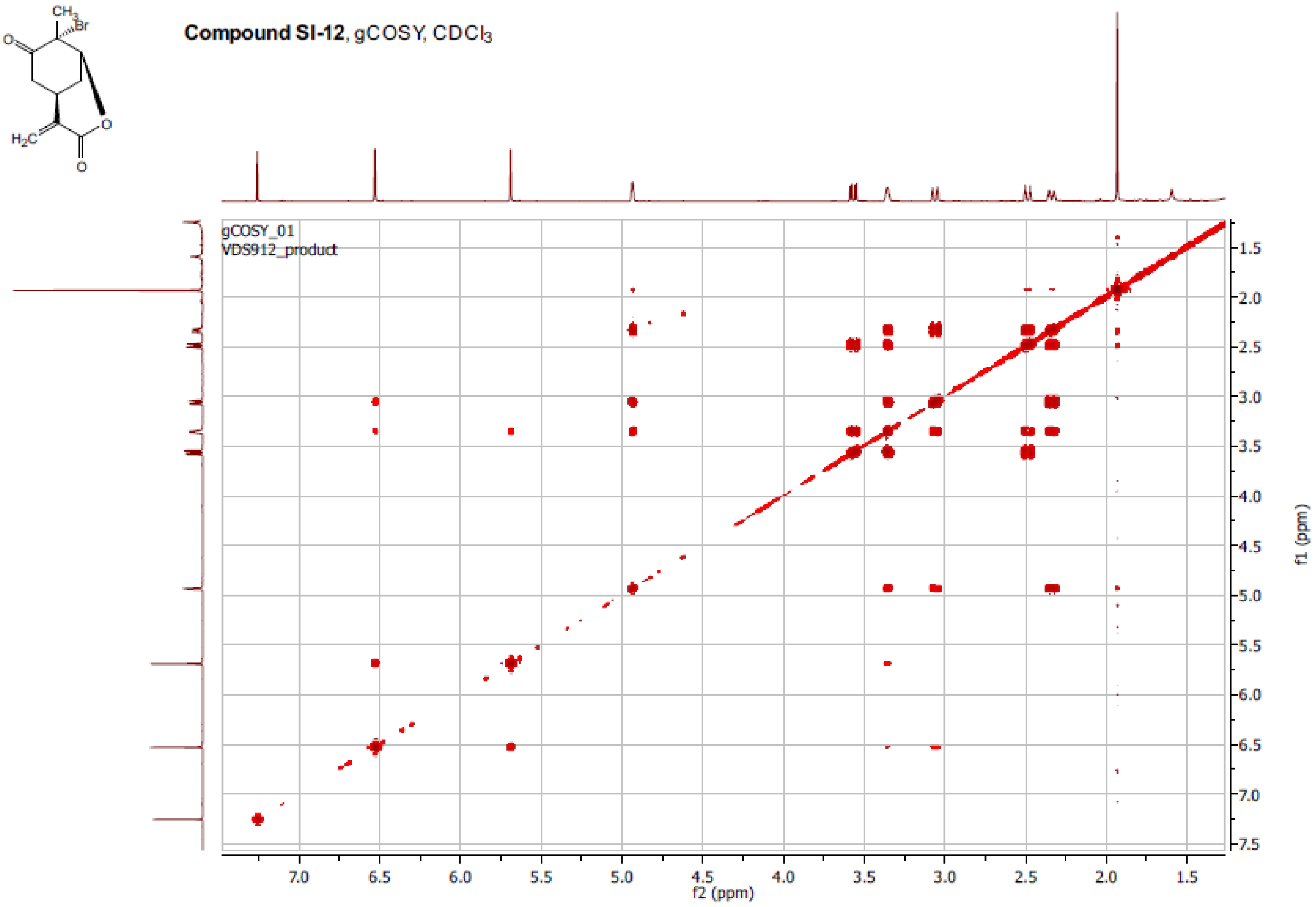

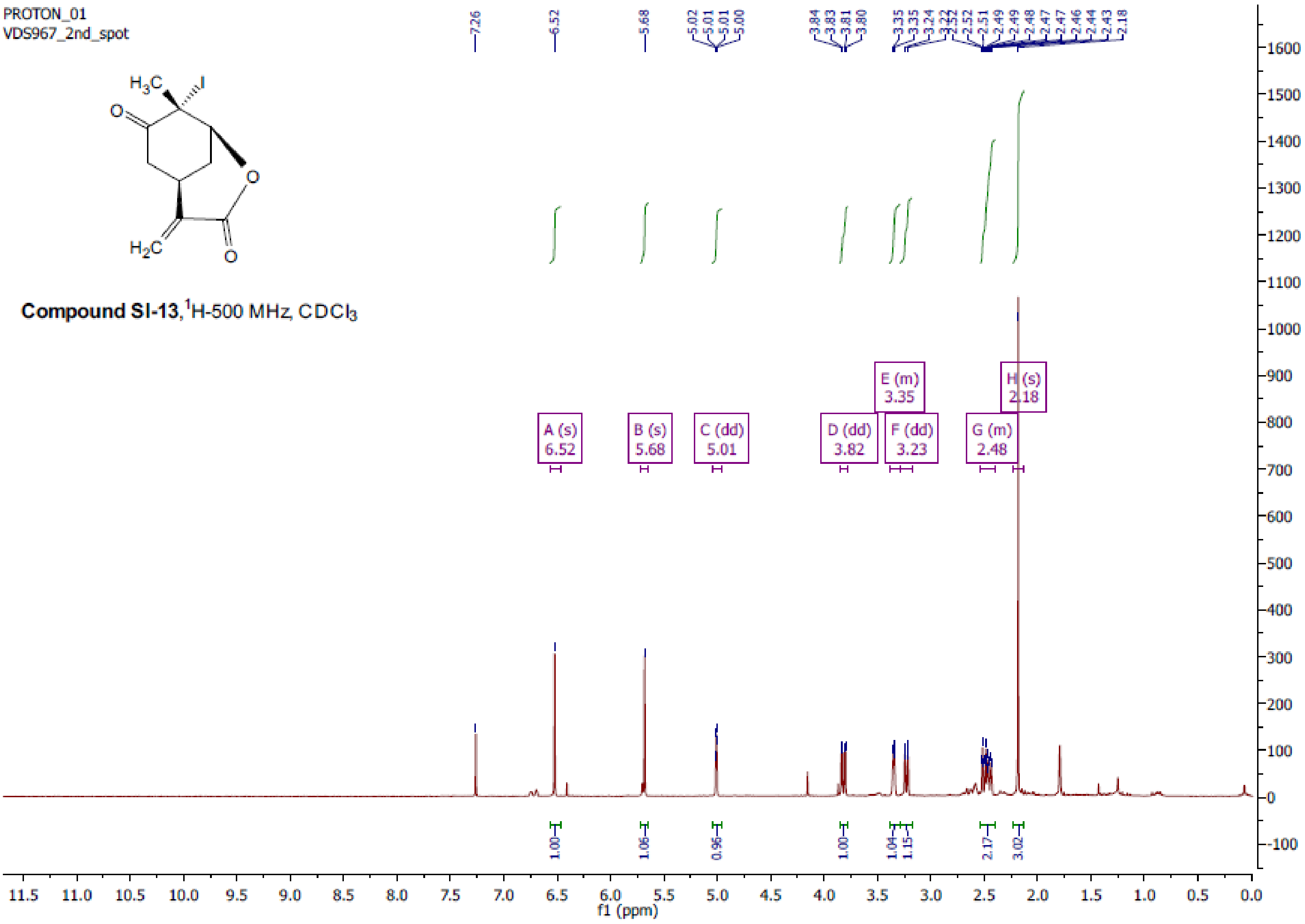
PROTON_01
VDS967_2nd_spot
Compound SI-13, 1H-500 MHz, CDCl3
A (s) 6.52
B (s) 5.68
C (dd) 5.01
D (dd) 3.82
E (m) 3.35
F (dd) 3.23
G (m) 2.48
H (s) 2.18
f1 (ppm)

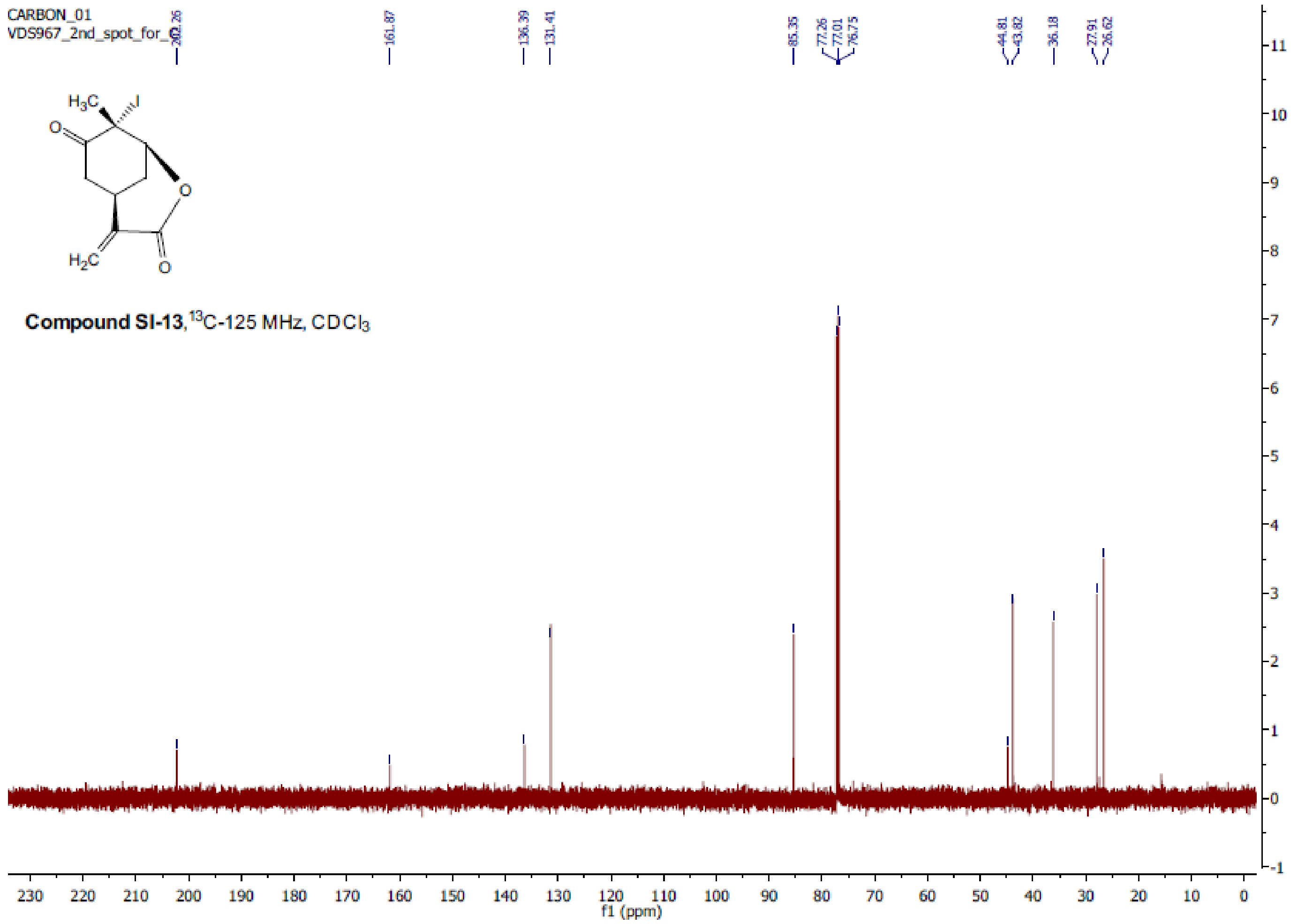
CARBON_01
Compound SI-13, 13C-125 MHz, CDCl3
161.87
136.39
131.41
85.35
77.26
77.01
76.75
44.81
43.82
36.18
27.91
26.62
f1 (ppm)

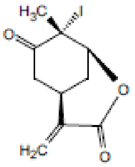

Compound SI-13, gCOSY-500 MHz, CDCl3

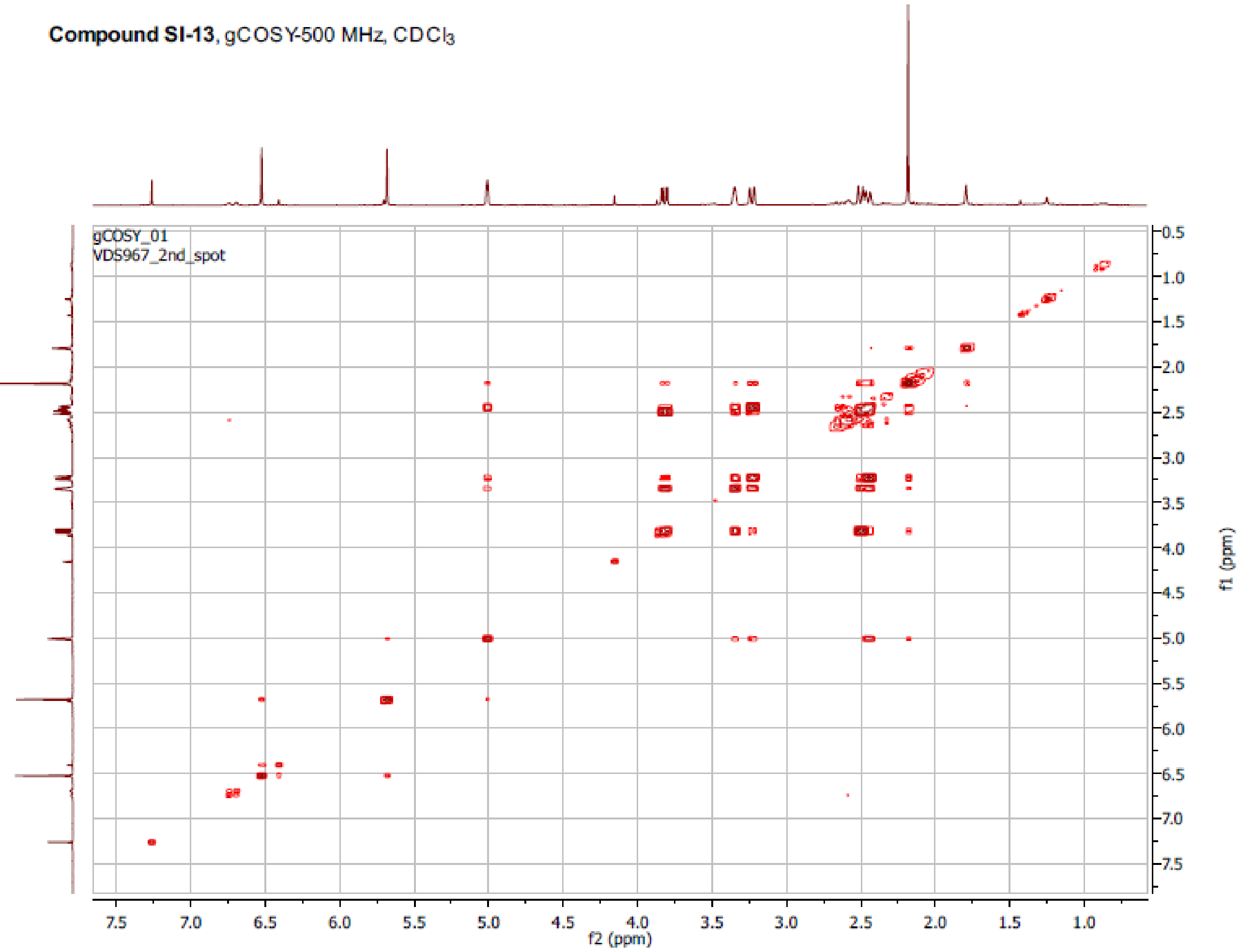

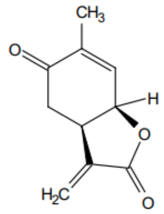

Compound 8, ^{1}H-500 MHz, CDCl$_3$

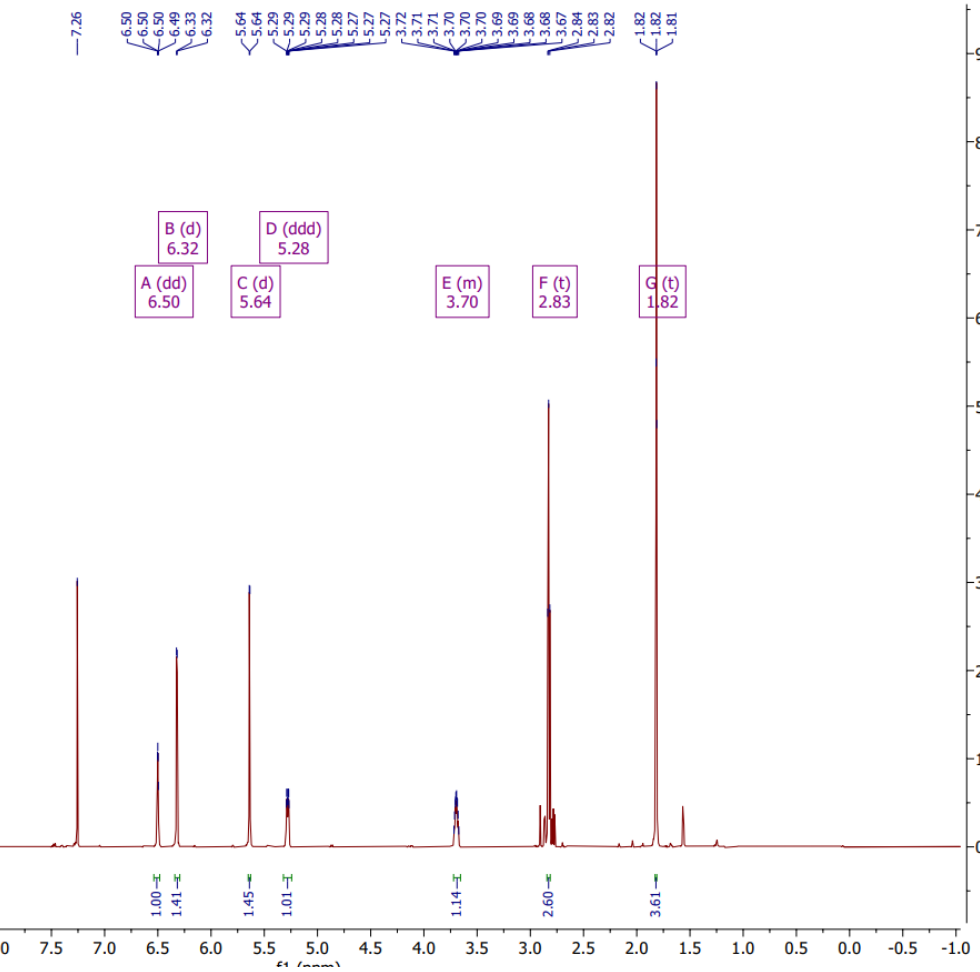

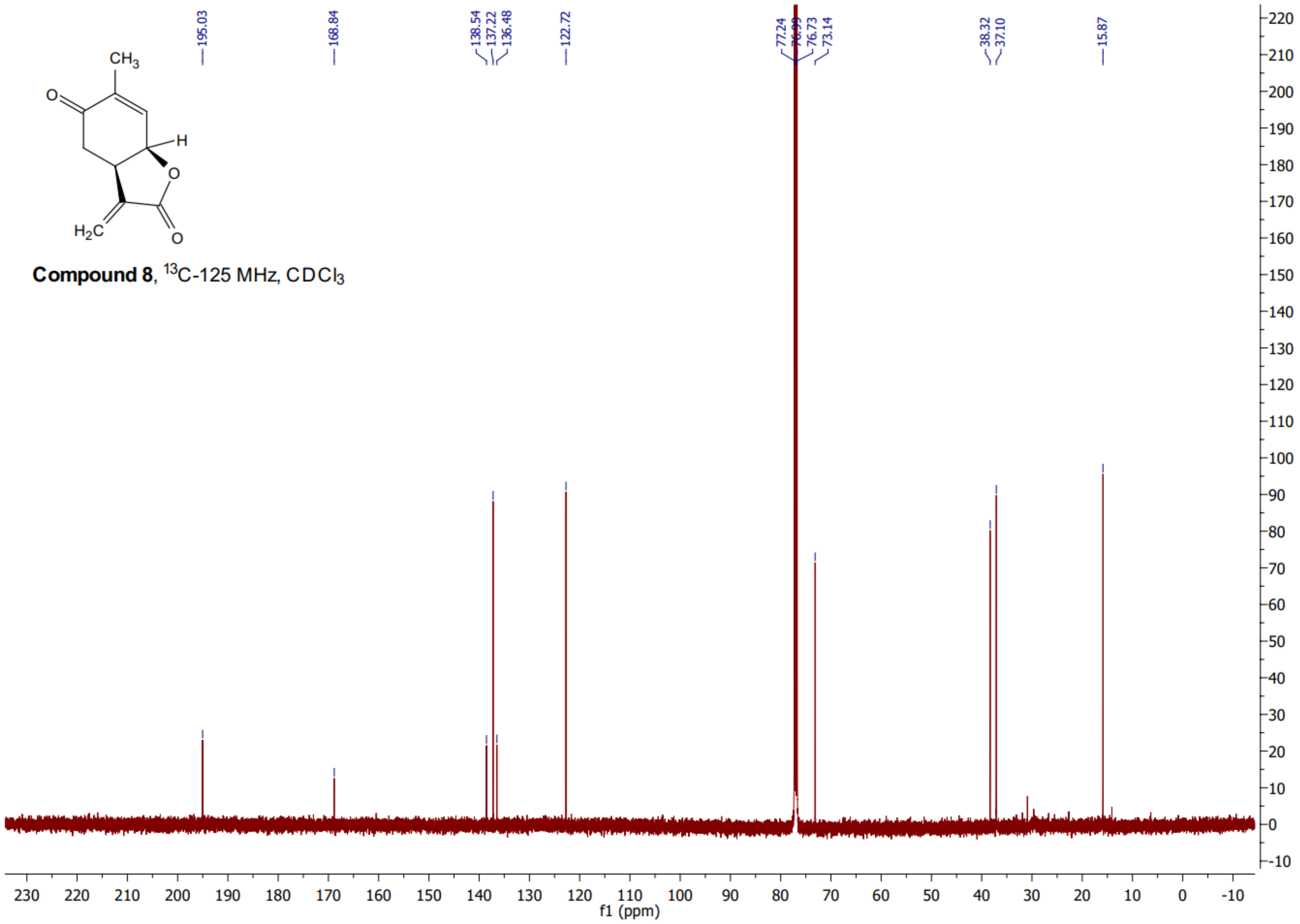
Compound 8, 13C-125 MHz, CDCl3
195.03
168.84
138.54
137.22
136.48
122.72
77.24
76.99
76.73
73.14
38.32
37.10
15.87
f1 (ppm)

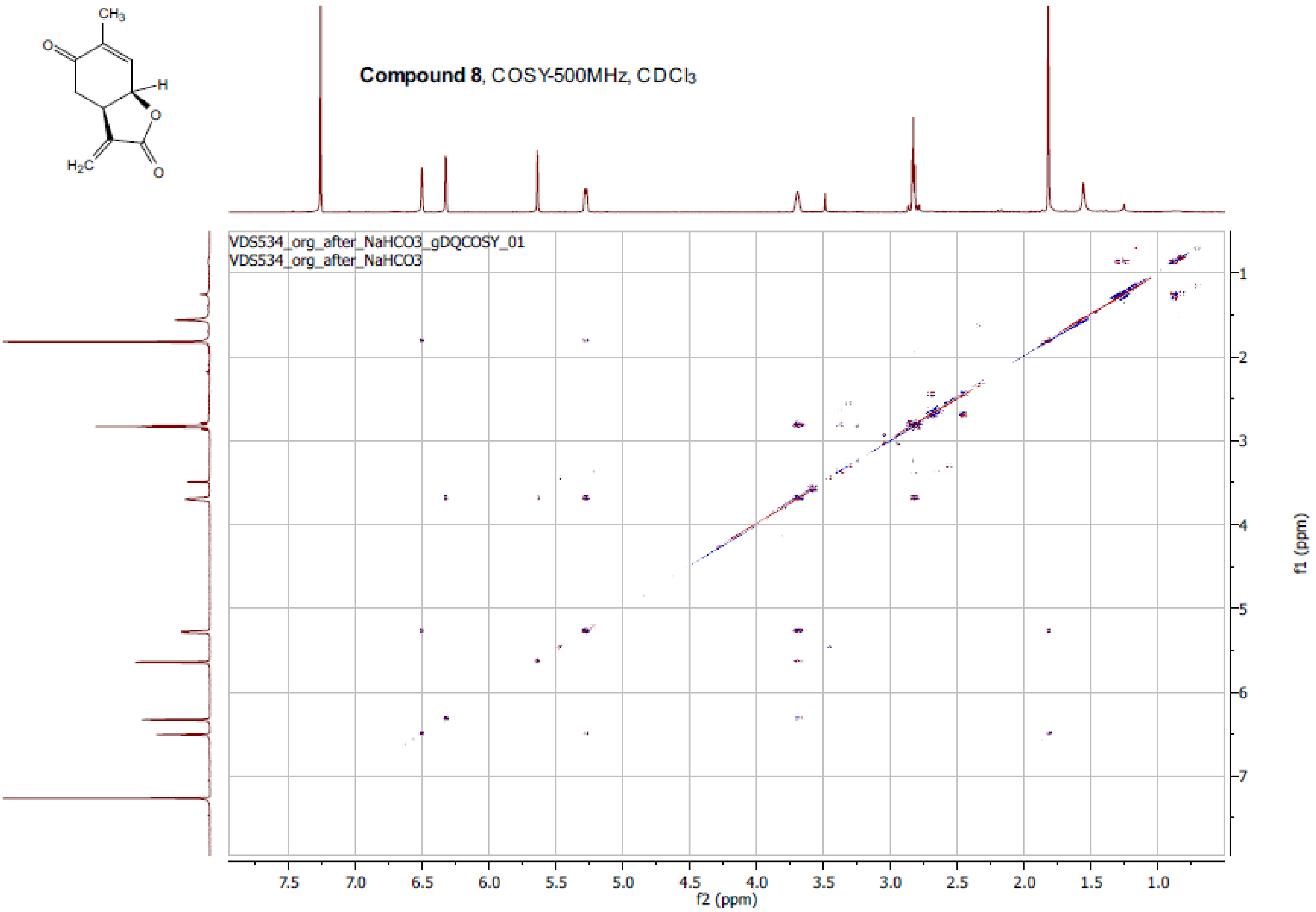
Compound 8, COSY-500MHz, CDCl3
VDS534_org_after_NaHCO3_gDQCOSY_01
VDS534_org_after_NaHCO3
f2 (ppm)
f1 (ppm)

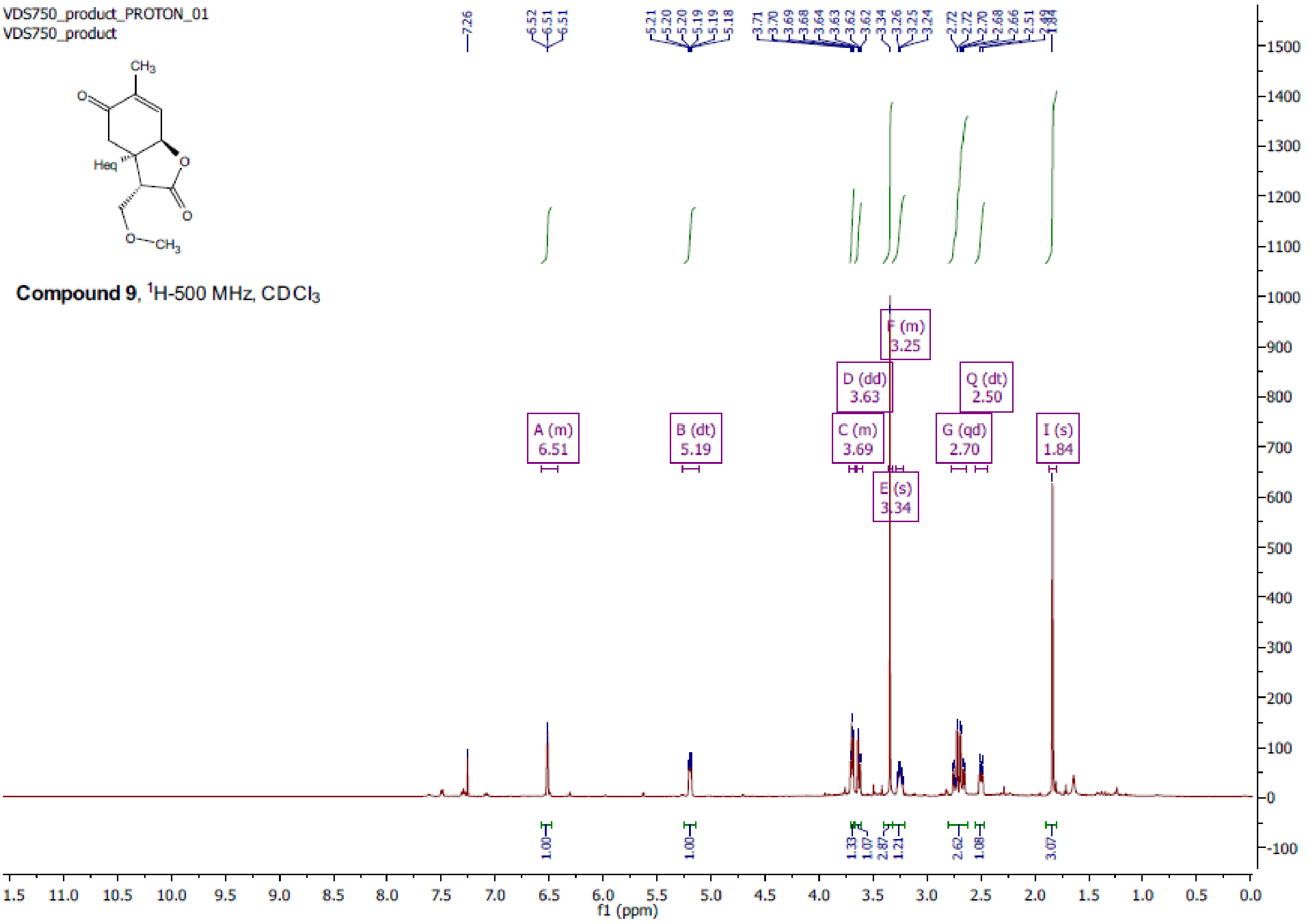

VDS750_product_PROTON_01
VDS750_product
Compound 9, 1H-500 MHz, CDCl3
A (m) 6.51
B (dt) 5.19
C (m) 3.69
D (dd) 3.63
E (s) 3.34
F (m) 3.25
G (qd) 2.70
Q (dt) 2.50
I (s) 1.84
f1 (ppm)

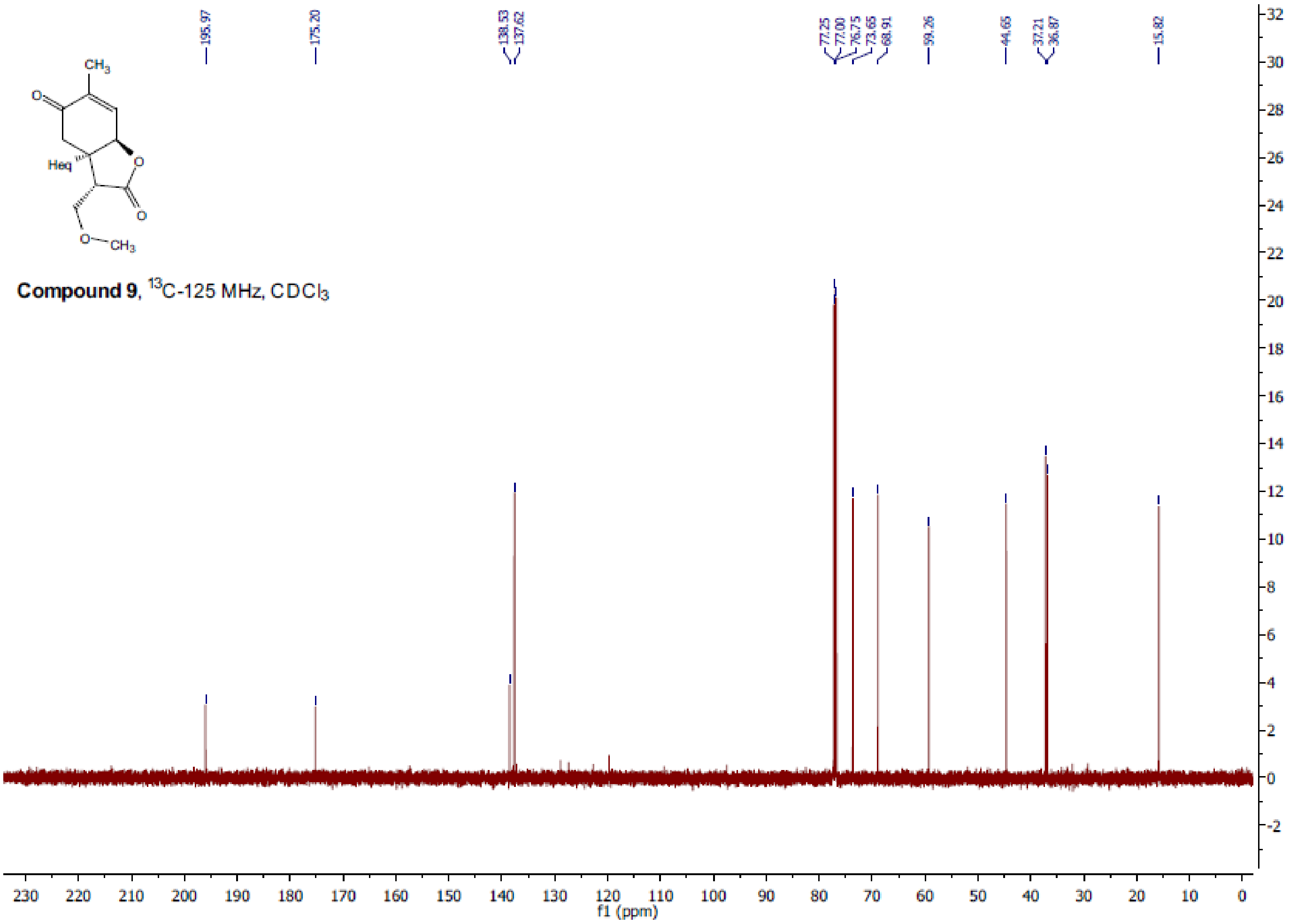

Compound 9, 13C-125 MHz, CDCl3
195.97
175.20
138.53
137.62
77.25
77.00
76.75
73.65
68.91
59.26
44.65
37.21
36.87
15.82
f1 (ppm)

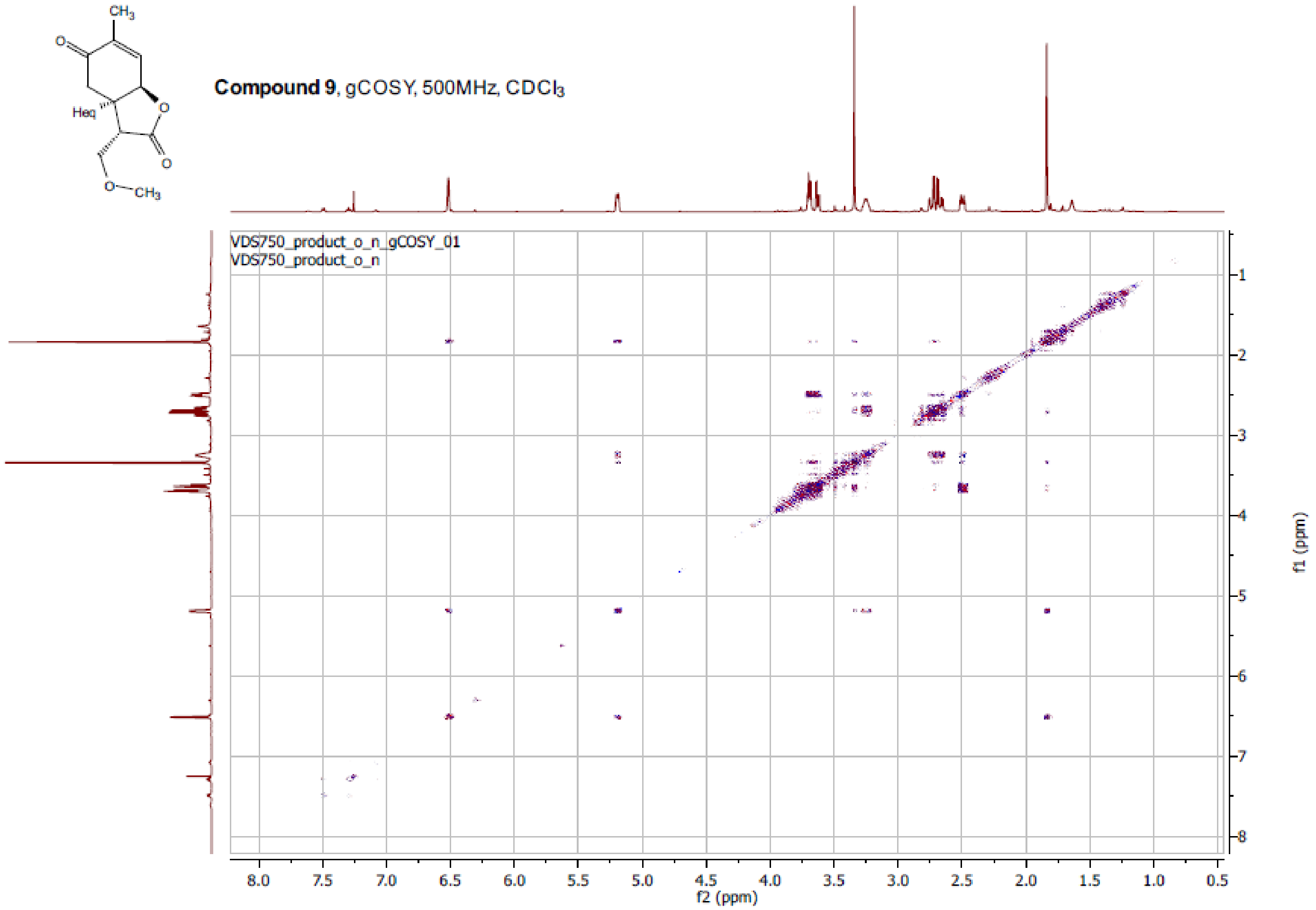
Compound 9, gCOSY, 500MHz, CDCl3
VDS750_product_o_n_gCOSY_01
VDS750_product_o_n
f2 (ppm)
f1 (ppm)

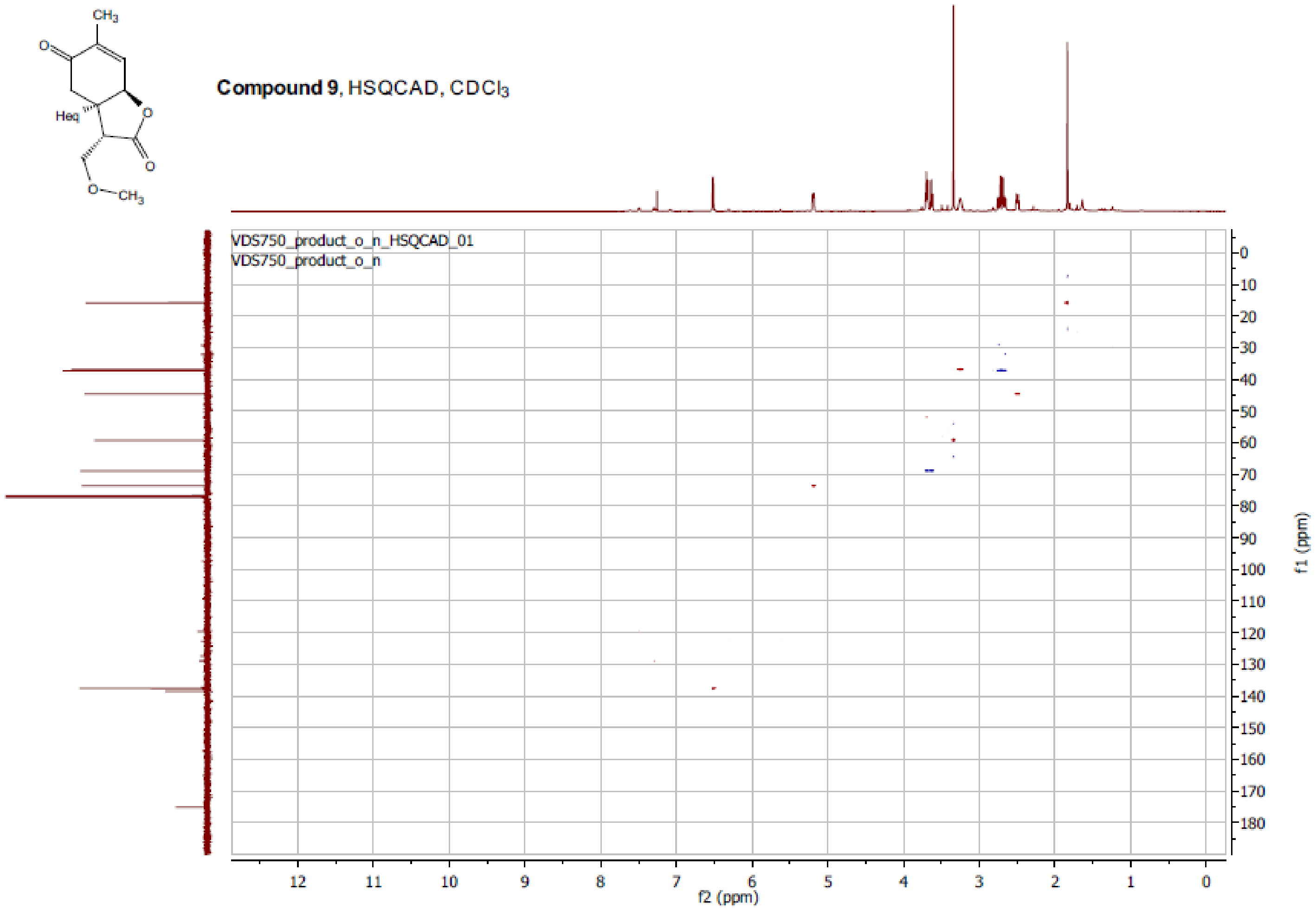
Compound 9, HSQCAD, CDCl3
VDS750_product_o_n_HSQCAD_01
VDS750_product_o_n
f2 (ppm)
f1 (ppm)

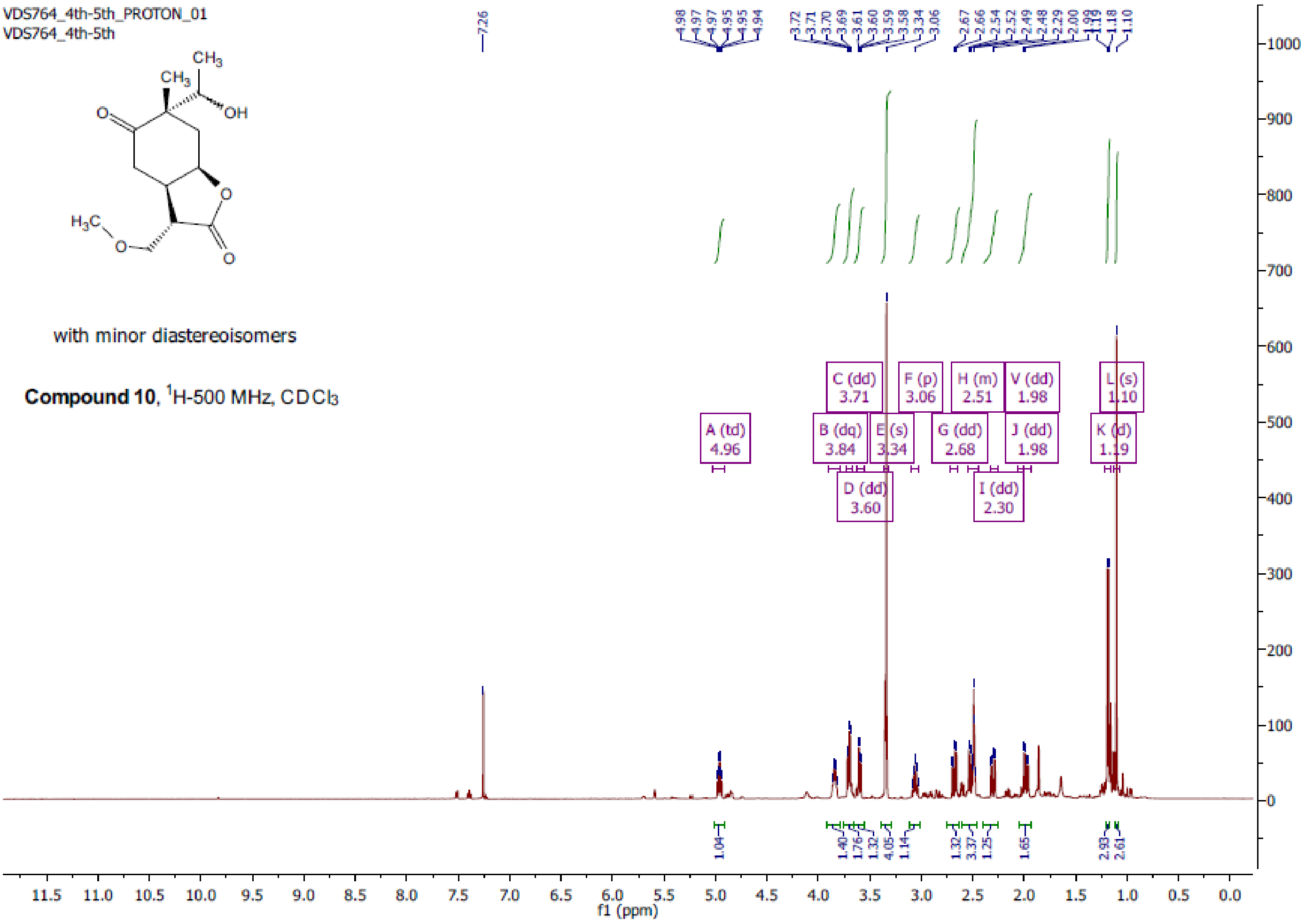
VDS764_4th-5th_PROTON_01
VDS764_4th-5th
with minor diastereoisomers
Compound 10, 1H-500 MHz, CDCl3
A (td) 4.96
B (dq) 3.84
C (dd) 3.71
D (dd) 3.60
E (s) 3.34
F (p) 3.06
G (dd) 2.68
H (m) 2.51
I (dd) 2.30
J (dd) 1.98
V (dd) 1.98
K (d) 1.19
L (s) 1.10
f1 (ppm)

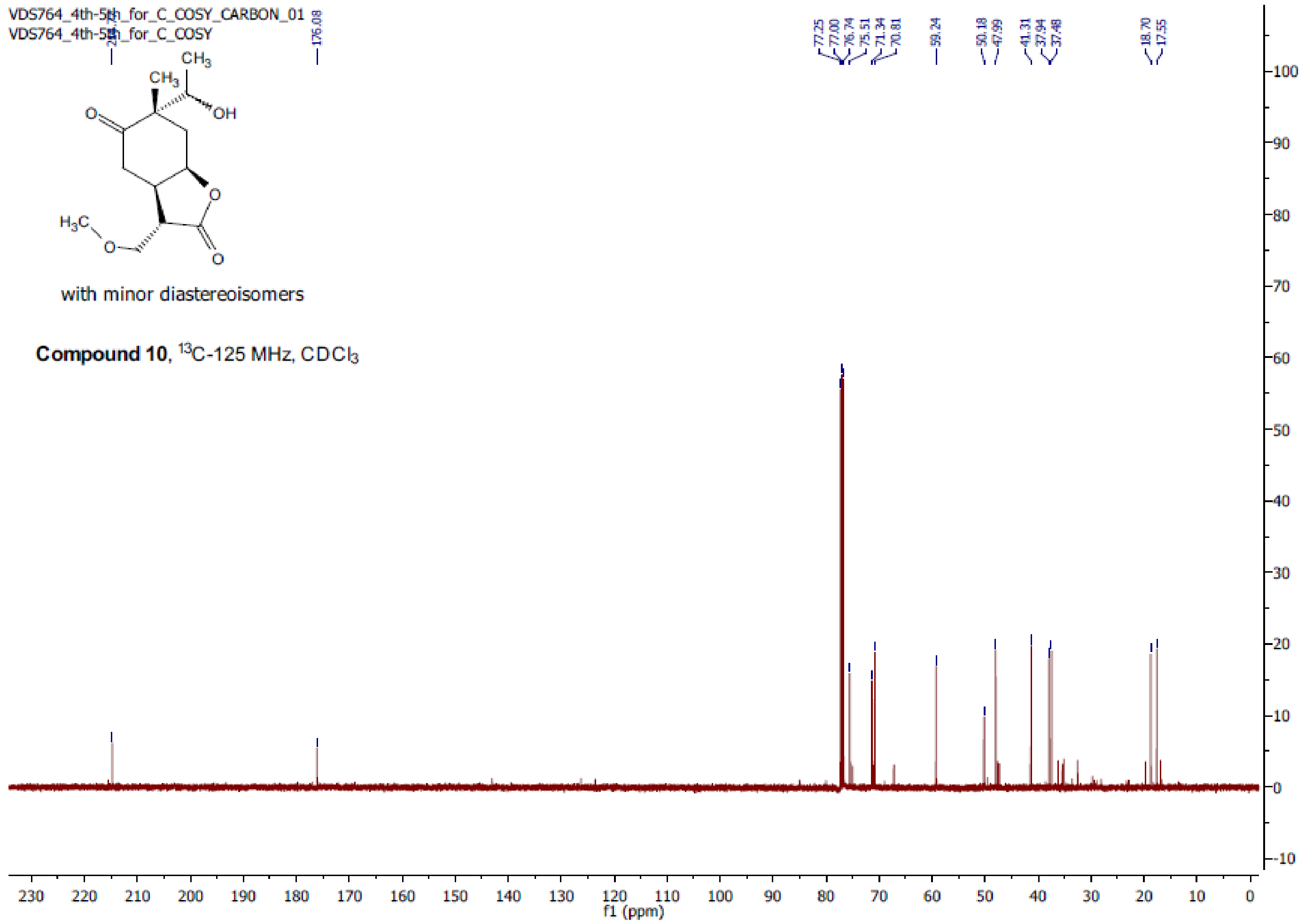
VDS764_4th-5th_for_C_COSY_CARBON_01
VDS764_4th-5th_for_C_COSY
with minor diastereoisomers
Compound 10, 13C-125 MHz, CDCl3
176.08
77.25
77.00
76.74
75.51
71.34
70.81
59.24
50.18
47.99
41.31
37.94
37.48
18.70
17.55
f1 (ppm)

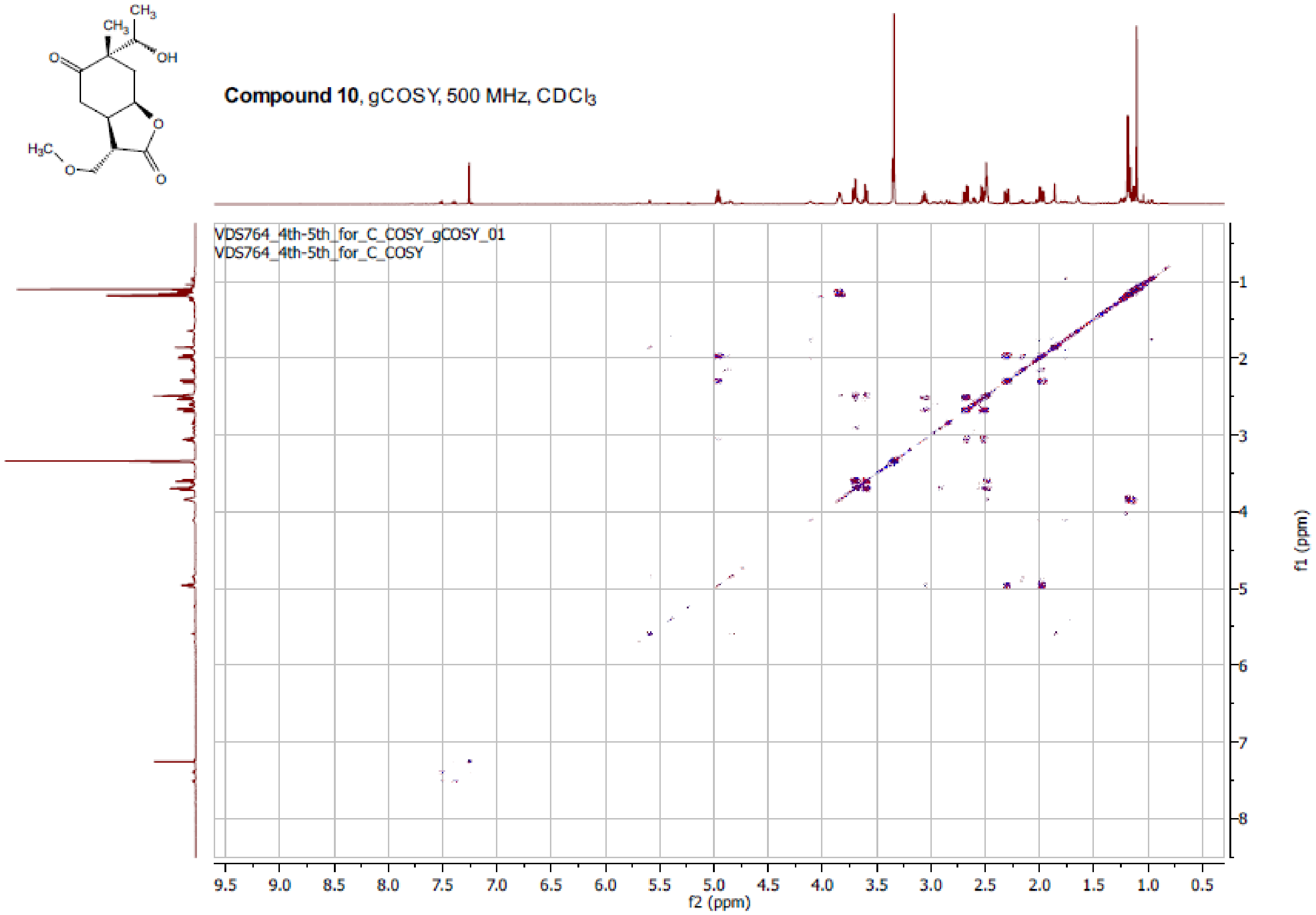
Compound 10, gCOSY, 500 MHz, CDCl3
VDS764_4th-5th_for_C_COSY_gCOSY_01
VDS764_4th-5th_for_C_COSY
f2 (ppm)
f1 (ppm)

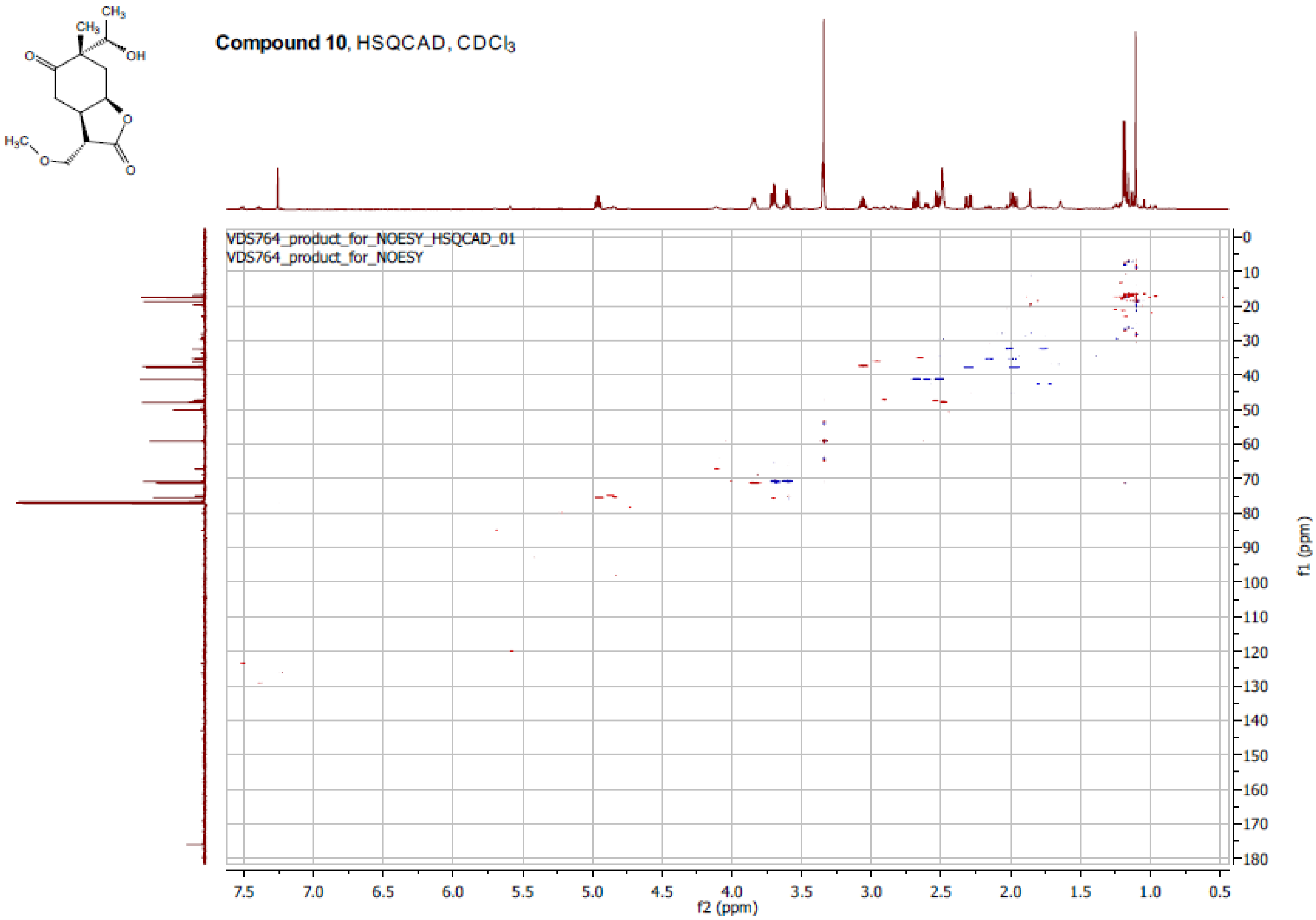
Compound 10, HSQCAD, CDCl3
VDS764_product_for_NOESY_HSQCAD_01
VDS764_product_for_NOESY
f2 (ppm)
f1 (ppm)

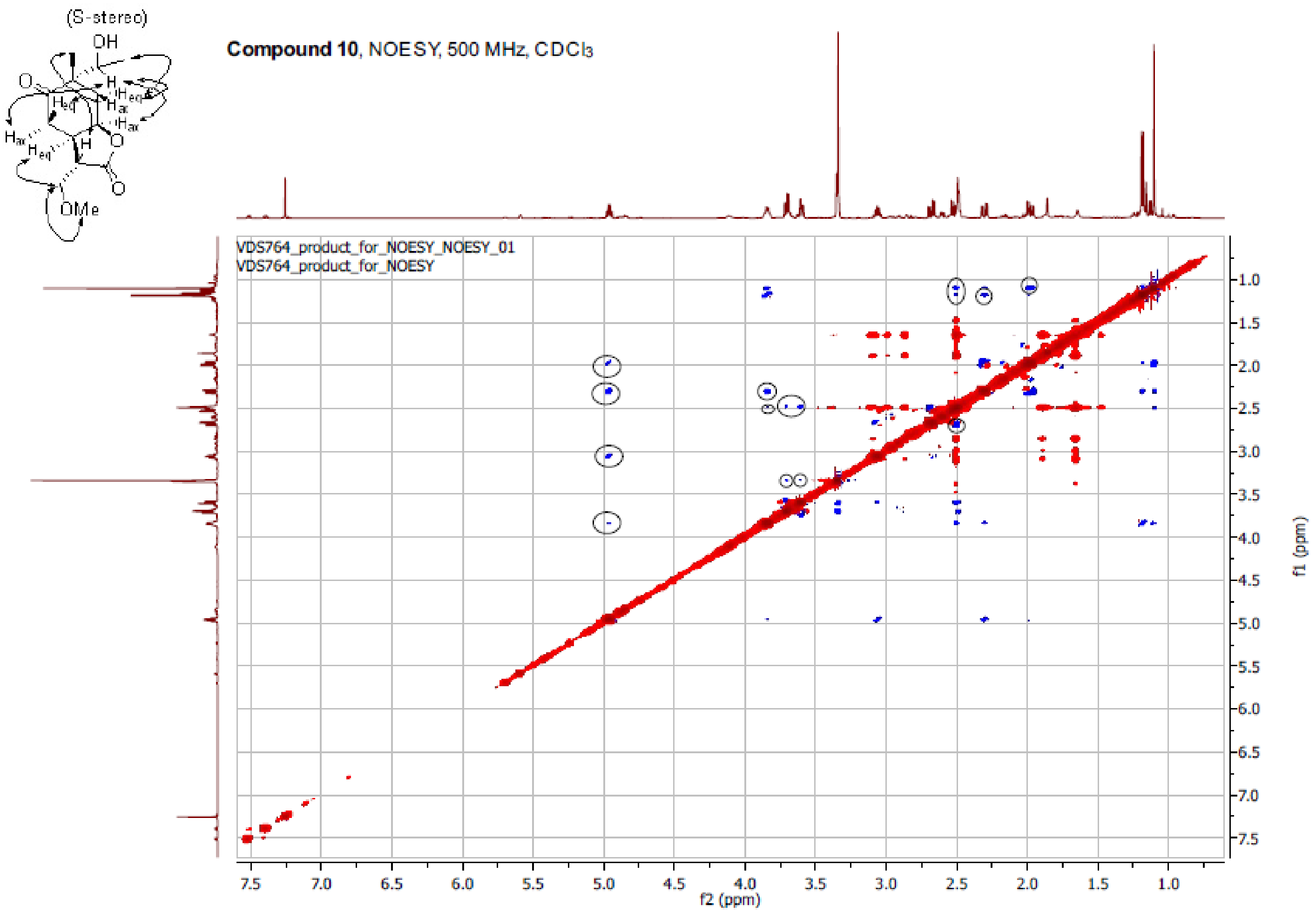
(S-stereo)
Compound 10, NOESY, 500 MHz, CDCl3
VDS764_product_for_NOESY_NOESY_01
VDS764_product_for_NOESY
f2 (ppm)
f1 (ppm)

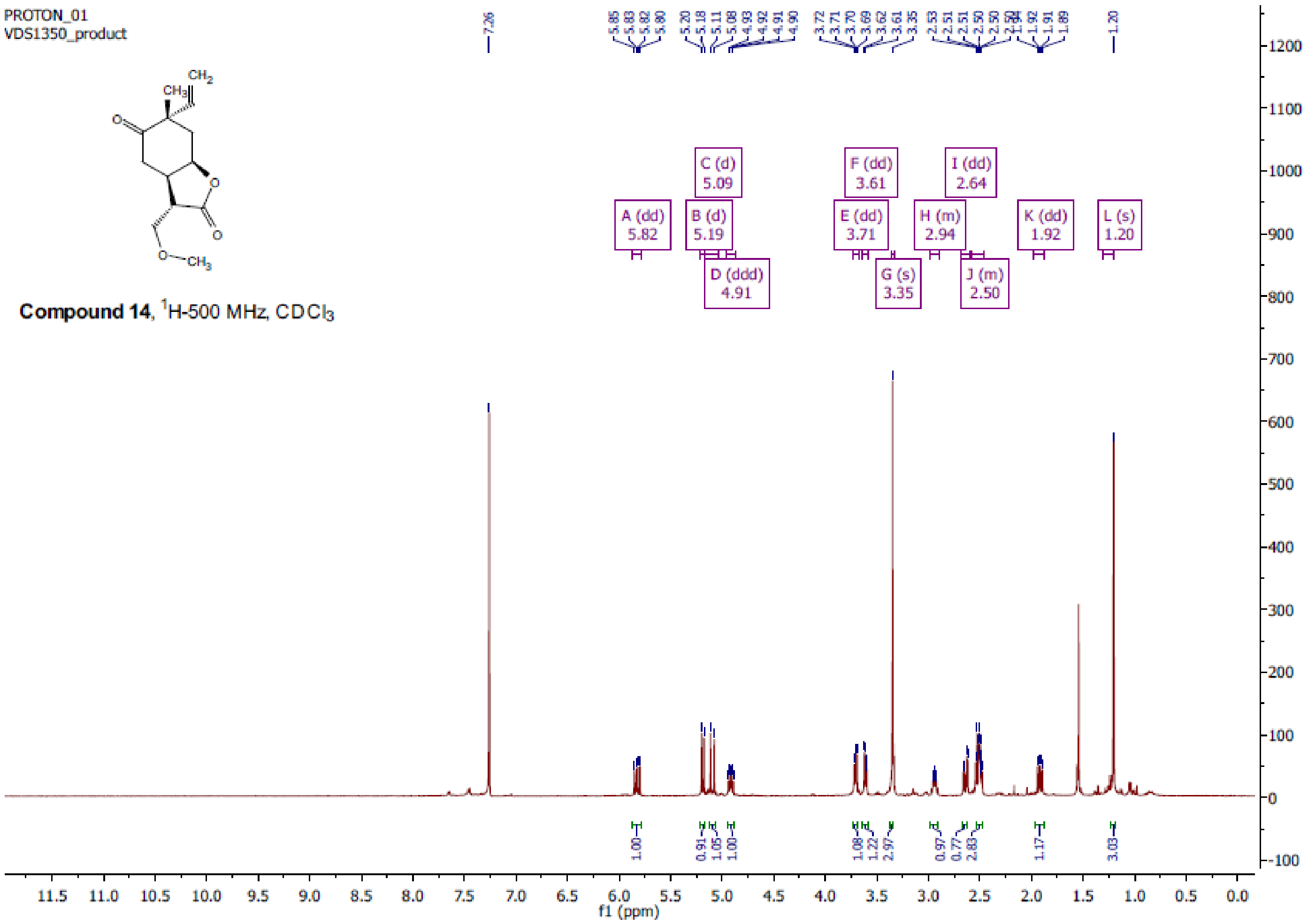
PROTON_01
VDS1350_product
Compound 14, 1H-500 MHz, CDCl3
A (dd) 5.82
B (d) 5.19
C (d) 5.09
D (ddd) 4.91
E (dd) 3.71
F (dd) 3.61
G (s) 3.35
H (m) 2.94
I (dd) 2.64
J (m) 2.50
K (dd) 1.92
L (s) 1.20
f1 (ppm)

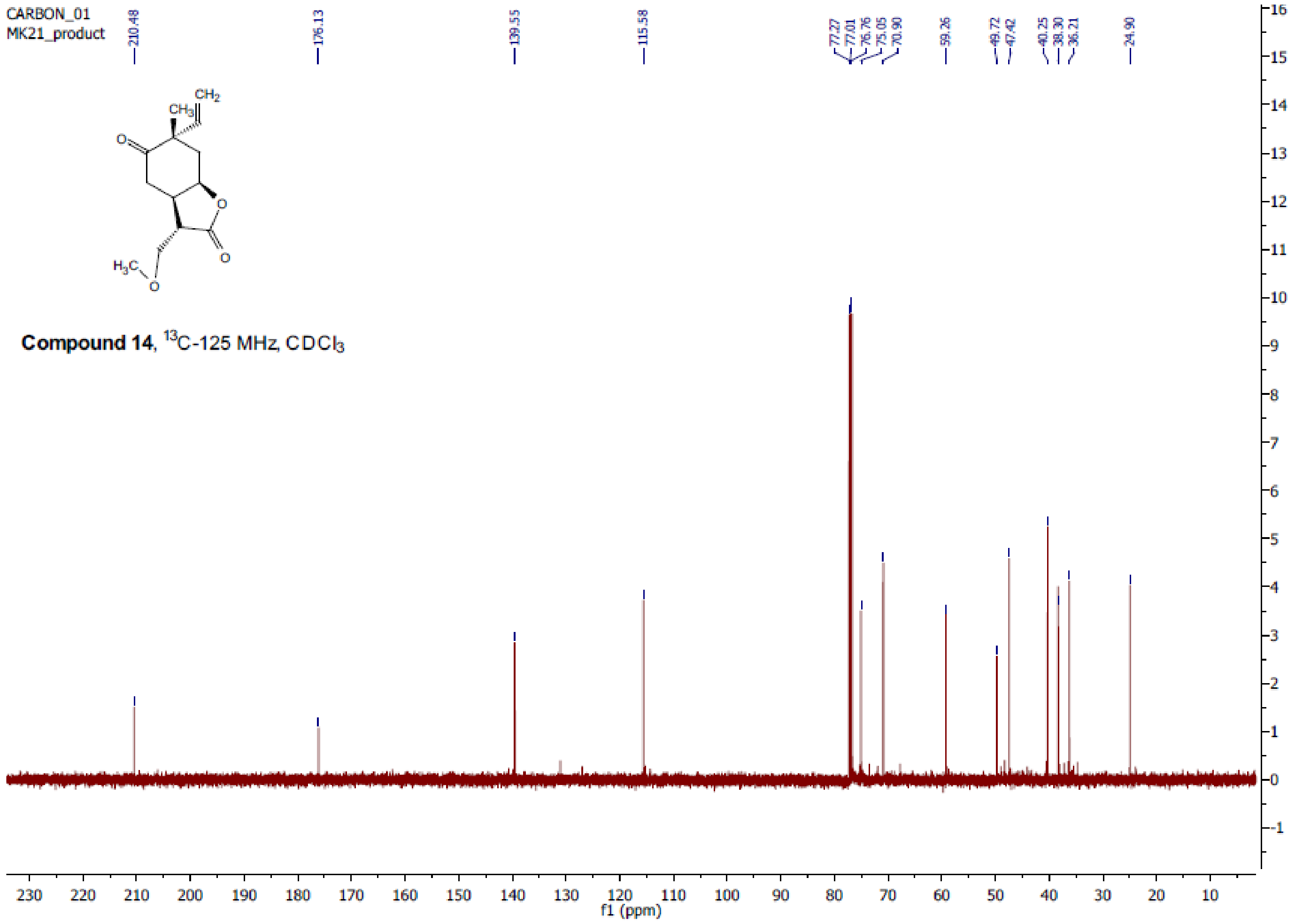
CARBON_01
MK21_product
210.48
176.13
139.55
115.58
77.27
77.01
76.76
75.05
70.90
59.26
49.72
47.42
40.25
38.30
36.21
24.90
Compound 14, 13C-125 MHz, CDCl3
f1 (ppm)

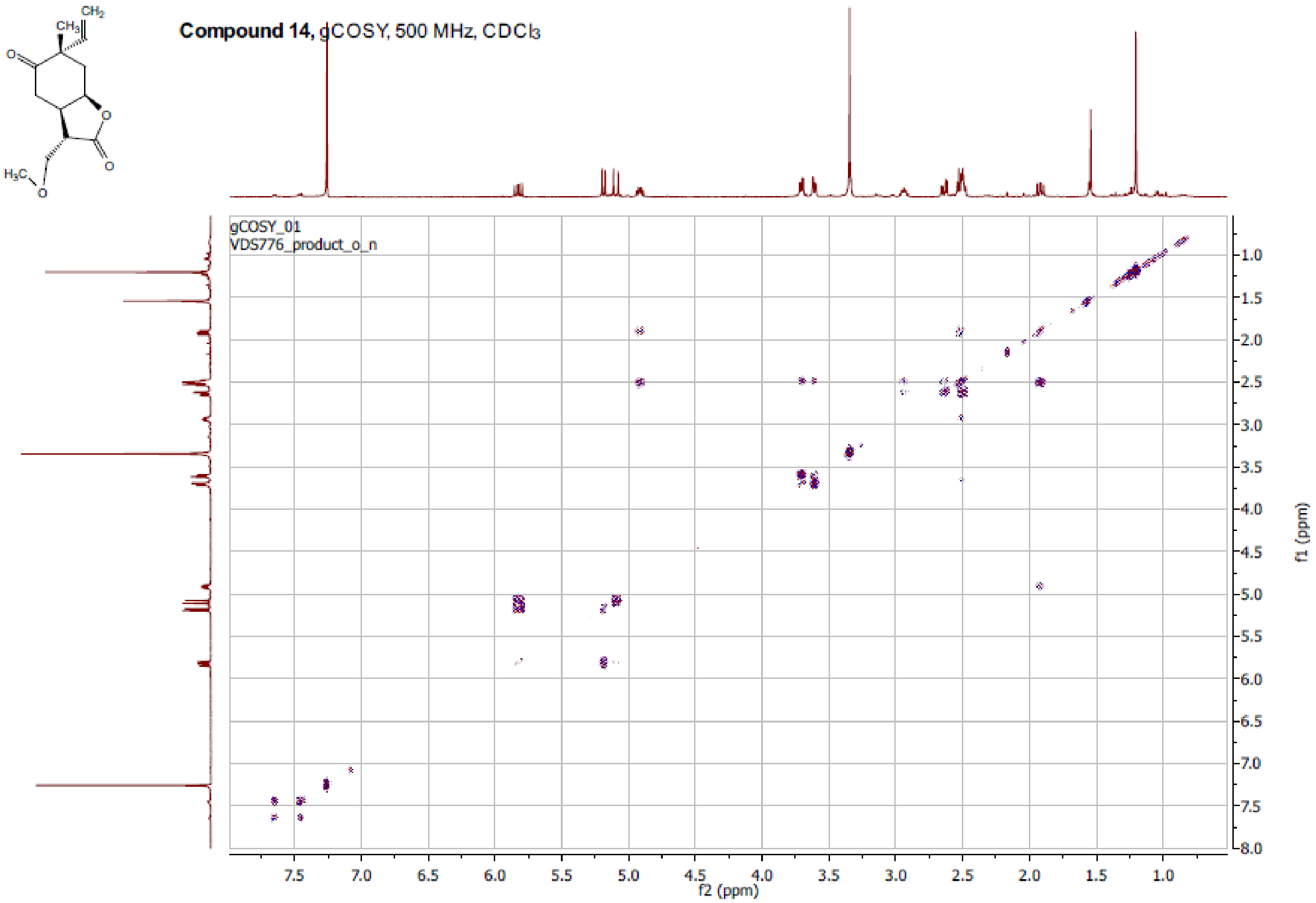
Compound 14, gCOSY, 500 MHz, CDCl3
gCOSY_01
VDS776_product_o_n
f2 (ppm)
f1 (ppm)

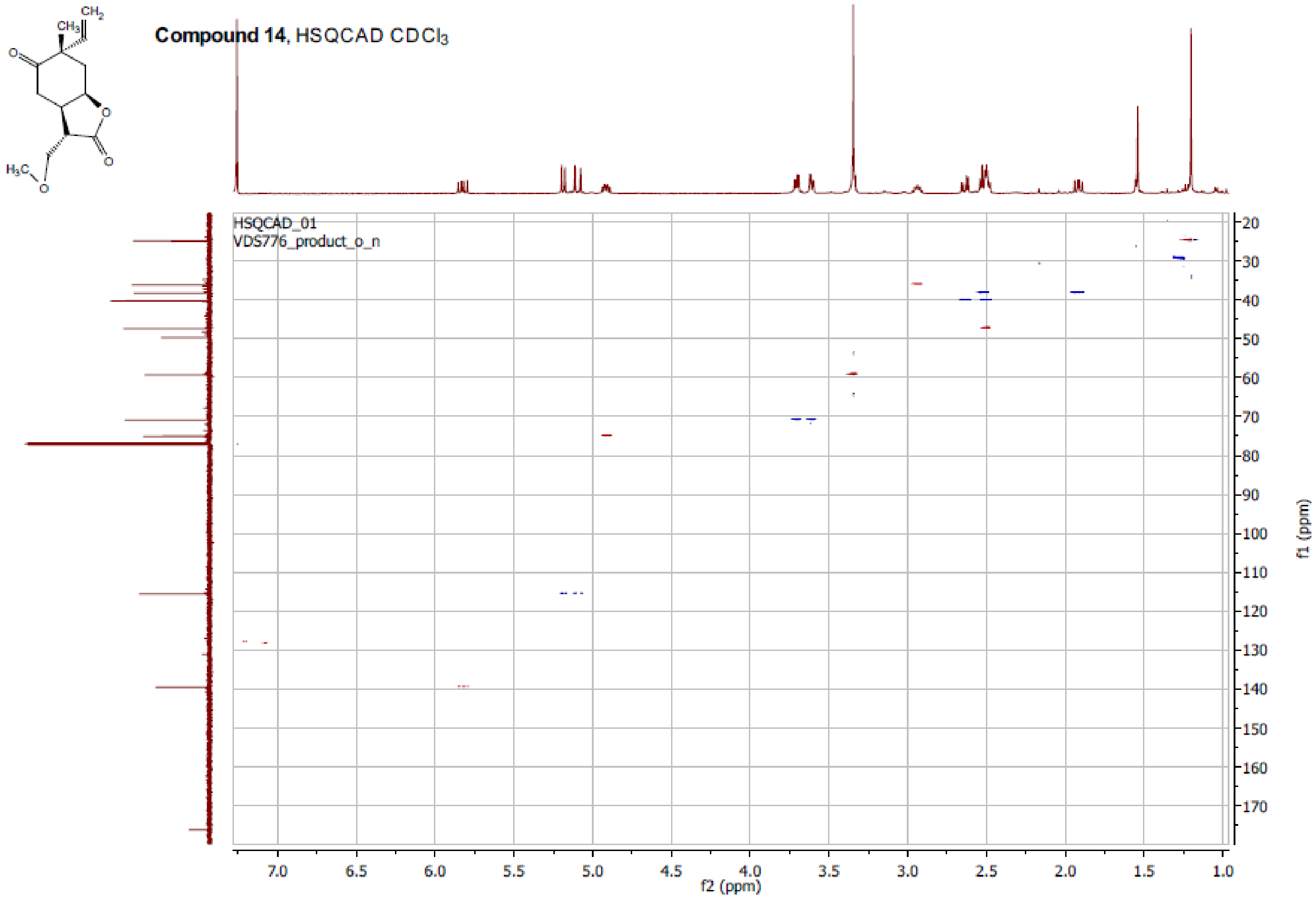
Compound 14, HSQCAD CDCl3
HSQCAD_01
VDS776_product_o_n
f2 (ppm)
f1 (ppm)

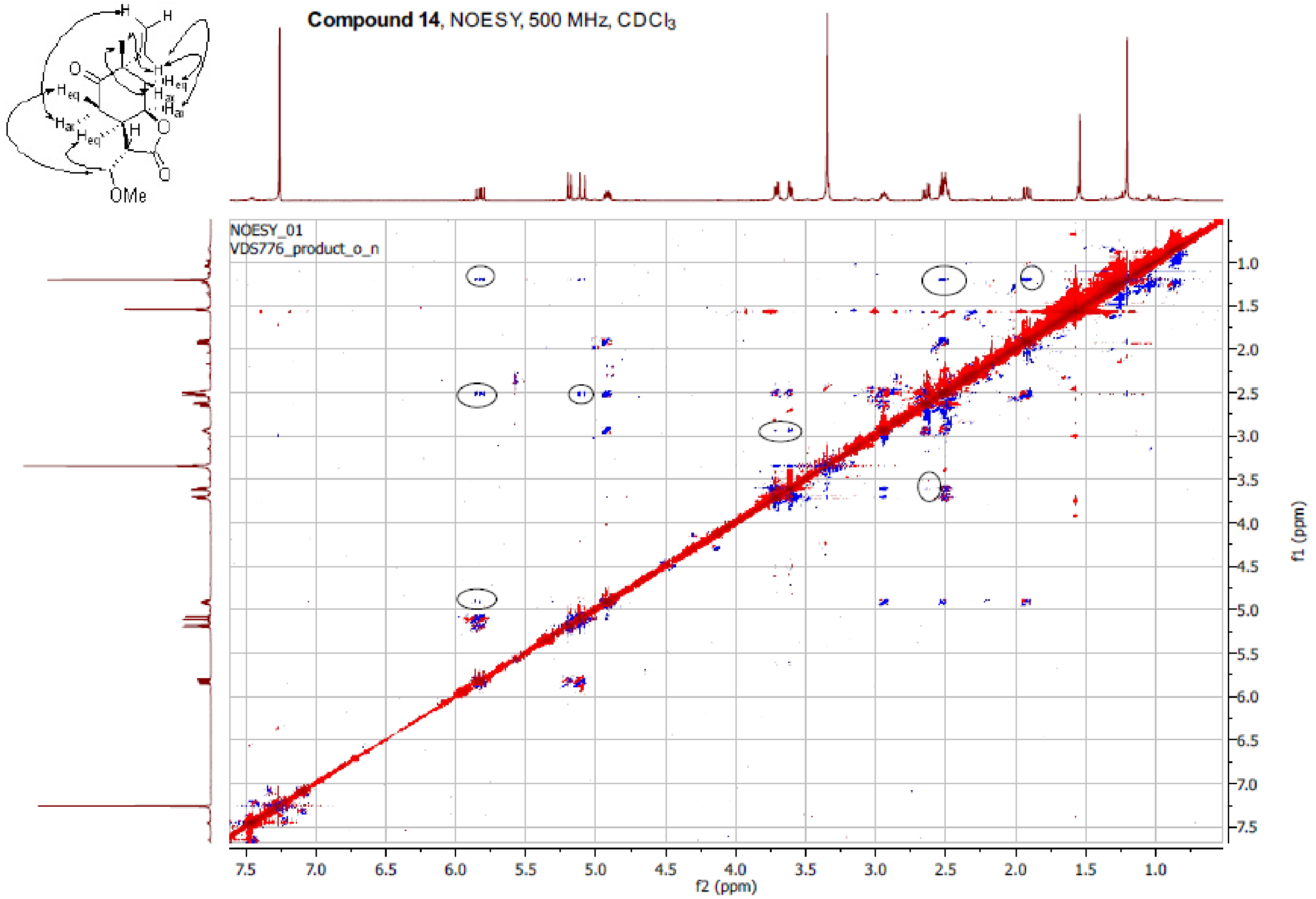
Compound 14, NOESY, 500 MHz, CDCl3
NOESY_01
VDS776_product_o_n
OMe
f2 (ppm)
f1 (ppm)

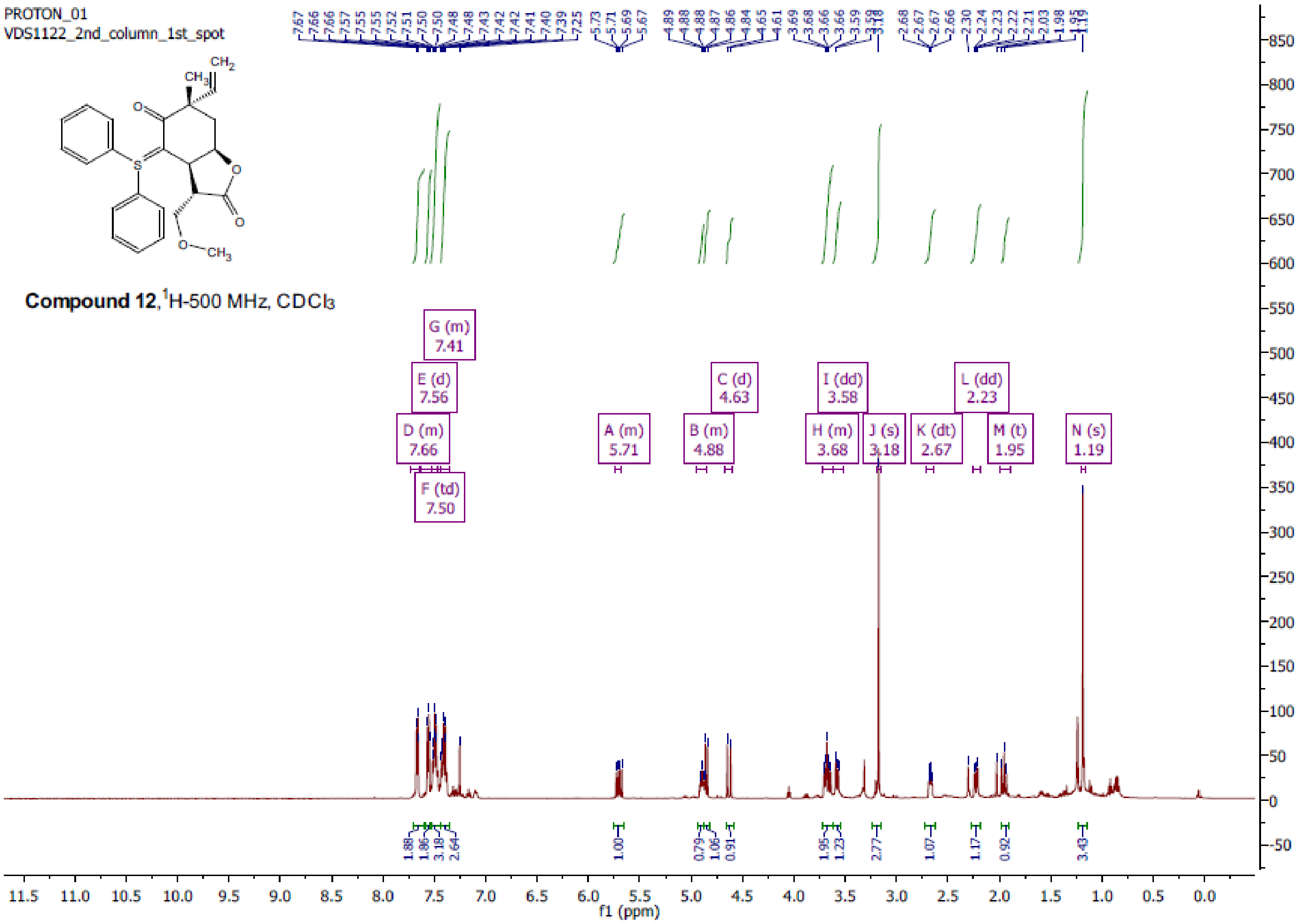
PROTON_01
VDS1122_2nd_column_1st_spot
Compound 12, 1H-500 MHz, CDCl3
G (m) 7.41
E (d) 7.56
D (m) 7.66
F (td) 7.50
A (m) 5.71
B (m) 4.88
C (d) 4.63
H (m) 3.68
I (dd) 3.58
J (s) 3.18
K (dt) 2.67
L (dd) 2.23
M (t) 1.95
N (s) 1.19
f1 (ppm)

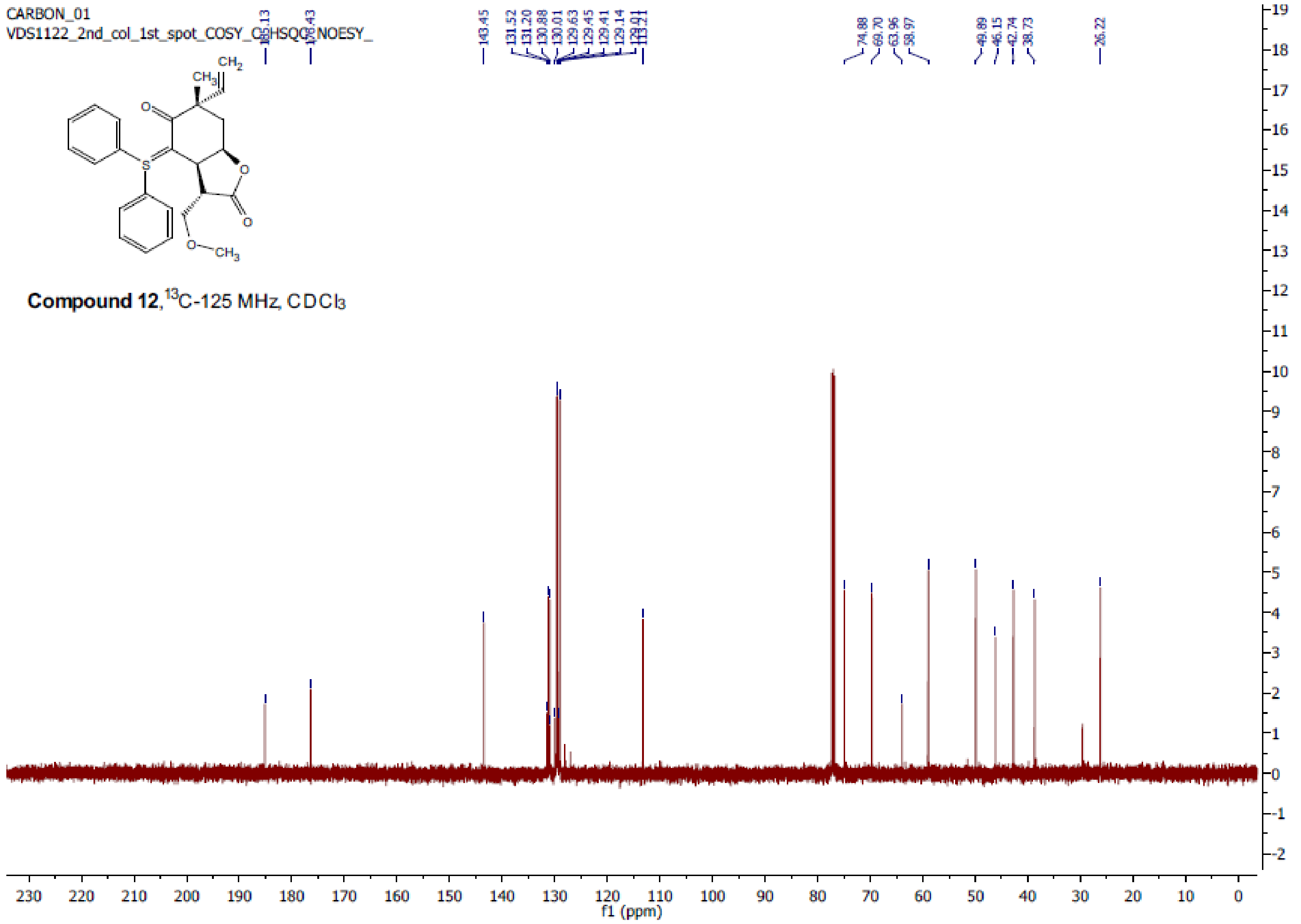
CARBON_01
VDS1122_2nd_col_1st_spot_COSY_C13HSQC_NOESY_
Compound 12, 13C-125 MHz, CDCl3
143.45
131.52
131.20
130.88
130.01
129.63
129.45
129.41
129.14
113.21
74.88
69.70
63.96
58.97
49.89
46.15
42.74
38.73
26.22
f1 (ppm)

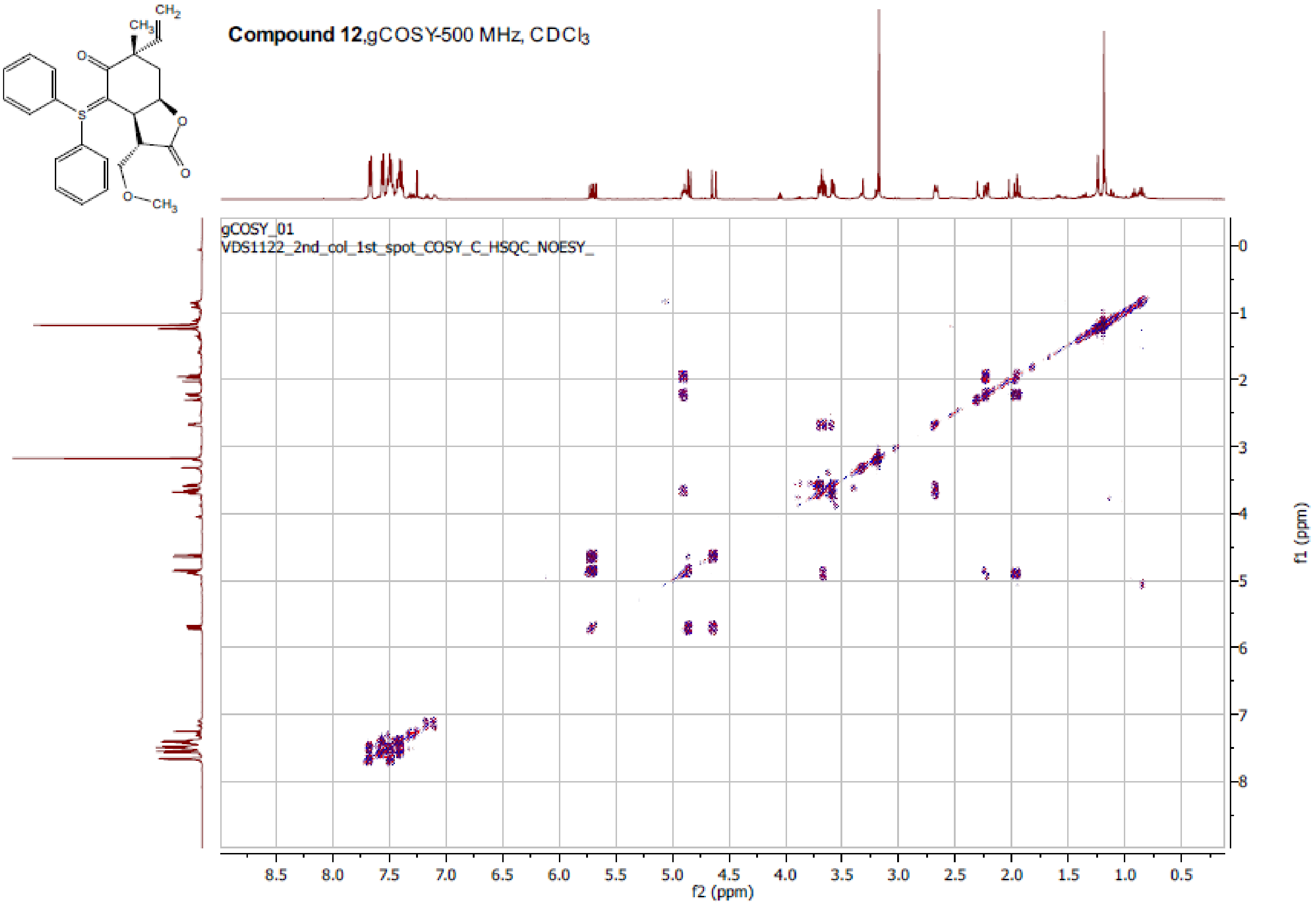
Compound 12,gCOSY-500 MHz, CDCl3
gCOSY_01
VDS1122_2nd_col_1st_spot_COSY_C_HSQC_NOESY_
f2 (ppm)
f1 (ppm)

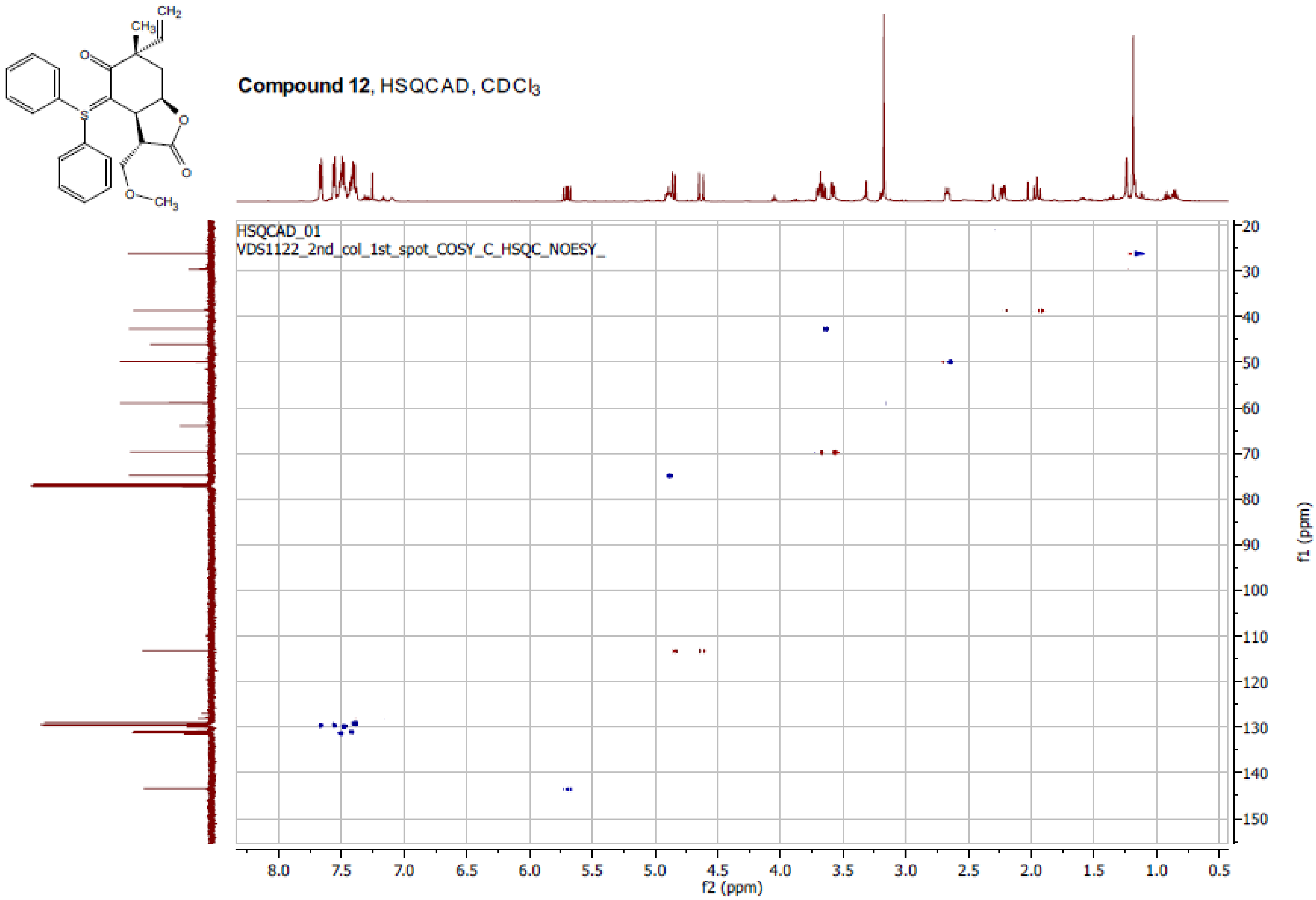
Compound 12, HSQCAD, CDCl3
HSQCAD_01
VDS1122_2nd_col_1st_spot_COSY_C_HSQC_NOESY_
f2 (ppm)
f1 (ppm)

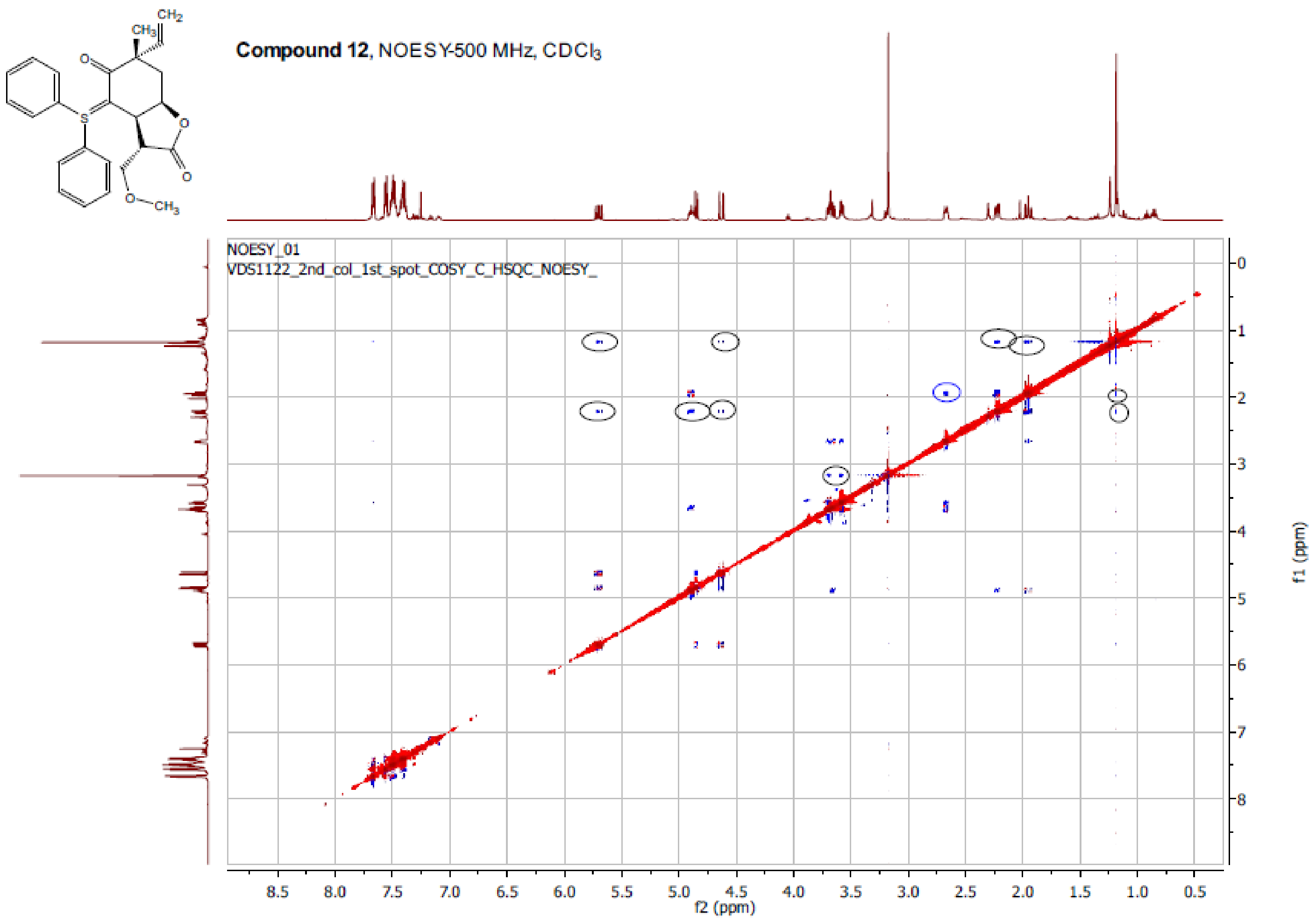
Compound 12, NOESY-500 MHz, CDCl3
NOESY_01
VDS1122_2nd_col_1st_spot_COSY_C_HSQC_NOESY_
f2 (ppm)
f1 (ppm)

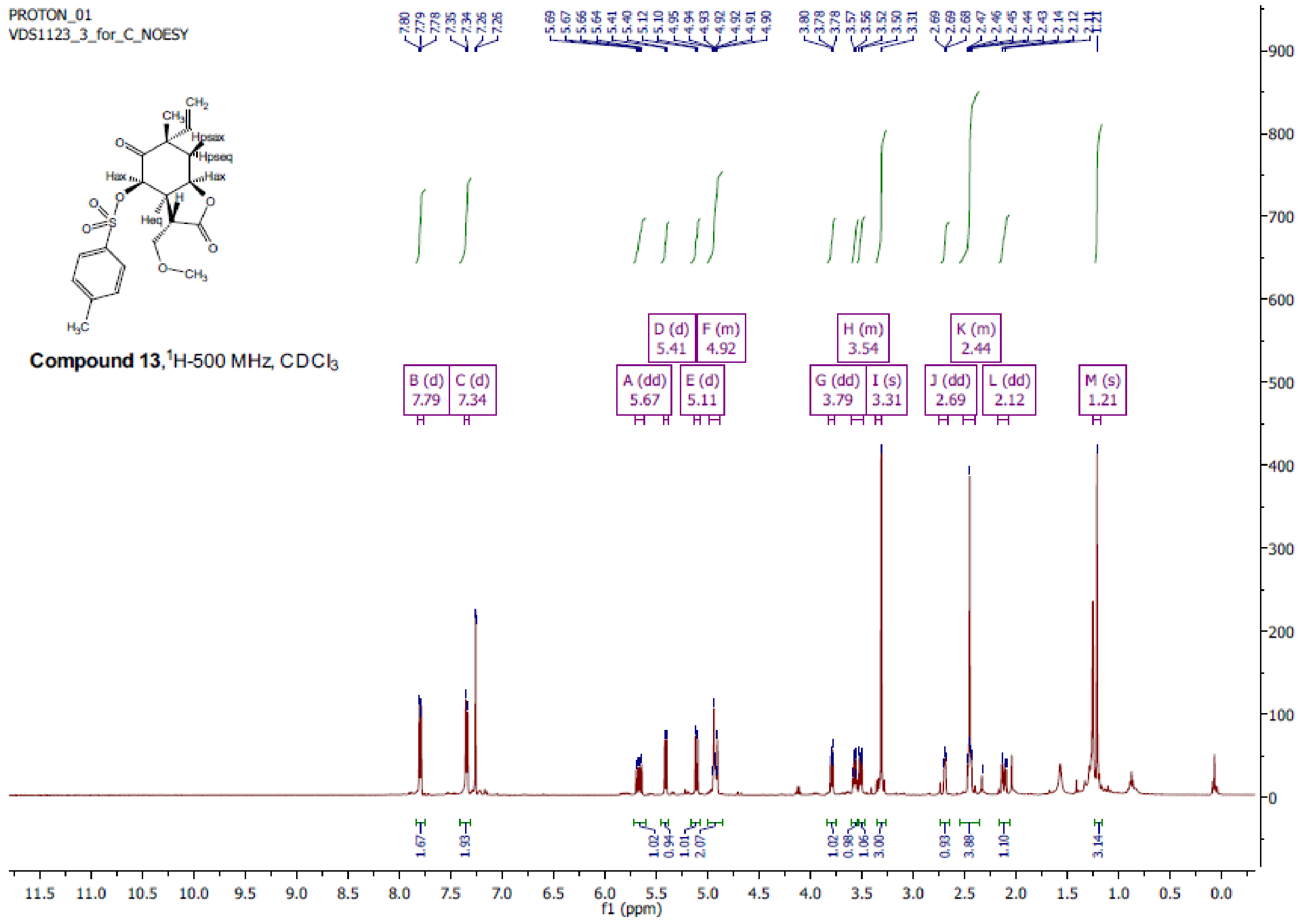
PROTON_01
VDS1123_3_for_C_NOESY
Compound 13, 1H-500 MHz, CDCl3
B (d) 7.79
C (d) 7.34
A (dd) 5.67
D (d) 5.41
E (d) 5.11
F (m) 4.92
G (dd) 3.79
H (m) 3.54
I (s) 3.31
J (dd) 2.69
K (m) 2.44
L (dd) 2.12
M (s) 1.21
f1 (ppm)

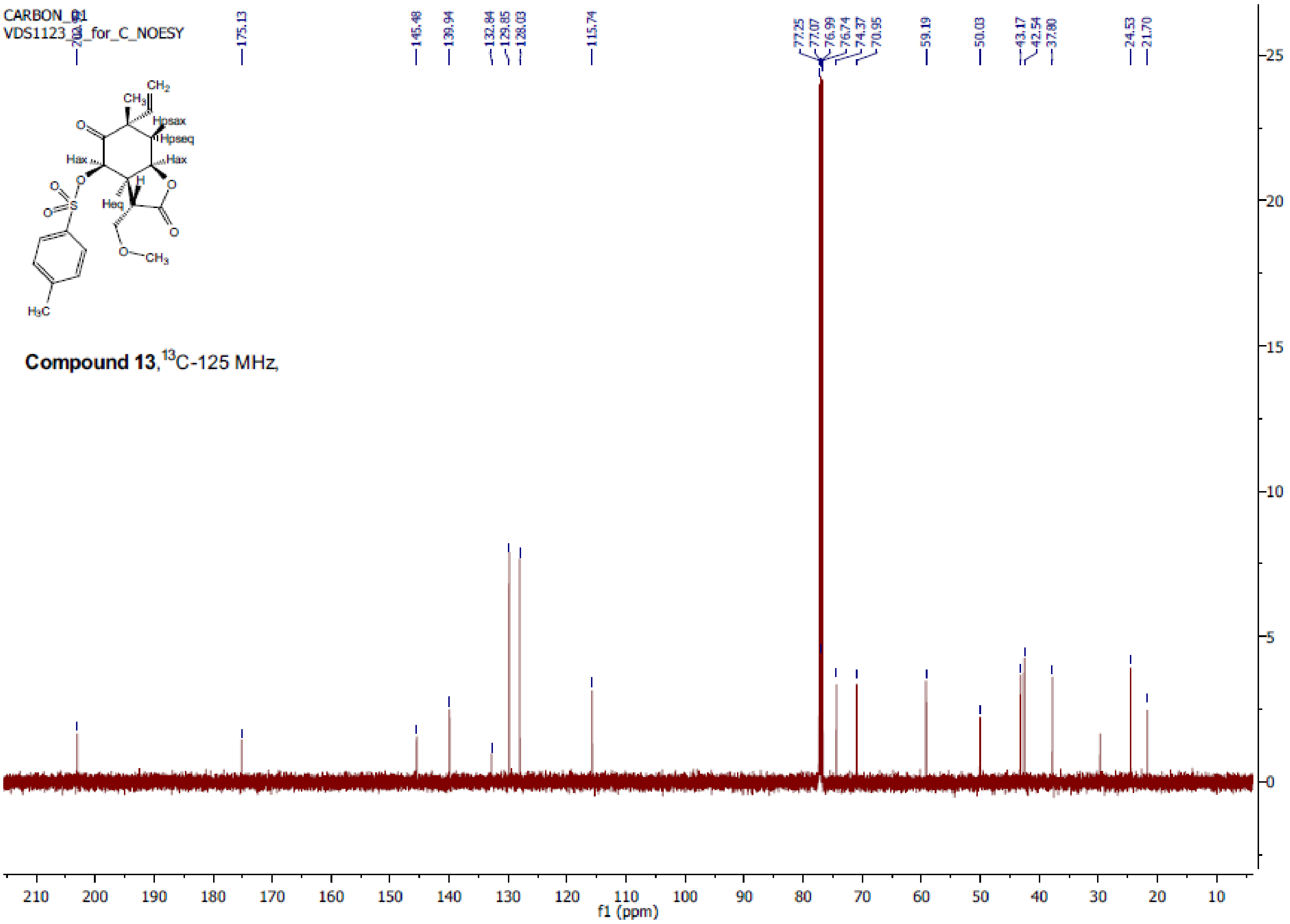
CARBON_01
VDS1123_3_for_C_NOESY
175.13
145.48
139.94
132.84
129.85
128.03
115.74
77.25
77.07
76.99
76.74
74.37
70.95
59.19
50.03
43.17
42.54
37.80
24.53
21.70
Compound 13, 13C-125 MHz,
f1 (ppm)

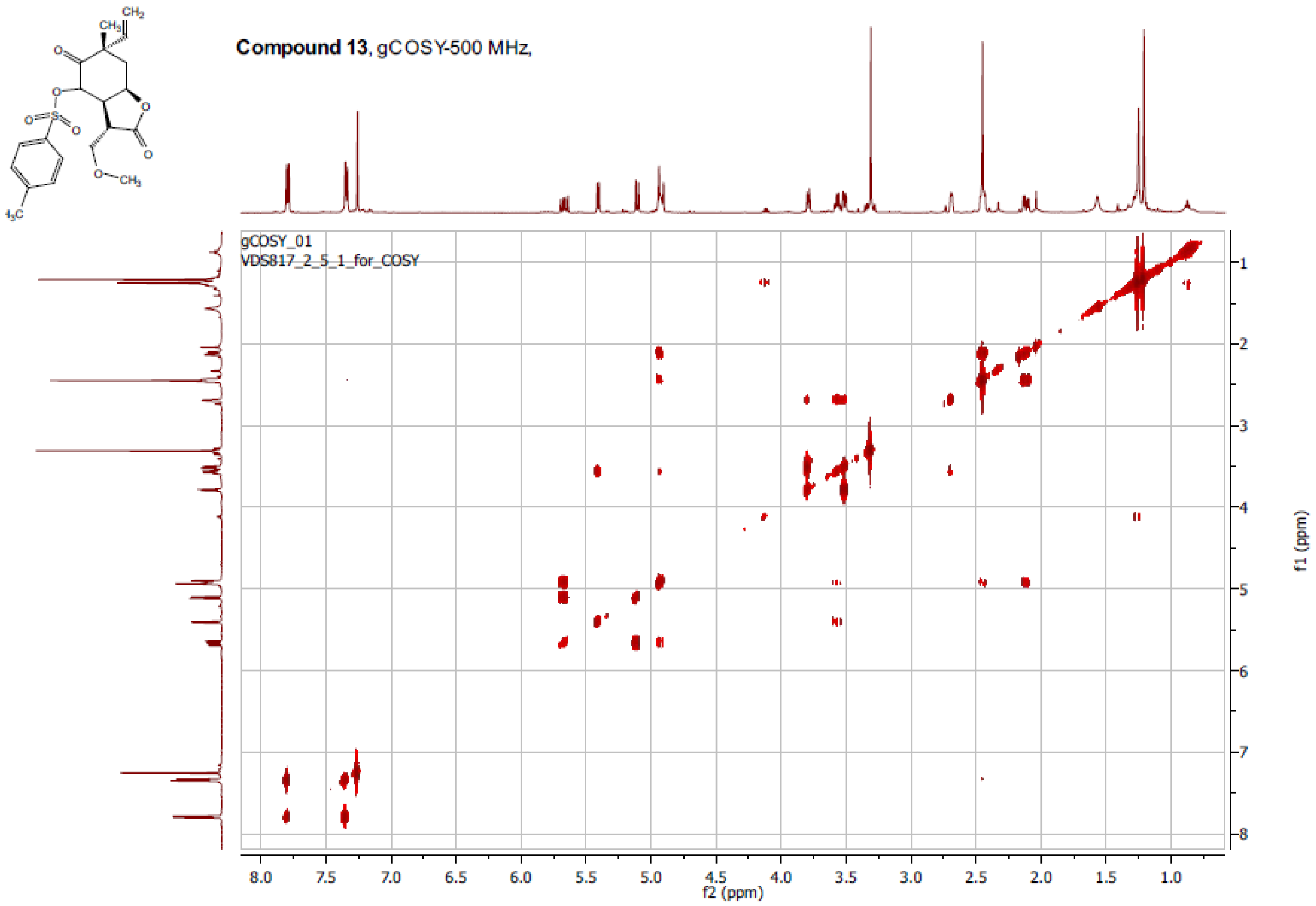
Compound 13, gCOSY-500 MHz,
gCOSY_01
VDS817_2_5_1_for_COSY
f2 (ppm)
f1 (ppm)

Compound 13, HSQCAD, $CDCl_3$

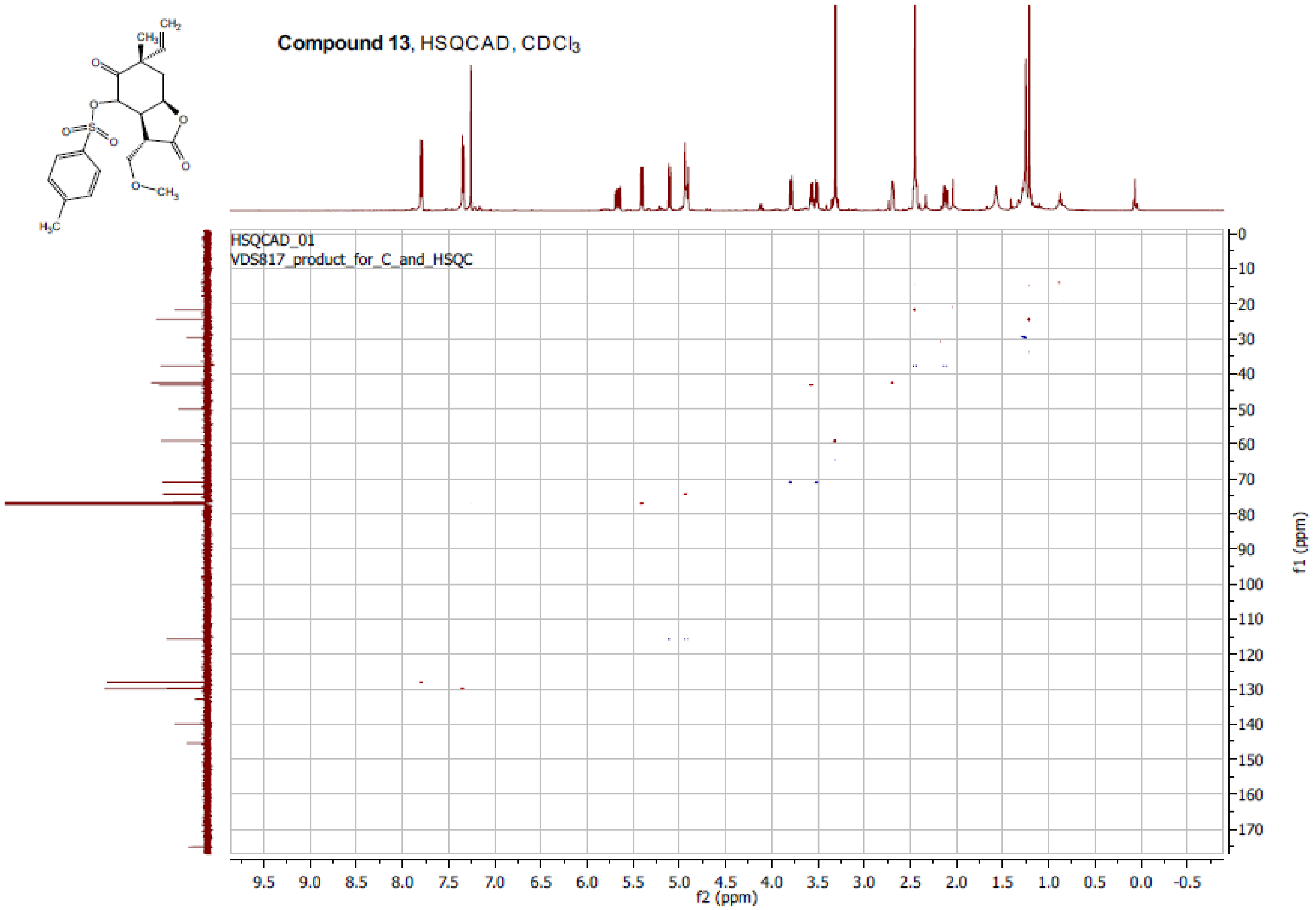

Compound 13, NOESY-500 MHz,

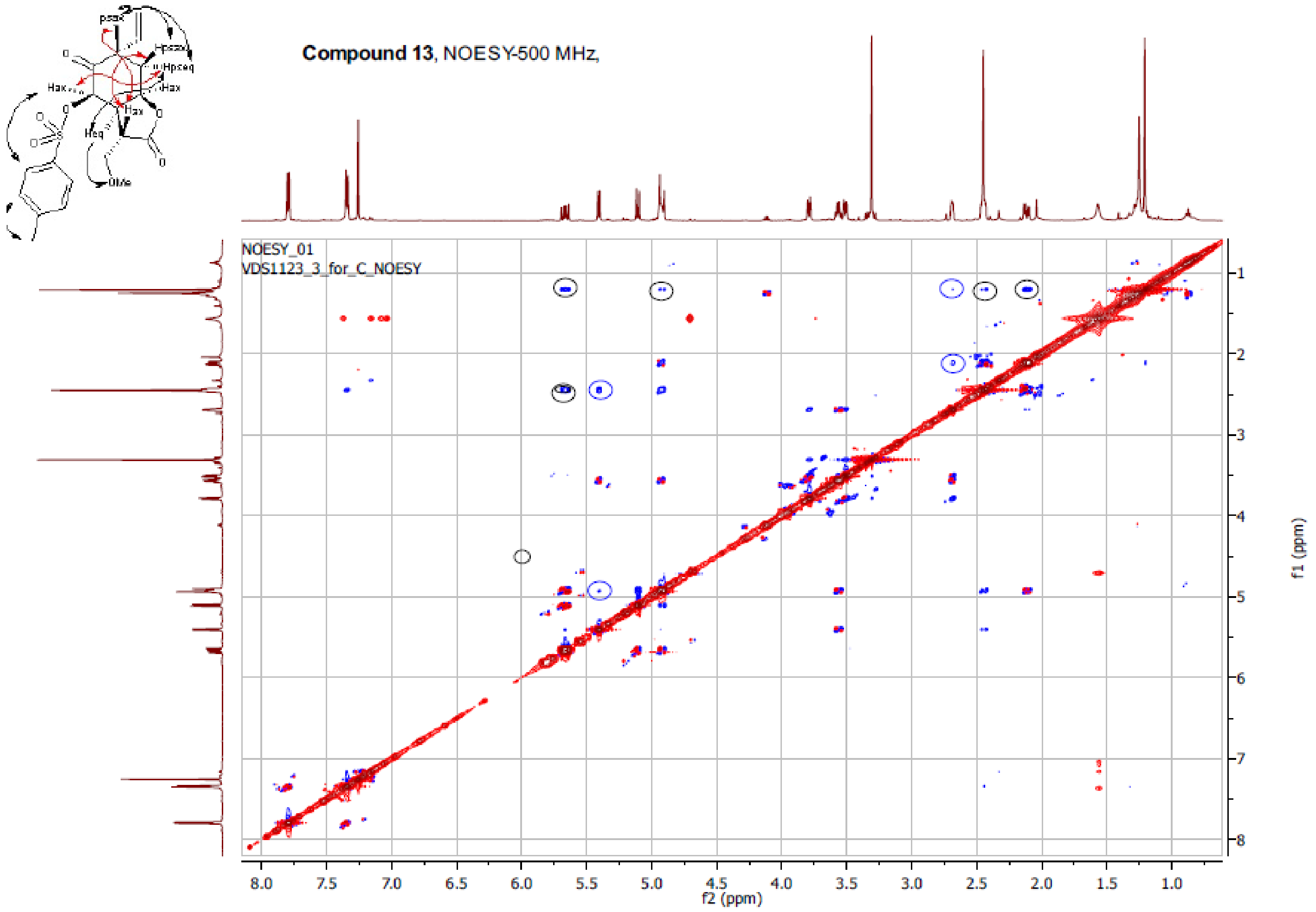

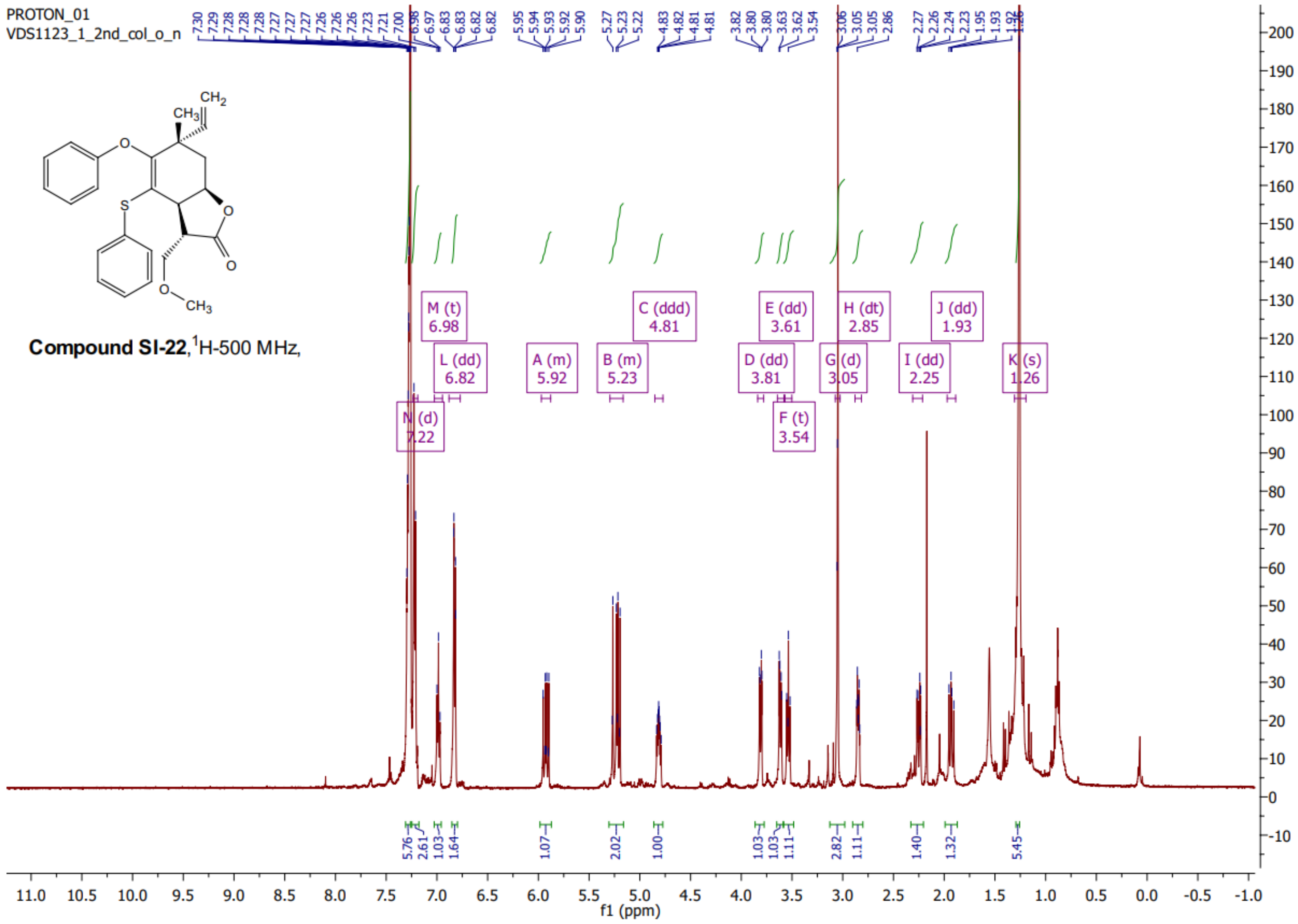

Compound SI-22, ^{1}H-500 MHz,

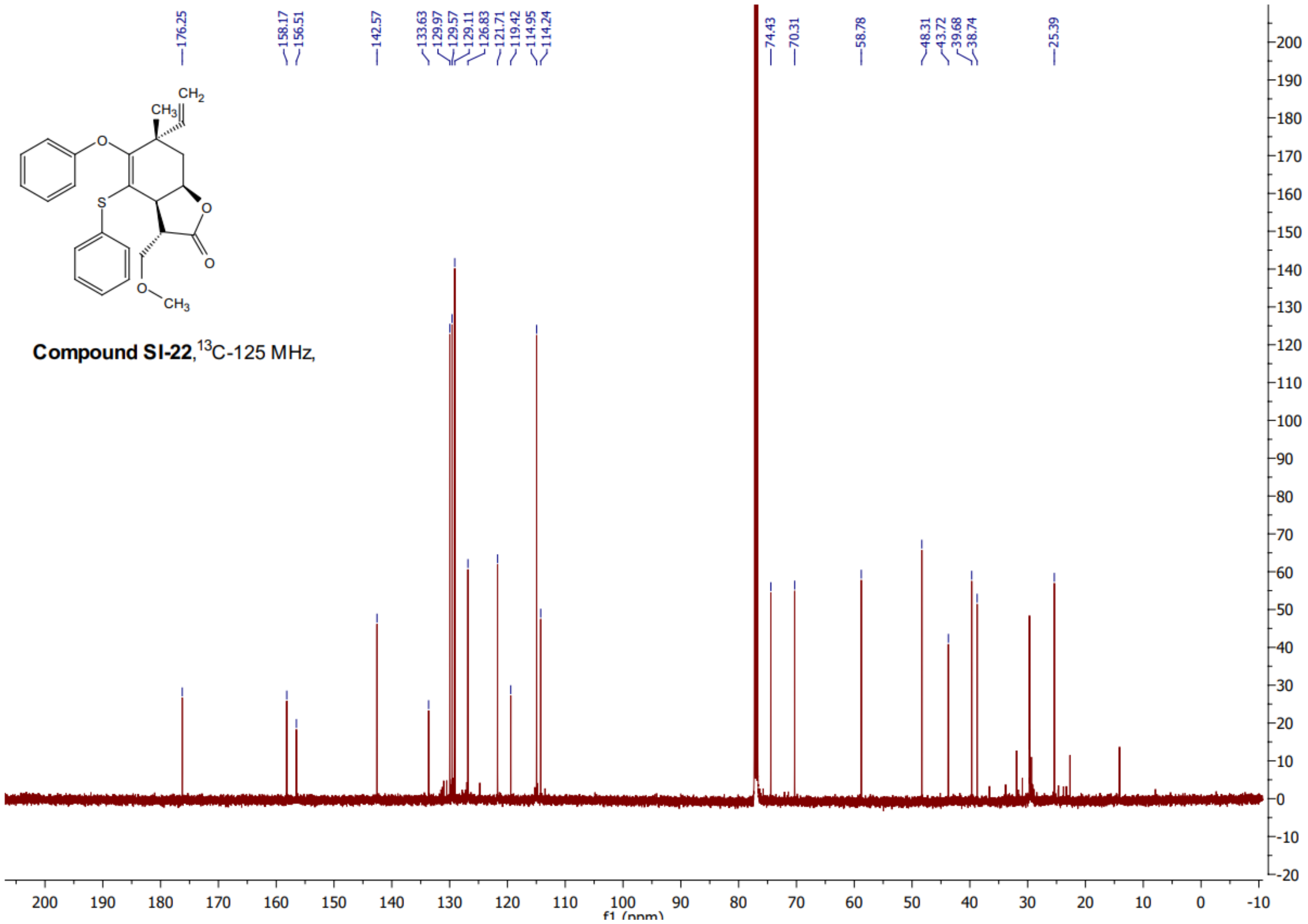

Compound SI-22, ^{13}C-125 MHz,

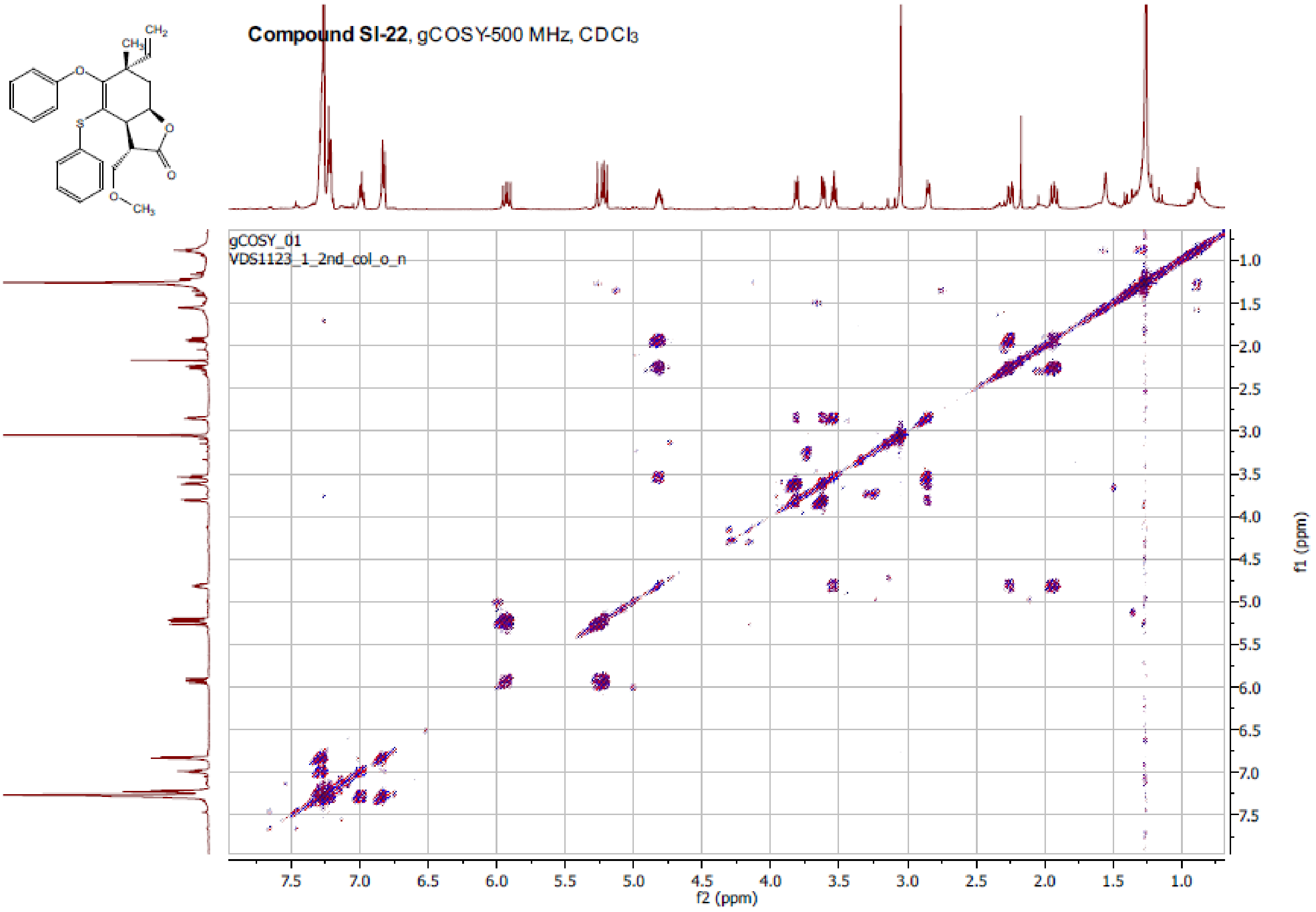
Compound SI-22, gCOSY-500 MHz, CDCl3
gCOSY_01
VDS1123_1_2nd_col_o_n
f2 (ppm)
f1 (ppm)

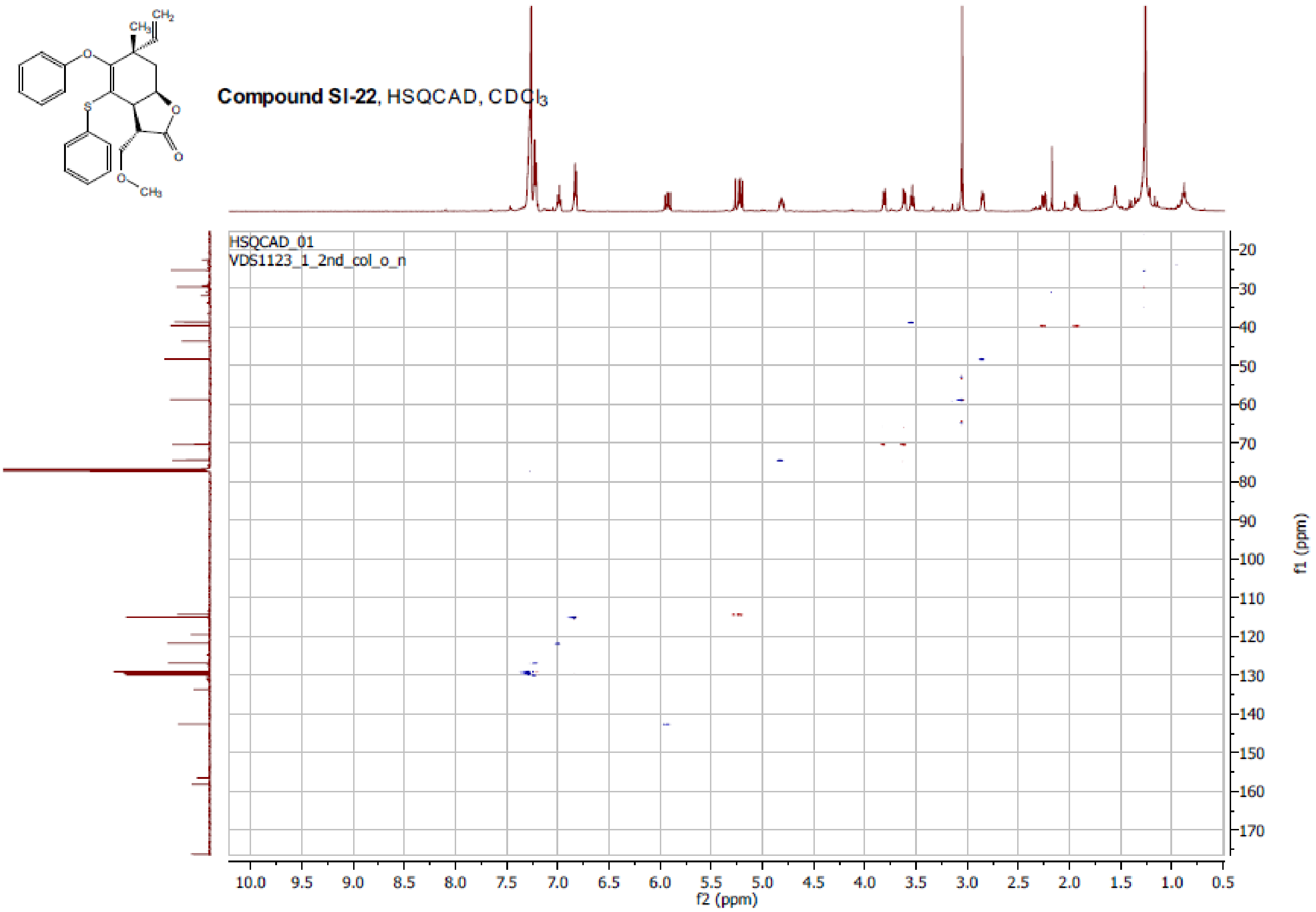
Compound SI-22, HSQCAD, CDCl3
HSQCAD_01
VDS1123_1_2nd_col_o_n
f2 (ppm)
f1 (ppm)

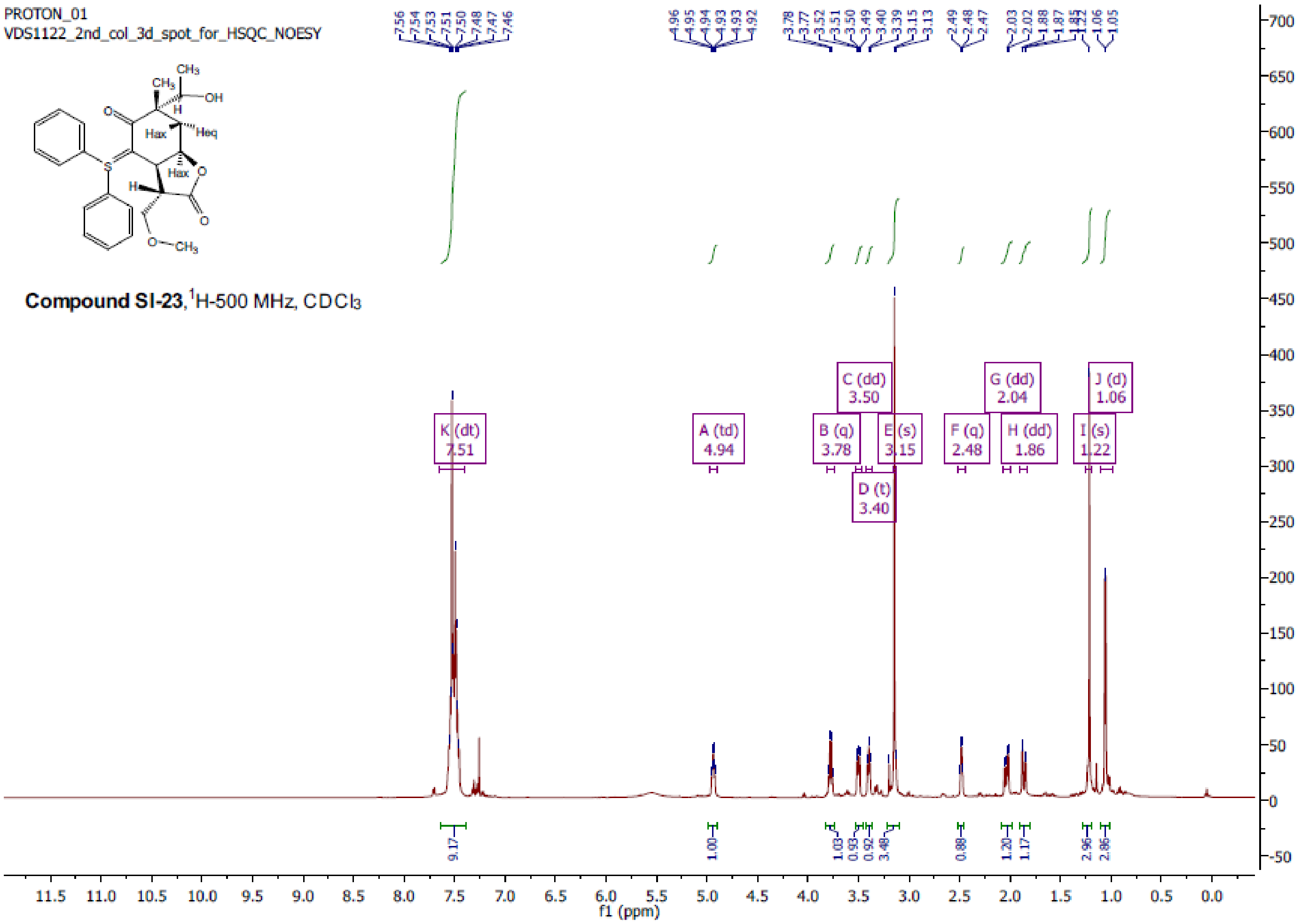

Compound SI-23, ^{1}H-500 MHz, $CDCl_3$

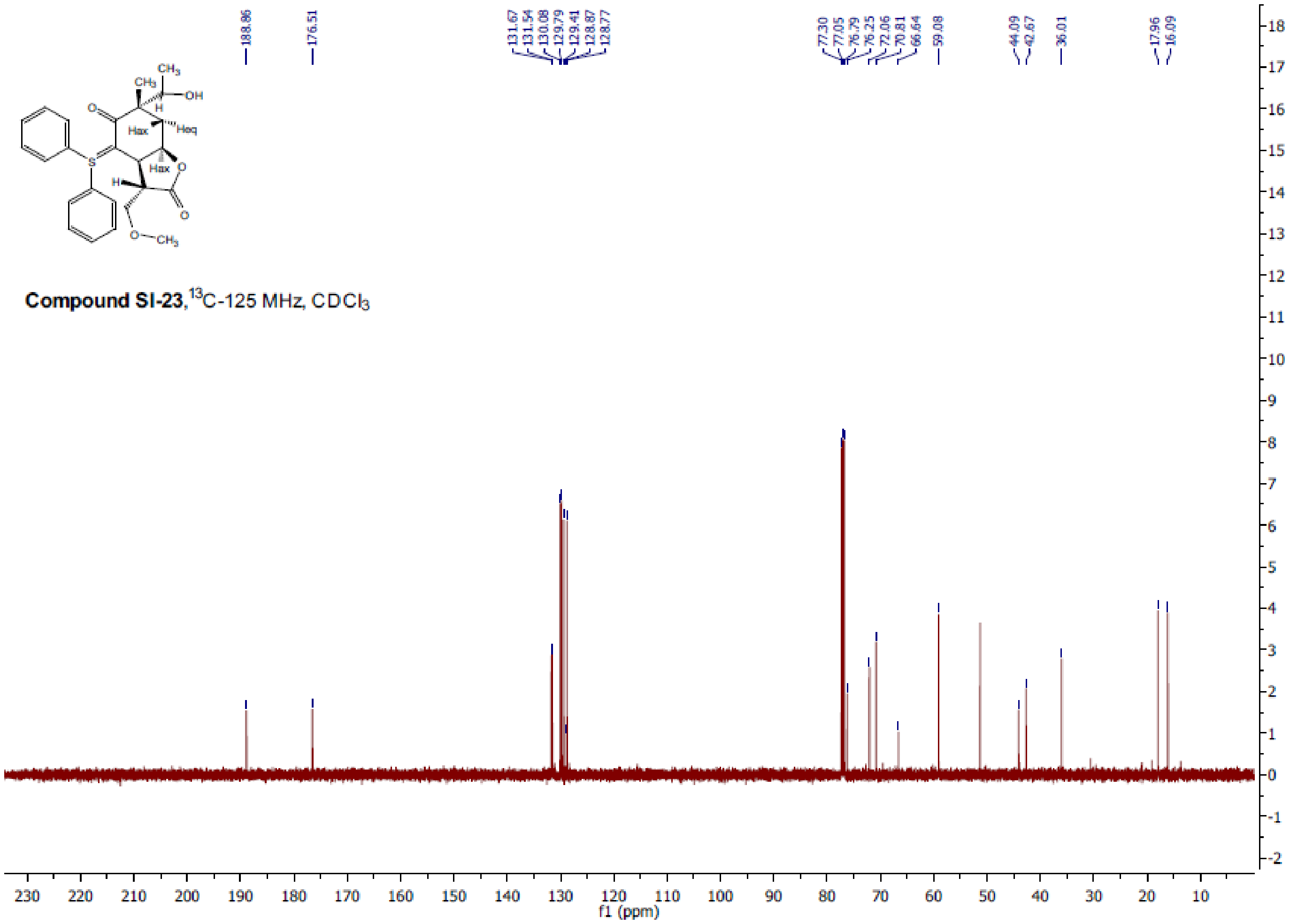

Compound SI-23, ^{13}C-125 MHz, $CDCl_3$

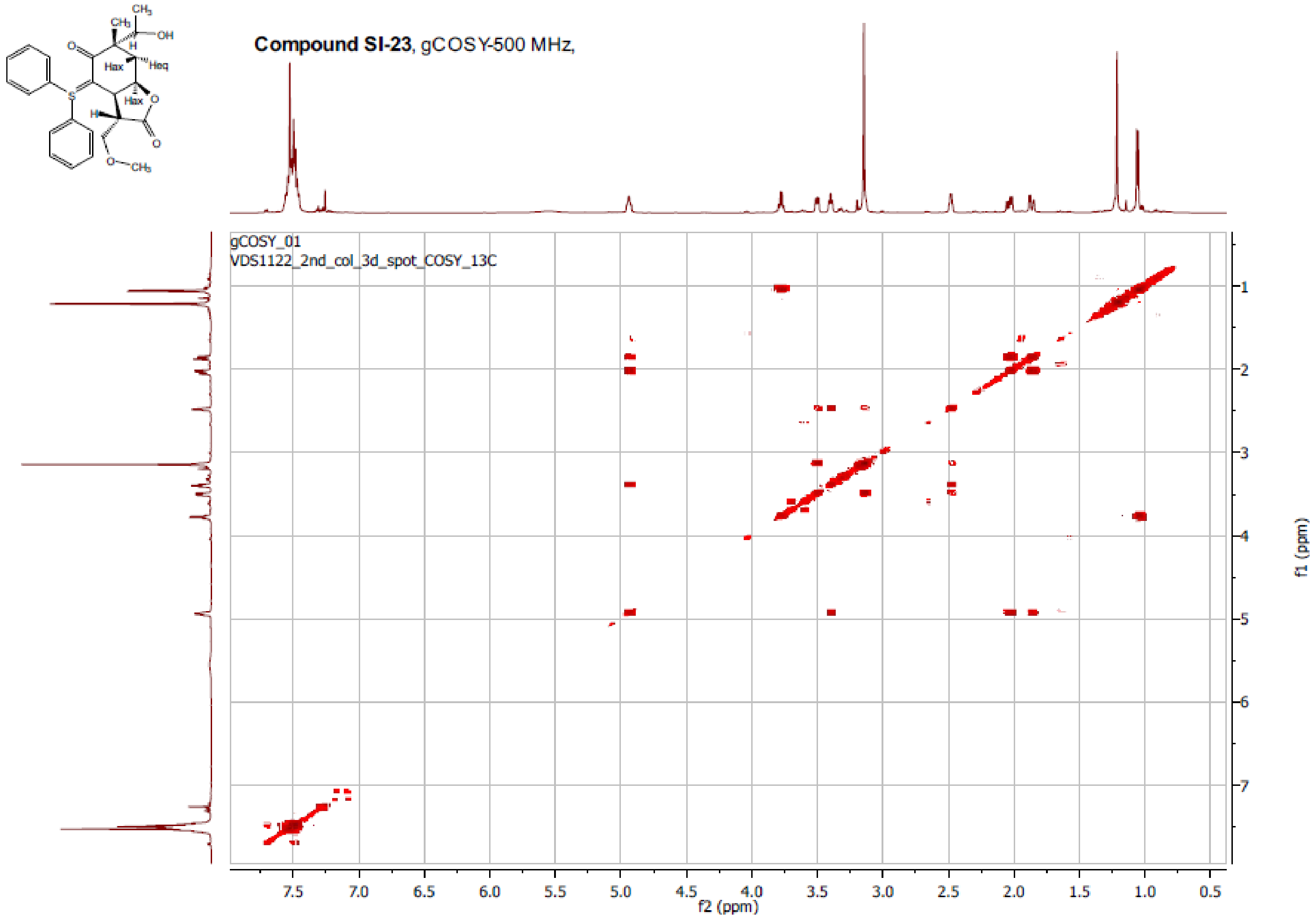
Compound SI-23, gCOSY-500 MHz,
gCOSY_01
VDS1122_2nd_col_3d_spot_COSY_13C
f2 (ppm)
f1 (ppm)

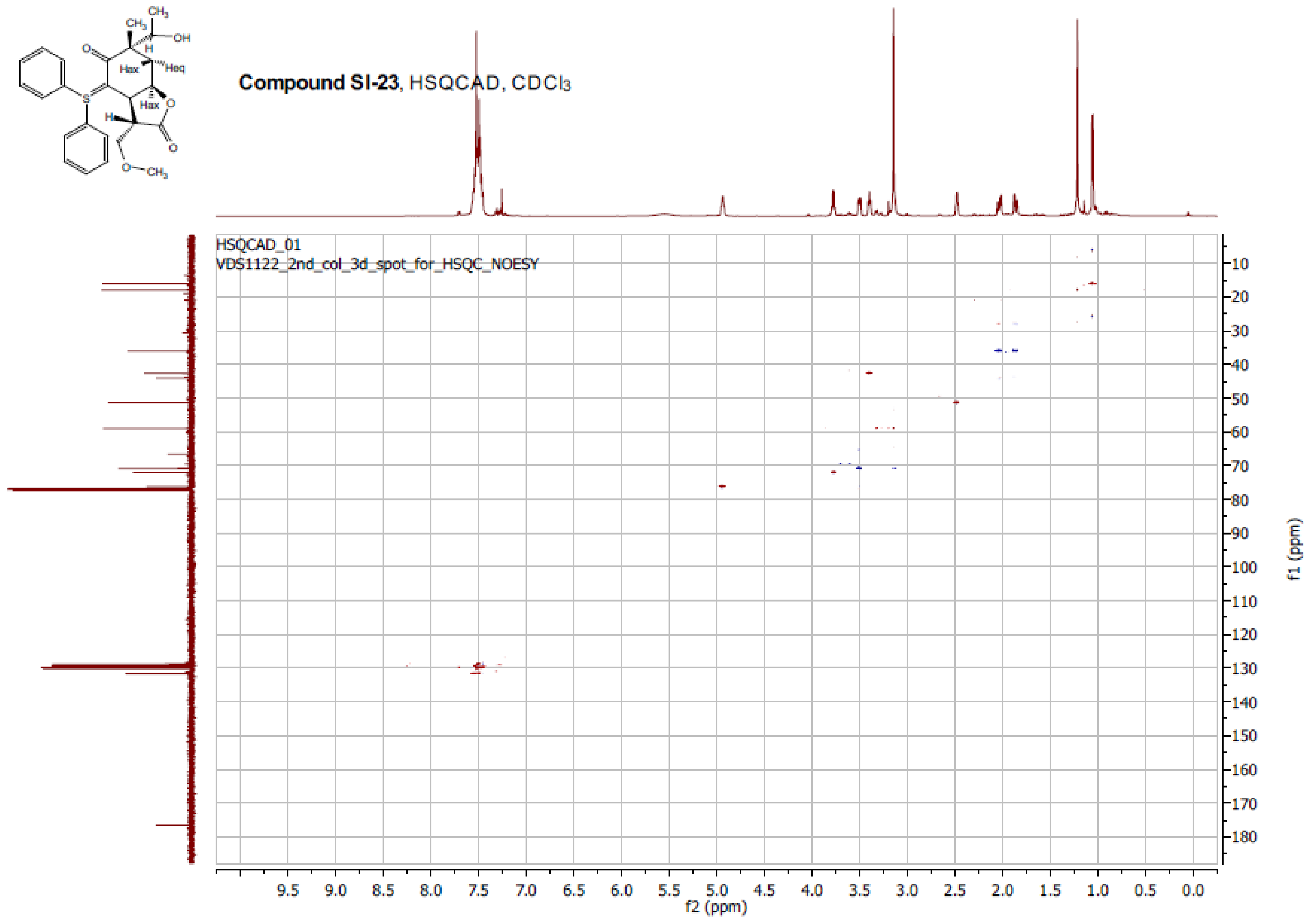
Compound SI-23, HSQCAD, CDCl3
HSQCAD_01
VDS1122_2nd_col_3d_spot_for_HSQC_NOESY
f2 (ppm)
f1 (ppm)

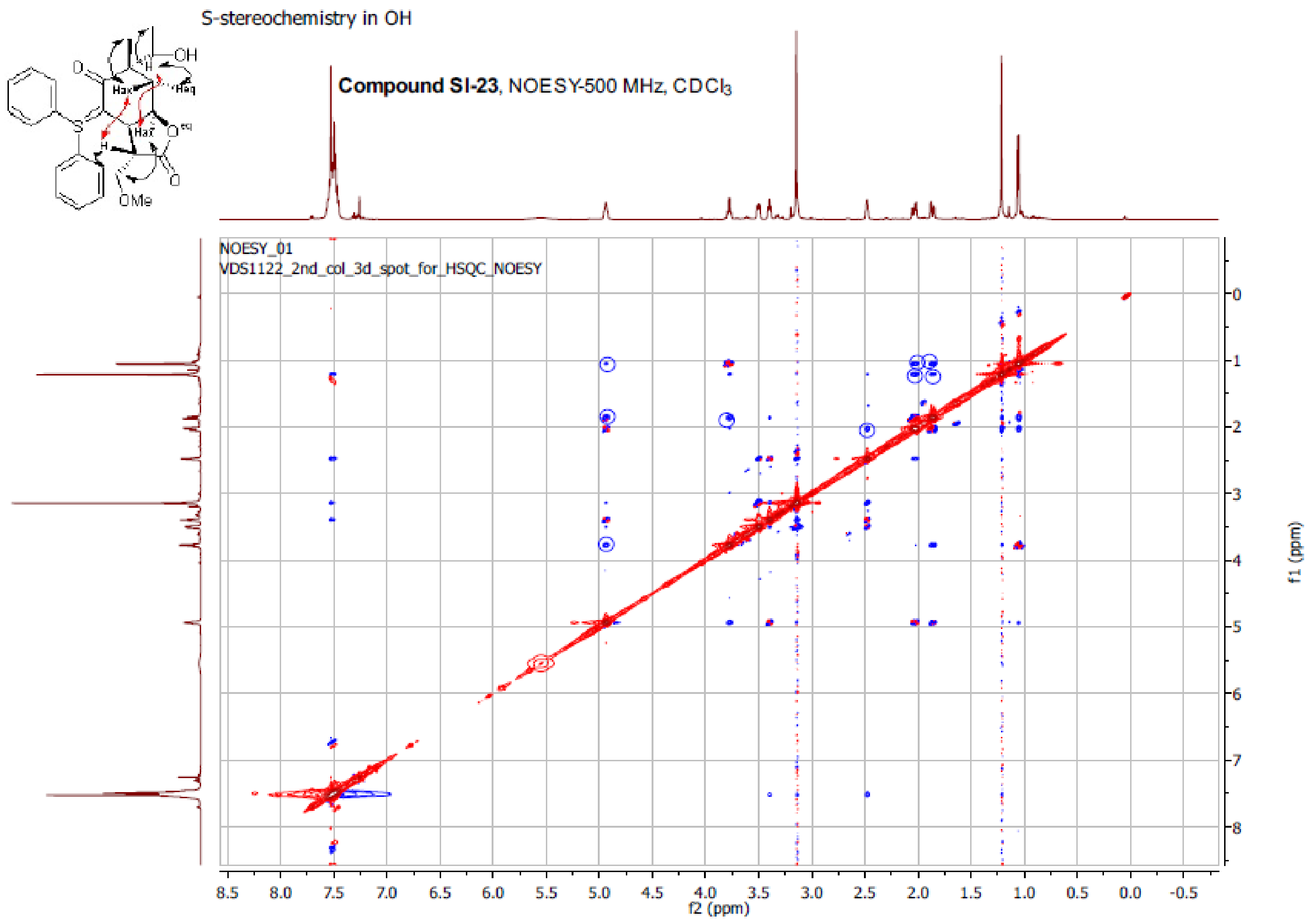
S-stereochemistry in OH
Compound SI-23, NOESY-500 MHz, $CDCl_3$
NOESY_01
VDS1122_2nd_col_3d_spot_for_HSQC_NOESY
f2 (ppm)
f1 (ppm)

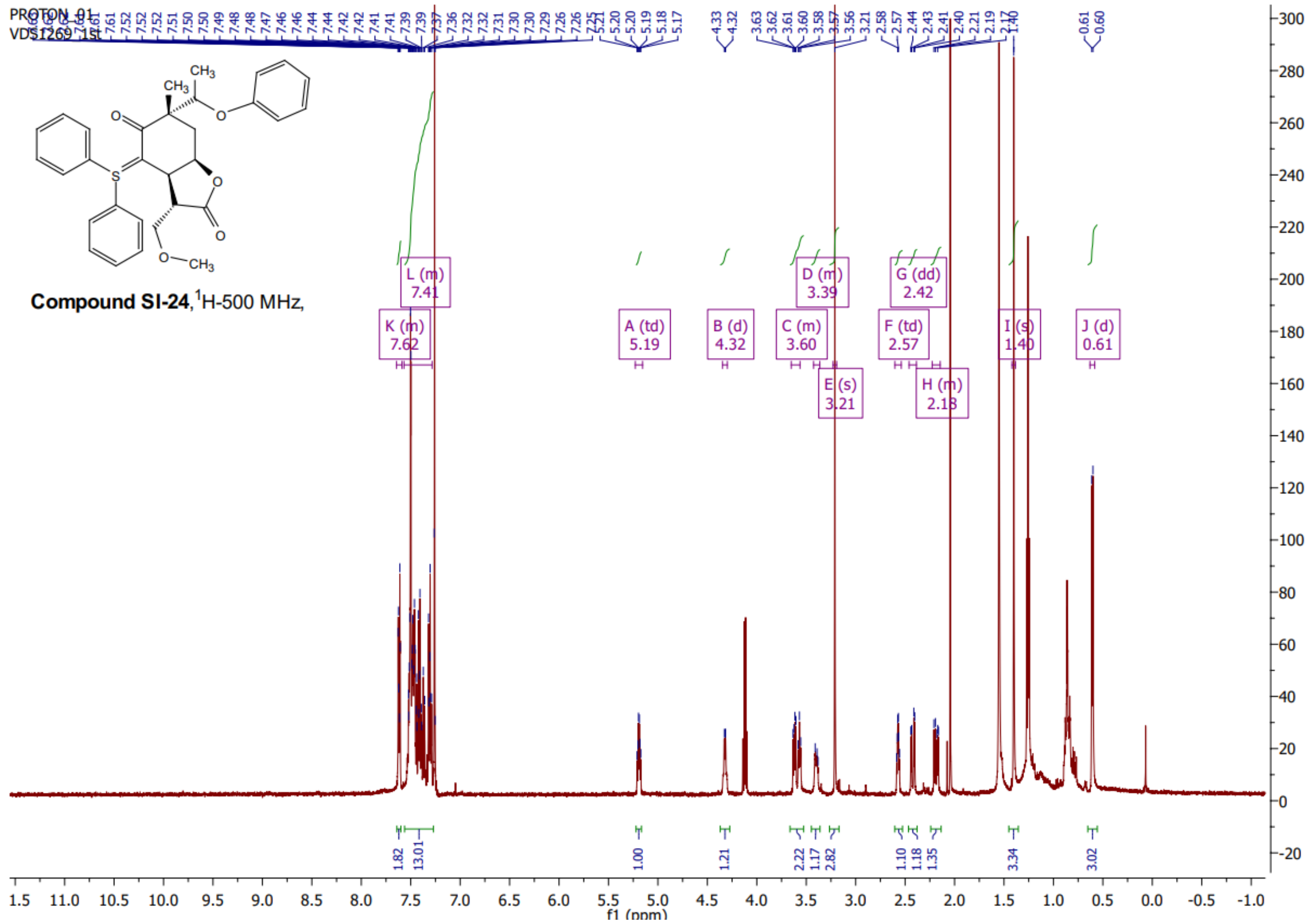
PROTON_01
Compound SI-24, ^{1}H-500 MHz,
K (m) 7.62
L (m) 7.41
A (td) 5.19
B (d) 4.32
C (m) 3.60
D (m) 3.39
E (s) 3.21
F (td) 2.57
G (dd) 2.42
H (m) 2.13
I (s) 1.40
J (d) 0.61
f1 (ppm)

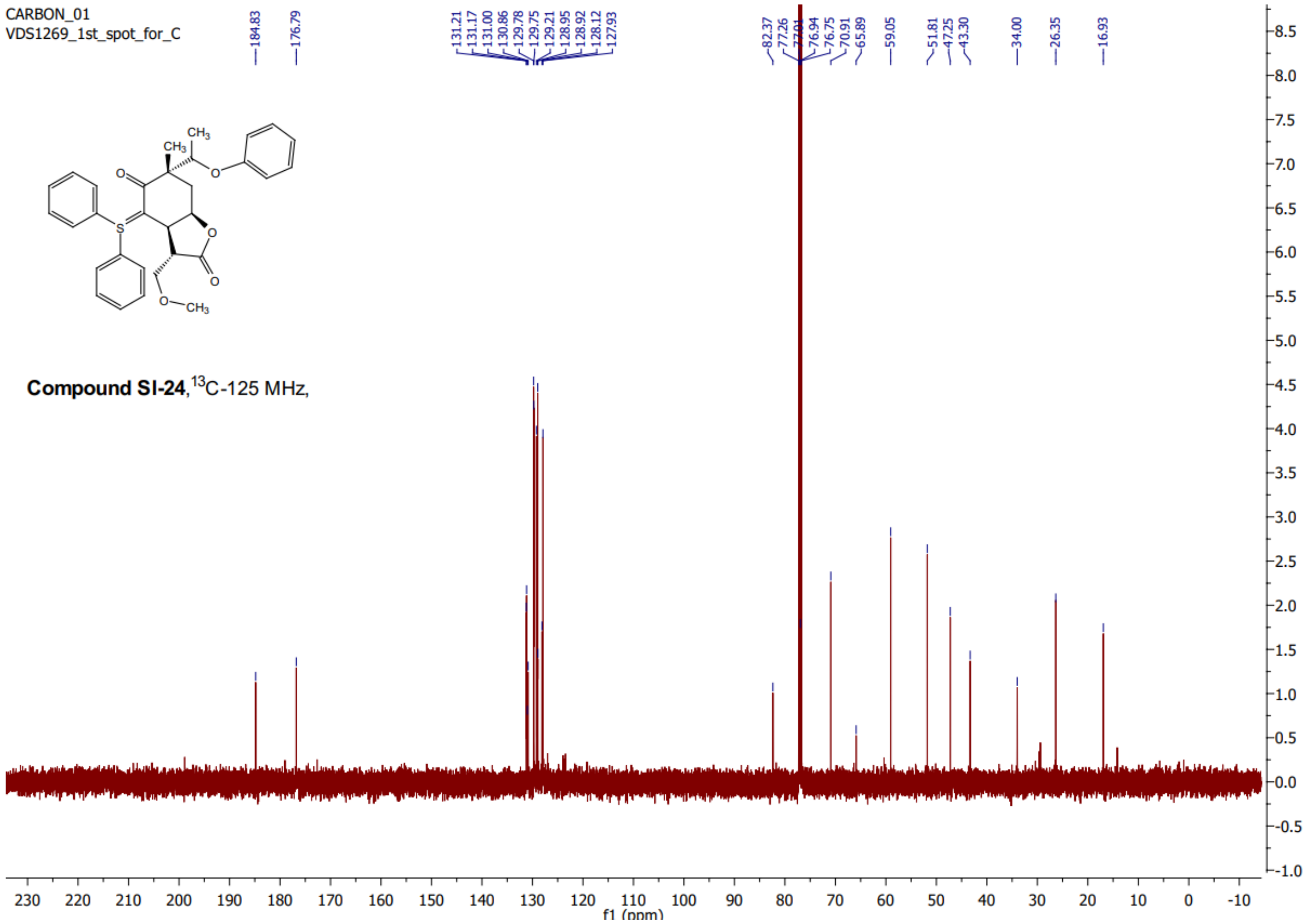
CARBON_01
VDS1269_1st_spot_for_C
Compound SI-24, 13C-125 MHz,
f1 (ppm)

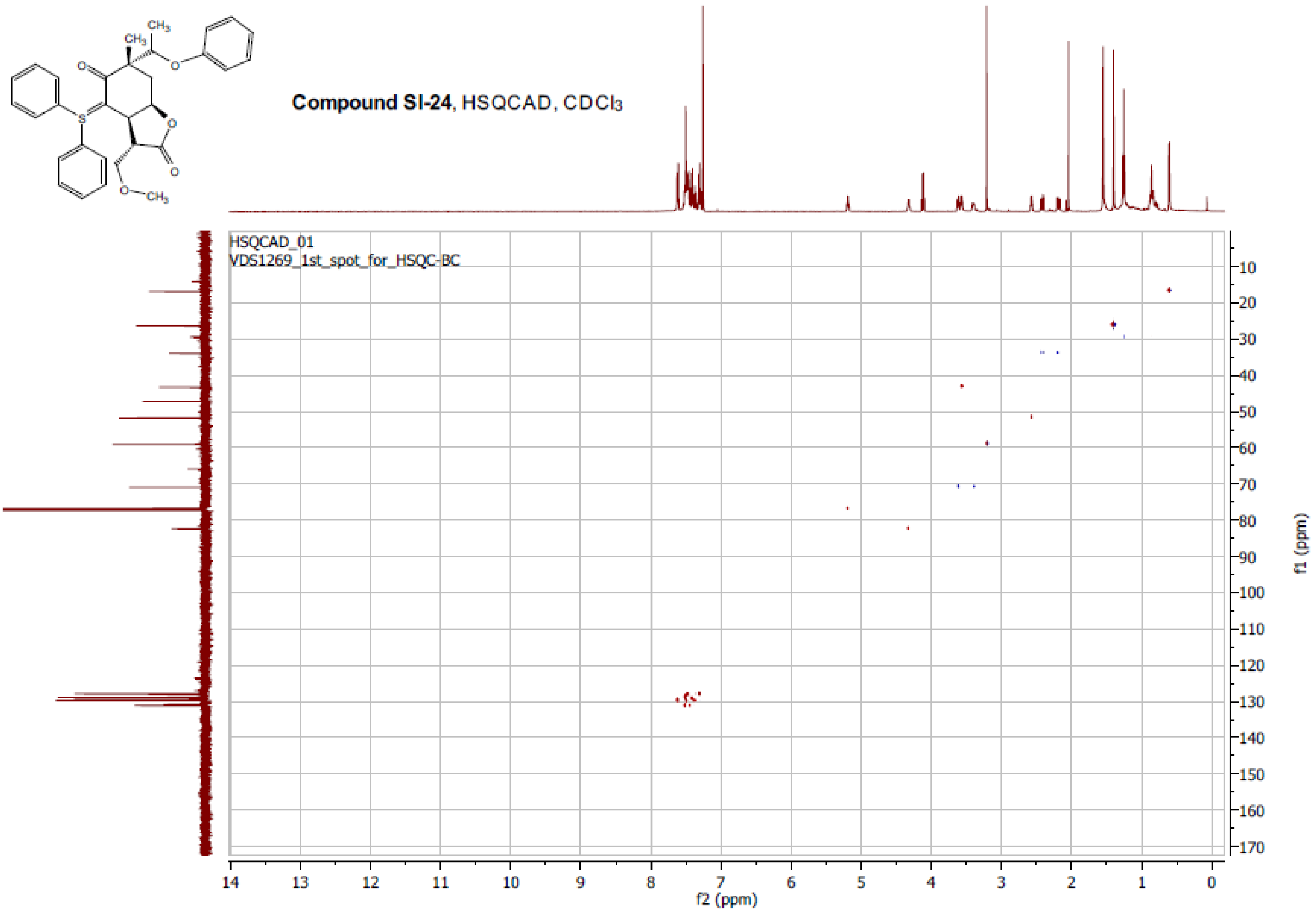
Compound SI-24, HSQCAD, CDCl3
HSQCAD_01
VDS1269_1st_spot_for_HSQC-BC
f2 (ppm)
f1 (ppm)

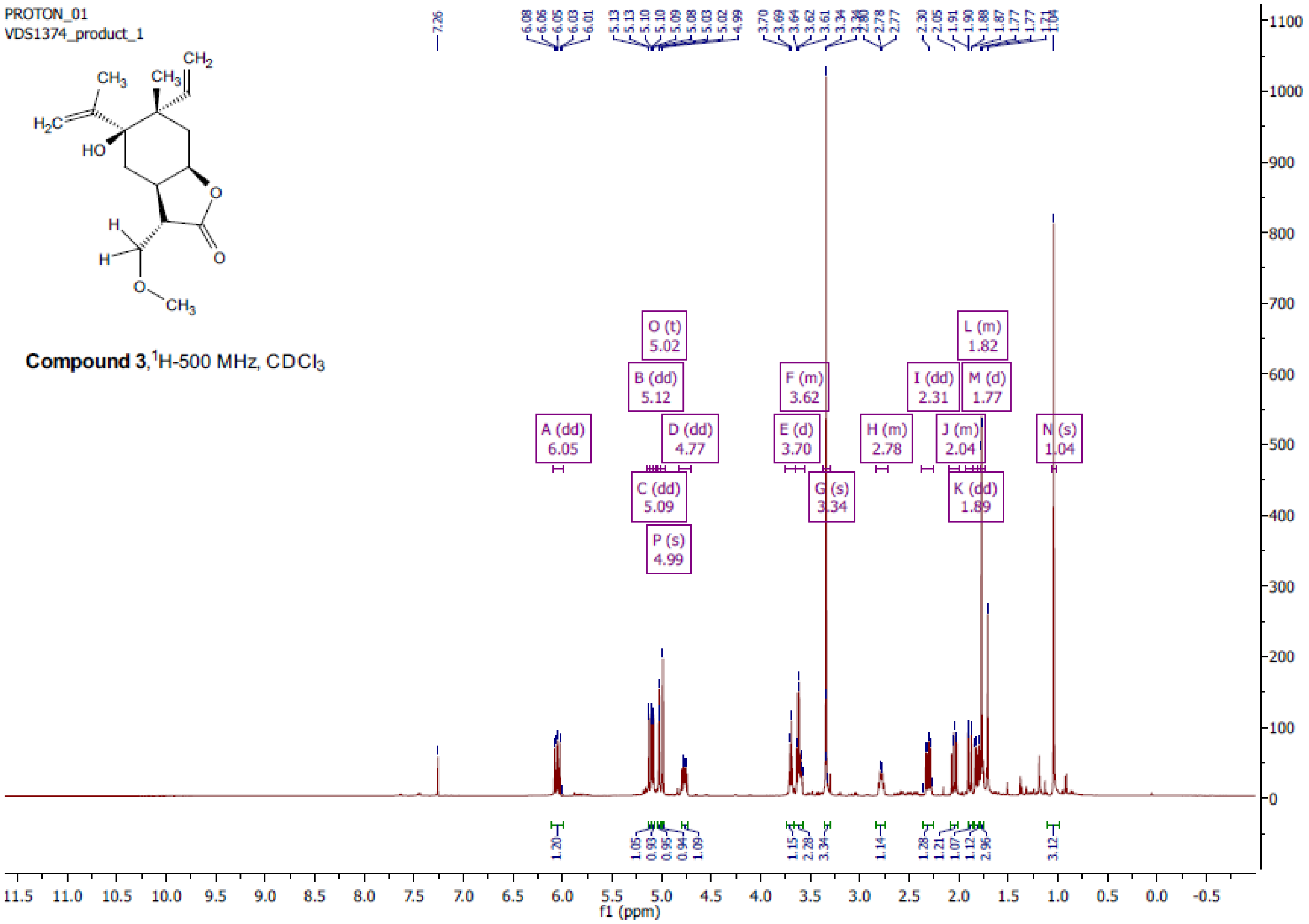
PROTON_01
VDS1374_product_1
Compound 3, 1H-500 MHz, CDCl3
f1 (ppm)

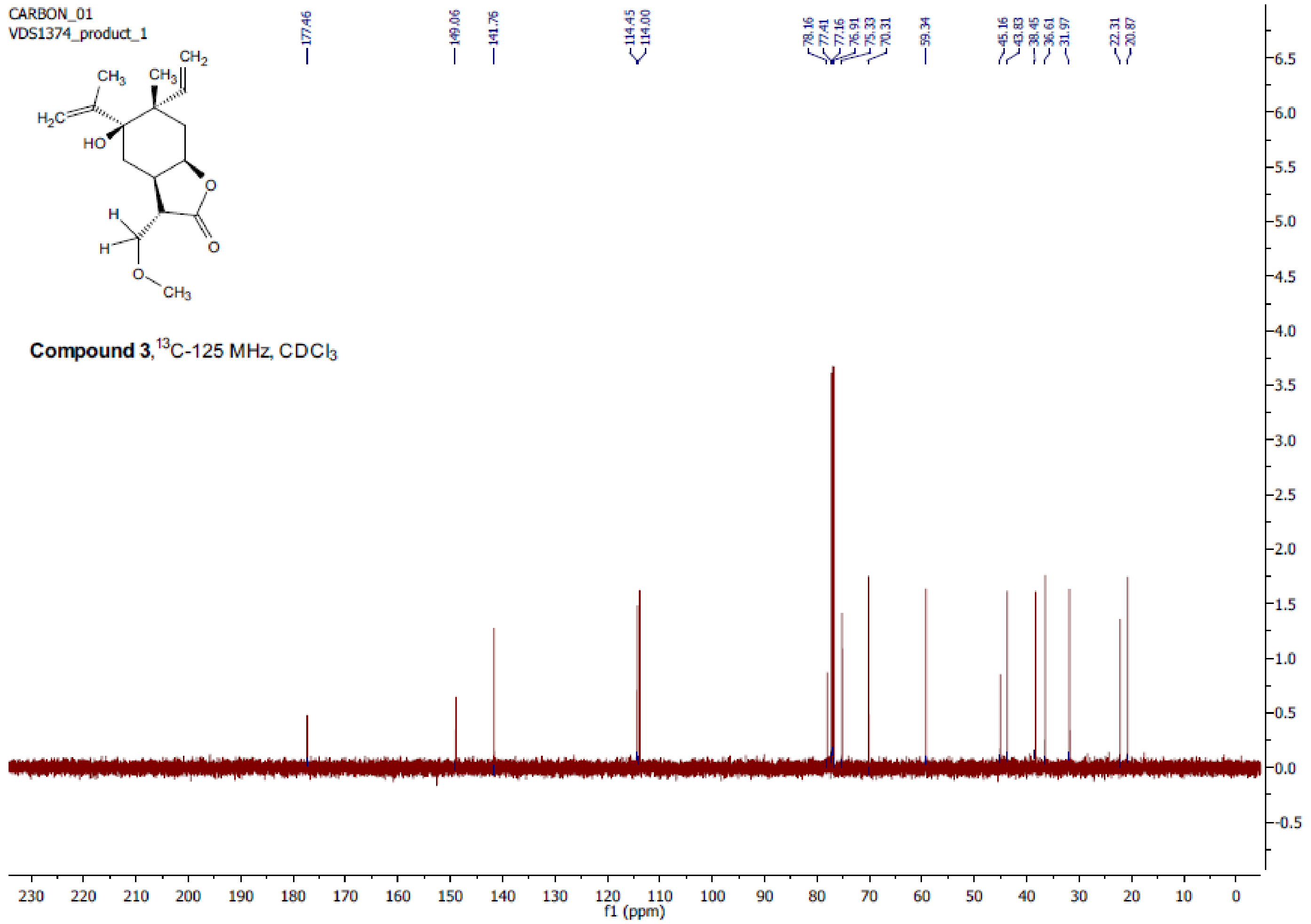
CARBON_01
VDS1374_product_1
Compound 3, 13C-125 MHz, CDCl3
f1 (ppm)

Compound 3, gCOSY-500 MHz, CDCl₃

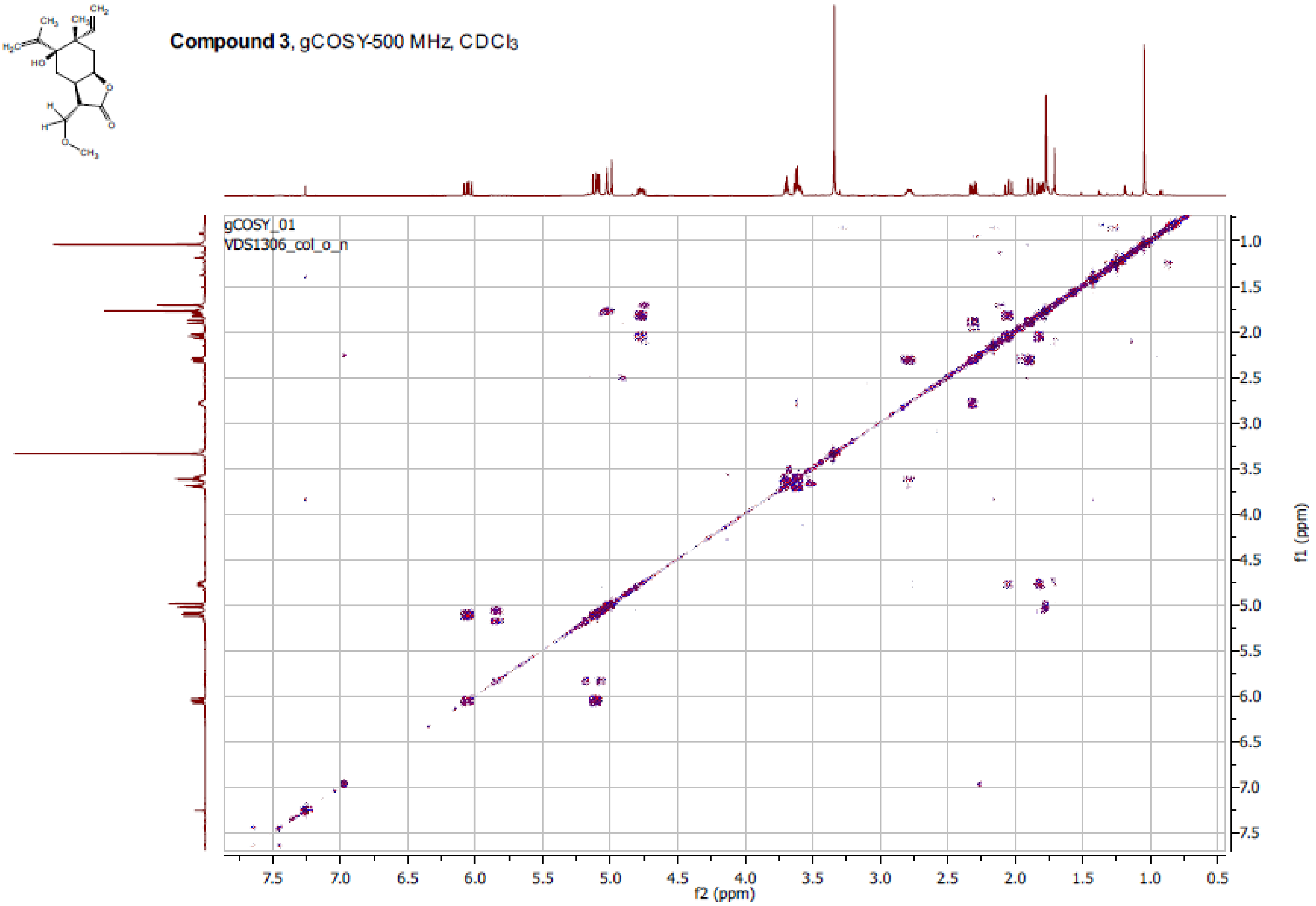

Compound 3, HSQCAD, $CDCl_3$

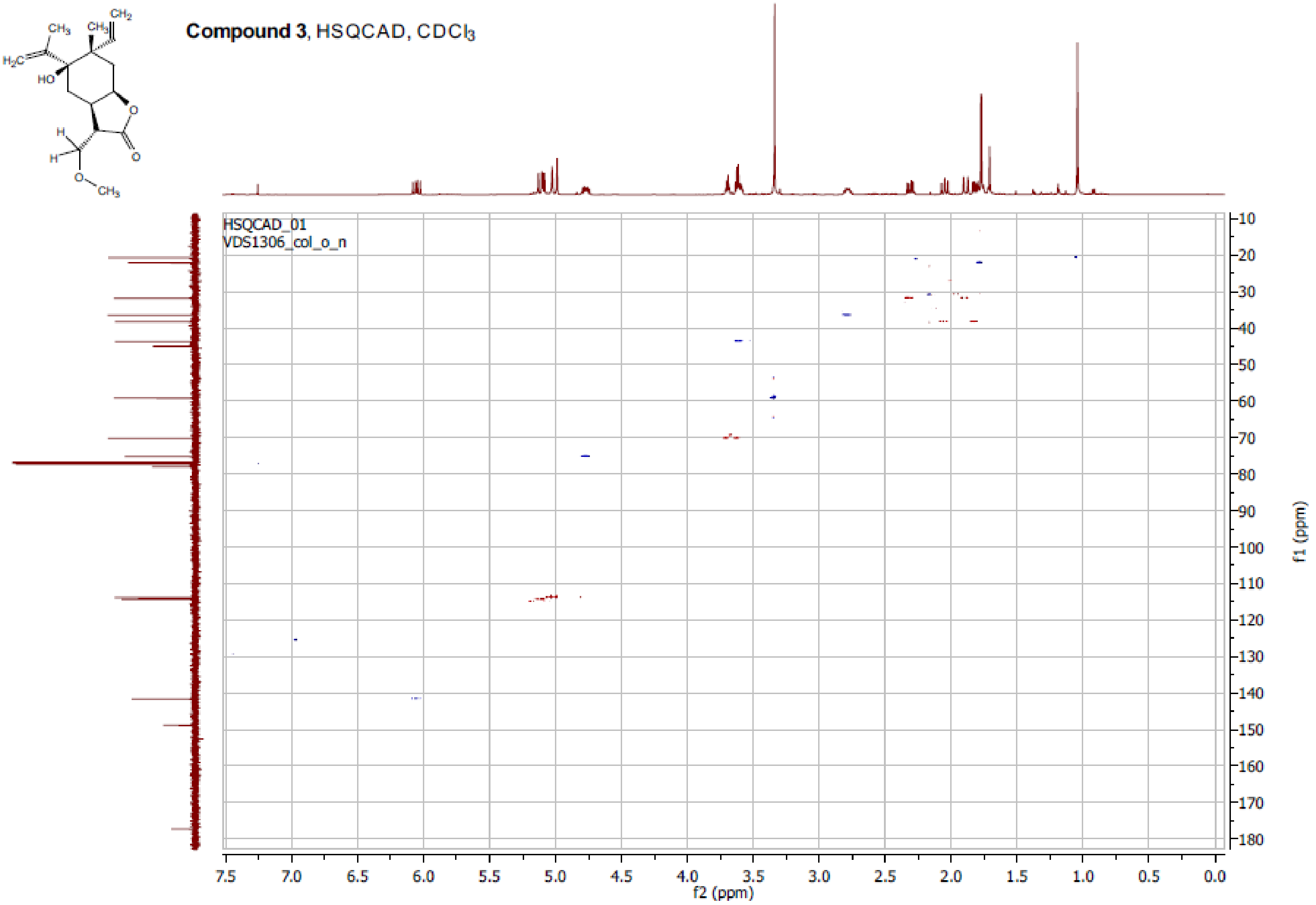

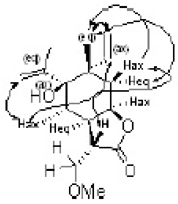

Compound 3, NOESY-500 MHz, $CDCl_3$

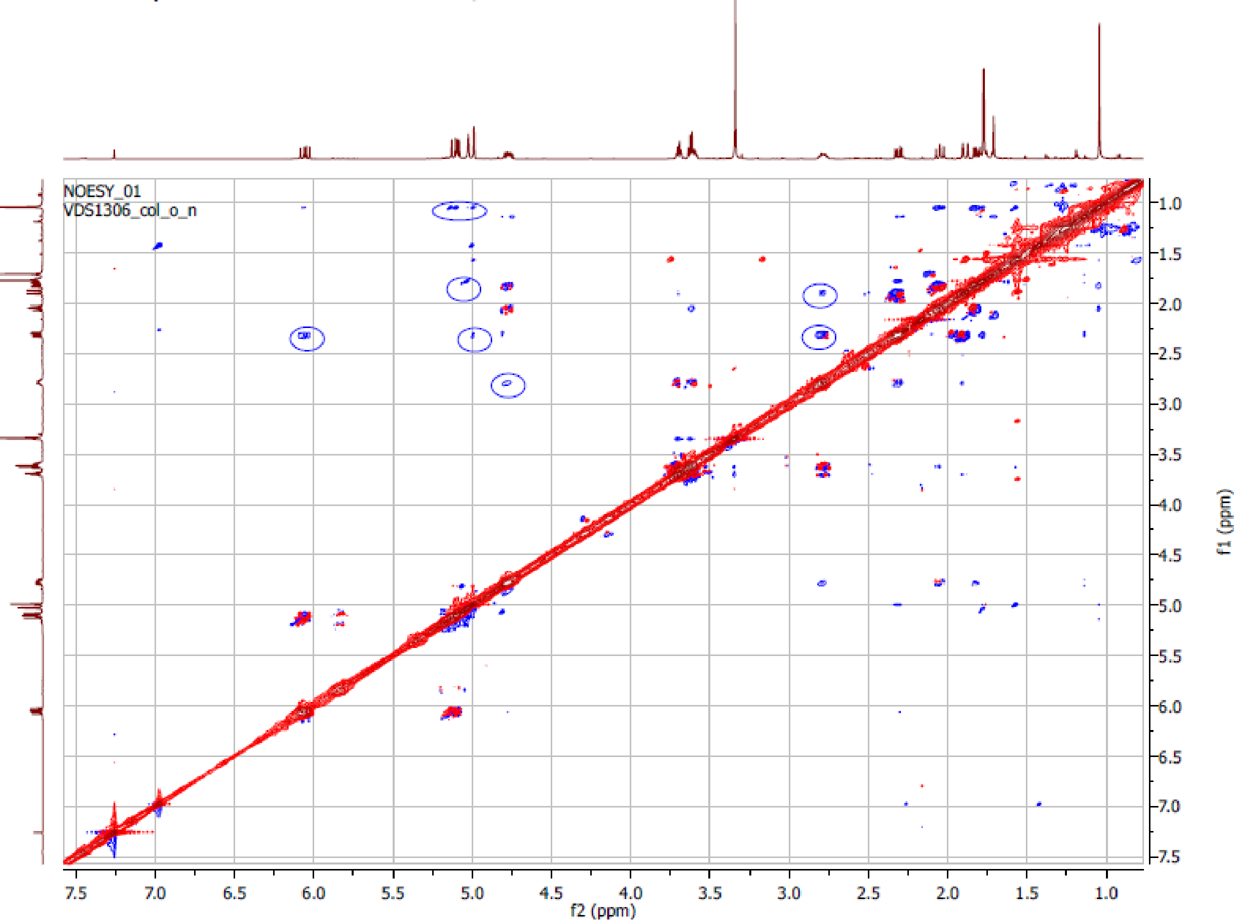

Compound SI-19, ^{1}H-500 MHz, $CDCl_3$

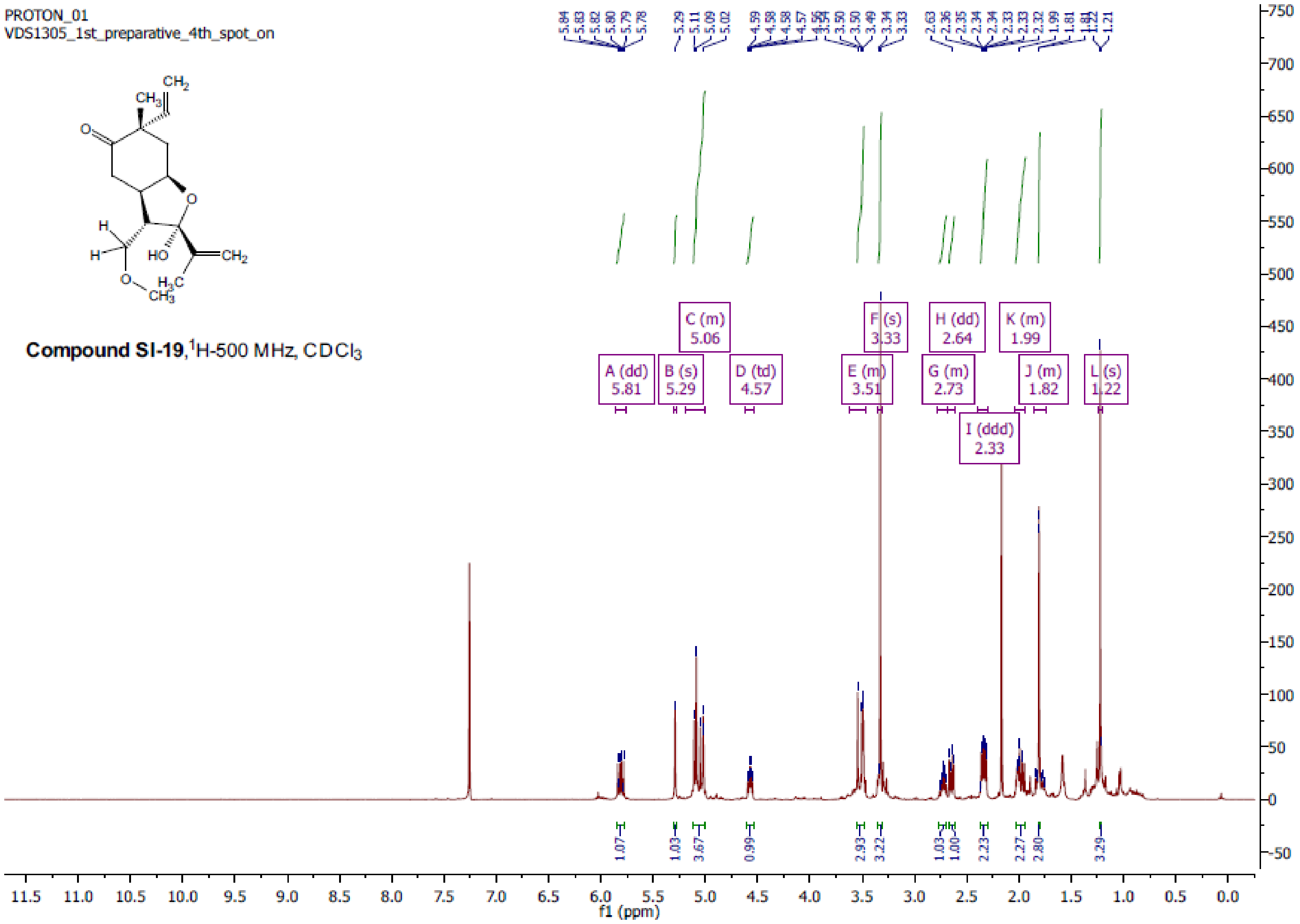

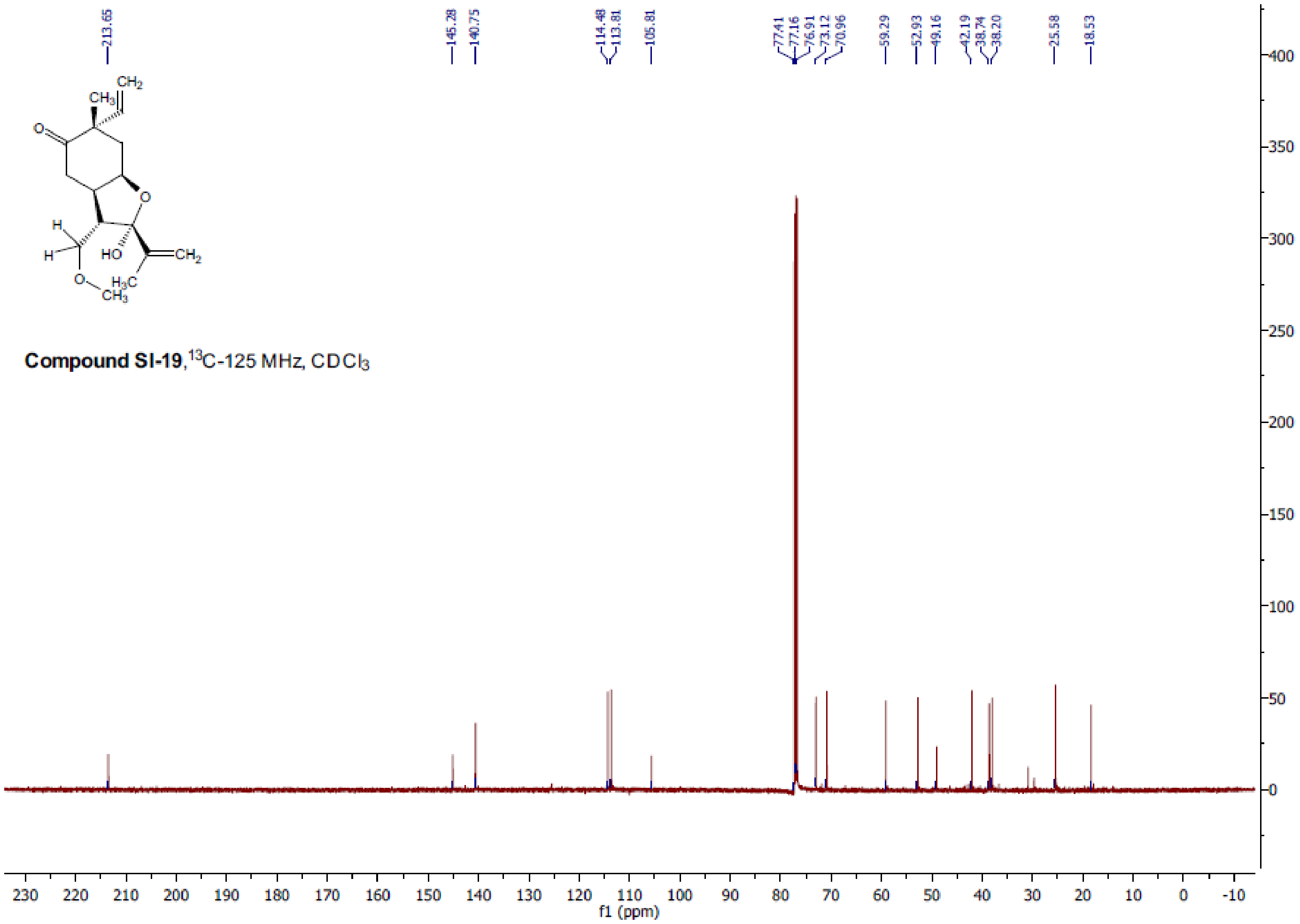
213.65
145.28
140.75
114.48
113.81
105.81
77.41
77.16
76.91
73.12
70.96
59.29
52.93
49.16
42.19
38.74
38.20
25.58
18.53
CH2
CH3
O
H
H
HO
CH2
O
H3C
CH3
Compound SI-19, 13C-125 MHz, CDCl3
f1 (ppm)

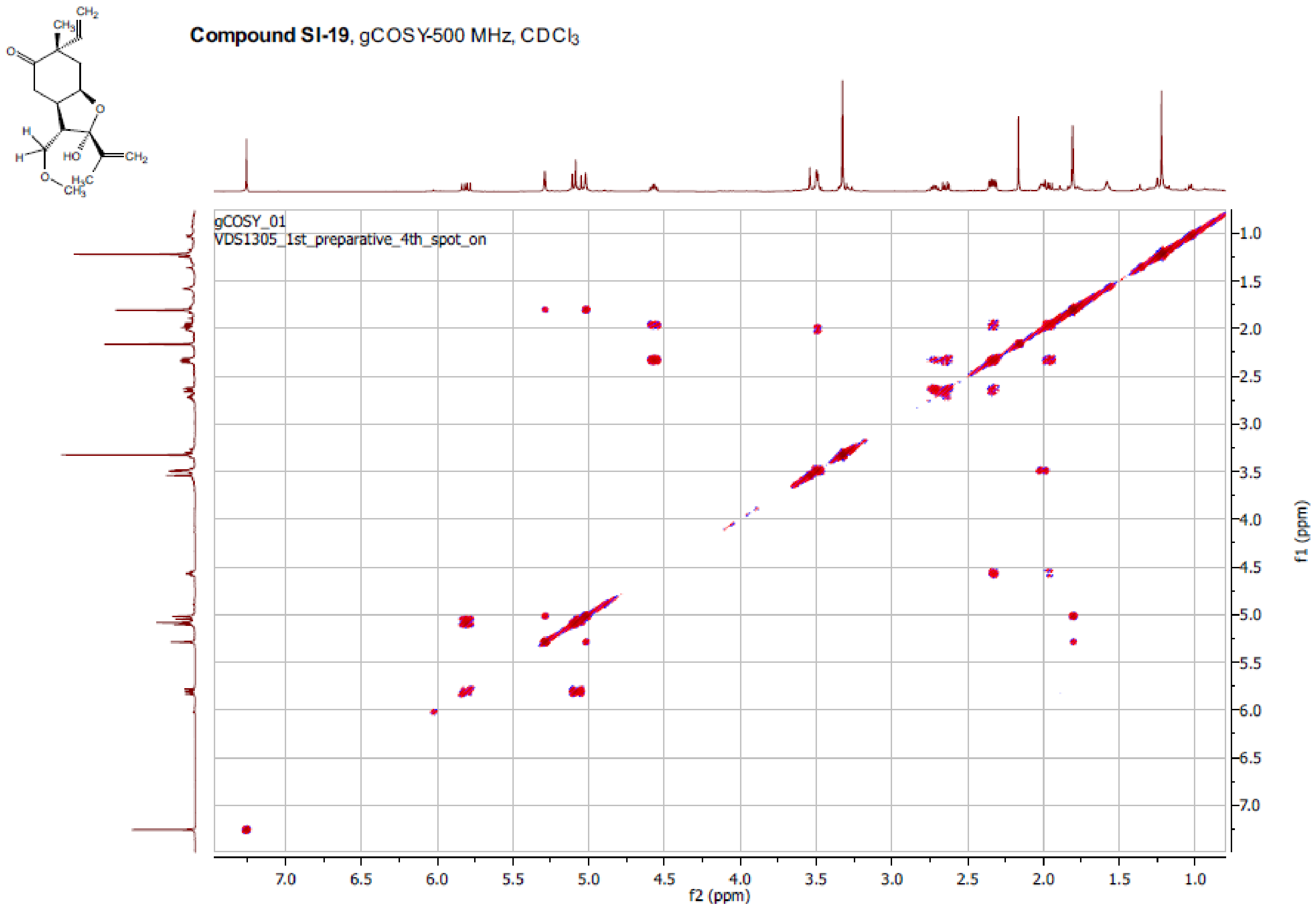
Compound SI-19, gCOSY-500 MHz, CDCl3
gCOSY_01
VDS1305_1st_preparative_4th_spot_on
f2 (ppm)
f1 (ppm)

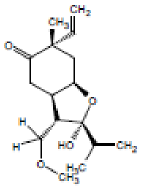

Compound SI-19, HSQCAD, $CDCl_3$

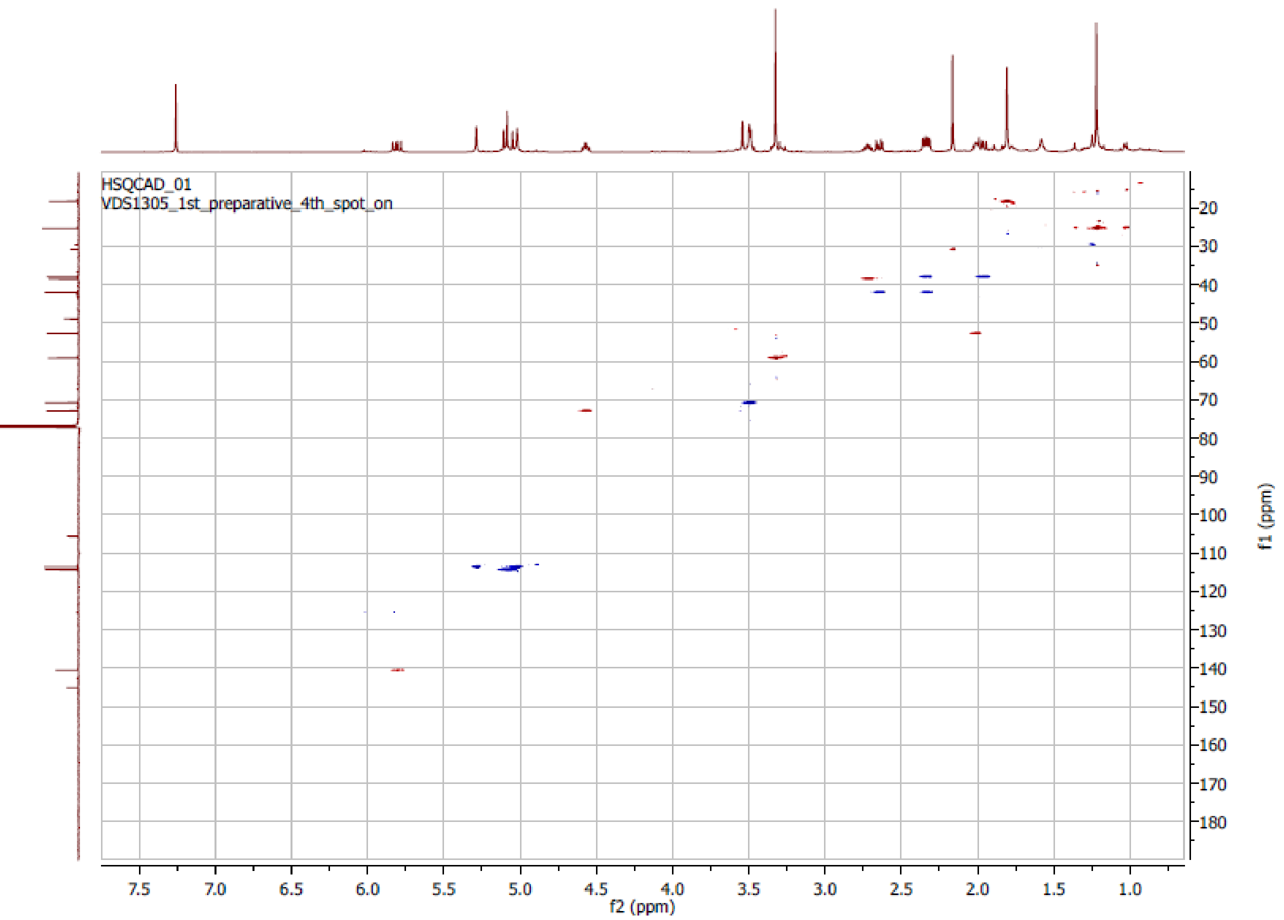

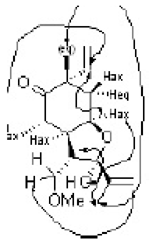

Compound SI-19, NOESY-500 MHz, $CDCl_3$

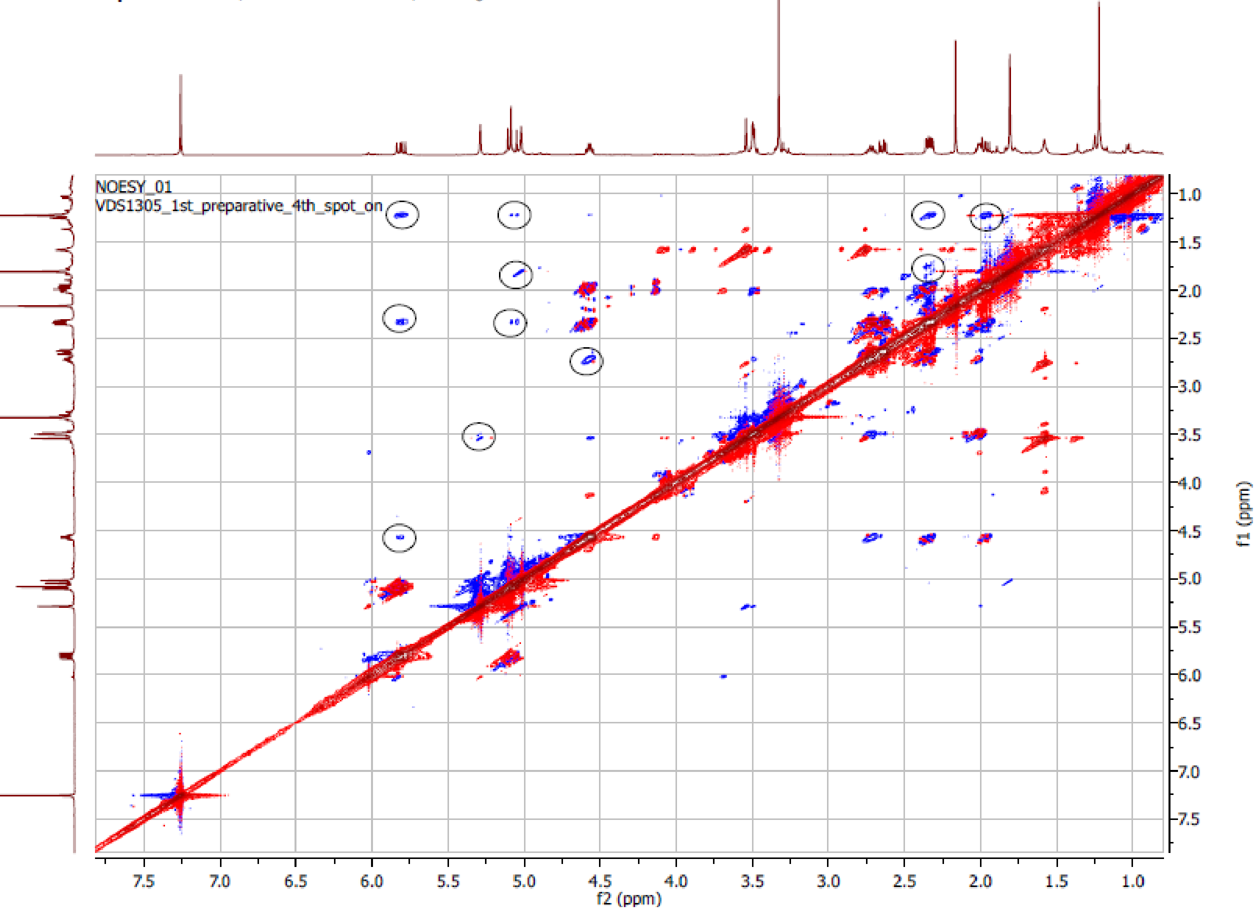

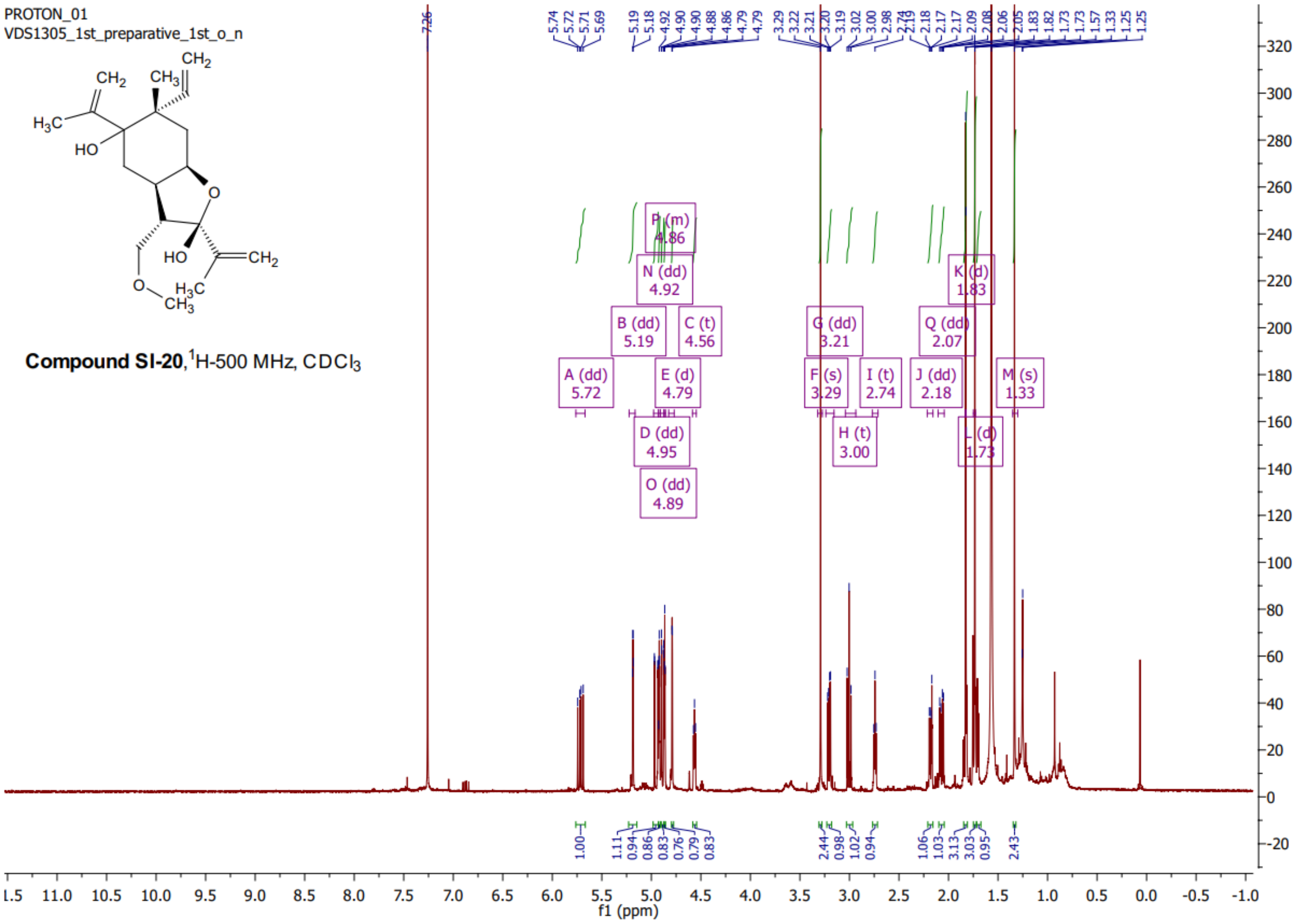
PROTON_01
VDS1305_1st_preparative_1st_o_n
Compound SI-20, 1H-500 MHz, CDCl3
f1 (ppm)

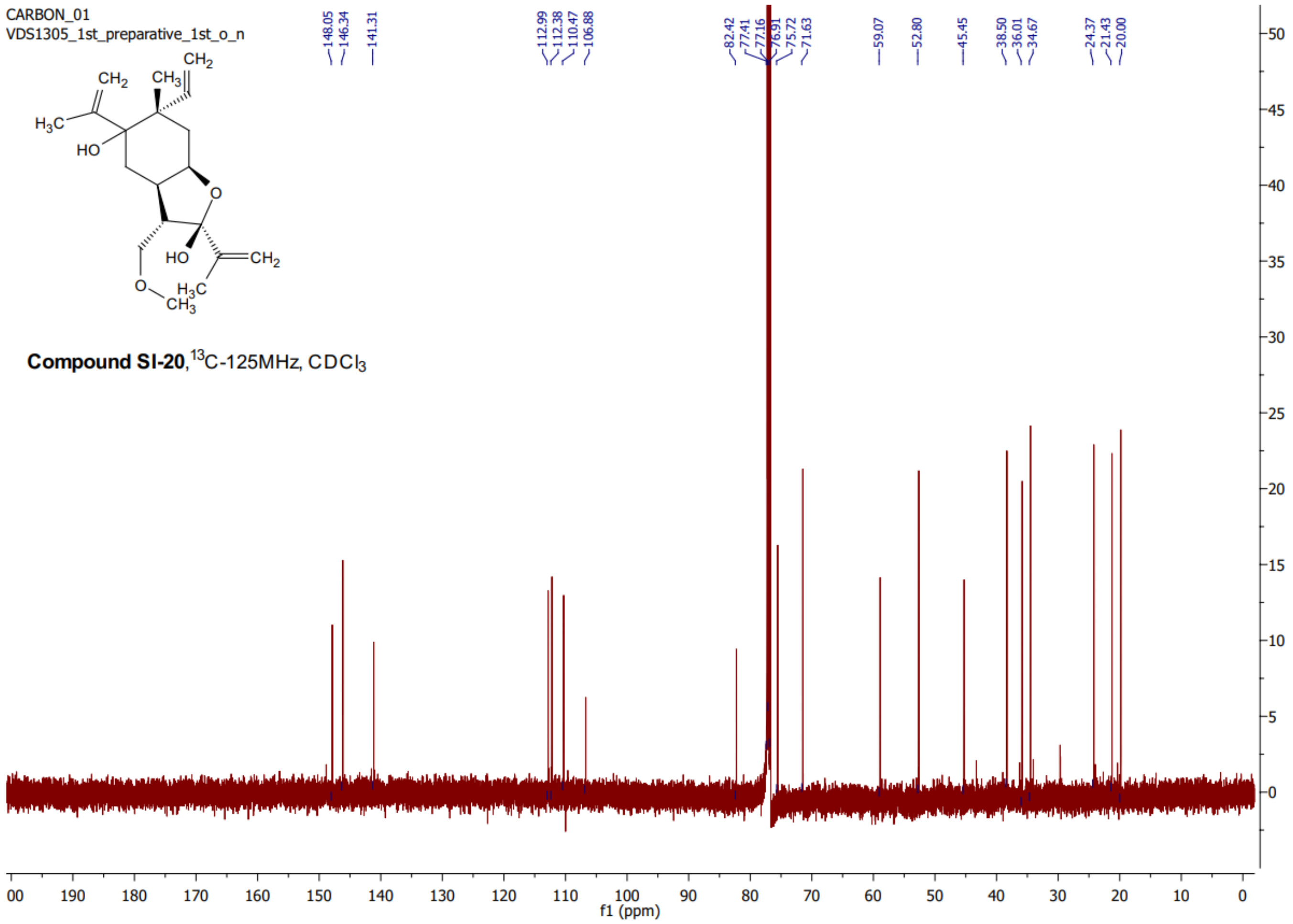
CARBON_01
VDS1305_1st_preparative_1st_o_n
Compound SI-20, 13C-125MHz, CDCl3
148.05
146.34
141.31
112.99
112.38
110.47
106.88
82.42
77.41
77.16
76.91
75.72
71.63
59.07
52.80
45.45
38.50
36.01
34.67
24.37
21.43
20.00
f1 (ppm)

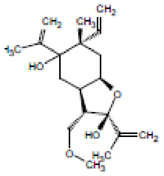

Compound SI-20, gCOSY-500 MHz, $CDCl_3$

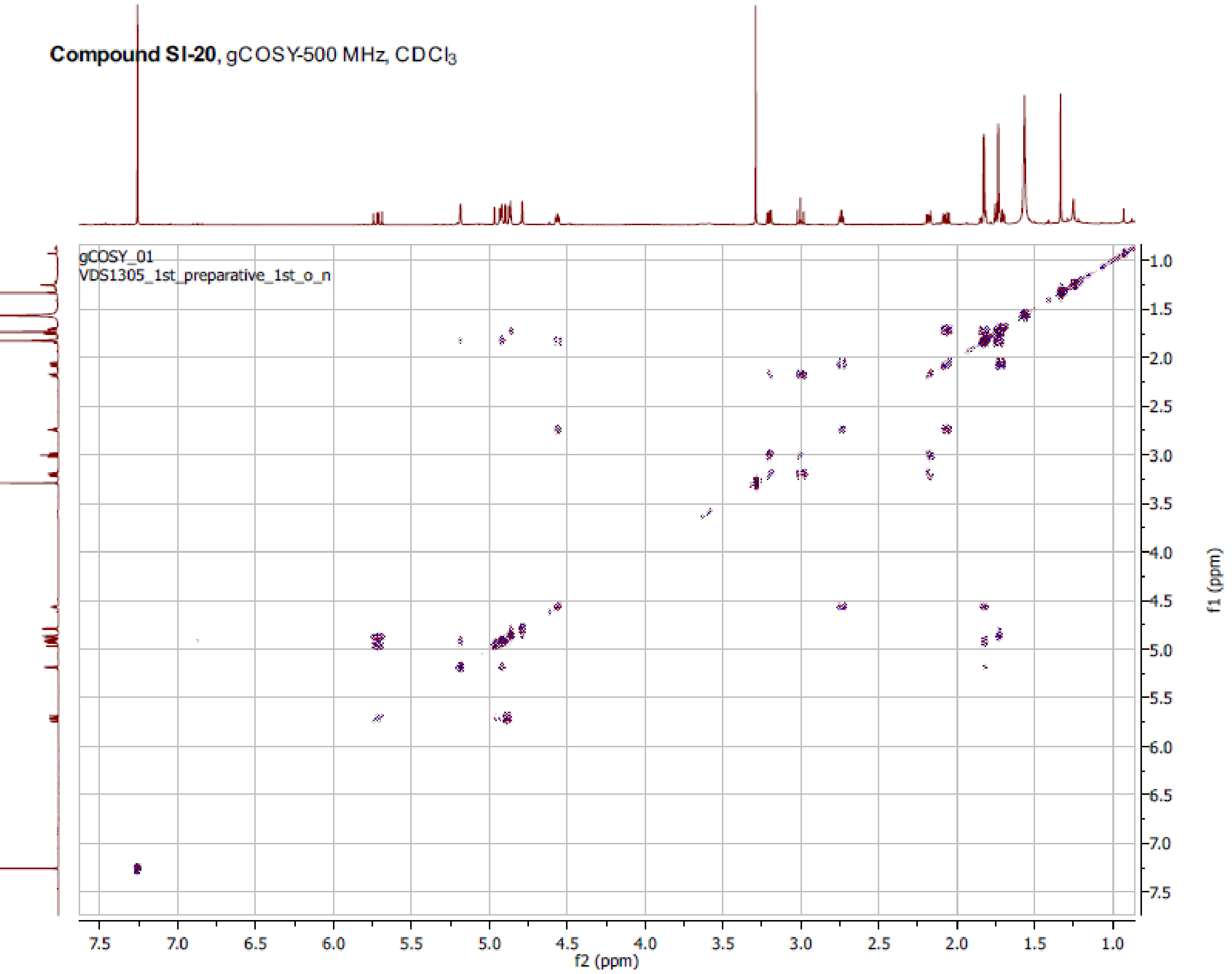

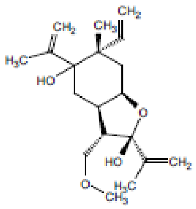

Compound SI-20, HSQCAD, $CDCl_3$

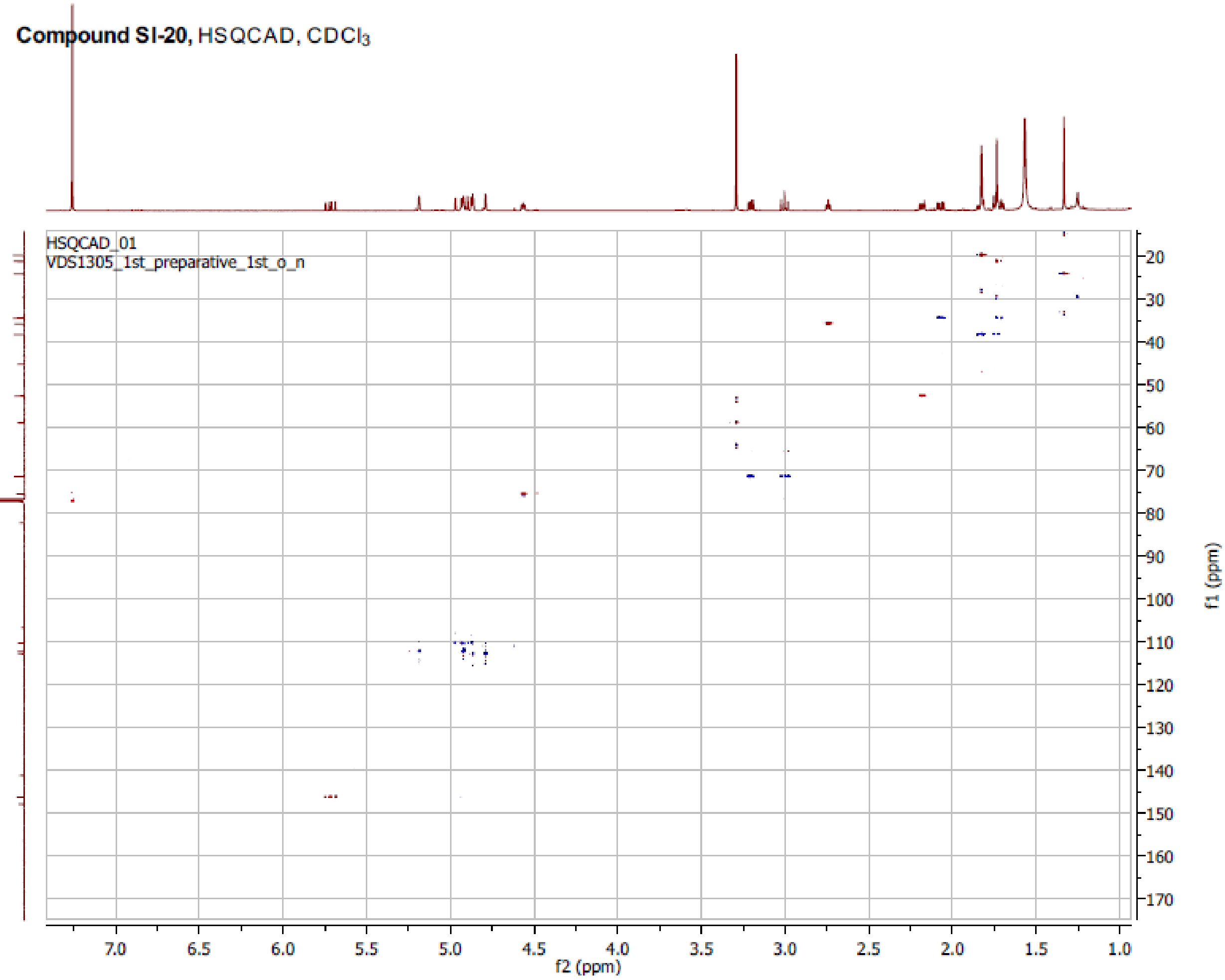

Compound SI-20, NOESY-500 MHz, CDCl3

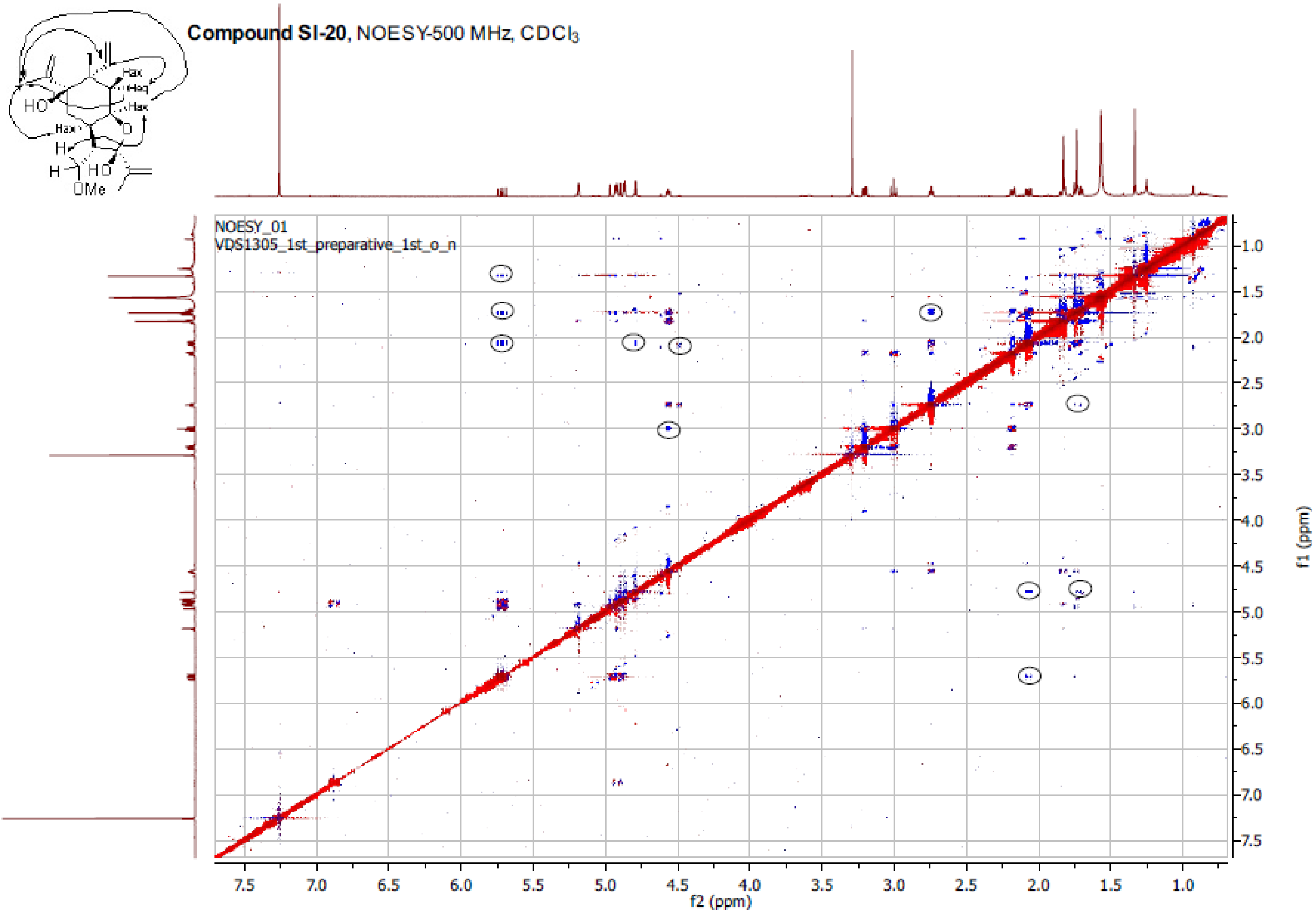

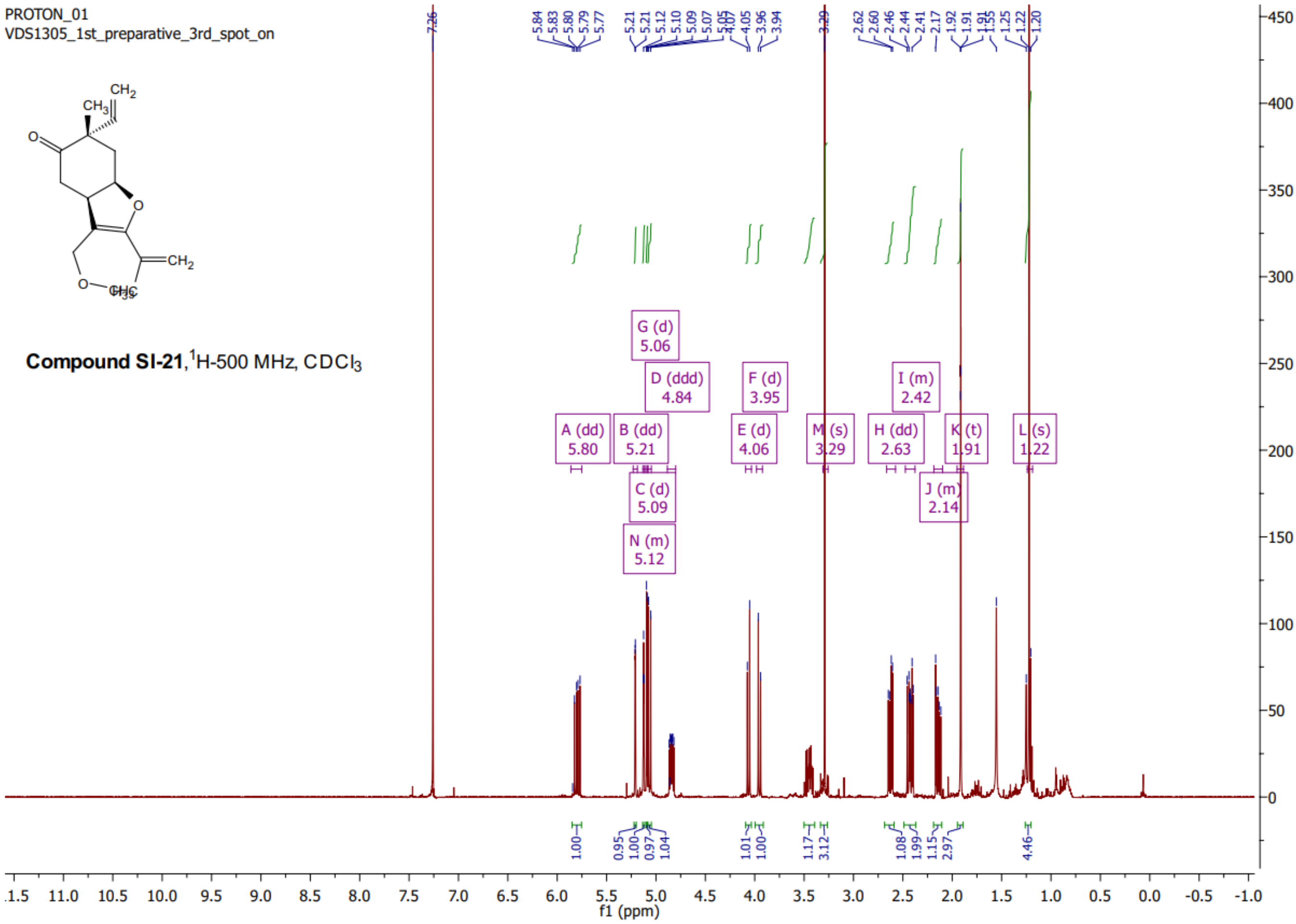

Compound SI-21, ^{1}H-500 MHz, CDCl3

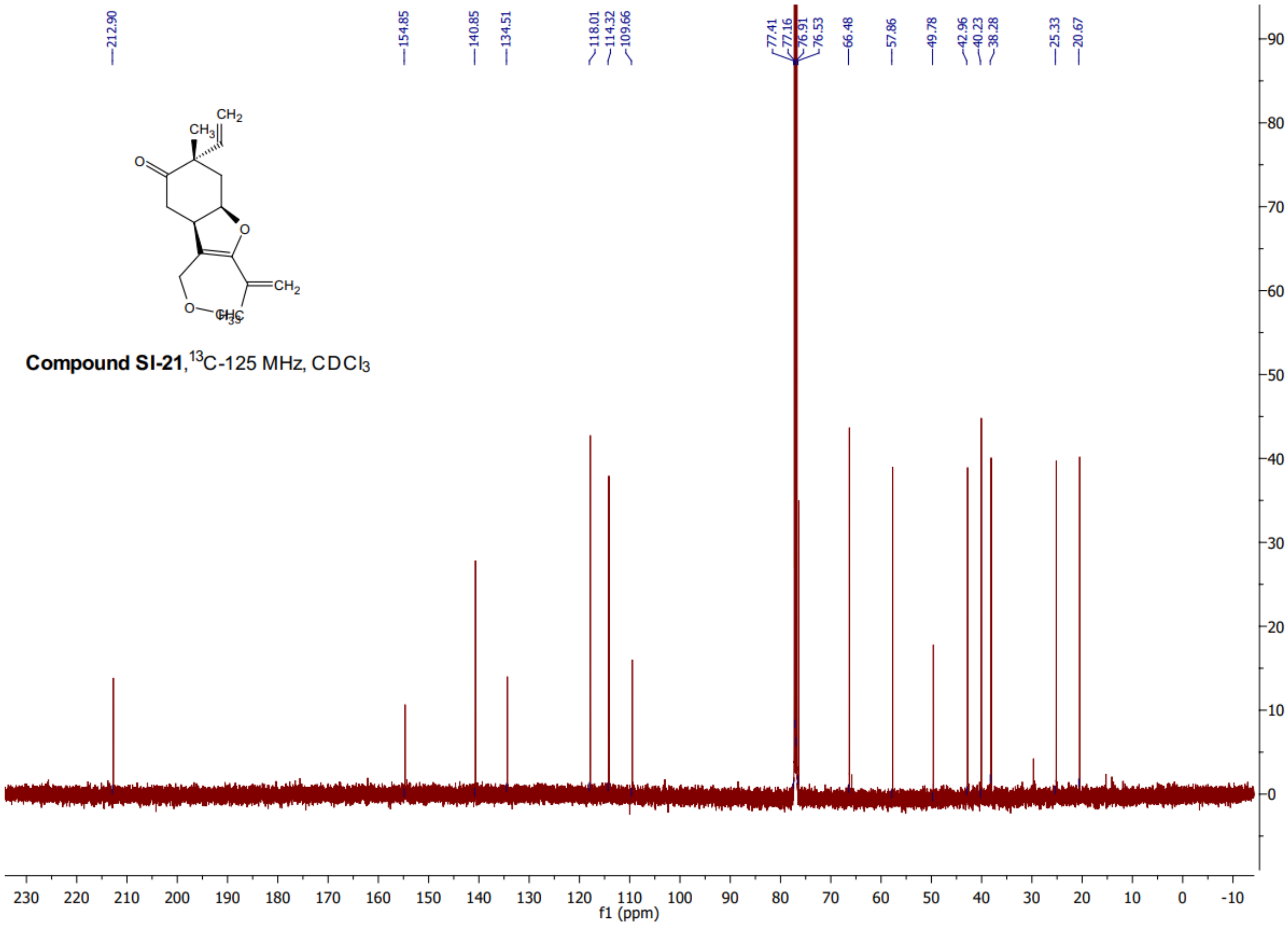

Compound SI-21, ^{13}C-125 MHz, $CDCl_3$

Compound SI-21, gCOSY-500 MHz, $CDCl_3$

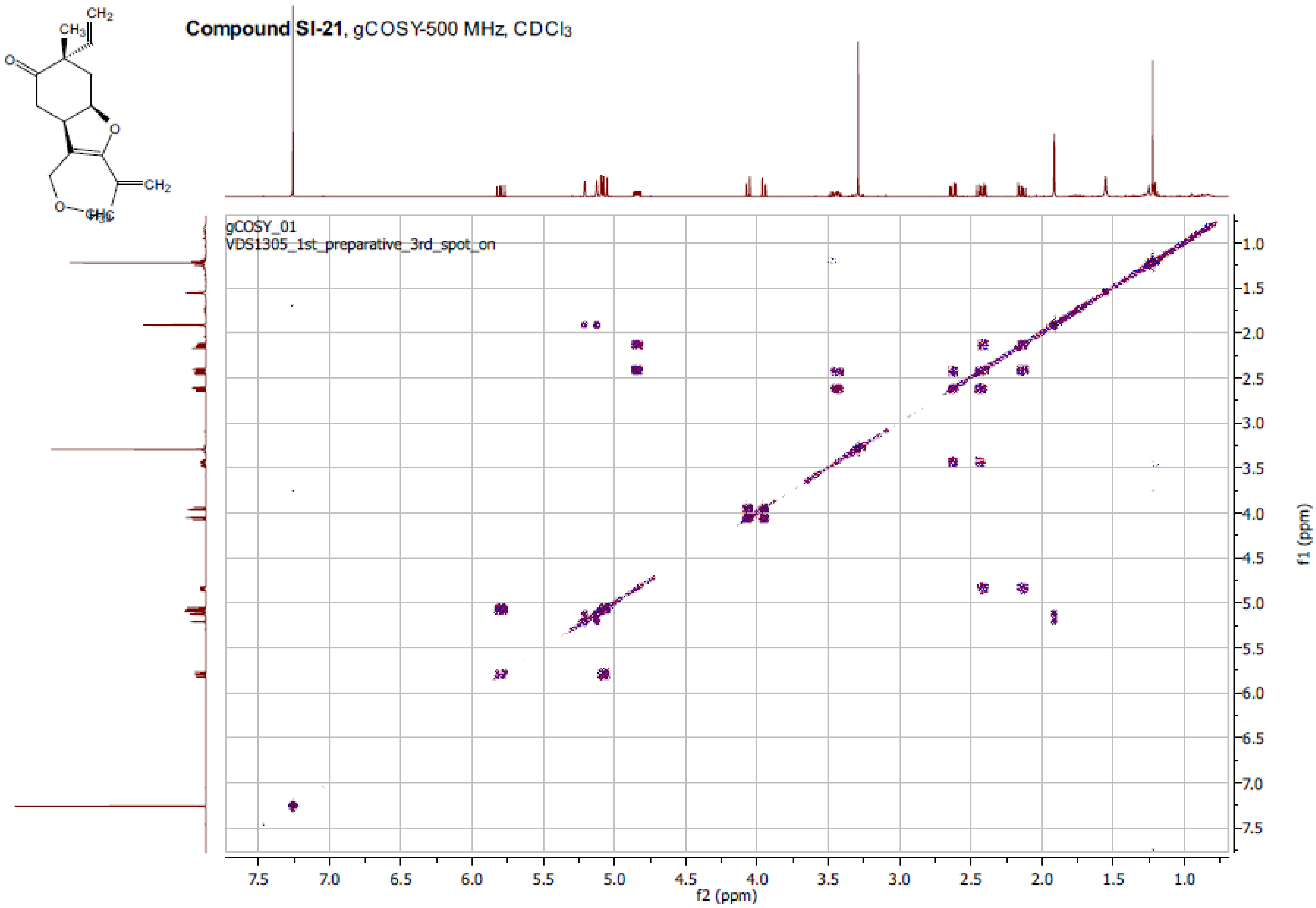

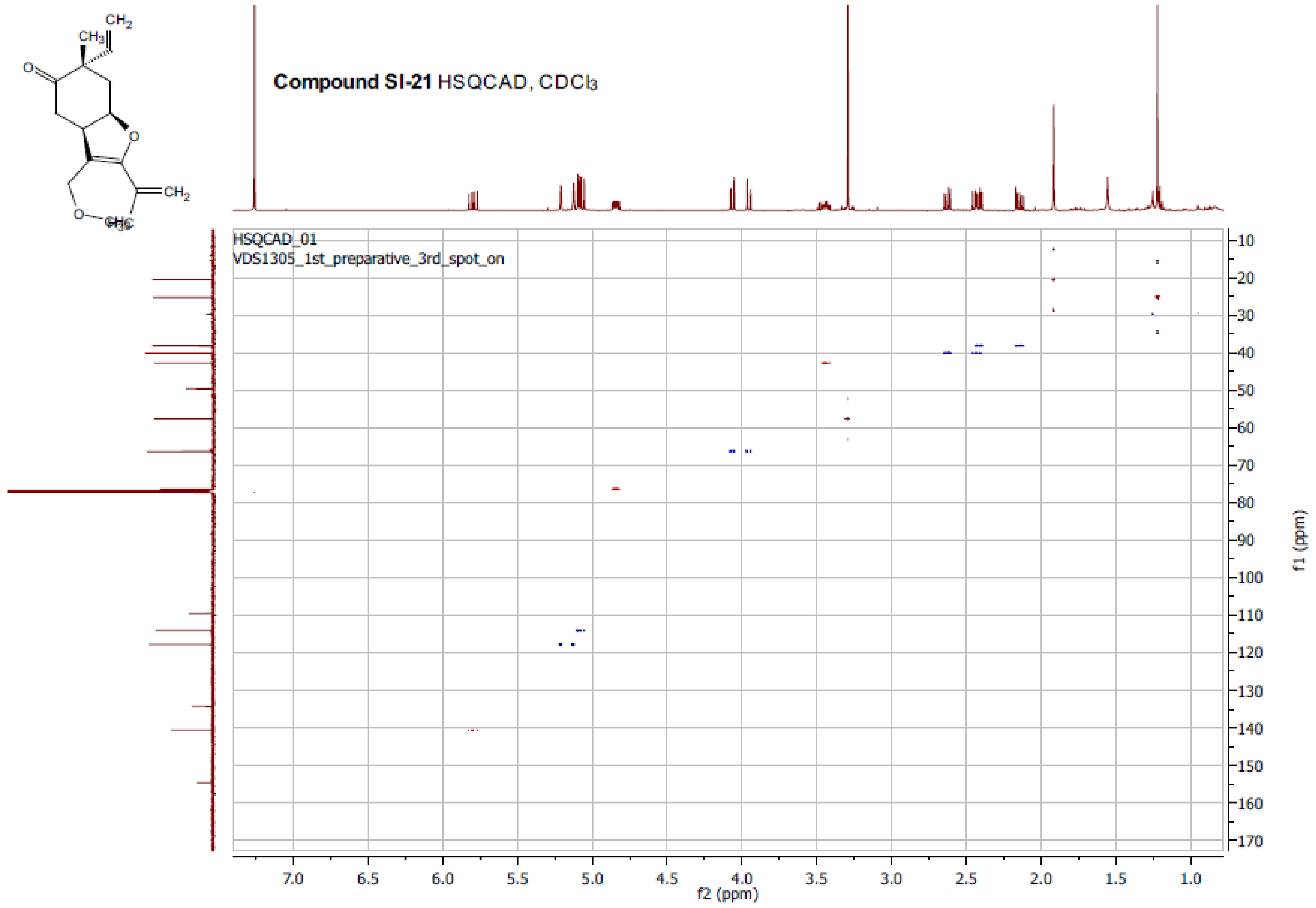
Compound SI-21 HSQCAD, CDCl3
HSQCAD_01
VDS1305_1st_preparative_3rd_spot_on
f2 (ppm)
f1 (ppm)

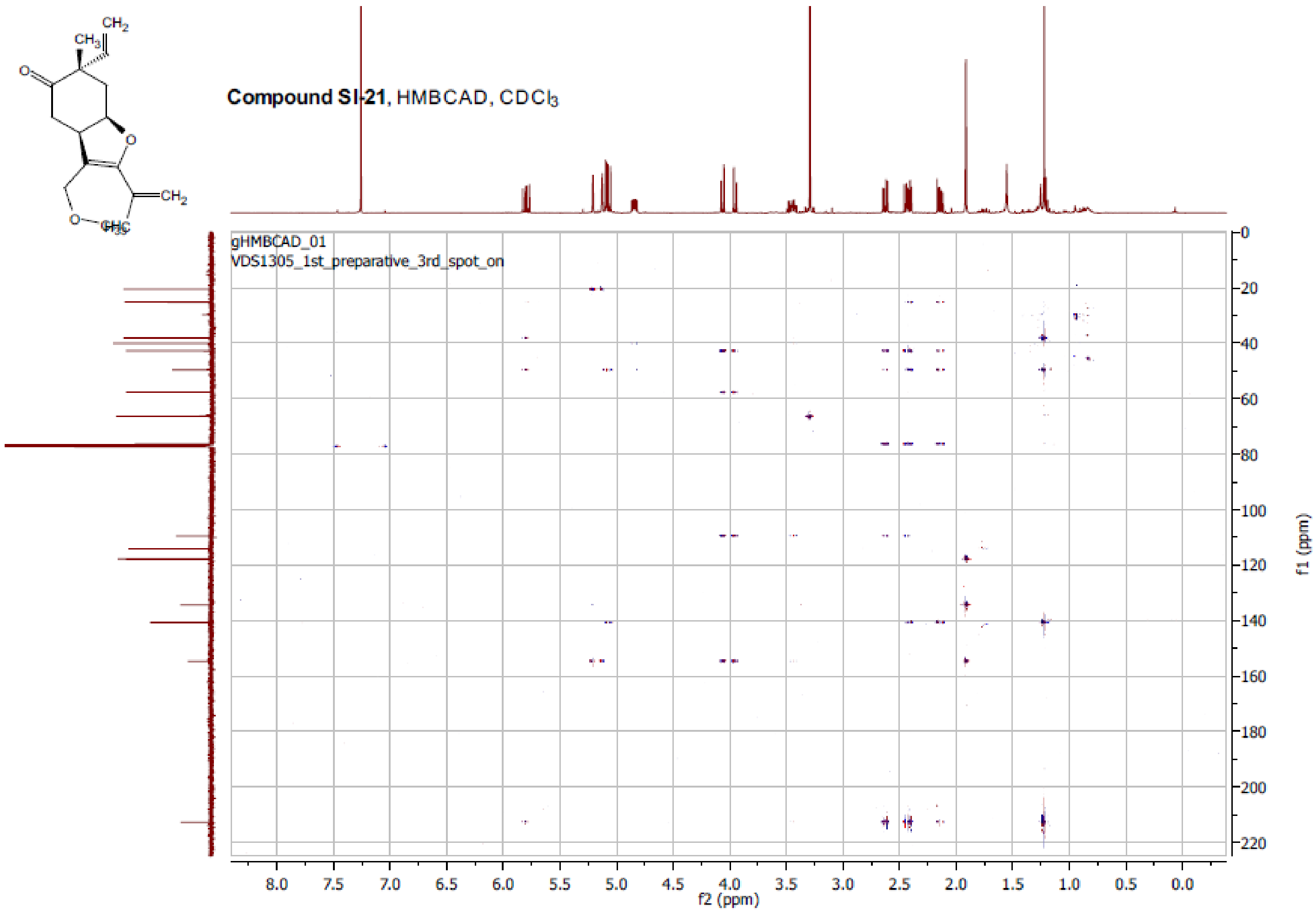
Compound SI-21, HMBCAD, CDCl3
gHMBCAD_01
VDS1305_1st_preparative_3rd_spot_on
f2 (ppm)
f1 (ppm)

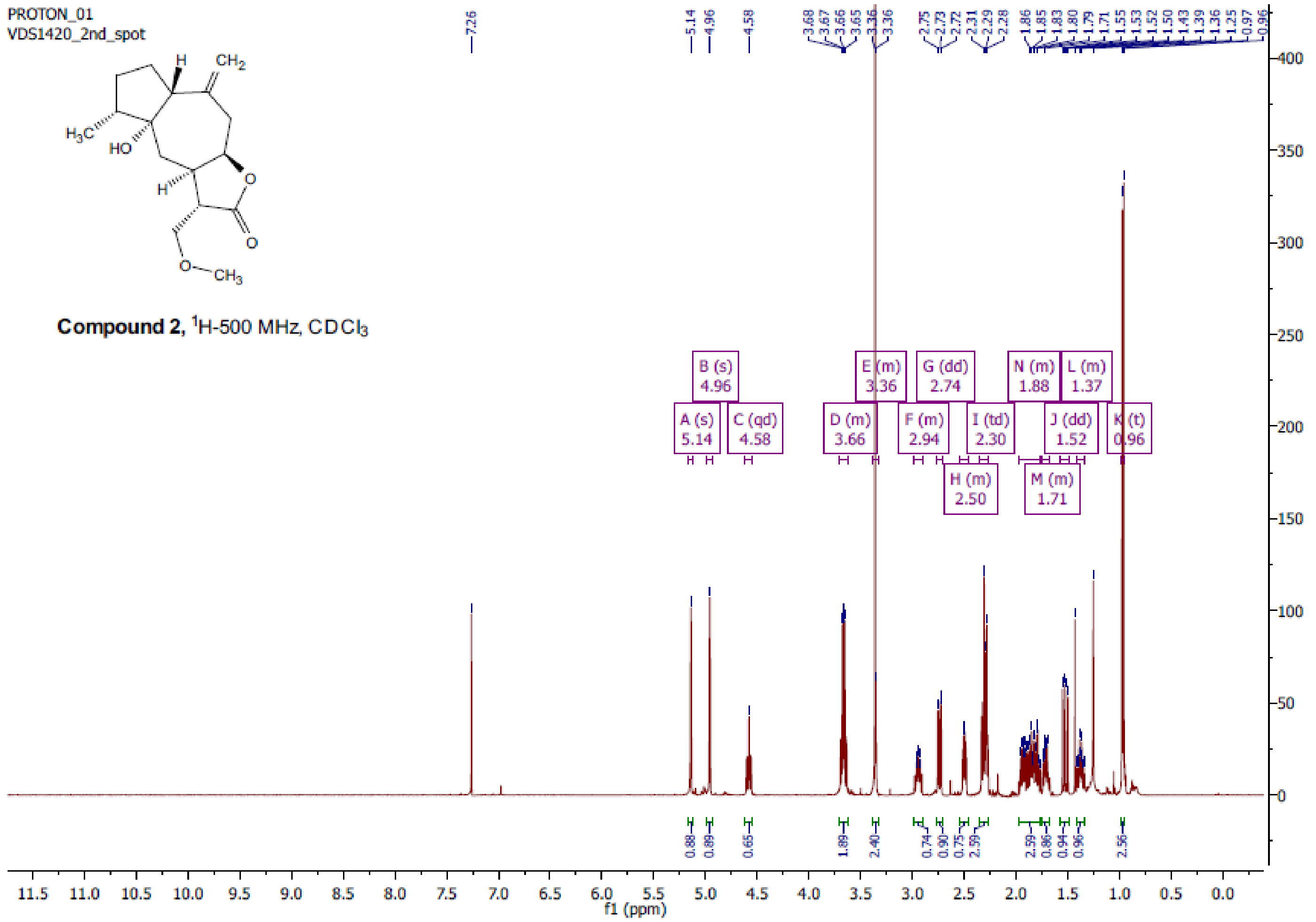
PROTON_01
VDS1420_2nd_spot
Compound 2, ¹H-500 MHz, CDCl3
f1 (ppm)

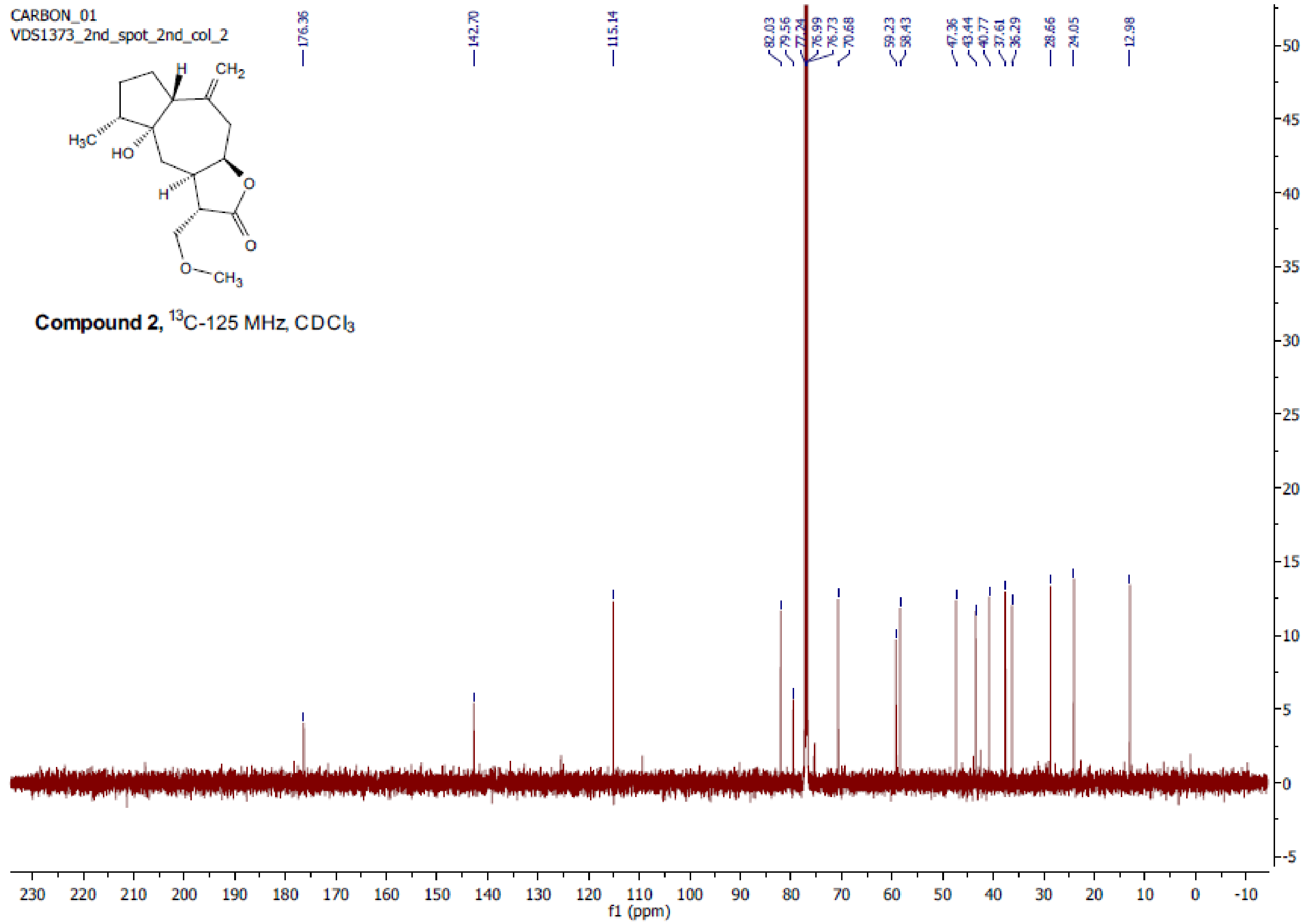
CARBON_01
VDS1373_2nd_spot_2nd_col_2
Compound 2, ¹³C-125 MHz, CDCl3
f1 (ppm)

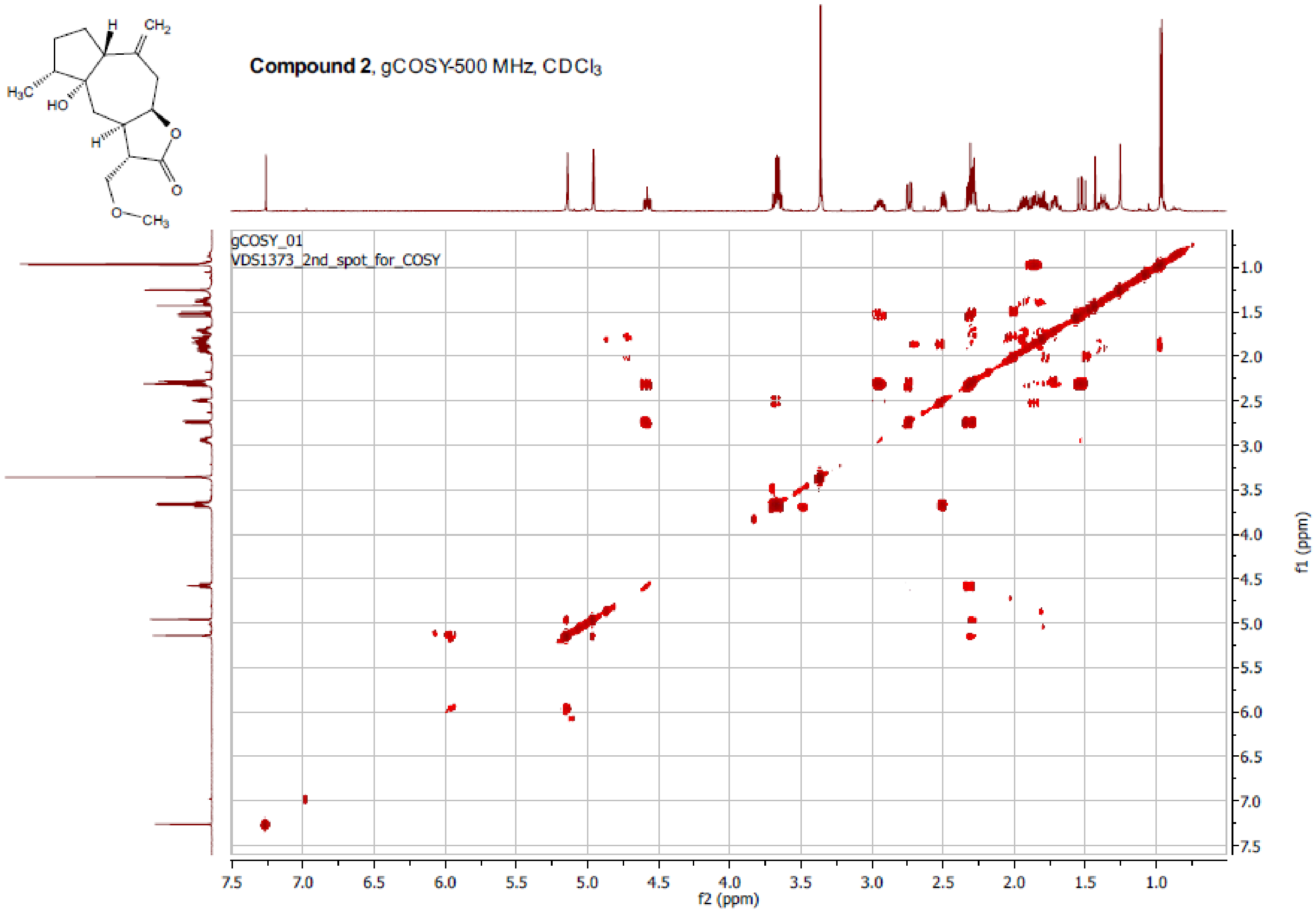
Compound 2, gCOSY-500 MHz, CDCl3
gCOSY_01
VDS1373_2nd_spot_for_COSY
f2 (ppm)
f1 (ppm)

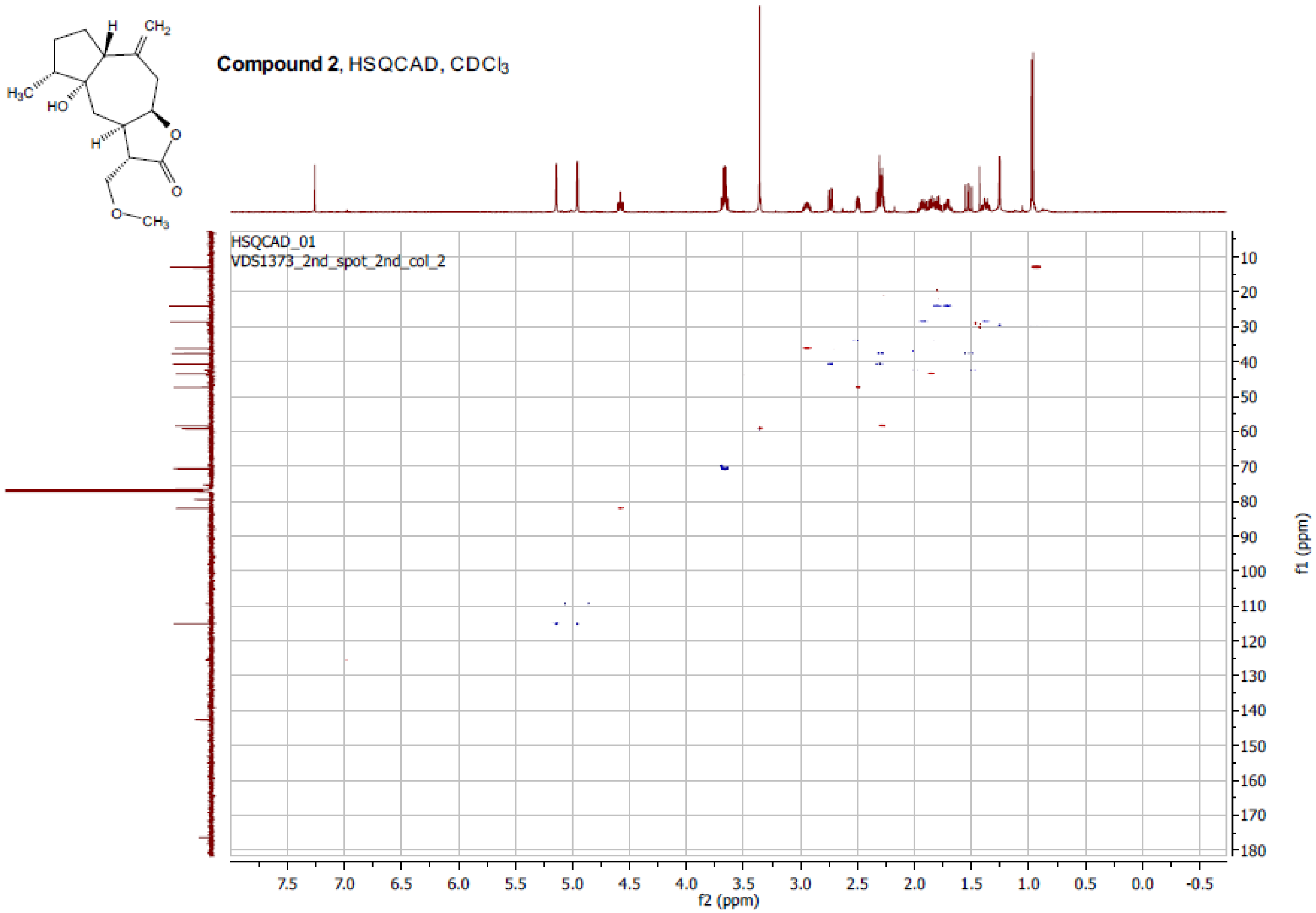
Compound 2, HSQCAD, CDCl3
HSQCAD_01
VDS1373_2nd_spot_2nd_col_2
f2 (ppm)
f1 (ppm)

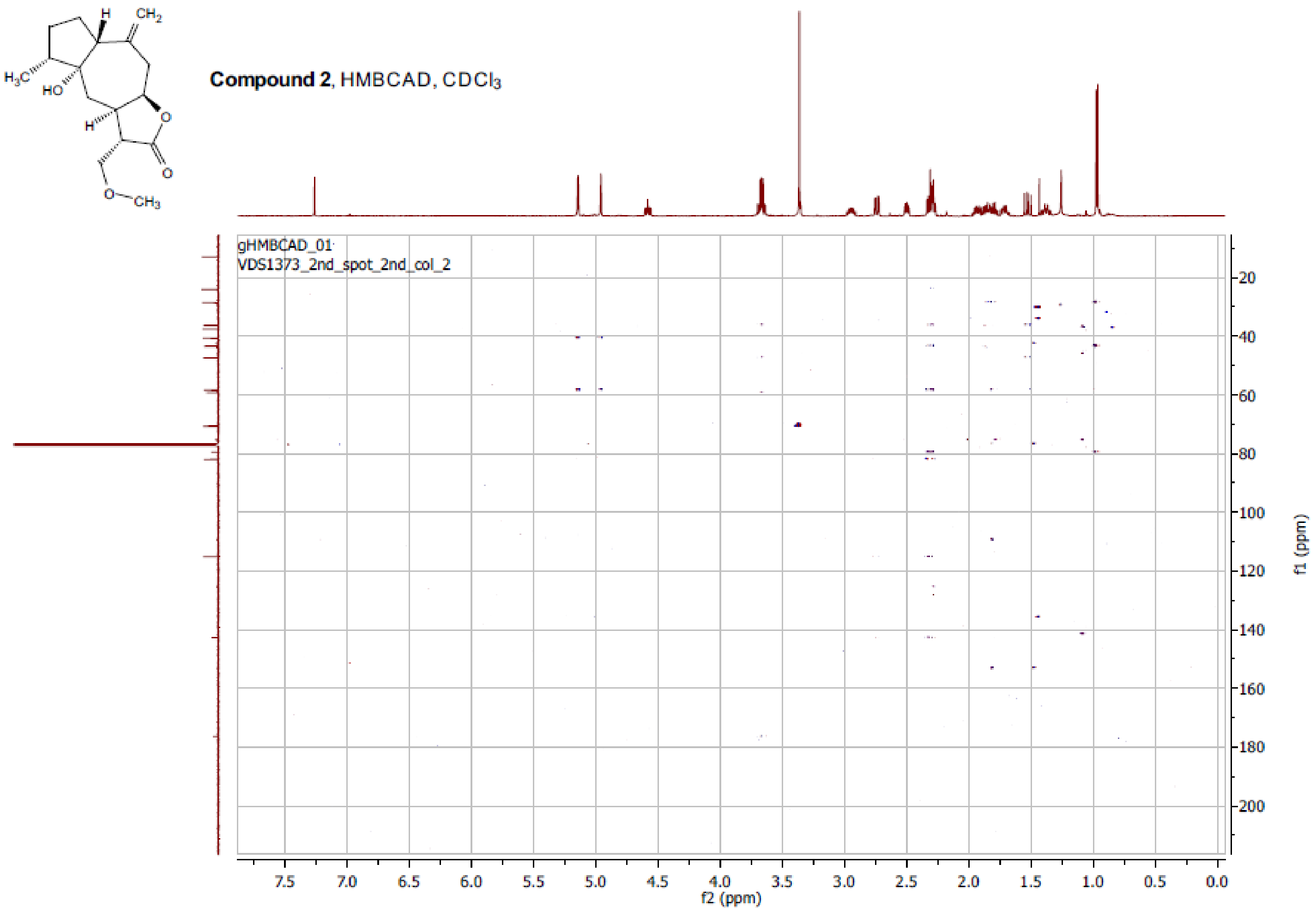

Compound 2, HMBCAD, CDCl3
gHMBCAD_01
VDS1373_2nd_spot_2nd_col_2
f2 (ppm)
f1 (ppm)

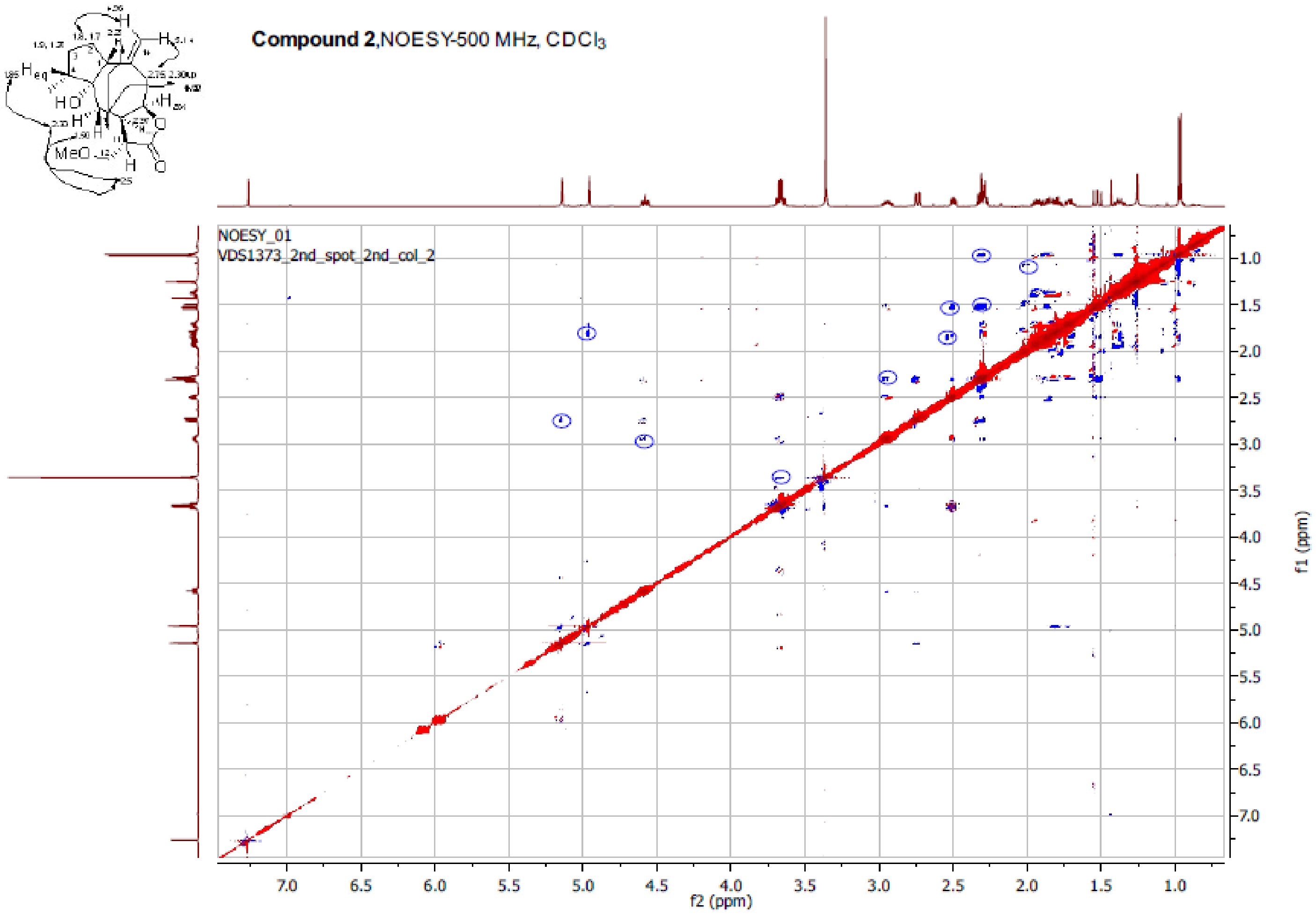

Compound 2,NOESY-500 MHz, CDCl3
NOESY_01
VDS1373_2nd_spot_2nd_col_2
f2 (ppm)
f1 (ppm)

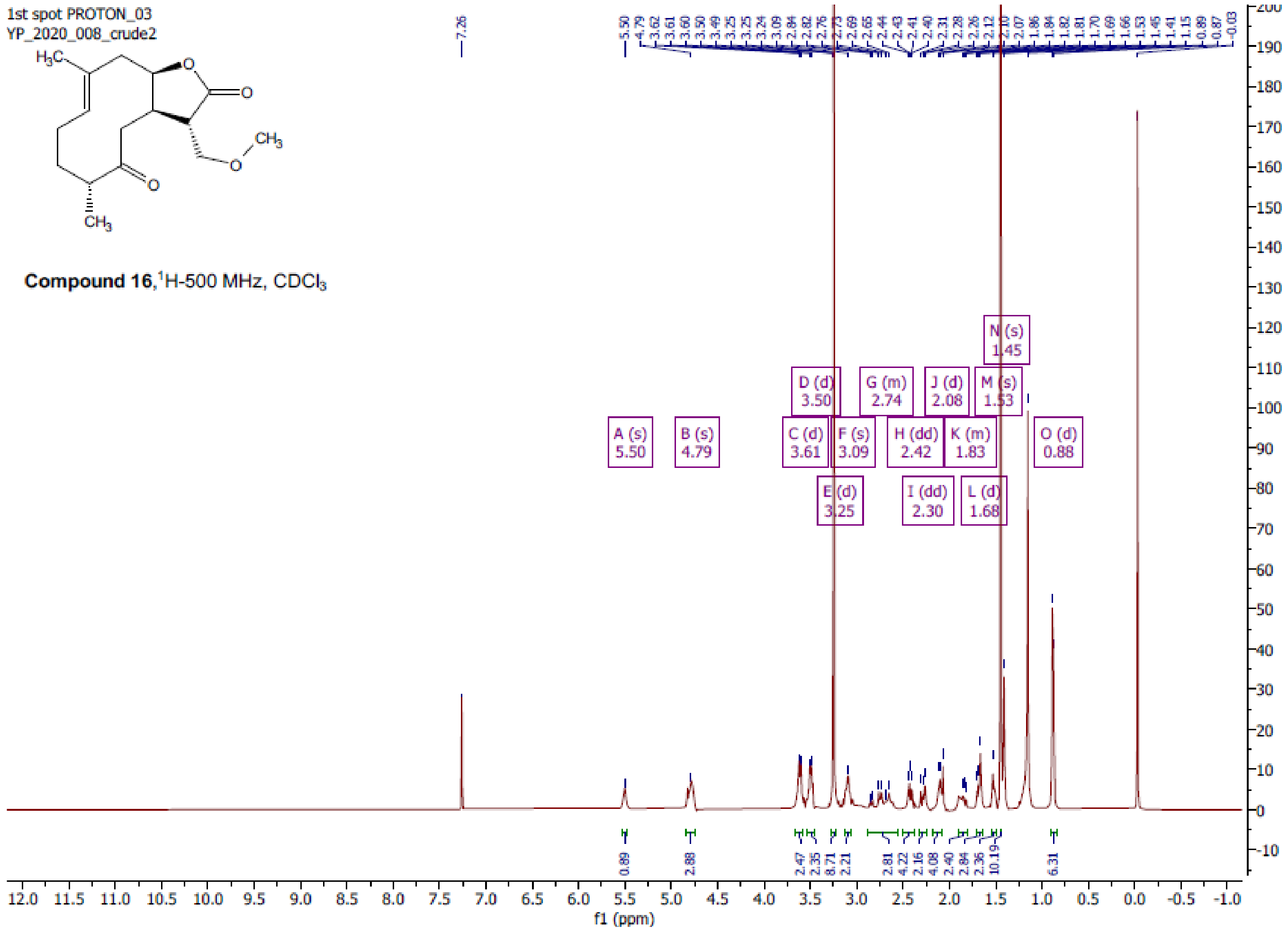

Compound 16, ¹H-500 MHz, $CDCl_3$

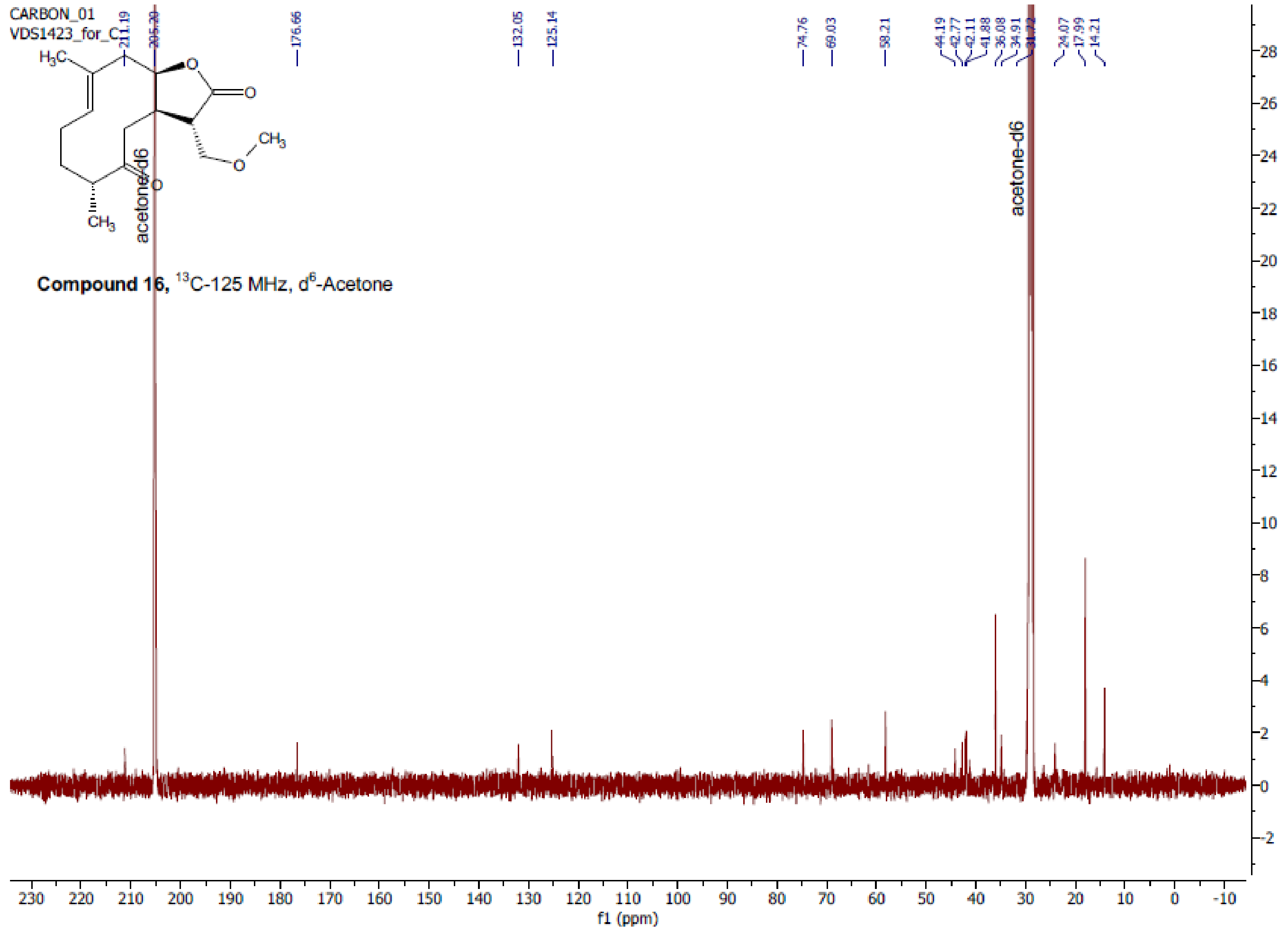

Compound 16, ¹³C-125 MHz, d⁶-Acetone

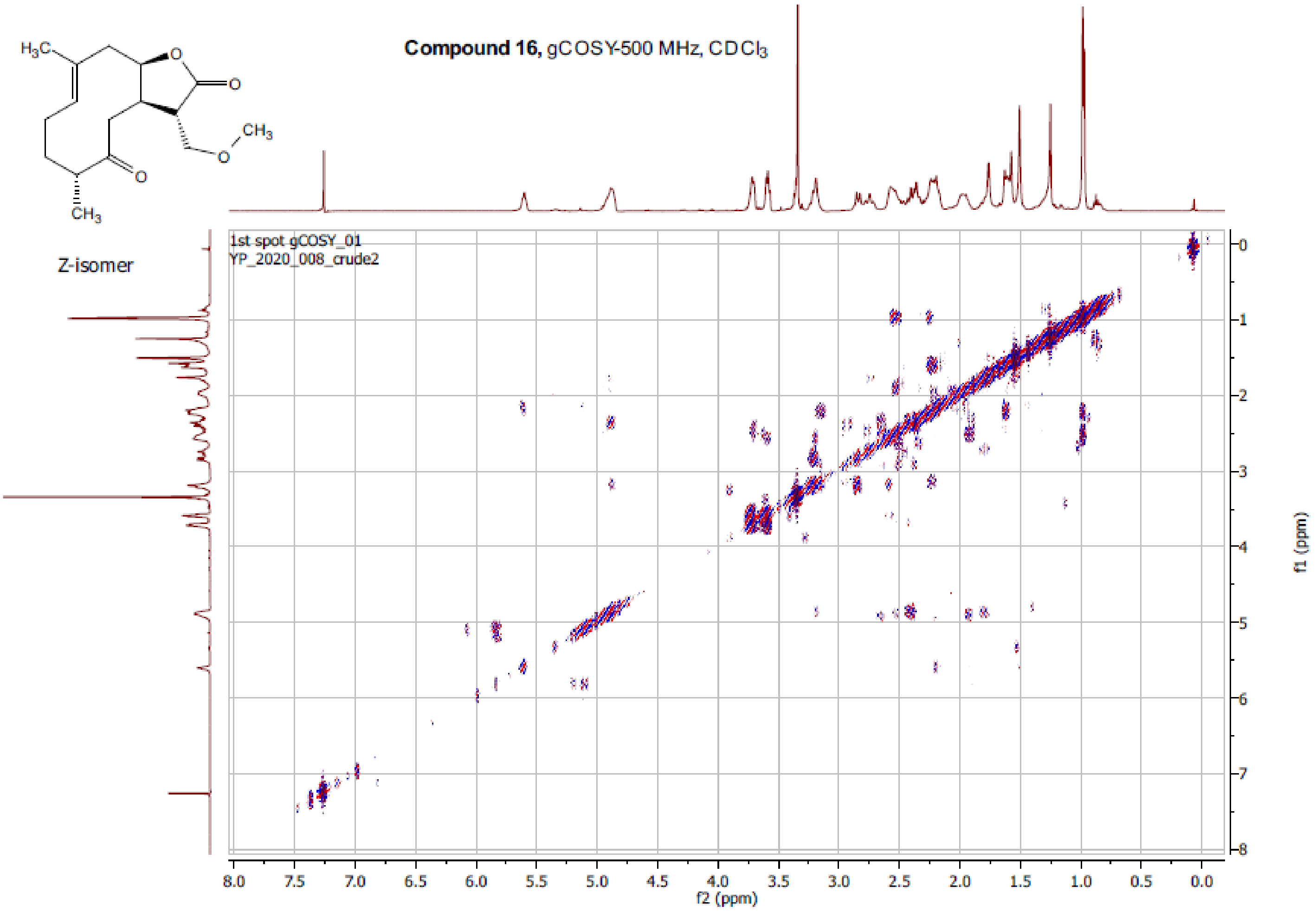
Compound 16, gCOSY-500 MHz, CDCl3
Z-isomer
1st spot gCOSY_01
YP_2020_008_crude2
f2 (ppm)
f1 (ppm)

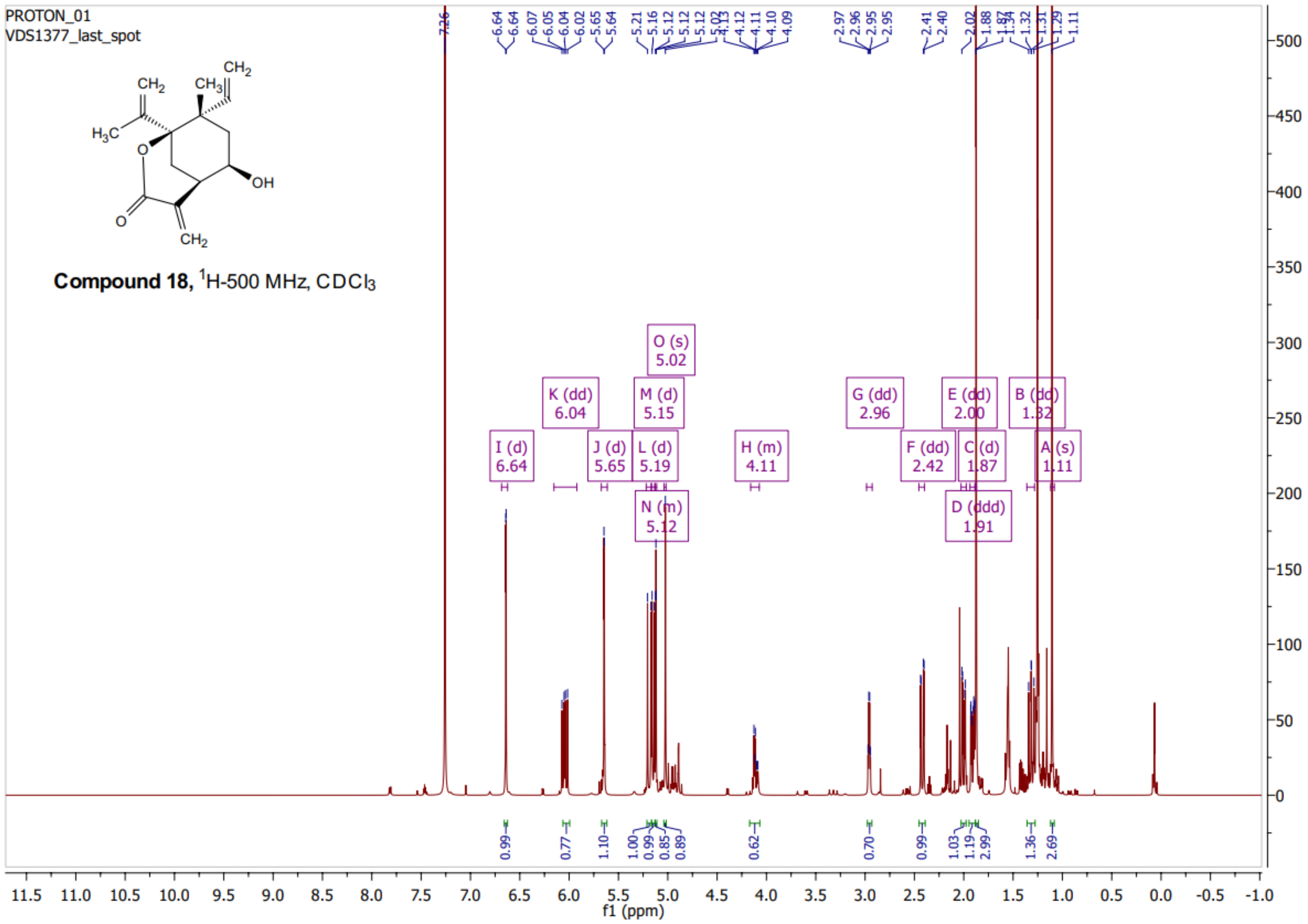
PROTON_01
VDS1377_last_spot
Compound 18, 1H-500 MHz, CDCl3
I (d) 6.64
K (dd) 6.04
J (d) 5.65
M (d) 5.15
L (d) 5.19
O (s) 5.02
N (m) 5.12
H (m) 4.11
G (dd) 2.96
F (dd) 2.42
E (dd) 2.00
C (d) 1.87
D (ddd) 1.91
B (dd) 1.32
A (s) 1.11
f1 (ppm)

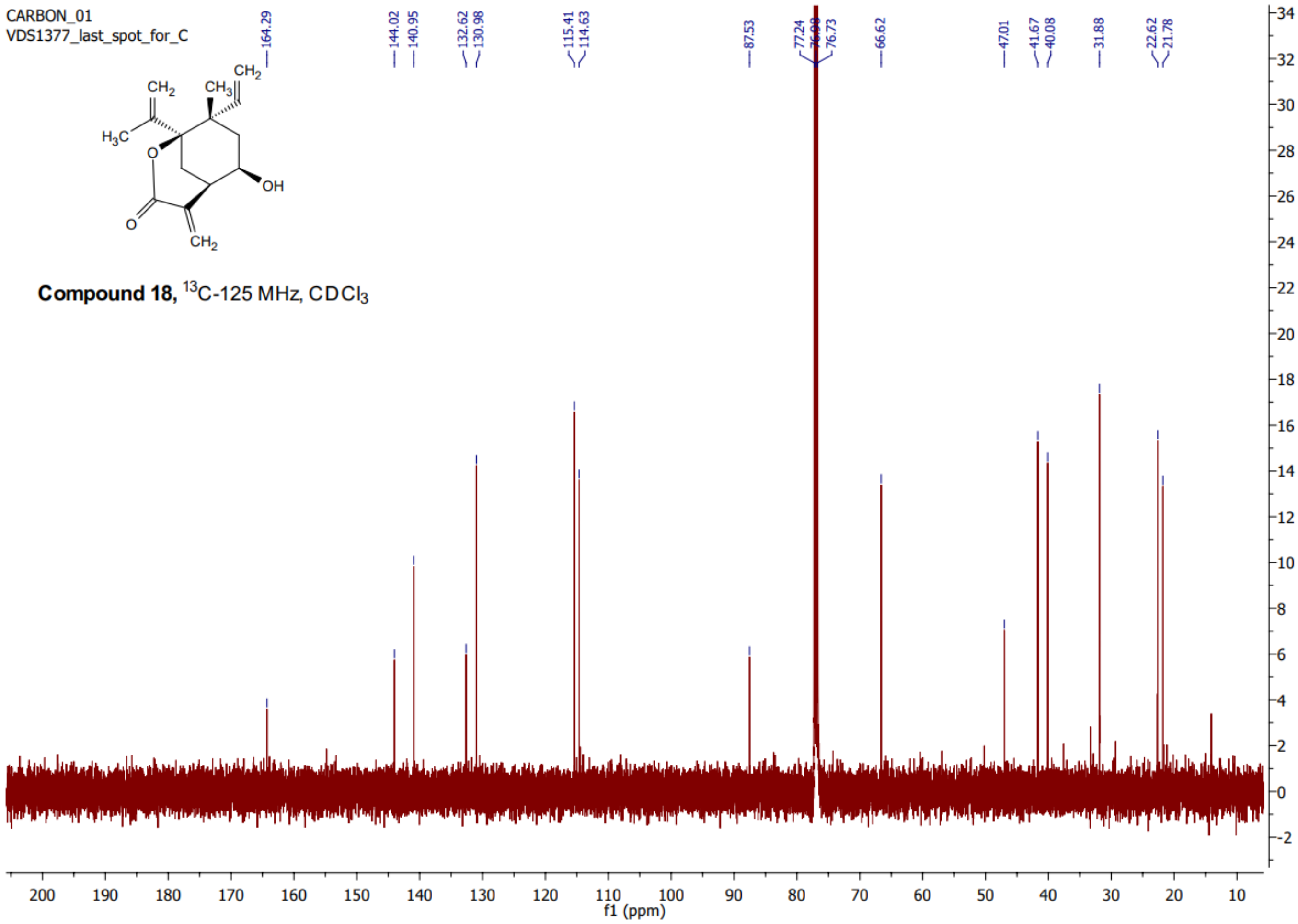
CARBON_01
VDS1377_last_spot_for_C
Compound 18, 13C-125 MHz, CDCl3
164.29
144.02
140.95
132.62
130.98
115.41
114.63
87.53
77.24
76.73
66.62
47.01
41.67
40.08
31.88
22.62
21.78
f1 (ppm)

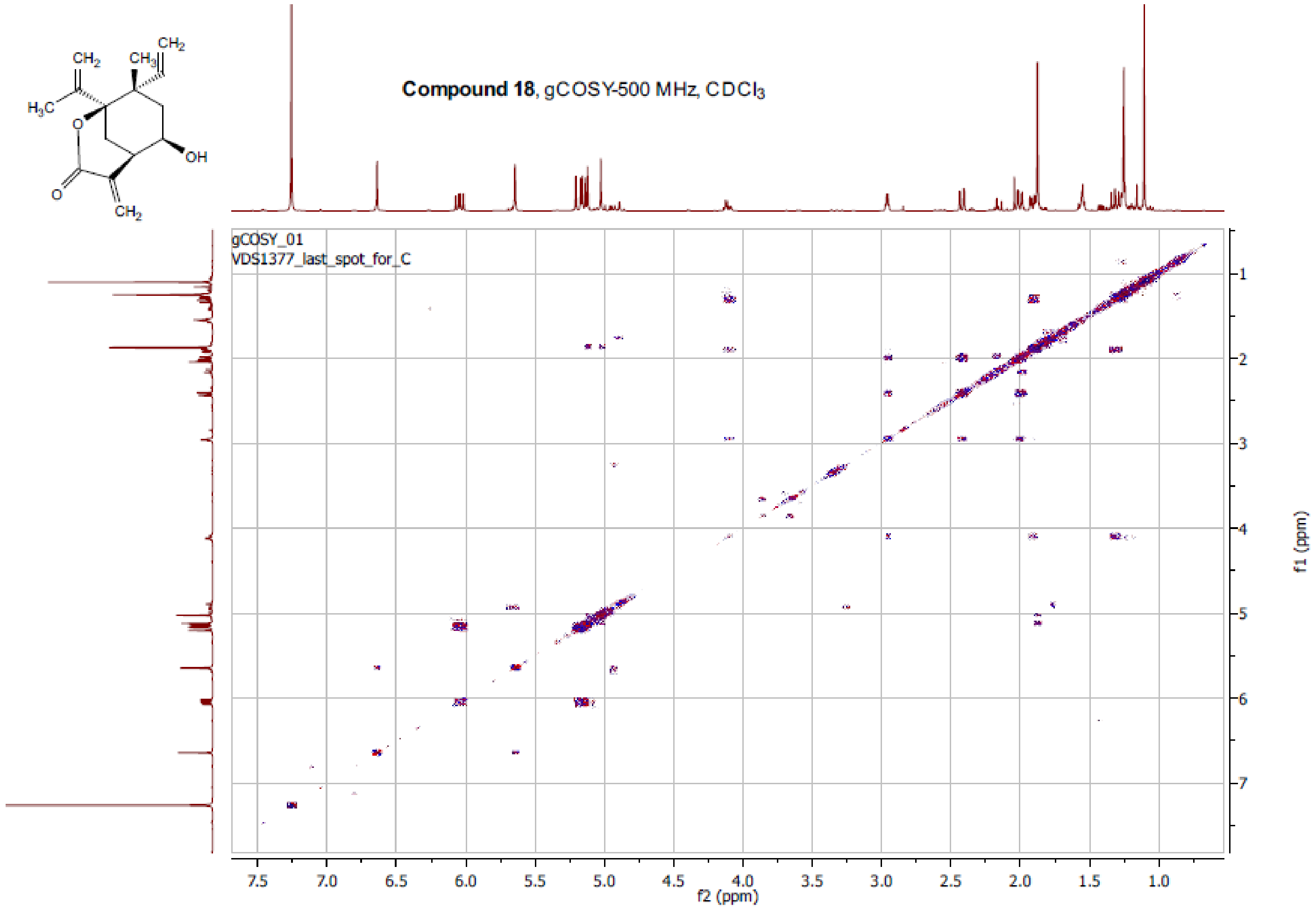
Compound 18, gCOSY-500 MHz, CDCl3
gCOSY_01
VDS1377_last_spot_for_C
f2 (ppm)
f1 (ppm)

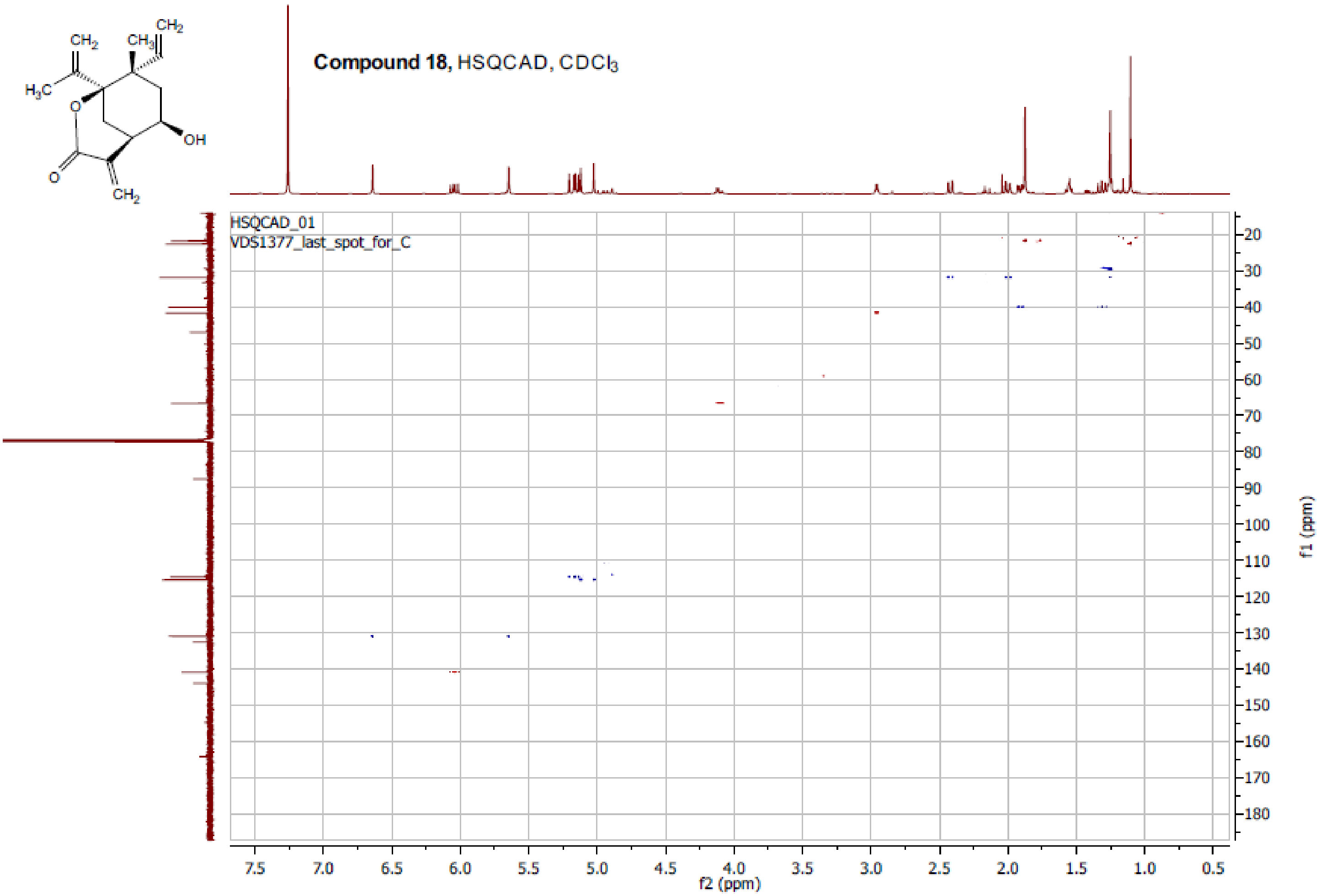
Compound 18, HSQCAD, CDCl3
HSQCAD_01
VDS1377_last_spot_for_C
f2 (ppm)
f1 (ppm)

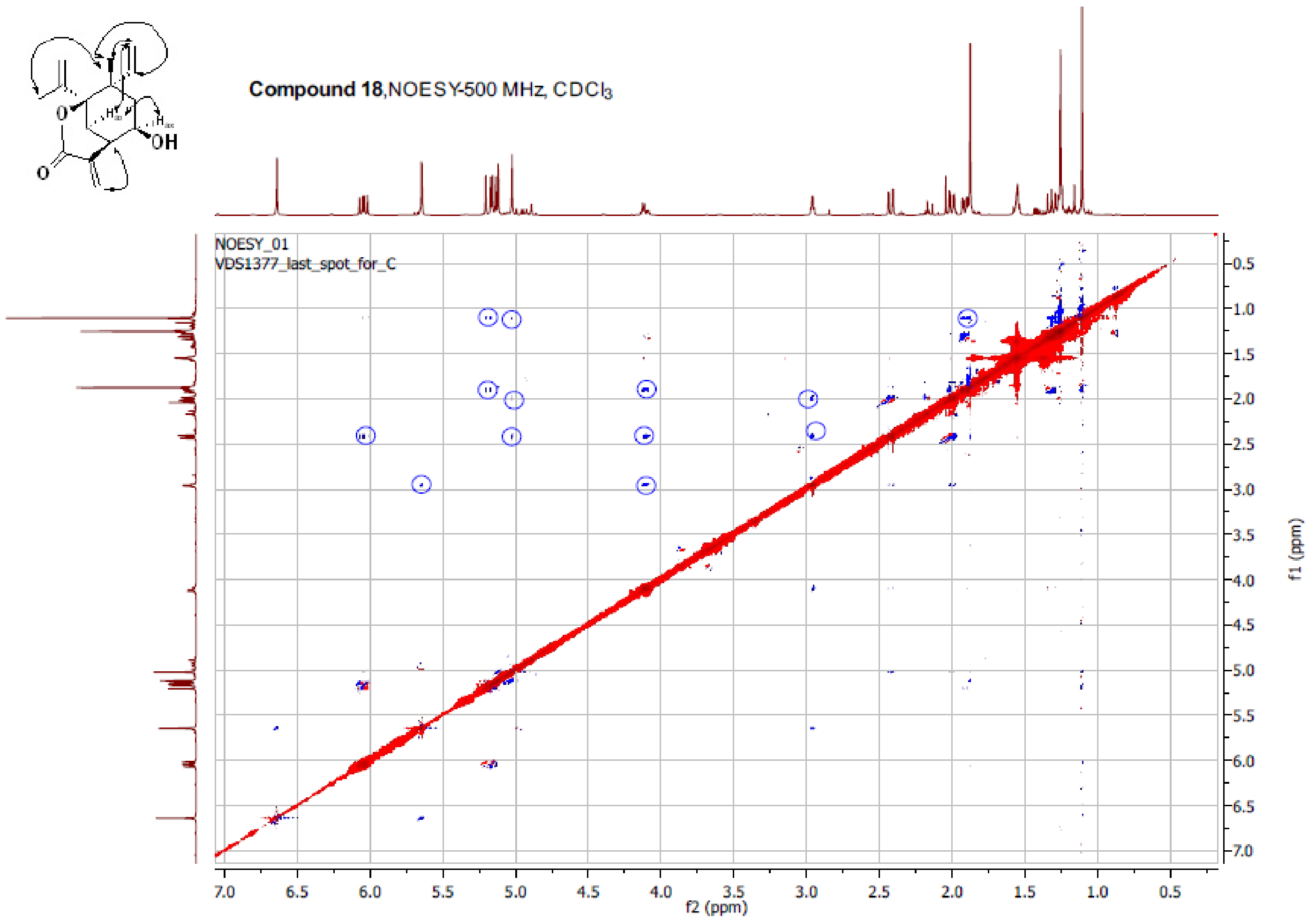
Compound 18,NOESY-500 MHz, CDCl3
NOESY_01
VDS1377_last_spot_for_C
f2 (ppm)
f1 (ppm)

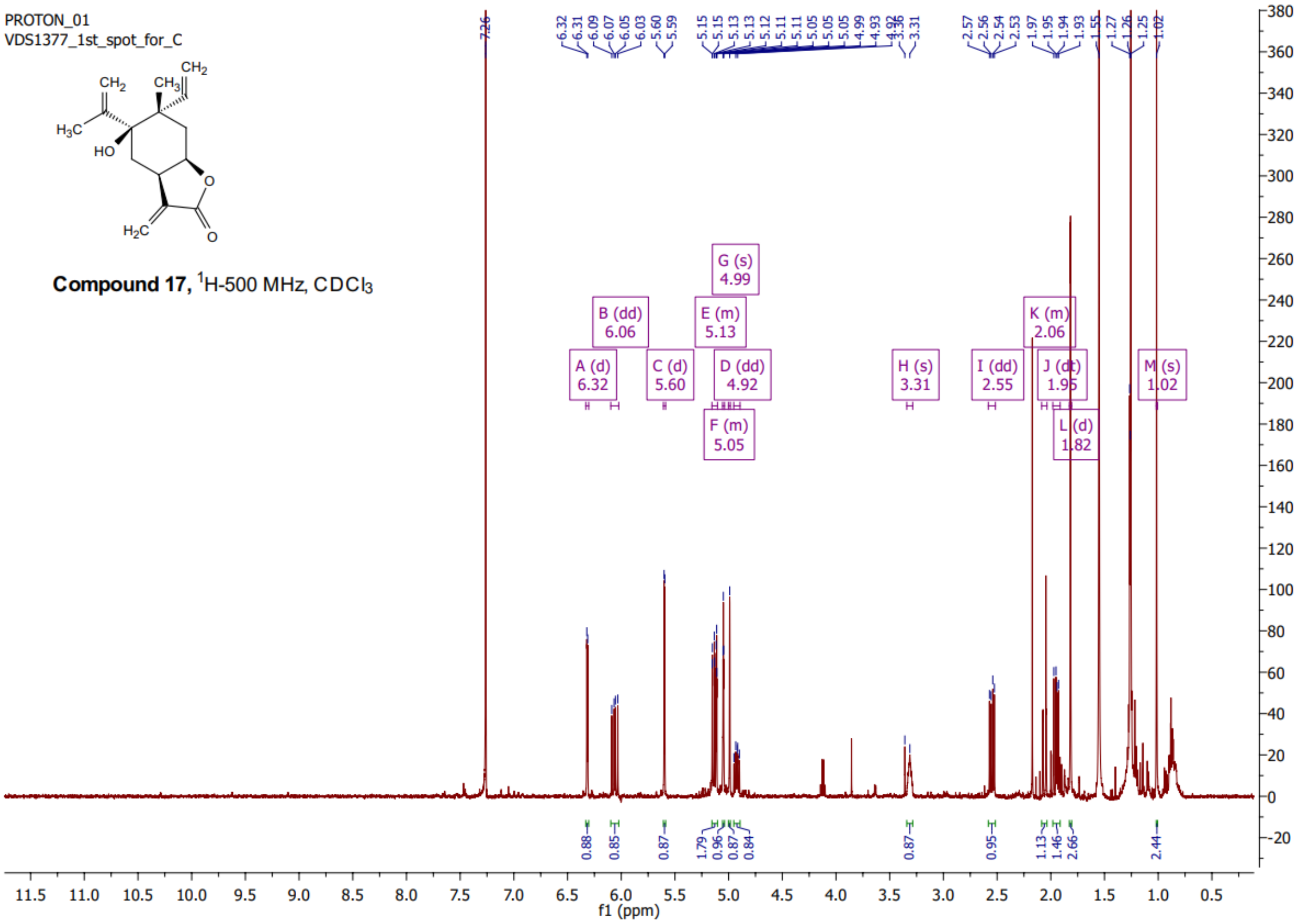
PROTON_01
VDS1377_1st_spot_for_C
Compound 17, 1H-500 MHz, CDCl3
A (d) 6.32
B (dd) 6.06
C (d) 5.60
D (dd) 4.92
E (m) 5.13
F (m) 5.05
G (s) 4.99
H (s) 3.31
I (dd) 2.55
J (dt) 1.95
K (m) 2.06
L (d) 1.82
M (s) 1.02
f1 (ppm)

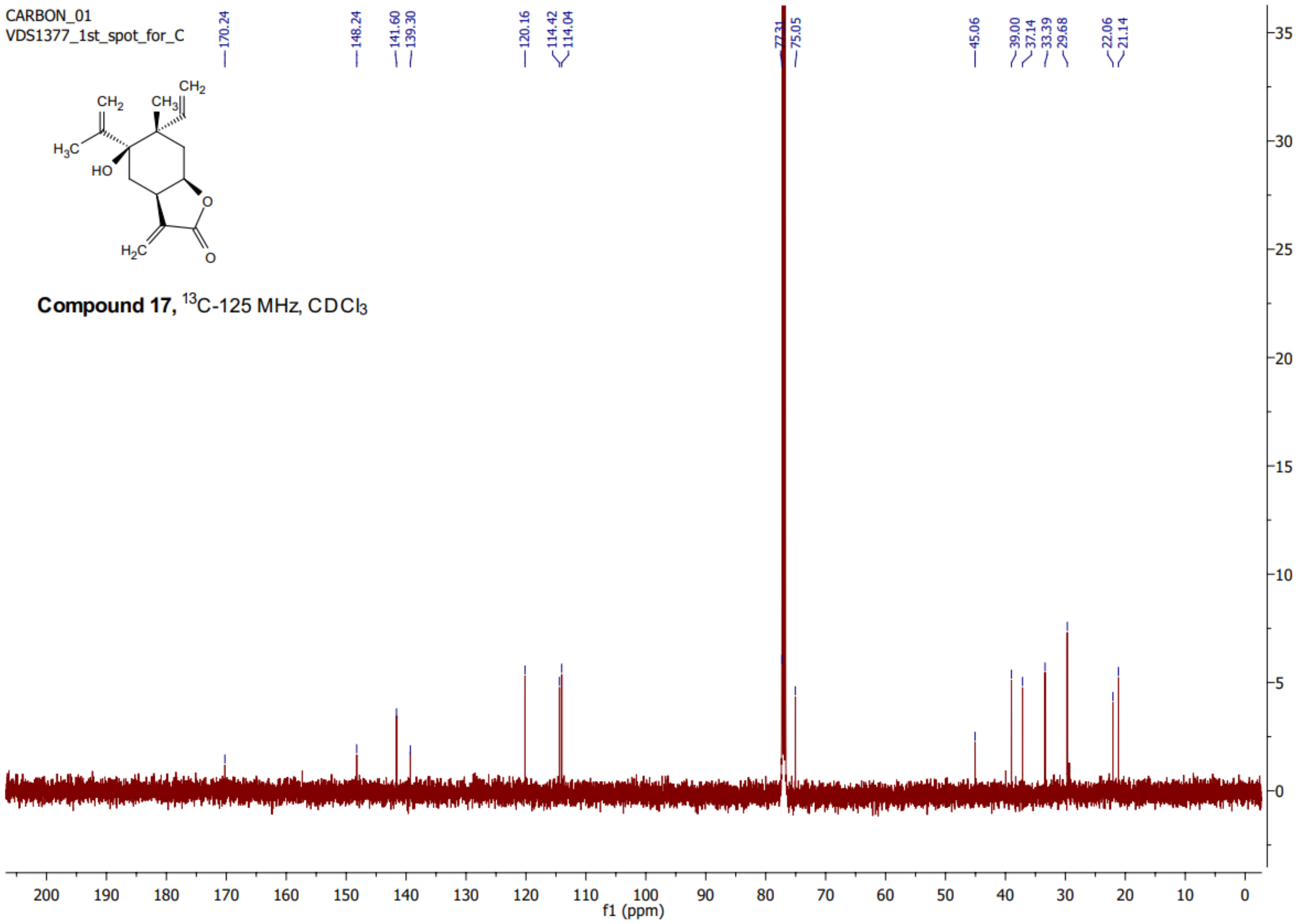
CARBON_01
VDS1377_1st_spot_for_C
Compound 17, 13C-125 MHz, CDCl3
170.24
148.24
141.60
139.30
120.16
114.42
114.04
77.31
75.05
45.06
39.00
37.14
33.39
29.68
22.06
21.14
f1 (ppm)

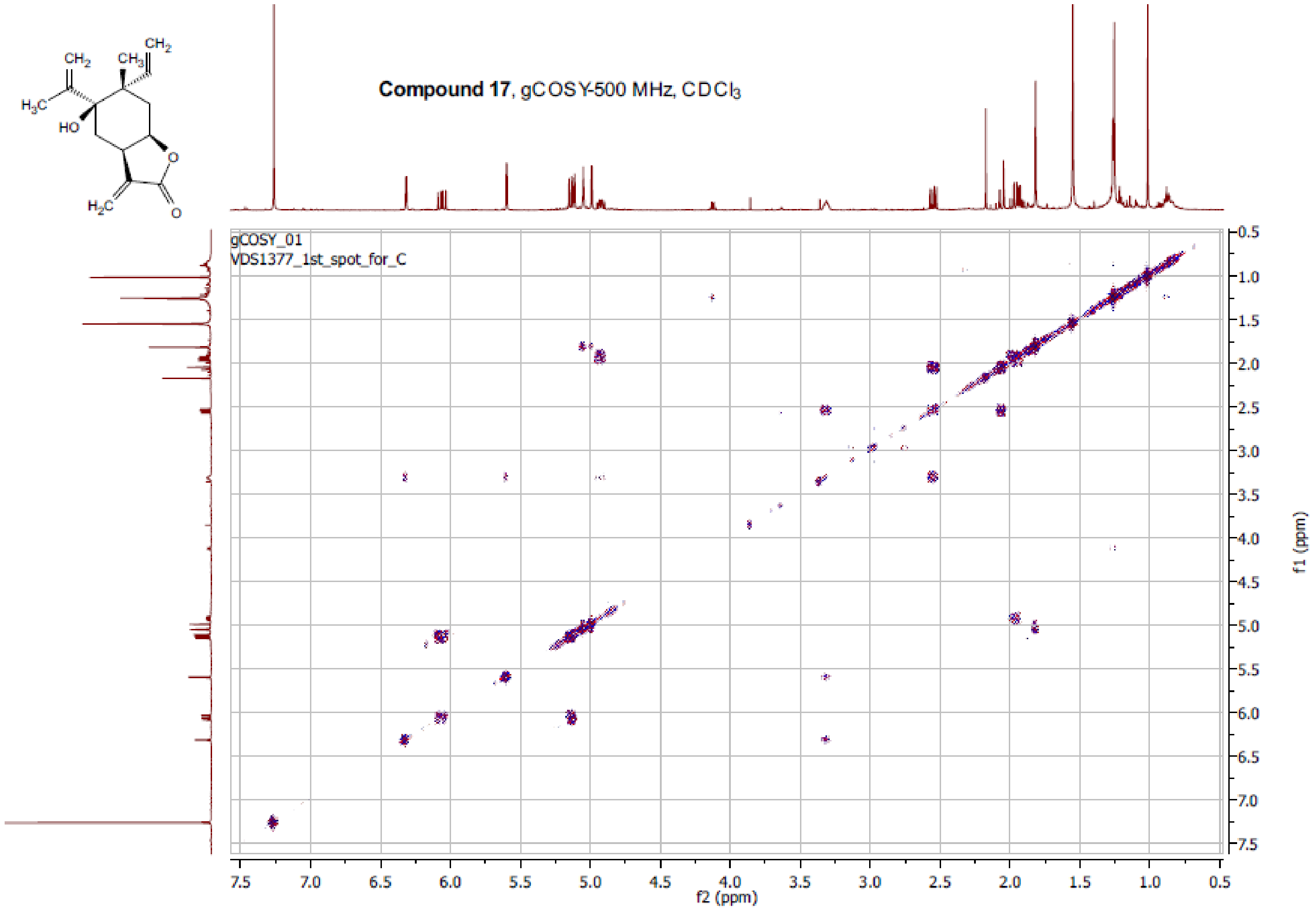
Compound 17, gCOSY-500 MHz, CDCl3
gCOSY_01
VDS1377_1st_spot_for_C
f2 (ppm)
f1 (ppm)

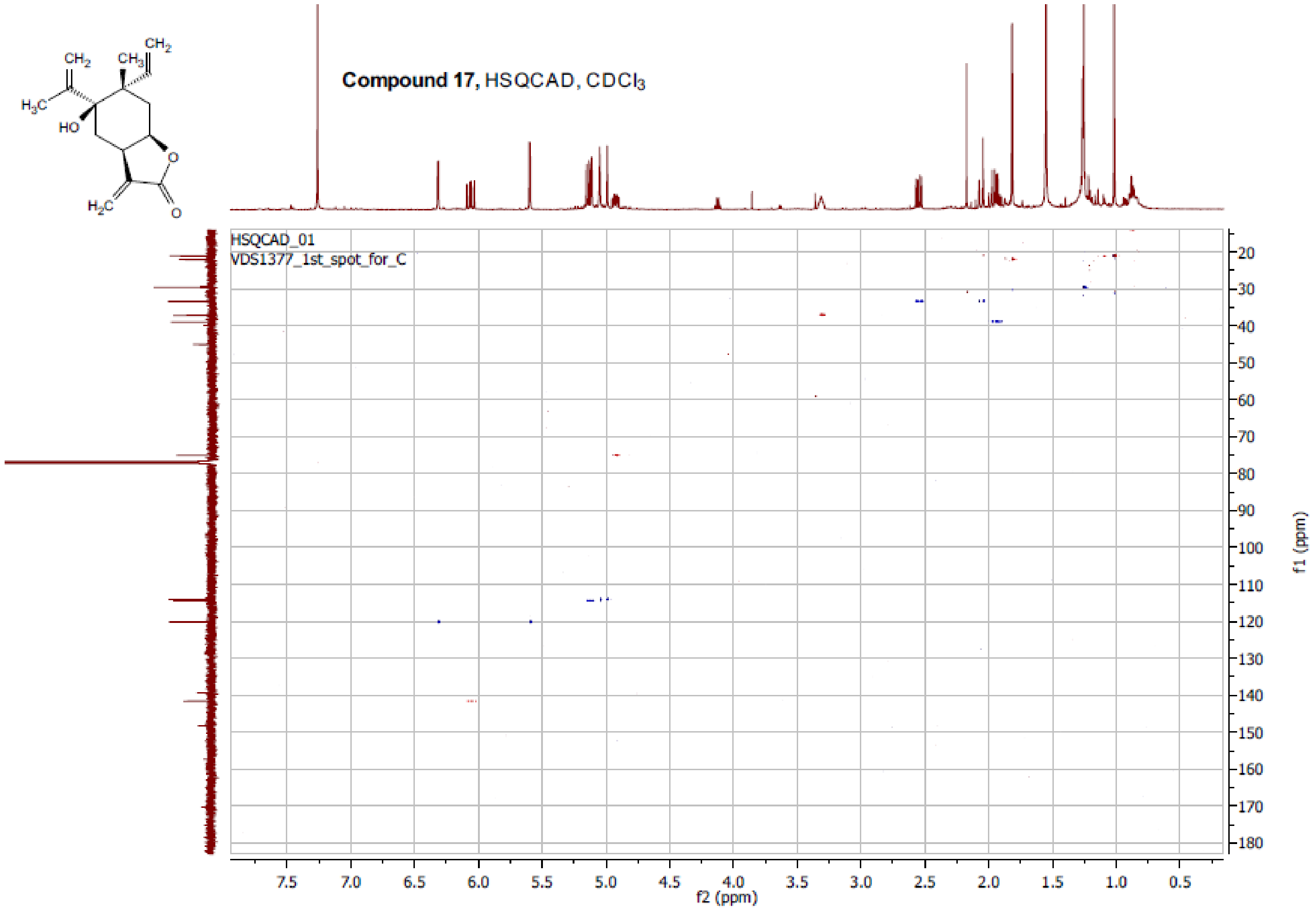
Compound 17, HSQCAD, CDCl3
HSQCAD_01
VDS1377_1st_spot_for_C
f2 (ppm)
f1 (ppm)

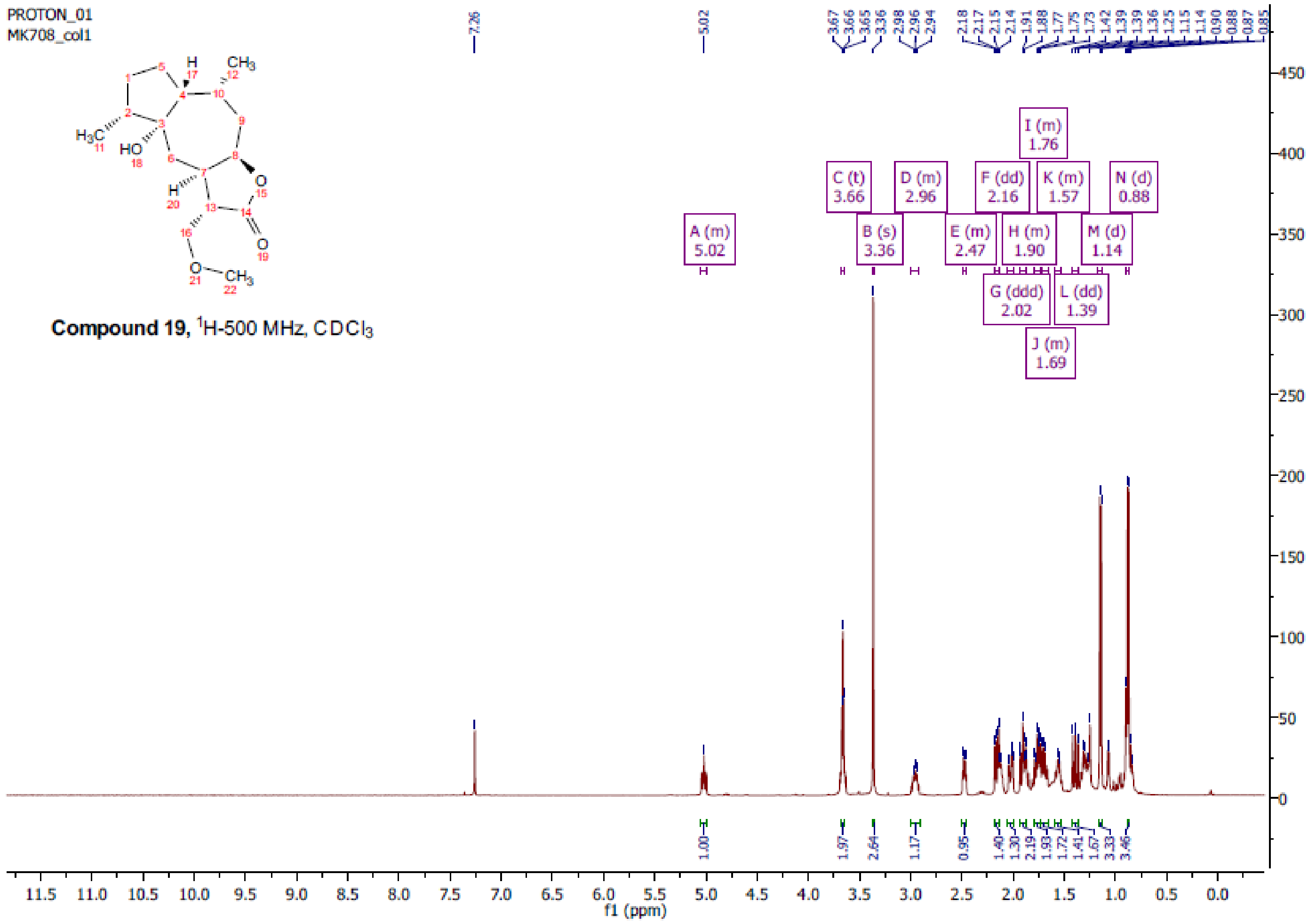

Compound 19, ^{1}H-500 MHz, $CDCl_3$

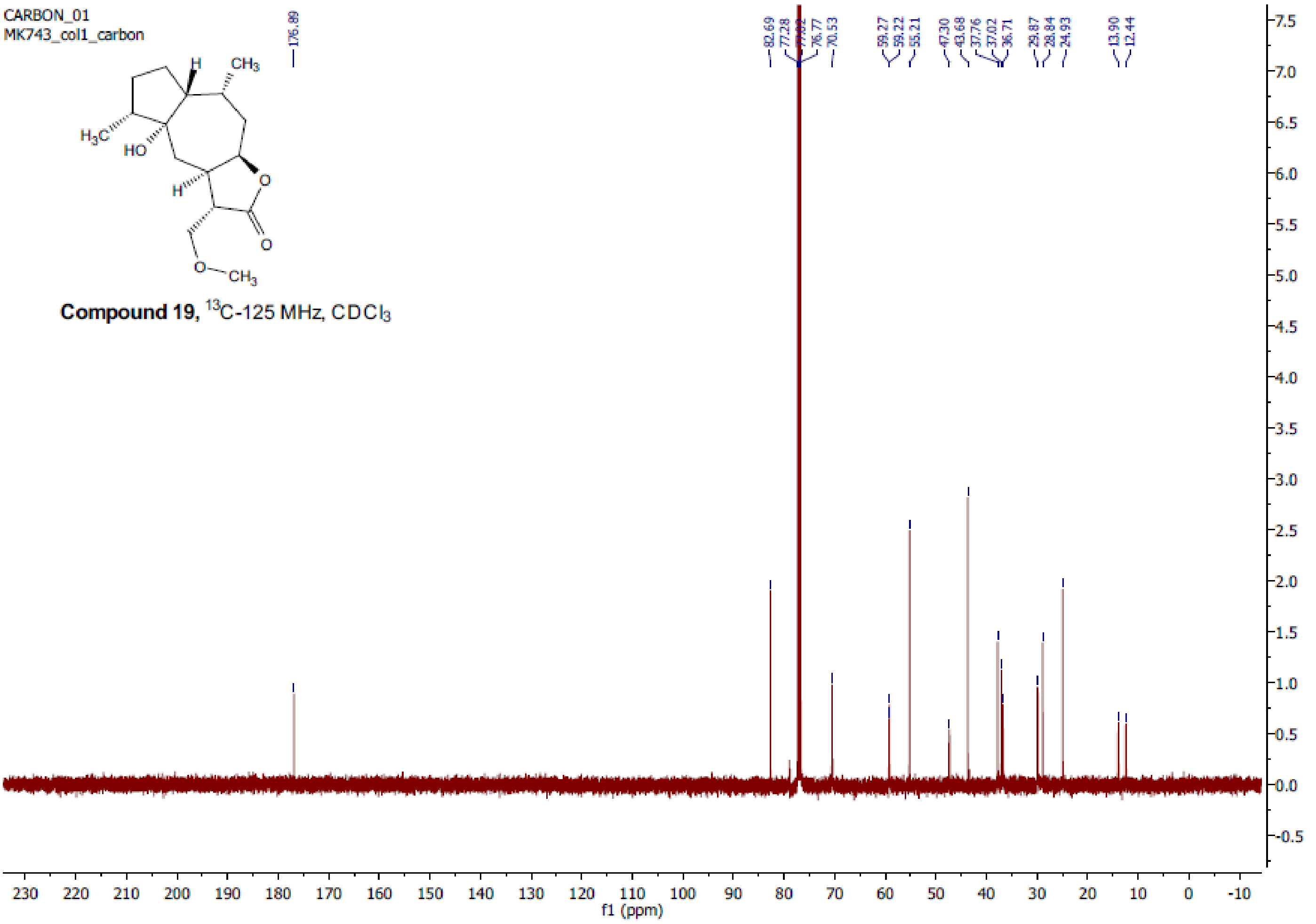

Compound 19, ^{13}C-125 MHz, $CDCl_3$

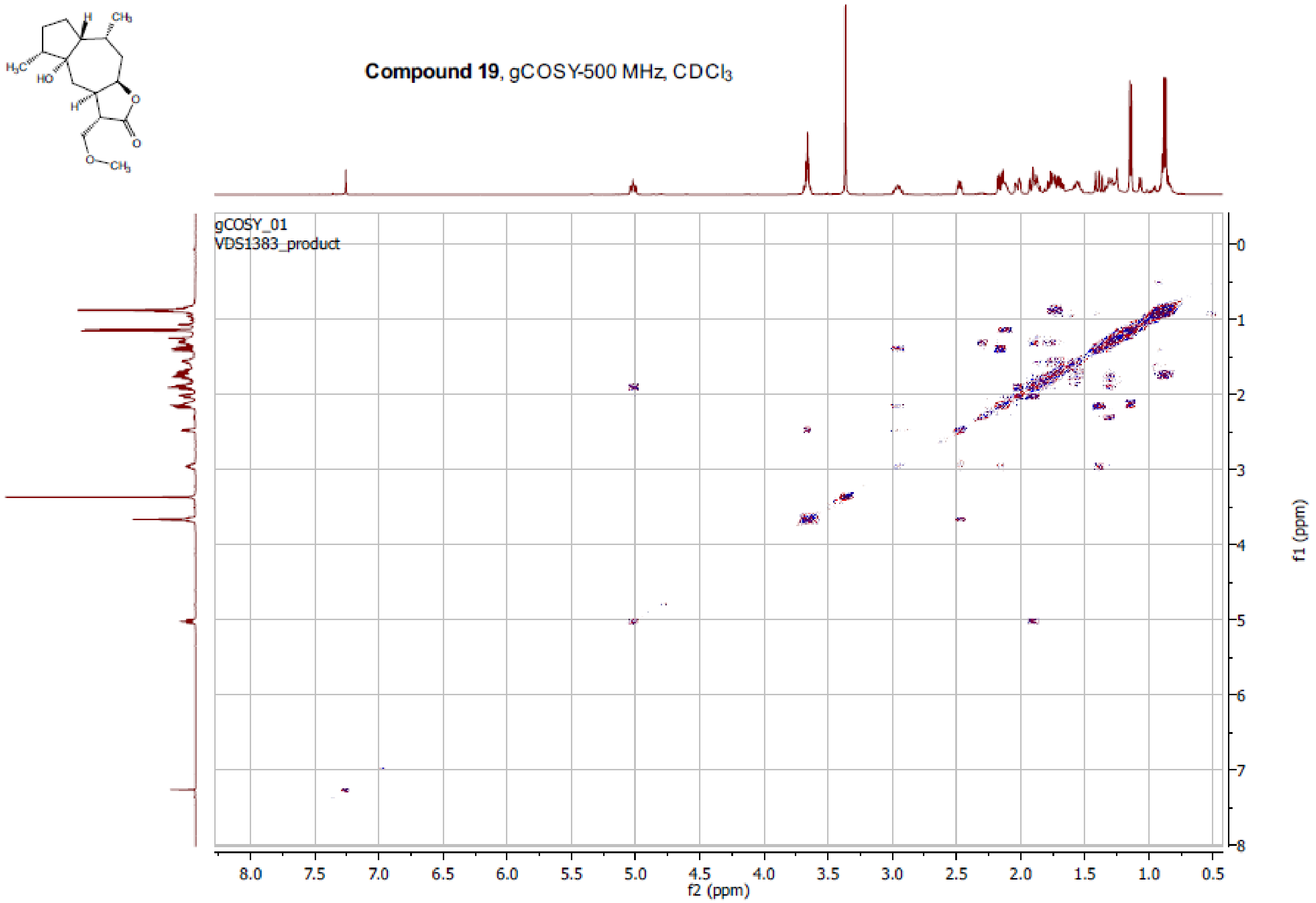
Compound 19, gCOSY-500 MHz, CDCl3
gCOSY_01
VDS1383_product
f2 (ppm)
f1 (ppm)

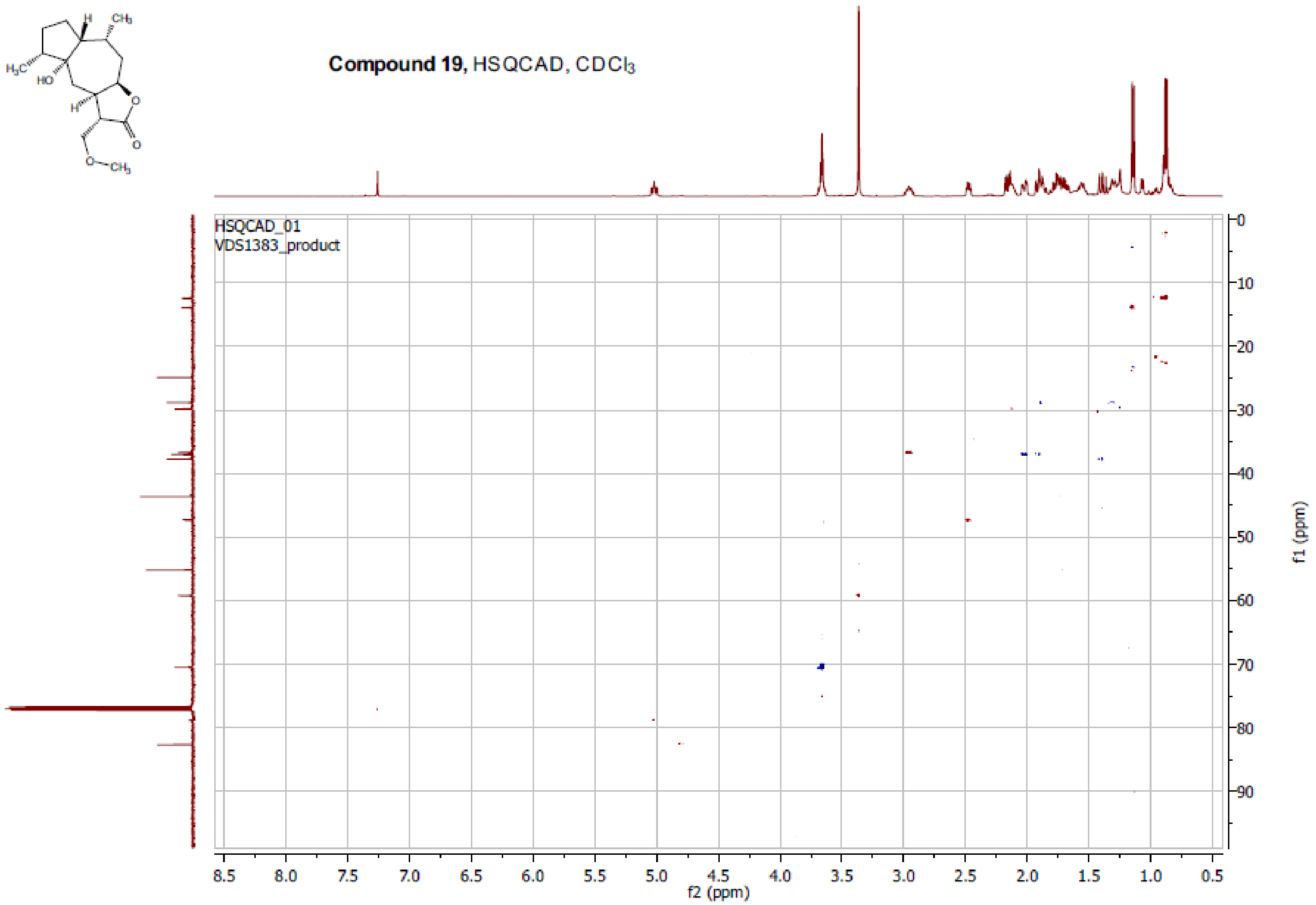
Compound 19, HSQCAD, CDCl3
HSQCAD_01
VDS1383_product
f2 (ppm)
f1 (ppm)

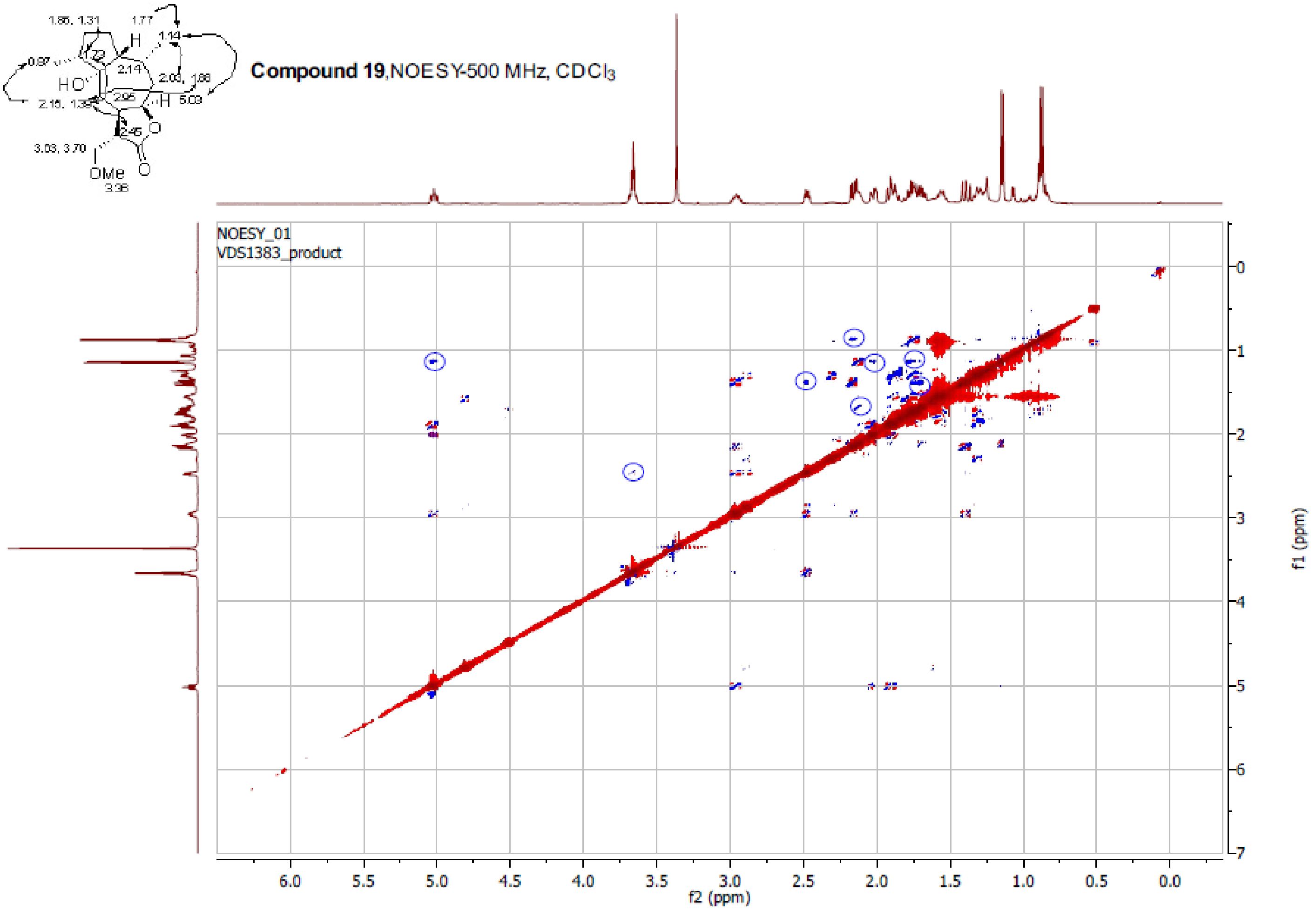

Compound 19,NOESY-500 MHz, CDCl3
NOESY_01
VDS1383_product
f2 (ppm)
f1 (ppm)

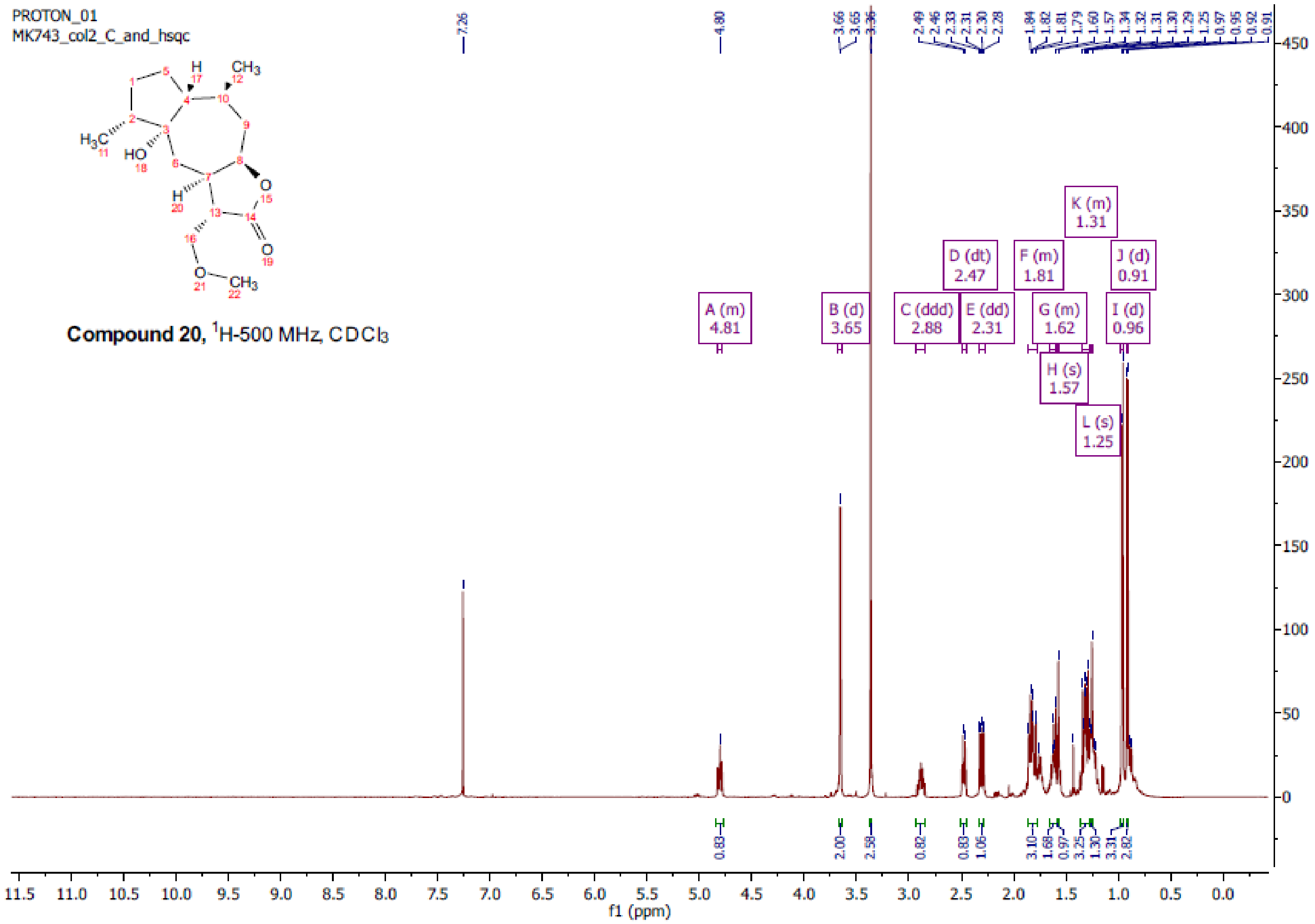

PROTON_01
MK743_col2_C_and_hsqc
Compound 20, 1H-500 MHz, CDCl3
A (m) 4.81
B (d) 3.65
C (ddd) 2.88
D (dt) 2.47
E (dd) 2.31
F (m) 1.81
G (m) 1.62
H (s) 1.57
K (m) 1.31
L (s) 1.25
I (d) 0.96
J (d) 0.91
f1 (ppm)

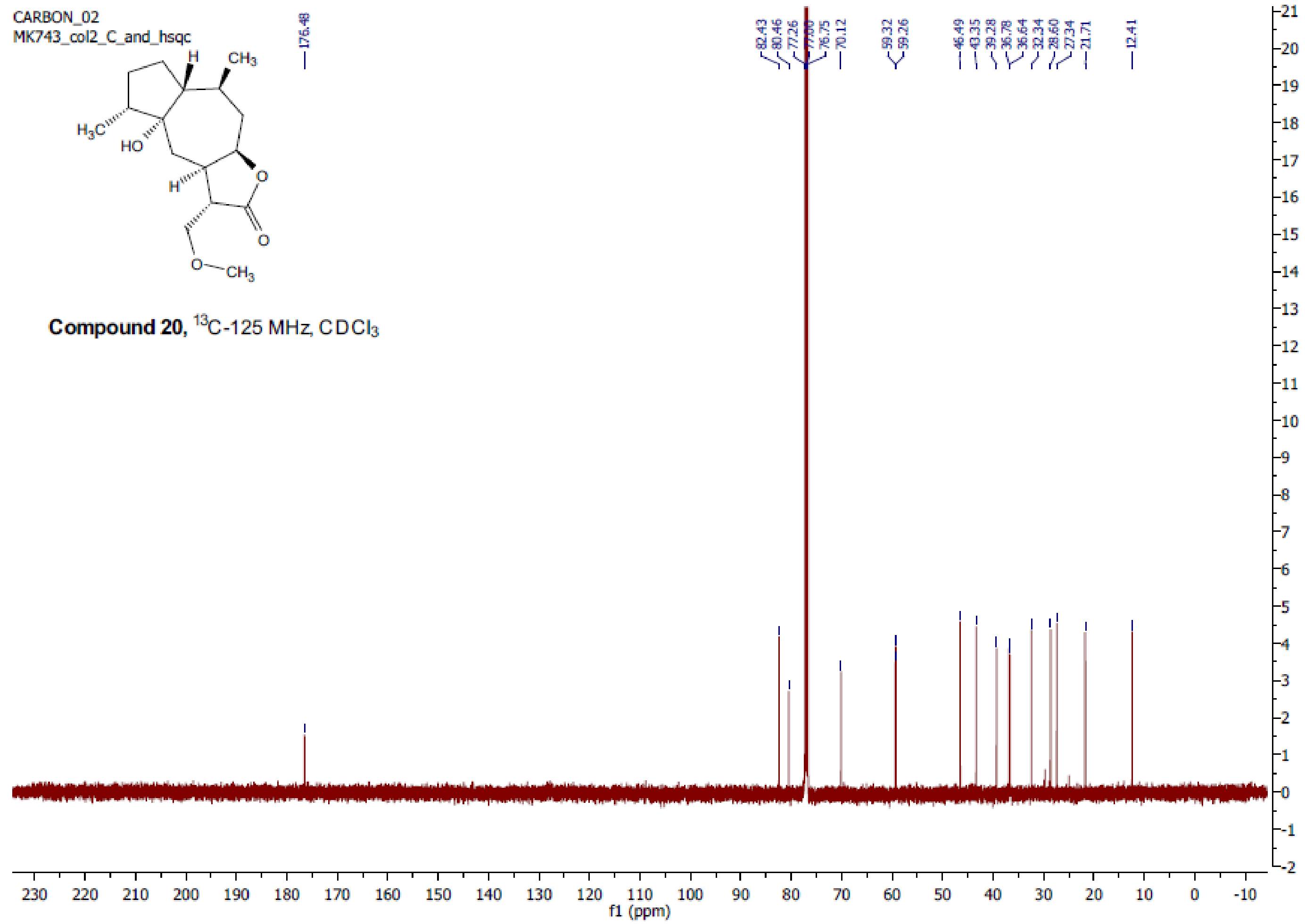

Compound 20, ^{13}C-125 MHz, $CDCl_3$

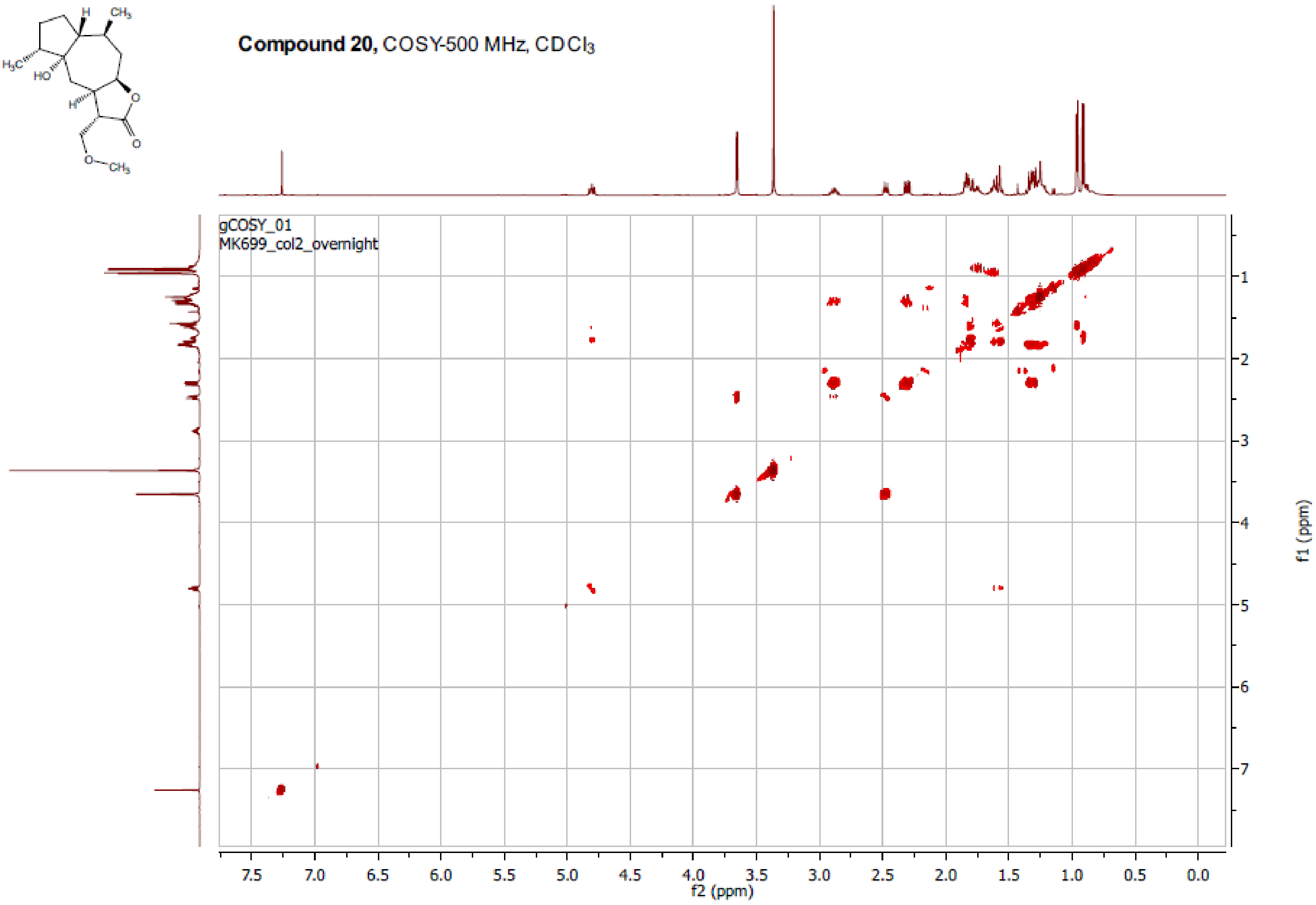

Compound 20, COSY-500 MHz, $CDCl_3$

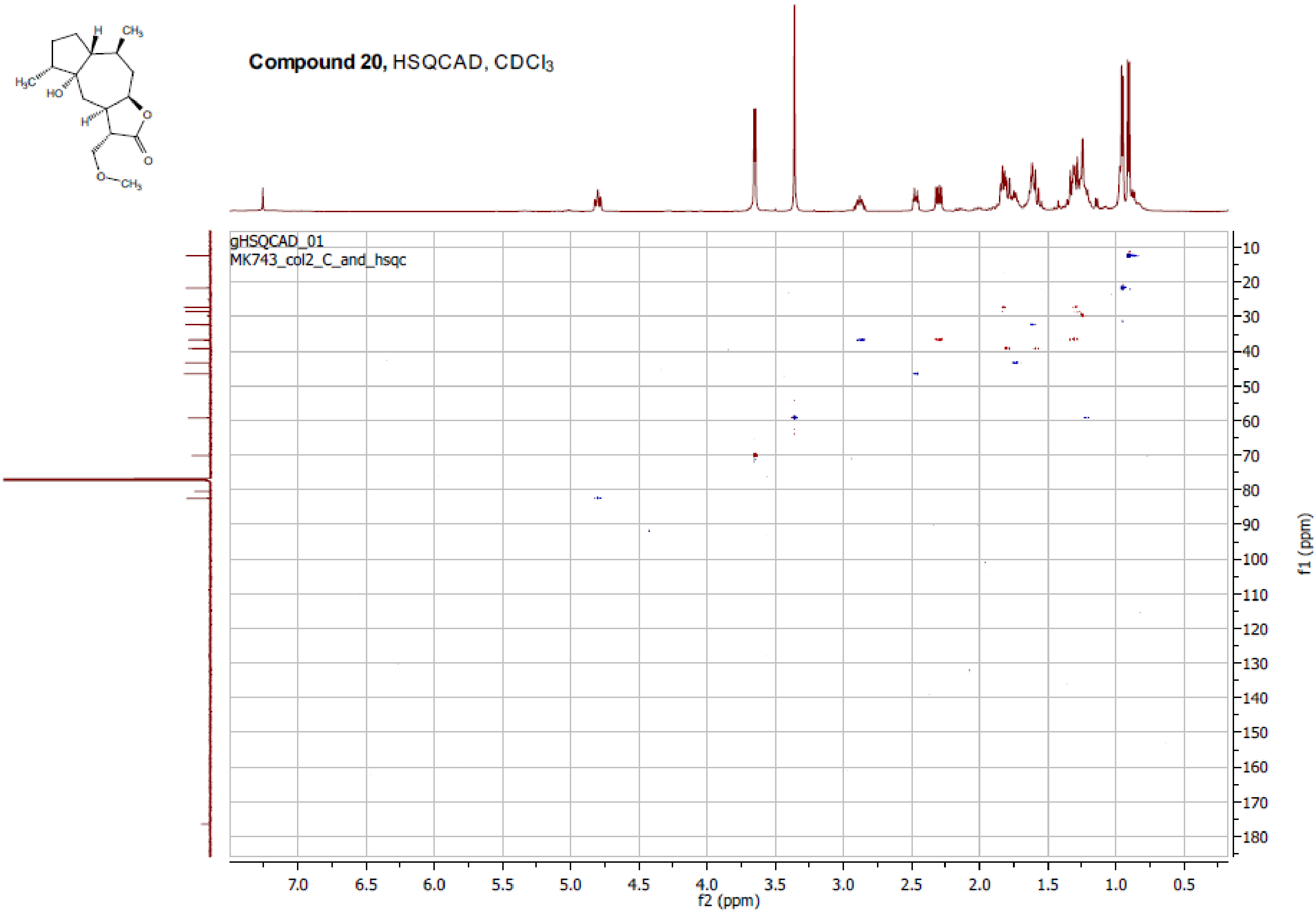
Compound 20, HSQCAD, CDCl3
gHSQCAD_01
MK743_col2_C_and_hsqc
f2 (ppm)
f1 (ppm)

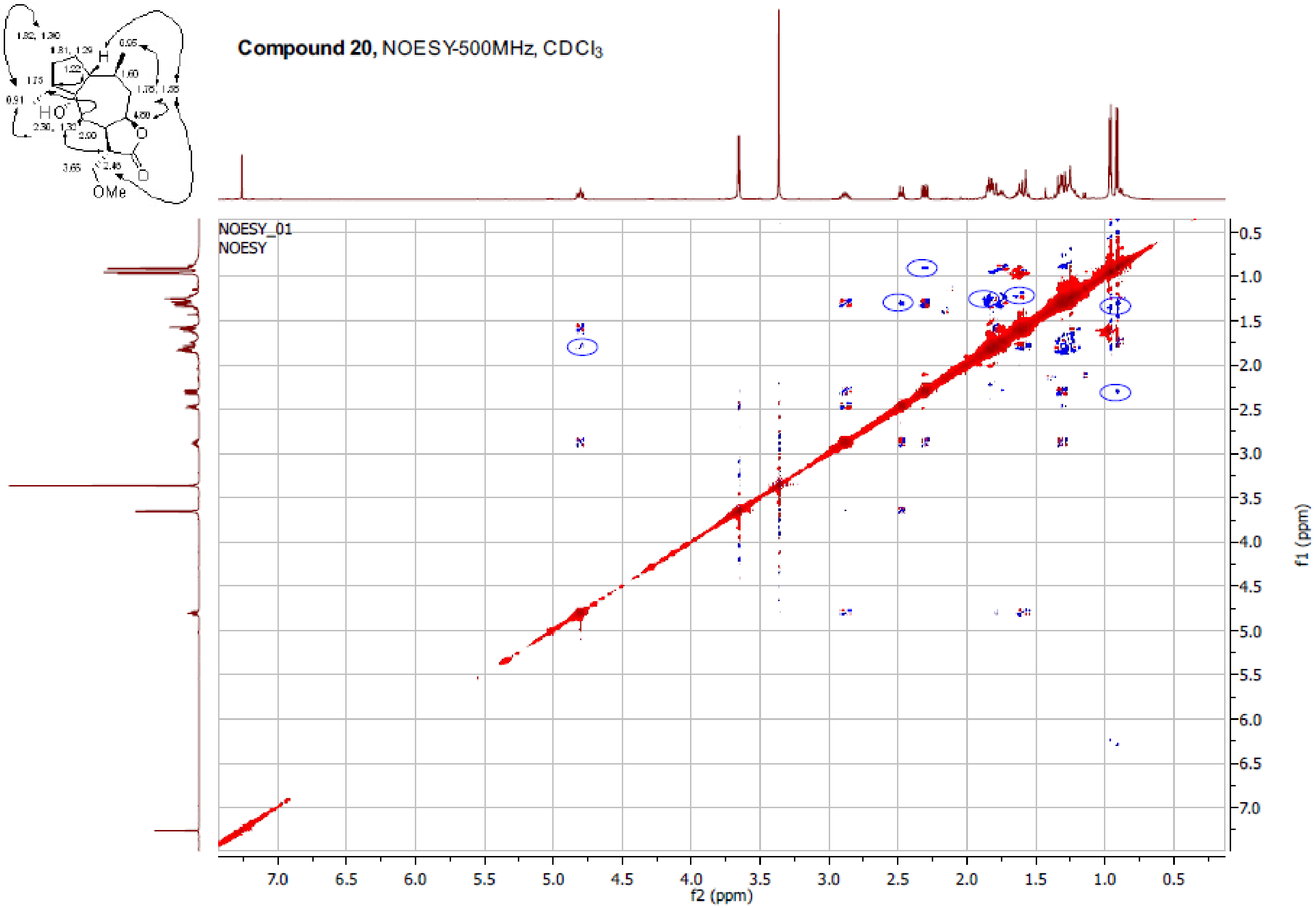
Compound 20, NOESY-500MHz, CDCl3
NOESY_01
NOESY
OMe
f2 (ppm)
f1 (ppm)

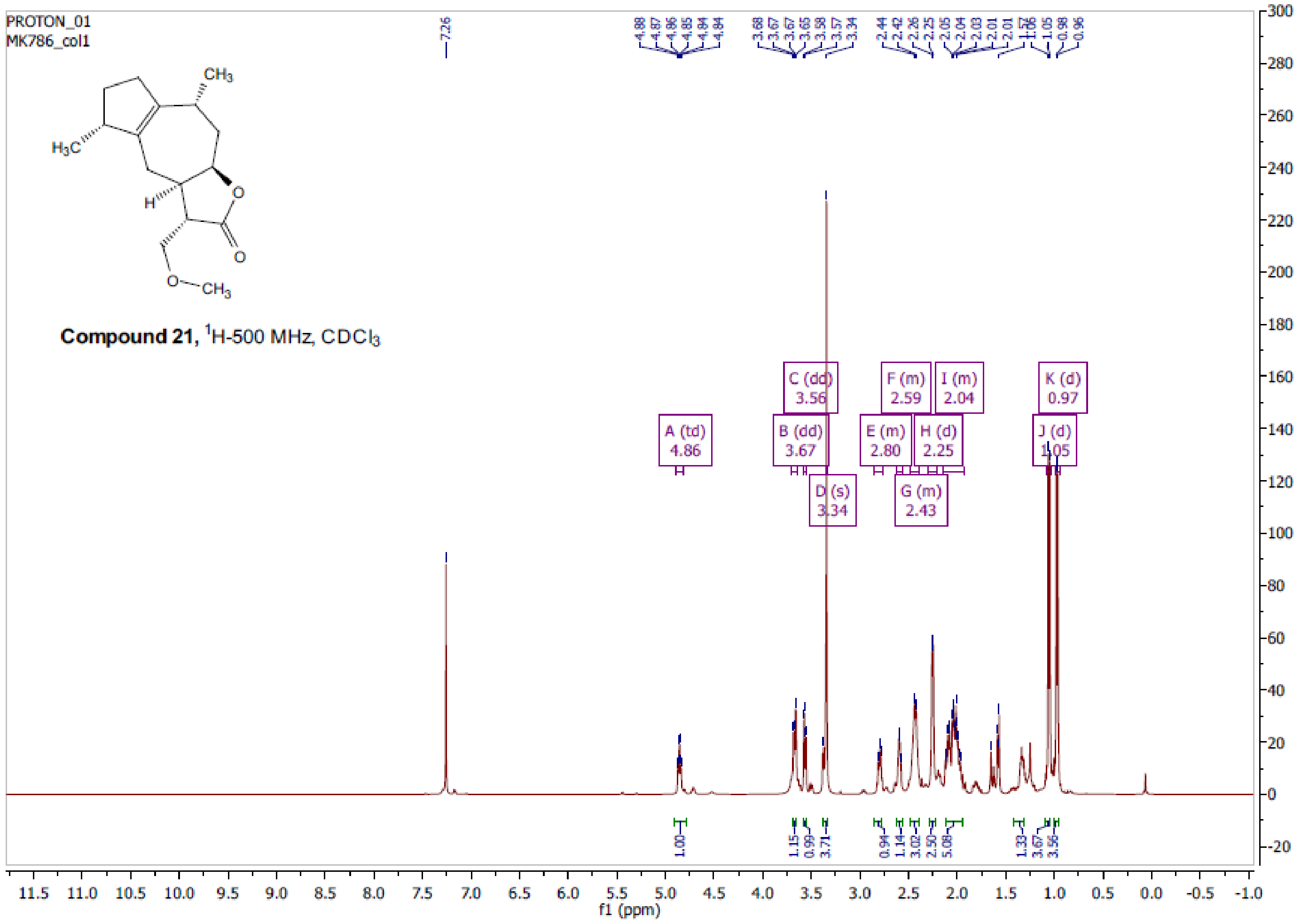
PROTON_01
MK786_col1
Compound 21, ¹H-500 MHz, CDCl3
A (td) 4.86
B (dd) 3.67
C (dd) 3.56
D (s) 3.34
E (m) 2.80
F (m) 2.59
G (m) 2.43
H (d) 2.25
I (m) 2.04
J (d) 1.05
K (d) 0.97
f1 (ppm)

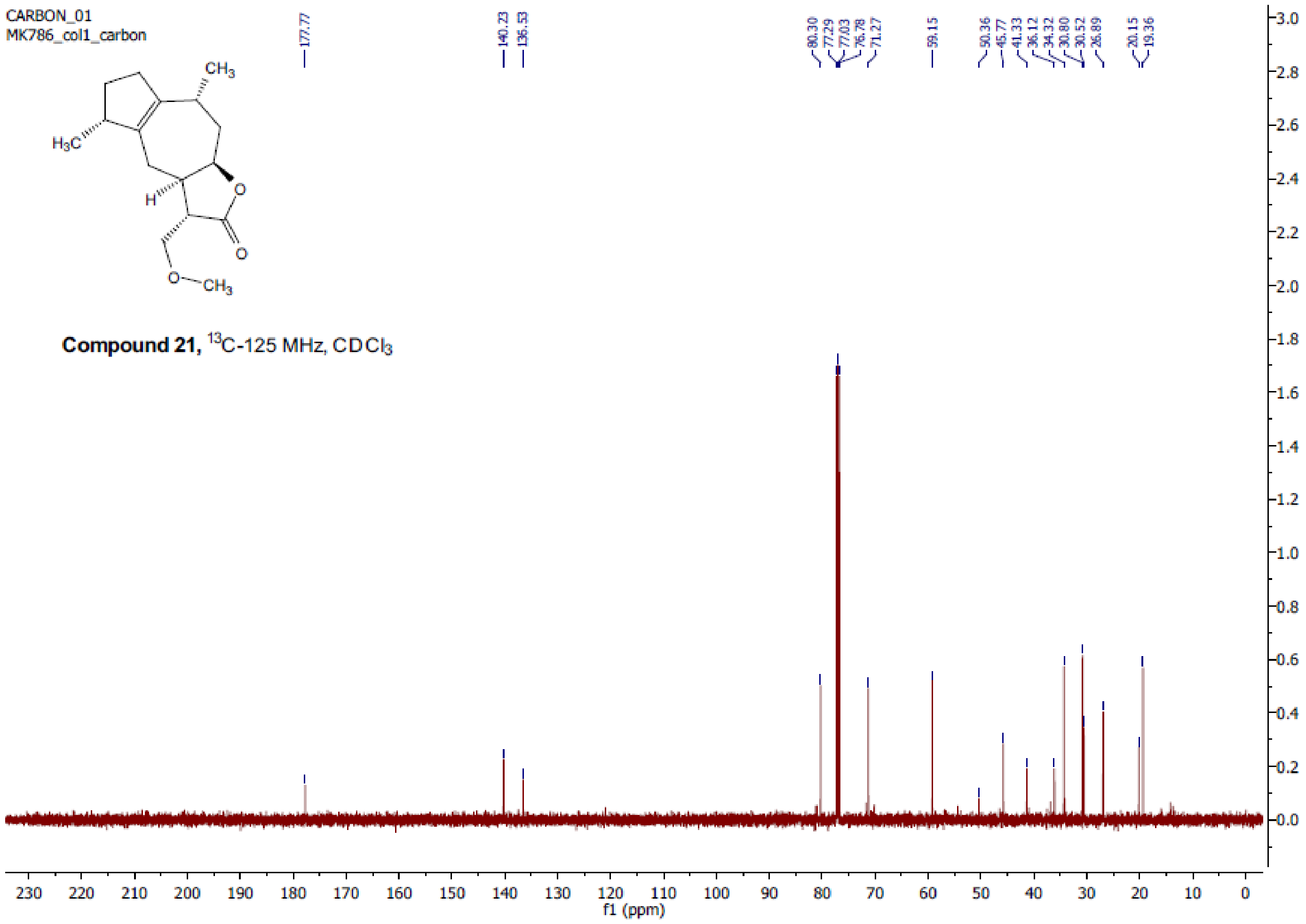
CARBON_01
MK786_col1_carbon
Compound 21, ¹³C-125 MHz, CDCl3
f1 (ppm)

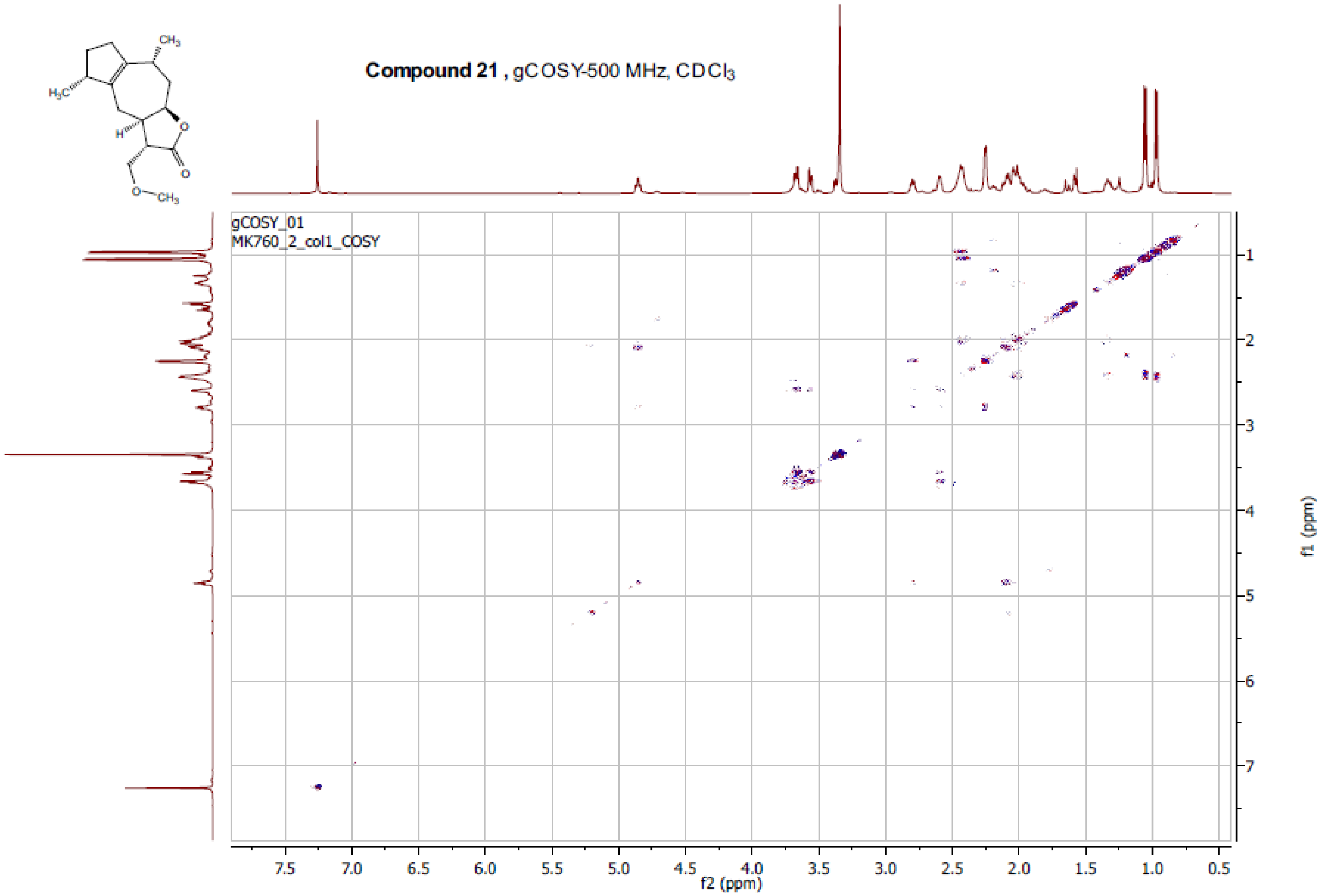

Compound 21, gCOSY-500 MHz, $CDCl_3$

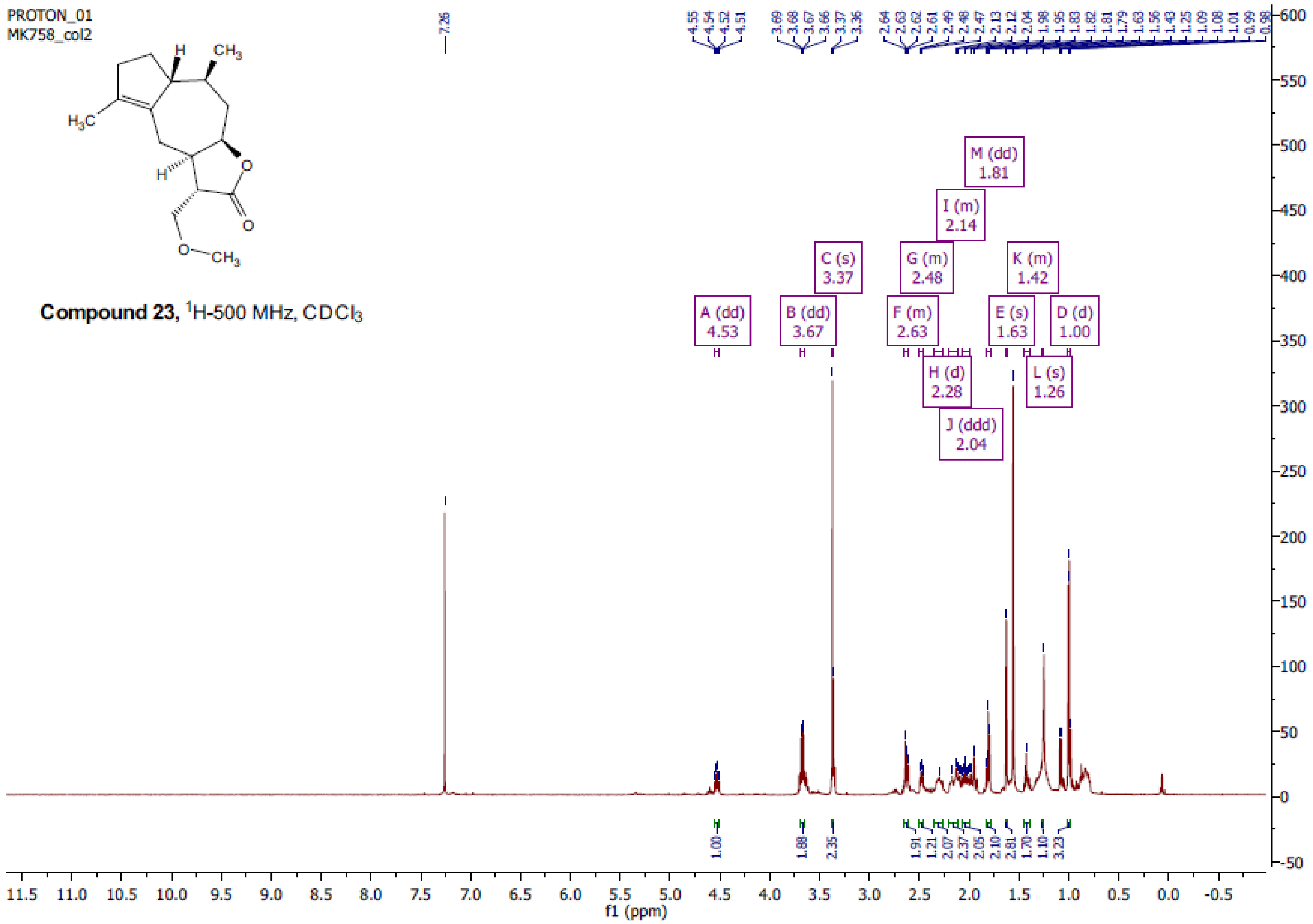

Compound 23, ^1H-500 MHz, $CDCl_3$

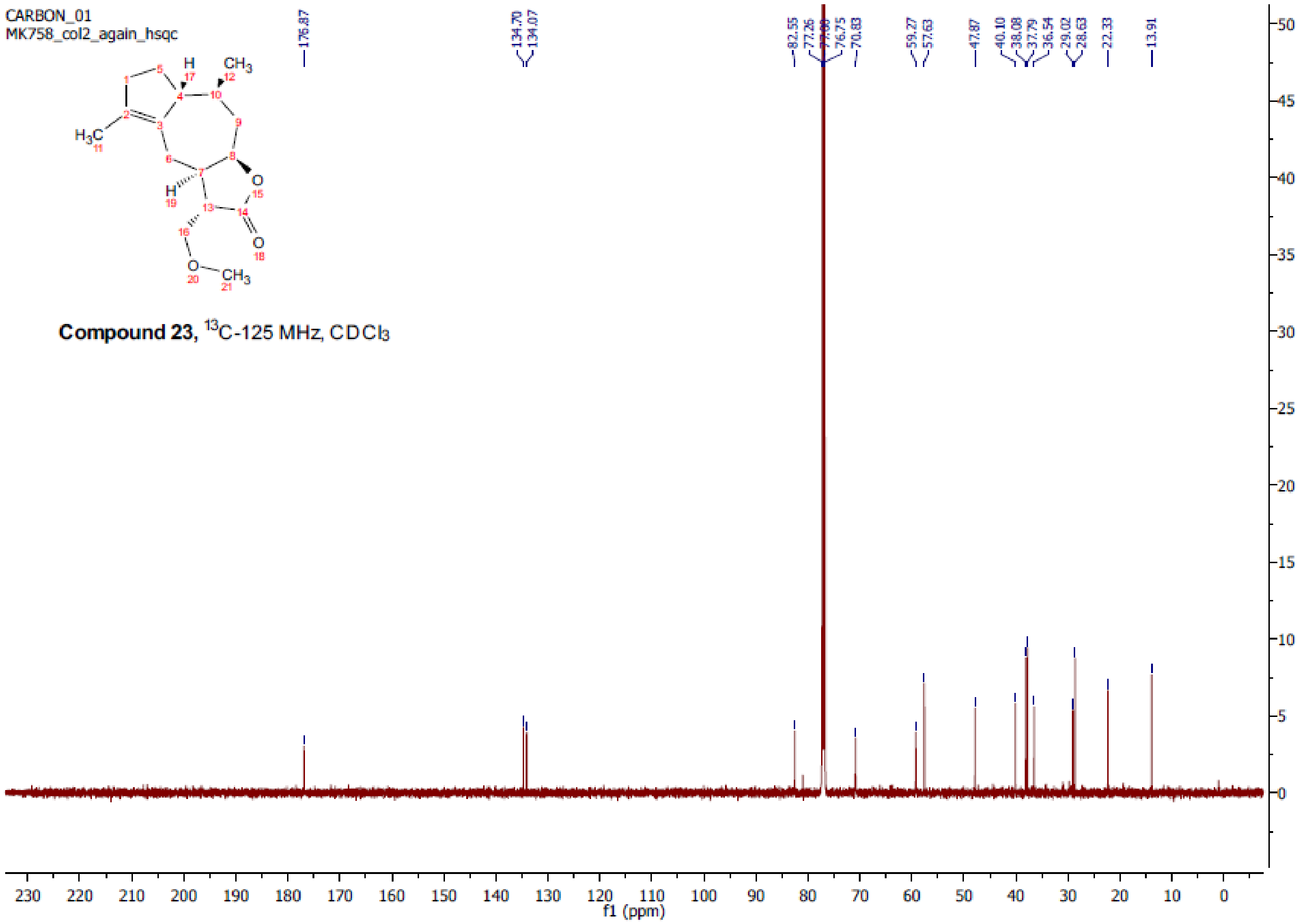

Compound 23, ^{13}C-125 MHz, $CDCl_3$

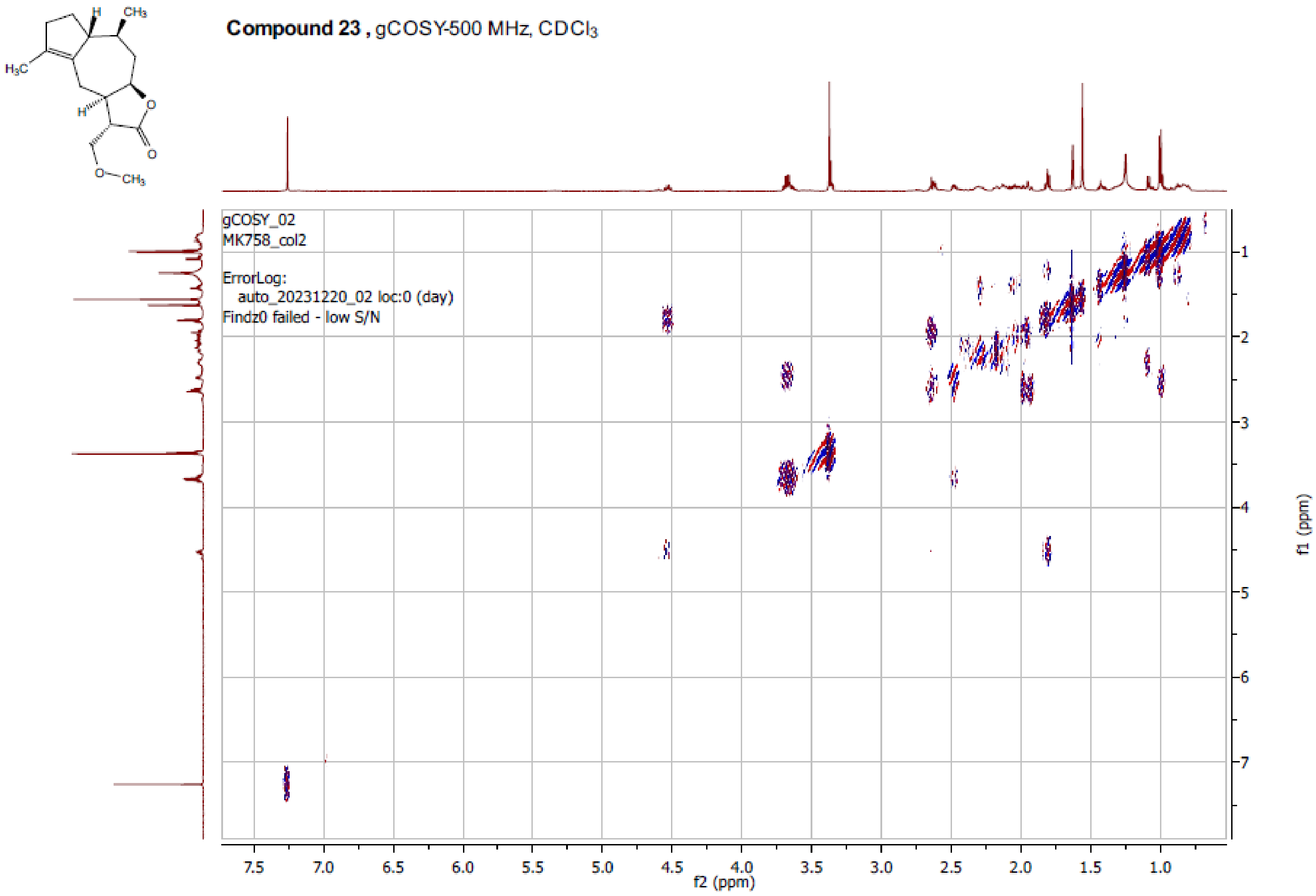

Compound 23 , gCOSY-500 MHz, $CDCl_3$

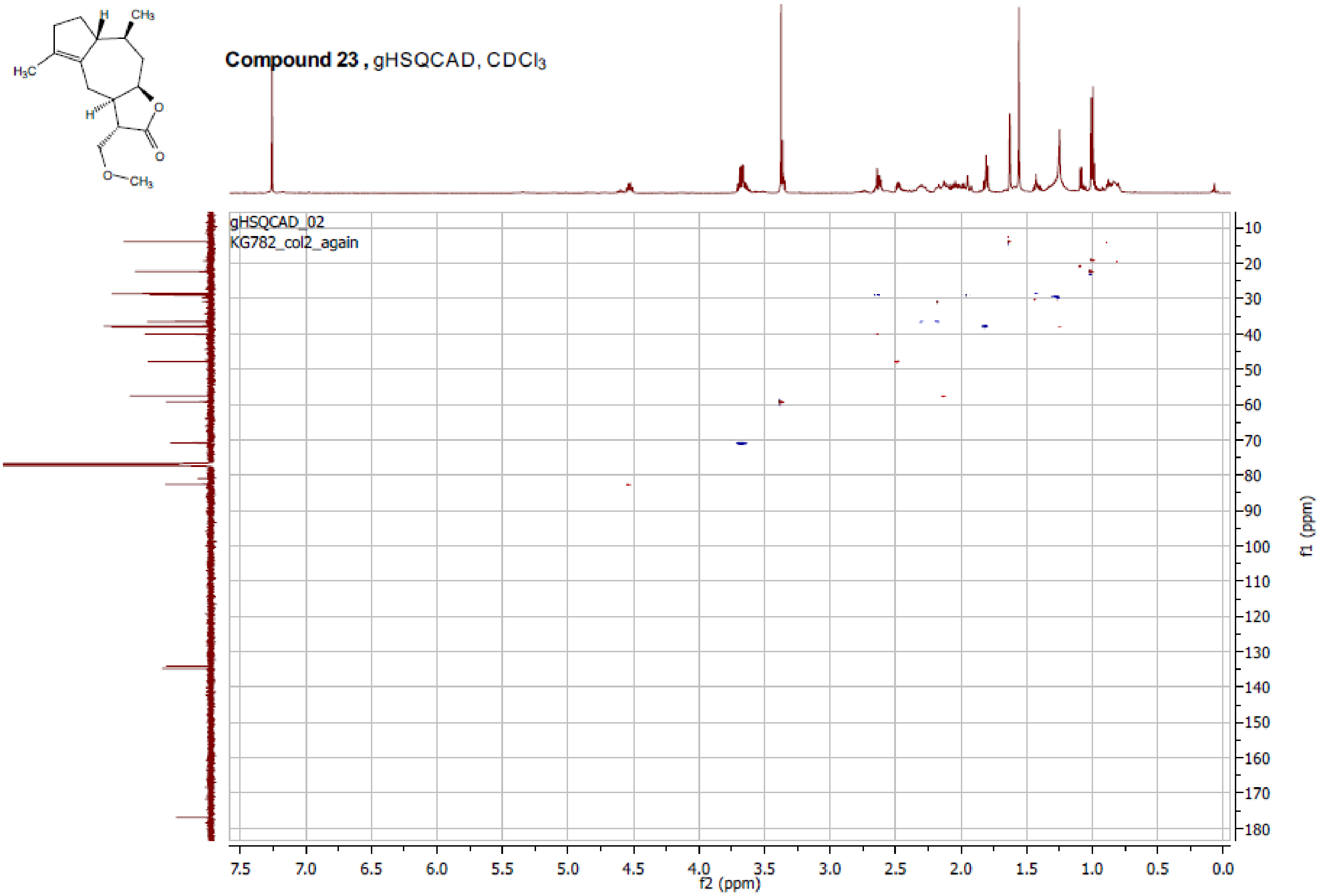
Compound 23 , gHSQCAD, CDCl3
gHSQCAD_02
KG782_col2_again
f2 (ppm)
f1 (ppm)

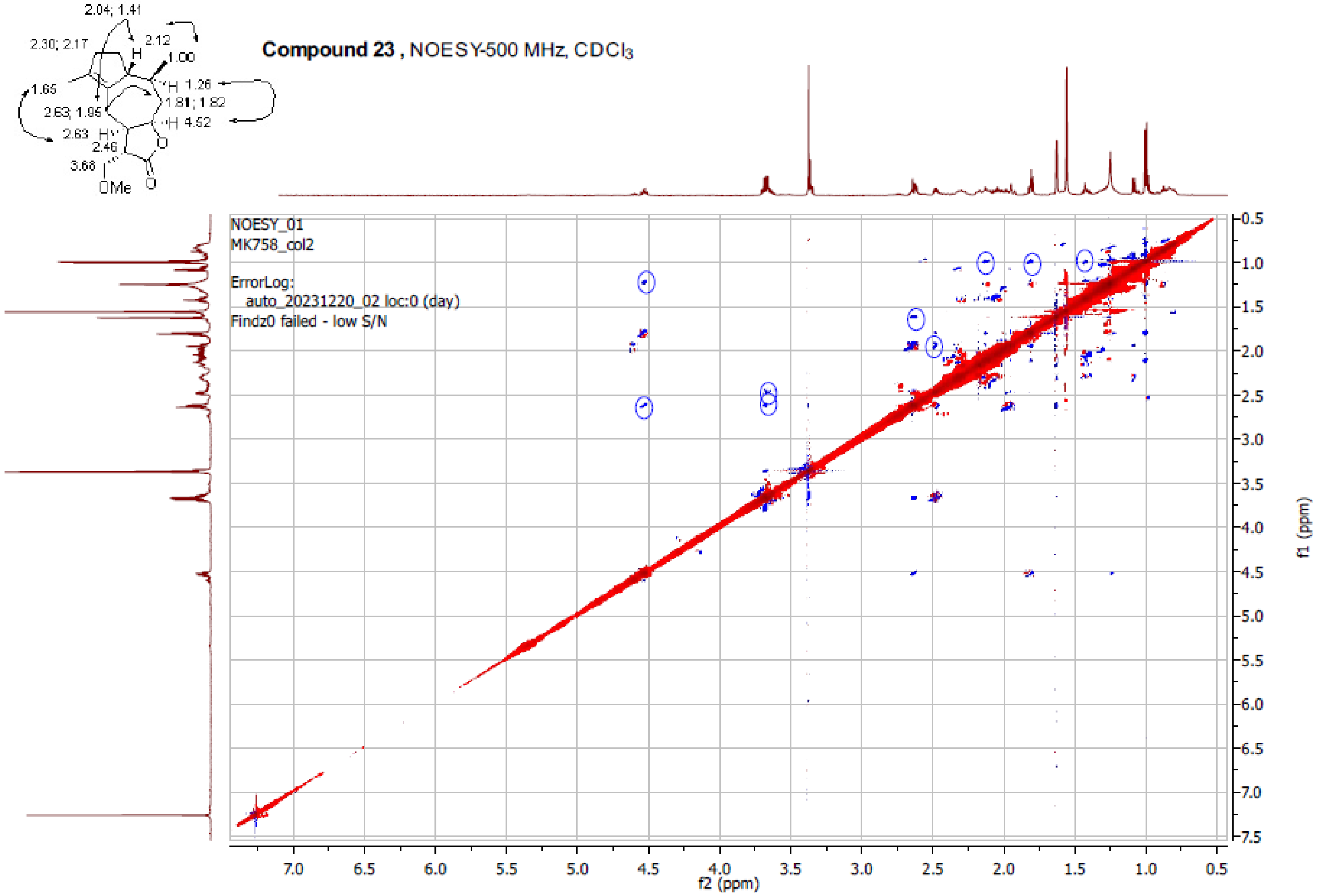
Compound 23 , NOESY-500 MHz, CDCl3
NOESY_01
MK758_col2
ErrorLog:
auto_20231220_02 loc:0 (day)
Findz0 failed - low S/N
f2 (ppm)
f1 (ppm)
OMe

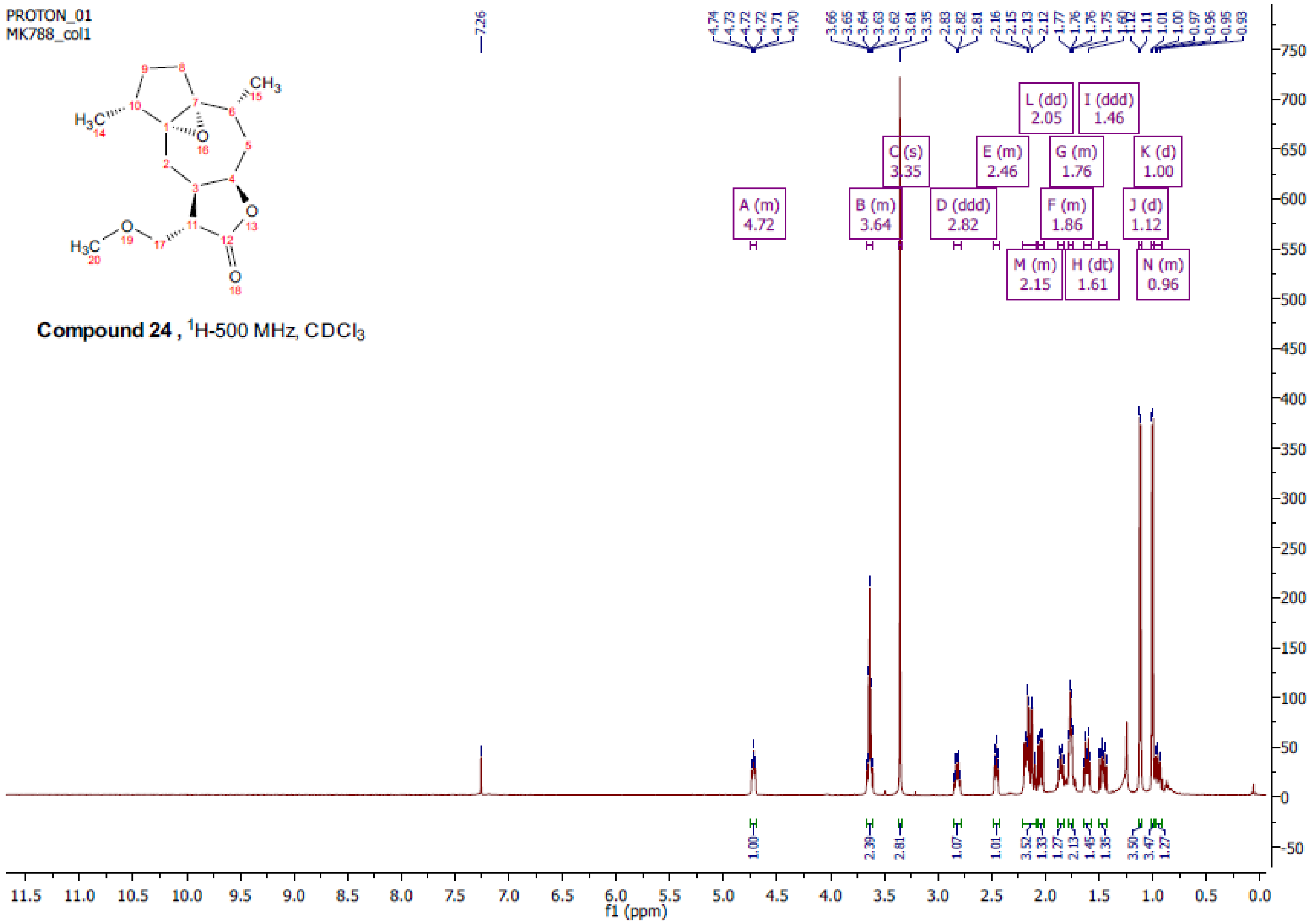
PROTON_01
MK788_col1
Compound 24 , ¹H-500 MHz, CDCl3
A (m) 4.72
B (m) 3.64
C (s) 3.35
D (ddd) 2.82
E (m) 2.46
F (m) 1.86
G (m) 1.76
H (dt) 1.61
I (ddd) 1.46
J (d) 1.12
K (d) 1.00
L (dd) 2.05
M (m) 2.15
N (m) 0.96
f1 (ppm)

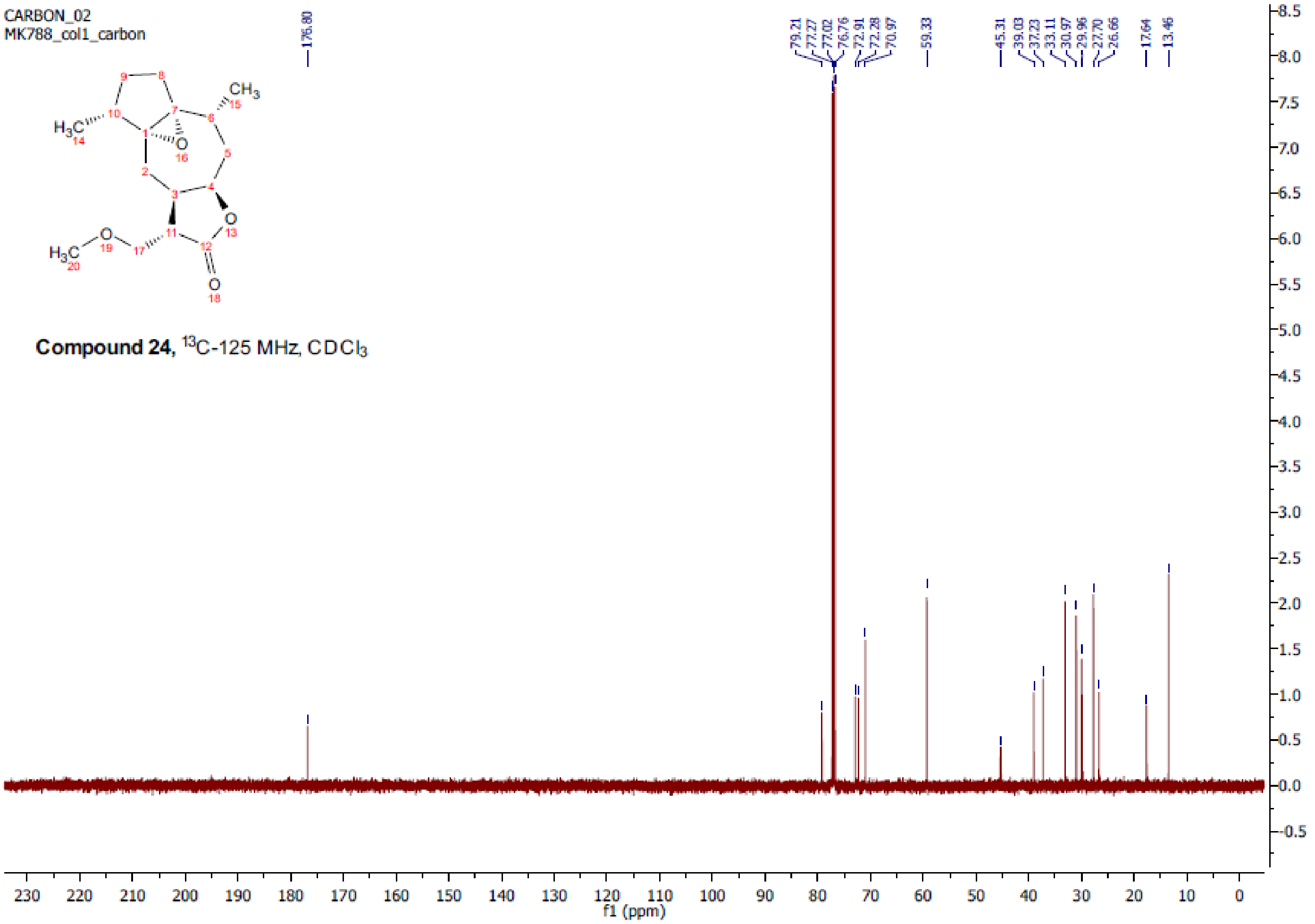
CARBON_02
MK788_col1_carbon
Compound 24, ¹³C-125 MHz, CDCl3
176.80
79.21
77.27
77.02
76.76
72.91
72.28
70.97
59.33
45.31
39.03
37.23
33.11
30.97
29.96
27.70
26.66
17.64
13.46
f1 (ppm)

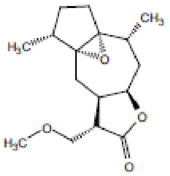

Compound 24 , gCOSY-500 MHz, CDCl3

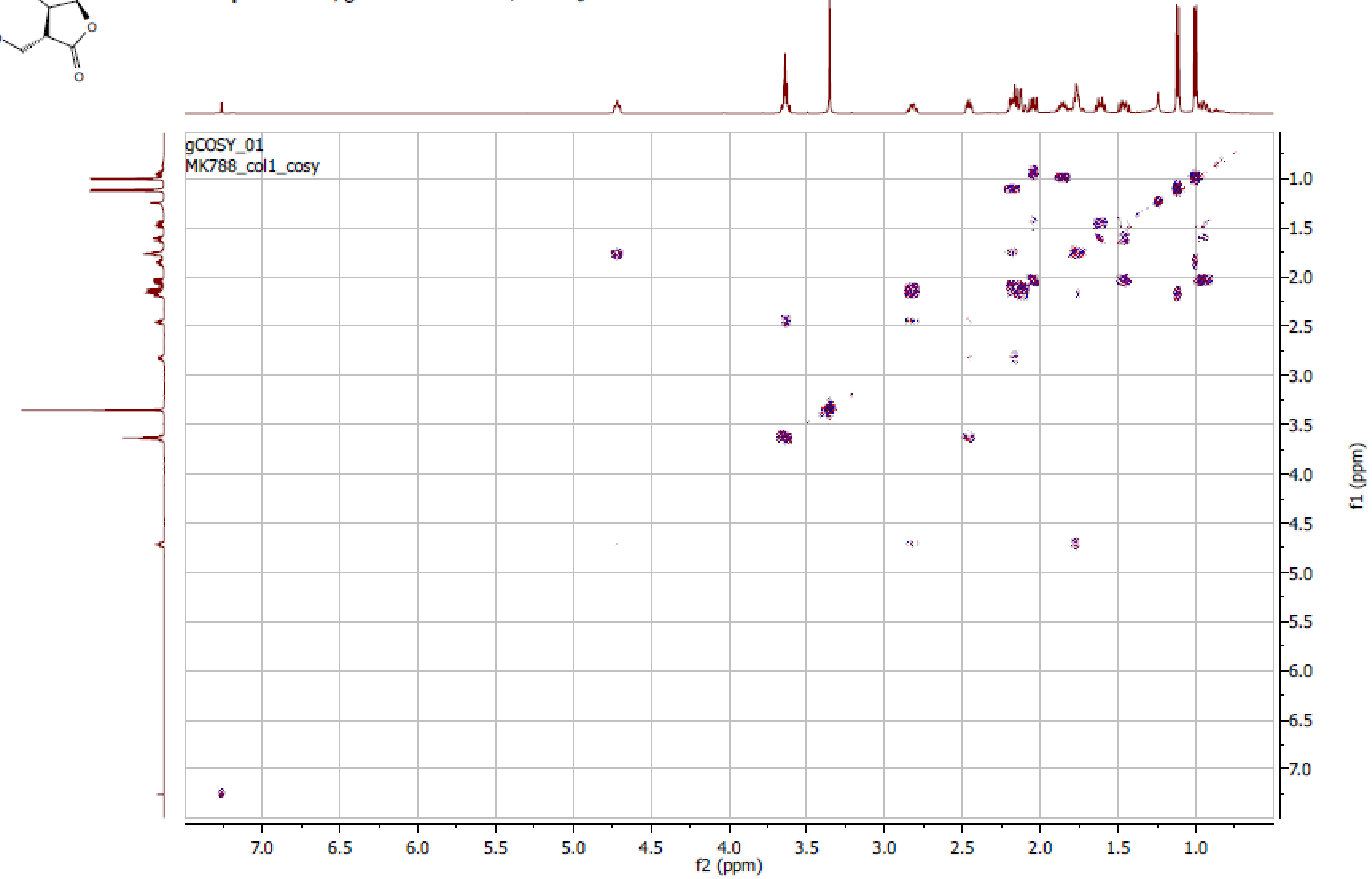

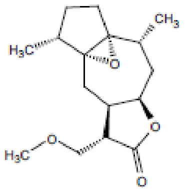

Compound 24 , gHSQCAD, CDCl3

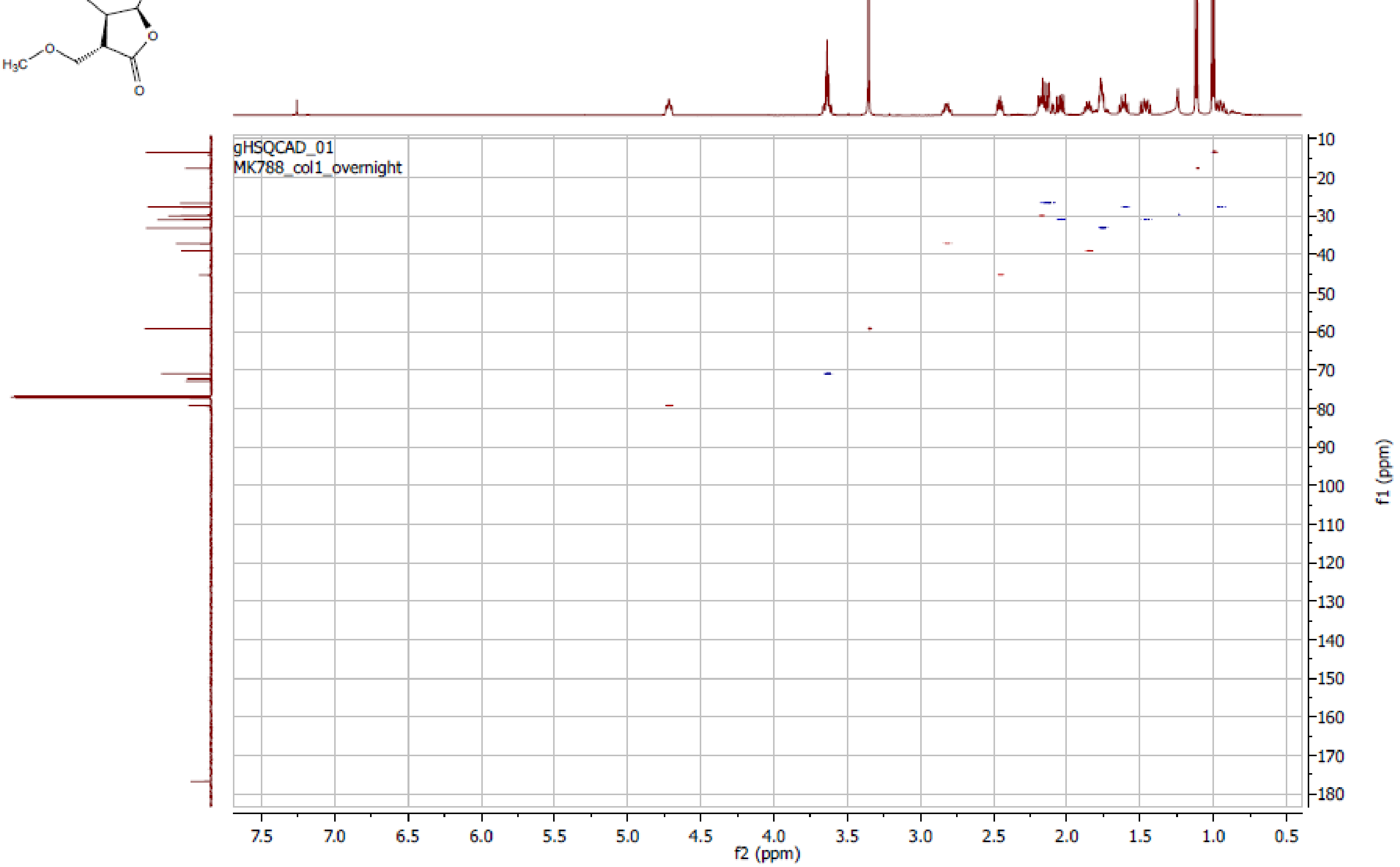

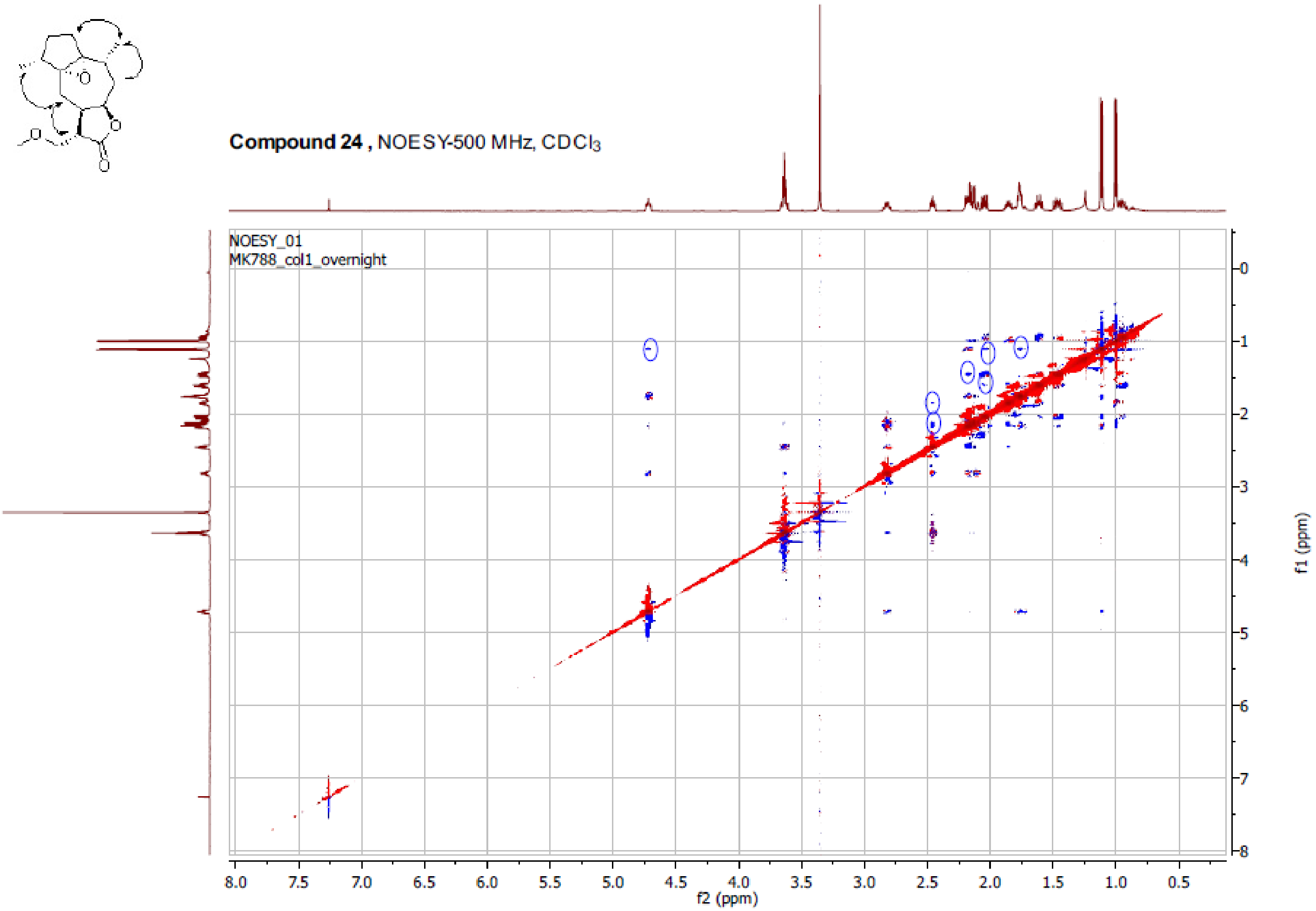
Compound 24 , NOESY-500 MHz, CDCl3
NOESY_01
MK788_col1_overnight
f2 (ppm)
f1 (ppm)

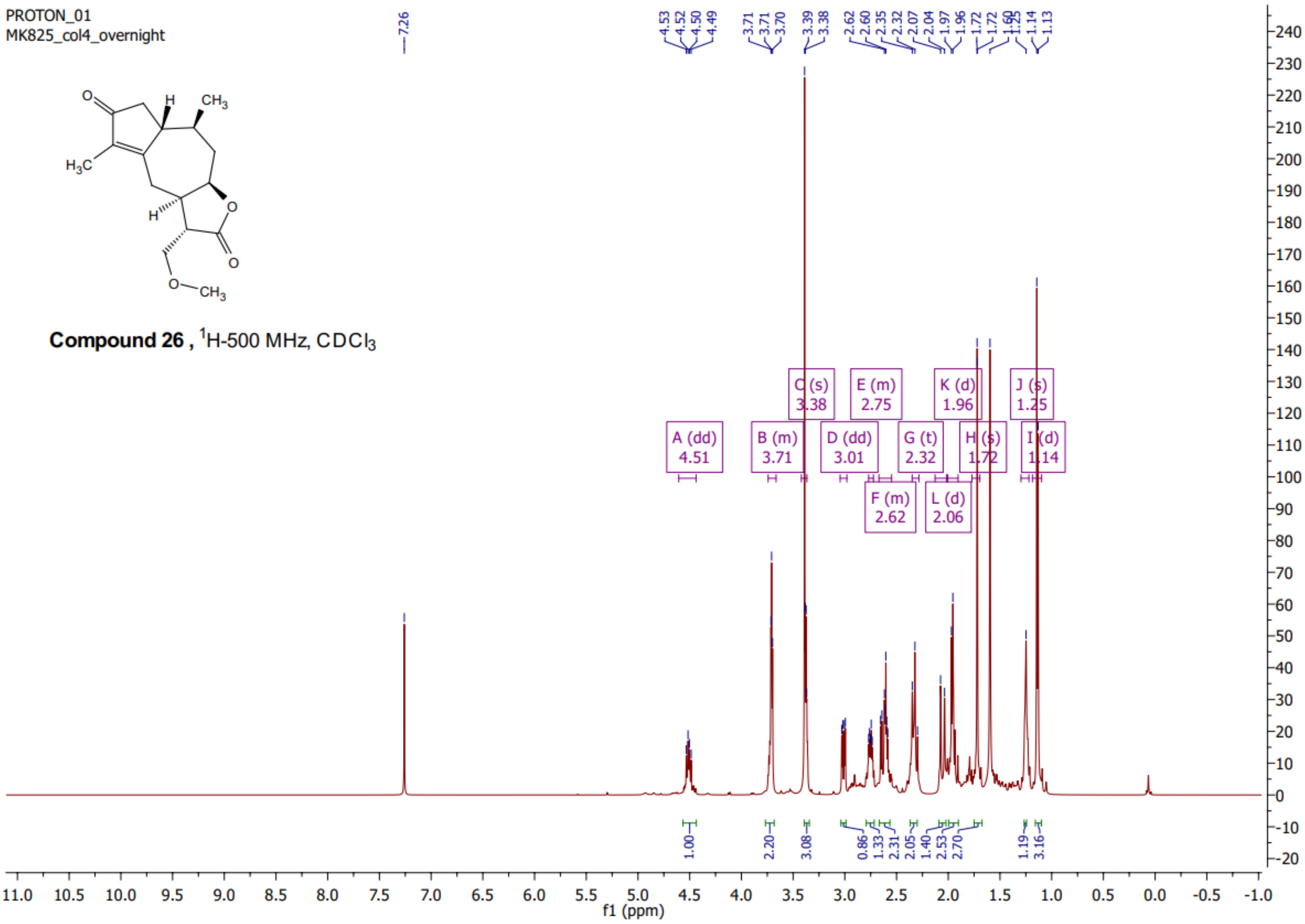
PROTON_01
MK825_col4_overnight
Compound 26 , 1H-500 MHz, CDCl3
7.26
4.53 4.52 4.50 4.49
3.71 3.71 3.70
3.39 3.38
2.62 2.60 2.35 2.32 2.07 2.04 1.97 1.96 1.72 1.72 1.60 1.25 1.14 1.13
A (dd) 4.51
B (m) 3.71
C (s) 3.38
D (dd) 3.01
E (m) 2.75
F (m) 2.62
G (t) 2.32
L (d) 2.06
K (d) 1.96
H (s) 1.72
J (s) 1.25
I (d) 1.14
1.00
2.20
3.08
0.86
1.33
2.31
2.05
1.40
2.53
2.70
1.19
3.16
f1 (ppm)

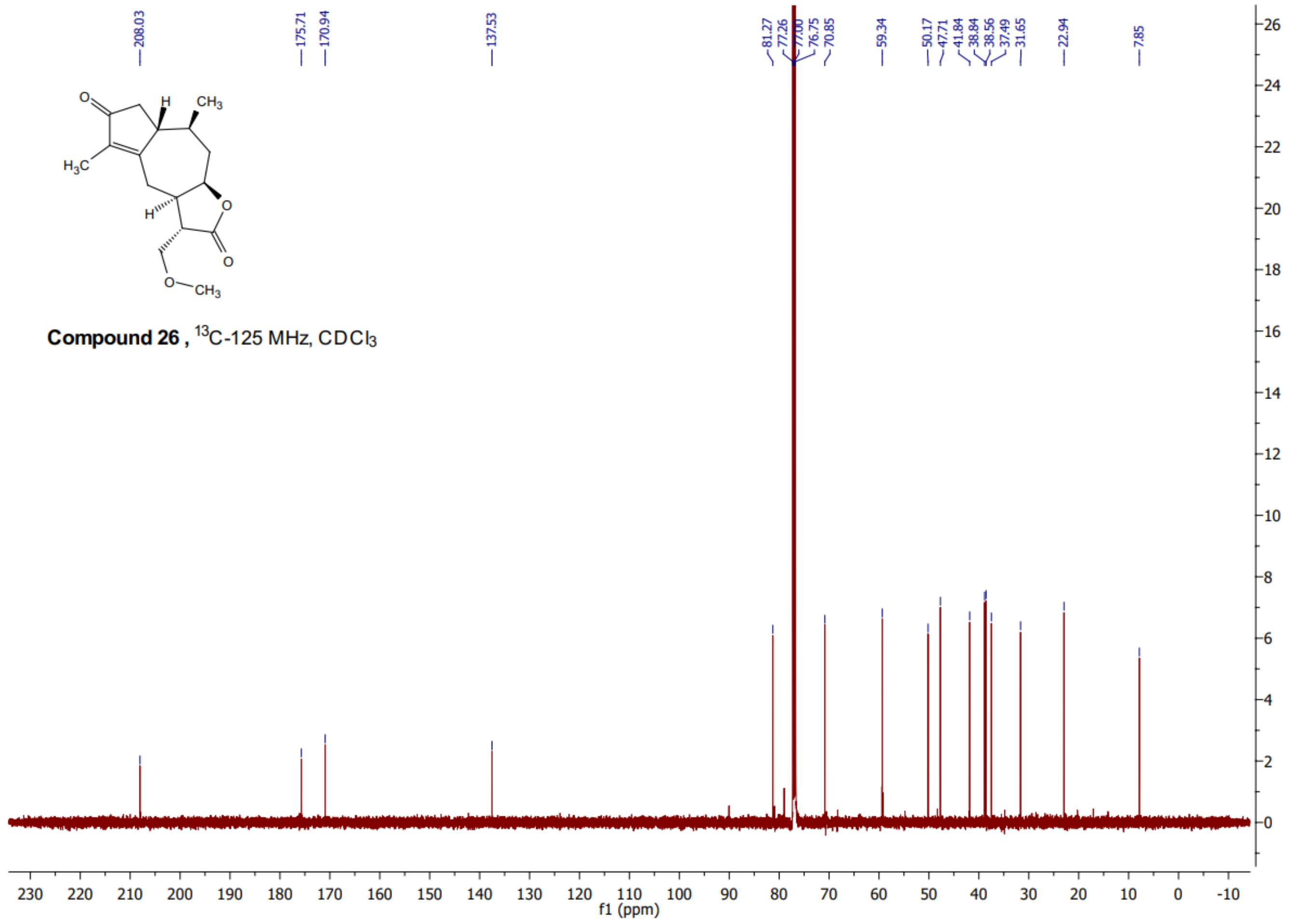

Compound 26 , ^{13}C-125 MHz, CDCl$_3$
208.03
175.71
170.94
137.53
81.27
77.26
77.00
76.75
70.85
59.34
50.17
47.71
41.84
38.84
38.56
37.49
31.65
22.94
7.85
f1 (ppm)

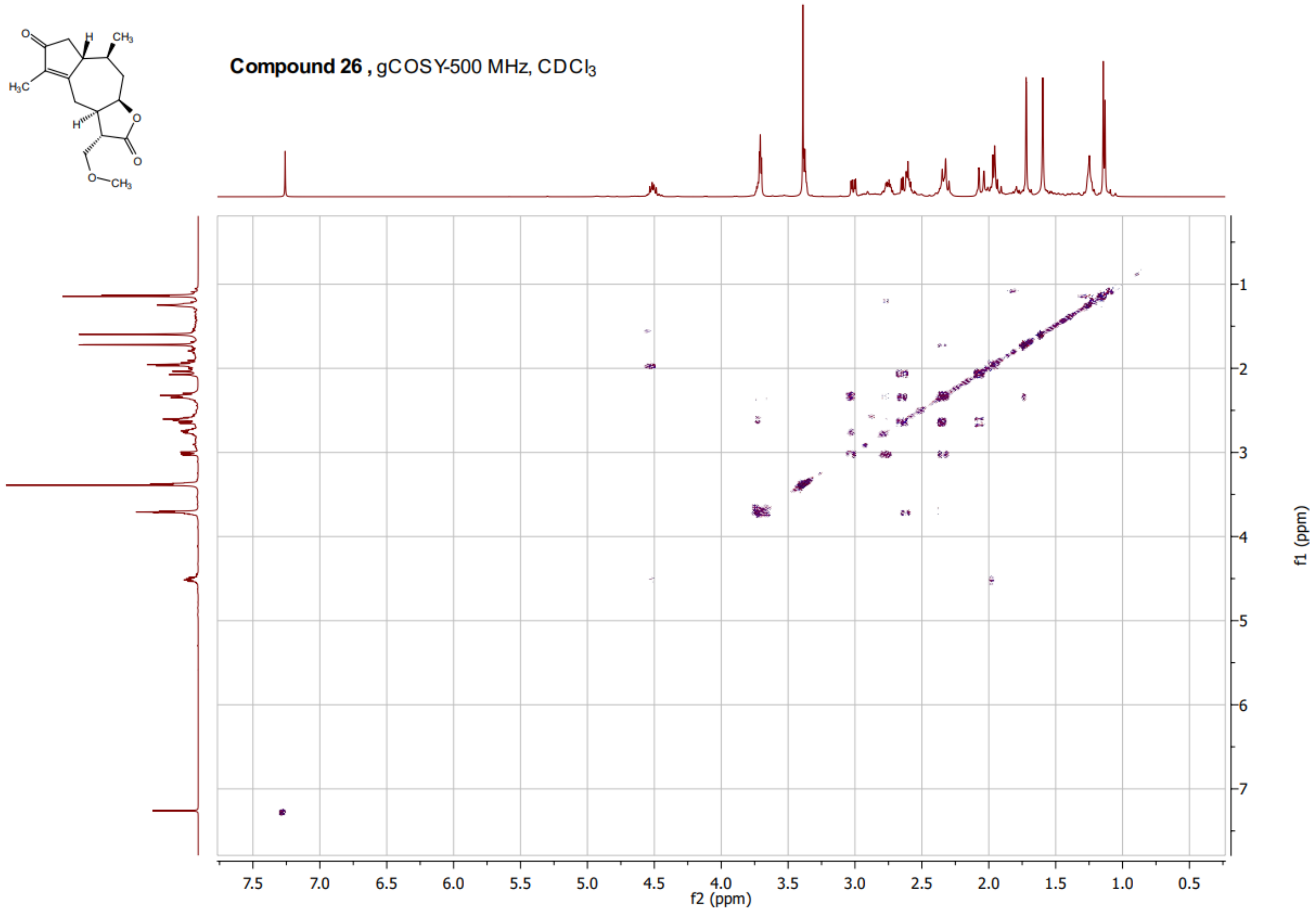

Compound 26 , gCOSY-500 MHz, CDCl$_3$
f2 (ppm)
f1 (ppm)

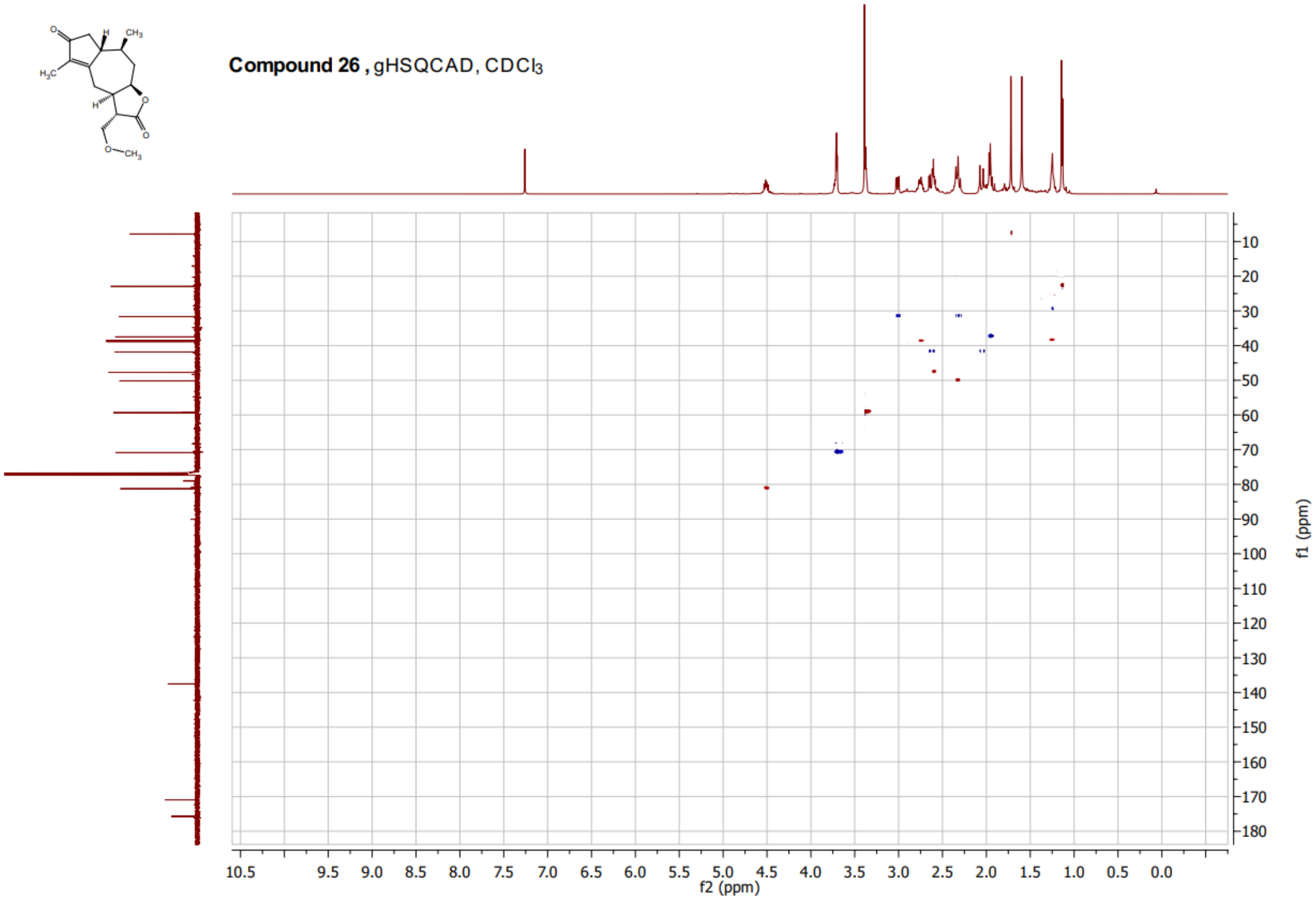
Compound 26 , gHSQCAD, CDCl3
f2 (ppm)
f1 (ppm)

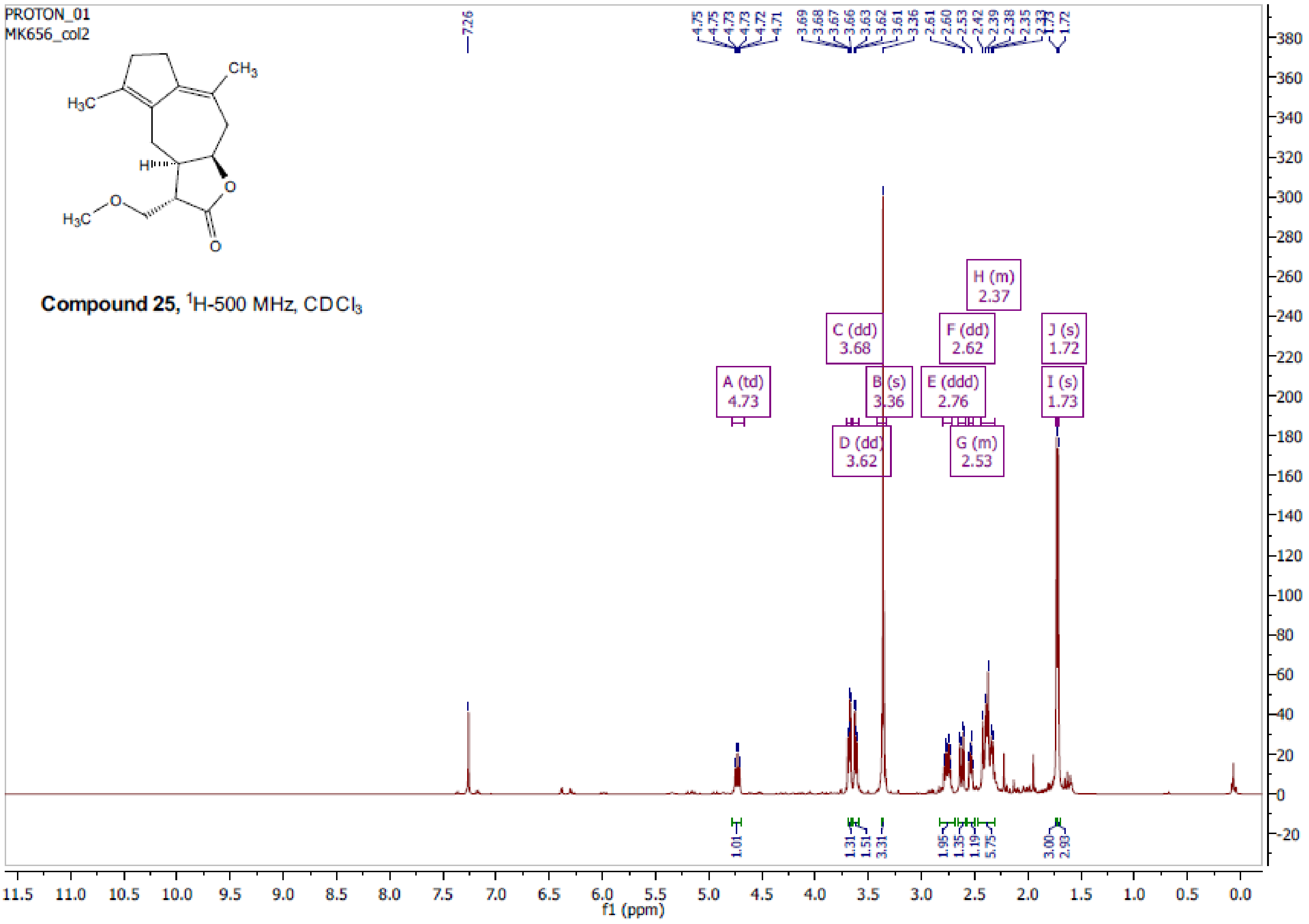
PROTON_01
MK656_col2
Compound 25, 1H-500 MHz, CDCl3
A (td) 4.73
B (s) 3.36
C (dd) 3.68
D (dd) 3.62
E (ddd) 2.76
F (dd) 2.62
G (m) 2.53
H (m) 2.37
I (s) 1.73
J (s) 1.72
f1 (ppm)

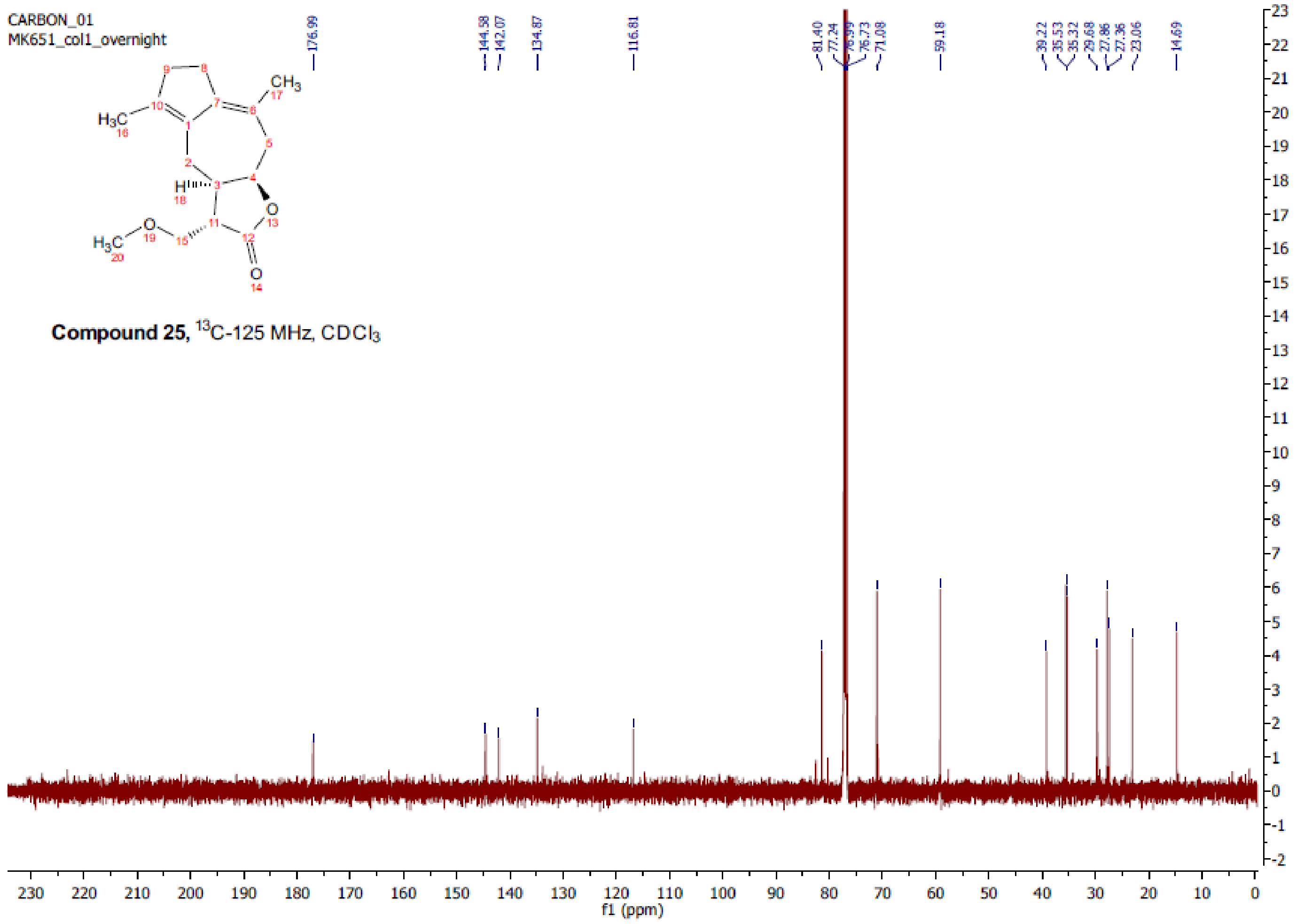
CARBON_01
MK651_col1_overnight
Compound 25, 13C-125 MHz, CDCl3
176.99
144.58
142.07
134.87
116.81
81.40
77.24
76.99
76.73
71.08
59.18
39.22
35.53
35.32
29.68
27.86
27.36
23.06
14.69
f1 (ppm)

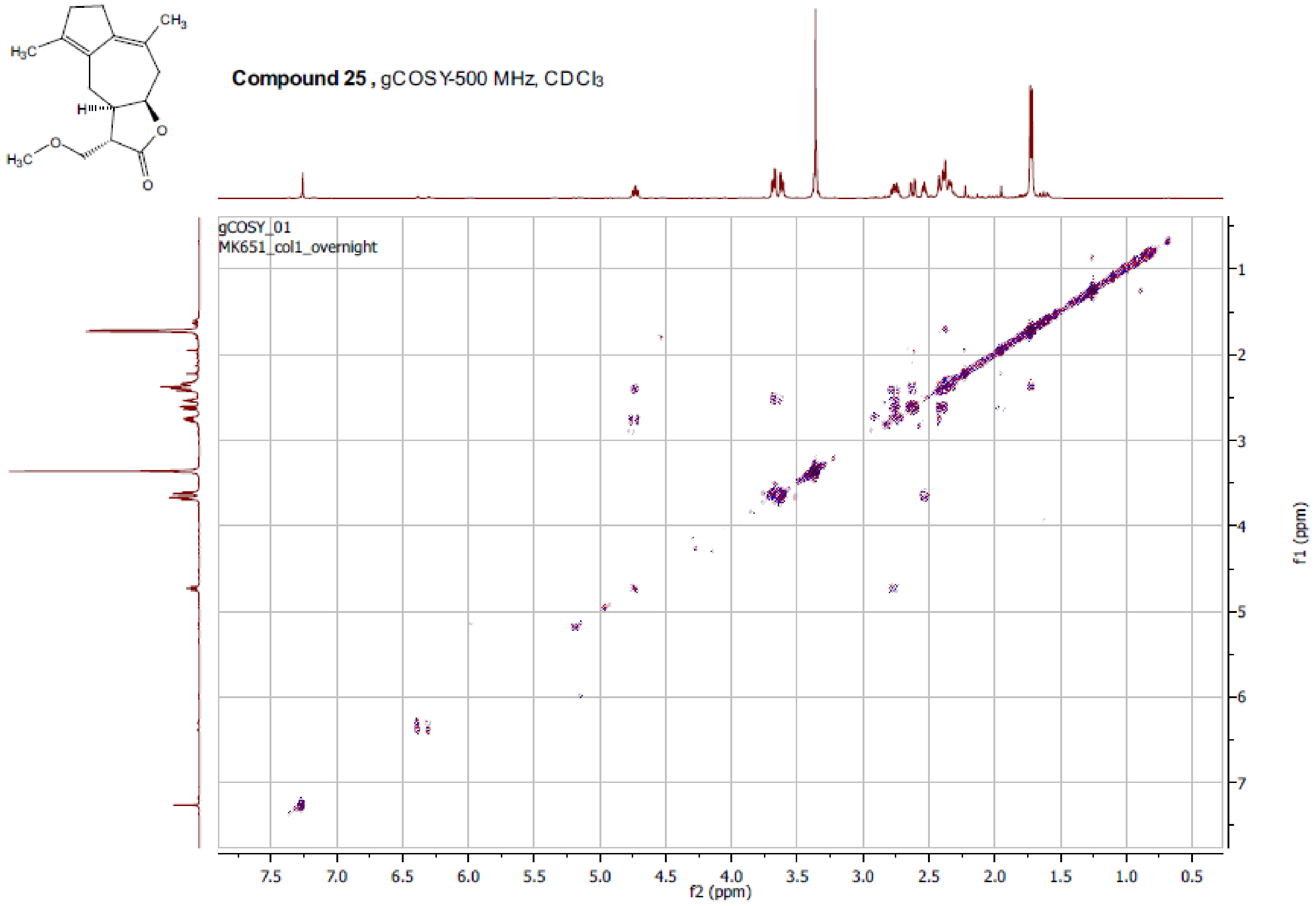
Compound 25 , gCOSY-500 MHz, CDCl3
gCOSY_01
MK651_col1_overnight
f2 (ppm)
f1 (ppm)

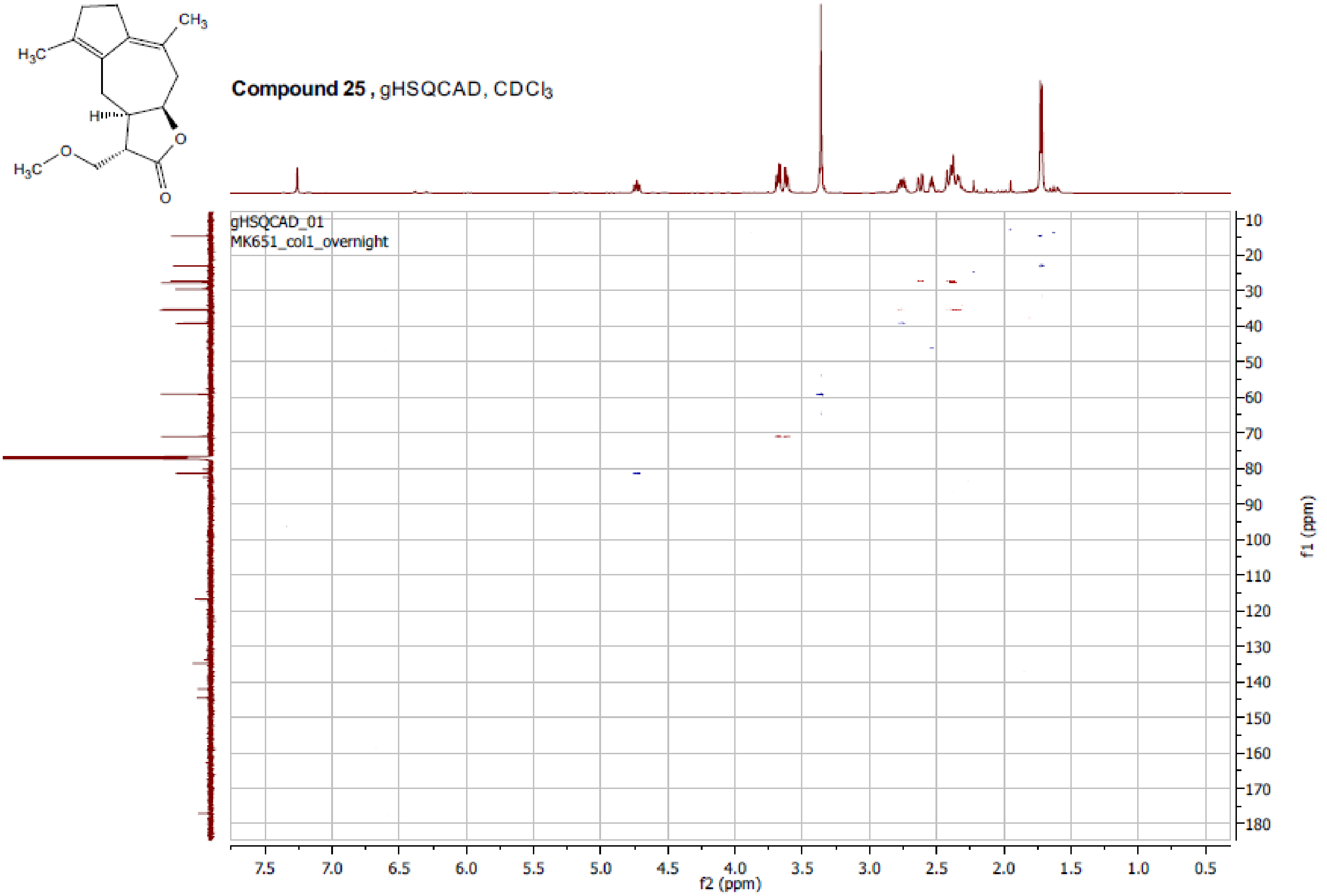
Compound 25 , gHSQCAD, CDCl3
gHSQCAD_01
MK651_col1_overnight
f2 (ppm)
f1 (ppm)

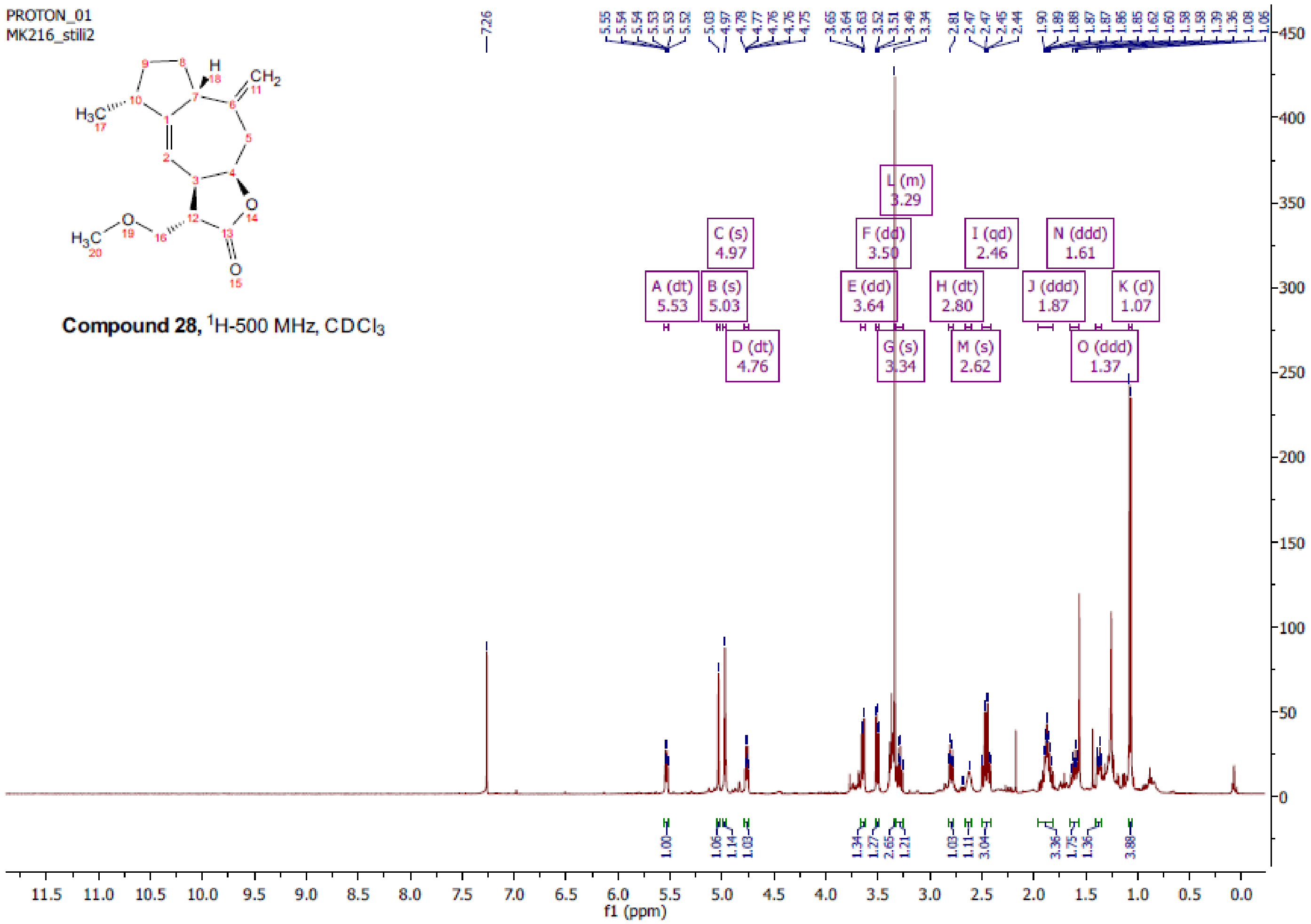
PROTON_01
MK216_stili2
Compound 28, 1H-500 MHz, CDCl3
A (dt) 5.53
B (s) 5.03
C (s) 4.97
D (dt) 4.76
E (dd) 3.64
F (dd) 3.50
G (s) 3.34
L (m) 3.29
H (dt) 2.80
M (s) 2.62
I (qd) 2.46
J (ddd) 1.87
N (ddd) 1.61
O (ddd) 1.37
K (d) 1.07
f1 (ppm)

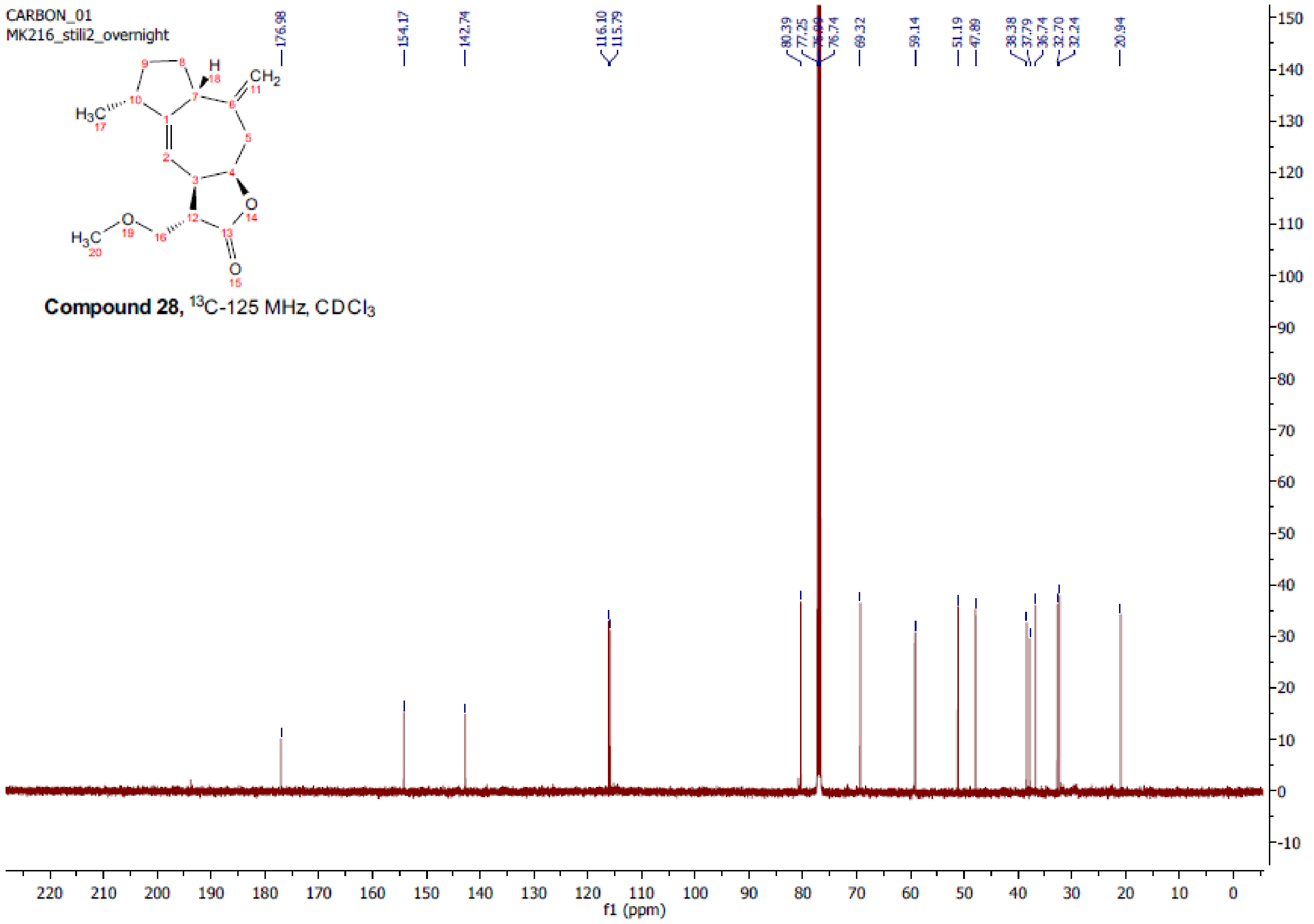
CARBON_01
MK216_stili2_overnight
Compound 28, ^{13}C-125 MHz, $CDCl_3$
176.98
154.17
142.74
116.10
115.79
80.39
77.25
76.74
69.32
59.14
51.19
47.89
38.38
37.79
36.74
32.70
32.24
20.94
f1 (ppm)

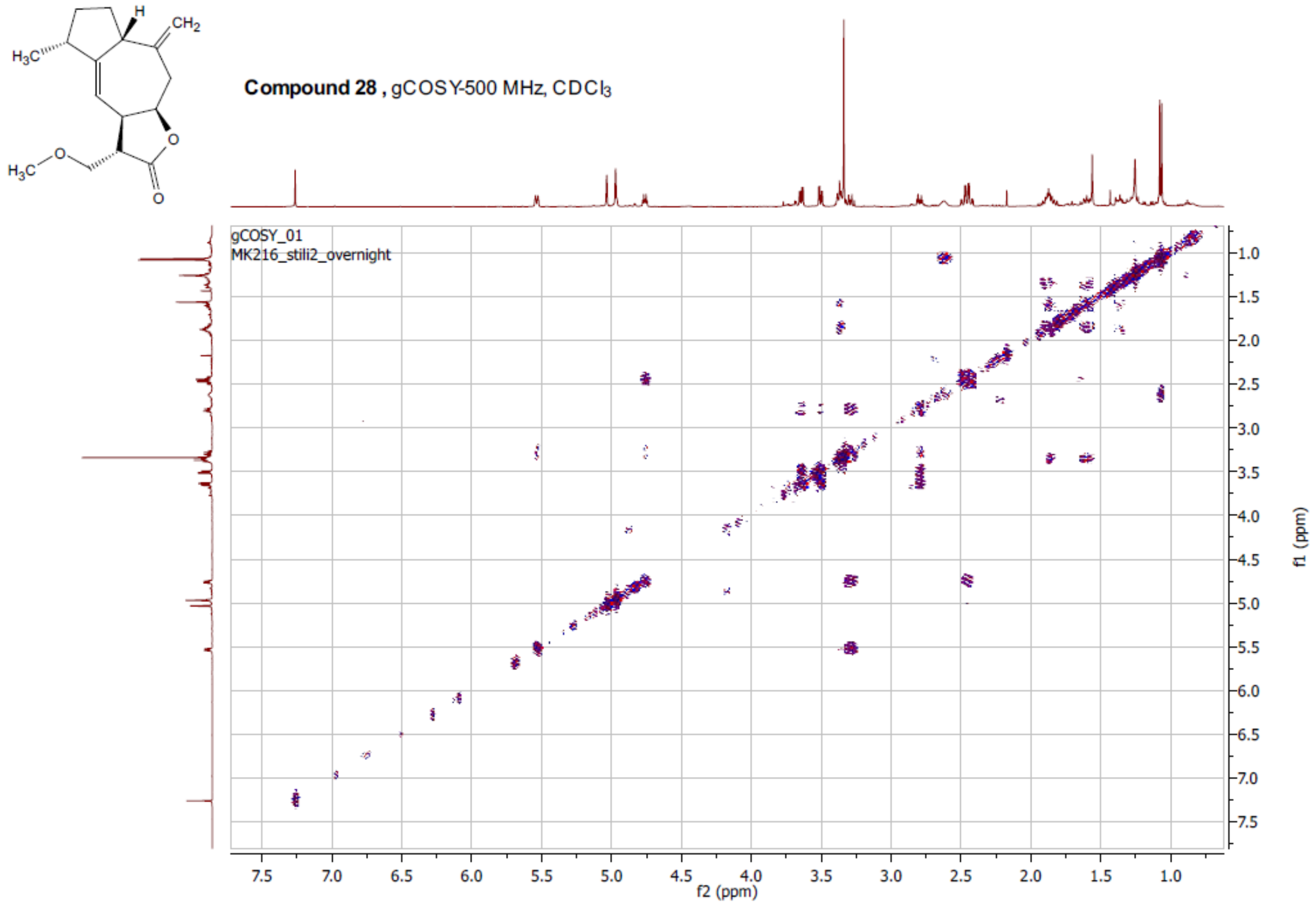
Compound 28 , gCOSY-500 MHz, $CDCl_3$
gCOSY_01
MK216_stili2_overnight
f2 (ppm)
f1 (ppm)

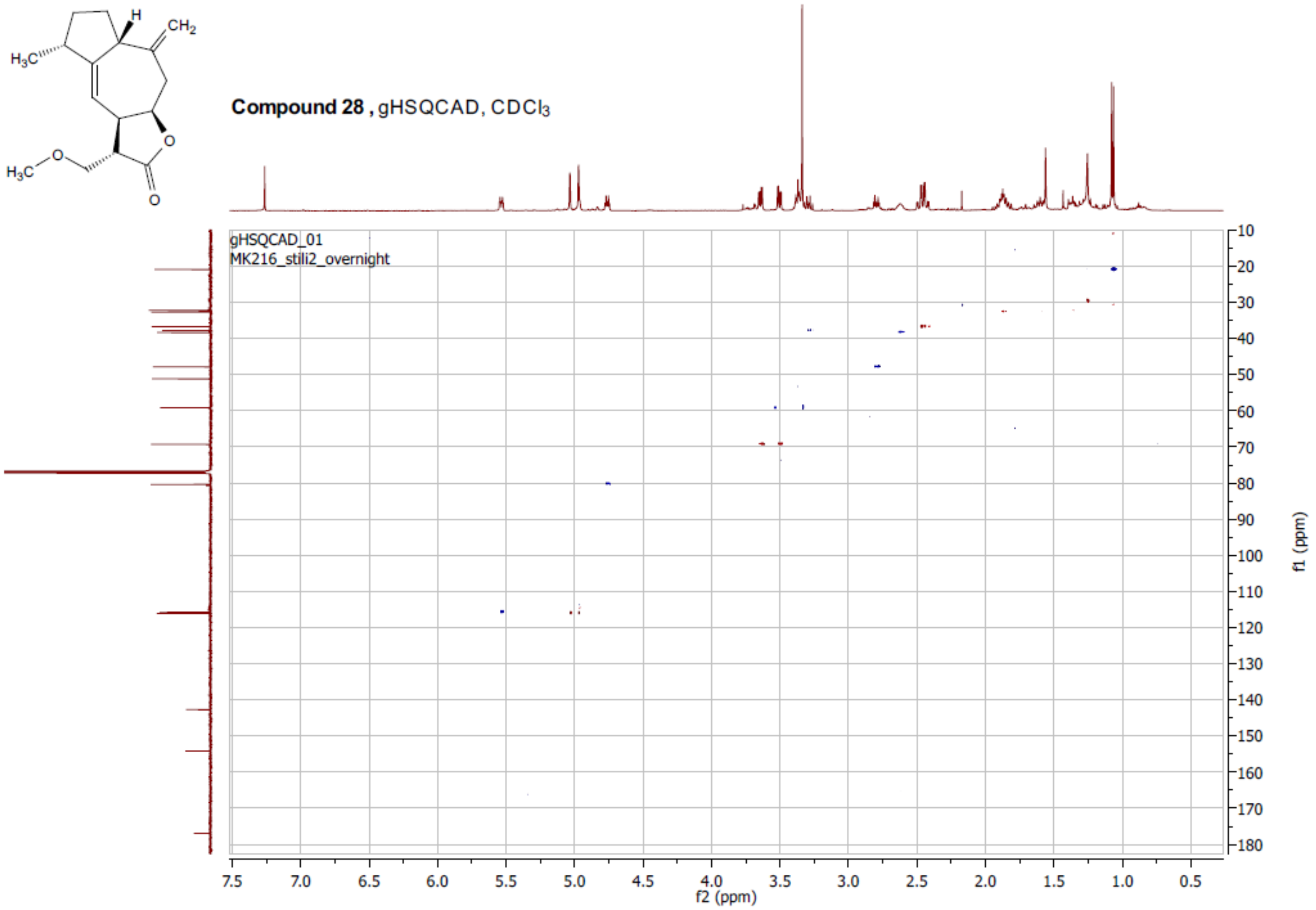
Compound 28 , gHSQCAD, CDCl3
gHSQCAD_01
MK216_stili2_overnight
f2 (ppm)
f1 (ppm)

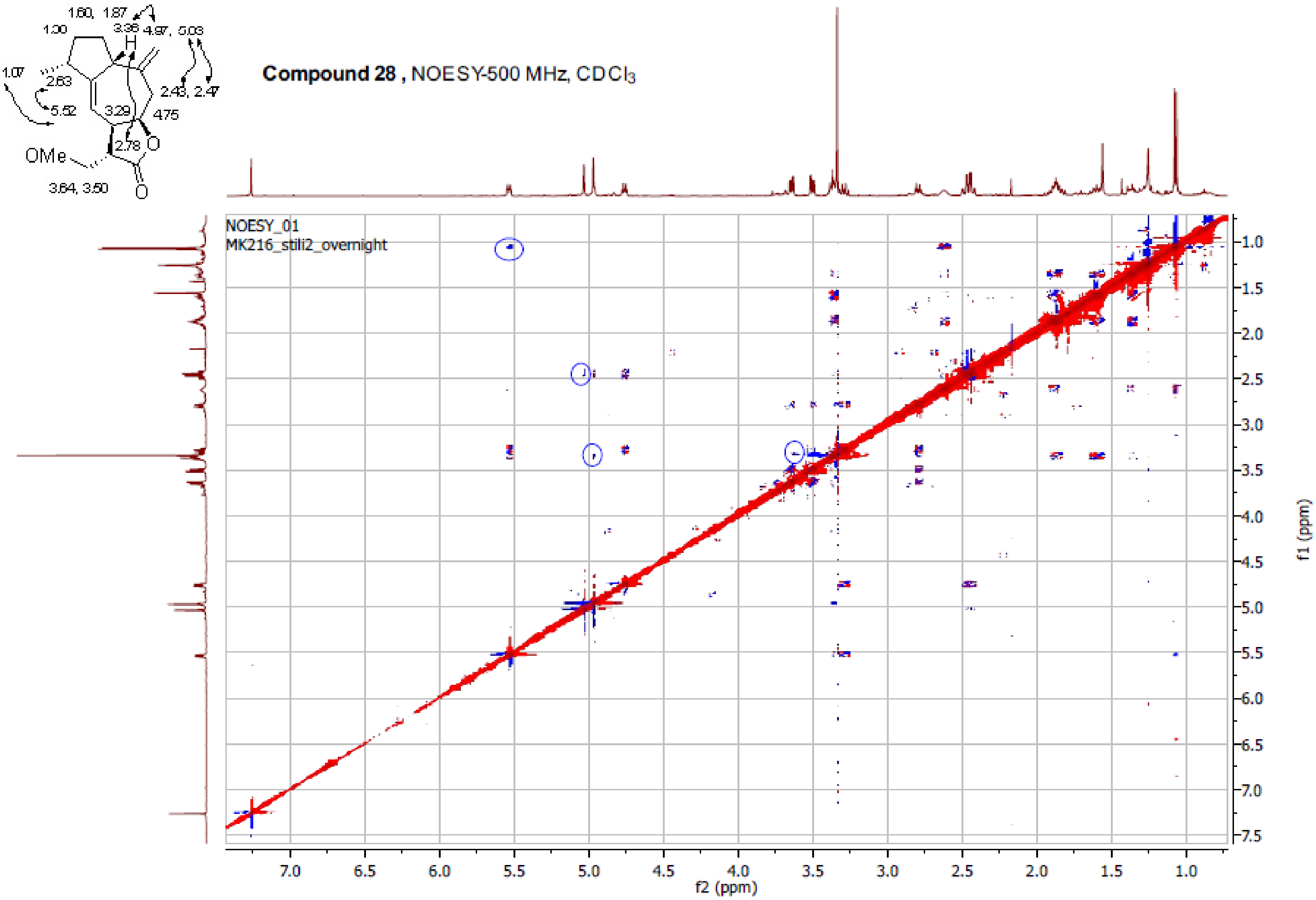
Compound 28 , NOESY-500 MHz, CDCl3
NOESY_01
MK216_stili2_overnight
f2 (ppm)
f1 (ppm)

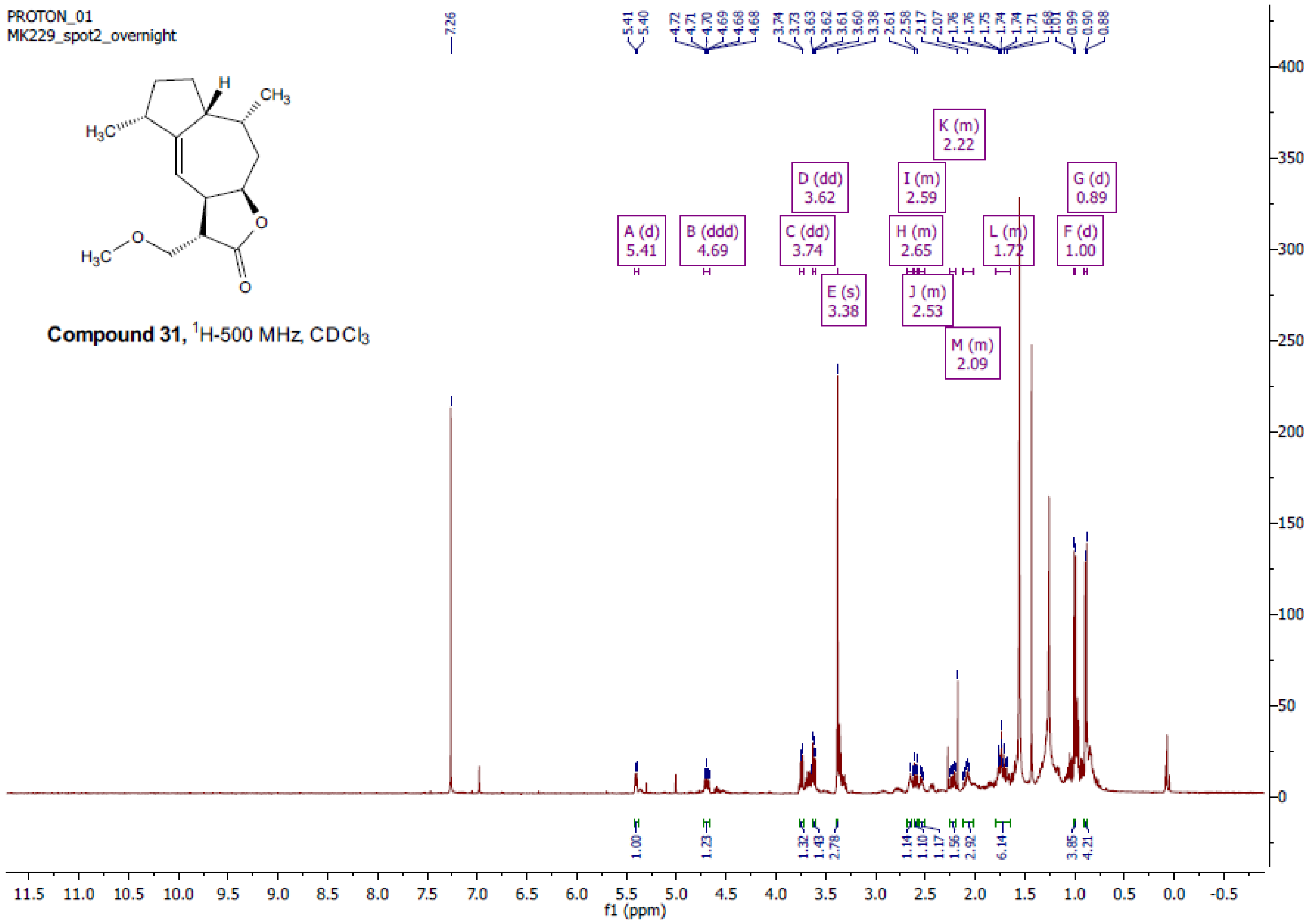
PROTON_01
MK229_spot2_overnight
Compound 31, 1H-500 MHz, CDCl3
A (d) 5.41
B (ddd) 4.69
C (dd) 3.74
D (dd) 3.62
E (s) 3.38
H (m) 2.65
I (m) 2.59
J (m) 2.53
K (m) 2.22
M (m) 2.09
L (m) 1.72
F (d) 1.00
G (d) 0.89
f1 (ppm)

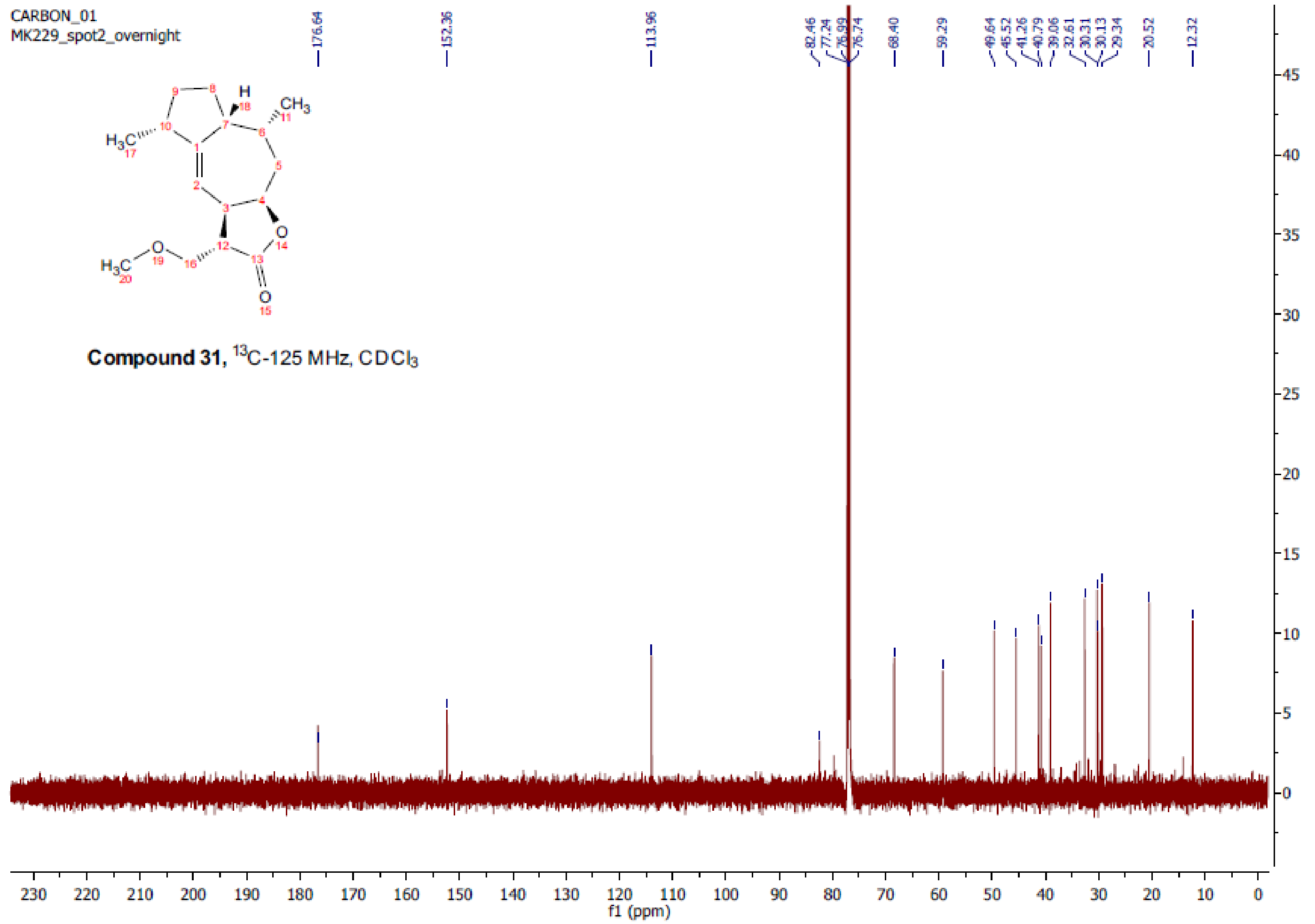
CARBON_01
MK229_spot2_overnight
Compound 31, 13C-125 MHz, CDCl3
176.64
152.36
113.96
82.46
77.24
76.99
76.74
68.40
59.29
f1 (ppm)

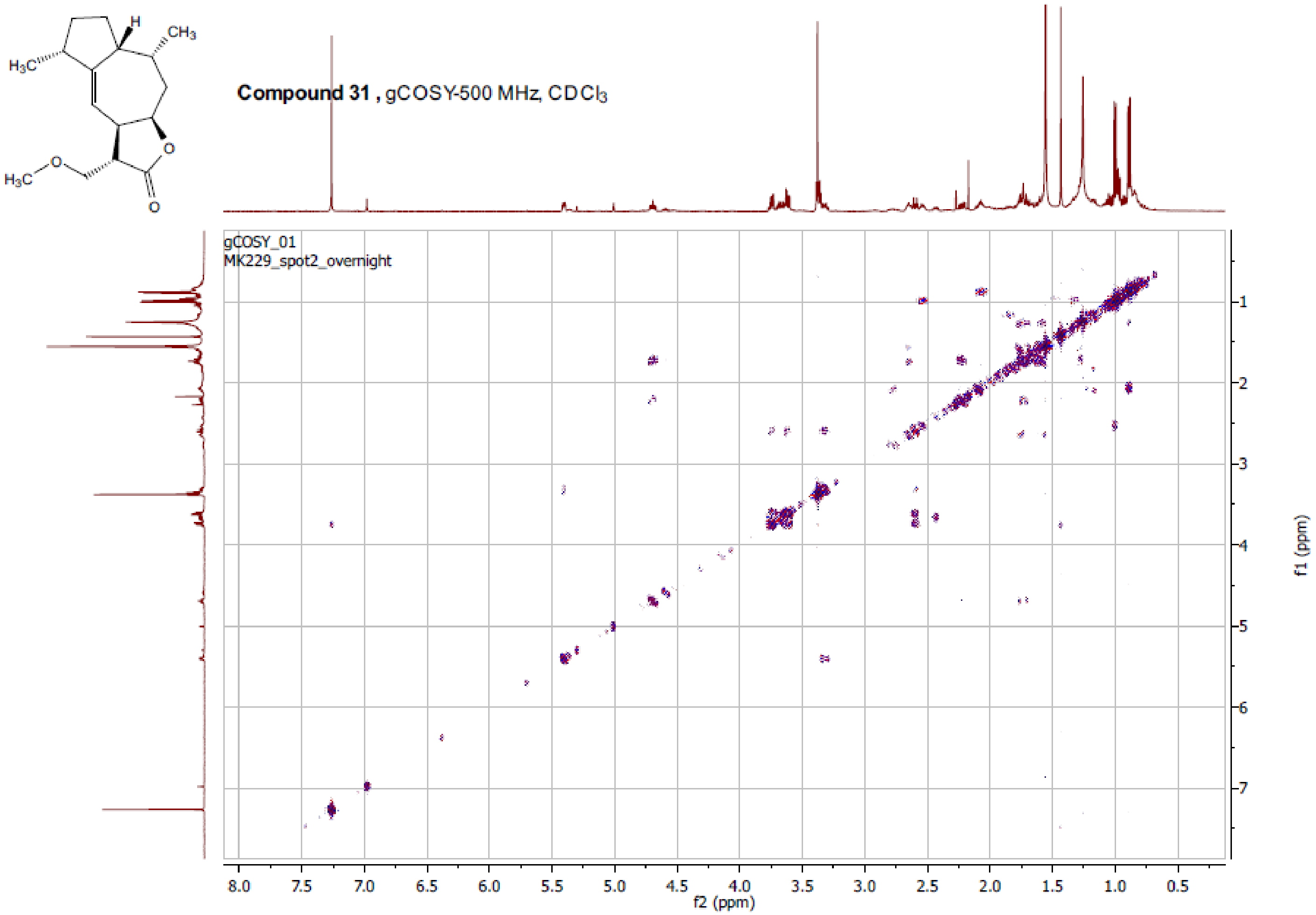
Compound 31 , gCOSY-500 MHz, CDCl3
gCOSY_01
MK229_spot2_overnight
f2 (ppm)
f1 (ppm)

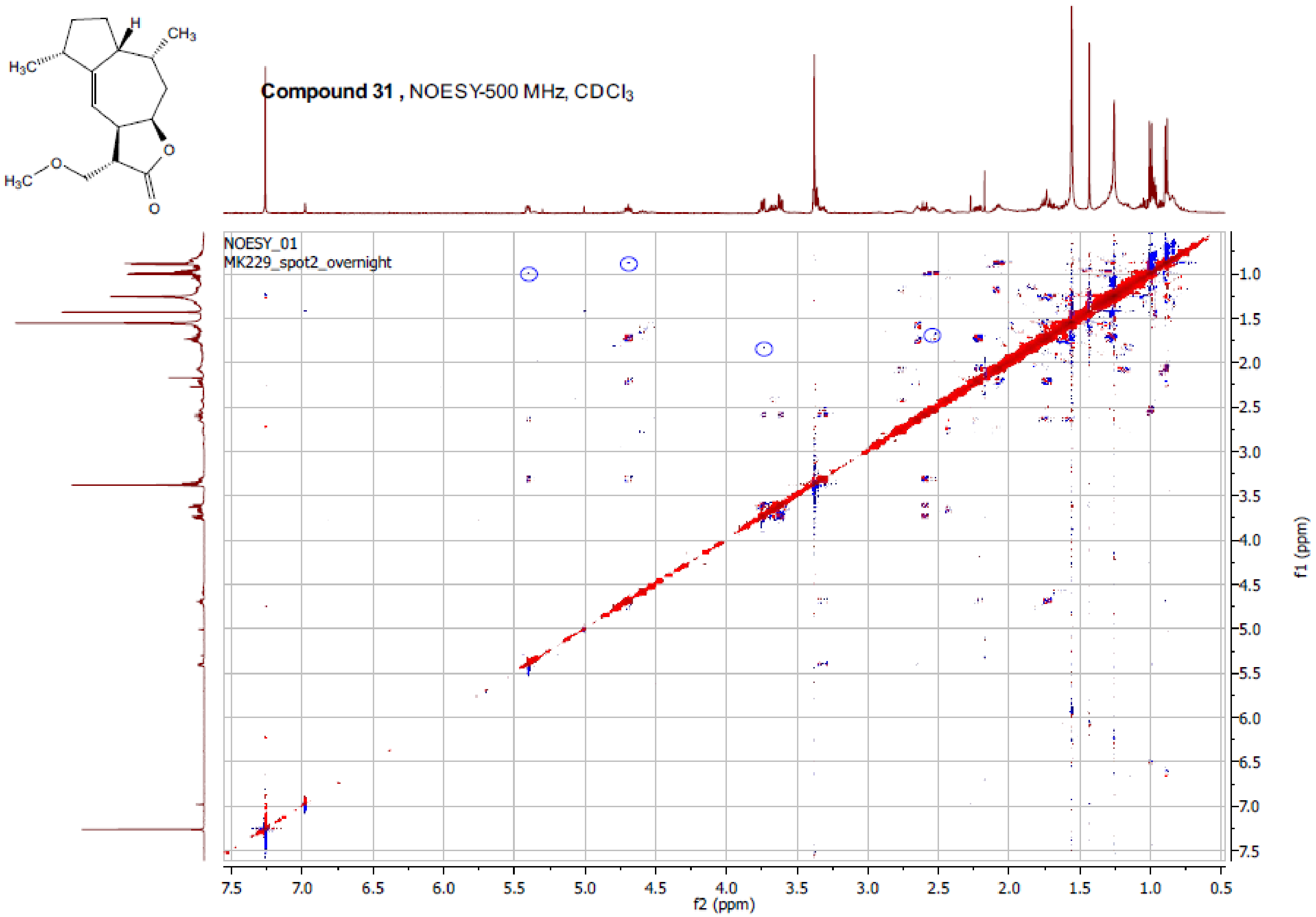
Compound 31 , NOESY-500 MHz, CDCl3
NOESY_01
MK229_spot2_overnight
f2 (ppm)
f1 (ppm)

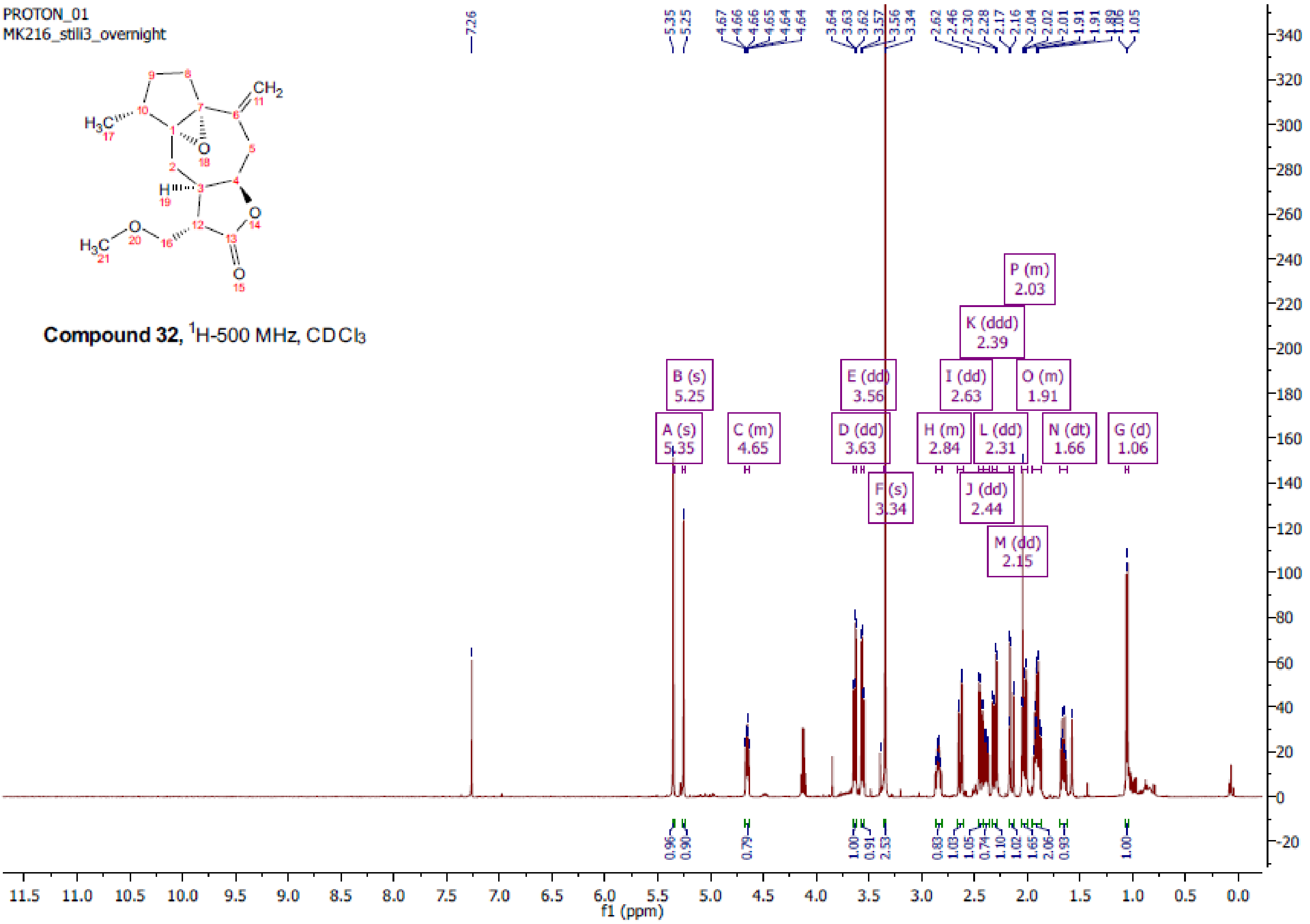
PROTON_01
MK216_stili3_overnight
Compound 32, 1H-500 MHz, CDCl3
A (s) 5.35
B (s) 5.25
C (m) 4.65
D (dd) 3.63
E (dd) 3.56
F (s) 3.34
G (d) 1.06
H (m) 2.84
I (dd) 2.63
J (dd) 2.44
K (ddd) 2.39
L (dd) 2.31
M (dd) 2.15
N (dt) 1.66
O (m) 1.91
P (m) 2.03
f1 (ppm)

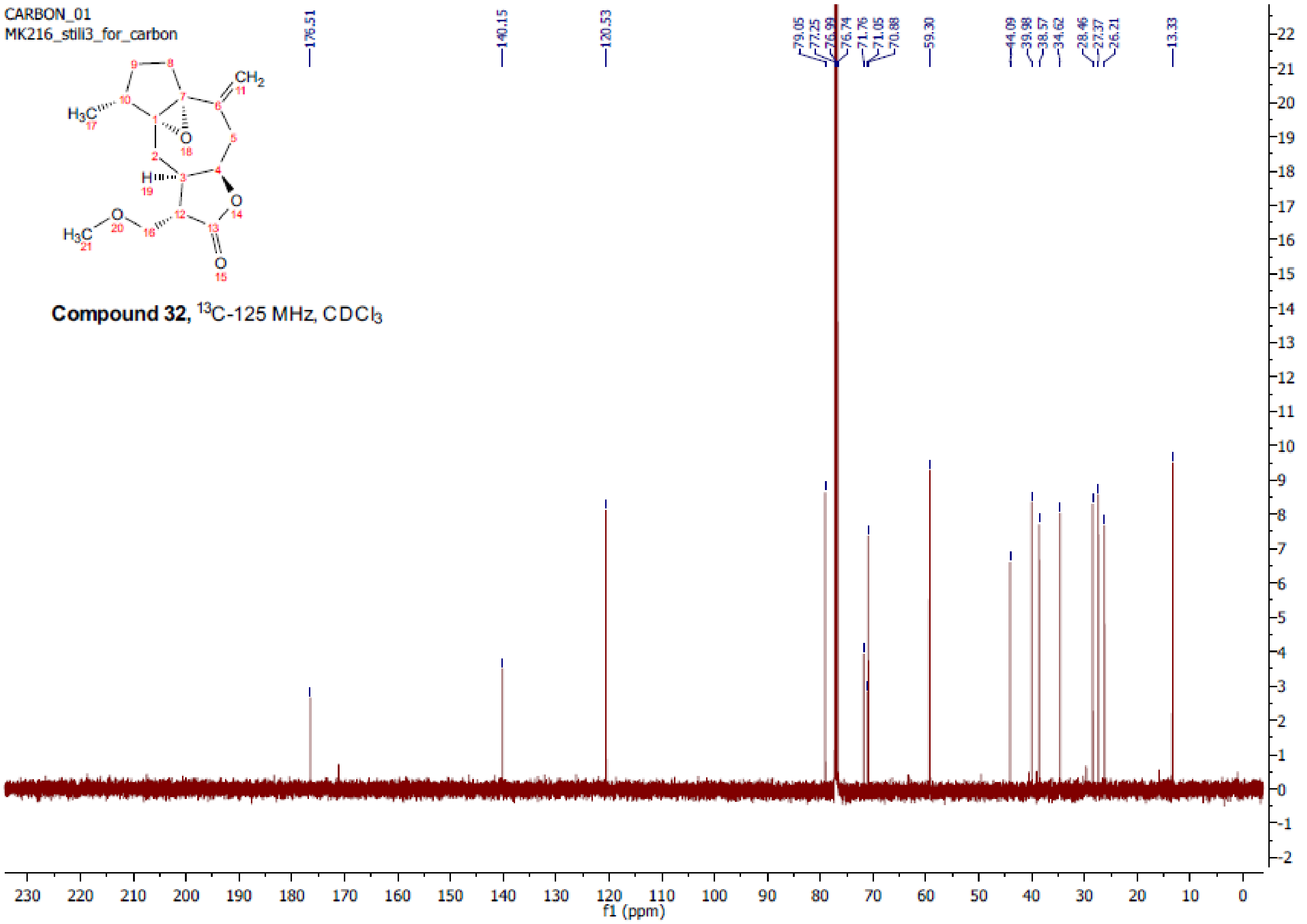
CARBON_01
MK216_stili3_for_carbon
Compound 32, 13C-125 MHz, CDCl3
176.51
140.15
120.53
79.05
77.25
76.99
76.74
71.76
71.05
70.88
59.30
44.09
39.98
38.57
34.62
28.46
27.37
26.21
13.33
f1 (ppm)

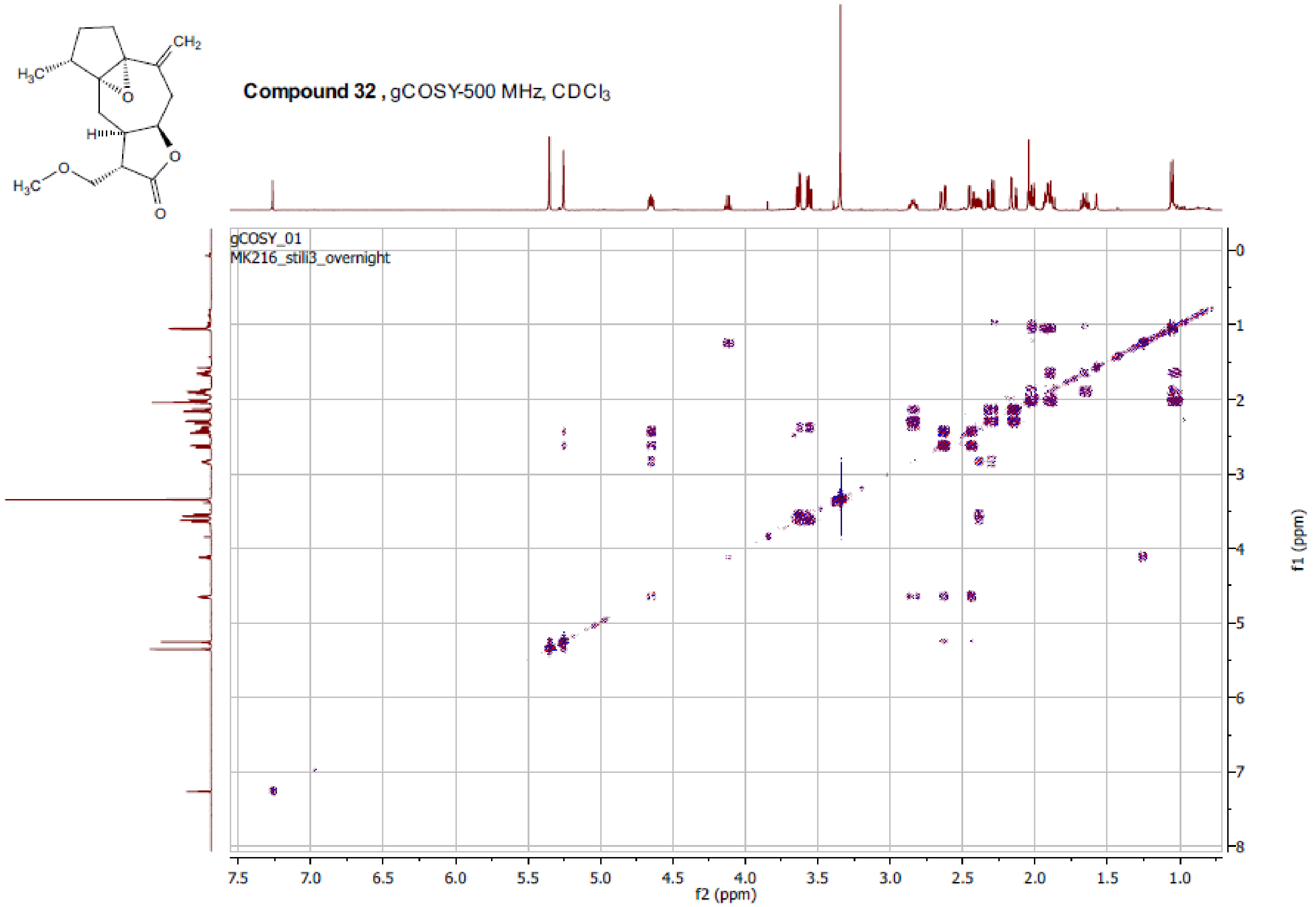

Compound 32, gCOSY-500 MHz, $CDCl_3$

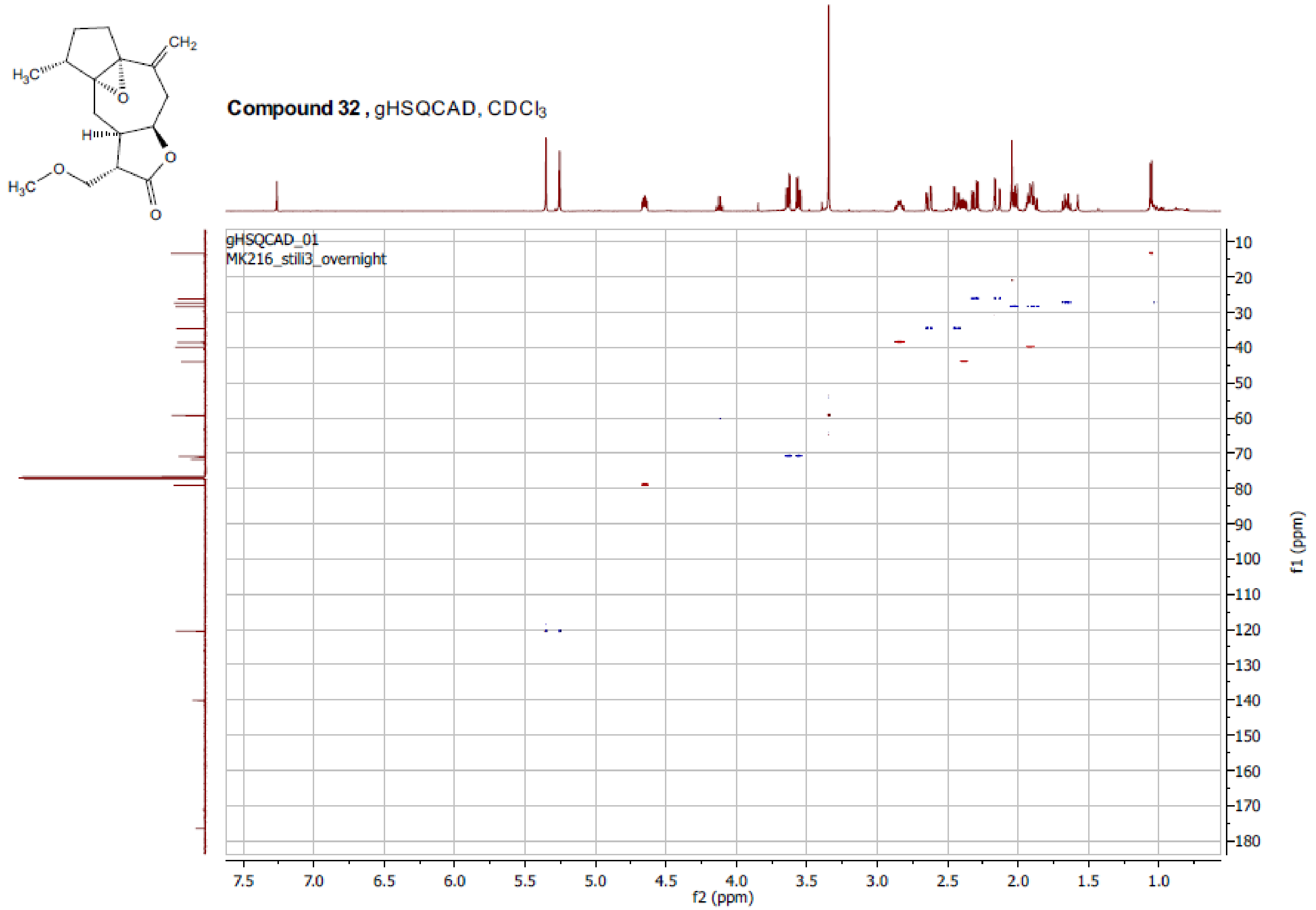

Compound 32, gHSQCAD, $CDCl_3$

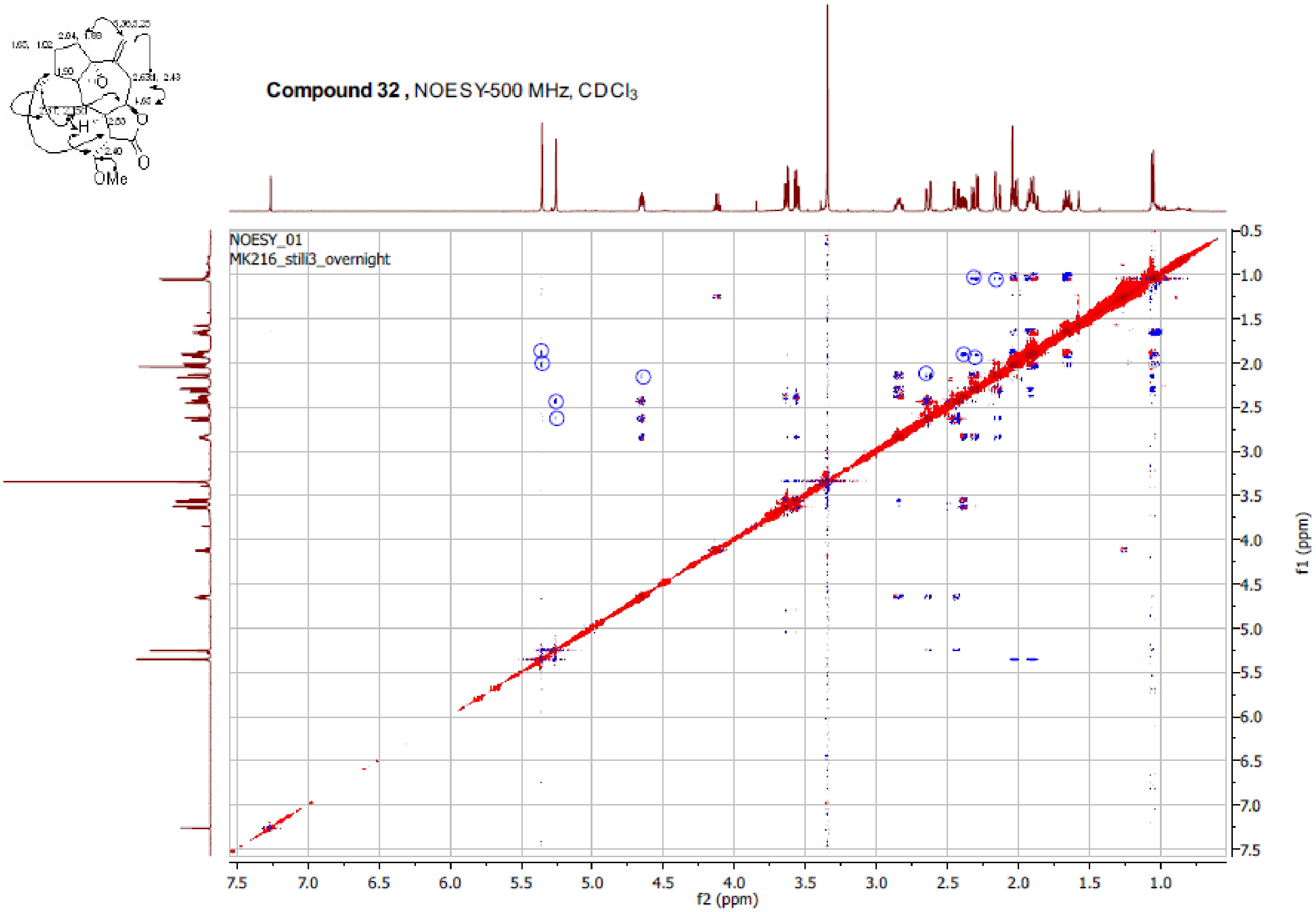
Compound 32 , NOESY-500 MHz, CDCl3
NOESY_01
MK216_stili3_overnight
f2 (ppm)
f1 (ppm)
OMe

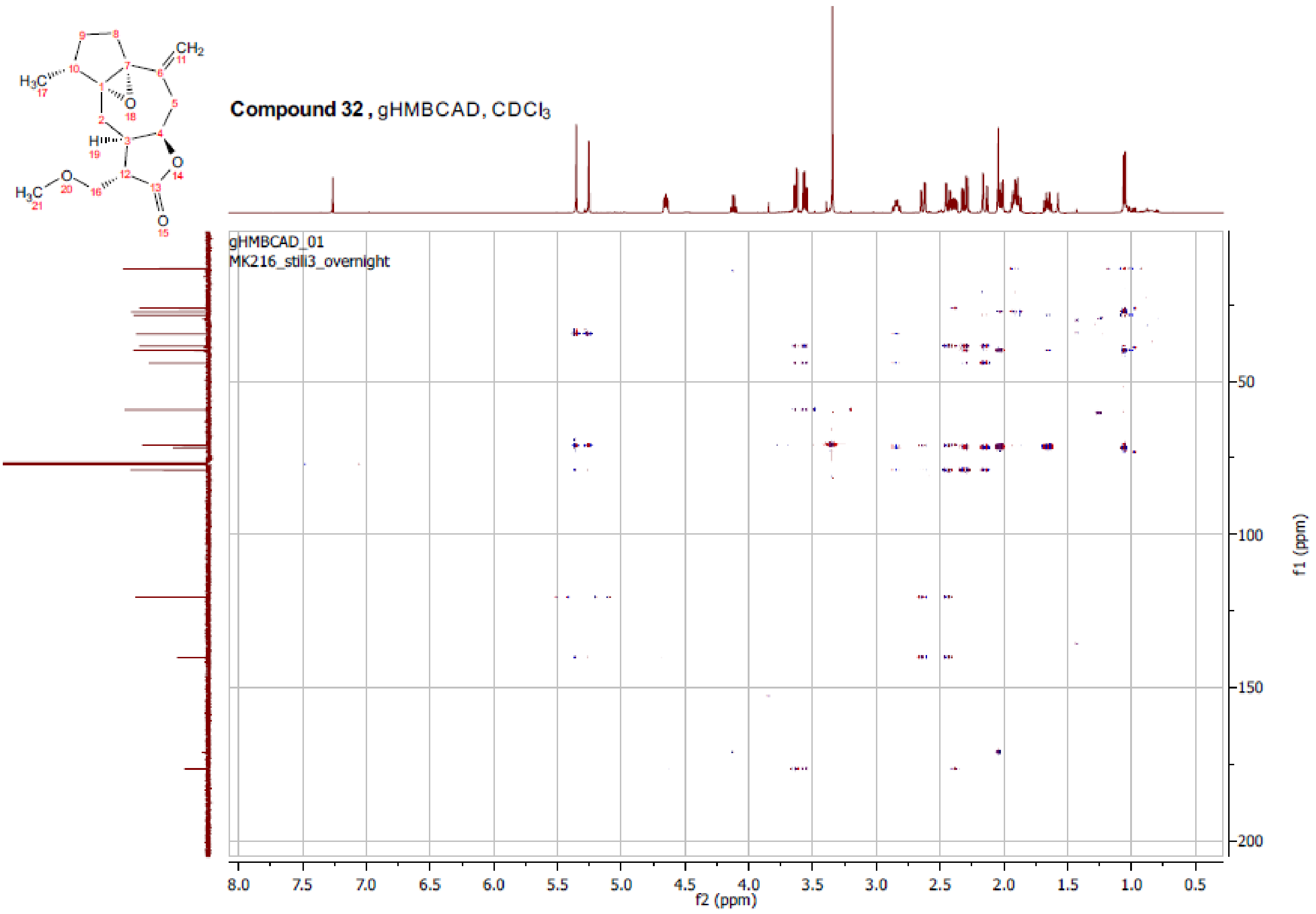
Compound 32 , gHMBCAD, CDCl3
gHMBCAD_01
MK216_stili3_overnight
f2 (ppm)
f1 (ppm)

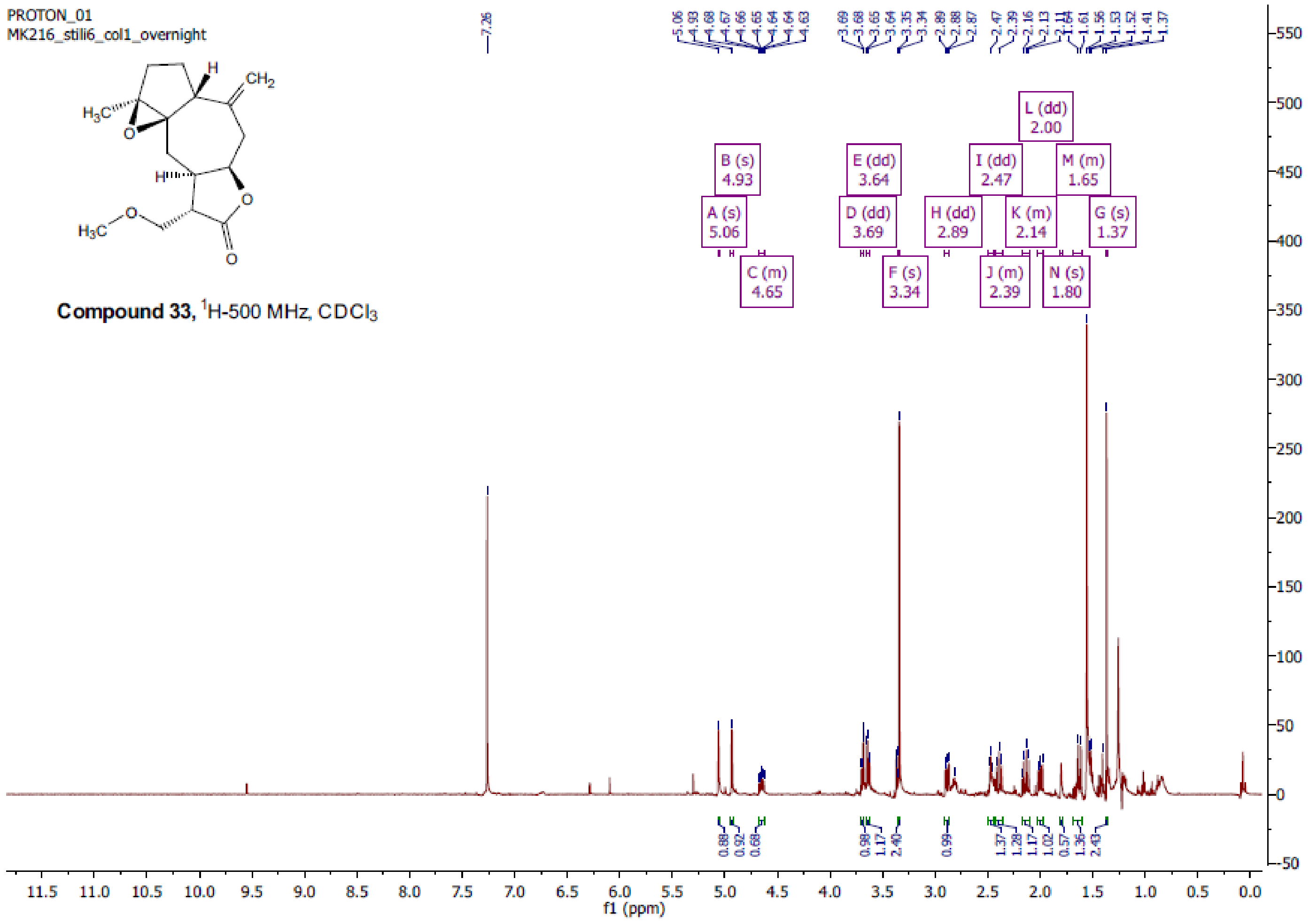
PROTON_01
MK216_stili6_col1_overnight
Compound 33, 1H-500 MHz, CDCl3
f1 (ppm)

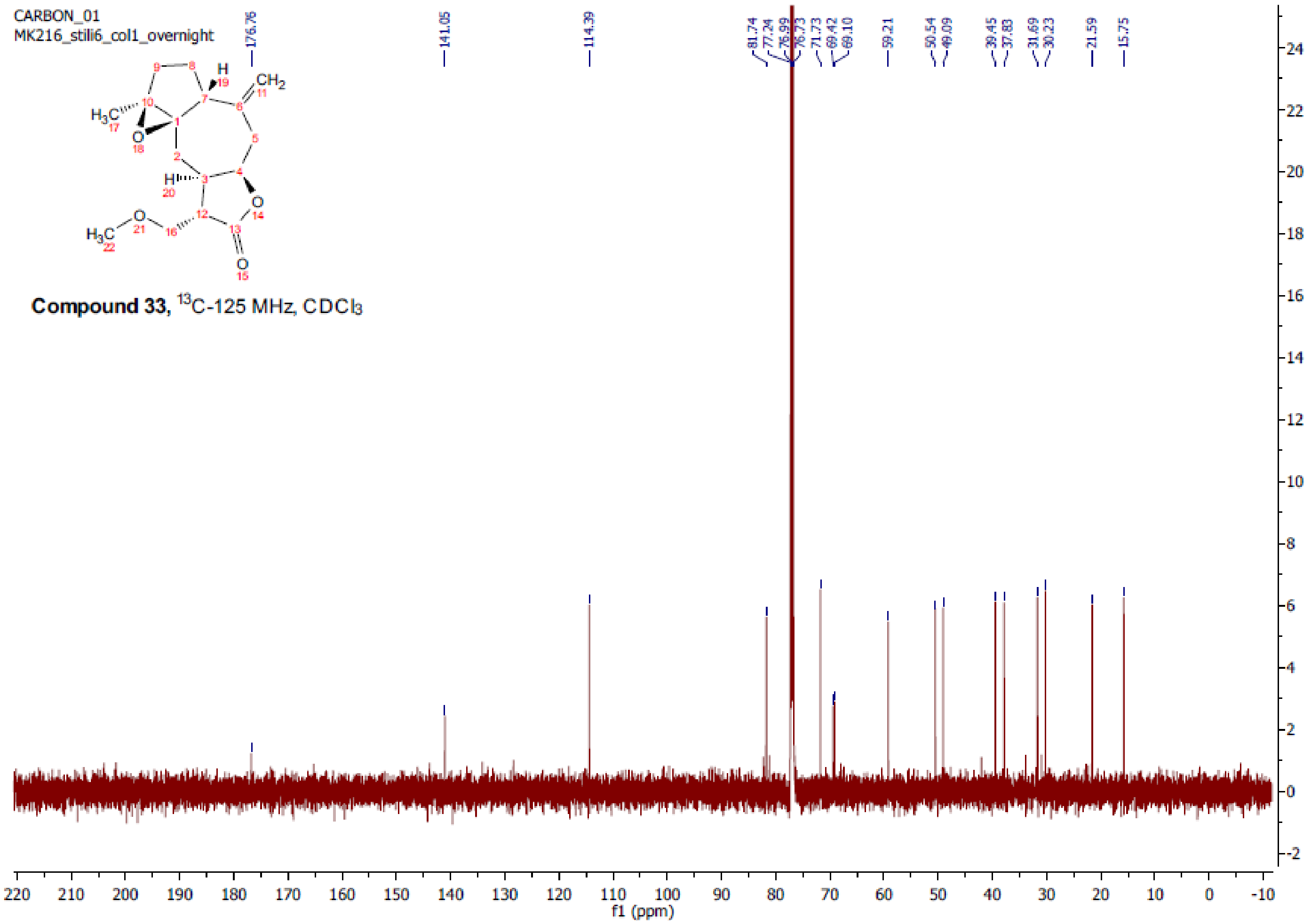
CARBON_01
MK216_stili6_col1_overnight
Compound 33, 13C-125 MHz, CDCl3
f1 (ppm)

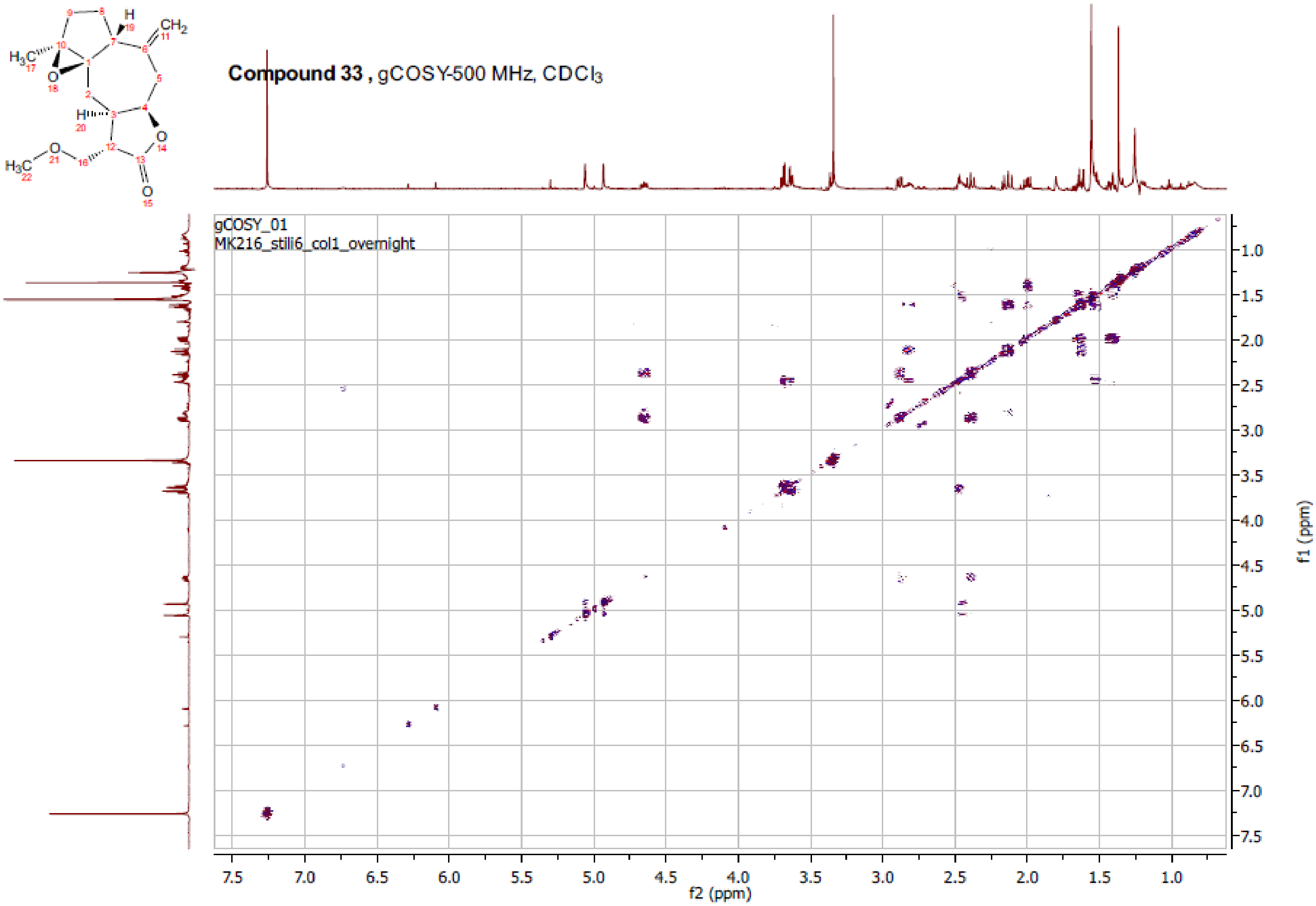
Compound 33 , gCOSY-500 MHz, CDCl3
gCOSY_01
MK216_stili6_col1_overnight
f2 (ppm)
f1 (ppm)

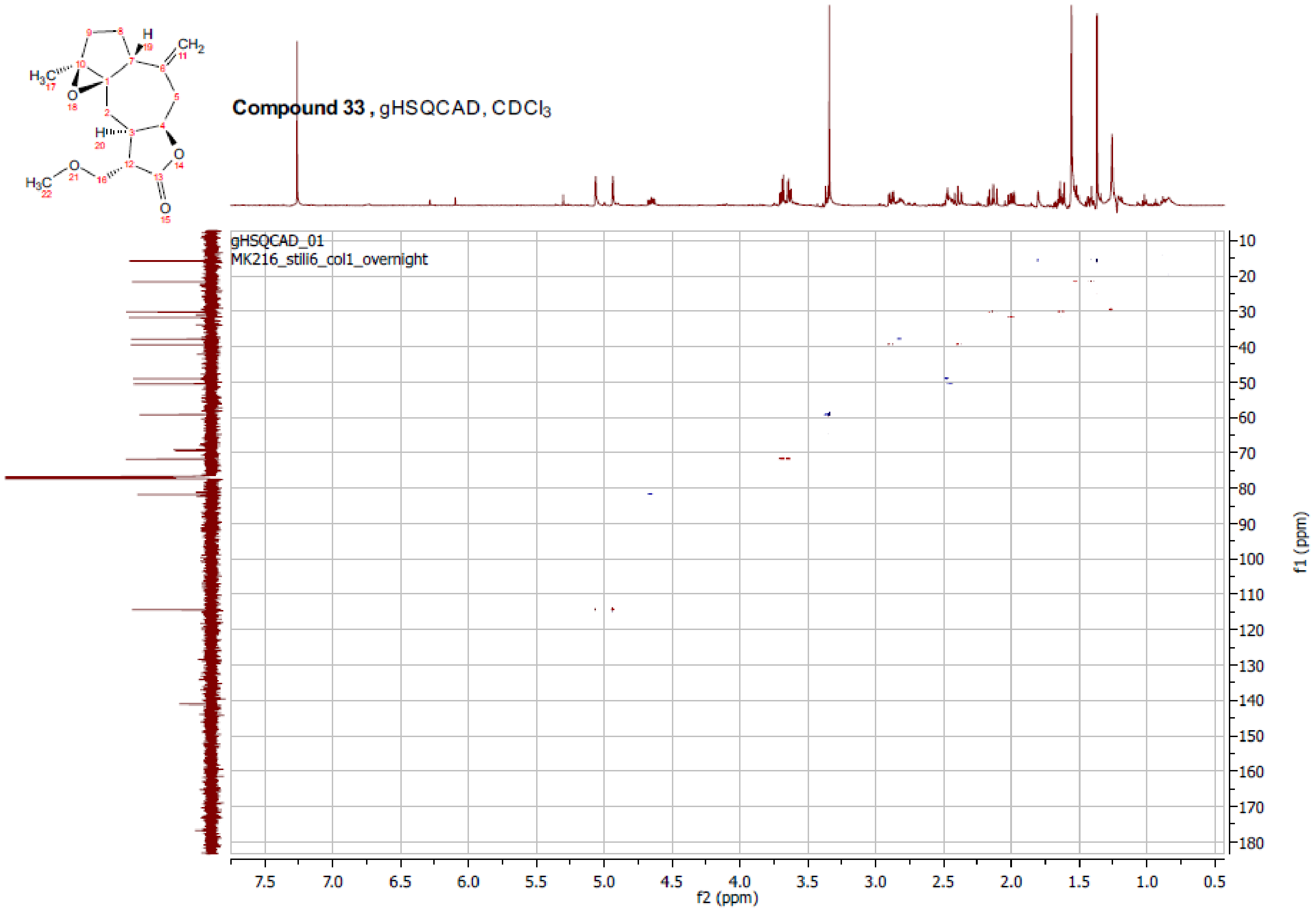
Compound 33 , gHSQCAD, CDCl3
gHSQCAD_01
MK216_stili6_col1_overnight
f2 (ppm)
f1 (ppm)

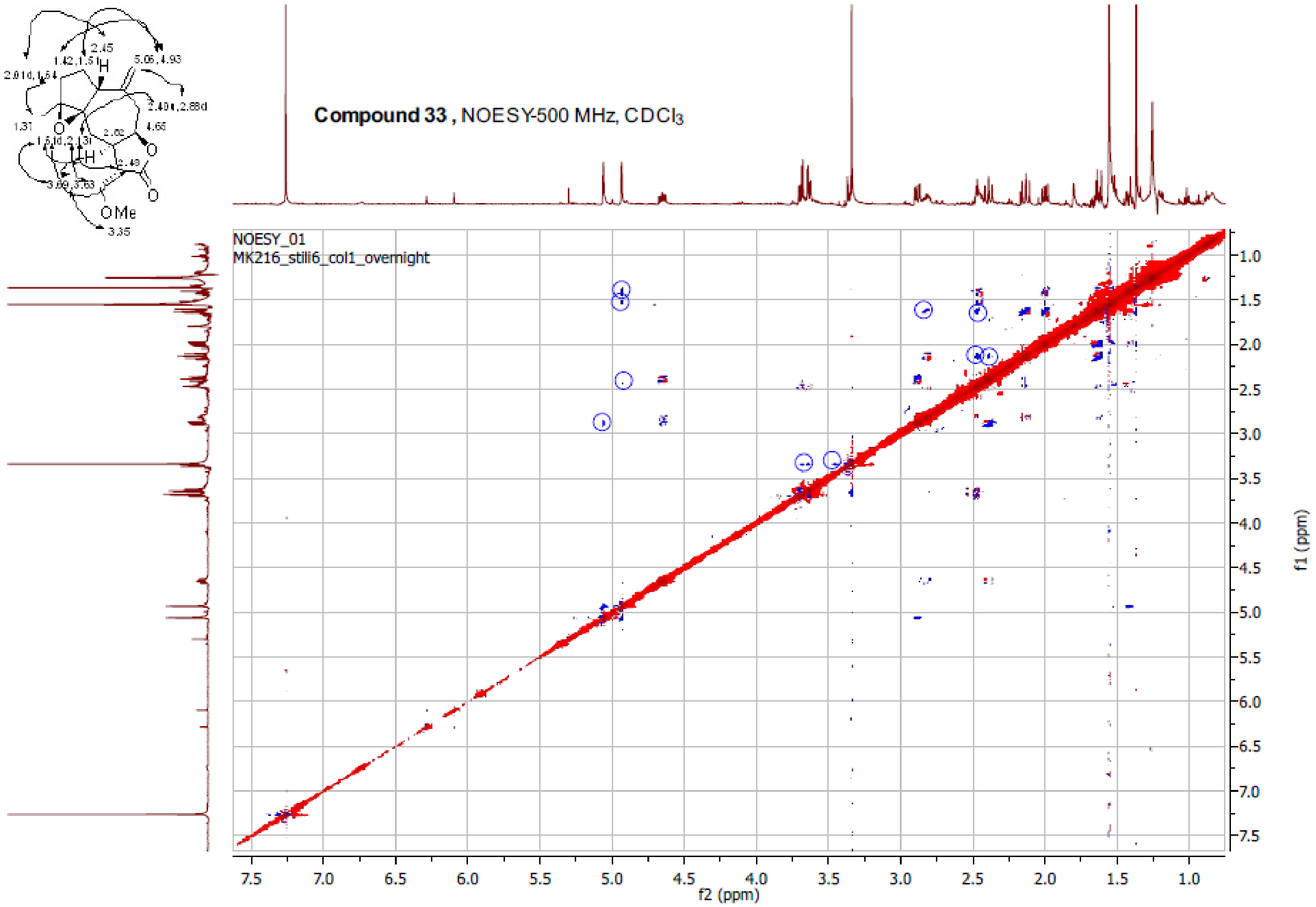
Compound 33 , NOESY-500 MHz, CDCl3
NOESY_01
MK216_stili6_col1_overnight
OMe
f2 (ppm)
f1 (ppm)

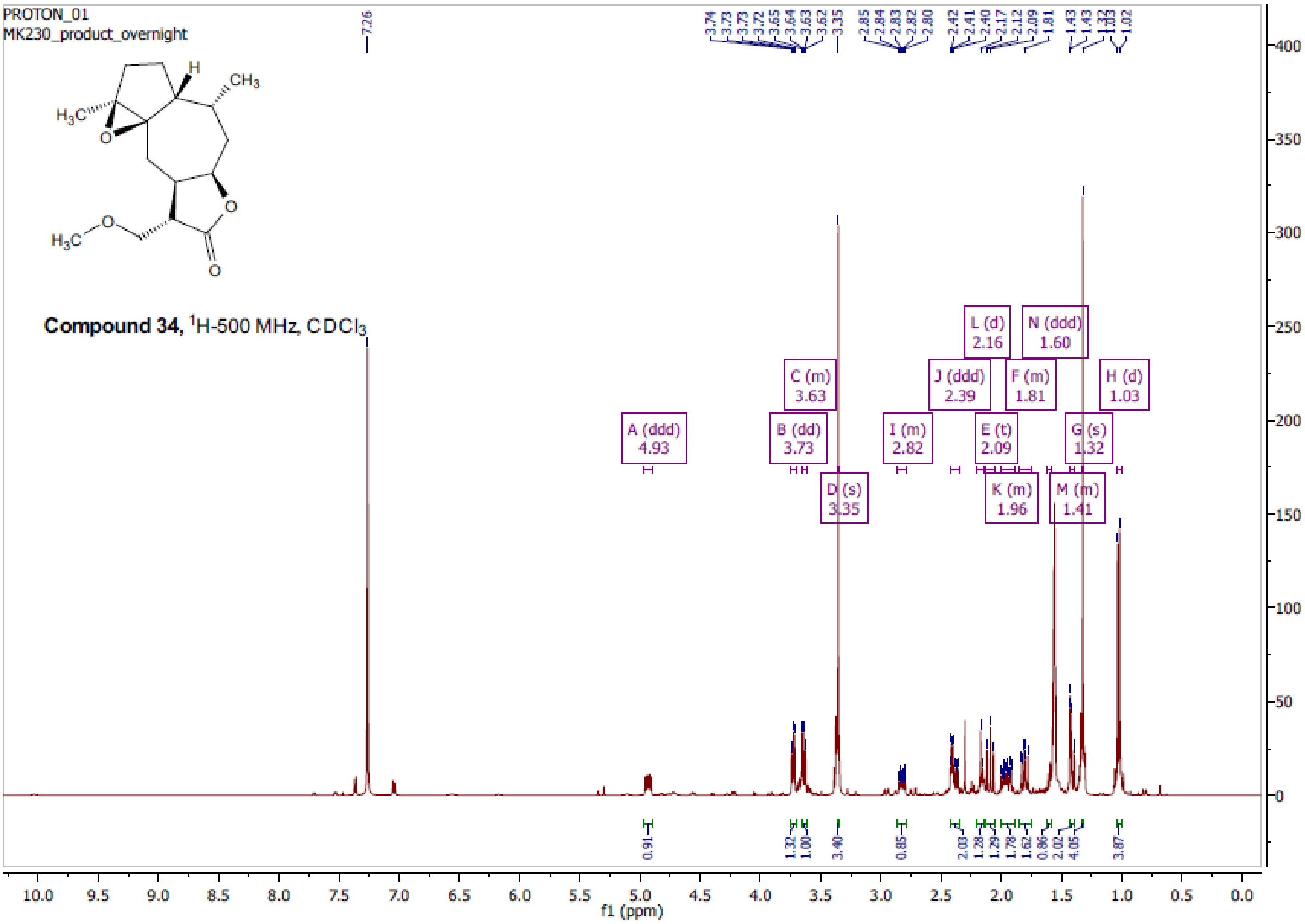
PROTON_01
MK230_product_overnight
Compound 34, 1H-500 MHz, CDCl3
A (ddd) 4.93
B (dd) 3.73
C (m) 3.63
D (s) 3.35
I (m) 2.82
J (ddd) 2.39
L (d) 2.16
E (t) 2.09
K (m) 1.96
F (m) 1.81
N (ddd) 1.60
M (m) 1.41
G (s) 1.32
H (d) 1.03
f1 (ppm)

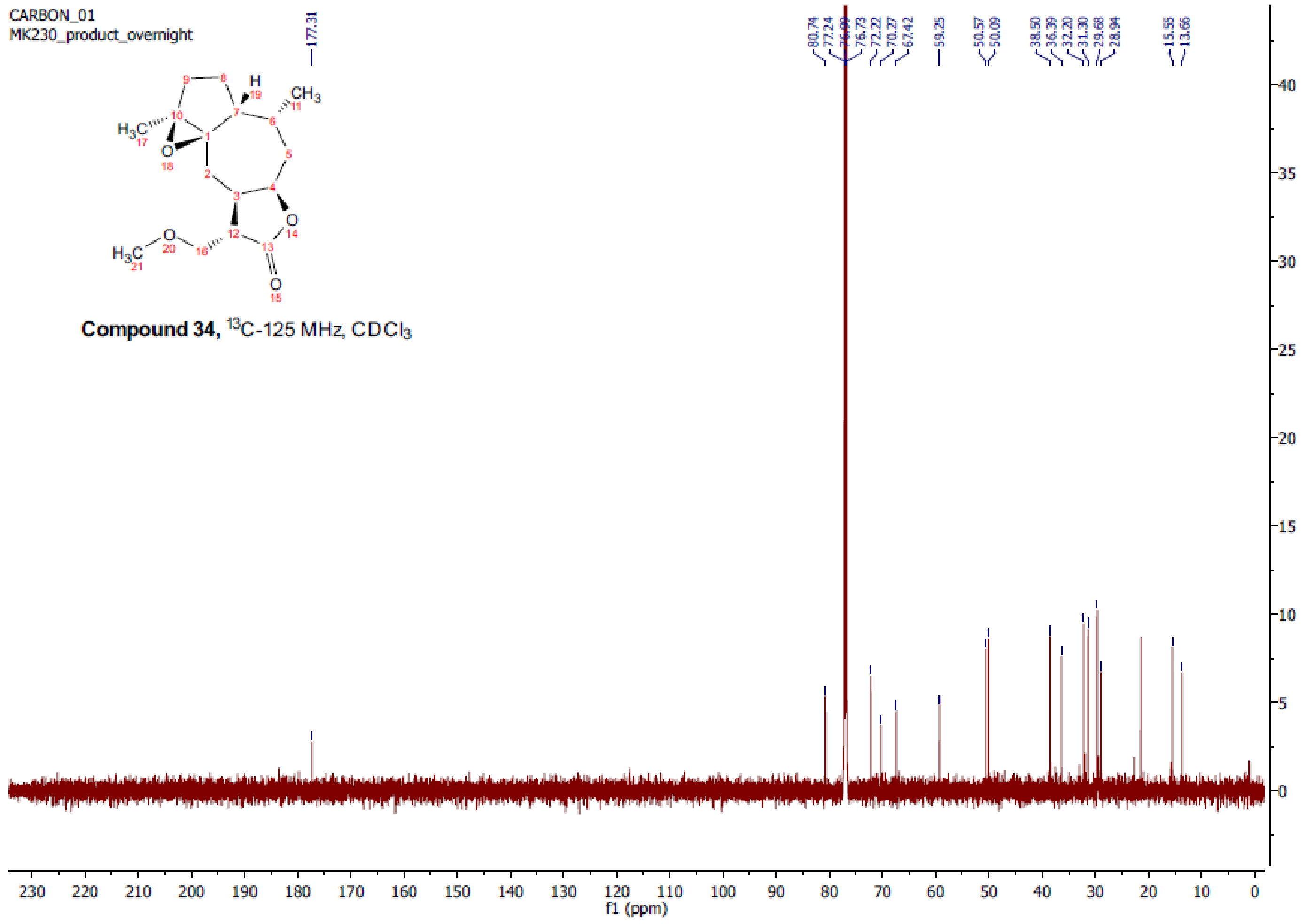

CARBON_01
MK230_product_overnight
177.31
80.74
77.24
76.99
76.73
72.22
70.27
67.42
59.25
50.57
50.09
38.50
36.39
32.20
31.30
29.68
28.94
15.55
13.66
Compound 34, 13C-125 MHz, CDCl3
f1 (ppm)

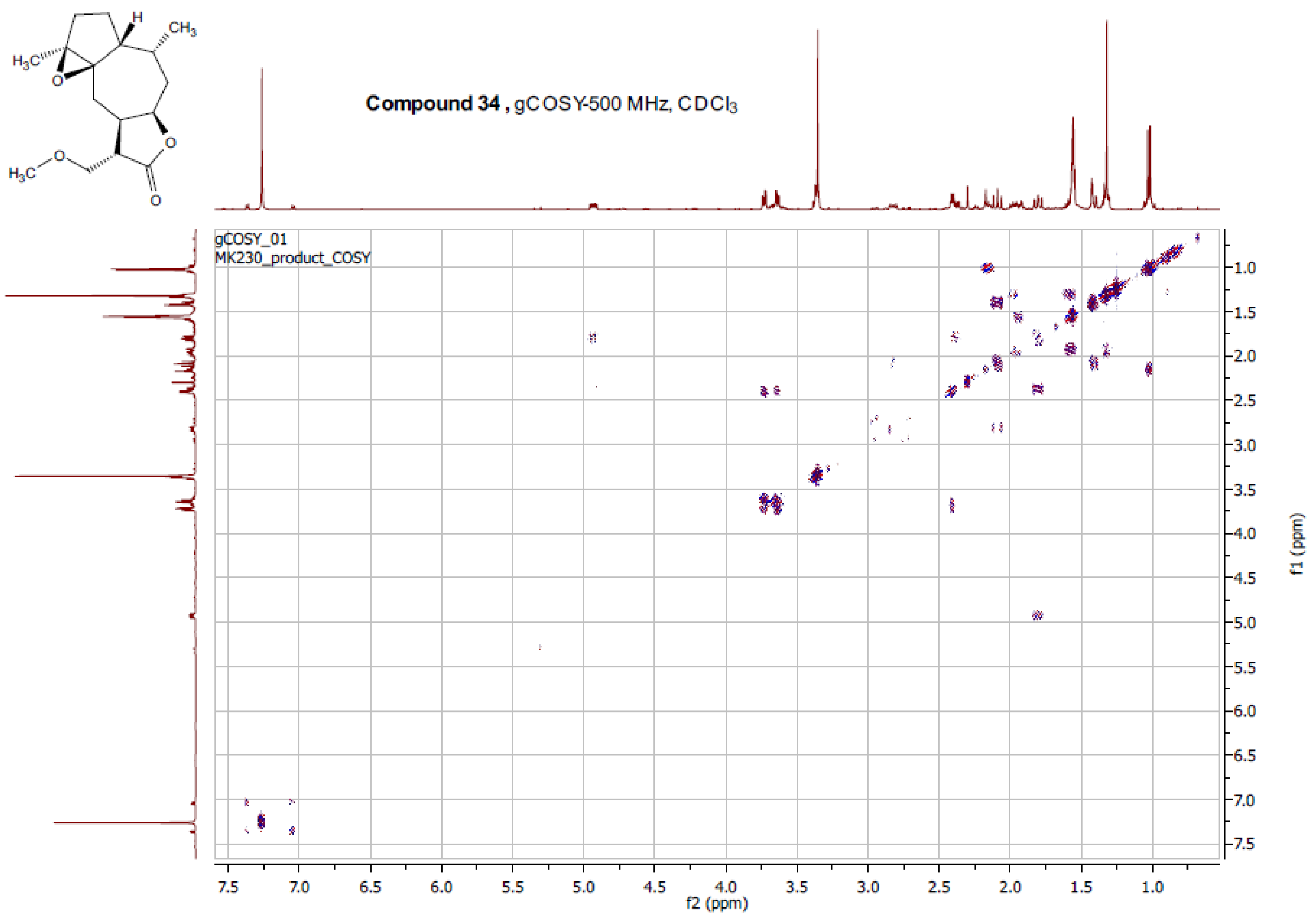

Compound 34 , gCOSY-500 MHz, CDCl3
gCOSY_01
MK230_product_COSY
f2 (ppm)
f1 (ppm)

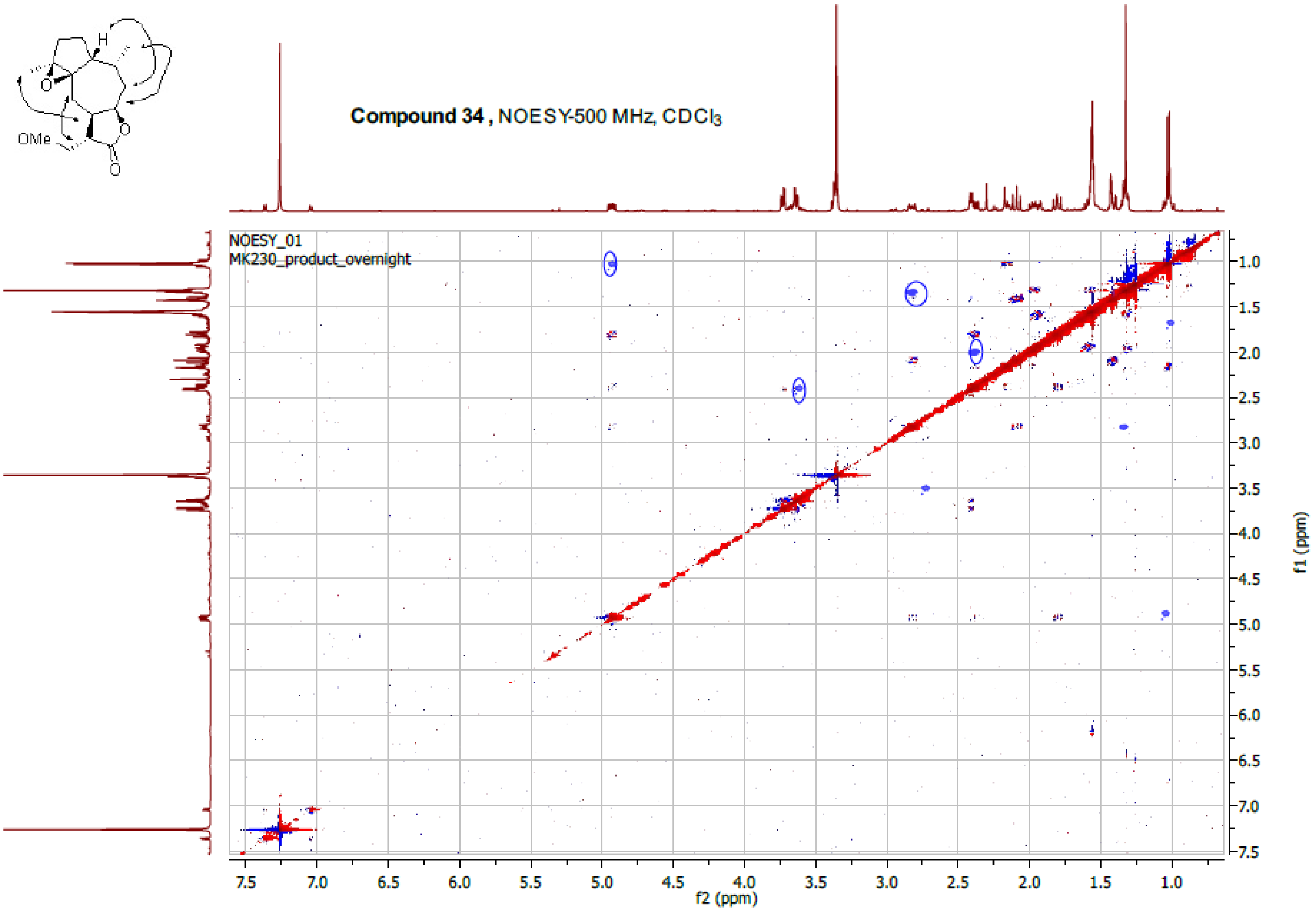
OMe
Compound 34 , NOESY-500 MHz, CDCl3
NOESY_01
MK230_product_overnight
f2 (ppm)
f1 (ppm)

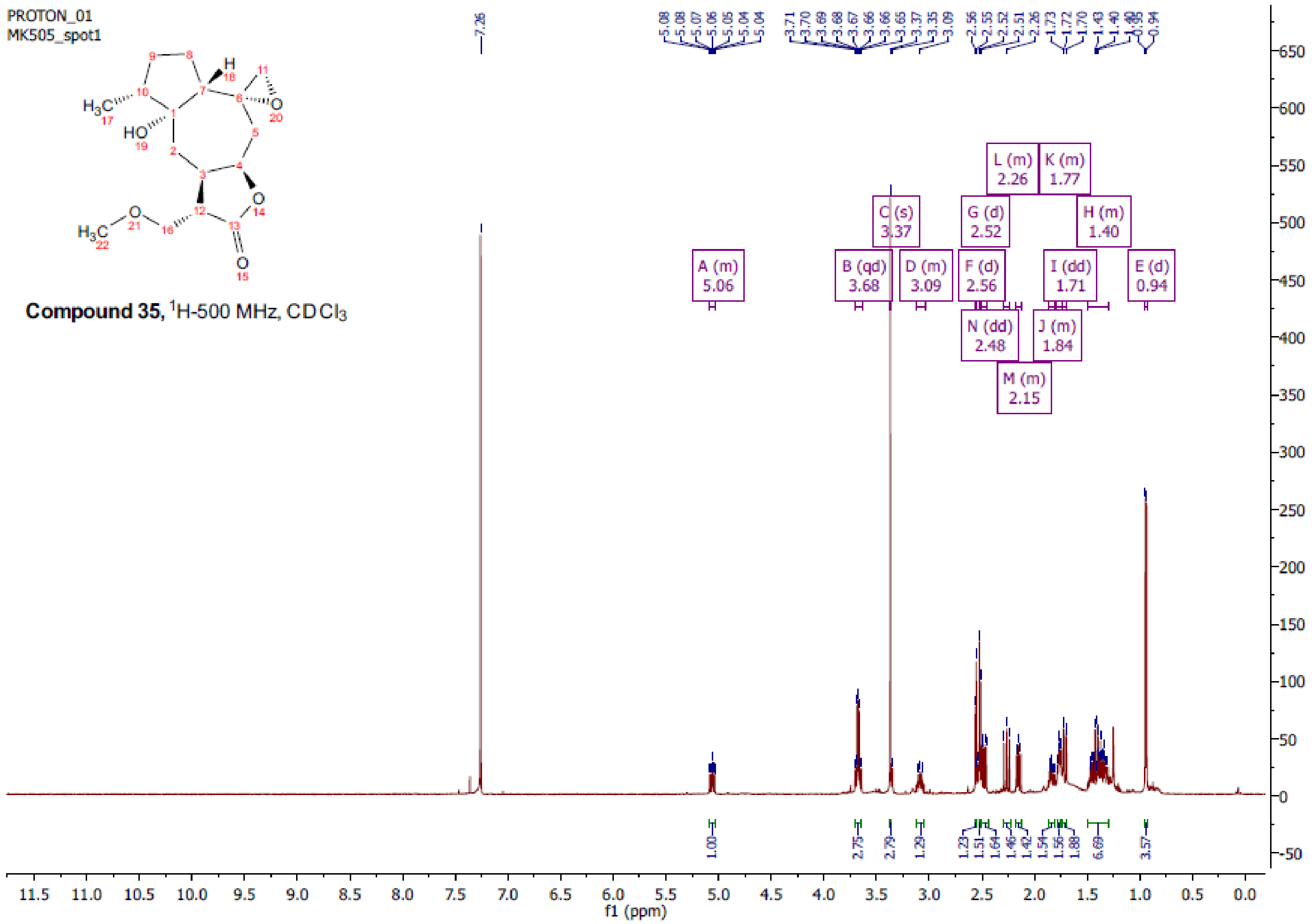
PROTON_01
MK505_spot1
H3C
HO
H3C
Compound 35, 1H-500 MHz, CDCl3
A (m) 5.06
B (qd) 3.68
C (s) 3.37
D (m) 3.09
E (d) 0.94
F (d) 2.56
G (d) 2.52
H (m) 1.40
I (dd) 1.71
J (m) 1.84
K (m) 1.77
L (m) 2.26
M (m) 2.15
N (dd) 2.48
f1 (ppm)

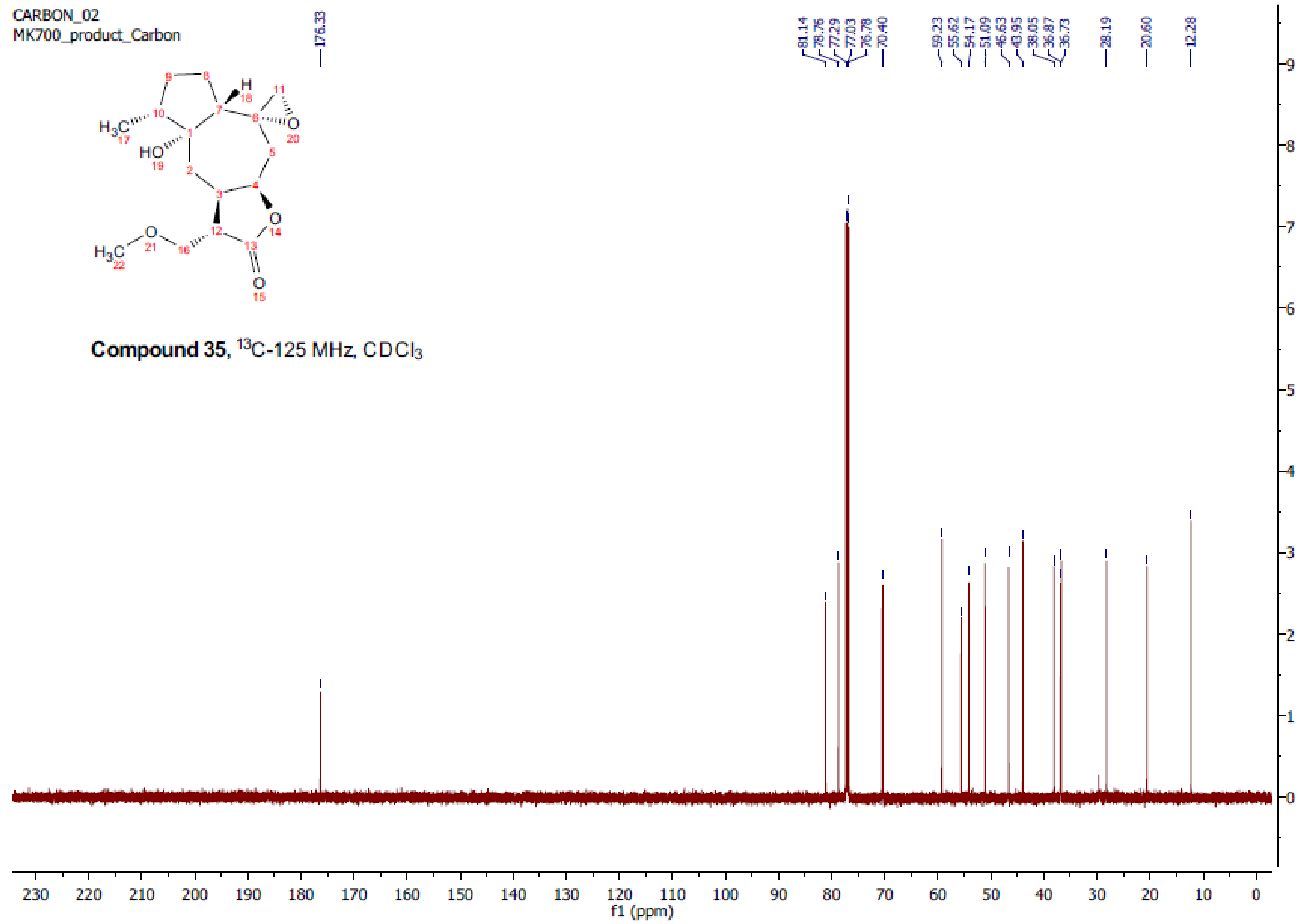
CARBON_02
MK700_product_Carbon
Compound 35, 13C-125 MHz, CDCl3
176.33
81.14
78.76
77.29
77.03
76.78
70.40
59.23
55.62
54.17
51.09
46.63
43.95
38.05
36.87
36.73
28.19
20.60
12.28
f1 (ppm)

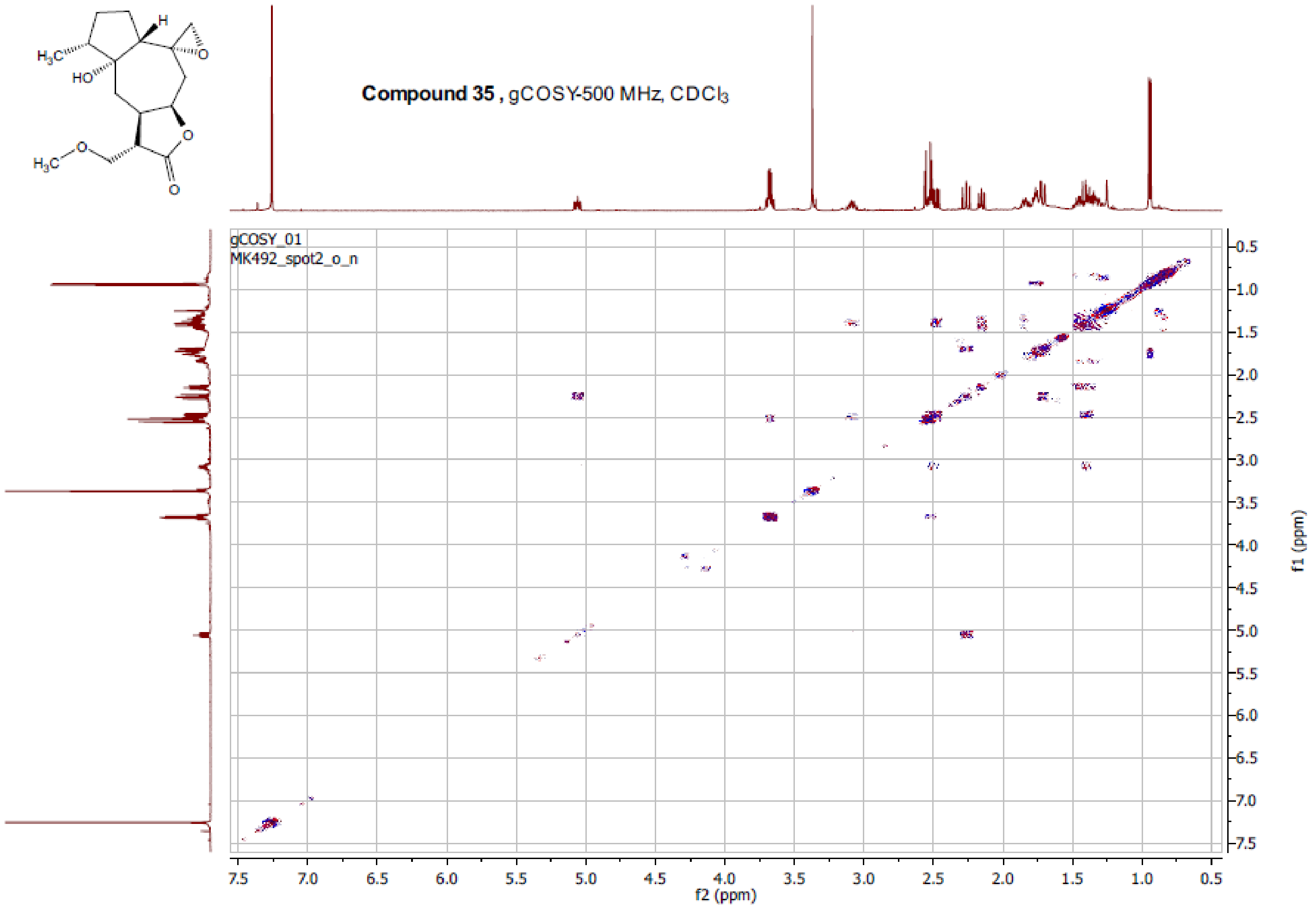
Compound 35 , gCOSY-500 MHz, CDCl3
gCOSY_01
MK492_spot2_o_n
f2 (ppm)
f1 (ppm)

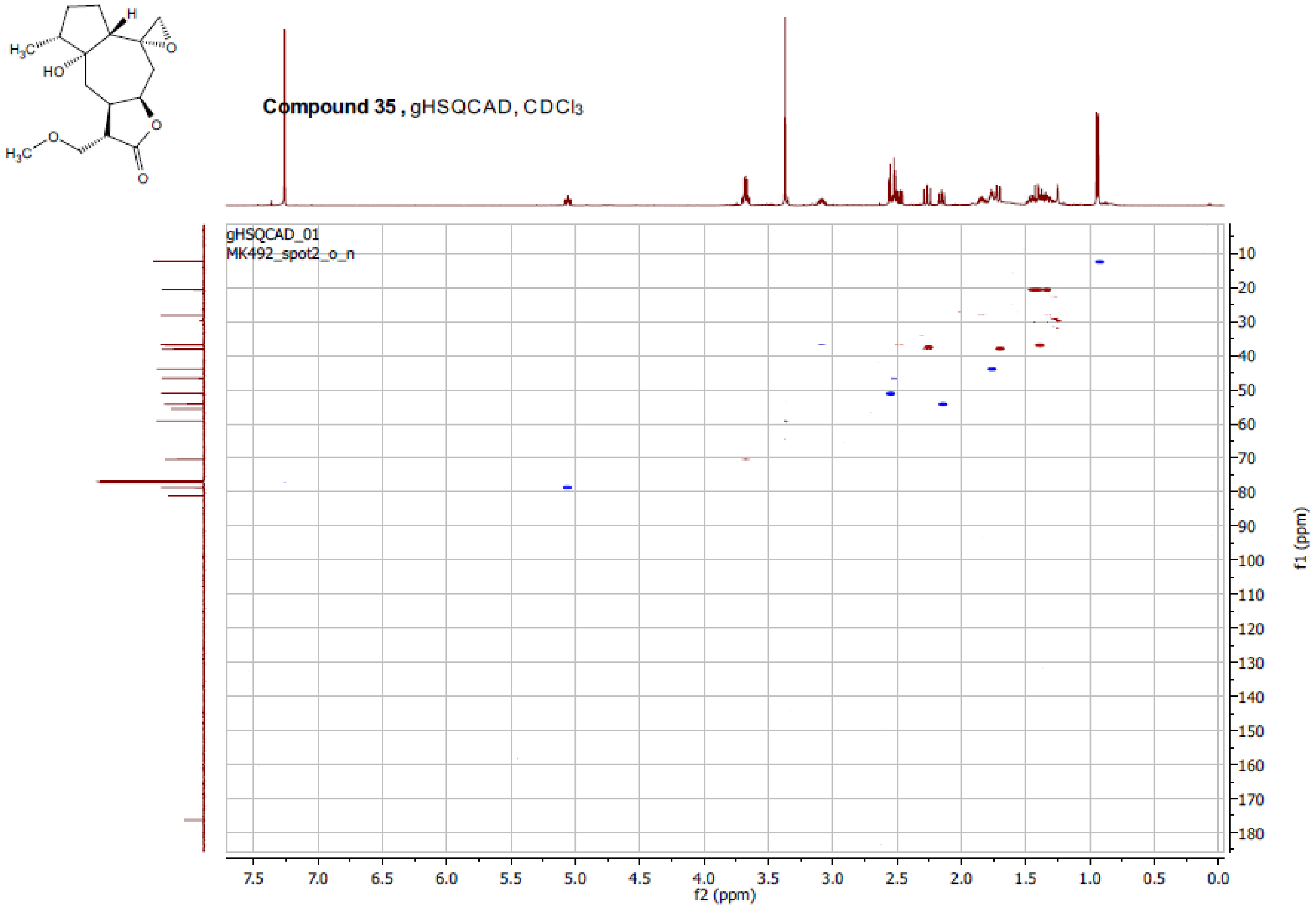
Compound 35 , gHSQCAD, CDCl3
gHSQCAD_01
MK492_spot2_o_n
f2 (ppm)
f1 (ppm)

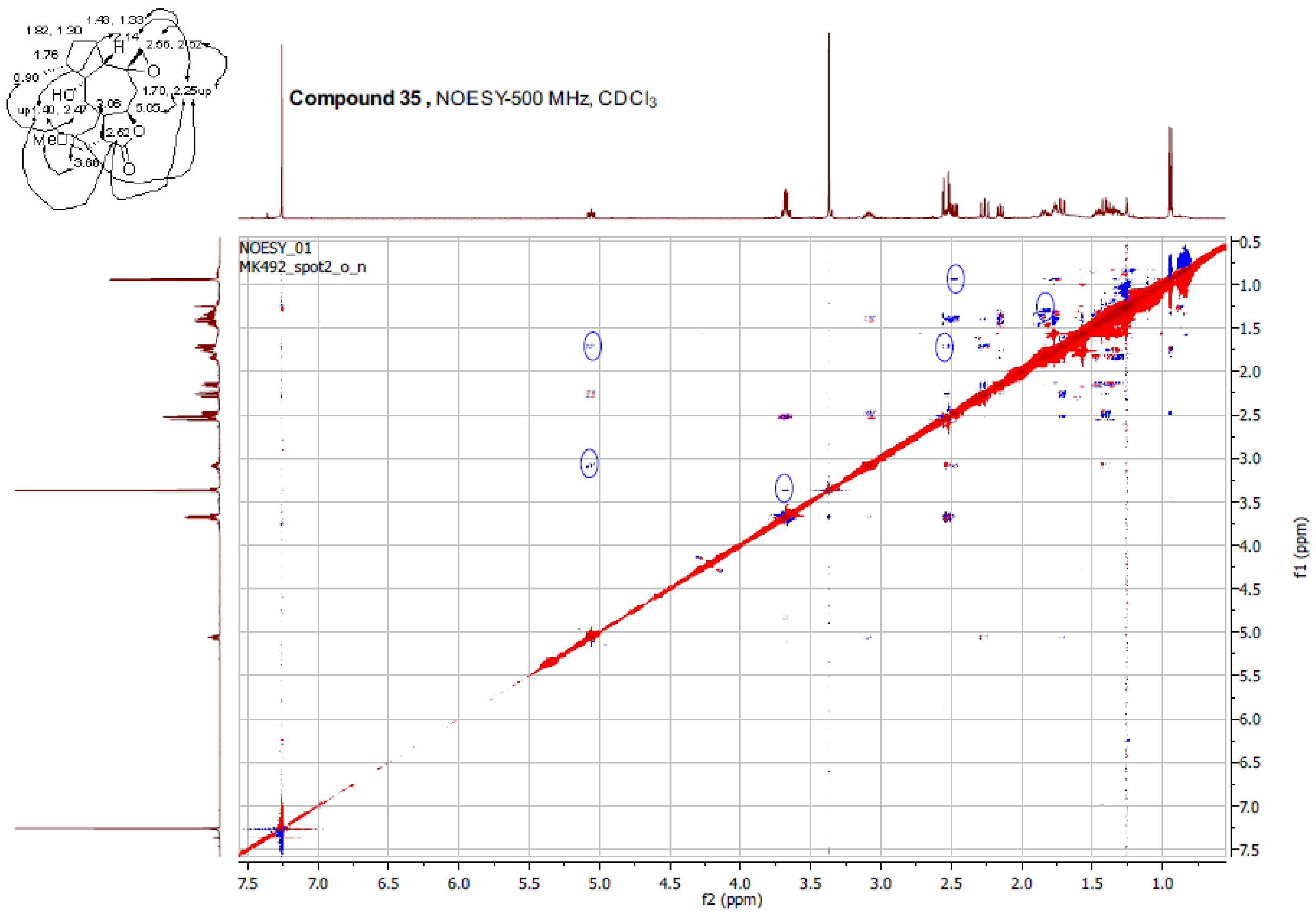
Compound 35 , NOESY-500 MHz, CDCl3
NOESY_01
MK492_spot2_o_n
f2 (ppm)
f1 (ppm)

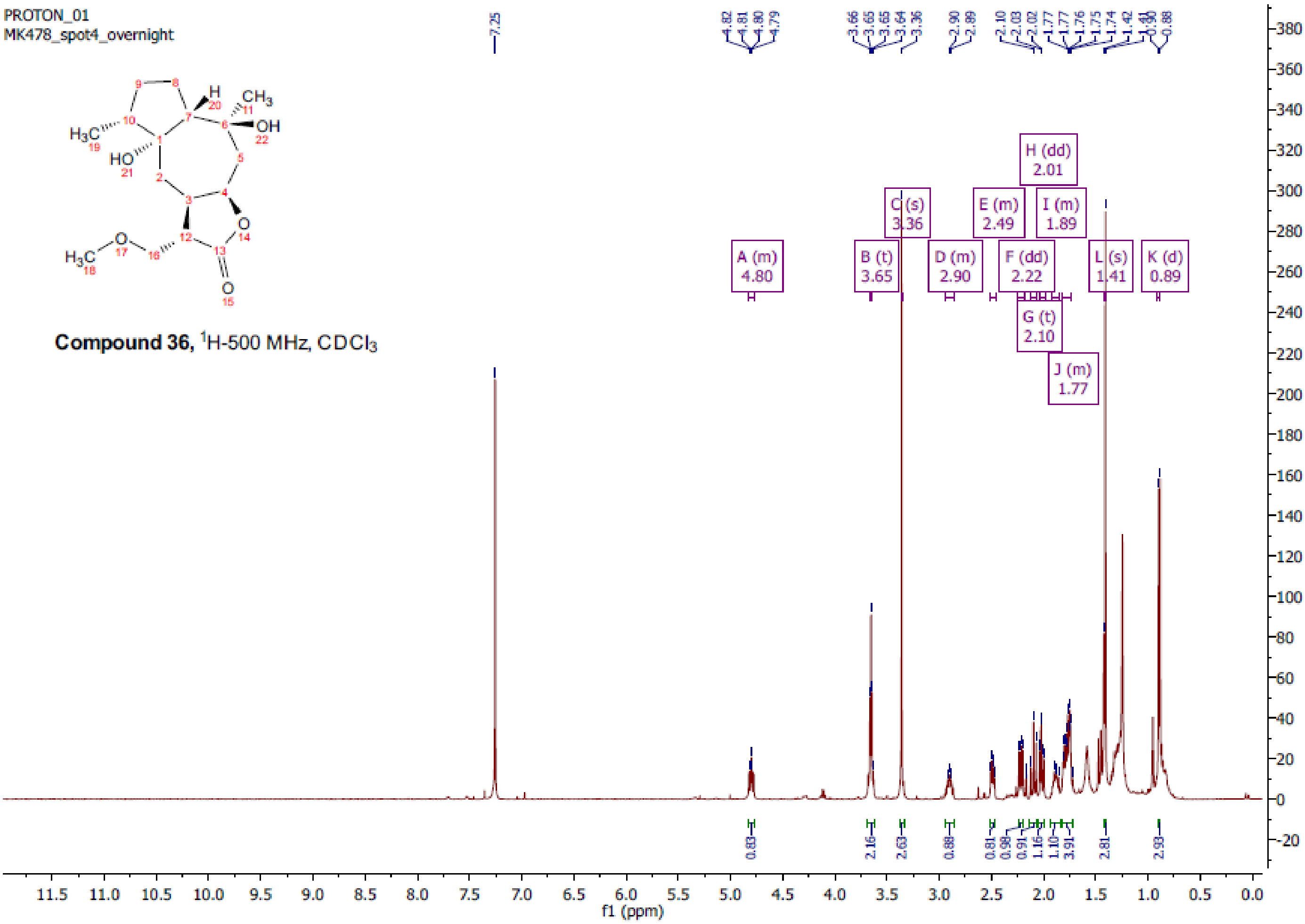

Compound 36, ^{1}H-500 MHz, $CDCl_3$

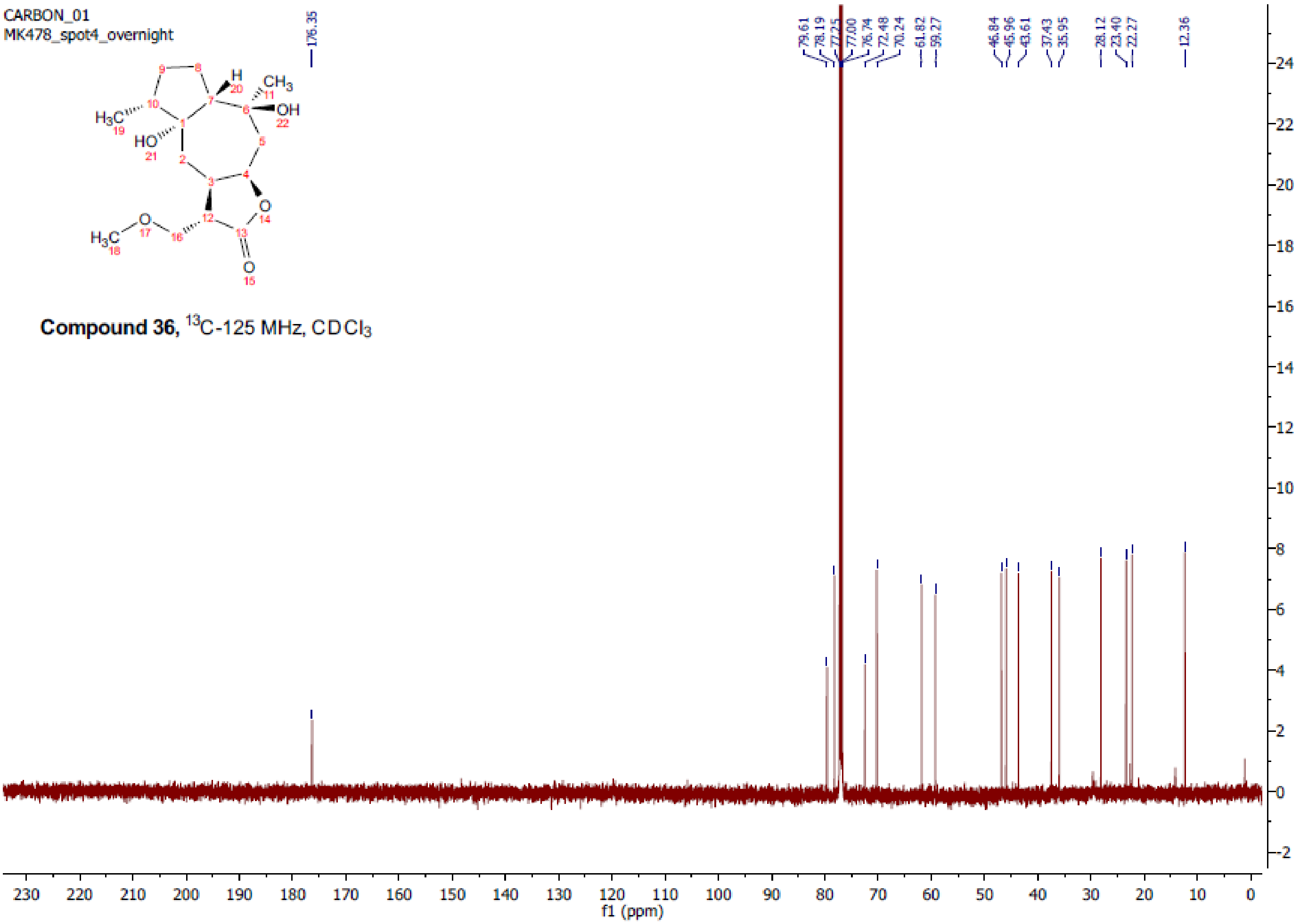

Compound 36, ^{13}C-125 MHz, $CDCl_3$

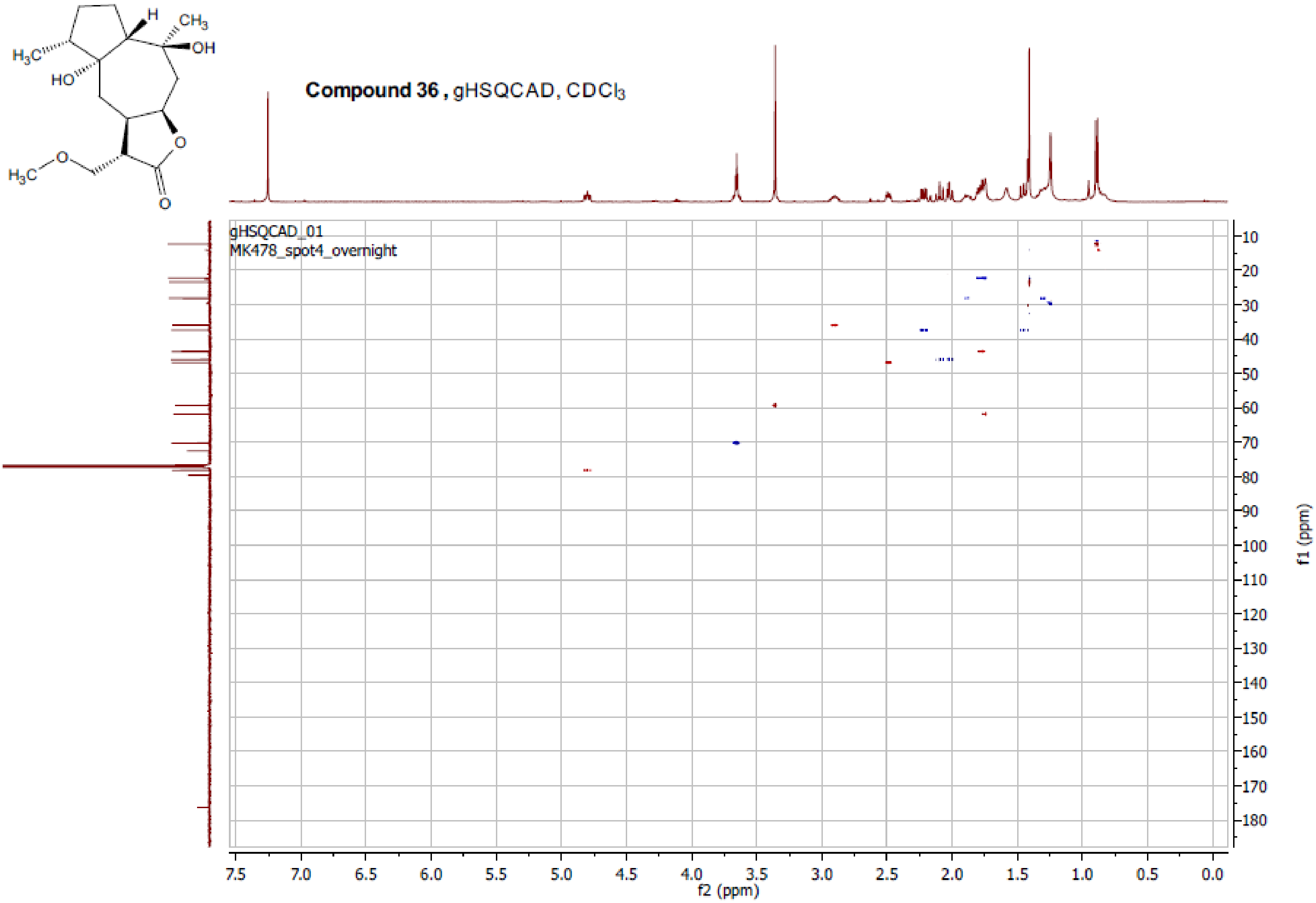

H
CH_3
OH
H_3C
HO
O
Compound 36 , gHSQCAD, $CDCl_3$
gHSQCAD_01
MK478_spot4_overnight
f2 (ppm)
f1 (ppm)

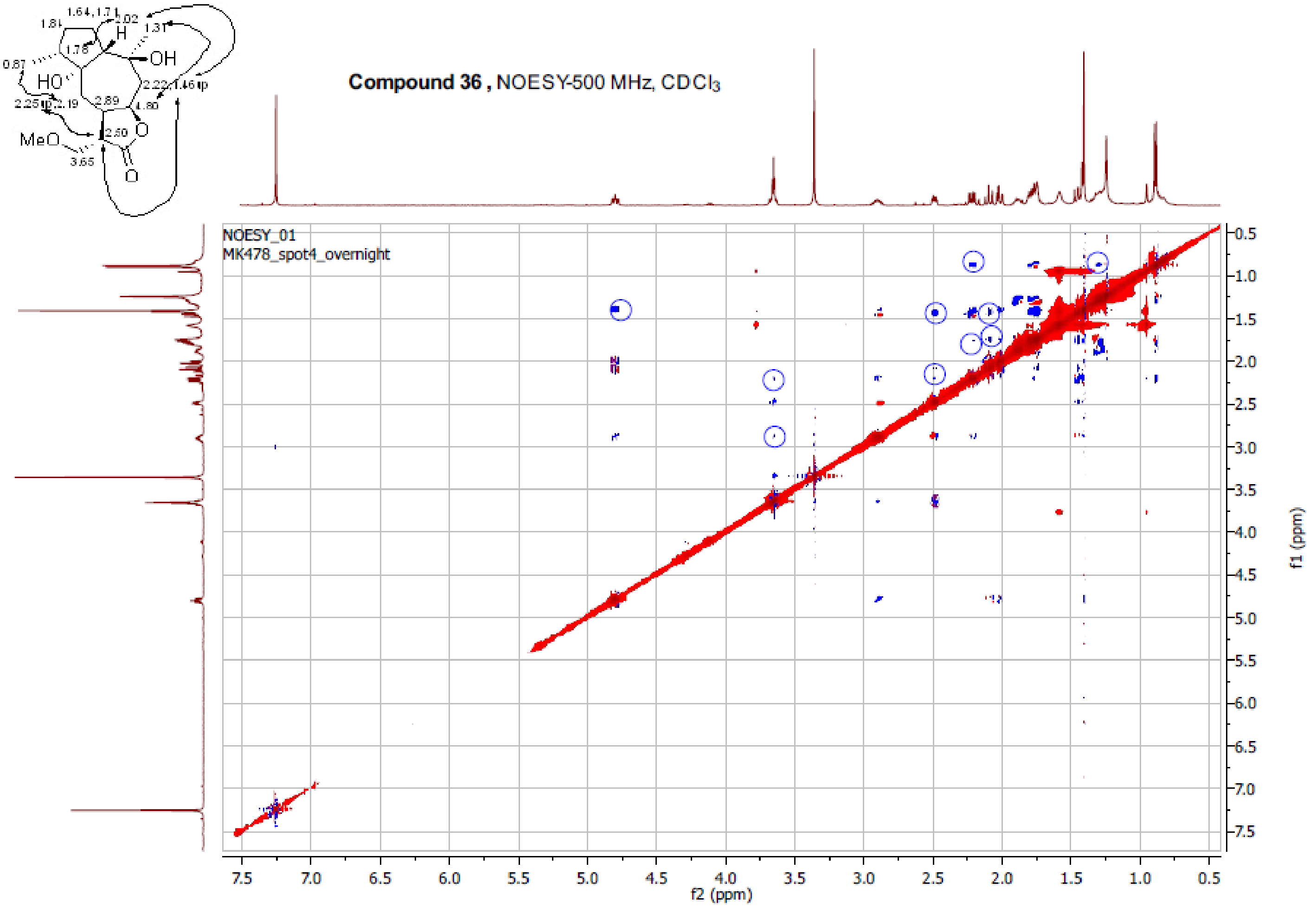

OH
HO
MeO
O
Compound 36 , NOESY-500 MHz, $CDCl_3$
NOESY_01
MK478_spot4_overnight
f2 (ppm)
f1 (ppm)

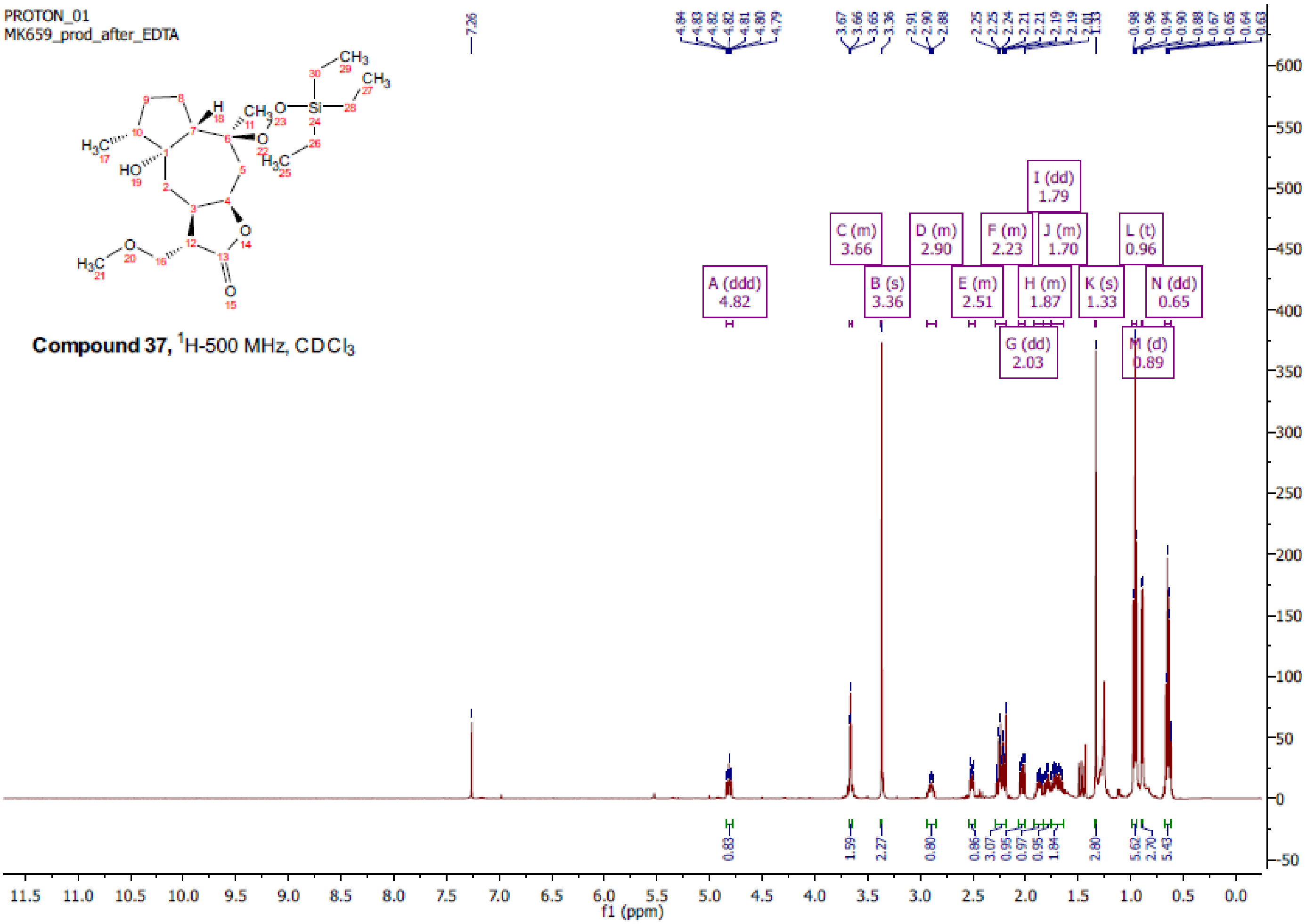
PROTON_01
MK659_prod_after_EDTA
Compound 37, 1H-500 MHz, CDCl3
A (ddd) 4.82
B (s) 3.36
C (m) 3.66
D (m) 2.90
E (m) 2.51
F (m) 2.23
G (dd) 2.03
H (m) 1.87
I (dd) 1.79
J (m) 1.70
K (s) 1.33
L (t) 0.96
M (d) 0.89
N (dd) 0.65
f1 (ppm)

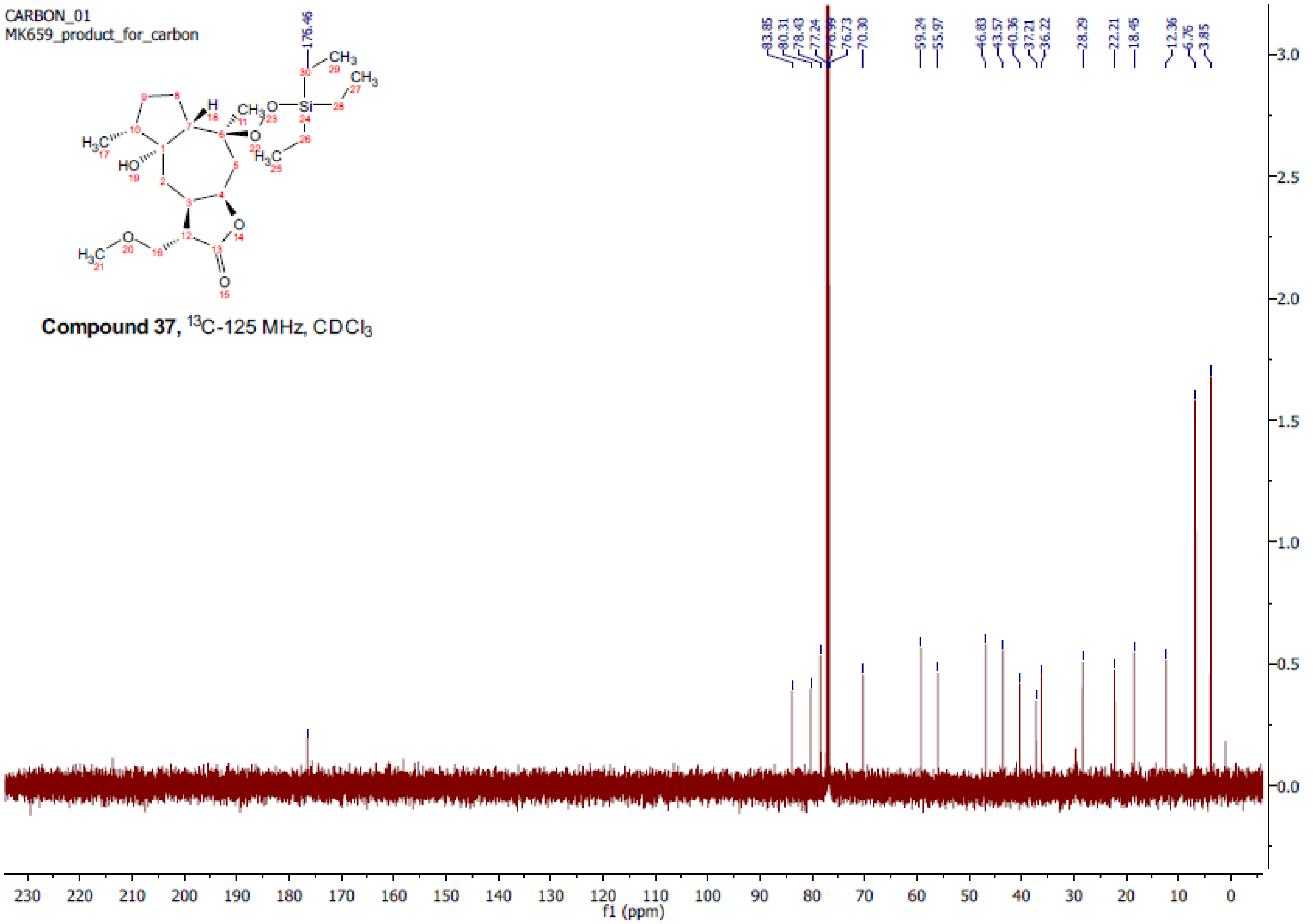
CARBON_01
MK659_product_for_carbon
Compound 37, 13C-125 MHz, CDCl3
176.46
83.85
80.31
78.43
77.24
76.99
76.73
70.30
59.24
55.97
46.83
43.57
40.36
37.21
36.22
28.29
22.21
18.45
12.36
6.76
3.85
f1 (ppm)

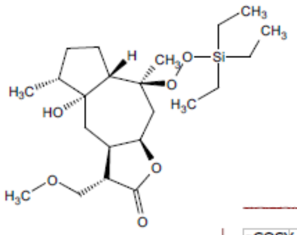

Compound 37, gCOSY-500 MHz, CDCl3

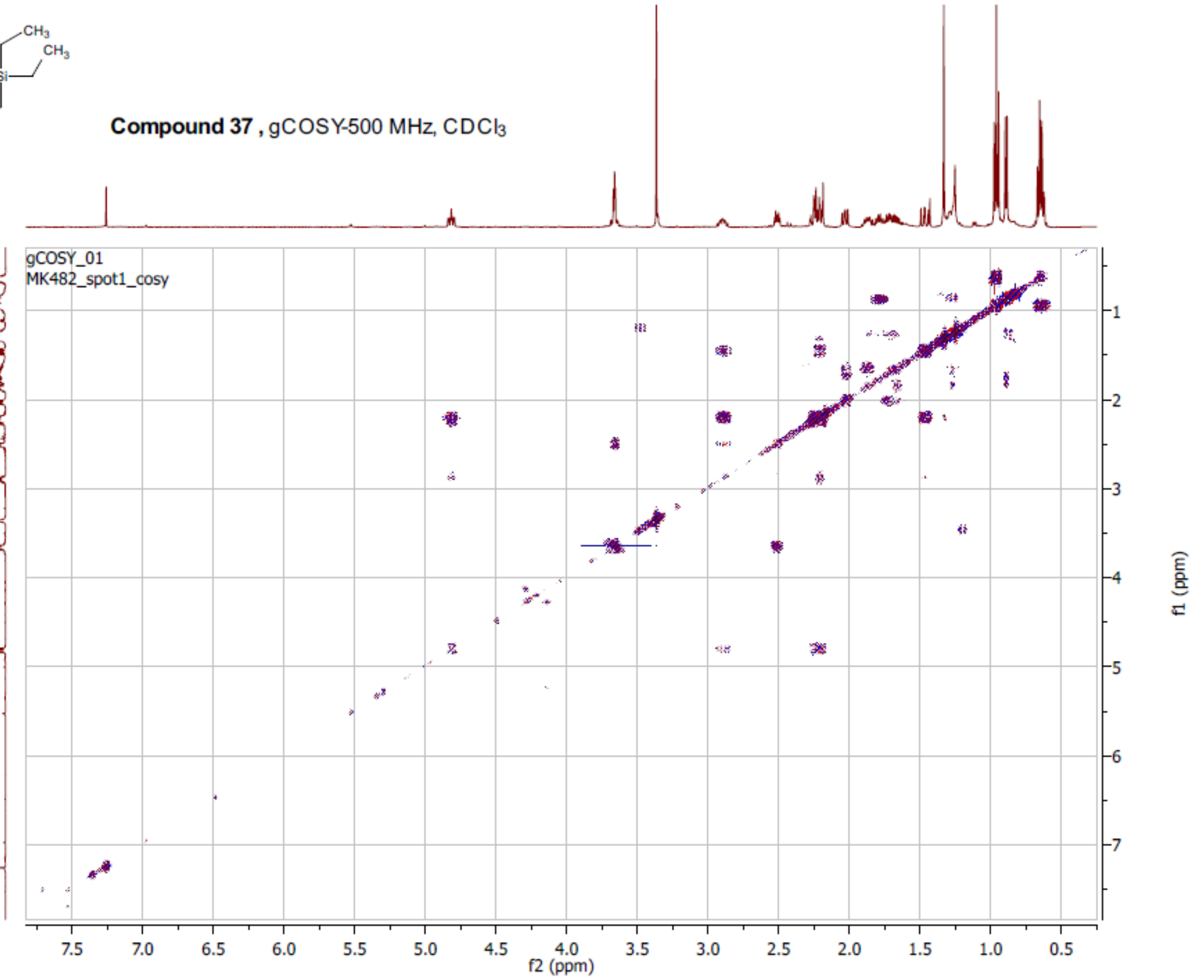

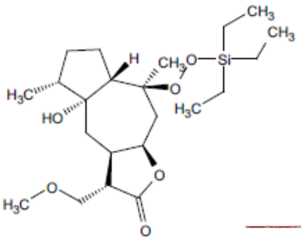

Compound 37, gHSQCAD, CDCl3

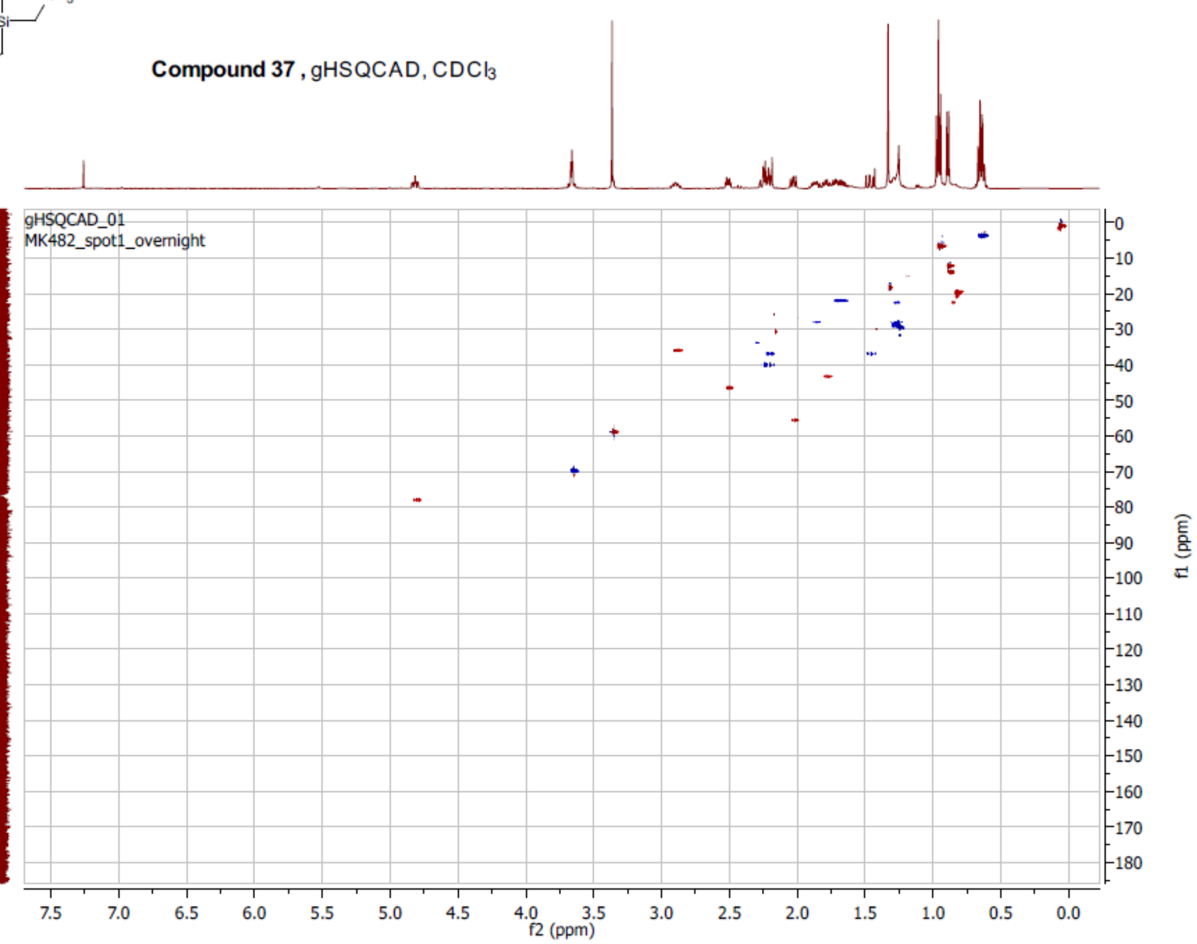

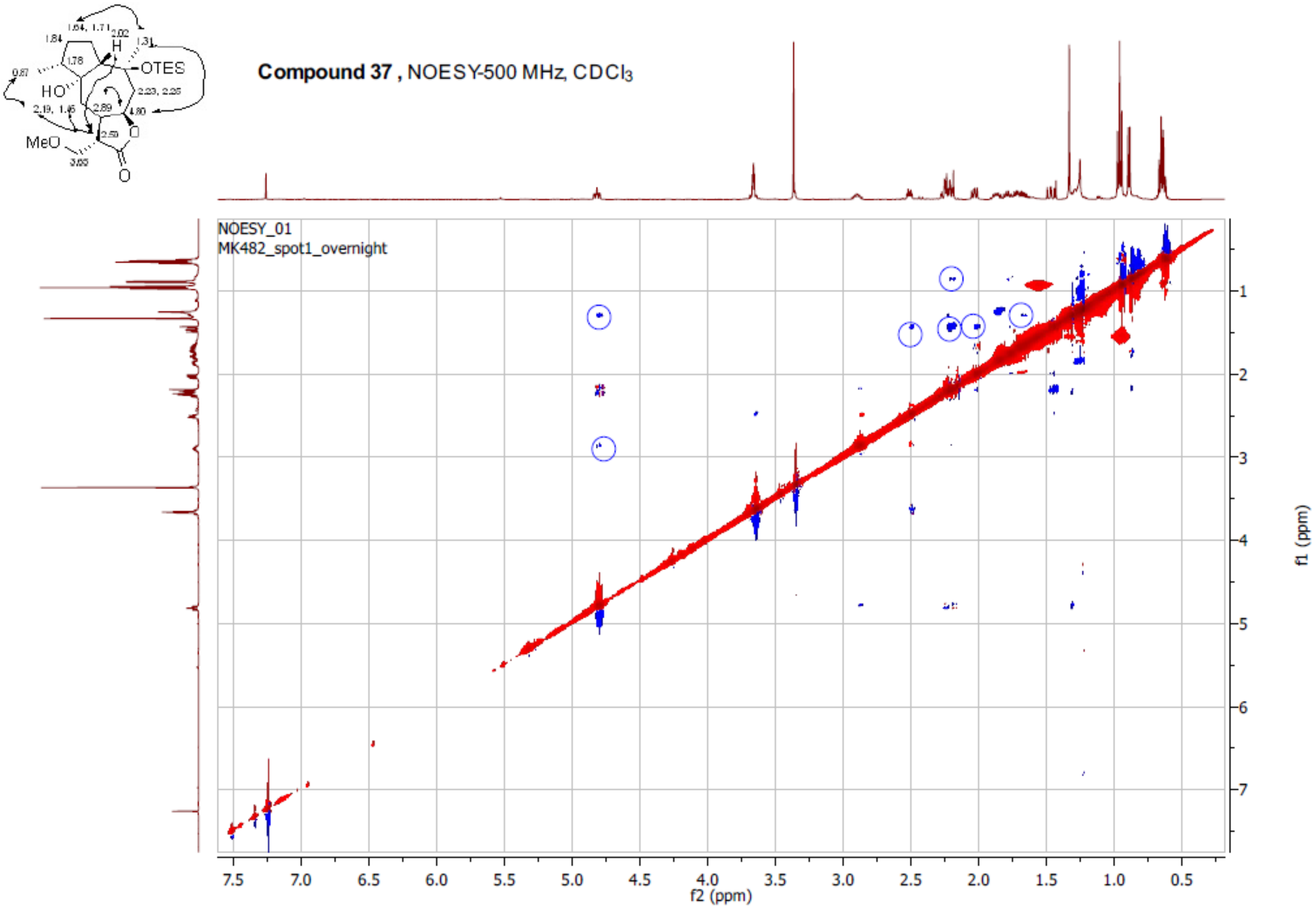
Compound 37 , NOESY-500 MHz, CDCl3
NOESY_01
MK482_spot1_overnight
OTES
HO
MeO
f2 (ppm)
f1 (ppm)

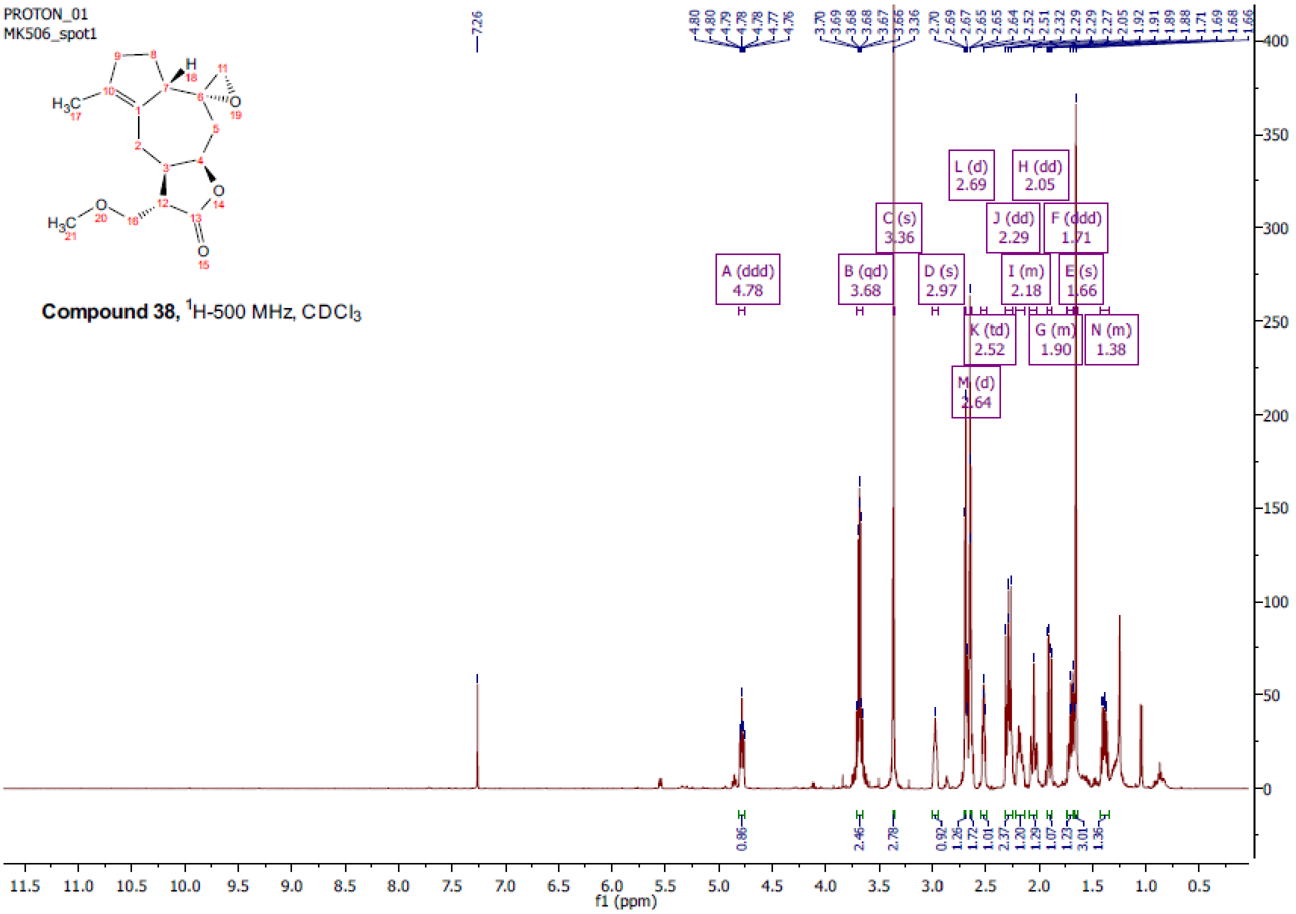
PROTON_01
MK506_spot1
Compound 38, 1H-500 MHz, CDCl3
A (ddd) 4.78
B (qd) 3.68
C (s) 3.36
D (s) 2.97
E (s) 1.66
F (ddd) 1.71
G (m) 1.90
H (dd) 2.05
I (m) 2.18
J (dd) 2.29
K (td) 2.52
L (d) 2.69
M (d) 2.64
N (m) 1.38
f1 (ppm)

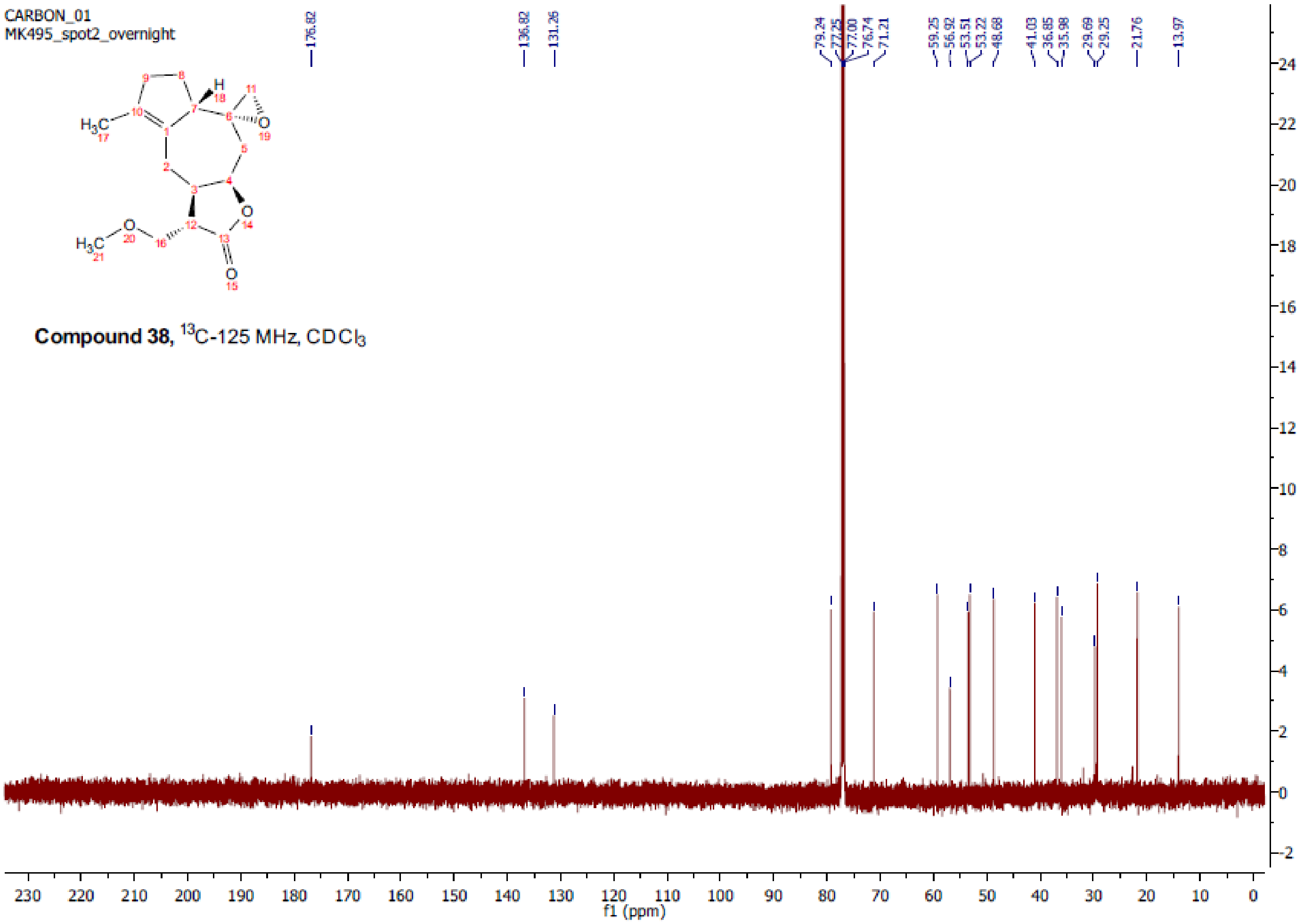

Compound 38, ^{13}C-125 MHz, CDCl$_3$

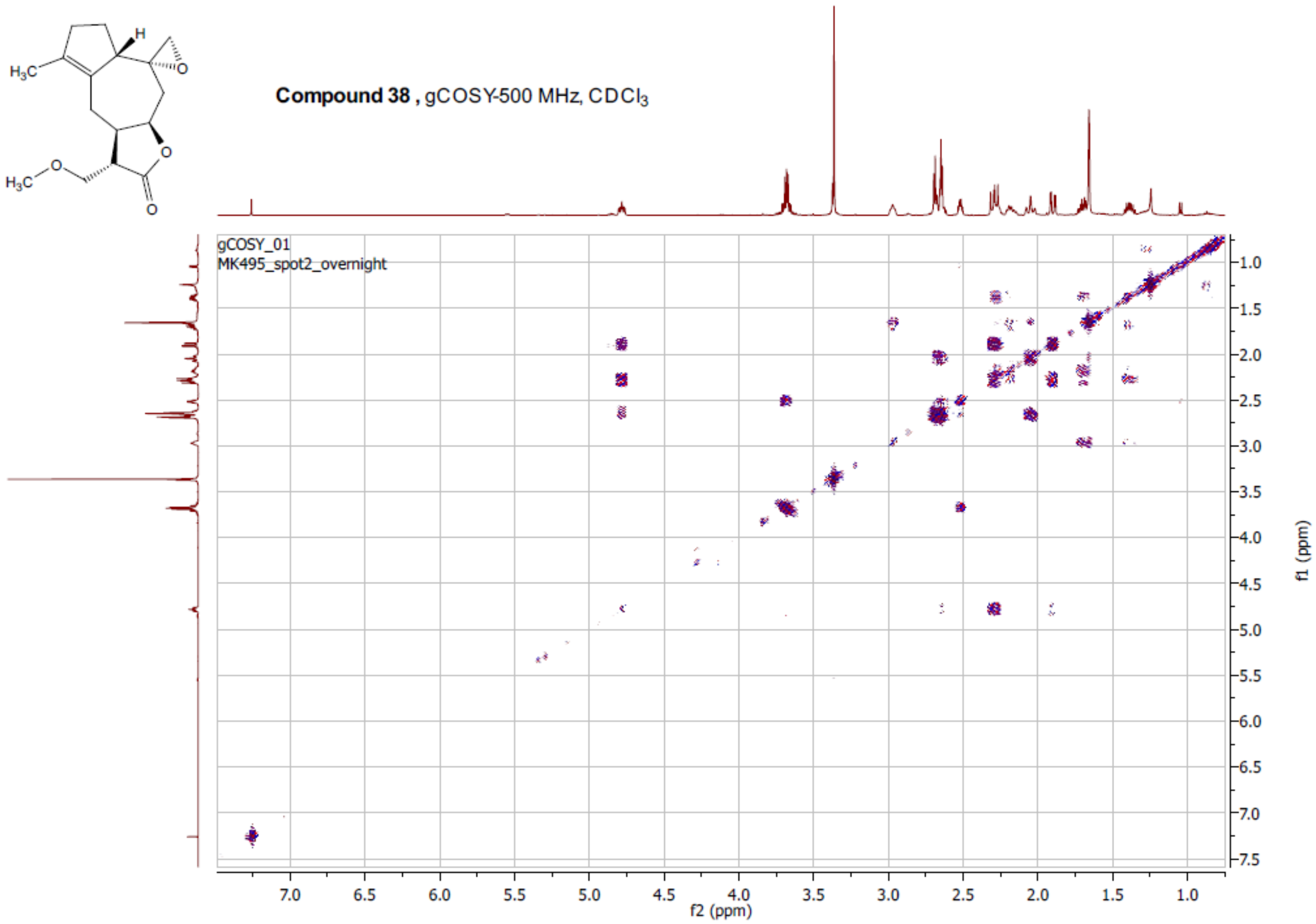

Compound 38 , gCOSY-500 MHz, CDCl$_3$

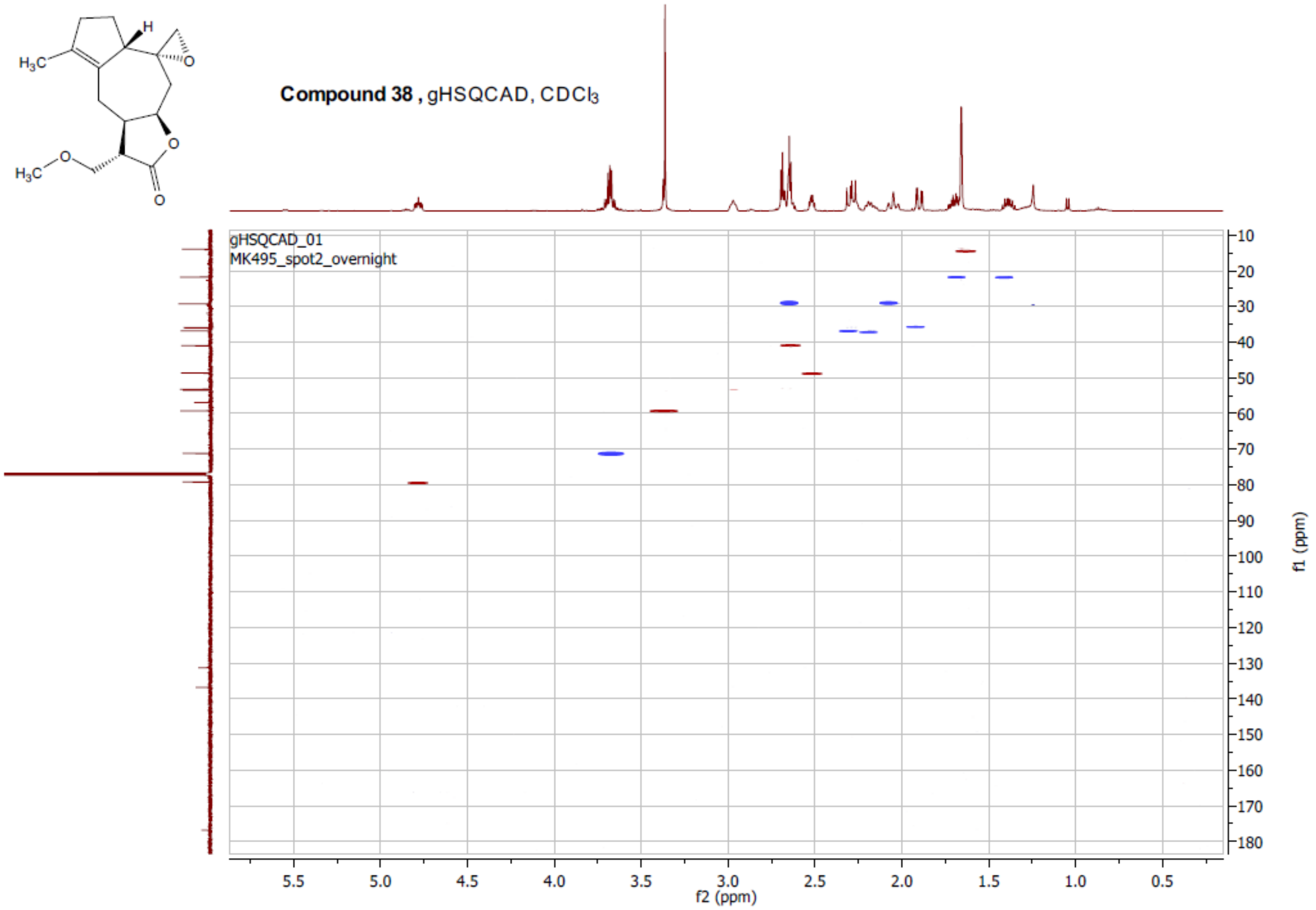
Compound 38 , gHSQCAD, CDCl3
gHSQCAD_01
MK495_spot2_overnight
f2 (ppm)
f1 (ppm)

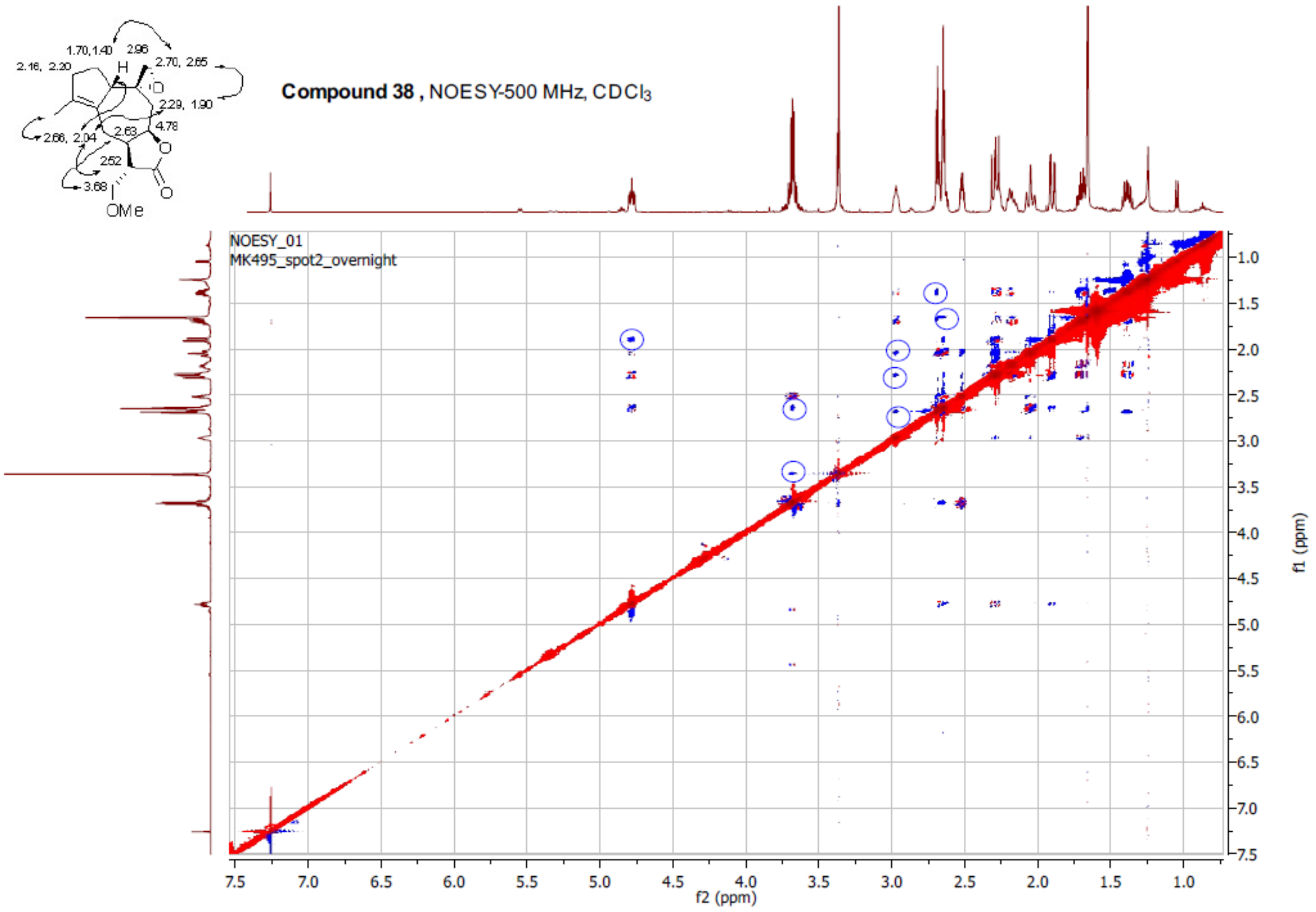
Compound 38 , NOESY-500 MHz, CDCl3
NOESY_01
MK495_spot2_overnight
f2 (ppm)
f1 (ppm)

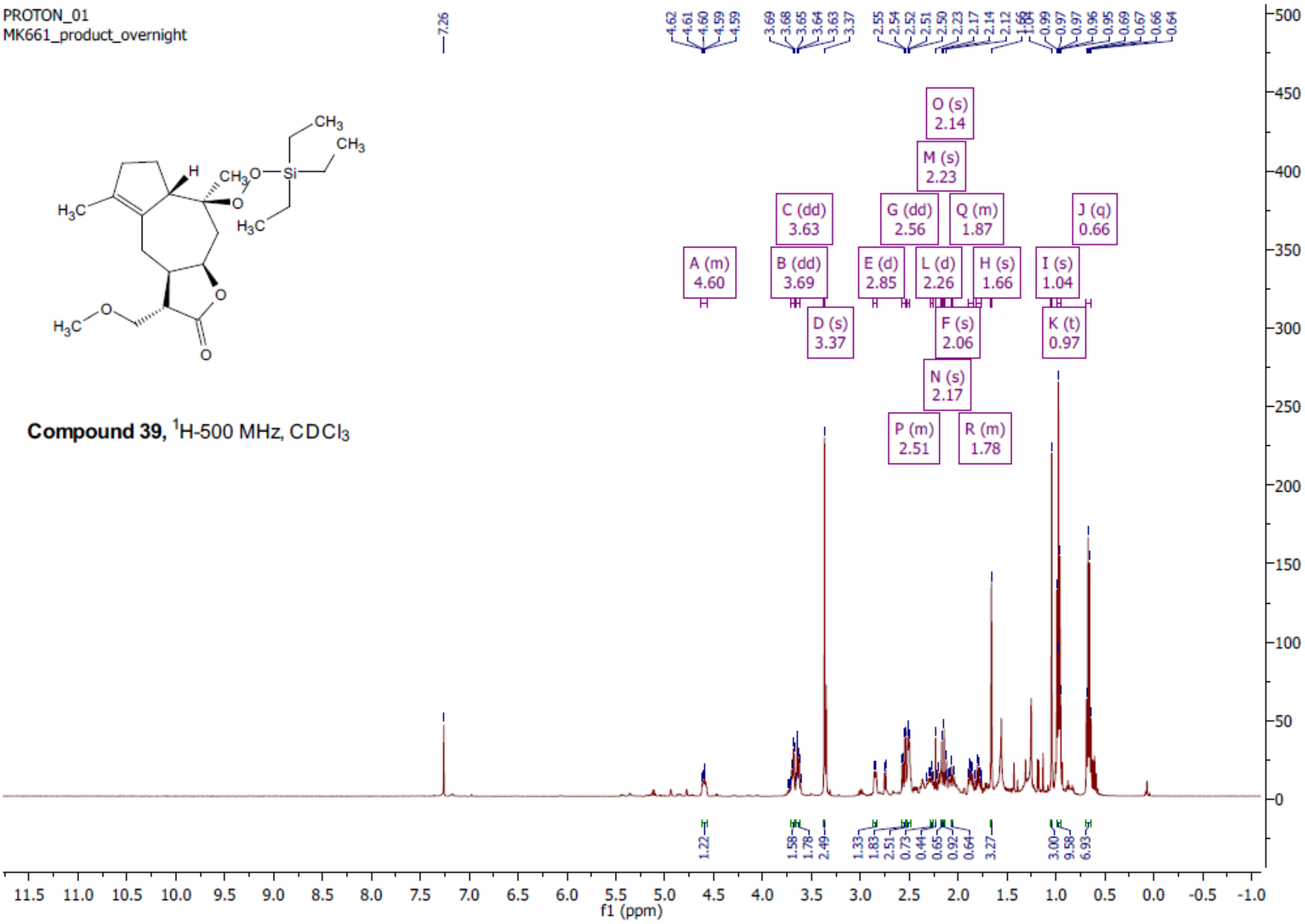

Compound 39, ^{1}H-500 MHz, $CDCl_3$

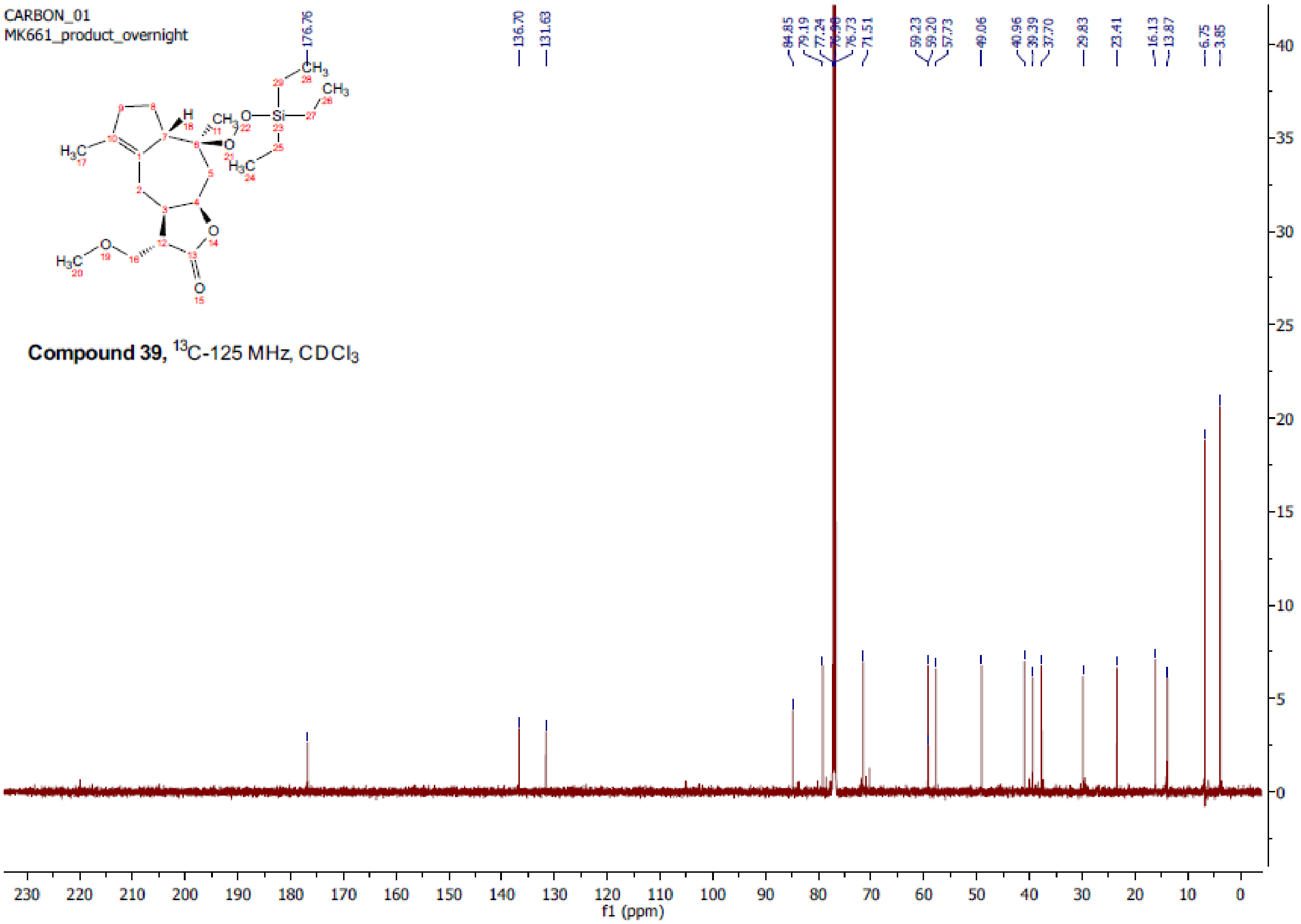

Compound 39, ^{13}C-125 MHz, $CDCl_3$

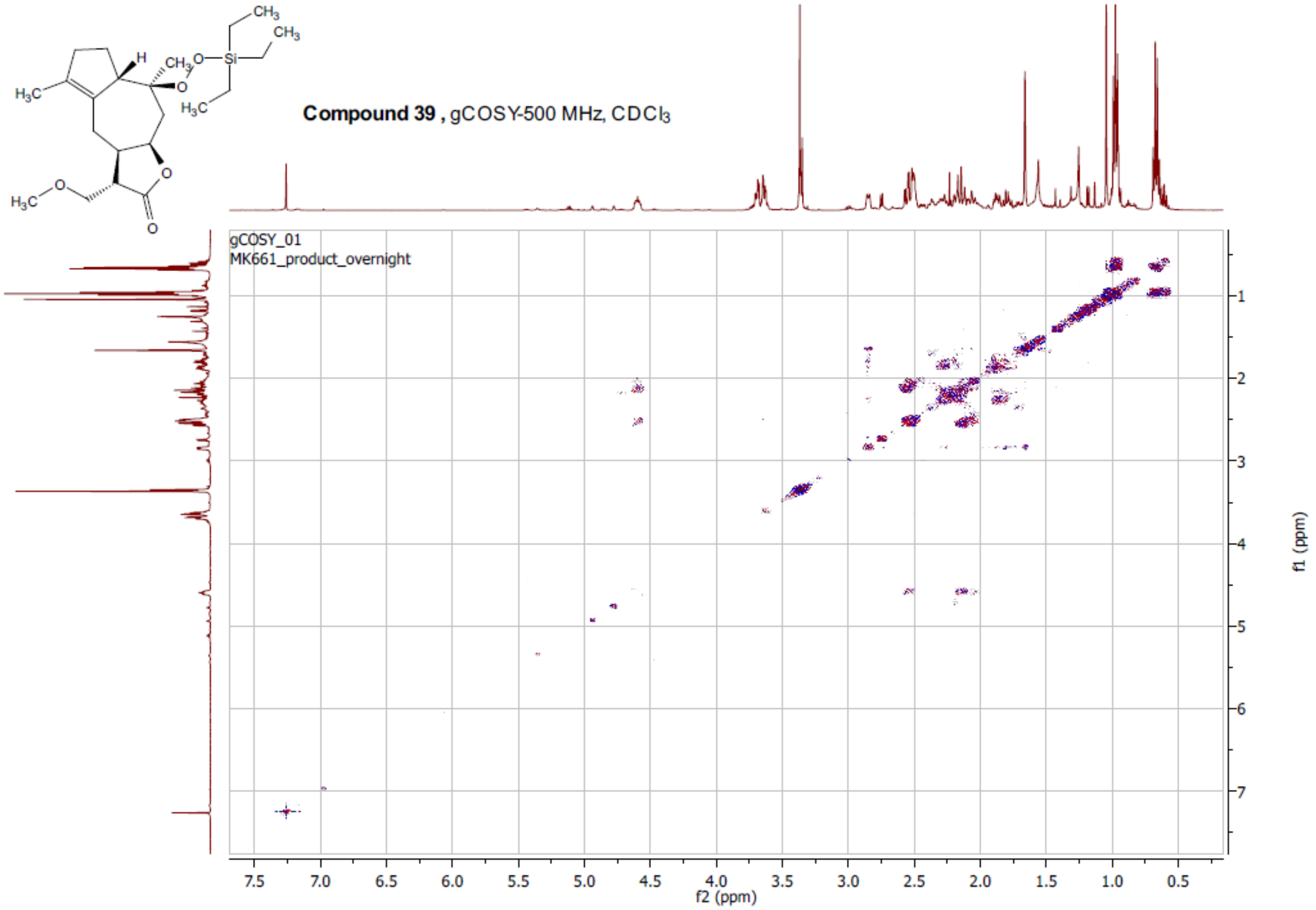
Compound 39 , gCOSY-500 MHz, CDCl3
gCOSY_01
MK661_product_overnight
f2 (ppm)
f1 (ppm)

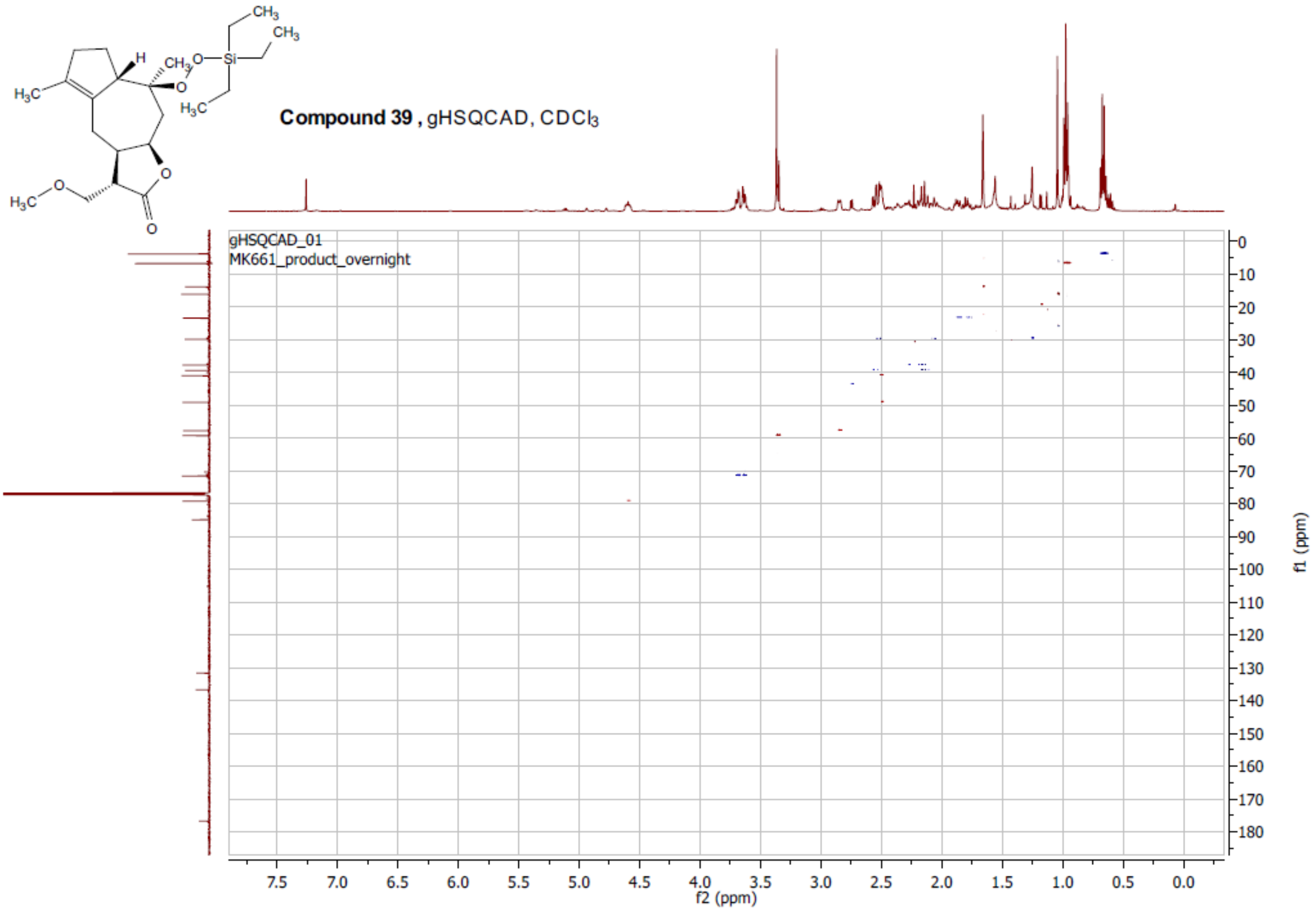
Compound 39 , gHSQCAD, CDCl3
gHSQCAD_01
MK661_product_overnight
f2 (ppm)
f1 (ppm)

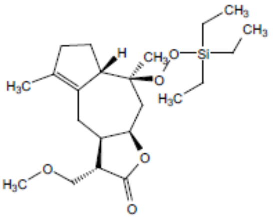

Compound 39, NOESY-500 MHz, CDCl$_3$

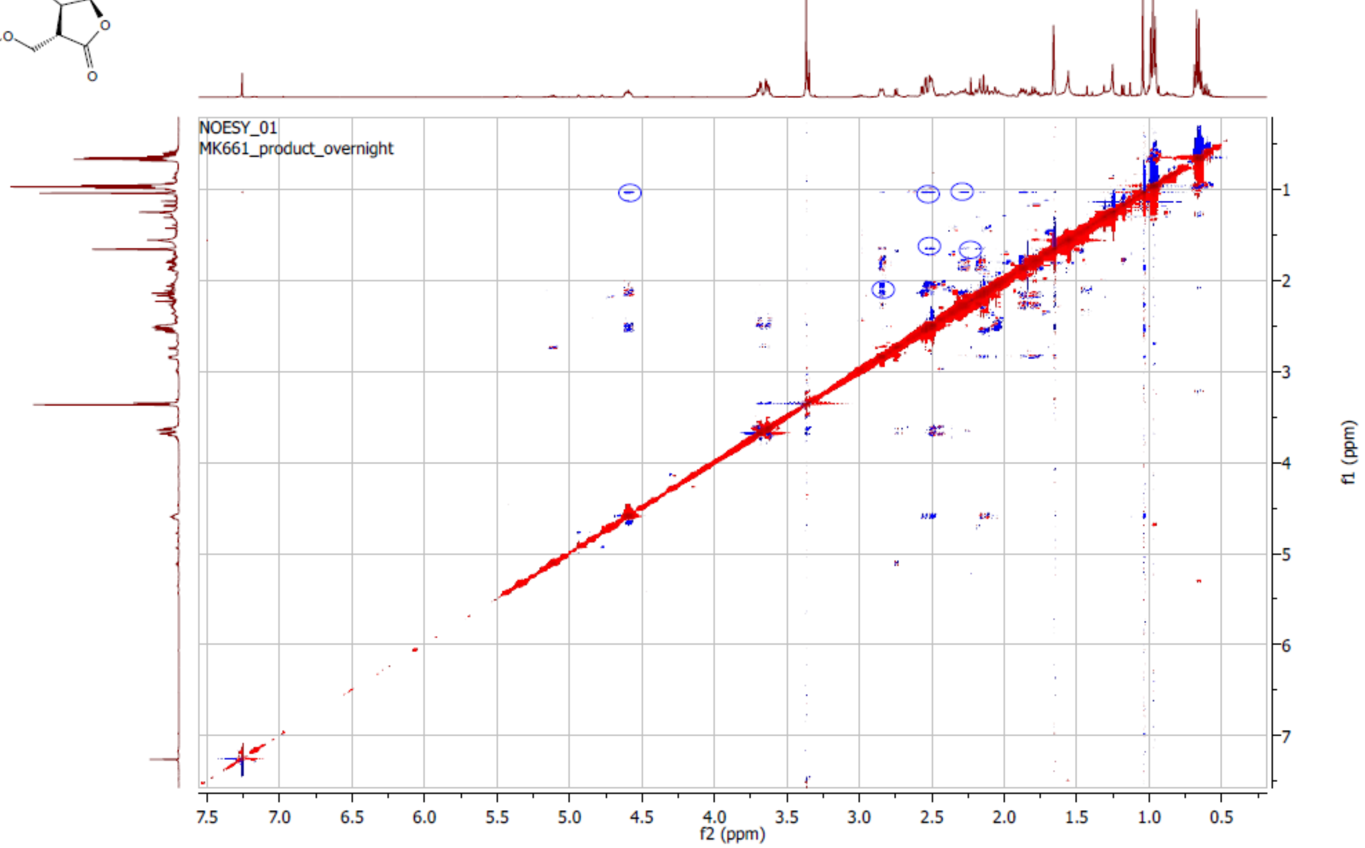

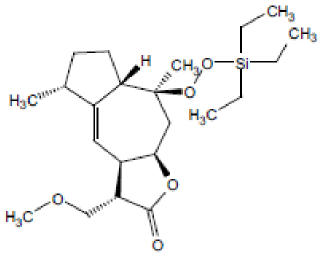

Compound 40, ^{1}H-500 MHz, CDCl$_3$

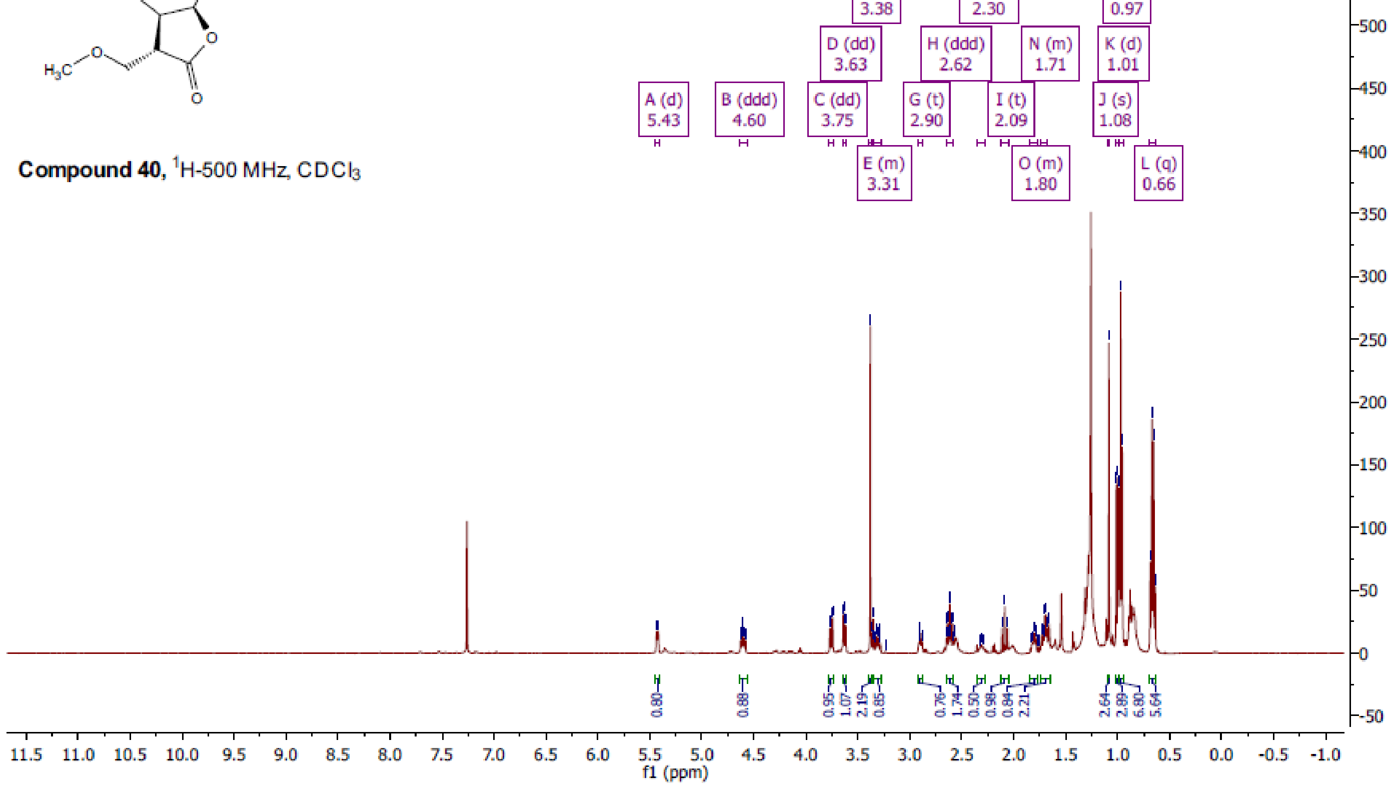

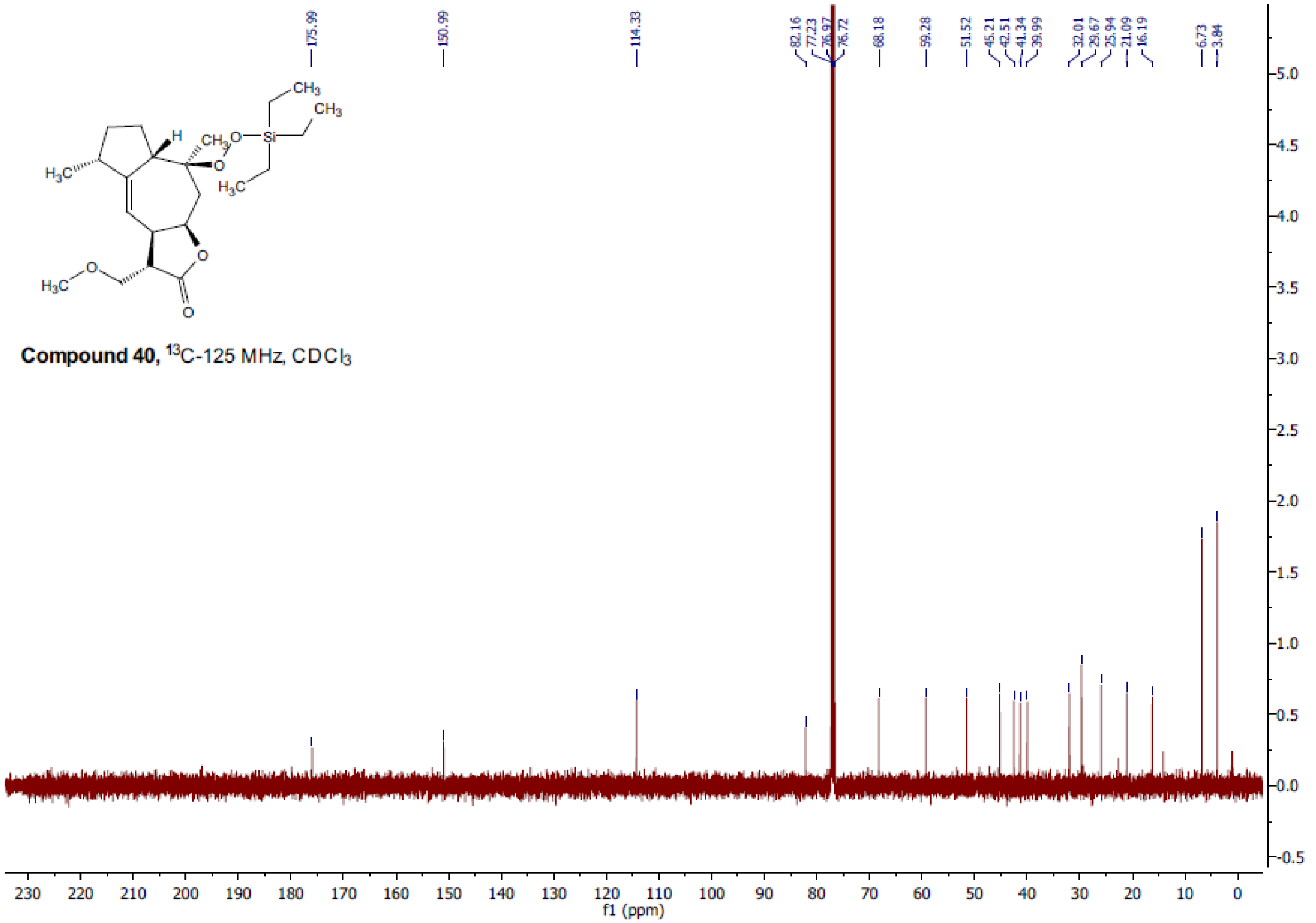
175.99
150.99
114.33
82.16
77.23
76.97
76.72
68.18
59.28
51.52
45.21
42.51
41.34
39.99
32.01
29.67
25.94
21.09
16.19
6.73
3.84
Compound 40, ^{13}C-125 MHz, $CDCl_3$
f1 (ppm)

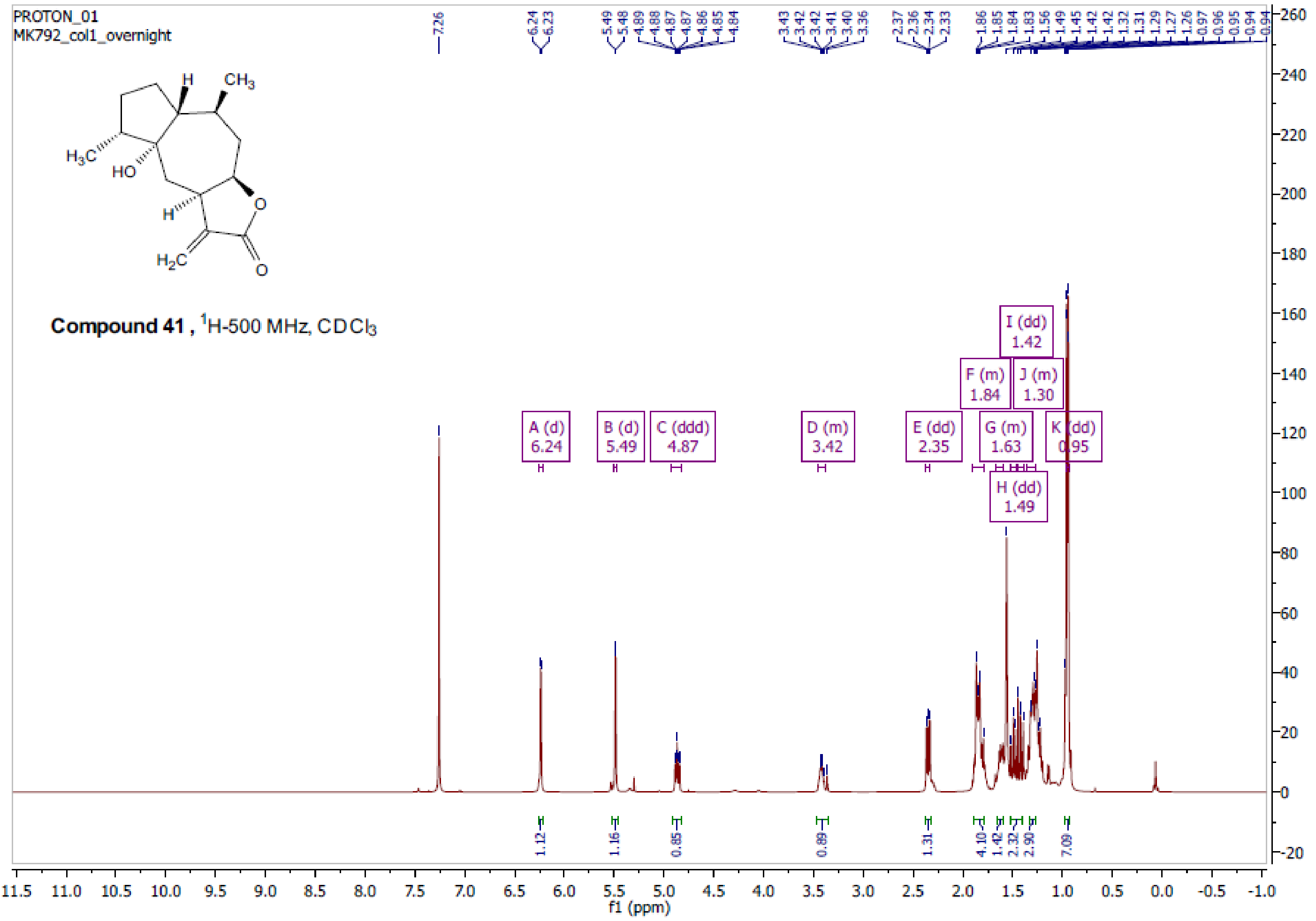
PROTON_01
MK792_col1_overnight
7.26
6.24
6.23
5.49
5.48
4.89
4.88
4.87
4.87
4.86
4.85
4.84
3.43
3.42
3.42
3.41
3.40
3.36
2.37
2.36
2.34
2.33
1.86
1.85
1.84
1.83
1.56
1.49
1.45
1.42
1.42
1.32
1.31
1.29
1.27
1.26
0.97
0.96
0.95
0.94
0.94
Compound 41 , ^{1}H-500 MHz, $CDCl_3$
A (d) 6.24
B (d) 5.49
C (ddd) 4.87
D (m) 3.42
E (dd) 2.35
F (m) 1.84
G (m) 1.63
H (dd) 1.49
I (dd) 1.42
J (m) 1.30
K (dd) 0.95
1.12
1.16
0.85
0.89
1.31
4.10
1.42
2.32
2.90
7.09
f1 (ppm)

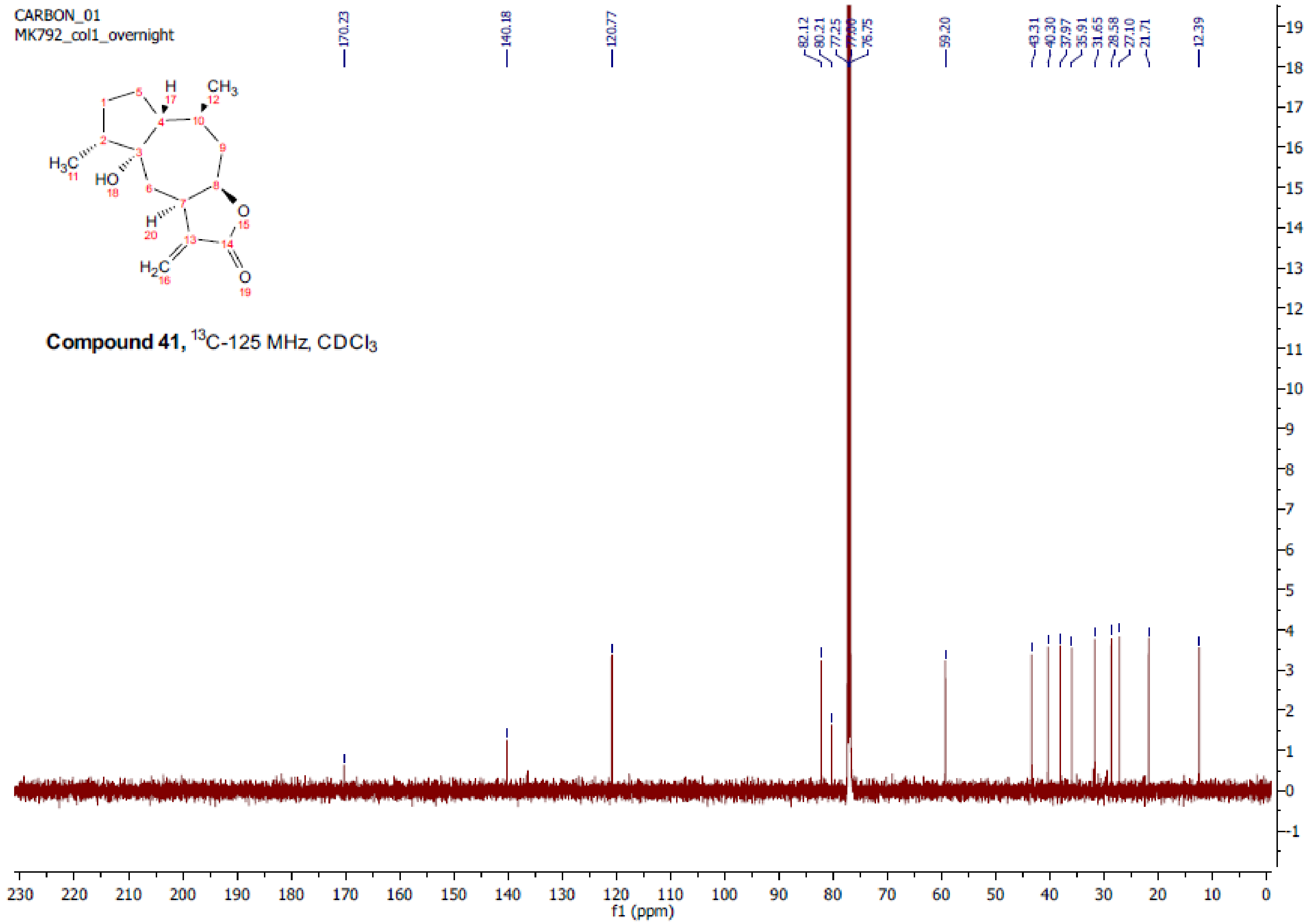
CARBON_01
MK792_col1_overnight
170.23
140.18
120.77
82.12
80.21
77.25
77.00
76.75
59.20
43.31
40.30
37.97
35.91
31.65
28.58
27.10
21.71
12.39
Compound 41, ^{13}C-125 MHz, CDCl$_3$
f1 (ppm)

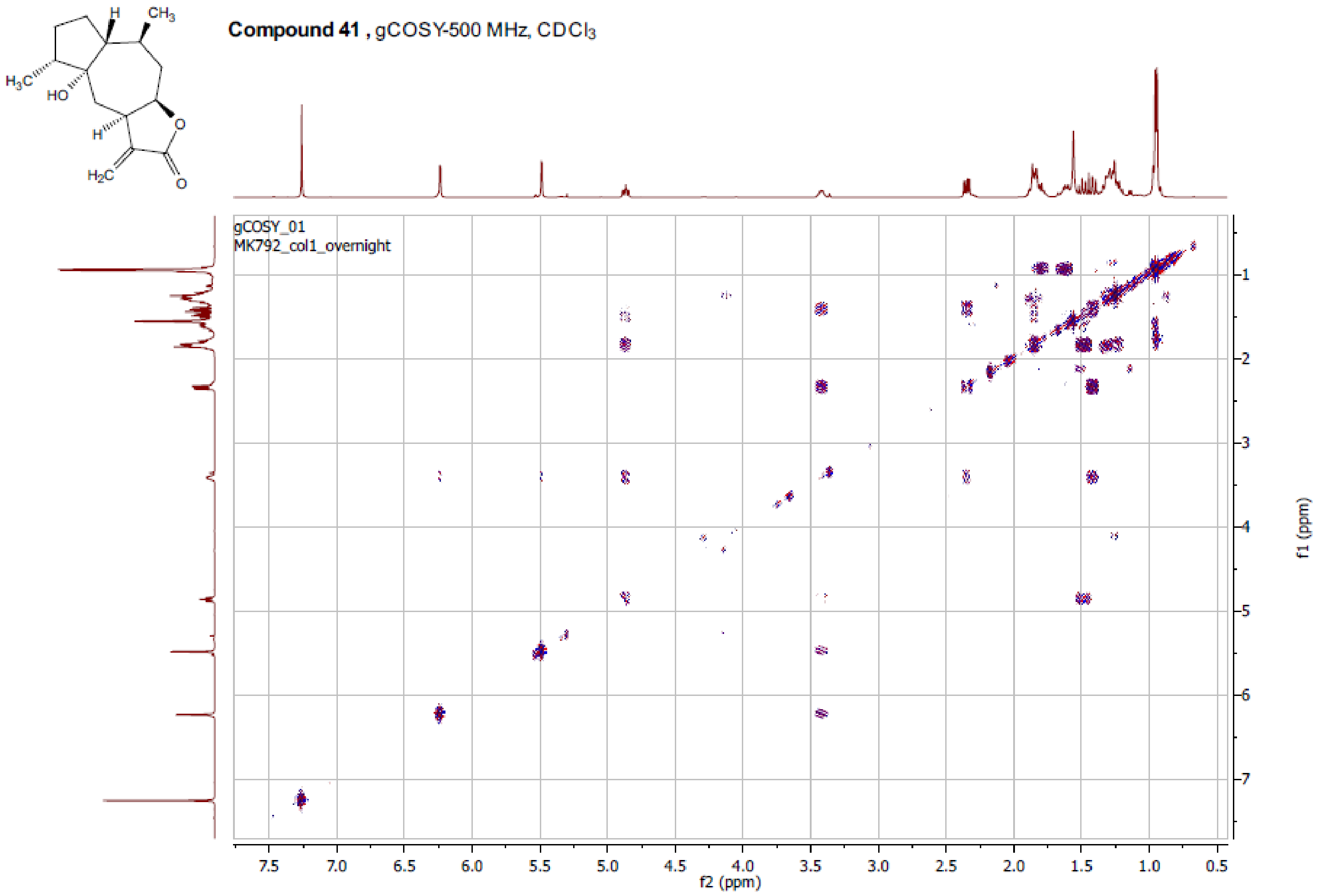
Compound 41 , gCOSY-500 MHz, CDCl$_3$
gCOSY_01
MK792_col1_overnight
f2 (ppm)
f1 (ppm)

Compound 41, gHSQCAD, $CDCl_3$

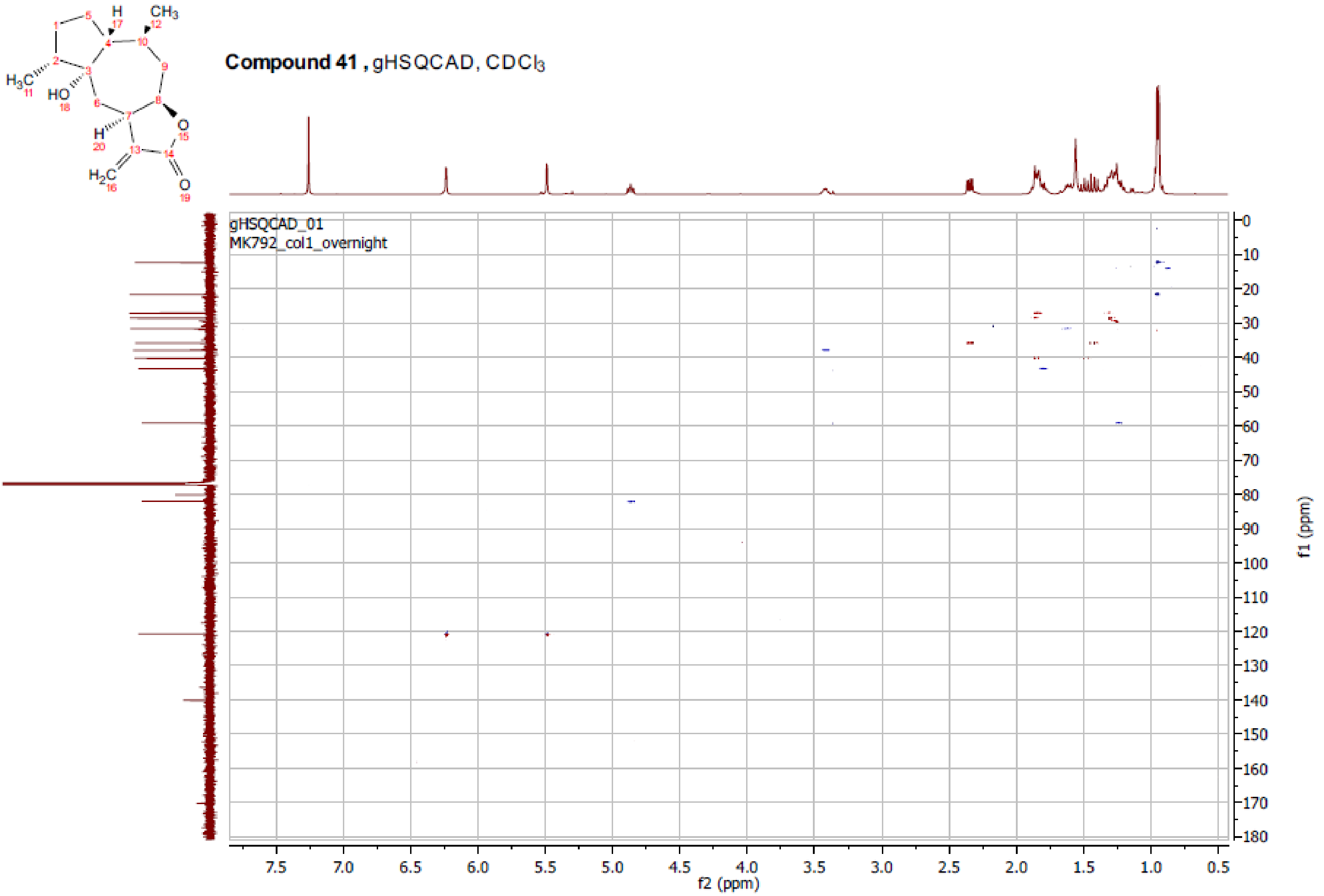

Compound 41, NOESY-500 MHz, $CDCl_3$

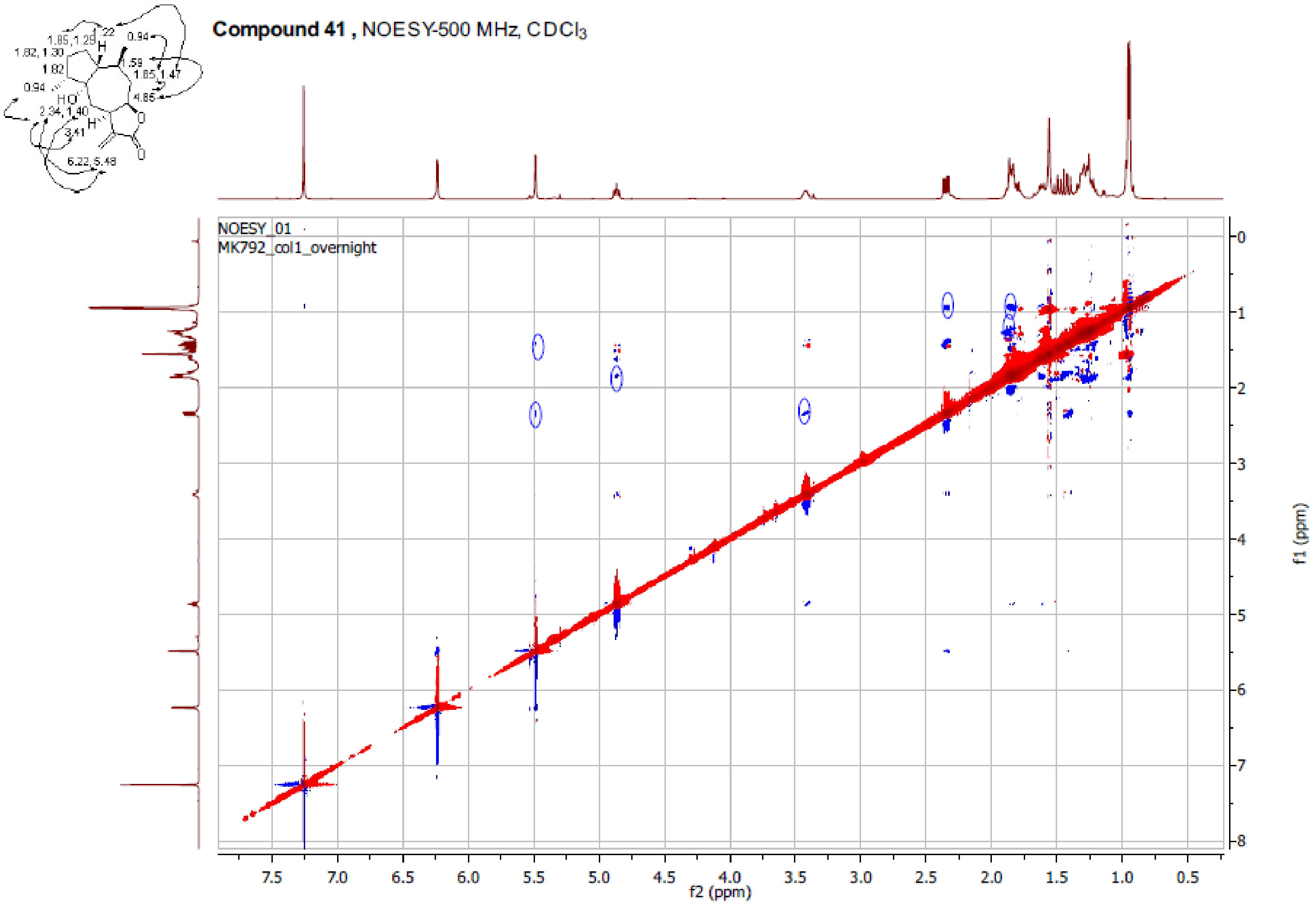

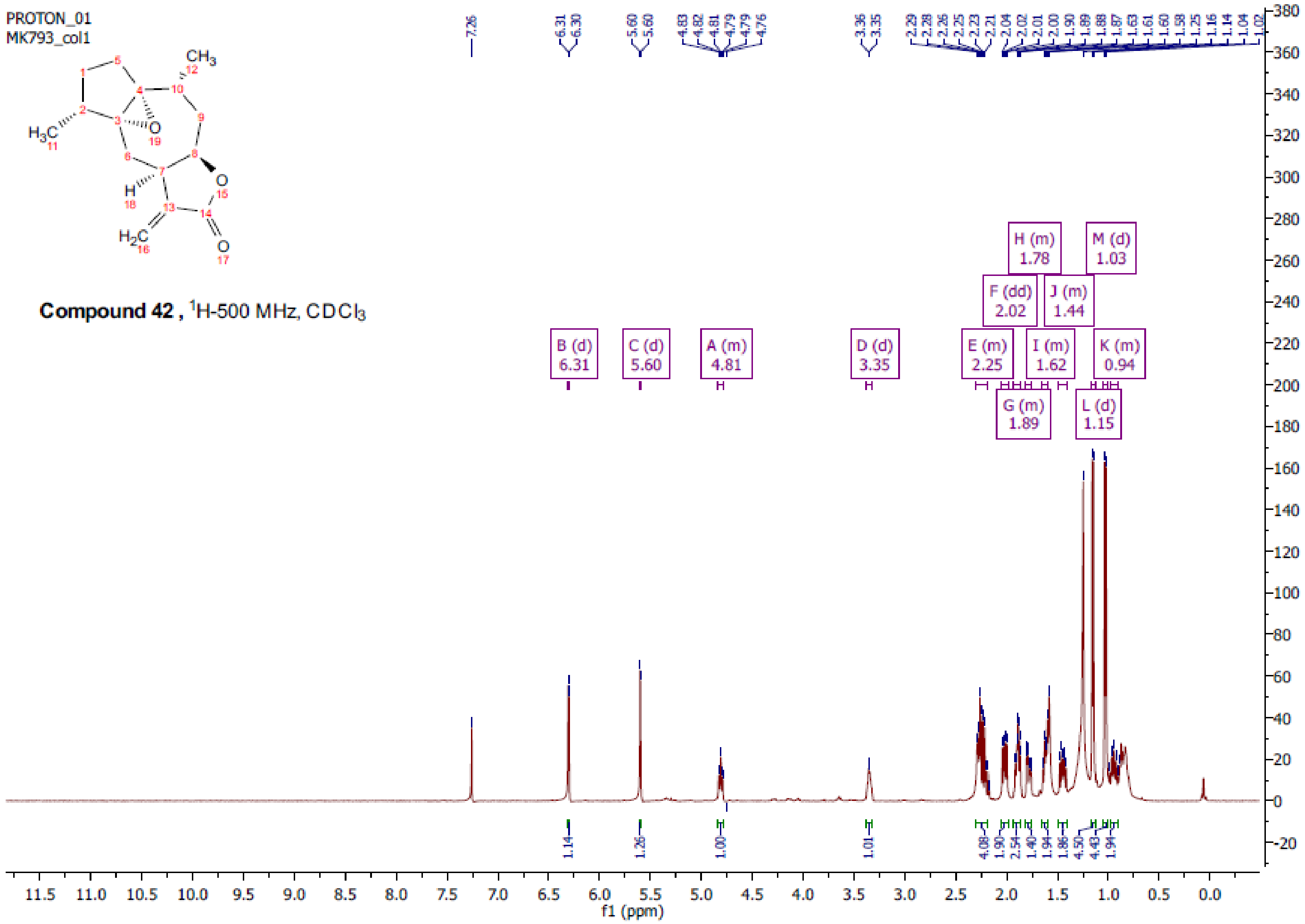
PROTON_01
MK793_col1
Compound 42 , 1H-500 MHz, CDCl3
7.26
6.31
6.30
5.60
5.60
4.83
4.82
4.81
4.79
4.79
4.76
3.36
3.35
B (d) 6.31
C (d) 5.60
A (m) 4.81
D (d) 3.35
E (m) 2.25
F (dd) 2.02
G (m) 1.89
H (m) 1.78
I (m) 1.62
J (m) 1.44
K (m) 0.94
L (d) 1.15
M (d) 1.03
f1 (ppm)

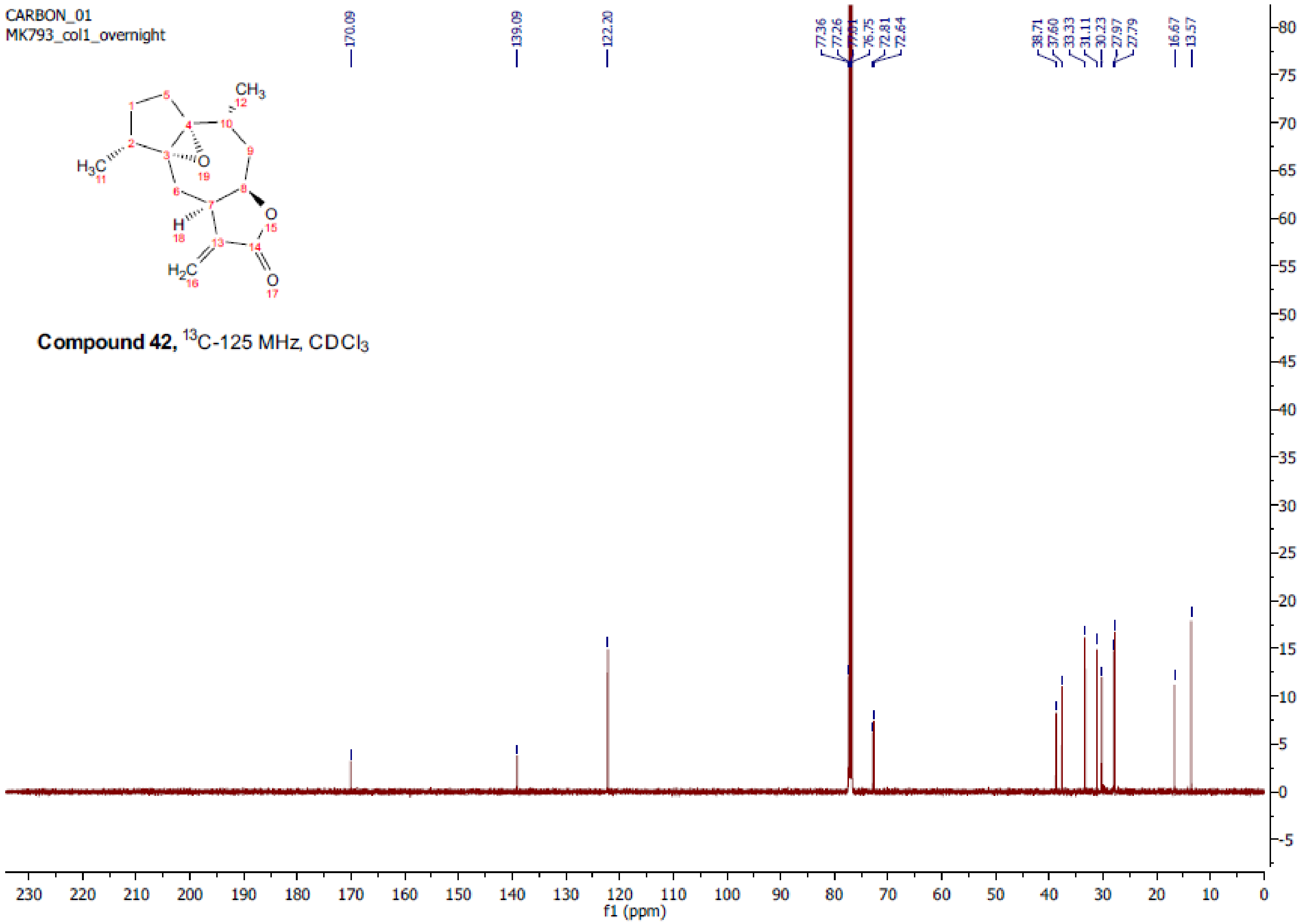
CARBON_01
MK793_col1_overnight
Compound 42, 13C-125 MHz, CDCl3
170.09
139.09
122.20
77.36
77.26
77.01
76.75
72.81
72.64
38.71
37.60
33.33
31.11
30.23
27.97
27.79
16.67
13.57
f1 (ppm)

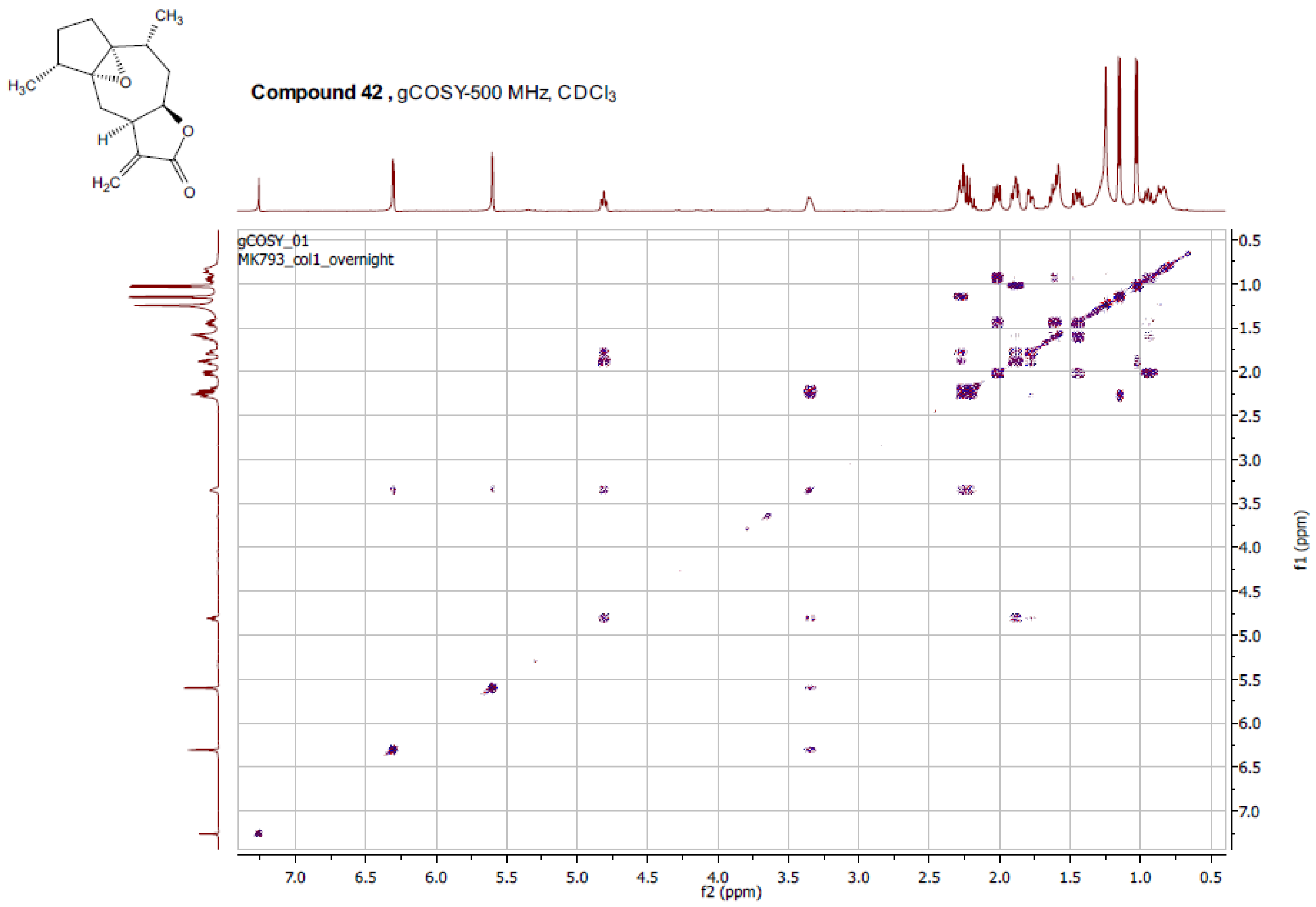

Compound 42 , gCOSY-500 MHz, $CDCl_3$
CH_3
H_3C
O
H
H_2C
gCOSY_01
MK793_col1_overnight
f2 (ppm)
f1 (ppm)

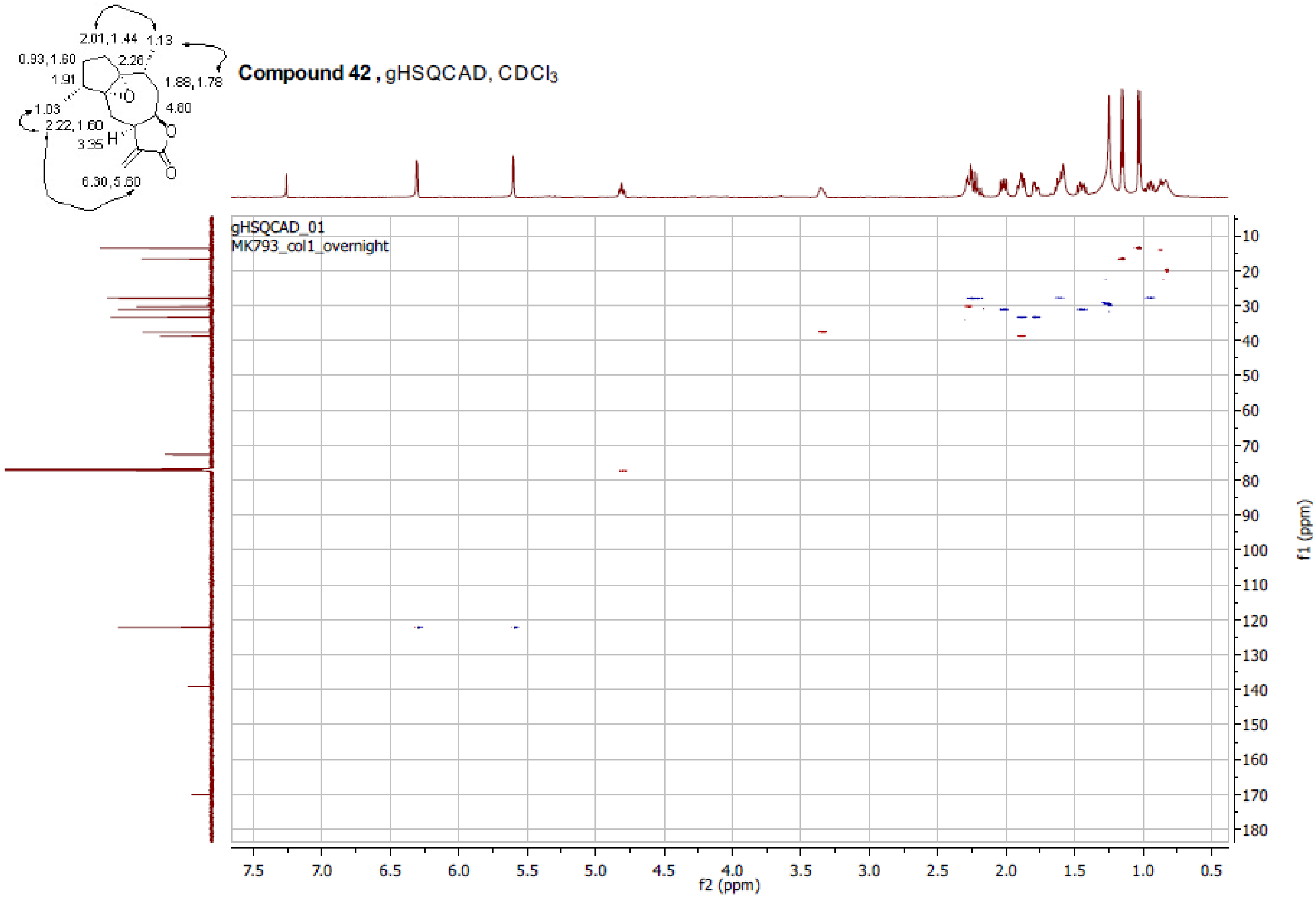

Compound 42 , gHSQCAD, $CDCl_3$
2.01,1.44
1.13
0.93,1.60
2.26
1.91
1.88,1.78
1.03
4.60
2.22,1.60
3.35
6.30,5.60
gHSQCAD_01
MK793_col1_overnight
f2 (ppm)
f1 (ppm)

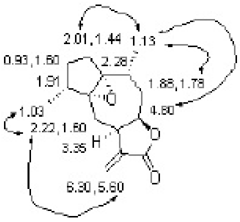

Compound 42, NOESY-500 MHz, $CDCl_3$

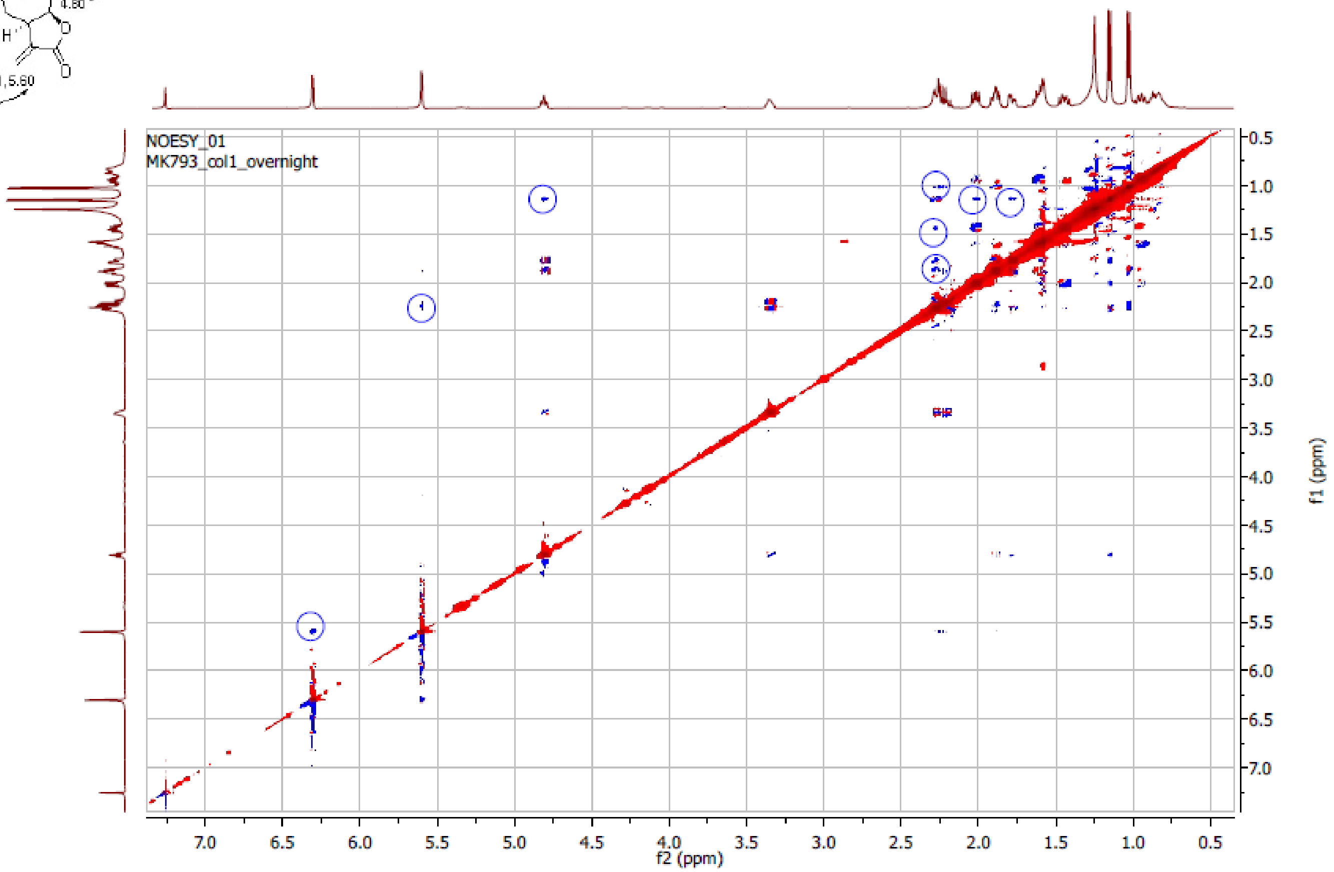

8. References

[1] A. C. Beekman, H.J. Woerdenbag, W. van Uden, N. Pras, A. W. T. Konings, H. V. Wirkstrom, T. J. Schmidt, *J. Nat. Prod.*, **1997**, *60*, 252-257.

[2]M. Kourgiantaki, V. P. Demertzidou, A. L. Zografos, *Org. lett.*, **2022**, 24, 8476-8480.

[3] S. G. Hegde, M. K. Vogel, J. Saddlerqq, T. Hrinyo, N. Rockwell, R. Haynes, M. Oliver, J. Wolinsky, *Tetr. Lett.* **1980**, *21*, 441-444.

[4] M. Xuan, I. Paterson, S. M. Dalby, *Org. Lett.*, **2012**, *14*, 5492-5495.

[5] a) K. E. Harding, L. M. May, K. F. Dick, *J. Org. Chem.* **1975**, *40, 11,* 1664-1665 b) X. Jiang, J. Zhang, S. Ma, *J. Am. Chem. Soc.* **2016**, 138, 8344-8347 c) J. M. Hoover, S. S. Stahl, *J. Am. Chem. Soc.* **2011**, *133*, 16901-16910.

[6] N. Kornblum, W. J. Jones, G. J. Anderson, *J. Am. Chem. Soc.* **2059**, *81*, 4113-4114.

[7] B. O. Lindgren, T. Nilsson, *Acta Chemica Scandinavica* **1973**, *27*, 888-890.

[8] B. Maurer, A. Hauser, J.-C. Froidevaux, *HELVETICCA HIMICA ACTA* **1989**, *72*, 1400-1415.

[9] M. Yang, X. Jiang, W.-J. Shi, Q.-L. Zhu, Z.-J. Shi, *Org. Lett*. **2013**, *15*, 690-693.

[10] A. L. García-Cabeza, R. Marín-Barrios, R. Azarken, F. J. Moreno-Dorado, M. J. Ortega, H. Vidal, J. M. Gatica, G. M. Massanet, F. M. Guerra, *Eur. J. Org. Chem.* **2013**, 8307–8314.

[11] J. G. Hubert, D. P. Furkert, M. A. Brimble, *J. Org. Chem.* **2015**, *80*, 2231–2239

[12] S. Sathyamoorthi, J. Du Bois, *Org. Lett*. **2016**, *18*, 6308-6311.

[13] H. L. Riley, J. F. Morley, N. A. C. Friend, *J. Chem. Soc.* **1932**, 1875-1883.

[14] J. Conception, C. G. Francisco, R. Hernandez, J. A. Salazar, E. Suarez, *Tetr. Lett.* **1984**, *25*, 1953-1956.

[15] X. Huang, R. Goddard, N. Maulide, *Angew. Chem. Int. Ed.* **2010**, *49*, 8979-8983.

[16] N. C. Wilde, M. Isomura, A. Mendoza, P. S. Baran, *J. Am. Chem. Soc.* **2014**, 136,4909-4912.

[17] S. Isayama, T. Mukaiyama, *Chemistry Letters*, **1989**, 18, 569-572.

[18] S. Isayama, T. Mukaiyama, *Chemistry Letters*, **1989**, 18, 573-576.

[19] S.M. Kupchan, D. C. Fessler, M. A. Eakin, T. J. Giacobbe, *Science*, **1970**, *168*, 376-378.

[20] X. Hu, A. J. Musacchio, X. Shen, Y. Tao, T. J. Maimone, *J. Am. Chem. Soc.*, **2019**,141, 14904-14915.
